# Genome-wide association mapping combined with gene-based haplotype analysis identify a novel gene for shoot length in rice (*Oryza sativa* L.)

**DOI:** 10.1007/s00122-023-04497-6

**Published:** 2023-11-20

**Authors:** Tifeng Yang, Jingfang Dong, Junliang Zhao, Longting Zhang, Lian Zhou, Wu Yang, Yamei Ma, Jian Wang, Hua Fu, Jiansong Chen, Wenhui Li, Haifei Hu, Xianya Jiang, Ziqiang Liu, Bin Liu, Shaohong Zhang

**Affiliations:** 1 Rice Research Institute, Guangdong Key Laboratory of New Technology in Rice Breeding, Guangdong Rice Engineering Laboratory, Key Laboratory of Genetics and Breeding of High Quality Rice in Southern China (Co-Construction By Ministry and Province), Ministry of Agriculture and Rural Affairs, Guangdong Academy of Agricultural Sciences, Guangzhou, 510640 China; 2 College of Agriculture, South China Agricultural University, Guangzhou, 510642 China; 3 Yangjiang Institute of Agricultural Science, Yangjiang, 529500 China

## Abstract

**Key message:**

Genome-wide association mapping revealed a novel QTL for shoot length across multiple environments. Its causal gene, *LOC_Os01g68500*, was identified firstly through gene-based haplotype analysis, gene expression and knockout transgenic verification.

**Abstract:**

Strong seedling vigor is an important breeding target for rice varieties used in direct seeding. Shoot length (SL) is one of the important traits associated with seedling vigor characterized by rapid growth of seedling, which enhance seedling emergence. Therefore, mining genes for SL and conducting molecular breeding help to develop varieties for direct seeding. However, few QTLs for SL have been fine mapped or cloned so far. In this study, a genome-wide association study of SL was performed in a diverse rice collection consisting of 391 accessions in two years, using phenotypes generated by different cultivation methods according to the production practice, and a total of twenty-four QTLs for SL were identified. Among them, the novel QTL *qSL-1f* on chromosome 1 could be stably detected across all three cultivation methods in the whole population and *indica* subpopulation. Through gene-based haplotype analysis of the annotated genes within the putative region of *qSL-1f*, and validated by gene expression and knockout transgenic experiments, *LOC_Os01g68500* (i.e., *Os01g0913100* in RAP-DB) was identified as the causal gene for SL, which has a single-base variation (C-to-A transversion) in its CDS region, resulting in the significant difference in SL of rice. *LOC_Os01g68500* encodes a DUF538 (Domain of unknown function) containing protein, and the function of DUF538 protein gene on rice seedling growth is firstly reported in this study. These results provide a new clue for exploring the molecular mechanism regulating SL, and promising gene source for the molecular breeding in rice.

**Supplementary Information:**

The online version contains supplementary material available at 10.1007/s00122-023-04497-6.

## Background

Rice is the staple foods for more than half of the world population, and rice production plays a critical role in world food security. However, increasing threats to rice production from labor shortage, energy scarcity, decline of water table and change in climatic conditions drive to search for an alternative cultivation system to traditional puddle transplanting (Singh et al. 2013). Being a light and simple, laborsaving and efficient cultivation technique, direct seeding has been adopted in many countries and will become an inevitable trend in rice-growing areas. Two major methods, wet seeding (pre-germinated seeds are broadcasted on the field) and dry seeding (dry seeds are broadcasted on the field) are popular in rice direct seeding systems in response to increased costs and reduced labor and water (Mahender et al. 2015; Sha et al. 2019). But due to the absence of standing water layer at seedling emergence, the infestation of weeds has become one of the major constraints to the grain yield of direct seeding rice and the popularization of direct seeding in rice (Chauhan 2013; Mahender et al. 2015; Dimaano et al. 2020). At present, the application of chemical herbicides is the important way to control weeds in direct seeding fields because of saving manpower and reducing labor cost (Liu et al. 2014; Chauhan et al. 2015). However, the use of herbicides not only increases the cost of rice production, but also causes environmental pollution.

Crop plant with strong vigor outgrows the weed plant and suppresses its growth. Rice seedling vigor determines rapid and uniform emergence, as well as seedling development, which is an imperative trait for the stable seedling establishment and enhancing the competitiveness against weeds in rice direct seeding system (Zhang et al. 2005; Mahender et al. 2015). Therefore, development and use of rice varieties with strong seedling vigor is the most economical and environmentally friendly way to control weeds. However, the previous studies suggest that seedling vigor is a complex trait controlled by multiple genes (Zhang et al. 2005; Sandhu et al. 2015; Yang et al. 2021; Zeng et al. 2021). It is difficult to develop rice variety with strong seedling vigor using the conventional breeding methods. Understanding its genetic basis is the key for molecular breeding in rice with strong seedling vigor.

Among the traits associated with seedling vigor in rice, shoot length (SL)/seedling height is one of the important traits characterized by rapid growth of seedling, which can enhance seedling emergence (Abe et al. 2012; Lu et al. 2016). Rapid and vigorous growth of seedlings helps to improve their advantage in competition of nutrient and light energy, and effectively suppress the growth of weeds (Rao et al. 2007; Singh et al. 2017; Dimaano et al. 2020). However, the modern rice varieties have been changed into adaptable for transplanting and semi-dwarf architecture, which is not amenable to direct seeding conditions (Mahender et al. 2015). Therefore, mining genes associated with SL and conducting molecular breeding can facilitate developing rice varieties for direct seeding. With the rapid development of molecular marker technology and genome sequencing technology, numerous QTLs for SL have been identified using bi-parental QTL analysis (Redona et al. 1996; Zhang et al. 2005; Lu et al. 2007; Zhou et al. 2007; Cairns et al. 2009; Abe et al. 2012; Yano et al. 2012; Diwan et al. 2013; Sandhu et al. 2015; Cordero-Lara et al. 2016; Singh et al. 2017; Zhang et al. 2017; Dimaano et al. 2020; Yang et al. 2021) and genome-wide association study (GWAS) in diverse natural populations (Dang et al. 2014; Anandan et al. 2016; Lu et al. 2016; Chen et al. 2019; Zhao et al. 2019; Zeng et al. 2021; Ma et al. 2022). Among these studies, Abe et al. (2012) identified four QTLs for seedling height of 14-days seedling, and Yano et al. (2012) identified two QTLs for seedling height of 30-days seedling. They both reported that *OsGA20ox1* related to gibberellin biosynthesis was the candidate and functional gene underlying *qPHS3-2* (Abe et al. 2012) and *qEPD2* (Yano et al. 2012) on chromosome 3, respectively.

Although more than 100 QTLs for seedling vigor in rice have been identified and mapped, these QTLs have not been effectively used in rice breeding. The uncertainty of these QTLs could be one of the main reasons. Before marker-assisted selection can be effectively deployed, the QTLs should be validated, fine mapped or even cloned. However, most of the QTLs for seedling vigor in rice have not been validated and few of their functional genes have been identified except *OsGA20ox1* for SL (Abe et al. 2012; Yano et al. 2012) and *SBM1* for the seedling biomass (Xu et al. 2021). More importantly, rice seedling vigor is a complex trait controlled by multiple genes, and most of these genes/QTLs have minor effects and their expression are frequently affected by environments (Zhang et al. 2005; Zhou et al. 2007; Cordero-Lara et al. 2016), but most of the reported QTLs were identified in a single environment, and their reliability and stability remain unclear. Therefore, identifying the stably expressed QTLs and cloning their functional genes will be pivotal to rice breeding for improving seedling vigor.

In this study, we conducted GWAS of SL using a subset of the Rice Diversity Panel 2 (sRDP2) consisting of 391 rice accessions, which has been genotyped using 700 K SNPs (McCouch et al. 2016). Considering the diversity of environment and methods in rice direct seeding, the SL was measured using three cultivation methods according to the production practice of wet and dry seeding. In total, twenty-four QTLs for SL were identified, among which *qSL-1f* on chromosome 1 was the novel and could be stably detected under the three cultivation methods in the whole population and *indica* subpopulation. Through gene-based haplotype analysis of the annotated genes within the *qSL-1f* region, and validated by gene expression and knockout transgenic experiments, *LOC_Os01g68500* (*Os01g0913100*), encoding a DUF538 (Domain of unknown function) containing protein was identified as the causal gene underlying *qSL-1f*, which controlled SL at the seedling stage. Sequence analysis revealed that a single-base transversion (C-to-A) in its CDS region caused the significant difference in SL. To our knowledge, it is the first time to discover the function of DUF538 protein gene on rice seedling growth. These results provide a new clue for exploring the molecular mechanism regulating SL and promising gene source for the molecular breeding of rice with strong seedling vigor.

## Materials and methods

### Plant materials

A subset of the RDP2 (sRDP2) consisting of 391 rice accessions from 56 countries were used for GWAS in this study (Table S1). These rice accessions were selected from the Rice Diversity Panel 2 (RDP2) consisting of 1568 rice accessions based on their diversity and origins (McCouch et al. 2016). All seeds used in this study were stored at room temperature for three months after harvested.

### Evaluation of shoot length

For GWAS population, the healthy and filled seeds were incubated at 49 °C for 96 h to break dormancy. After sterilized with 3% sodium hypochlorite solution, the seeds were soaked in distilled water for 24 h, and sown using three cultivation methods according to the production practice. Method 1 (GST): the seeds were pre-germinated and sown in plastic trays (35.0 cm × 23.0 cm × 6.0 cm); Method 2 (GSF): the seeds were pre-germinated and sown in the paddy field; Method 3 (DST): the seeds were directly sown in plastic trays without pre-germination. GST were conducted in May 2018, while GSF and DST were conducted between late April and early May 2019. For GST and GSF, the soaked seeds were placed in a 7 cm petri dish with two layers of wet filter paper, and put into a growth chamber with 30 °C and relative humidity of 70% for germination in dark. After 2 days, the germinating seeds were sown into plastic trays filled with fine soil (GST in 2018) or in paddy field (GSF in 2019). For DST, the seeds were directly sown into plastic trays filled with fine soil without pre-germination. After 14 days of growth in natural environment, the shoot length (SL) of seedling were measured with a ruler. In this study, three replicates with 10 plants per accession were adopted in SL evaluation, and the average air temperature was 28.7 °C, 23.6 °C and 24.1 °C under GST, GSF and DST, respectively.

For knockout transgenic (KO) lines, the healthy and filled seeds of the KO lines and their wild-type line were incubated at 49 °C for 96 h to break dormancy. After sterilized with 3% sodium hypochlorite solution, the seeds were soaked in distilled water for 24 h. The pre-germinated seeds were sown in black plastic culture boxes (12 cm × 8.6 cm × 11 cm) filled with 0.1% Yoshida nutrient solution (July 2022) or fine soil (June 2023), then put into a growth chamber with 30 °C and relative humidity of 70% for growth (12 h light/12 h dark). After 14 days, the SL of seedling were measured. Four replicates with 20 plants per lines were adopted in SL evaluation.

### GWAS of sRDP2 and QTL delimitation

GWAS analysis was performed as described in our previous study by using software GAPIT version 2 and HDRA dataset consisting of 700 K single-nucleotide polymorphisms (SNPs) (Zhao et al. 2018; Yang et al. 2020). SNPs were filtered by the criteria of having less than 30% missing data and minor allele frequency (MAF) > 0.05. In order to reduce the effect of population structure on GWAS, the mixed linear model (MLM) was selected in which the kinship matrix was used jointly with PC in GAPIT, and three PCs were included when analyzing the whole population consisting of *indica*, *japonica* and *aus* (Yang et al. 2020). Manhattan plots were produced using R package qqman. A region having three or more than three significant SNPs (*P* < 0.0001) (Shakiba et al. 2017; Wang et al. 2018; Jiang et al. 2019) within 200 kb is considered as one QTL (Zhao et al. 2018; Yang et al. 2020).

### DNA sequence analysis

A total of 343 rice accessions were re-sequenced using the Illumina NovaSeq6000 platform, and sequencing data analysis was performed using the pipeline from our previous study (Wang et al. 2023; Yang et al. 2023). Firstly, the adaptor sequences and low-quality sequence reads were removed from the data sets, and raw sequences were transformed into clean reads after data processing. Next, the clean reads were mapped to the reference genome sequence and only reads with a perfect match or one mismatch were further analyzed and annotated based on the Nipponbare IRGSP1.0 genome using Hisat2 tools soft. Finally, GATK software was used to detect SNP and Indel (≦ 50 bp) and PAVs (the presence/absence variation > 50 bp) were called based on the mapping coverage of sequencing reads to the pangenome for each accession. All raw sequence data have been deposited in the NCBI sequence read archive (BioProject accession PRJNA820969).

### Haplotype analysis and candidate gene identification

For QTL haplotype analysis, three significant SNPs including the peak SNP within the QTL interval were used for analysis. For gene-based haplotype analysis, the indel, SNP and PAV within the QTL interval were firstly analyzed with Nipponbare as the reference genome using the re-sequencing information (50×) of 343 rice accessions (Yang et al. 2023; Wang et al. 2023). Secondly, all annotated genes within the QTL interval were performed to identify their haplotypes based on their sequence variations, respectively. Finally, the accessions were grouped based on the haplotypes of each gene and performed the significance test of the differences in SL among the major haplotypes (containing more than 10 accessions). A gene was considered to be a candidate gene if the significant differences in SLs were observed among haplotypes of a gene under all cultivation environments.

### Gene differential expression analysis by qRT-PCR

Five rice accessions with long SL and five rice accessions with short SL were selected for differential expression analysis of candidate genes based on the haplotype analysis. The germinating seeds were sown into trays filled with fine soil as described above. Sampling was conducted on the 3rd, 6th and 9th d after sowing, respectively, with three biological replications. Total RNA was extracted from shoots using Trizol reagent (Invitrogen, Carlsbad, CA, USA) and purified using RNeasy Plant Mini Kit (Qiagen, Valencia, CA).

RNA reverse transcription reactions were performed using the Prime Script TM RT reagent kit (Takara, Japan). The primers for qRT-PCR were designed by Primer Premier 5.0. Quantitative real-time PCR was carried out using the SYBR Premix ExTaqTM kit (Takara, Japan), following the manufacturer’s instructions, on a BioRad CFX 96 Real-Time System. The *actin* was used as endogenous normalized genes for mRNA. All reactions were run in triplicate. Primers used to amplify the selected genes are listed in Table S2.

### Validation of candidate genes for shoot length

In order to validate the candidate gene for SL, we conducted the knockout transgenic experiments. To generate the CRISPR/Cas9 vectors, *LOC_Os01g68460* and *LOC_Os01g68500* single-guide RNA (sgRNA) sequences were cloned using pYLgRNA-OsU3, respectively, as described previously (Ma et al. 2015). The target site sequence of *LOC_Os01g68460* was 5′-GCTGGCACGCGAGTGTTGAG-3′, which contained a protospacer adjacent motif (PAM) AGG at the 3′ end; the target site sequence of *LOC_Os01g68500* was 5′-CGGATTTCAGGCACGACGAA-3′, which contained a PAM of GGG at the 3′ end. The positive plasmids were electroporated into *Agrobacterium tumefaciens* EHA105 and then introduced into calli of the cultivar Nipponbare via Agrobacterium-mediated genetic transformation, respectively.

At the T_2_ generation, the homozygous positive transgenic plants of the candidate genes were selected by gene cloning and sequencing. The seeds of the homozygous positive plants were used to evaluate SL as described above, and the wild-type plants (Nipponbare) were used as control.

### Data analysis

A *t*-test or Duncan’s multiple range test was conducted using *SPSS*10.0 to detect the differences in SL and expression levels of the candidate gene between or among the tested rice accessions.

## Results

### Variations of shoot length in sRDP2 population under the three cultivation methods

**Fig. 1:**
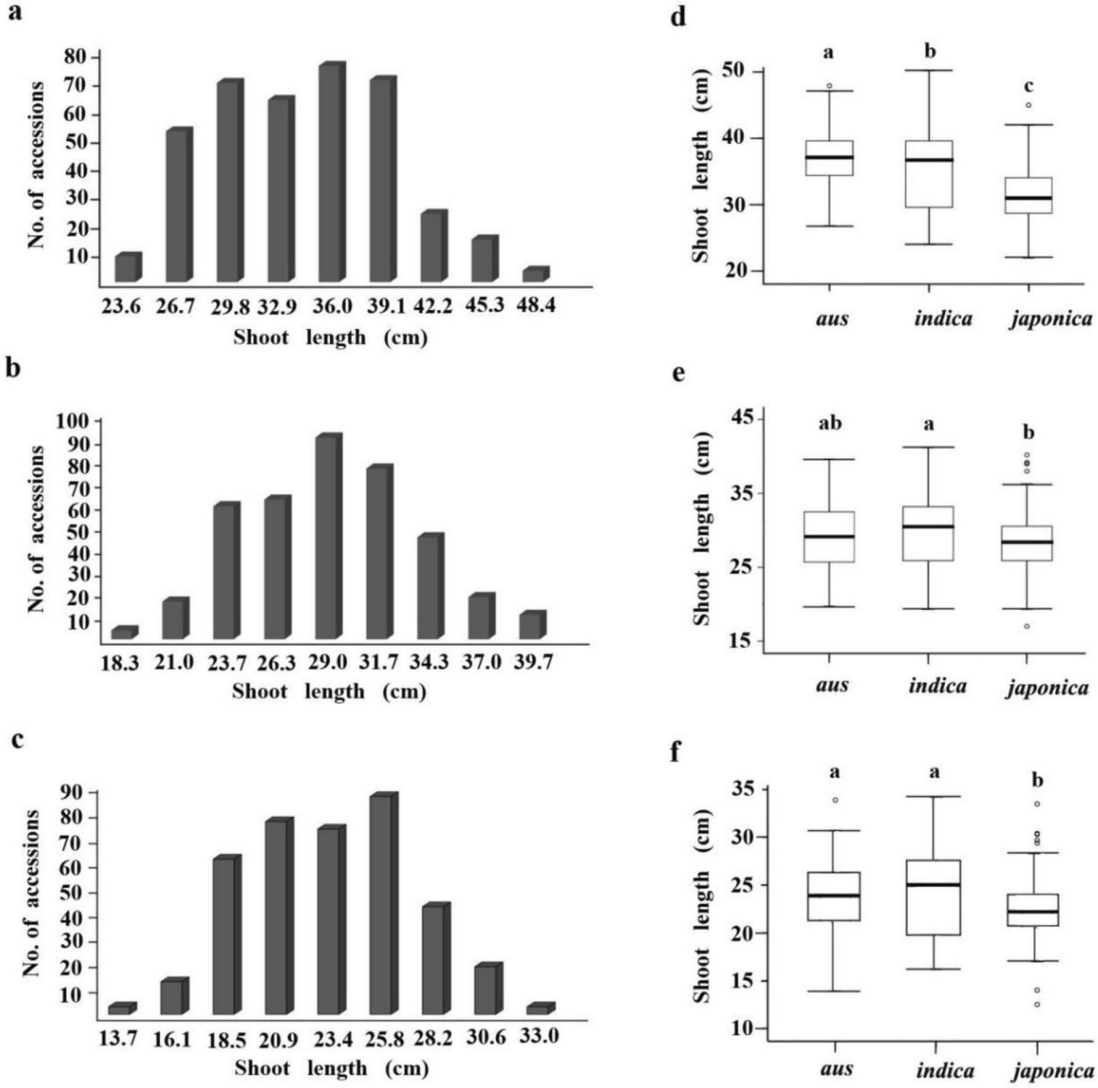
Distribution and variations of shoot length in the 391 rice accessions under three cultivation methods. **a**–**c**, Distribution of shoot length in the 391 rice accessions under the cultivation method of GST (**a**), GSF (**b**) and DST (**c**). **d**–**f** Boxplots of the shoot length variation in the three subpopulations under the cultivation method of GST (**d**), GSF (**e**) and DST (**f**). The black horizontal lines represent the median value; the upper side and lower side of the box represent the upper quartile and lower quartile, respectively; the whiskers represent the range of data, and small circles represent outliers. The same letter upon the boxplot means no significant difference in shoot length at *P* = 0.05, based on Duncan’s multiple range test

Large variations in SL were observed in sRDP2 population using the three cultivation methods (Table S1), ranging from 22.0 to 50.2 cm, with an average of 34.3 cm and a variation coefficient of 16.3% under the GST; from 17.0 to 41.2 cm, with an average of 29.2 cm and a variation coefficient of 15.4% under the GSF; from 12.5 to 34.2 cm, with an average of 23.3 cm and a variation coefficient of 16.3% under the DST. The SL in 391 rice accessions displayed a continuous and normal distribution (Figs. 1a–c), suggesting quantitative inheritance and the involvement of multiple genes for SL.

The phenotype comparisons among different groups revealed that the SL of *indica* group was significantly higher than that of *japonica* group (*P* < 0.05) under all of the three cultivation methods; while the SL of *aus* group was significantly higher than that of *japonica* group under the GST and DST (*P* < 0.05) and *indica* group under GST (*P* < 0.05), but not significantly different from that of *indica* group under GSF and DST, and *japonica* group under GSF (*P* > 0.05) (Fig. 1d–f).

The pairwise correlations of SL between different cultivation methods were highly correlated (*P* < 0.0001), with correlation coefficients of 0.726, 0.769 and 0.787 between GST and GSF, GST and DST, GSF and DST, respectively, suggesting there were stably QTLs associated with SL.

### Mapping QTLs for shoot length through GWAS under the three cultivation methods

**Fig. 2:**
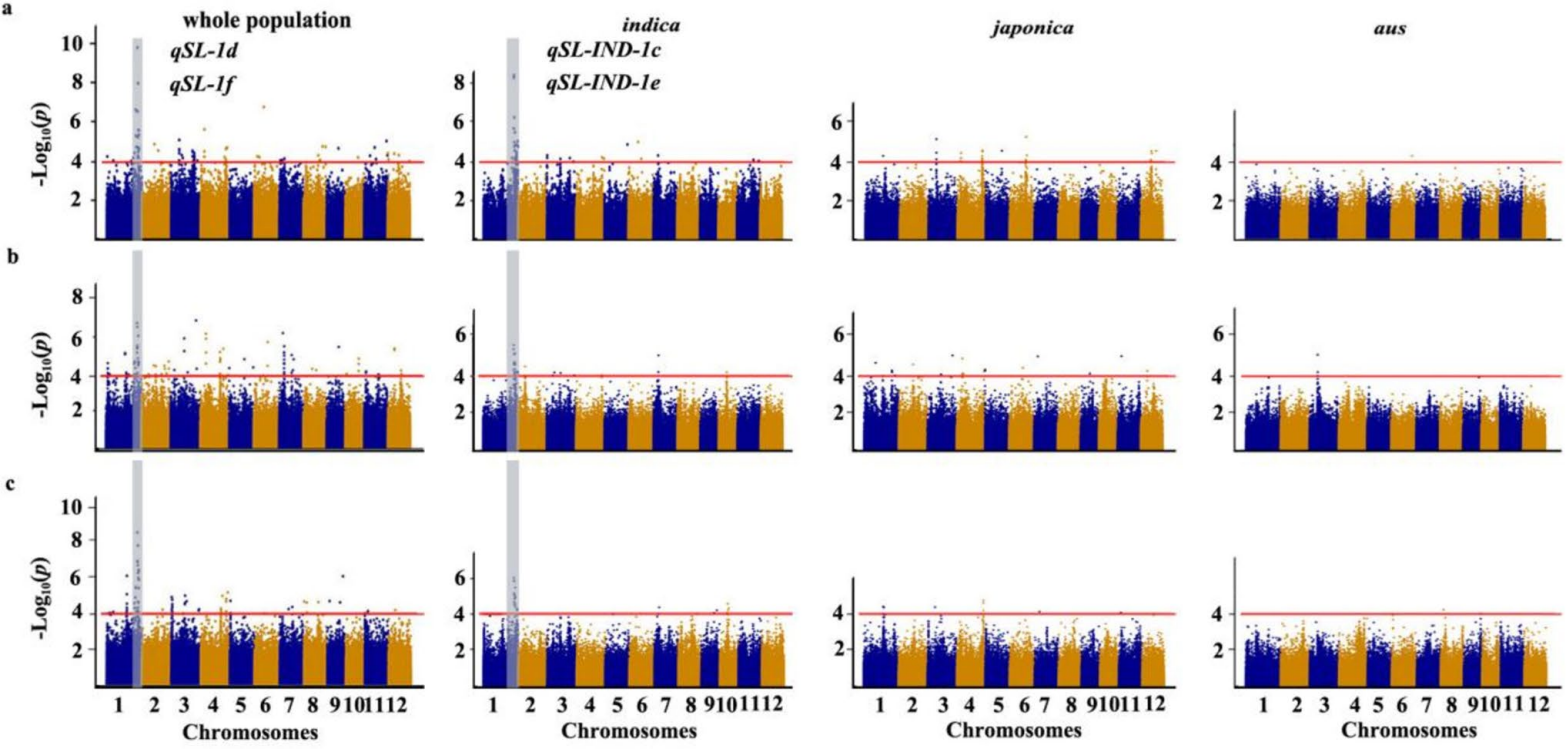
Genome-wide association study of shoot length in the whole population and three sub-populations using the three cultivation methods. **a**–**c** Manhattan plots of GWAS for shoot length in 12 chromosomes under GST (**a**), GSF (**b**) and DST (**c**)

Based on the criteria of having less than 30% missing data and minor allele frequency (MAF) more than 5% in the sRDP2 population, 446,536 SNPs were selected for GWAS from the 700 K SNPs dataset in the Open Rice GWAS Platform (McCouch et al. 2016). Population structure of the sRDP2 estimated by Admixture software, principal component analysis and kinship analysis suggested that there were three subpopulations in this panel, which are consistent to *indica*, *japonica* and *aus* subpopulation (Yang et al. 2020). Therefore, GWAS of SL was conducted in the whole population, *indica*, *japonica* and *aus* subpopulation, respectively, using phenotypes generated under the three cultivation methods. Based on about 100 kb of the linkage disequilibrium (LD) decay in tested rice accessions (Yang et al. 2020), we delimited a QTL to a 200 kb region with center on the most significant SNP, in which there are three or more than three significant SNPs (*P* < 0.0001) (Yang et al. 2020). Accordingly, a total of twenty-four QTLs for SL were identified in the present study (Fig. 2 and Table 1).

**Table1:** QTLs for shoot length identified using the three cultivation methods and their co-location QTLs identified in the previous studies

QTL	Chr	GST			GSF			DST			Co-location QTL^c^	Reference
		Position (bp)^a^	*P* value	PVE (%)^b^	Position (bp)	*P* value	PVE (%)	Position (bp)	*P* value	PVE (%)		
whole												
*qSL-1a*	1				2,263,294	1.96E−05	2.95					
*qSL-1b*	1							25,002,596	9.27E−07	4.27		
*qSL-1c*	1				37,933,570	1.24E−07	4.59	37,933,570	3.78E−09	6.24		
***qSL-1d***	1	38,538,838	1.10E−08	4.45	38,538,838	2.02E−07	4.43	38,538,838	1.96E−08	5.65	*qSSL1b*	Zhang et al. (2017)
*qSL-1e*	1							39,032,129	1.20E−06	4.18		
***qSL-1f***	1	39,621,602	2.83E−06	2.94	39,588,846	6.24E−07	4.06	39,588,846	4.35E−07	4.53		
*qSL-3a*	3							1,275,175	1.90E−05	3.22	*qPHS 3-1*	Abe et al. (2012)
*qSL-3b*	3	10,131,706	8.67E−06	2.65								
*qSL-3c*	3	26,463,704	3.11E−05	2.32								
*qSL-3d*	3	28,623,558	4.05E−05	2.25							*qSEV-3-3*	Lu et al. (2007)
*qSL-4a*	4	4,588,660	2.47E−06	2.98								
*qSL-4b*	4				7,108,227	4.79E−07	4.15					
*qSL-4c*	4				24,152,902	4.85E−06	3.4				Loci 97	Zeng et al. (2021)
*qSL-4d*	4				24,578,040	1.05E−05	3.15					
*qSL-4e*	4	31,156,462	2.41E−05	2.38				31,159,946	1.72E−05	3.25	*qPHS4*	Abe et al. (2012)
											*qSSL4*	Zhang et al. (2017)
*qSL-7a*	7				6,342,507	2.33E−06	3.63					
*qSL-7b*	7				15,418,806	7.17E−06	3.27					
*qSL-10*	10				16,580,444	1.13E−05	3.13					
*indica*												
*qSL_IND-1a*	1	37,933,570	6.02E−09	11.54				37,933,570	1.39E−06	7.69		
*qSL_IND-1b*	1				38,112,582	2.81E−06	6.68					
***qSL_IND-1c***	1	38,538,838	6.18E−07	8.22	38,538,838	8.39E−06	6.00	38,538,838	2.10E−06	7.41	*qSSL1b*	Zhang et al. (2017)
*qSL_IND-1d*	1				39,285,177	2.20E−05	5.41					
***qSL_IND-1e***	1	39,621,602	1.56E−05	6.05	39,621,602	7.62E−06	6.06	39,621,602	4.60E−06	6.88		
*qSL_IND-1f*	1	41,260,184	3.45E−05	5.53							seq-rs609	Lu et al. (2016)
*japonica*												
*qSL_JAP-3*	3	10,131,706	7.79E−06	11.11								
*qSL_JAP-4a*	4	4,588,660	3.89E−05	9.31								
*qSL_JAP-4b*	4				7,108,227	1.29E−05	10.12					
*qSL_JAP-4c*	4	31,300,240	2.91E−05	9.63							*qPHS4*	Abe et al. (2012)
											*qSSL4*	Zhang et al. (2017)
*qSL_JAP-4d*	4							33,106,021	2.17E−05	12.01		
*aus*												
*qSL_AUS-3*	3				10,219,707	7.14E−06	12.63					

*GST* pre-germinated seeds were sown in plastic trays, *GSF* pre-germinated seeds were sown in the paddy field; *DST* seeds were directly sown in plastic trays without pre-germination

^a^Position of the most significant SNP at the QTL region

^b^Percentage of variance explained

^c^QTLs for shoot length identified by the previous studies

In the whole population, eighteen QTLs located on chromosome 1, 3, 4, 7 and 10 were identified under three cultivation methods (Fig. 2 and Table 1). Among them, *qSL-1d* and *qSL-1f* on chromosome 1 could be identified under all three cultivation methods, *qSL-1c* on chromosome 1 and *qSL-4e* on chromosome 4 could be identified under two cultivation methods, while the other 14 QTLs could only be identified under one cultivation method. Moreover, six QTLs located on chromosome 1 were identified in *indica* subpopulation, in which *qSL_IND-1c* and *qSL_IND-1e* could be identified under all three cultivation methods, *qSL_IND-1a* could be identified under two cultivation methods; while five QTLs on chromosome 3 and 4, and one QTL on chromosome 4 were identified in the *japonica* and *aus* subpopulation, respectively, which could only be identified under one cultivation method (Table 1).

Comparisons of the QTLs identified in different populations indicated that *qSL_IND-1a, qSL_IND-1c* and *qSL_IND-1e* from the *indica* subpopulation overlapped with *qSL-1c, qSL-1d* and *qSL-1f* identified in the whole population, respectively, while *qSL_JAP-3, qSL_JAP-4a* and *qSL_JAP-4b* from the *japonica* subpopulation overlapped with *qSL-3b, qSL-4a* and *qSL-4b* identified in the whole population, respectively.

Among the twenty-four QTLs identified in the present study, eighteen QTLs (*qSL-1a, qSL-1b*, *qSL-1c/qSL_IND-1a*, *qSL-1e, qSL-1f/qSL_IND-1e*, *qSL-3b/qSL_JAP-3*, *qSL-3c*, *qSL-4a/qSL_JAP-4a*, *qSL-4b/qSL_JAP-4b*, *qSL-4d, qSL-7a, qSL-7b, qSL-10*, *qSL_AUS-3*, *qSL_JAP-4c*, *qSL_JAP-4d*, *qSL_IND-1b* and *qSL_IND-1d*) are firstly reported in the present study, and the other six QTLs co-localized with the previously identified SL QTLs (Table 1). Particularly, *qSL-1d* and *qSL-1f* could be stably detected using the three cultivation methods in the whole population and *indica* subpopulation, and had larger contribution to SL variation in 391 rice accessions. The *qSL-1f* was identified firstly in the present study, while *qSL-1d* co-localized with the previously identified QTL for SL (Zhang et al. 2017).

## Haplotype analysis of *qSL-1f*

**Fig. 3:**
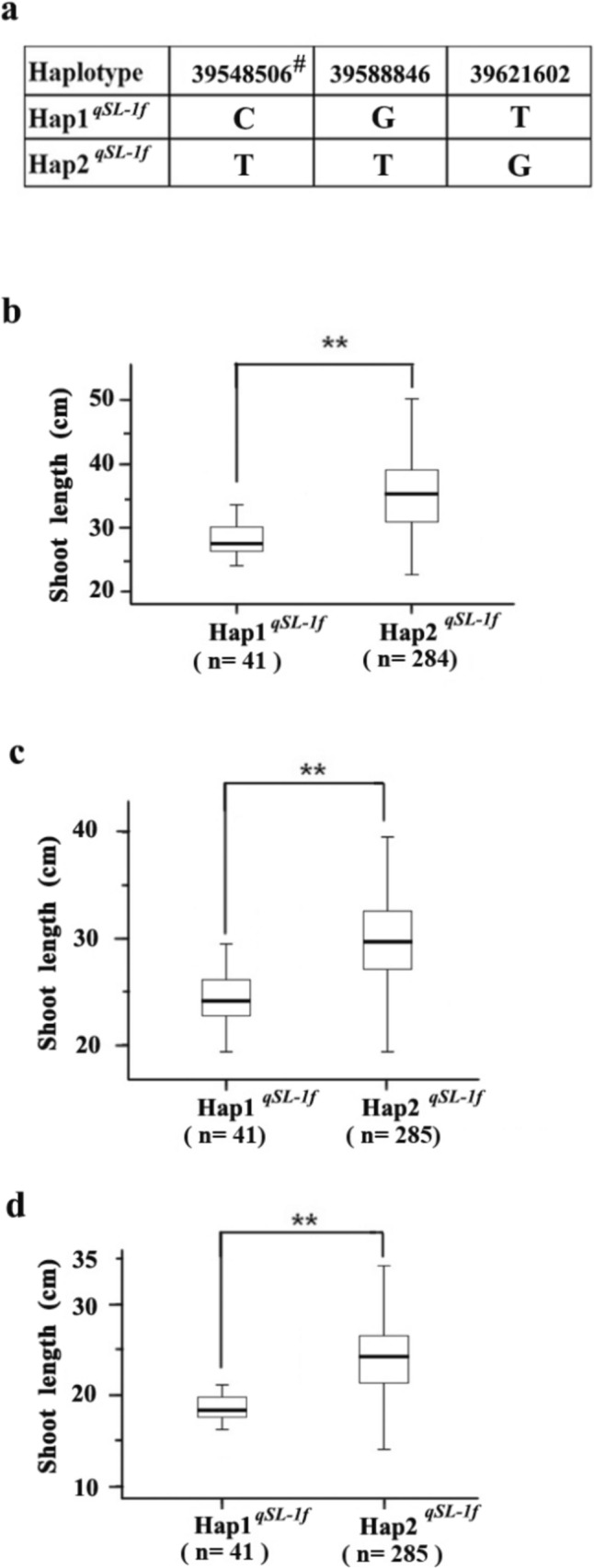
Differences in shoot length between the main haplotypes of *qSL-1f*. **a** The main haplotypes of *qSL-1f*. ^#^The SNP position (bp); **b**–**d** Boxplots for shoot length based on the haplotypes of *qSL-1f* under GST (**b**), GSF (**c**) and DST (**d**). Numbers in parenthesis indicate the number of rice accessions with the haplotype. The black horizontal lines represent the median value; the upper side and lower side of the box represent the upper quartile and lower quartile, respectively; the whiskers represent the range of data. *Double asterisk* indicates the significant difference in shoot length at *P* = 0.01 based on *t*-test

Being the novel and detectable in different populations and cultivation regimes, it is believed that *qSL-1f* is stably expressed QTL for SL and has great potential value in rice breeding for improving seedling vigor. To search for the favorable haplotype, haplotype analysis was performed based on the three significant SNPs within the *qSL-1f* interval, and two main haplotypes were identified (Fig. 3a).

Significant differences in SL were observed between the two main haplotypes of *qSL-1f* (Fig. 3b–d) under the three cultivation methods (*P* < 0.01). The average SLs of the lines carrying Hap2^*qSL−1f*^ were 35.3, 29.7 and 24.1 cm, which were significantly longer than that of the lines carrying Hap1^*qSL−1f*^, with the average SLs of 29.0, 24.5 and 19.2 cm under the cultivation method of GST, GSF and DST, respectively.

### Candidate genes analysis of* qSL-1f*

**Fig. 4:**
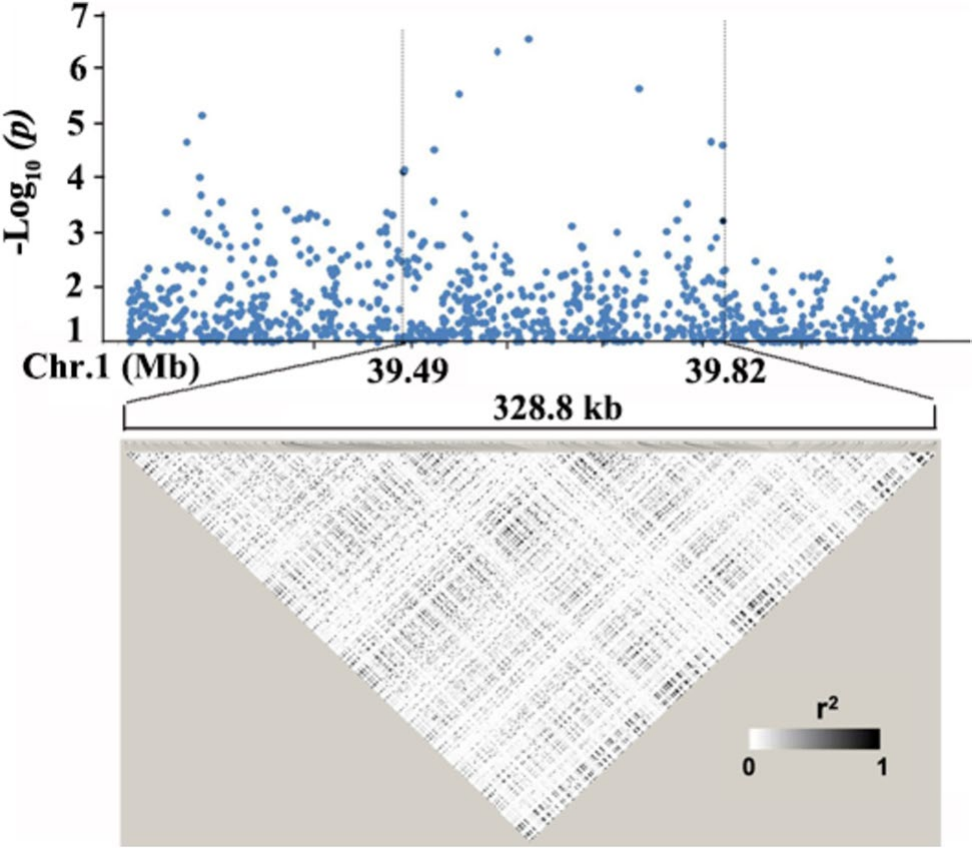
Candidate region of *qSL-1f* on chromosome 1. Local Manhattan plot (top) and LD heat map (bottom) of *qSL-1f*, indicating the candidate region between 39.49 and 39.82 Mb

The LD decay analysis in the QTL region indicated that an approximately 328.8 kb region at the associated locus was the putative region for *qSL-1f* (Fig. 4). Based on release 7 of the MSU Rice Genome Annotation Project on rice IRGSP-1.0 genome (http://rice.plantbiology.msu.edu/) (Kawahara et al. 2013), there are 54 annotated genes within the putative region.

Using the genome re-sequencing information (50×) of 343 rice accessions used in this study (Wang et al. 2023), the haplotypes of all annotated genes within the putative region were analyzed according to the CDS region variation of each gene, and the significance of differences in SL among the major haplotypes (containing more than 10 lines) were tested.

**Fig. 5:**
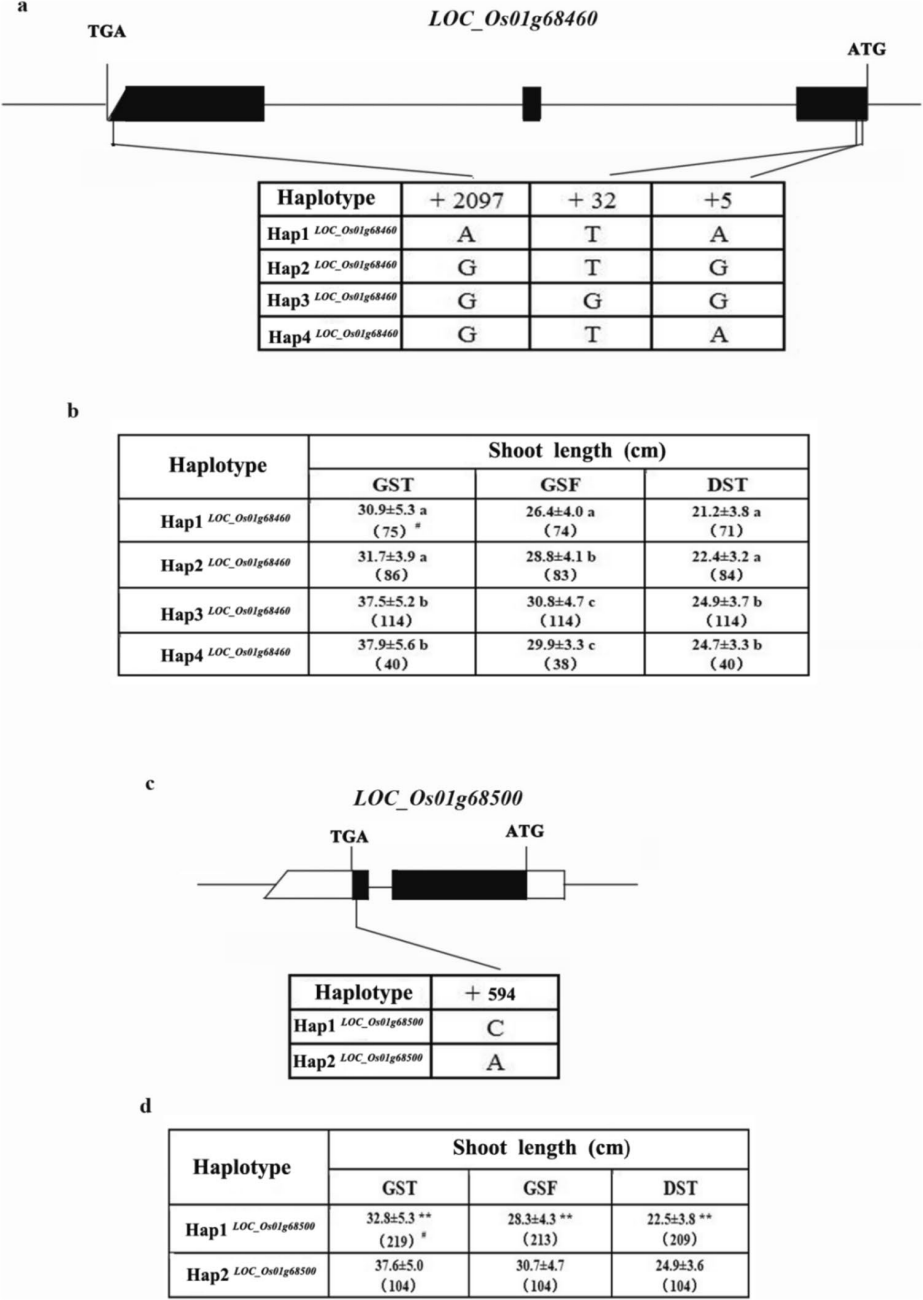
Gene structure and haplotype analysis of the candidate genes underlying *qSL-1f*. **a** and **c** The sequence variations in the CDS region and the resulting haplotypes of *LOC_Os01g68460* (**a**) and *LOC_Os01g68500* (**c**). **b** and **d**, The shoot length of various haplotypes for *LOC_Os01g68460* (**b**) and *LOC_Os01g68500* (**d**) under GST, GSF and DST. Shoot length is presented in mean ± SD. The values with the same lower letter indicate no significant difference in shoot length at *P* = 0.05 based on Duncan’s multiple range test; *double asterisk* indicates the significant difference in shoot length at *P* = 0.01 based on *t*-test; ^#^Numbers in parenthesis indicate the number of rice accessions

Only two genes, *LOC_Os01g68460* (*Os01g0912800*) and *LOC_Os01g68500* (*Os01g0913100*), showed significant differences in SL among their haplotypes, respectively (Fig. 5, Table S3). For *LOC_Os01g68460*, there were three major single-base variations in the CDS region that cause amino acid substitution. Four haplotypes were identified based on the variations, in which Hap2^*LOC_Os01g68460*^ is corresponding to reference genome Nipponbare (Fig. 5a). The lines carrying Hap3^*LOC_Os01g68460*^ and Hap4^*LOC_Os01g68460*^ exhibited significantly longer SL than those carrying Hap1^*LOC_Os01g68460*^ and Hap2^*LOC_Os01g68460*^ under the three cultivation methods (*P* < 0.05) (Fig. 5b). For *LOC_Os01g68500*, there was mainly a single-base variation in its CDS region that caused amino acid substitution (Ala-Ser), and SL exhibited significantly different between the two haplotypes identified based on this variation. Hap1^*LOC_Os01g68500*^ is consistent with the allele of Nipponbare (Fig. 5c). Under the three cultivation methods, the lines carrying Hap2^*LOC_Os01g68500*^ exhibited significantly longer SL than those carrying Hap1^*LOC_Os01g68500*^ (*P* < 0.01) (Fig. 5d). However, we could not figure out the most possible candidate gene for *qSL-1f* based on these results.

**Fig. 6:**
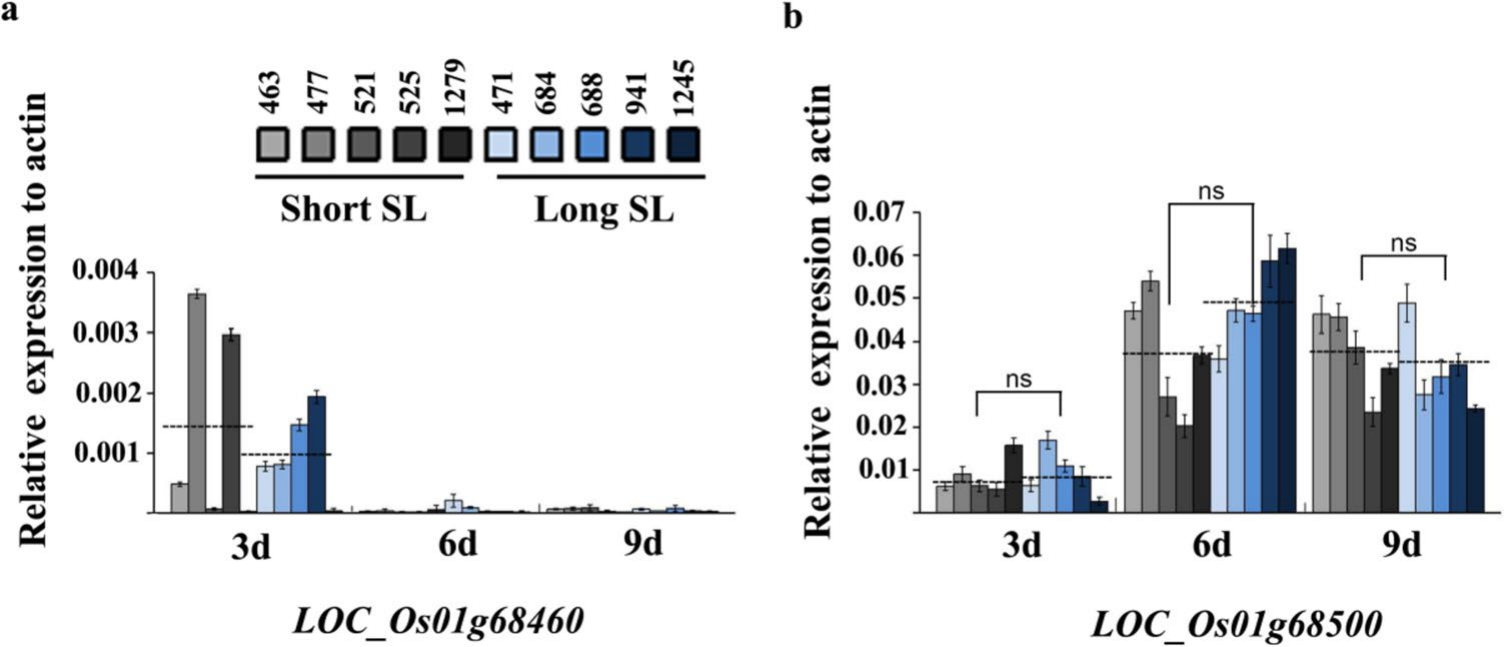
Temporal expression patterns of the two candidate genes in short SL lines (accession 463, 477, 521, 525 and 1279) and long SL lines (accession 471, 684, 688, 941 and 1245) measured by qRT-PCR. ns means no significant difference at *P* = 0.05, based on *t-*test

To further confirm the candidates, gene differential expression analysis was conducted using two sets of contrasting lines. According to the haplotype analysis of *qSL-1f* (Fig. 3), five lines (accession 463, 477, 521, 525 and 1279) with short SL and five lines (accession 471, 684, 688, 941 and 1245) with long SL were selected from the short and long SL haplotype panel, respectively. The qRT-PCR assays exhibited that the expression of *LOC_Os01g68460* was hard to be detected at all three time points, suggesting that this gene could be unlikely to be involved in regulating seedling growth, while there was no significant difference in the expression of *LOC_Os01g68500* between the short and long SL lines (*P* > 0.05, Fig. 6).

### Functional confirmation of the candidates

**Fig. 7:**
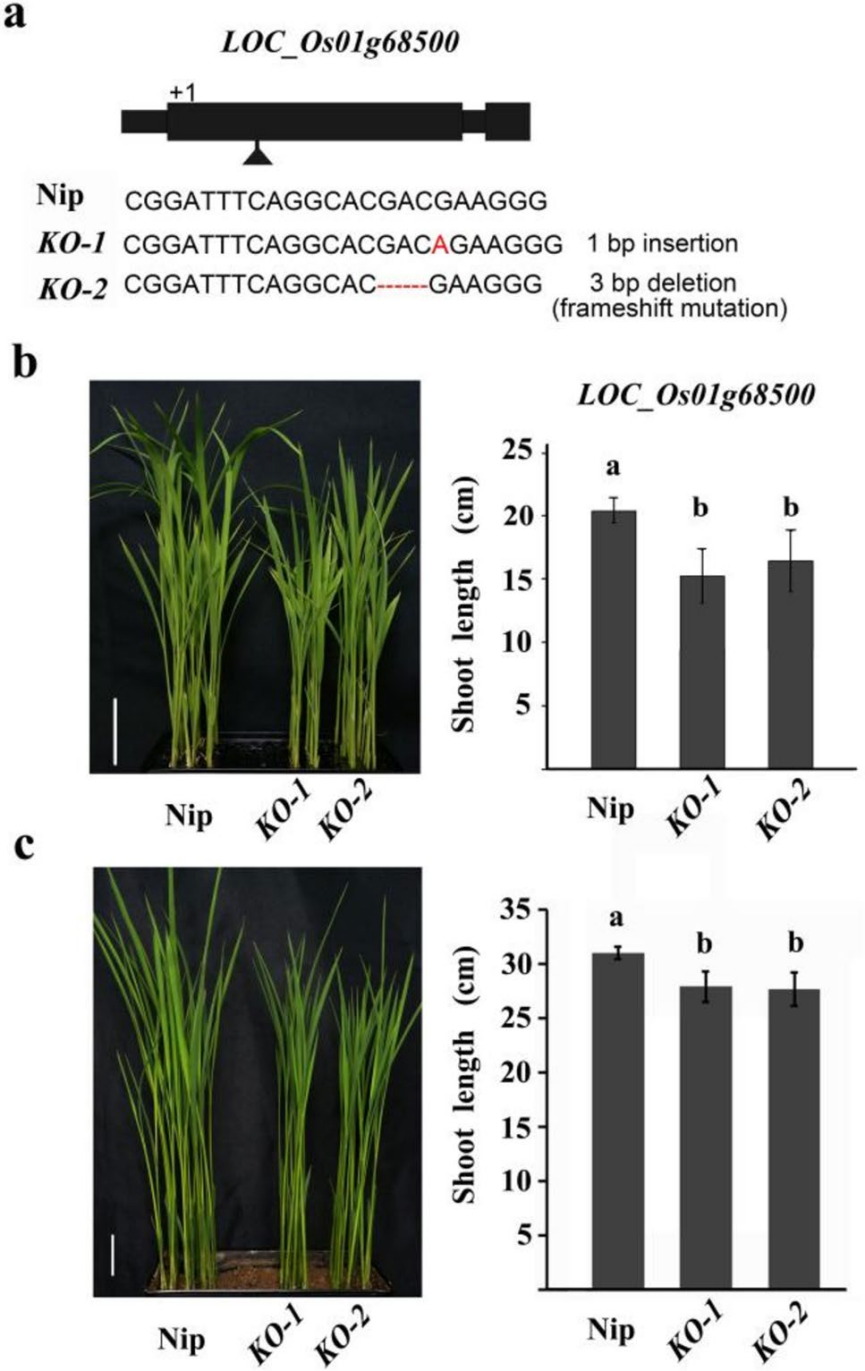
Mutation types and shoot lengths of the knockout transgenic lines of *LOC_Os01g68500*. **a** The mutation types of *LOC_Os01g68500*. **b** and **c** The shoot length of the knockout transgenic (*KO*) lines of *LOC_Os01g68500* were significantly shorter than that of their wild-type Nipponbare (Nip) in nutrition solution (**b**) and soil culture (**c**). The different letter upon the histogram indicates the significant difference at *P* = 0.05 based on Duncan’s multiple range test. Scale bar, 3 cm

To validate the effect of the candidate genes on SL, CRISPR/Cas9 was applied to knock out *LOC_Os01g68460* and *LOC_Os01g68500* in Nipponbare, respectively. In *T*_2_ generation, two homozygous lines of each transgenic plant were selected for SL measurement using two cultivation methods (Fig. S1a, Fig. 7a). The results showed that there was significant difference in SL between the *LOC_Os01g68500* knockout transgenic (*KO*) lines and Nipponbare (the wild type) (Fig. 7b, c), but none between the *LOC_Os01g68460 KO* lines and Nipponbare (Fig. S1b, c). In nutrient solution, the SL of Nipponbare was 20.5 cm, while that of the *LOC_Os01g68500 KO* lines were 16.4 cm and 15.2 cm (Fig. 7b); in soil culture, the SL of Nipponbare was 30.9 cm, while that of the *LOC_Os01g68500 KO* lines were 27.9 cm and 27.7 cm (Fig. 7c). Compared with their wild type, the SL of the *KO* lines were significantly shortened (*P* < 0.05), with an average reduction of 22.9% and 10.0% in nutrient solution and soil culture, respectively, indicating that *LOC_Os01g68500* is the causal gene for SL in rice.

### The pyramiding effects of* qSL-1d *and *LOC_Os01g68500* on shoot length

Pyramiding favorable haplotypes of different QTLs/genes for SL is an efficient way to develop rice varieties with stronger seedling vigor. In the present study, two stably expressed QTLs, *qSL-1d* and *qSL-1f* would be beneficial to improve seedling vigor. For *qSL-1f*, there were two main haplotypes underlying its causal gene *LOC_Os01g68500*, of which Hap2^*LOC_Os01g68500*^ exhibited longer SL (Fig. 5d); For *qSL-1d*, two main haplotypes were identified based on three significant SNPs within its interval, of which Hap2^*qSL−1d*^ exhibited longer SL (Fig. S2). To analyze the pyramiding effects of the two stable QTL, the rice accessions in the population were grouped according to their haplotypes of the QTL/gene and three haplotype combinations were found (Table 2). The SLs among different combinations showed significant differences, with identical trends in the three cultivation environments. Particularly, the combination 3, which pyramiding the favorable haplotypes of two QTLs, exhibited the longest SL (Table 2).

**Table 2:** The pyramiding effect of *qSL-1d* and *LOC_Os01g68500* on shoot length

Combinations	QTL/gene		Shoot length (cm)		
	*qSL-1d*	*LOC_Os01g68500*	GST	GSF	DST
1	Hap1^*qSL-1d*^	Hap1^*LOC_Os01g68500*^	28.8 ± 4.2a (36)	24.3 ± 3.2a (36)	18.9 ± 2.5a (34)
2	Hap2^*qSL-1d*^	Hap1^*LOC_Os01g68500*^	33.6 ± 5.1b (154)	28.9 ± 3.6b (154)	23.4 ± 3.4b (153)
3	Hap2^*qSL-1d*^	Hap2^*LOC_Os01g68500*^	37.7 ± 4.8c (97)	30.6 ± 4.6c (97)	24.8 ± 3.3c (97)

Shoot length is presented in mean ± SD. The values with the different letters indicate the significant difference at *P* = 0.05 based on Duncan’s multiple range test. Numbers in parenthesis indicate the number of rice accessions

## Discussion

### The *qSL-1f* has great potential value in rice breeding for direct seeding

Seedling vigor is important for rice to compete with weeds at the early stage of growth in direct seeding rice systems (Mahajan and Chauhan 2013; Kumar et al. 2016), in which SL is one of the important traits to enhance seedling emergence (Abe et al. 2012; Lu et al. 2016). Numerous studies have shown that SL in rice is a complex trait controlled by multiple genes/QTLs, and the expression of most of these QTLs is affected by environments (Zhou et al. 2007; Cairns et al. 2009; Cordero-Lara et al. 2016; Lu et al. 2016; Zhang et al. 2017; Zeng et al. 2021). Therefore, when the QTL is used in breeding, the feasibility of marker-assisted selection is dependent on the reproducibility of marker-QTL associations across populations and environments. Due to the diversity of environment and methods in rice direct seeding system, identifying the stably expressed QTLs is pivotal for successful molecular breeding.

In the present study, a total of twenty-four QTLs for SL were identified through GWAS. Among these QTLs, the eighteen are firstly reported in the present study, and the other six co-localized with the previously identified QTLs for SL (Table 1), suggesting the diversity of the rice germplasm used in the present study and the reliability of the results. In addition, among the 24 QTLs identified in the present study, *qSL-1d/qSL_IND-1c* and *qSL-1f/qSL_IND-1e* could be identified using all three cultivation methods, *qSL-1c/qSL_IND-1a* and *qSL-4e* could be identified using two cultivation methods, while the other 20 QTLs could only be identified under one cultivation method, indicating that QTLs for SL were significantly affected by environments. Therefore, being a newly identified QTL with large effect on SL, and stably detected under various cultivation regimes based on the production practice (Table 1), *qSL-1f* has great potential value in rice breeding for direct seeding.

### *LOC_ Os01g68500* is a novel gene for shoot length in rice

Although great progress has been made in the analysis of the genetic basis of SL in rice, few of their functional genes have been identified except for *qPHS3-2* (Abe et al. 2012)/*qEPD2* (Yano et al. 2012). In this study, we found that *LOC_Os01g68500* underlying *qSL-1f* was the causal gene for SL through gene-based haplotype analysis, gene expression and knockout transgenic verification.

Two major haplotypes in *LOC_Os01g68500* were identified in the present study. Sequence comparison of CDS region revealed a single-base transversion (C-to-A) at the position of + 594, causing an amino acid substitution from alanine to serine, and resulting in a significant difference in SL between Hap1^*LOC_Os01g68500*^ and Hap2^*LOC_Os01g68500*^ (Fig. 5c, d). Further expression analysis exhibited no significant difference in expression level between the short and long SL lines (Fig. 6b). These results indicate that the CDS variation in *LOC_Os01g68500* is the cause for the difference in SL.

*LOC_Os01g68500* (*Os01g0913100*) encodes a DUF containing protein which belongs to DUF538 family (http://rapdb.dna.affrc.go.jp). DUF538 proteins were widely distributed in plants (Takahashi et al. 2013; Yu et al. 2021), and their involvement in various biological processes, including plant growth and development, defense and adaptation to environmental stresses, has been reported. Structural and functional study suggested that *Celosia* DUF538 protein, was the potential homologue of mammalian BPI (bactericidal/permeability increasing) proteins and affected the bacterial growth by binding to the bacterial membranes (Gholizadeh and Kohnehrouz 2013). Subsequently, the DUF538 proteins were predicted to possess an esterase-type hydrolytic activity to chlorophylls (Gholizadeh 2016) and a methylesterase activity to pectins (Gholizadeh 2020), as well as structural and functional homologues of lipocalins (Gholizadeh 2022), suggesting their potential roles in the process of chlorophyll degradation in photosynthetic apparatus, cell wall-associated defense responses and trafficking or metabolizing mechanism in plants. It has been reported that *gl6*, the gene encoding a DUF538 protein in maize was involved in the intracellular trafficking of cuticular waxes, thereby influencing cuticular wax accumulation and cuticle permeability, and regulating drought tolerance (Li et al. 2019). Another DUF538 protein gene, *Ptom.006G00815*, was identified as one of the hub genes in the genetic network underlying stomatal morphology, which regulated leaf physiology and drought tolerance in *Populus* via modulation of stomatal shape and density (Li et al. 2023). By transcriptome analysis, a gene with DUF538, *SVB* (SMALLER WITH VARIABLE BRANCHES) was identified to alter trichome branch formation in *Arabidopsis* (Marks et al. 2009). Recently, a study reported *SVB* and *SVBL* (*SVB-Like*) synergistically modulated plant growth and trichome development in *Arabidopsis* through the transcriptional regulation of *GLABRA1*, one of the central hub genes for trichome development (Yu et al. 2021). Furthermore, *CU406238*, a DUF538 protein gene was found to be highly expressed in 3-days germinating seeds of rice (Lu et al. 2008). In the present study, the effect *LOC_Os01g68500* on SL in rice was validated by knockout transgenic experiment. Further, the sequence analysis was performed based on multiple sequence alignments of LOC_Os01g68500 protein and the above four reported DUF538 proteins. The results showed that the DUF538 domains were conserved among the five plant species (Fig. S3), indicating that this domain is important for the gene functions and these conserved amino acids in the domain may play important roles for this domain, although they performed their specific functions in different plant species. To our knowledge, it is the first time to discover the function of DUF538 protein gene on rice seedling growth. Further study is needed to understand the regulation mechanism of *LOC_Os01g68500* on SL.

### The suitable haplotypes of *LOC_Os01g68500* are beneficial to achieve the desired shoot length in rice breeding

The utilization of natural variation in genes is important for rice breeding. Map-based cloning is the main approach to explore the functional genes with natural variation, but usually uses populations developed by two parents, in which contains only two kinds of alleles and the obtained one may not be the optimal. In order to make better use of cloned genes for molecular breeding, it is necessary to understand their haplotypes, so that the favorable haplotype can be obtained. A gene-based haplotype analysis can use to discover variations in specific genes, which helps to determine the types of mutation in a gene, and explore how the gene affects the trait.

For *LOC_Os01g68500*, two main haplotypes were identified in 343 diverse rice accessions used in this study, in which Hap1^*LOC_Os01g68500*^ exhibited short SL, while Hap2^*LOC_Os01g68500*^ exhibited long SL (Fig. 5d). Sequence analysis reveals that one-base transversion in its CDS region is the cause for the significant difference in SL between the two main haplotypes (Fig. 5c, d). Therefore, this single base variation could be considered as a functional variation and used in molecular breeding. Molecular markers could be developed based on this functional variation, and the suitable alleles can be used to breed varieties with the desired SL for different breeding objectives. In addition, pyramiding the favorable haplotypes of *LOC_Os01g68500* and *qSL-1d* can significantly increase SL (Table 2), which provides evidence for improving SL through pyramiding breeding.

### GWAS combined with gene-based haplotype analysis is an effective way to identified candidate genes

GWAS can make full use of the recombination information accumulated in populations during long-term natural evolution and artificial selection, which can significantly improve the accuracy of QTL mapping. After identifying the interested QTLs, further determination of their causal genes is important for understanding the genetic basis of phenotypic variation and carrying out molecular breeding in rice. Although the range of the candidate interval can be roughly delimited based on the LD decay, the interval is usually 200–300 kb, in which there are dozens of annotated genes. It is time-consuming and laborious to find candidate genes accurately. However, based on the genome re-sequencing information of each line, we can perform haplotype analysis on the putative genes, and then analyze the significance of differences in the target traits among various haplotypes, thus a large number of genes not associated with the target trait are excluded and the candidate gene can be accurately anchored. Numbers of novel genes for heading, flowering, plant height in rice have been successfully identified using this strategy (Yano et al. 2016).

In this study, there are 54 annotated genes within the region of *qSL-1f*. When testing the significance of differences in SL among the major haplotypes of these annotated genes, only two genes (*LOC_Os01g68460* and *LOC_Os01g68500*) showed significant differences in SL. Combined with gene expression analysis, it was easy to obtain the candidate gene underlying *qSL-1f*. Further knockout transgenic experiments confirmed that *LOC_Os01g68500* is causal gene for SL. These results suggest that GWAS combined with gene-based haplotype analysis is an effective way to identify the candidate gene, as well as its favorable haplotype/allele.

### Supplementary Information

Below is the link to the electronic supplementary material.

Supplementary file 1 (PDF 125 KB)

Supplementary file 2 (PDF 71 KB)

Supplementary file 3 (PDF 768 KB)

Fig. S1 Mutation types and shoot lengths of the knockout transgenic lines of *LOC_Os01g68460* a, The mutation types of LOC_Os01g68460. **b** and **c** The shoot length of the knockout transgenic (KO) lines of *LOC_Os01g68460* were not significantly different from that of their wild-type Nipponbare (Nip) in nutrition solution (**b**) and soil culture (**c**). The same letter upon the histogram indicates no significant difference at *P* = 0.05 based on Duncan’s multiple range test. Scale bar, 3 cm (PDF 105 KB)

Fig. S2 Differences in shoot length between the major haplotypes of *qSL-1d*. **a** The main haplotypes of *qSL-1d. * #The significant SNP position (bp); **b**–**d** Boxplots for shoot length based on the haplotypes of qSL-1d under GST (**b**), GSF (**c**) and DST (**d**). Numbers in parenthesis indicate the number of rice accessions with the haplotype. The black horizontal lines represent the median value; the upper side and lower side of the box represent the upper quartile and lower quartile, respectively; the whiskers represent the range of data, and small circle represents outlier. Double asterisk indicates the significant difference in shoot length at *P* = 0.01 based on t-test. (PDF 95 KB)

Fig. S3 The multiple sequence alignments between LOC_Os01g68500 and the four reported DUF538 proteins from other plants. The conserved amino acids are highlighted in dark color, and gaps are marked with dashes. The red line indicates the DUF538 domain (33-142 amino acids) of LOC_Os01g68500 protein. The amino acid sequence of LOC_Os01g68500 protein was collected from the Rice Genome Annotation Project website (http://rice.Plantbiology.msu.edu/). The full-length protein sequences of SVB in *Arabidopsis*, Ptom.006G00815 in *Populus*, GL6 in Maize and Celosia DUF538 were downloaded from the corresponding references, respectively (Marks et al. 2009; Li et al. 2019; Li et al. 2023; Gholizadeh and Kohnehrouz 2013). The multiple sequence alignments were performed using MEGA6 and GeneDoc (PDF 233 KB)

## Data Availability

The datasets supporting the conclusions of this article are provided within the article and its additional files.

## Abbreviations

DST: Direct seeding into the trays
GST: Pre-germinated seeds were sown in trays
GSF: Pre-germinated seeds were sown in field
GWAS: Genome-wide association study
LD: Linkage disequilibrium
MAF: Minor allele frequency
PC: Principal component
QTLs: Quantitative trait loci
RDP2: Rice diversity panel 2
SNP: Single-nucleotide polymorphism
SL: Shoot length
