# Supplementary material for: Genome-wide association mapping combined with gene-based haplotype analysis identify a novel gene for shoot length in rice (Oryza sativa L.)
Source: Theor Appl Genet. 2023 Nov 20;136(12):251. doi: 10.1007/s00122-023-04497-6 (PMC10661777; doi:10.1007/s00122-023-04497-6)
Supplement: Supplementary file 1 — (PDF 125 KB) [file 122_2023_4497_MOESM1_ESM.pdf]

Table S1 The shoot length and information of 391 rice accessions

Accession number in this study	Subpopulation	Shoot length (cm)			Accession name	Country of origin
		Pre-germinated seeds were sown in plastic trays (GST)	Pre-germinated seeds were sown in the paddy field (GSF)	Seeds were directly sown in plastic trays without pre-germination (DST)		
**6**	Aus	**39.1**	**24.9**	**20.2**	AUS JOTA::IRGC 66767-1	Bangladesh
**7**	Aus	**47.1**	**39.6**	**27.8**	BARAN BORO::IRGC 27509-1	Bangladesh
**14**	Indica	**36.3**	**27.4**	**24.7**	CHITRAJ (DA 23)::IRGC 6208-1	Bangladesh
**19**	Indica	**28.1**	**26.4**	**20.6**	CO 39::IRGC 51231-1	India
**20**	Indica	**36.1**	**31.4**	**30.3**	DA 1::IRGC 45624-1	India
**24**	Aus	**39.6**	**32.5**	**24.4**	DA 8::IRGC 6422-1	Bangladesh
**25**	Indica	**29.3**	**23.3**	**20.9**	DA 9::IRGC 5854-1	Bangladesh
**30**	Indica	**33.6**	**28.0**	**24.7**	FANDRAPOTSY::IRGC 10984-1	Madagascar
**36**	Japonica	**36.4**	**25.6**	**24.0**	GOMPA 2::IRGC 12894-1	India
**37**	Japonica	**28.6**	**27.0**	**20.1**	HAIFUGOYA::IRGC 17054-1	Taiwan,China
**39**	Japonica	**29.8**	**25.2**	**20.8**	INDANE::IRGC 33130-1	Myanmar
**46**	Aus	**38.1**	**29.7**	**22.8**	JHONA 26::IRGC 27967-1	Pakistan
**50**	Japonica	**29.9**	**29.0**	**25.7**	KHAO DAM::IRGC 23385-1	Lao People's Democratic Republic
**51**	Japonica	**30.1**	**27.3**	**22.4**	KINANDANG PATONG::IRGC 23364-1	Philippines
**56**	Indica	**50.2**	**37.6**	**30.5**	MADAEL::IRGC 7722-1	Sri Lanka
**58**	Japonica	**30.1**	**29.2**	**21.1**	MALAGKIT PIRURUTONG::IRGC 8182-1	Philippines
**62**	Indica	**39.4**	**31.0**	**25.0**	MTU 9::IRGC 7919-1	India
**64**	Japonica	**35.1**	**30.1**	**-**	NHTA 5::IRGC 186-1	India
**66**	Indica	**44.0**	**36.2**	**31.1**	PACHEHAI PERUMAL::IRGC 8188-1	India
**77**	Indica	**38.6**	**30.4**	**25.6**	PTB 9::IRGC 6274-1	India
**78**	Japonica	**23.1**	**21.3**	**17.2**	RATHAL::IRGC 31524-1	Sri Lanka

**79**	Japonica	**24.5**	**22.3**	**17.0**	RATHAL::IRGC 31525-1	Sri Lanka
**82**	Japonica	**29.3**	**27.3**	**22.9**	RT 1031-69::IRGC 15092-1	The Democratic Republic of the Congo
**84**	Aus	**37.8**	**31.3**	**25.2**	TCHAMPA::IRGC 32362-1	Iran
**86**	Indica	**46.3**	**31.5**	**27.4**	TD 25::IRGC 9146-1	Thailand
**87**	Aus	**42.8**	**35.2**	**28.7**	TEPI BORO::IRGC 27519-1	Bangladesh
**414**	Aus	**34.1**	**25.1**	**19.1**	AUS 196::IRGC 29016-1	Bangladesh
**416**	Aus	**33.5**	**25.4**	**20.7**	DHALA BHADOI::IRGC 66779-1	Bangladesh
**423**	Aus	**32.3**	**26.6**	**21.3**	29 A 2::IRGC 28364-1	Pakistan
**427**	Indica	**35.4**	**31.9**	**24.8**	AN QING ZAO::IRGC 72577-1	China
**433**	Aus	**33.0**	**27.4**	**24.2**	ARC 11755::IRGC 21618-1	India
**436**	Aus	**40.6**	**31.9**	**26.1**	ARC 15340::IRGC 42019-1	India
**440**	Aus	**34.3**	**22.7**	**21.1**	AUS 55::IRGC 28918-1	Bangladesh
**442**	Indica	**38.5**	**30.5**	**25.2**	BADA DHAN::IRGC 26540-1	Bangladesh
**443**	Aus	**38.0**	**36.6**	**25.9**	BAILAM::IRGC 25835-1	Bangladesh
**449**	Aus	**43.1**	**29.9**	**24.1**	BAULAN::IRGC 64766-1	Bangladesh
**450**	Aus	**47.9**	**39.6**	**33.9**	BAWOI::IRGC 34737-1	Bangladesh
**452**	Aus	**37.1**	**26.2**	**22.6**	BENAMURI::IRGC 25840-1	Bangladesh
**453**	Indica	**38.5**	**31.9**	**25.7**	BG 301::IRGC 117315-1	Sri Lanka
**454**	Indica	**24.8**	**21.2**	**18.4**	BG 34-11::IRGC 15782-1	Sri Lanka
**455**	Aus	**39.4**	**31.2**	**25.0**	BHADOIA 303::IRGC 6588-1	Bangladesh
**456**	Indica	**35.7**	**32.1**	**21.9**	BIRAIN 360::IRGC 6550-1	Bangladesh
**457**	Aus	**-**	**28.7**	**23.3**	BIRANJ::IRGC 74733-1	India
**458**	Aus	**35.0**	**32.1**	**26.9**	BOILAN::IRGC 49022-1	Bangladesh
**461**	Indica	**30.1**	**29.5**	**-**	BR IRGA 409::IRGC 116960-1	Brazil
**462**	Indica	**45.4**	**32.3**	**25.8**	BYAT KYAR::IRGC 33004-1	Myanmar
**463**	Indica	**27.7**	**20.7**	**16.3**	CAMPONI SML::IRGC 50640-1	Surinam
**468**	Aus	**33.2**	**22.6**	**19.0**	CHIADI NAKI::IRGC 52101-1	India
**469**	Aus	**41.8**	**31.3**	**25.3**	CHIKON SHONI::IRGC 64771-1	Bangladesh
**471**	Indica	**44.2**	**38.0**	**25.1**	CHORUA KARTIKSAIL::IRGC 77230-1	Bangladesh
**472**	Indica	**36.6**	**34.0**	**25.5**	CHUA DAU::IRGC 4785-1	China
**473**	Aus	**39.7**	**32.5**	**26.2**	CHUNGUR BALI::IRGC 25855-1	Bangladesh

**476**	Indica	**27.5**	**22.0**	**19.5**	CR 5272::IRGC 116971-1	Costa Rica
**477**	Indica	**27.0**	**23.2**	**-**	CR 8334::IRGC 116972-1	Costa Rica
**484**	Indica	**44.8**	**34.6**	**26.1**	DEVAREDDIRI::IRGC 40788-1	Sri Lanka
**488**	Aus	**34.9**	**28.6**	**22.7**	DHARIA BOALIA::IRGC 64774-1	Bangladesh
**489**	Aus	**40.8**	**33.0**	**26.5**	DHARIA::IRGC 64773-1	Bangladesh
**491**	Indica	**29.5**	**27.7**	**20.6**	DJOGOLON DJOGOLON::IRGC 75577-1	Burkina Faso
**494**	Indica	**30.5**	**26.3**	**19.8**	E ZI 124::IRGC 70215-1	China
**500**	Aus	**30.0**	**22.3**	**19.3**	FULKATI::IRGC 66783-1	Bangladesh
**501**	Aus	**43.1**	**34.6**	**26.8**	GADRA::IRGC 73098-1	Pakistan
**502**	Aus	**37.9**	**28.4**	**25.2**	GARIA::IRGC 25854-1	Bangladesh
**505**	Indica	**41.2**	**35.2**	**27.6**	GOPAL::IRGC 61953-1	Nepal
**507**	Aus	**43.0**	**33.0**	**26.2**	GUL MURALI::IRGC 66792-1	Bangladesh
**511**	Aus	**31.7**	**23.8**	**21.1**	HARBHOONDI::IRGC 74744-1	India
**515**	Aus	**42.8**	**38.9**	**30.7**	HOLOI BASH (SOLOI BASH)::IRGC 64778-1	Bangladesh
**516**	Indica	**37.0**	**32.3**	**25.6**	HONG MI DONG MAO ZHAN::IRGC 68078-1	China
**517**	Indica	**47.7**	**33.0**	**31.1**	HONG ZUI ER::IRGC 72706-1	China
**518**	Indica	**42.3**	**34.9**	**25.8**	HONG ZUI ZAO::IRGC 68090-1	China
**520**	Indica	**37.8**	**29.8**	**24.5**	HUO SHAO TZU WU CHAN::IRGC 65097-1	Taiwan,China
**521**	Indica	**30.5**	**22.6**	**17.9**	ICTA PAZOS::IRGC 116996-1	Guatemala
**522**	Indica	**28.7**	**19.3**	**16.4**	ICTA POLOCHIC::IRGC 116997-1	Guatemala
**523**	Aus	**45.9**	**32.4**	**26.0**	IKRA::IRGC 77250-1	Bangladesh
**525**	Indica	**26.3**	**24.2**	**18.2**	IR 31917-45-3-2::IRGC 78132-1	Philippines
**527**	Indica	**26.4**	**24.3**	**19.3**	IR 43::IRGC 117005-1	Philippines
**528**	Indica	**33.6**	**27.0**	**20.6**	IR 59469-2B-3-2::IRGC 99703-1	Philippines
**532**	Indica	**29.1**	**23.1**	**18.3**	IR 77384-12-35-3-12-1-B::IRGC 117299-1	Philippines
**536**	Aus	**39.0**	**28.0**	**25.9**	JAMBALI BUSSA::IRGC 73102-1	Pakistan
**539**	Aus	**33.0**	**24.6**	**19.0**	JHUM BEGUNBICHI::IRGC 25867-1	Bangladesh
**542**	Indica	**25.3**	**24.2**	**17.8**	JUMA 51::IRGC 117009-1	Dominican Republic
**543**	Aus	**39.5**	**29.7**	**27.3**	KAL SHONI::IRGC 64780-1	Bangladesh
**546**	Aus	**40.3**	**29.6**	**25.8**	KALO KUCHI::IRGC 66806-1	Bangladesh
**547**	Aus	**38.3**	**32.9**	**28.5**	KALU A 30::IRGC 49746-1	Sri Lanka
**548**	Aus	**39.5**	**32.8**	**26.4**	KANGRO::IRGC 73105-1	Pakistan
**549**	Indica	**46.5**	**35.4**	**28.6**	KANNI MURUNGA::IRGC 15432-1	Sri Lanka

**550**	Indica	**43.6**	**37.9**	**29.5**	KARAYAL::IRGC 51001-1	Sri Lanka
**551**	Aus	**34.5**	**27.6**	**20.8**	KASAPUR::IRGC 74751-1	India
**555**	Aus	**35.4**	**26.9**	**22.4**	KELE BARDHAN::IRGC 34983-1	India
**556**	Aus	**33.3**	**28.1**	**23.7**	KELEE::IRGC 66807-1	Bangladesh
**557**	Indica	**40.7**	**28.9**	**25.5**	KHAO DAW TAI::IRGC 24108-1	Thailand
**558**	Indica	**40.4**	**31.7**	**27.5**	KHAO PON::IRGC 48114-1	Thailand
**561**	Indica	**39.6**	**28.6**	**25.0**	KINANDANG PUTI::IRGC 44513-1	Philippines
**562**	Indica	**38.0**	**33.9**	**26.6**	KIRIMURUNGA::IRGC 15585-1	Sri Lanka
**564**	Aus	**36.3**	**30.0**	**23.8**	KOYRA::IRGC 77267-1	Bangladesh
**565**	Indica	**37.3**	**31.6**	**24.6**	KUAN CHIN::IRGC 5589-1	Taiwan,China
**566**	Indica	**39.8**	**30.4**	**27.1**	KUMBI::IRGC 752-1	China
**571**	Indica	**36.0**	**32.2**	**22.8**	LAL BAGDAR::IRGC 77272-1	Bangladesh
**572**	Indica	**40.6**	**31.3**	**26.8**	LALBAJAM::IRGC 49227-1	Bangladesh
**576**	Indica	**41.6**	**33.7**	**29.9**	LIEN CHAN SHA PU TSAN::IRGC 4541-1	China
**578**	Indica	**35.7**	**28.9**	**21.3**	LOKU SAMBA::IRGC 31462-1	Sri Lanka
**581**	Indica	**39.6**	**32.9**	**27.0**	MA GU ZI HE::IRGC 68212-1	China
**583**	Aus	**41.3**	**35.0**	**26.5**	MADHABSAIL 741::IRGC 29374-1	Bangladesh
**585**	Indica	**36.7**	**28.3**	**25.5**	MAKALIOKA::IRGC 77864-1	Madagascar
**589**	Indica	**25.9**	**23.0**	**18.3**	MG 2::IRGC 79837-1	Brazil
**591**	Aus	**43.4**	**30.0**	**24.3**	MICOCHU::IRGC 25888-1	Bangladesh
**592**	Aus	**30.0**	**24.5**	**21.5**	MIKOTCHU::IRGC 25893-1	Bangladesh
**593**	Aus	**37.0**	**28.3**	**23.5**	MOSHIA BHADOI::IRGC 66817-1	Bangladesh
**594**	Aus	**40.5**	**28.9**	**23.4**	MOSHUR::IRGC 64789-1	Bangladesh
**595**	Indica	**35.5**	**28.3**	**24.8**	MOTTA SAMBA::IRGC 36489-1	Sri Lanka
**596**	Aus	**41.3**	**30.6**	**25.4**	MOYNA MOTI::IRGC 66818-1	Bangladesh
**598**	Indica	**39.6**	**30.1**	**25.9**	NAKABAWA::IRGC 70676-1	Kenya
**603**	Aus	**34.1**	**22.6**	**19.4**	NCS 160::IRGC 51903-1	India
**606**	Indica	**33.1**	**24.8**	**22.3**	NORUNKAN::IRGC 8934-1	Sri Lanka
**607**	Aus	**33.0**	**24.2**	**22.0**	NP 125::IRGC 32559-1	India
**608**	Indica	**42.4**	**31.3**	**28.2**	NS (VRAC) 130::IRGC 68972-1	Madagascar
**609**	Indica	**37.5**	**26.8**	**26.4**	NS 113::IRGC 68838-1	Madagascar
**611**	Indica	**24.0**	**20.4**	**17.0**	NSICRC 106::IRGC 117370-1	Philippines
**612**	Indica	**42.9**	**32.7**	**19.6**	PANAKALI::IRGC 47399-1	Sri Lanka

**613**	Aus	**37.1**	**27.4**	**22.6**	PANKHIRAJ::IRGC 25911-1	Bangladesh
**615**	Indica	**39.4**	**32.6**	**30.0**	PAWHTUN::IRGC 33562-1	Myanmar
**616**	Indica	**26.4**	**20.3**	**19.3**	PERLA::IRGC 117021-1	Cuba
**620**	Indica	**45.2**	**35.0**	**30.6**	PICONEGRO::IRGC 117022-1	Ecuador
**622**	Indica	**38.9**	**30.5**	**27.0**	PINURSIGI::IRGC 26889-1	Philippines
**625**	Indica	**40.0**	**29.1**	**24.8**	PURBIA (KALANSAR)::IRGC 59189-1	Nepal
**628**	Indica	**44.1**	**39.5**	**30.5**	RACE::IRGC 15706-1	Sri Lanka
**630**	Aus	**39.2**	**37.9**	**26.9**	RAKHOIL::IRGC 64793-1	Bangladesh
**632**	Indica	**38.6**	**33.7**	**24.4**	RATHKANDIRAM::IRGC 36507-1	Sri Lanka
**633**	Indica	**37.7**	**30.6**	**26.3**	RAY JAZAYKAYZ::IRGC 62181-1	Bhutan
**640**	Aus	**37.1**	**35.1**	**27.4**	SADA SOLAY::IRGC 73118-1	Pakistan
**643**	Indica	**40.4**	**33.3**	**27.7**	SAN DU BAI MI HONG GU::IRGC 59849-1	China
**645**	Aus	**36.7**	**30.6**	**26.3**	SANTHI SUFAID 207::IRGC 28212-1	Pakistan
**649**	Aus	**38.6**	**31.2**	**24.2**	SHIRKATI::IRGC 14528-1	Afghanistan
**650**	Indica	**29.8**	**25.4**	**22.1**	SHONTH::IRGC 74717-1	India
**651**	Aus	**35.7**	**31.2**	**23.7**	SIMUL KHURI::IRGC 35154-1	India
**653**	Indica	**44.0**	**31.4**	**29.1**	SOKOU MALSIRA::IRGC 77301-1	Bangladesh
**654**	Aus	**35.0**	**21.3**	**19.3**	SOLOI::IRGC 37598-1	Bangladesh
**655**	Indica	**34.3**	**26.6**	**21.5**	SOMIMADAMO::IRGC 69044-1	Madagascar
**659**	Aus	**29.6**	**21.2**	**18.1**	SUFAID 246::IRGC 28303-1	Pakistan
**664**	Aus	**36.2**	**33.1**	**26.5**	TAK SUFAID::IRGC 73127-1	Pakistan
**667**	Indica	**43.2**	**35.3**	**25.8**	TI KU::IRGC 1224-1	China
**671**	Indica	**35.6**	**31.1**	**28.2**	TSIPALA MENA::IRGC 69977-1	Madagascar
**674**	Indica	**36.2**	**28.3**	**23.3**	UBA MURALI::IRGC 25928-1	Bangladesh
**676**	Aus	**39.1**	**25.7**	**22.1**	UPRB 56::IRGC 44229-1	India
**678**	Aus	**36.3**	**34.1**	**29.2**	URYEE BOOTA::IRGC 74719-1	India
**682**	Aus	**33.8**	**28.1**	**20.6**	WEDA HEENATI::IRGC 67661-1	Sri Lanka
**683**	Indica	**35.5**	**32.4**	**25.6**	XI GU HONG::IRGC 74226-1	China
**684**	Indica	**40.0**	**34.2**	**28.1**	XI NUO ZAO::IRGC 68279-1	China
**685**	Indica	**38.4**	**35.0**	**31.2**	YAKADA::IRGC 51096-1	Sri Lanka
**686**	Indica	**-**	**36.3**	**26.9**	YE TI ZHAN::IRGC 68296-1	China
**687**	Indica	**36.2**	**30.2**	**26.4**	ZALCHA::IRGC 62190-1	Bhutan
**688**	Indica	**41.4**	**33.5**	**28.9**	ZAO SHAO ZHAN::IRGC 68318-1	China

**689**	Indica	**28.1**	**24.5**	**21.1**	ZAO SHOU 691-11::IRGC 70447-1	China
**690**	Indica	**39.4**	**36.4**	**27.5**	ZI GAN NAN GU::IRGC 70468-1	China
**692**	Indica	**45.8**	**33.7**	**28.5**	AI JIAO XIAN::IRGC 72573-1	China
**694**	Indica	**39.6**	**31.4**	**23.8**	KAUKYAGYI::IRGC 33208-1	Myanmar
**696**	Indica	**36.4**	**31.5**	**27.7**	MAHADETHE::IRGC 74762-1	India
**700**	Indica	**38.6**	**35.7**	**25.6**	XI GU ZAO::IRGC 72360-1	China
**701**	Aus	**32.8**	**21.1**	**19.9**	SURJA MUKHI::IRGC 35166-1	India
**702**	Japonica	**30.3**	**28.5**	**24.8**	1-52-6::IRGC 39111-1	Brazil
**704**	Indica	**25.9**	**26.7**	**19.2**	AI QING 99::IRGC 63544-1	China
**706**	Aus	**35.8**	**29.2**	**25.2**	ARC 11559::IRGC 21477-1	India
**711**	Japonica	**29.8**	**24.4**	**21.0**	AZMIL 85::IRGC 289-1	Philippines
**713**	Japonica	**29.8**	**31.7**	**22.2**	BAKUNG (H)::IRGC 60220-1	Malaysia
**716**	Japonica	**34.4**	**28.6**	**22.4**	DUANG OJOY::IRGC 3832-1	Philippines
**717**	Aus	**36.2**	**25.3**	**21.4**	DULAR::IRGC 32561-1	India
**721**	Indica	**31.4**	**27.2**	**23.5**	GEETA::IRGC 74742-1	India
**725**	Aus	**29.8**	**25.3**	**20.5**	IC 27525::IRGC 53989-1	India
**726**	Aus	**33.4**	**23.5**	**13.9**	JHUM SONALICHIKON::IRGC 25874-1	Bangladesh
**730**	Japonica	**31.4**	**23.2**	**20.0**	MERCURY::IRGC 77141-1	United States of America
**731**	Japonica	**33.4**	**25.7**	**24.1**	MIKHUDEB::IRGC 25892-1	Bangladesh
**732**	Japonica	**36.0**	**-**	**-**	MIMIDAM::IRGC 25897-1	Bangladesh
**733**	Japonica	**30.8**	**25.9**	**19.9**	MO WAE DO::IRGC 2356-1	Republic of Korea
**740**	Aus	**38.9**	**30.4**	**22.7**	SOKANA::IRGC 52763-1	India
**745**	Indica	**37.6**	**35.5**	**25.2**	THAPACHINIYA::IRGC 16234-1	Nepal
**752**	Aus	**45.5**	**36.5**	**28.5**	DHOLI BORO::IRGC 27513-2	Bangladesh
**757**	Indica	**42.0**	**36.1**	**27.5**	DA 29 (SR 26 B)::IRGC 25850-1	Bangladesh
**761**	Japonica	**31.5**	**27.7**	**24.4**	62-667::IRGC 15147-1	Ivory Coast
**762**	Japonica	**34.7**	**30.1**	**23.4**	7507-137::IRGC 40081-1	Japan
**764**	Indica	**25.4**	**24.4**	**17.6**	78-48::IRGC 51498-1	China
**767**	Indica	**39.9**	**32.7**	**28.2**	AI KHAO PLE::IRGC 71012-1	Thailand
**772**	Aus	**34.4**	**28.1**	**16.6**	ANDIKULAN::IRGC 7738-1	Sri Lanka
**773**	Indica	**38.0**	**31.2**	**24.8**	ARC 10411::IRGC 12454-1	India
**787**	Japonica	**31.0**	**33.1**	**-**	ARGO::IRGC 82418-1	Italy

**794**	Japonica	**33.5**	**30.9**	**27.9**	BAGANAN ADONGKO::IRGC 71501-1	Malaysia
**795**	Japonica	**-**	**30.9**	**22.5**	BAGANAN ASALAO::IRGC 71503-1	Malaysia
**798**	Japonica	**31.6**	**27.3**	**20.7**	BALAYAN::IRGC 71505-1	Malaysia
**801**	Japonica	**34.0**	**32.8**	**22.9**	BANAT 2270::IRGC 65648-1	Romania
**806**	Japonica	**34.5**	**33.7**	**22.1**	BEN DI HONG KE NUO::IRGC 70065-1	China
**807**	Japonica	**35.8**	**32.6**	**26.3**	BLUE ROSE::IRGC 151-1	United States of America
**810**	Japonica	**30.4**	**27.1**	**23.4**	BOSSA::IRGC 57781-1	Guinea
**811**	Indica	**40.6**	**36.9**	**34.2**	BOTOHAVANA::IRGC 79459-1	Madagascar
**812**	Japonica	**34.2**	**27.4**	**24.4**	BOTPA BARA::IRGC 62162-1	Bhutan
**813**	Japonica	**28.7**	**28.6**	**25.5**	BOTRA FOTSY::IRGC 77840-1	Madagascar
**820**	Japonica	**31.9**	**33.5**	**28.3**	C 57-5043::IRGC 4057-1	United States of America
**821**	Japonica	**32.0**	**28.0**	**22.6**	C 8434::IRGC 13496-1	Papua New Guinea
**822**	Japonica	**28.1**	**27.7**	**20.6**	CAIAPO::IRGC 116962-1	Brazil
**823**	Japonica	**33.3**	**30.7**	**18.1**	CALMOCHI 202::IRGC 57082-1	United States of America
**829**	Japonica	**36.8**	**33.9**	**23.0**	CHANGYOUNG 7::IRGC 82284-1	Republic of Korea
**831**	Indica	**35.1**	**25.2**	**25.2**	CHINAN 2::IRGC 54656-1	India
**837**	Japonica	**26.4**	**27.2**	**22.2**	CNAR 2888-B-47::IRGC 117325-1	Brazil
**844**	Aus	**37.4**	**29.1**	**25.2**	DEHULA::IRGC 74737-1	India
**846**	Japonica	**28.7**	**30.0**	**20.8**	DOBONGBYEO (SUWEON 223)::IRGC 58315-1	Republic of Korea
**847**	Japonica	**27.6**	**24.4**	**17.7**	DOONGARA::IRGC 78392-1	Australia
**851**	Aus	**36.4**	**26.9**	**21.4**	DULAR::IRGC 636-1	India
**852**	Indica	**37.5**	**30.3**	**27.8**	DUMSIKALAM::IRGC 58968-1	Nepal
**854**	Japonica	**23.8**	**23.7**	**18.1**	ELVO::IRGC 82422-1	Italy
**859**	Japonica	**30.5**	**28.4**	**25.9**	FAROX 299::IRGC 63102-1	Nigeria
**861**	Japonica	**27.5**	**28.5**	**23.1**	GBANTE::IRGC 16081-1	Cote d'Ivoire
**871**	Japonica	**32.6**	**32.8**	**23.0**	HE JIANG 15::IRGC 61801-1	China
**874**	Japonica	**32.9**	**35.2**	**21.9**	HOMURA 8::IRGC 5388-1	Japan
**876**	Japonica	**31.0**	**33.8**	**20.9**	HUANG JING NUO::IRGC 80953-1	China
**880**	Japonica	**29.4**	**28.0**	**22.3**	IAC 164::IRGC 55860-1	Brazil
**883**	Japonica	**31.6**	**26.4**	**21.2**	INUWAY::IRGC 67437-1	Philippines

**885**	Japonica	**29.2**	**23.9**	**20.6**	IRAT 170::IRGC 64850-1	Cote d'Ivoire
**888**	Japonica	**29.0**	**26.1**	**20.8**	ITA 235::IRGC 64854-1	Cote d'Ivoire
**893**	Japonica	**28.7**	**27.1**	**24.0**	JI BO YA::IRGC 77446-1	Congo
**896**	Aus	**36.2**	**26.9**	**21.0**	JUMA::IRGC 66800-1	Bangladesh
**897**	Japonica	**39.4**	**31.2**	**26.0**	KAM MRA::IRGC 62172-1	Bhutan
**900**	Aus	**42.4**	**36.0**	**25.5**	KARUTHA CHEENEDDI::IRGC 47381-1	Sri Lanka
**902**	Aus	**36.9**	**32.6**	**26.2**	KHADASIYA 3 (STRAW)::IRGC 54072-1	India
**905**	Japonica	**34.7**	**34.0**	**30.4**	KHAO KAP SANG::IRGC 23423-1	Lao People's Democratic Republic
**908**	Indica	**37.9**	**34.1**	**-**	Khie tom::IRGC 11887-1	Lao People's Democratic Republic
**910**	Indica	**29.4**	**25.5**	**16.9**	KI 68::IRGC 34768-1	India
**912**	Indica	**34.9**	**29.2**	**21.4**	KOMOL BHOG::IRGC 77266-1	Bangladesh
**916**	Japonica	**29.3**	**27.7**	**22.7**	KPOGON::IRGC 30467-1	Liberia
**918**	Japonica	**28.1**	**28.1**	**18.2**	KULOB::IRGC 71544-1	Malaysia
**919**	Japonica	**32.0**	**27.5**	**24.8**	KULU::IRGC 11337-1	Australia
**922**	Japonica	**42.0**	**33.9**	**25.3**	KYRMYZY::IRGC 36164-1	Uzbekistan
**923**	Japonica	**34.5**	**33.0**	**21.0**	L 4-32::IRGC 13402-1	Romania
**927**	Japonica	**41.4**	**33.9**	**20.7**	LAO HU DAO::IRGC 59736-1	China
**929**	Japonica	**31.1**	**29.0**	**21.1**	LEVANTE HOMEM::IRGC 50492-1	Brazil
**931**	Indica	**39.2**	**32.2**	**27.2**	LI CHUAN DA BAI GU::IRGC 70325-1	China
**932**	Japonica	**28.7**	**34.9**	**29.7**	LIGERITO (CORTO PUBESCENTE)::IRGC 19919-1	Colombia
**937**	Japonica	**38.2**	**28.3**	**22.7**	M 9::IRGC 34282-1	United States of America
**940**	Indica	**32.5**	**28.3**	**19.4**	MAHSURI::IRGC 10929-1	Malaysia
**941**	Indica	**43.3**	**38.4**	**28.7**	MAI HSI TSAN::IRGC 51366-1	China
**942**	Indica	**39.4**	**33.5**	**24.9**	MALACHAN::IRGC 54748-1	India
**953**	Japonica	**32.3**	**30.1**	**22.8**	MIMIDIM::IRGC 25898-1	Bangladesh
**954**	Indica	**35.2**	**35.4**	**26.1**	MIN CHE NIEN HSU THOU::IRGC 4471-1	China
**963**	Japonica	**33.1**	**35.2**	**22.7**	NABOHAI::IRGC 17062-1	Taiwan,China
**967**	Indica	**37.1**	**31.1**	**29.2**	NAYIMA::IRGC 9503-1	Iraq
**973**	Japonica	**33.9**	**29.7**	**22.8**	NOUAKPO::IRGC 56704-1	Cote d'Ivoire
**981**	Japonica	**29.1**	**26.4**	**22.6**	ORIENTE 10::IRGC 55808-1	Ecuador

**988**	Japonica	**29.9**	**24.7**	**17.9**	PECOS::IRGC 66758-1	United States of America
**989**	Japonica	**36.9**	**27.3**	**24.8**	Peek::IRGC 11821-1	Lao People's Democratic Republic
**993**	Aus	**35.1**	**25.0**	**20.0**	PODI HEENATI::IRGC 36345-1	Sri Lanka
**995**	Japonica	**30.2**	**29.9**	**-**	POPONG::IRGC 13297-1	Malaysia
**999**	Indica	**36.7**	**27.3**	**25.0**	RAMJAWAIN::IRGC 74773-1	India
**1003**	Japonica	**27.9**	**26.0**	**21.3**	RHS 107-2-1-2TB-1JM::IRGC 117025-1	Mexico
**1006**	Japonica	**38.7**	**39.0**	**27.9**	RIZZOTTO 264::IRGC 65727-1	Italy
**1008**	Japonica	**27.2**	**26.5**	**19.5**	RXAR RGUE::IRGC 1943-1	United States of America
**1009**	Japonica	**26.1**	**24.8**	**20.5**	SACIA 1 (TACU)::IRGC 117027-1	Bolivia
**1010**	Japonica	**27.5**	**27.3**	**22.9**	SACIA 4 (JISUNU)::IRGC 117028-1	Bolivia
**1015**	Aus	**39.3**	**32.4**	**24.0**	SAREINA::IRGC 67757-1	India
**1017**	Japonica	**27.2**	**28.6**	**19.4**	SELENIO::IRGC 82434-1	Italy
**1023**	Indica	**28.7**	**23.9**	**16.7**	SIJAMBI SAPUSI::IRGC 66650-1	Indonesia
**1025**	Japonica	**31.2**	**33.9**	**20.6**	SSAL BYEO 2::IRGC 19868-1	Korea Rep
**1035**	Indica	**32.7**	**29.7**	**24.1**	SZIMAI::IRGC 65743-1	China
**1037**	Japonica	**-**	**24.9**	**19.1**	TAINAN IKU 512::IRGC 2993-1	Taiwan,China
**1043**	Japonica	**28.8**	**27.9**	**19.2**	TOANG::IRGC 19144-1	Indonesia
**1044**	Japonica	**28.6**	**28.8**	**20.7**	TOS 10483::IRGC 56723-1	Guinea-Bissau
**1047**	Indica	**37.8**	**32.9**	**27.5**	TSON HSU DAU::IRGC 4711-1	China
**1048**	Indica	**39.7**	**32.5**	**26.8**	TUO TUO HUANG::IRGC 72851-1	China
**1052**	Indica	**26.7**	**22.1**	**17.3**	VARY LAVA::IRGC 11033-1	Madagascar
**1054**	Japonica	**26.3**	**27.4**	**19.7**	VELA::IRGC 82437-1	Italy
**1055**	Japonica	**33.6**	**28.9**	**23.3**	VICTORIA::IRGC 3266-1	Argentina
**1056**	Japonica	**24.0**	**23.0**	**17.9**	WAB 176-8-HB::IRGC 117358-1	Cote d'Ivoire
**1057**	Japonica	**27.8**	**24.5**	**20.6**	WAB 368-B-1-H1-HB::IRGC 117359-1	Cote d'Ivoire
**1058**	Japonica	**24.4**	**22.8**	**18.7**	WAB 99-16::IRGC 117360-1	Cote d'Ivoire
**1060**	Japonica	**29.5**	**22.9**	**23.2**	WC 2615::IRGC 74460-1	Turkey
**1061**	Japonica	**36.8**	**34.5**	**23.4**	WIR 1072::IRGC 57496-1	Belgium
**1064**	Japonica	**38.8**	**40.2**	**30.3**	WIR 2521::IRGC 51658-1	Ukraine
**1068**	Japonica	**28.8**	**32.7**	**21.8**	WU KE NUO::IRGC 59990-1	China

**1069**	Japonica	**33.8**	**34.9**	**22.2**	YAKUMO::IRGC 5320-1	Japan
**1070**	Japonica	**32.6**	**35.1**	**22.8**	YAMATO CHIKARA::IRGC 5294-1	Japan
**1074**	Japonica	**32.8**	**35.7**	**25.0**	YU WEOL ZO::IRGC 19894-1	Republic of Korea
**1075**	Indica	**26.2**	**26.8**	**19.6**	ZHEN JIANG 2::IRGC 53439-1	China
**1079**	Japonica	**34.5**	**28.8**	**20.3**	ARC 11708::IRGC 21575-1	India
**1087**	Japonica	**38.7**	**28.8**	**26.1**	KETAN MERAH::IRGC 24977-1	Indonesia
**1088**	Japonica	**38.9**	**-**	**-**	KIUKI 46::IRGC 2486-1	Japan
**1089**	Japonica	**29.8**	**24.5**	**22.6**	L 201::IRGC 51099-1	United States of America
**1091**	Japonica	**32.2**	**30.1**	**20.6**	M 301::IRGC 55229-1	United States of America
**1092**	Japonica	**-**	**24.5**	**19.6**	M 401::IRGC 57084-1	United States of America
**1098**	Japonica	**29.4**	**32.2**	**20.9**	OOURA::IRGC 501-1	Japan
**1100**	Japonica	**40.1**	**39.2**	**33.5**	RIENALDO BERZANO::IRGC 3230-1	Turkey
**1104**	Japonica	**45.0**	**32.8**	**26.5**	WC 296::IRGC 1220-1	China
**1108**	Aus	**35.7**	**26.3**	**21.3**	JABARSHAIL::IRGC 25865-2	Bangladesh
**1111**	Indica	**26.3**	**25.9**	**19.5**	IR 5::IRGC 10321-1	Philippines
**1114**	Japonica	**35.6**	**28.0**	**24.0**	ARC 10176::IRGC 20765-1	India
**1117**	Indica	**32.8**	**28.8**	**22.5**	ARC 14500::IRGC 42957-1	India
**1120**	Japonica	**35.4**	**30.2**	**26.8**	BENGIZA::IRGC 69845-1	Madagascar
**1125**	Aus	**26.8**	**19.7**	**17.9**	CHANDARHAT::IRGC 25845-1	Bangladesh
**1128**	Indica	**25.7**	**23.2**	**17.5**	CR 762022::IRGC 53378-1	United States of America
**1131**	Indica	**38.0**	**37.1**	**25.6**	E KHA KEHA::IRGC 2764-1	Taiwan,China
**1140**	Japonica	**27.0**	**30.4**	**18.5**	JING XI 17::IRGC 67676-1	China
**1141**	Indica	**34.6**	**28.7**	**23.0**	JOALBHANGA 499::IRGC 6560-1	Bangladesh
**1143**	Indica	**46.8**	**38.5**	**30.0**	KAPUTU HOTA::IRGC 66516-1	Sri Lanka
**1147**	Indica	**46.7**	**37.8**	**28.9**	LUMBINI::IRGC 11947-1	Sri Lanka
**1153**	Japonica	**34.0**	**29.3**	**23.1**	PARE TORO::IRGC 54333-1	Indonesia
**1154**	Indica	**40.1**	**36.0**	**30.7**	POONAGARI PERUMAL::IRGC 47408-1	Sri Lanka
**1166**	Japonica	**28.8**	**29.0**	**23.4**	TOS 5790::IRGC 117256-1	Nigeria
**1170**	Indica	**31.6**	**26.1**	**19.7**	WU BAI LI::IRGC 70404-1	China
**1190**	Japonica	**33.1**	**28.2**	**21.3**	KWADWO AMOA::IRGC 14738-1	Ghana

**1192**	Japonica	**27.3**	**27.9**	**18.5**	MUT IAC 25-44-807::IRGC 68799-1	Guyana
**1203**	Indica	**39.7**	**32.4**	**27.4**	SR 26 B::IRGC 10798-1	Japan
**1214**	Japonica	**28.0**	**29.0**	**17.4**	PACHOLINHA::IRGC 50531-1	Brazil
**1216**	Japonica	**29.8**	**21.7**	**19.5**	CURINCA::C1	Brazil
**1217**	Japonica	**33.6**	**30.0**	**28.3**	HONDURAS::GERVEX 529-C1	Spain
**1219**	Japonica	**36.5**	**30.1**	**28.3**	MANDRIRAVINA 3512::GERVEX 8319-C1	Madagascar
**1222**	Japonica	**28.5**	**21.6**	**18.4**	ADAIR::GERVEX 1640-C1	United States of America
**1225**	Indica	**28.3**	**25.1**	**21.4**	APO::C1	Philippines
**1226**	Japonica	**34.1**	**28.5**	**24.9**	ARAGUAIA::IRTP 17399-C1	Brazil
**1229**	Japonica	**34.7**	**26.6**	**23.9**	ARROZ CEBADA::IRGC 65646-C1	Venezuela
**1234**	Indica	**38.6**	**32.4**	**27.1**	BELOHAKILA 119::GERVEX 8342-C1	Madagascar
**1235**	Japonica	**27.4**	**21.5**	**17.1**	BENGALY VAKARINA::GERVEX 4750-C1	Madagascar
**1237**	Indica	**39.6**	**33.5**	**30.0**	BODOMANO::GERVEX 8343-C1	Madagascar
**1245**	Indica	**40.0**	**41.2**	**30.3**	CHERIVIRUPPU::IRGC 19928-C1	India
**1251**	Japonica	**26.3**	**25.6**	**17.4**	COLINA::GERVEX 524-C1	Spain
**1252**	Japonica	**22.6**	**19.3**	**14.0**	DELLROSE::GERVEX 1648-C1	United States of America
**1254**	Japonica	**22.0**	**17.0**	**12.5**	DIXIEBELLE::GERVEX 1649-C1	United States of America
**1255**	Japonica	**30.4**	**27.8**	**23.4**	EARLY MUTANT IAC 165::GERVEX 8508-C1	Brazil
**1257**	Japonica	**31.0**	**29.6**	**19.4**	FOHISOMOTRA::GERVEX 8329-C1	Madagascar
**1261**	Japonica	**36.5**	**27.2**	**25.5**	GOGO LEMPAK::IRGC 43392-C1	Indonesia
**1262**	Japonica	**25.8**	**28.1**	**19.7**	GUARANI::GERVEX 8506-C1	Brazil
**1264**	Japonica	**29.0**	**23.7**	**21.5**	HD 1-4::C1	France
**1266**	Japonica	**29.7**	**23.3**	**22.1**	IDSA 77::C1	Cote d'Ivoire
**1267**	Japonica	**30.6**	**25.9**	**18.6**	ILANG-ILANG::GERVEX 509-C1	Republic of Korea
**1268**	Indica	**27.4**	**24.1**	**17.8**	IR 2006-P12-12-2::IRGC 32675-C1	Philippines
**1272**	Indica	**29.6**	**28.8**	**19.6**	IR 55419-04::C1	Philippines
**1273**	Indica	**27.9**	**25.7**	**18.8**	IR 60::IRGC 63493-C1	Philippines
**1275**	Japonica	**27.9**	**26.9**	**21.6**	IR 63372-8::C1	Philippines
**1276**	Japonica	**26.0**	**28.9**	**27.2**	IR 65261-09-1-B::C1	Philippines
**1279**	Indica	**26.3**	**22.5**	**17.0**	IR 71676-90-2-2::C1	Philippines

**1280**	Indica	**29.3**	**24.2**	**19.1**	IR 72::C1	Philippines
**1281**	Japonica	**28.4**	**22.8**	**18.3**	IRAT 109::GERVEX 4988-C1	Cote d'Ivoire
**1283**	Japonica	**34.6**	**29.2**	**20.2**	IRAT 212::GERVEX 7698-C1	Cote d'Ivoire
**1286**	Japonica	**32.7**	**27.3**	**25.5**	IRAT 2::GERVEX 606-C1	Senegal
**1291**	Indica	**27.3**	**25.9**	**18.4**	JAMAJIGI::C1	Mali
**1292**	Japonica	**34.5**	**27.2**	**26.3**	JIMBRUK JOLOWORO::IRGC 43420-C1	Indonesia
**1294**	Indica	**36.9**	**30.4**	**26.8**	KALINGA III::C1	India
**1295**	Japonica	**35.1**	**33.4**	**24.7**	KETAN KONIR::IRGC 43444-C1	Indonesia
**1298**	Indica	**35.1**	**28.7**	**24.3**	KITRANA 1890::GERVEX 5115-C1	Madagascar
**1301**	Japonica	**31.5**	**-**	**-**	KUROKA::IRGC 74556-C1	Japan
**1302**	Japonica	**27.8**	**24.5**	**21.3**	KYEEMA::GERVEX 1656-C1	Australia
**1305**	Indica	**34.9**	**29.5**	**23.9**	LOHAMBITRO 224::GERVEX 5144-C1	Madagascar
**1307**	Japonica	**31.2**	**26.6**	**23.7**	LUDAN::IRGC 64189-C1	Philippines
**1310**	Japonica	**31.8**	**23.9**	**18.1**	M 204::GERVEX 1665-C1	United States
**1312**	Japonica	**41.6**	**39.0**	**-**	MAIORAL::GERVEX 1069-C1	Portugal
**1313**	Japonica	**31.2**	**28.9**	**22.8**	MANANELATRA 520::GERVEX 8445-C1	Madagascar
**1314**	Japonica	**29.8**	**25.7**	**22.6**	MANGAVAVA FOTSILANSTSIKA 1177:: GERVEX 5719-C1	Madagascar
**1320**	Japonica	**38.7**	**31.1**	**27.7**	OLCENENGO::GERVEX 41-C1	Italy
**1321**	Japonica	**27.1**	**24.3**	**18.9**	ORYZICA SABANA 6::C1	Colombia
**1325**	Japonica	**26.5**	**23.8**	**21.7**	PCT 4\SA\4\1>1076-2-4-1-5::C1	Colombia
**1327**	Japonica	**34.1**	**22.3**	**22.8**	PEH PI NUO::IRGC 8266-C1	China
**1329**	Japonica	**32.2**	**24.3**	**21.1**	PRIMAVERA::C1	Brazil
**1332**	Indica	**40.1**	**34.9**	**30.1**	ROJOMENA 1034::GERVEX 8412-C1	Madagascar
**1334**	Japonica	**34.1**	**38.0**	**25.0**	ROVUMA::GERVEX 1245-C1	Portugal
**1339**	Japonica	**33.2**	**28.8**	**20.7**	SOURE::GERVEX 1294-C1	Portugal
**1341**	Indica	**25.3**	**19.6**	**16.2**	SUWEON 280::GERVEX 1306-C1	Republic of Korea
**1342**	Indica	**27.9**	**24.1**	**17.4**	TELIMANI::C1	Mali
**1347**	Indica	**36.6**	**33.7**	**28.0**	TSAKA::IRGC 64934-C1	Bhutan
**1348**	Japonica	**35.9**	**36.3**	**29.4**	TSIPALA 89::GERVEX 8385-C1	Madagascar
**1349**	Indica	**36.5**	**32.4**	**28.1**	TSIPALA FOTSY 1883::GERVEX 5387-C1	Madagascar
**1350**	Indica	**29.1**	**23.1**	**21.2**	UPL RI 5::IRTP 7034-C1	Philippines

**1353**	Japonica	**33.3**	**28.7**	**24.9**	VARY SOMOTRA SIHANAKA::GERVEX 8321-C1	Madagascar
**1358**	Japonica	**27.4**	**27.6**	**20.5**	WAB 56-125::IRTP 19771-C1	Cote d'Ivoire
**1360**	Japonica	**30.3**	**24.7**	**20.7**	WAB 706-3-4-K4-KB-1::C1	Cote d'Ivoire
**1361**	Indica	**26.6**	**24.8**	**17.3**	WAS 169-B-B-4-2-1::C1	Senegal
**1362**	Indica	**25.2**	**23.4**	**17.5**	WAS 181-B-6-3::C1	Senegal
**1365**	Indica	**27.0**	**23.0**	**18.3**	WAS 194-B-3-2-5::C1	Senegal
**1370**	Indica	**27.4**	**24.2**	**18.1**	WAS 208-B-B-5-1-1-3::C1	Senegal
**1372**	Indica	**25.8**	**23.9**	**19.4**	WAS 30-11-4-6-2-2-1::C1	Senegal
**1374**	Japonica	**30.4**	**28.9**	**21.6**	YANCAOUSSA::IRGC 16071-C1	Cote d'Ivoire
**1377**	Japonica	**31.5**	**24.0**	**22.3**	ZENA::GERVEX 101-C1	Italy
**1387**	Indica	**37.6**	**34.1**	**27.3**	ARC 18262::IRGC 42363-1	India
**1388**	Japonica	**32.1**	**32.6**	**25.5**	BAKAW::IRGC 11169-1	Philippines
**1390**	Japonica	**33.4**	**29.7**	**21.5**	BORUBI::IRGC25181-1	Indonesia
**1399**	Indica	**37.5**	**31.5**	**23.8**	LUA NHE DEN::IRGC 16724-1	Viet Nam
**1400**	Indica	**38.5**	**31.6**	**26.8**	MAHUDU KIRIYAL::IRGC 40844-1	Sri Lanka
**1409**	Indica	**38.9**	**29.4**	**21.8**	11-049::IRGC 36400-1	Sri Lanka
**1416**	Indica	**38.8**	**31.3**	**26.2**	BEKASAKA 158::IRGC 68361-1	Madagascar
**1419**	Japonica	**28.6**	**32.0**	**22.0**	BOMALASANG::IRGC 12052-1	Philippines
**1420**	Japonica	**26.1**	**24.2**	**18.1**	CINA::IRGC 27116-1	Indonesia
**1431**	Indica	**30.2**	**22.7**	**20.0**	JUMA 58::IRGC 117010-1	Dominican Republic
**1435**	Indica	**39.4**	**30.8**	**27.3**	KUMBIPHOU::IRGC 51990-1	India
**1436**	Indica	**32.6**	**24.1**	**18.5**	KUMDIN::IRGC 74756-1	India
**1438**	Japonica	**29.2**	**30.1**	**20.6**	NEP ME HOA BINH::IRGC 78366-1	Viet Nam
**1442**	Japonica	**29.1**	**30.0**	**22.0**	PATO DE GALLINAZO Y 5371::IRGC 5766-1	Australia
**1445**	Japonica	**35.1**	**32.6**	**25.8**	P. TINGAGEW DAYKET QAY DAYON::IRGC 8046-1	Philippines
**1449**	Indica	**33.4**	**33.1**	**22.1**	SPTLR 7201-PRE 26-2-GM-4::IRGC 117356-1	Thailand
**1453**	Japonica	**29.9**	**28.6**	**21.3**	TORO::IRGC 50596-1	Brazil

- Missing data
