# Supplementary material for: Genome-wide association mapping combined with gene-based haplotype analysis identify a novel gene for shoot length in rice (Oryza sativa L.)
Source: Theor Appl Genet. 2023 Nov 20;136(12):251. doi: 10.1007/s00122-023-04497-6 (PMC10661777; doi:10.1007/s00122-023-04497-6)
Supplement: Supplementary file 2 — (PDF 71 KB) [file 122_2023_4497_MOESM2_ESM.pdf]

Table S2 Primer sequences for qRT-PCR used in this study

Gene	Forward Primer 5'-3'	Reverse Primer 5'-3'
Actin	CACATTCCAGCAGATGTGGA	GCGATAACAGCTCCTCTTGG
LOC_Os01g68460	GGAAAGCTCTCGTTTCGCA	GGAAGCTGTTGCATTCCACA
LOC_Os01g68500	ATCGCGGATTTCAGGCACGA	AGACGCCCGACAGCGACGCGAT
