# Supplementary material for: Genome-wide association mapping combined with gene-based haplotype analysis identify a novel gene for shoot length in rice (Oryza sativa L.)
Source: Theor Appl Genet. 2023 Nov 20;136(12):251. doi: 10.1007/s00122-023-04497-6 (PMC10661777; doi:10.1007/s00122-023-04497-6)
Supplement: Supplementary file 3 — (PDF 768 KB) [file 122_2023_4497_MOESM3_ESM.pdf]

Table S3 The sequence information of *LOC_Os01g68460* and *LOC_Os01g68500* within the *qSL-1f* region on chromosome 1

Positon (bp)	Reference	Alternative	1003	1006	1008	1015	1037	1043	1044	1047	1048	1054	1064	1074	1075	1079	1087	1108	1114	1117	1120	1125
LOC_Os01g68460																						
39781104	C	T	0/0	0/0	0/0	0/0	0/0	0/0	0/0	0/0	0/0	0/0	0/0	0/0	0/0	0/0	0/0	0/0	0/0	0/0	0/0	0/0
39781249	G	A	0/1	0/0	1/1	0/0	0/0	0/0	0/0	0/0	0/0	0/0	0/0	0/0	0/1	0/0	0/0	0/0	0/0	1/1	0/0	0/0
39783117	TGAA	T	0/0	0/0	0/0	0/0	0/0	0/0	0/0	0/0	0/0	0/0	0/0	0/0	0/0	0/0	0/0	0/0	0/0	0/0	0/0	0/0
39783133	G	A	0/0	0/0	0/0	0/0	0/0	0/0	0/0	0/0	0/0	0/0	0/0	0/0	0/0	0/0	0/0	0/0	0/0	0/0	0/0	0/0
39783241	G	A	0/0	0/0	0/0	1/1	0/0	0/0	0/0	1/1	1/1	0/0	0/0	0/0	0/1	1/1	0/0	0/0	1/1	0/0	0/0	1/1
39783314	T	G	0/0	0/0	0/0	1/1	0/0	0/0	0/0	1/1	1/1	0/0	0/0	0/0	0/1	1/1	0/0	0/0	1/1	0/0	0/0	1/1
39783340	G	GACAGGCC CGTCGATC GCGGAGA	0/0	0/0	0/0	0/0	0/0	0/0	0/0	0/0	0/0	0/0	0/0	0/0	0/0	0/0	0/0	0/0	0/0	0/0	0/0	0/0
39783341	G	A	0/1	0/0	1/1	0/0	1/1	0/0	0/0	0/0	0/0	0/0	0/0	0/0	0/1	0/0	0/0	1/1	0/0	1/1	0/0	0/0
39783479	T	C	0/0	0/0	0/0	0/0	0/0	0/0	0/0	0/0	0/0	0/0	0/0	0/0	0/0	0/0	0/0	0/0	0/0	0/0	0/0	0/0
39783511	T	G	0/0	0/0	0/0	0/0	0/0	0/0	0/0	0/0	0/0	0/0	0/0	0/0	0/0	0/0	0/0	0/0	0/0	0/0	0/0	0/0
39783515	T	G	0/0	0/0	0/0	0/0	0/0	0/1	0/0	0/0	0/0	0/1	0/0	0/0	0/0	0/0	0/0	0/0	0/0	0/0	0/0	0/0
39783632	G	T	0/0	0/0	0/0	0/0	0/0	0/0	0/0	0/0	0/0	0/0	0/0	0/0	0/0	0/0	0/0	0/0	0/0	0/0	0/0	0/0
39783699	C	G	0/0	0/0	0/0	1/1	0/0	0/0	0/0	1/1	1/1	0/0	0/0	0/0	0/1	1/1	0/0	0/0	1/1	0/0	0/1	1/1
39783800	G	GAA	0/0	0/0	0/0	0/0	0/0	0/0	0/0	0/0	0/0	0/0	0/0	0/0	0/0	0/0	0/0	0/0	0/0	0/0	0/0	0/0
39783803	G	A	0/0	0/0	1/1	0/0	1/1	0/0	0/0	0/0	./.	0/0	0/0	0/0	0/1	0/0	0/0	1/1	0/0	1/1	./.	0/0
39783804	A	AGCG	0/0	0/0	0/0	0/0	0/0	0/0	0/0	0/0	./.	0/0	0/0	0/0	0/0	0/0	0/0	0/0	0/0	./.	./.	0/0

39783806	AG	AGG,AGGG,AGGGG,A,GG,AGGGGG	0/1	0/0	0/1	0/0	0/4	0/0	0/2	2/2	./.	0/4	0/0	0/0	3/6	2/3	1/1	1/1	0/1	./.	1/1	2/3
39783814	G	GA,GGA	0/0	0/0	0/0	0/0	0/0	0/0	0/0	0/0	0/0	0/0	1/1	0/0	0/0	0/0	0/0	0/0	0/0	0/0	0/0	0/0
39783817	A	G	0/0	0/0	0/0	0/0	0/0	0/0	0/0	0/0	0/0	0/0	0/0	0/0	0/0	0/0	0/0	0/0	0/0	0/0	0/0	0/0
39783844	A	G	0/0	0/0	0/0	1/1	0/0	0/0	0/0	1/1	1/1	0/0	0/0	0/0	0/1	1/1	0/0	0/0	1/1	0/0	0/0	1/1
39783880	A	G	0/1	0/0	1/1	0/0	1/1	0/0	0/0	0/0	0/0	0/0	0/0	0/0	0/1	0/0	0/0	1/1	0/0	1/1	0/0	0/0
39783889	G	C	0/0	0/0	0/0	0/0	0/0	0/0	0/0	0/0	0/0	0/0	0/0	0/0	0/0	0/0	0/0	0/0	0/0	0/0	0/0	0/0
39783926	C	T	0/1	0/0	1/1	0/0	1/1	0/0	0/0	0/0	0/0	0/0	0/0	0/0	0/1	0/0	0/0	1/1	0/0	1/1	0/0	0/0
39783967	C	A	0/0	0/0	0/0	1/1	0/0	0/0	0/0	1/1	1/1	0/0	0/0	0/0	0/1	1/1	0/0	0/0	1/1	0/0	0/0	1/1
39783994	AT	A	0/0	0/0	0/0	0/0	0/0	./.	0/0	0/0	0/0	0/0	0/0	0/0	0/0	0/0	0/0	0/0	0/0	0/0	0/0	0/0
39783995	T	A,*	0/0	0/0	0/0	1/1	1/1	./.	0/0	1/1	1/1	0/0	0/0	0/0	0/1	1/1	0/0	1/1	./.	0/0	0/0	1/1
39783996	T	A	0/0	0/0	0/0	0/0	0/0	./.	0/0	0/0	0/0	0/0	0/0	0/0	0/0	0/0	0/0	0/0	0/0	0/0	0/0	0/0
39784002	T	C	0/0	0/0	0/0	1/1	0/0	0/0	0/0	1/1	1/1	0/0	0/0	0/0	0/1	1/1	0/0	0/0	./.	0/0	0/0	1/1
39784160	T	G	0/1	0/0	1/1	1/1	1/1	0/0	0/0	1/1	1/1	0/0	0/0	0/0	1/1	1/1	0/0	1/1	1/1	1/1	0/0	1/1
39784164	C	A	0/0	0/0	0/0	0/0	0/0	0/0	0/0	0/0	0/0	0/0	0/0	0/0	0/0	0/0	0/0	0/0	0/0	0/0	0/0	0/0
39784174	A	G	0/0	0/0	0/0	0/0	0/0	0/0	0/0	0/0	0/0	0/0	0/0	0/0	0/0	0/0	0/0	0/0	0/0	0/0	0/0	0/0
39784241	T	C	0/1	0/0	1/1	1/1	1/1	0/0	0/0	1/1	1/1	0/0	0/0	0/0	1/1	1/1	0/0	1/1	1/1	1/1	0/1	1/1
39784244	G	A	0/0	0/0	0/0	1/1	0/0	0/0	0/0	1/1	1/1	0/0	0/0	0/0	0/1	1/1	0/0	0/0	1/1	0/0	0/1	1/1
39784262	G	T	0/0	0/0	0/0	0/0	0/0	0/0	0/0	0/0	0/0	0/0	0/0	0/0	0/0	0/0	0/0	0/0	0/0	0/0	0/0	0/0
39784385	G	A	0/1	0/0	1/1	0/0	1/1	0/0	0/0	0/0	0/0	0/0	0/0	0/0	0/1	0/0	0/0	1/1	0/0	1/1	0/0	0/0
39784436	G	A	0/1	0/0	1/1	0/0	0/0	0/0	0/0	0/0	0/0	0/0	0/0	0/0	0/1	0/0	0/0	0/0	0/0	1/1	0/0	0/0
39784447	T	C	0/0	0/0	0/0	0/0	0/0	0/0	0/0	0/0	0/0	0/0	0/0	0/0	0/0	0/0	0/0	0/0	0/0	0/0	0/0	0/0
39784484	A	G	0/1	0/0	1/1	0/0	1/1	0/0	0/0	0/0	0/0	0/0	0/0	0/0	0/1	0/0	0/0	1/1	0/0	1/1	0/0	0/0
39784538	G	A	0/0	0/0	0/0	0/0	0/0	0/0	0/0	0/0	0/0	0/0	0/0	0/0	0/0	0/0	0/0	0/0	0/0	0/0	0/0	0/0
39784560	G	A	0/1	0/0	1/1	1/1	1/1	0/0	0/0	1/1	1/1	0/0	0/0	0/0	1/1	1/1	0/0	1/1	1/1	1/1	0/0	1/1
39784731	C	A	0/0	0/0	0/0	1/1	0/0	0/0	0/0	1/1	1/1	0/0	0/0	0/0	0/1	1/1	0/0	0/0	1/1	0/0	0/0	1/1
39784739	C	CA,CAA	0/1	0/0	1/1	0/0	0/0	0/0	0/0	0/0	0/0	0/0	0/0	0/0	0/1	0/0	0/0	0/0	0/0	1/1	0/0	0/0

39784872	CACTA	C	0/1	0/0	1/1	1/1	1/1	0/0	0/0	1/1	1/1	0/0	0/0	0/0	1/1	1/1	1/1	1/1	1/1	1/1	0/0	1/1
39784939	AT	A	0/0	0/0	0/0	1/1	0/0	0/0	0/0	1/1	1/1	0/0	0/0	0/0	0/1	1/1	0/0	0/0	1/1	0/0	0/0	1/1
39784966	C	G	0/0	0/0	0/0	1/1	0/0	0/0	0/0	1/1	1/1	0/0	0/0	0/0	0/1	1/1	0/0	0/0	1/1	0/0	0/0	1/1
39784983	C	T	0/0	0/0	0/0	0/0	0/0	0/0	0/0	0/0	0/0	0/0	0/0	0/0	0/0	0/0	0/0	0/0	0/0	0/0	0/0	0/0
39785125	A	C	0/1	0/0	1/1	1/1	1/1	0/0	0/0	1/1	1/1	0/0	0/0	0/0	1/1	1/1	0/0	1/1	1/1	1/1	0/0	1/1
39785272	A	T	0/1	0/0	1/1	1/1	1/1	0/0	0/0	1/1	1/1	0/0	0/0	0/0	1/1	1/1	0/0	1/1	1/1	1/1	0/0	1/1
39785344	G	GT	0/1	0/0	1/1	1/1	1/1	0/0	0/0	1/1	1/1	0/0	0/0	0/0	1/1	1/1	0/0	1/1	1/1	1/1	0/0	1/1
39785377	A	C	0/0	0/0	0/0	0/0	0/0	0/0	0/0	0/0	./.	0/0	0/0	0/0	0/0	0/0	0/0	0/0	./.	0/0	0/0	0/0
39785380	A	AGAAAAAT	0/0	0/0	0/0	1/1	0/0	0/0	0/0	1/1	1/1	0/0	0/0	0/0	0/1	1/1	0/0	0/0	1/1	0/0	0/0	1/1
39785412	C	T	0/0	0/0	0/0	0/0	0/0	0/0	0/0	0/0	0/0	0/0	1/1	0/0	0/0	0/0	0/0	0/0	0/0	0/0	0/0	0/0
39785476	C	T	0/1	0/0	1/1	0/0	1/1	0/0	0/0	0/0	0/0	0/0	0/0	0/0	0/1	0/0	0/0	1/1	0/0	1/1	0/0	0/0
39785477	C	T	0/0	0/0	0/0	0/0	0/0	0/0	0/0	0/0	0/0	0/0	0/0	0/0	0/0	0/0	0/0	0/0	0/0	0/0	0/0	0/0
39785478	G	A	0/0	0/0	0/0	0/0	0/0	0/0	0/0	0/0	0/0	0/0	0/0	0/0	0/0	0/0	0/0	0/0	0/0	0/0	0/0	0/0
39785479	G	A	0/0	0/0	0/0	0/0	1/1	0/0	0/0	0/0	0/0	0/0	0/0	0/0	0/0	0/0	0/0	1/1	0/0	0/0	0/0	0/0
39785530	T	C	0/1	0/0	1/1	1/1	1/1	0/0	0/0	1/1	1/1	0/0	0/0	0/0	1/1	1/1	0/0	1/1	1/1	1/1	0/0	1/1
39785531	C	T	0/0	0/0	0/0	0/0	0/0	0/0	0/0	0/0	0/0	0/0	0/0	0/0	0/0	0/0	0/0	0/0	0/0	0/0	0/0	0/0
39785567	G	A	0/0	0/0	0/0	0/0	0/0	0/0	0/0	0/0	0/0	0/0	0/0	0/0	0/0	0/0	0/0	0/0	0/0	0/0	0/0	0/0
39785570	G	A	0/0	0/0	0/0	0/0	0/0	0/0	0/0	0/0	0/0	0/0	0/0	0/0	0/0	0/0	0/0	0/0	0/0	0/0	0/0	0/0
39785595	C	T	0/1	0/0	1/1	0/0	1/1	0/0	0/0	0/0	0/0	0/0	0/0	0/0	0/1	0/0	0/0	1/1	0/0	1/1	0/0	0/0
39785602	G	A	0/0	0/0	0/0	0/0	0/0	0/0	0/0	0/0	0/0	0/0	0/0	0/0	0/0	0/0	0/0	0/0	0/0	0/0	0/0	0/0
39785610	T	C	0/1	0/0	1/1	1/1	1/1	0/0	0/0	1/1	1/1	0/0	0/0	0/0	1/1	1/1	0/0	1/1	1/1	1/1	0/0	1/1
39785619	C	A	0/1	0/0	1/1	0/0	1/1	0/0	0/0	0/0	0/0	0/0	0/0	0/0	0/1	0/0	0/0	1/1	0/0	1/1	0/0	0/0
39785645	T	A	0/1	0/0	1/1	0/0	1/1	0/0	0/0	0/0	0/0	0/0	0/0	0/0	0/1	0/0	0/0	1/1	0/0	1/1	0/0	0/0
39785651	C	A	0/0	0/0	0/0	1/1	0/0	0/0	0/0	1/1	1/1	0/0	0/0	0/0	0/1	1/1	0/0	0/0	1/1	0/0	0/0	1/1
39785657	T	C	0/1	0/0	1/1	1/1	1/1	0/0	0/0	1/1	1/1	0/0	0/0	0/0	1/1	1/1	0/0	1/1	1/1	1/1	0/0	1/1
39785694	T	TC	0/0	0/0	0/0	1/1	0/0	0/0	0/0	1/1	1/1	0/0	0/0	0/0	0/1	1/1	0/0	0/0	1/1	0/0	0/0	1/1
39785730	C	T	0/0	0/0	0/0	0/0	0/0	0/0	0/0	0/0	0/0	0/0	0/0	0/0	0/0	0/0	0/0	0/0	0/0	0/0	0/0	0/0
39785751	C	T	0/0	0/0	0/0	0/0	0/0	0/0	0/0	0/0	0/0	0/0	0/0	0/0	0/0	0/0	0/0	0/0	0/0	0/0	0/0	0/0
39785753	A	G	0/1	0/0	1/1	1/1	1/1	0/0	0/0	1/1	1/1	0/0	0/0	0/0	1/1	1/1	0/0	1/1	1/1	1/1	0/0	1/1
39785801	G	A	1/1	0/0	0/0	0/0	0/0	1/1	1/1	0/0	0/0	0/0	0/0	0/0	0/0	0/0	1/1	0/0	0/0	0/0	1/1	0/0
39785831	T	C	0/1	0/0	1/1	1/1	1/1	0/0	0/0	1/1	1/1	0/0	0/0	0/0	1/1	1/1	0/0	1/1	1/1	1/1	0/0	1/1
39785847	G	A	0/0	0/0	0/0	1/1	0/0	0/0	0/0	1/1	1/1	0/0	0/0	0/0	0/1	1/1	0/0	0/0	1/1	0/0	0/0	1/1
39785849	A	C	0/1	0/0	1/1	1/1	1/1	0/0	0/0	1/1	1/1	0/0	0/0	0/0	1/1	1/1	0/0	1/1	1/1	1/1	0/0	1/1
39785850	G	A	0/0	0/0	0/0	0/0	0/0	0/0	0/0	0/0	0/0	0/0	0/0	0/0	0/0	0/0	0/0	0/0	0/0	0/0	0/0	0/0
39785856	G	A	0/0	0/0	0/0	1/1	0/0	0/0	0/0	1/1	1/1	0/0	0/0	0/0	0/1	1/1	0/0	0/0	1/1	0/0	0/0	1/1

39785868	C	T	0/1	0/0	1/1	0/0	1/1	0/0	0/0	0/0	0/0	0/0	0/0	0/0	0/1	0/0	0/0	1/1	0/0	1/1	0/0	0/0
39785869	T	A	0/0	0/0	0/0	0/0	0/0	0/0	0/0	0/0	0/0	0/0	0/0	0/0	0/0	1/1	0/0	0/0	1/1	0/0	0/0	0/0
39785874	G	A,GT	0/1	0/0	1/1	0/0	0/0	0/0	0/0	0/0	0/0	0/0	0/0	0/0	0/1	0/0	0/0	0/0	0/0	1/1	0/0	0/0
39785896	A	G	0/1	0/0	1/1	1/1	1/1	0/0	0/0	1/1	1/1	0/0	0/0	0/0	1/1	1/1	0/0	1/1	1/1	1/1	0/0	1/1
39785947	G	GAT	0/0	0/0	0/0	0/0	0/0	0/0	0/0	0/0	0/0	0/0	0/0	0/0	0/0	0/0	0/0	0/0	0/0	0/0	0/0	0/0
39785952	G	A	0/0	0/0	1/1	0/0	1/1	0/0	0/0	0/0	0/0	0/0	0/0	0/0	0/1	0/0	0/0	1/1	0/0	1/1	0/0	0/0
39785965	A	G	0/1	0/0	1/1	1/1	1/1	0/0	0/0	1/1	1/1	0/0	0/0	0/0	1/1	1/1	0/0	1/1	1/1	1/1	0/0	1/1
39785975	T	C	0/0	0/0	0/0	1/1	0/0	0/0	0/0	1/1	1/1	0/0	0/0	0/0	0/1	1/1	0/0	0/0	1/1	0/0	0/0	1/1
39785988	AT	ATT,A	0/1	0/0	1/1	0/0	1/1	0/0	0/0	0/0	0/0	0/0	0/0	0/0	0/1	0/0	0/0	1/1	0/0	1/1	0/0	0/0
39786004	G	C	0/0	0/0	0/0	0/0	0/0	0/0	0/0	0/0	0/0	0/0	0/0	0/0	0/0	0/0	0/0	0/0	0/0	0/0	0/0	0/0
39786012	G	T	0/0	0/0	1/1	0/0	1/1	0/0	0/0	0/0	0/0	0/0	0/0	0/0	0/1	0/0	0/0	1/1	0/0	1/1	0/0	0/0
39786048	G	A	0/0	0/0	0/0	0/0	0/0	0/0	0/0	0/0	0/0	0/0	0/0	0/0	0/0	0/0	0/0	0/0	0/0	0/0	0/0	0/0
39786050	G	A	0/0	0/0	0/0	0/0	0/0	0/0	0/0	0/0	0/0	0/0	0/0	0/0	0/0	0/0	0/0	0/0	0/0	0/0	0/0	0/0
39786055	G	A	0/0	0/0	0/0	0/0	0/0	0/0	0/0	0/0	0/0	0/0	0/0	0/0	0/0	0/0	0/0	0/0	0/0	0/0	0/0	0/0
39786074	G	A	0/1	0/0	1/1	1/1	1/1	0/0	0/0	1/1	1/1	0/0	0/0	0/0	1/1	1/1	0/0	1/1	1/1	1/1	0/0	1/1
39786106	A	G	0/1	0/0	1/1	1/1	1/1	0/0	0/0	1/1	1/1	0/0	0/0	0/0	1/1	1/1	0/0	1/1	1/1	1/1	0/0	1/1
39786111	A	T	0/1	0/0	1/1	1/1	1/1	0/0	0/0	1/1	1/1	0/0	0/0	0/0	1/1	1/1	0/0	1/1	1/1	1/1	0/0	1/1
39786167	GGAA	G,GA	0/0	0/0	1/1	0/0	./.	0/0	0/0	./.	./.	0/0	./.	0/0	./.	./.	0/0	./.	./.	./.	0/0	./.
39786168	GAAAA	GA,GAAA,GAA,*	0/1	0/0	4/4	3/3	1/1	0/0	0/0	3/3	3/3	0/0	2/2	0/0	1/3	3/3	0/0	1/1	3/3	1/1	0/2	3/3
39786187	G	A	0/0	0/0	0/0	0/0	0/0	0/0	0/0	1/1	1/1	0/0	0/0	0/0	0/1	0/0	0/0	0/0	0/0	0/0	0/0	0/0
39786196	G	A	0/0	0/0	0/0	0/0	0/0	0/0	0/0	0/0	0/0	0/0	0/0	0/0	0/0	0/0	0/0	0/0	0/0	0/0	0/0	0/0
39786198	G	A	0/0	0/0	0/0	0/0	0/0	0/0	0/0	0/0	0/0	0/0	0/0	0/0	0/0	0/0	0/0	0/0	0/0	0/0	0/0	0/0
39786208	C	T	0/0	0/0	0/0	0/0	0/0	0/0	0/0	0/0	0/0	0/0	0/0	0/0	0/0	0/0	0/0	0/0	0/0	0/0	0/0	0/0
39786241	C	T	0/0	0/0	0/0	0/0	0/0	0/0	0/0	./.	0/0	0/0	0/0	0/0	0/0	./.	0/0	0/0	0/0	0/0	0/0	0/0
39786247	T	TTCGTAC	0/0	0/0	0/0	1/1	0/0	0/0	0/0	1/1	1/1	0/0	0/0	0/0	0/1	1/1	0/0	0/0	1/1	0/0	0/1	1/1

39786251	T	TACTCGTACGTTATATATAA	0/0	0/0	0/0	0/0	0/0	0/0	0/0	0/0	0/0	0/0	0/0	0/0	0/0	0/0	0/0	0/0	0/0	0/0	0/0	0/0
39786281	A	G	0/0	0/0	0/0	1/1	0/0	0/0	0/0	1/1	1/1	0/0	0/0	0/0	0/1	1/1	0/0	0/0	1/1	0/0	0/1	1/1
39786363	T	C	0/0	0/0	0/0	1/1	0/0	0/0	0/0	1/1	1/1	0/0	0/0	0/0	0/1	1/1	0/0	0/0	1/1	0/0	0/0	1/1
39786485	G	A	0/1	0/0	1/1	1/1	1/1	0/0	0/0	1/1	1/1	0/0	0/0	0/0	1/1	0/1	0/0	1/1	1/1	1/1	0/0	1/1
39786567	A	G	0/1	0/0	1/1	1/1	1/1	0/0	0/0	1/1	1/1	0/0	0/0	0/0	1/1	0/1	0/0	1/1	1/1	1/1	0/1	1/1
39786573	T	C	0/0	0/0	0/0	1/1	0/0	0/0	0/0	0/0	0/0	0/0	0/0	0/0	0/0	0/0	0/0	0/0	0/0	0/0	0/0	0/0
39786646	AT	A	0/1	0/0	1/1	1/1	1/1	0/0	0/0	1/1	1/1	0/0	0/0	0/0	1/1	1/1	0/0	1/1	1/1	1/1	0/1	1/1
39786687	T	A	0/0	0/0	0/0	1/1	0/0	0/0	0/0	1/1	1/1	0/0	0/0	0/0	0/1	1/1	0/0	0/0	1/1	0/0	0/1	1/1
39786691	G	A	0/0	0/0	0/0	0/0	0/0	0/0	0/0	0/0	0/0	0/0	0/0	0/0	0/0	0/0	0/0	0/0	0/0	0/0	0/0	0/0
39786899	G	GTATA	0/0	0/0	0/0	0/0	0/0	0/0	0/0	0/0	0/0	0/0	0/0	0/0	0/0	0/0	0/0	0/0	0/0	0/0	0/0	0/0
39787000	A	G	0/0	0/0	0/0	1/1	0/0	0/0	0/0	1/1	1/1	0/0	0/0	0/0	0/1	1/1	0/0	0/0	1/1	0/0	0/0	1/1
39787160	C	T	0/0	0/0	0/0	0/0	0/0	0/0	0/0	0/0	0/0	0/0	0/0	0/0	0/0	0/0	0/0	0/0	0/0	0/0	0/0	0/0
39787214	T	C	0/1	0/0	1/1	0/0	1/1	0/0	0/0	0/0	0/0	0/0	0/0	0/0	0/1	0/0	0/0	1/1	0/0	1/1	0/0	0/0
39787229	C	T	0/0	0/0	0/0	1/1	0/0	0/0	0/0	1/1	1/1	0/0	0/0	0/0	0/1	1/1	0/0	0/0	1/1	0/0	0/0	1/1
39787238	T	C	0/0	0/0	0/0	0/0	0/0	0/0	0/0	0/0	0/0	0/0	0/0	0/0	0/0	0/0	0/0	0/0	0/0	0/0	0/0	0/0
39787259	A	G	0/1	0/0	1/1	1/1	1/1	0/0	0/0	1/1	1/1	0/0	0/0	0/0	1/1	1/1	0/0	1/1	1/1	1/1	0/0	1/1
39787263	C	T	0/1	0/0	1/1	0/0	1/1	0/0	0/0	0/0	0/0	0/0	0/0	0/0	0/1	0/0	0/0	1/1	0/0	1/1	0/0	0/0
39787282	G	A	0/0	0/0	0/0	0/0	1/1	0/0	0/0	0/0	0/0	0/0	0/0	0/0	0/0	0/0	0/0	1/1	0/0	0/0	0/0	0/0
39787296	C	T	0/0	0/0	0/0	0/0	0/0	0/0	0/0	0/0	./.	0/0	0/0	0/0	0/0	./.	0/0	0/0	0/0	0/0	0/0	0/0
39787304	C	CTAT	0/0	0/0	0/0	1/1	0/0	0/0	0/0	1/1	1/1	0/0	0/0	0/0	0/1	1/1	0/0	0/0	1/1	0/0	0/1	1/1
39787327	G	A	0/0	0/0	0/0	1/1	0/0	0/0	0/0	1/1	1/1	0/0	0/0	0/0	0/1	1/1	0/0	0/0	1/1	0/0	0/0	1/1
39787336	G	A	0/0	0/0	0/0	0/0	0/0	0/0	0/0	0/0	0/0	0/0	0/0	0/0	0/0	0/0	0/0	0/0	0/0	0/0	0/0	0/0
39787347	A	G	0/1	0/0	1/1	1/1	1/1	0/0	0/0	1/1	1/1	0/0	0/0	0/0	1/1	1/1	0/0	1/1	1/1	1/1	0/0	1/1
39787351	T	C,TG	0/0	0/0	0/0	0/0	0/0	0/0	0/0	0/0	0/0	0/0	0/0	0/0	0/0	0/0	0/0	0/0	0/0	0/0	0/0	0/0
39787355	G	A	0/0	0/0	0/0	0/0	0/0	0/0	0/0	0/0	0/0	0/0	0/0	0/0	0/0	0/0	0/0	0/0	0/0	0/0	0/0	0/0
39787386	A	G	0/0	0/0	0/0	1/1	0/0	0/0	0/0	1/1	1/1	0/0	0/0	0/0	0/1	1/1	0/0	0/0	1/1	0/0	0/0	1/1
39787414	AT	A	0/0	0/0	0/0	0/0	0/0	0/0	0/0	0/0	0/0	0/0	0/0	0/0	0/0	0/0	0/0	0/0	0/0	0/0	0/0	0/0
39787431	C	T	0/1	0/0	1/1	0/0	1/1	0/0	0/0	0/0	0/0	0/0	0/0	0/0	0/1	0/0	0/0	1/1	0/0	1/1	0/0	0/0
39787438	G	A	0/0	0/0	0/0	1/1	0/0	0/0	0/0	1/1	1/1	0/0	0/0	0/0	0/1	1/1	0/0	0/0	1/1	0/0	0/0	1/1
39787730	C	T	0/0	0/0	0/0	0/0	0/0	0/0	0/0	0/0	0/0	0/0	0/0	0/0	0/0	0/0	0/0	0/0	0/0	0/0	0/0	0/0
39787771	C	T	0/1	0/0	1/1	1/1	1/1	0/0	0/0	1/1	1/1	0/0	0/0	0/0	1/1	1/1	0/0	1/1	1/1	1/1	0/1	1/1
39787807	T	C	0/0	0/0	0/0	0/0	0/0	0/0	0/0	0/0	0/0	0/0	0/0	0/0	0/0	0/0	0/0	0/0	0/0	0/0	0/0	0/0
39787845	G	A	0/0	0/0	0/0	0/0	0/0	0/0	0/0	0/0	0/0	0/0	0/0	0/0	0/0	0/0	0/0	0/0	0/0	0/0	0/0	0/0

39787965	T	A	0/1	0/0	1/1	1/1	1/1	0/0	0/0	1/1	1/1	0/0	0/0	0/0	1/1	1/1	0/0	1/1	1/1	1/1	0/0	1/1
39788011	C	T	0/0	0/0	0/0	0/0	0/0	0/0	0/0	0/0	0/0	0/0	0/0	0/0	0/0	0/0	0/0	0/0	0/0	0/0	0/0	0/0
39788038	A	G	0/1	0/0	1/1	1/1	1/1	0/0	0/0	1/1	1/1	0/0	0/0	0/0	1/1	1/1	0/0	1/1	1/1	1/1	0/1	1/1
39788087	C	T	0/0	0/0	0/0	0/0	0/0	0/0	0/0	0/0	0/0	0/0	0/0	0/0	0/0	0/0	0/0	0/0	0/0	0/0	0/0	0/0
39788105	G	A	0/1	0/0	1/1	0/0	0/0	0/0	0/0	0/0	0/0	0/0	0/0	0/0	0/1	0/0	0/0	0/0	0/0	1/1	0/0	0/0
39788124	G	A	0/0	0/0	0/0	0/0	1/1	0/0	0/0	0/0	0/0	0/0	0/0	0/0	0/0	0/0	0/0	0/0	0/0	0/0	0/0	0/0
39788139	G	A	0/0	0/0	0/0	0/0	0/0	0/0	0/0	0/0	0/0	0/0	0/0	0/0	0/0	0/0	0/0	0/0	0/0	0/0	0/0	0/0
39788143	G	A	0/0	0/0	0/0	0/0	1/1	0/0	0/0	0/0	0/0	0/0	0/0	0/0	0/0	0/0	0/0	1/1	0/0	0/0	0/0	0/0
39788149	G	A	0/0	0/0	0/0	0/0	0/0	0/0	0/0	0/0	0/0	0/0	0/0	0/0	0/0	0/0	0/0	0/0	0/0	0/0	0/0	0/0
39788172	G	A	0/0	0/0	0/0	0/0	0/0	0/0	0/0	0/0	0/0	0/0	0/0	0/0	0/0	0/0	0/0	0/0	0/0	0/0	0/0	0/0
39788201	A	G	0/1	0/0	1/1	1/1	1/1	0/0	0/0	1/1	1/1	0/0	0/0	0/0	1/1	1/1	0/0	1/1	1/1	1/1	0/0	1/1
39788204	G	A	0/1	0/0	1/1	1/1	1/1	0/0	0/0	1/1	1/1	0/0	0/0	0/0	1/1	1/1	0/0	1/1	1/1	1/1	0/0	1/1
39788242	C	T	0/0	0/0	0/0	0/0	0/0	0/0	0/0	0/0	0/0	0/0	0/0	0/0	0/0	0/0	0/0	0/0	0/0	0/0	0/0	0/0
39788254	T	C	0/1	0/0	1/1	0/0	0/0	0/0	0/0	0/0	0/0	0/0	0/0	0/0	0/1	0/0	0/0	0/0	0/0	1/1	0/0	0/0
39788257	C	T	0/0	0/0	0/0	1/1	0/0	0/0	0/0	1/1	1/1	0/0	0/0	0/0	0/1	1/1	0/0	0/0	1/1	0/0	0/0	1/1
39788274	T	TA,TAA	0/1	0/0	1/1	1/1	1/1	0/0	0/0	1/1	1/1	0/0	0/0	0/0	1/1	1/1	0/0	1/1	0/1	1/1	0/0	0/1
39788342	G	A	0/1	0/0	1/1	1/1	1/1	0/0	0/0	1/1	1/1	0/0	0/0	0/0	1/1	1/1	0/0	1/1	1/1	1/1	0/0	1/1
LOC_Os01g68500																						
39801532	G	A	0/0	0/0	0/0	1/1	0/0	0/0	0/0	1/1	1/1	0/0	0/0	0/0	0/1	1/1	0/0	0/0	1/1	0/0	0/0	1/1
39801791	C	A	0/0	0/0	0/0	1/1	0/0	0/0	0/0	1/1	1/1	0/0	0/0	0/0	0/1	1/1	0/0	0/0	1/1	0/0	0/0	1/1
39801890	TG	T	0/1	0/0	1/1	0/0	0/0	0/0	0/0	0/0	0/0	0/0	0/0	0/0	0/1	0/0	0/0	0/0	0/0	1/1	0/0	0/0
39802088	G	A	0/0	0/0	0/0	0/0	0/0	0/0	0/0	0/0	0/0	0/0	0/0	0/0	0/0	0/0	0/0	0/0	0/0	0/0	0/0	0/0
39802297	C	T	0/0	0/0	0/0	0/0	0/0	0/0	0/0	0/0	0/0	0/0	0/0	0/0	0/0	0/0	0/0	0/0	0/0	0/0	0/0	0/0
39802311	G	C,GCCCC	0/0	0/0	0/0	0/0	0/0	0/0	0/0	0/0	0/0	0/0	0/0	0/0	0/0	0/0	0/0	0/0	0/0	0/0	0/0	0/0
39802312	C	A	0/0	0/0	0/0	0/0	0/0	0/0	0/0	0/0	0/0	0/0	0/0	0/0	0/0	0/0	0/0	0/0	0/0	0/0	0/0	0/0
39802314	A	C	0/0	0/0	0/0	0/0	0/0	0/1	0/0	0/0	0/0	0/0	0/1	0/0	0/0	0/0	0/0	0/0	0/0	0/1	0/0	0/0
39802319	G	C	0/0	0/0	0/0	0/0	0/0	0/1	0/0	0/0	0/0	0/0	0/1	0/0	0/0	0/0	0/0	0/0	0/0	0/1	0/0	0/0

39802324	A	C	0/0	0/0	0/0	0/0	0/0	0/0	0/0	0/0	0/0	0/0	0/1	0/0	0/0	0/0	0/0	0/0	0/0	0/1	0/0	0/0
39802329	G	C	0/0	0/0	0/0	0/0	0/0	0/0	0/0	0/0	0/0	0/0	0/1	0/0	0/0	0/0	0/0	0/0	0/0	0/1	0/0	0/0
39802332	G	C	0/0	0/0	0/0	0/0	0/0	0/0	0/0	0/0	0/0	0/0	0/1	0/0	0/0	0/0	0/0	0/0	0/0	0/1	0/0	0/0
39802335	G	C	0/0	0/0	0/0	0/0	0/0	0/0	0/0	0/0	0/0	0/0	0/1	0/0	0/0	0/0	0/0	0/0	0/0	0/1	0/0	0/0
39802338	A	C	0/0	0/0	0/0	0/0	0/0	0/0	0/0	0/0	0/0	0/0	0/1	0/0	0/0	0/0	0/0	0/0	0/0	0/1	0/0	0/0
39802341	A	C	0/0	0/0	0/0	0/0	0/0	0/0	0/0	0/0	0/0	0/0	0/1	0/0	0/0	0/0	0/0	0/0	0/0	0/1	0/0	0/0
39802343	CAT	C	0/0	0/0	0/0	0/0	0/0	0/0	0/0	0/0	0/0	0/0	0/0	0/0	0/0	0/0	0/0	0/0	0/0	0/1	0/0	0/0
39802344	A	C,*	0/0	0/0	0/0	0/0	0/0	0/0	0/0	0/0	0/0	0/0	0/0	0/0	0/0	0/0	0/0	0/0	0/0	0/2	0/0	0/0
39802345	T	A,*	0/0	0/0	0/0	0/0	0/0	0/0	0/0	0/0	0/0	0/0	0/0	0/0	0/0	0/0	0/0	0/0	0/0	0/2	0/0	0/0
39804405	G	A	0/0	0/0	0/0	0/0	0/0	0/0	0/0	0/0	0/0	0/0	0/0	0/0	0/0	0/0	0/0	0/0	0/0	0/0	0/0	0/0
39804515	A	T	0/1	0/0	1/1	1/1	1/1	0/0	0/0	1/1	1/1	0/0	0/0	0/0	1/1	1/1	0/0	1/1	1/1	1/1	0/0	1/1
39804521	AG	A	0/0	0/0	0/0	0/0	0/0	0/0	0/0	0/0	0/0	0/0	0/0	0/0	0/0	0/0	0/0	0/0	0/0	0/0	0/0	0/0
39804626	G	C	0/0	0/0	0/0	0/0	0/0	0/0	0/0	0/0	0/0	0/0	0/0	0/0	0/0	0/0	0/0	0/0	0/0	0/0	0/0	0/0
39804738	G	A	0/0	0/0	0/0	0/0	1/1	0/0	0/0	0/0	0/0	0/0	0/0	0/0	0/0	0/0	0/0	1/1	0/0	0/0	0/0	0/0
39804762	A	G	0/0	0/0	0/0	0/0	0/0	0/0	0/0	0/0	0/0	0/0	0/0	0/0	0/0	0/0	0/0	0/0	0/0	0/0	0/0	0/0
39804849	G	T	0/0	0/0	0/0	0/0	0/0	0/0	0/0	0/0	0/0	0/0	0/0	0/0	0/0	0/0	0/0	0/0	0/0	0/0	0/0	0/0
39804945	A	C	0/1	0/0	1/1	1/1	1/1	0/0	0/0	1/1	1/1	0/0	0/0	0/0	1/1	1/1	0/0	1/1	1/1	1/1	0/1	1/1
39805138	C	T	0/0	0/0	0/0	0/0	0/0	0/0	0/0	0/0	./.	0/0	0/0	0/0	0/0	./.	0/0	0/0	./.	0/0	0/0	./.
39805140	G	GT	0/1	0/0	1/1	0/0	1/1	0/0	0/0	./.	./.	0/0	0/0	0/0	0/1	./.	0/0	1/1	./.	1/1	0/0	./.
39805144	T	TTC	0/0	0/0	0/0	1/1	0/0	0/0	0/0	1/1	1/1	0/0	0/0	0/0	0/1	1/1	0/0	0/0	1/1	0/0	0/0	1/1
39805225	C	T	0/0	0/0	0/0	1/1	0/0	0/0	0/0	1/1	1/1	0/0	0/0	0/0	0/1	1/1	0/0	0/0	1/1	0/0	0/1	1/1
39805235	GAAAACA	GA,G	0/0	0/0	./.	0/0	./.	0/0	0/0	0/0	0/0	0/0	0/0	0/0	0/0	0/0	0/0	./.	0/0	./.	0/0	0/0
39805236	AAAAC	A,*	./.	0/0	1/1	0/0	0/1	./.	./.	0/0	0/0	0/0	0/0	0/0	0/0	0/0	0/0	./.	0/0	./.	0/0	0/0
39805237	AAAC	A,*	0/1	0/0	2/2	0/0	0/1	./.	./.	0/0	0/0	0/0	0/0	0/0	0/1	0/0	0/0	1/1	0/0	0/1	0/0	0/0

39805238	AAC	A,*	0/2	0/0	2/2	0/0	0/2	./.	./.	0/0	0/0	0/0	0/0	0/0	0/2	0/0	0/0	2/2	0/0	0/1	0/0	0/0
39805240	CA	C,CAA,CAAA,*	0/1	0/0	4/4	0/0	0/4	3/3	1/1	0/0	0/0	0/0	0/0	0/0	0/4	0/0	0/0	4/4	0/0	0/4	0/1	0/0
39805252	T	A	0/0	0/0	0/0	0/0	0/0	0/0	0/0	0/0	0/0	0/0	0/0	0/0	0/0	0/0	0/0	0/0	0/0	0/0	0/0	0/0
39805255	A	G	0/1	0/0	1/1	1/1	1/1	0/0	0/0	1/1	1/1	0/0	0/0	0/0	1/1	1/1	0/0	1/1	1/1	1/1	0/1	1/1
39805308	G	A	0/1	0/0	1/1	0/0	1/1	0/0	0/0	0/0	0/0	0/0	0/0	0/0	0/1	0/0	0/0	1/1	0/0	1/1	0/0	0/0
39805314	C	T	0/0	0/0	0/0	0/0	0/0	0/0	0/0	0/0	0/0	0/0	0/0	0/0	0/0	0/0	0/0	0/0	0/0	0/0	0/0	0/0
39805334	G	A	0/0	0/0	0/0	0/0	0/0	0/0	0/0	0/0	0/0	0/0	0/0	0/0	0/0	0/0	0/0	0/0	0/0	0/0	0/0	0/0
39805341	A	G	0/1	0/0	1/1	1/1	1/1	0/0	0/0	1/1	1/1	0/0	0/0	0/0	1/1	1/1	0/0	1/1	1/1	1/1	0/0	1/1
39805368	T	TTC	0/0	0/0	./.	0/0	./.	0/0	0/0	0/0	0/0	0/0	0/0	0/0	0/0	0/0	0/0	./.	0/0	0/0	0/0	0/0
39805370	C	CTT,CTTT	0/1	0/0	1/1	0/0	1/1	0/0	0/0	0/0	0/0	0/0	0/0	0/0	0/1	0/0	0/0	1/1	0/0	1/1	0/0	0/0
39805385	C	T	0/1	0/0	1/1	0/0	1/1	0/0	0/0	0/0	0/0	0/0	0/0	0/0	0/1	0/0	0/0	1/1	0/0	1/1	0/0	0/0
39805388	C	G	0/1	0/0	1/1	0/0	1/1	0/0	0/0	0/0	0/0	0/0	0/0	0/0	0/1	0/0	0/0	1/1	0/0	1/1	0/0	0/0
39805400	A	G	0/0	0/0	0/0	1/1	0/0	0/0	0/0	1/1	1/1	0/0	0/0	0/0	0/1	1/1	0/0	0/0	1/1	0/0	0/0	1/1
39805420	G	A	0/1	0/0	1/1	0/0	1/1	0/0	0/0	0/0	0/0	0/0	0/0	0/0	0/1	0/0	0/0	1/1	0/0	1/1	0/0	0/0
39805426	T	C	0/1	0/0	1/1	1/1	1/1	0/0	0/0	1/1	1/1	0/0	0/0	0/0	1/1	1/1	0/0	1/1	1/1	1/1	0/0	1/1
39805439	G	A	0/0	0/0	0/0	0/0	0/0	0/0	0/0	0/0	0/0	0/0	0/0	0/0	0/0	0/0	0/0	0/0	0/0	0/0	0/0	0/0
39805442	C	T	0/0	0/0	0/0	0/0	0/0	0/0	0/0	0/0	0/0	0/0	0/0	0/0	0/0	0/0	0/0	0/0	0/0	0/0	0/0	0/0
39805461	G	A	0/0	0/0	0/0	1/1	0/0	0/0	0/0	1/1	1/1	0/0	0/0	0/0	0/1	1/1	0/0	0/0	1/1	0/0	0/0	1/1
39805465	TACGCTA	T	0/0	0/0	0/0	0/0	0/0	0/0	0/0	0/0	0/0	0/0	0/0	0/0	0/0	0/0	0/0	0/0	0/0	0/0	0/0	0/0
39805476	C	A	0/0	0/0	0/0	1/1	0/0	0/0	0/0	1/1	1/1	0/0	0/0	0/0	0/1	1/1	0/0	0/0	1/1	0/0	0/0	1/1
39805494	G	A	0/0	0/0	0/0	0/0	1/1	0/0	0/0	0/0	0/0	0/0	0/0	0/0	0/0	0/0	0/0	1/1	0/0	0/0	0/0	0/0
39805502	G	A	0/0	0/0	0/0	1/1	0/0	0/0	0/0	1/1	1/1	0/0	0/0	0/0	0/1	1/1	0/0	0/0	1/1	0/0	0/0	1/1
39805553	G	C	0/0	0/0	0/0	1/1	0/0	0/0	0/0	1/1	1/1	0/0	0/0	0/0	0/1	1/1	0/0	0/0	1/1	0/0	0/0	1/1
39805582	C	T	0/0	0/0	0/0	0/0	0/0	0/0	0/0	0/0	0/0	0/0	0/0	0/0	0/0	0/0	0/0	0/0	0/0	0/0	0/0	0/0
39805583	C	T	0/0	0/0	0/0	0/0	0/0	0/0	0/0	0/0	0/0	0/0	0/0	0/0	0/0	0/0	0/0	0/0	0/0	0/0	0/0	0/0
39805628	C	T	0/1	0/0	1/1	0/0	1/1	0/0	0/0	0/0	0/0	0/0	0/0	0/0	0/1	0/0	0/0	1/1	0/0	1/1	0/0	0/0
39805643	G	A	0/0	0/0	0/0	0/0	0/0	0/0	0/0	0/0	0/0	0/0	0/0	0/0	0/0	0/0	0/0	0/0	0/0	0/0	0/0	0/0
39805646	T	C	0/1	0/0	1/1	1/1	1/1	0/0	0/0	1/1	1/1	0/0	0/0	0/0	1/1	1/1	0/0	1/1	1/1	1/1	0/1	1/1
39805665	C	G	0/0	0/0	0/0	0/0	0/0	0/0	0/0	0/0	0/0	0/0	0/0	0/0	0/0	0/0	0/0	0/0	0/0	0/0	0/0	0/0
39805686	T	C	0/1	0/0	1/1	1/1	1/1	0/0	0/0	1/1	1/1	0/0	0/0	0/0	1/1	1/1	0/0	1/1	1/1	1/1	0/1	1/1

39805701	C	A	0/0	0/0	0/0	0/0	0/0	0/0	0/0	0/0	0/0	0/0	0/0	0/0	0/0	0/0	0/0	0/0	0/0	0/0	0/0	0/0
39805730	G	A	0/0	0/0	0/0	0/0	0/0	0/0	0/0	0/0	0/0	0/0	0/0	0/0	0/0	0/0	0/0	0/0	0/0	0/0	0/0	0/0
39805732	C	T	0/0	0/0	0/0	0/0	0/0	0/0	0/0	0/0	0/0	0/0	0/0	0/0	0/0	0/0	0/0	0/0	0/0	0/0	0/0	0/0
39805733	G	A	0/1	0/0	1/1	0/0	0/0	0/0	0/0	0/0	0/0	0/0	0/0	0/0	0/1	0/0	0/0	0/0	0/0	1/1	0/0	0/0
39805739	C	T	0/1	0/0	1/1	0/0	1/1	0/0	0/0	0/0	0/0	0/0	0/0	0/0	0/1	0/0	0/0	1/1	0/0	1/1	0/0	0/0
39805744	T	C	0/1	0/0	1/1	1/1	1/1	0/0	0/0	1/1	1/1	0/0	0/0	0/0	1/1	1/1	0/0	1/1	1/1	1/1	0/0	1/1
39805762	C	T	0/1	0/0	1/1	0/0	1/1	0/0	0/0	0/0	0/0	0/0	0/0	0/0	0/1	0/0	0/0	1/1	0/0	1/1	0/0	0/0
39805771	T	C	0/0	0/0	0/0	0/0	1/1	0/0	0/0	0/0	0/0	0/0	0/0	0/0	0/0	0/0	0/0	1/1	0/0	0/0	0/0	0/0
39805782	G	C	0/0	0/0	0/0	0/0	0/0	0/0	0/0	0/0	0/0	0/0	0/0	0/0	0/0	0/0	0/0	0/0	0/0	0/0	0/0	0/0
39805819	CG	C	0/0	0/0	0/0	0/0	0/0	0/0	0/0	0/0	1/1	0/0	0/0	0/0	0/0	0/0	0/0	0/0	0/0	0/0	0/0	0/0
39805847	T	C	0/0	0/0	0/0	0/0	0/0	0/0	0/0	0/0	0/0	0/0	0/0	0/0	0/0	0/0	0/0	0/0	0/0	0/0	0/0	0/0
39805903	T	C	0/0	0/0	0/0	1/1	0/0	0/0	0/0	1/1	1/1	0/0	0/0	0/0	0/1	1/1	0/0	0/0	1/1	0/0	0/1	1/1
39805909	TA	TAA,T	0/1	0/0	1/1	1/1	0/0	0/0	0/0	1/1	1/1	0/0	0/0	0/0	1/1	1/1	0/0	0/0	1/1	1/1	0/1	1/1
39805930	T	C	0/1	0/0	1/1	1/1	1/1	0/0	0/0	1/1	1/1	0/0	0/0	0/0	1/1	1/1	0/0	1/1	1/1	1/1	0/1	1/1
39805956	T	C	1/1	1/1	1/1	1/1	1/1	1/1	1/1	1/1	1/1	0/0	1/1	0/0	1/1	1/1	1/1	1/1	1/1	1/1	1/1	1/1
39805959	CCA	C	0/0	0/0	0/0	0/0	0/0	0/0	0/0	0/0	0/0	0/0	0/0	0/0	0/0	0/0	0/0	0/0	0/0	0/0	0/0	0/0
39805967	C	T	0/1	0/0	1/1	0/0	0/0	0/0	0/0	0/0	0/0	0/0	0/0	0/0	0/1	0/0	0/0	0/0	0/0	1/1	0/0	0/0
39805984	A	G	0/1	0/0	1/1	0/0	1/1	0/0	0/0	0/0	0/0	0/0	0/0	0/0	0/1	0/0	0/0	1/1	0/0	1/1	0/0	0/0
39805991	T	C	0/1	0/0	1/1	1/1	1/1	0/0	0/0	1/1	1/1	0/0	0/0	0/0	1/1	1/1	0/0	1/1	1/1	1/1	0/1	1/1
39805992	C	T	0/0	0/0	0/0	0/0	0/0	0/0	0/0	0/0	0/0	0/0	0/0	0/0	0/0	0/0	0/0	0/0	0/0	0/0	0/0	0/0
39805998	C	T	0/0	0/0	0/0	0/0	0/0	0/0	0/0	0/0	0/0	0/0	0/0	0/0	0/0	0/0	0/0	0/0	0/0	0/0	0/0	0/0
39806017	C	T	0/0	0/0	0/0	0/0	0/0	0/0	0/0	0/0	0/0	0/0	0/0	0/0	0/0	0/0	0/0	0/0	0/0	0/0	0/0	0/0
39806023	T	C	0/1	0/0	1/1	1/1	1/1	0/0	0/0	1/1	1/1	0/0	0/0	0/0	1/1	1/1	0/0	1/1	1/1	1/1	0/1	1/1
39806032	G	A	0/0	0/0	0/0	0/0	0/0	0/0	0/0	0/0	0/0	0/0	0/0	0/0	0/0	0/0	0/0	0/0	0/0	0/0	0/0	0/0
39806036	G	A	0/0	0/0	0/0	0/0	0/0	0/0	0/0	0/0	0/0	0/0	0/0	0/0	0/0	0/0	0/0	0/0	0/0	0/0	0/0	0/0
39806040	A	G	0/0	0/0	0/0	0/0	0/0	0/0	0/0	0/0	0/0	0/0	0/0	0/0	0/0	0/0	0/0	0/0	0/0	0/0	0/0	0/0
39806049	G	A	0/0	0/0	0/0	0/0	0/0	0/0	0/0	0/0	0/0	0/0	0/0	0/0	0/0	0/0	0/0	0/0	0/0	0/0	0/0	0/0
39806054	A	T	0/0	0/0	0/0	0/0	0/0	0/0	0/0	0/0	0/0	0/0	0/0	0/0	0/0	0/0	0/0	0/0	0/0	0/0	0/0	0/0
39806060	A	C	0/0	0/0	0/0	0/0	0/0	0/0	0/0	0/0	0/0	0/0	0/0	0/0	0/0	0/0	0/0	0/0	0/0	0/0	0/0	0/0
39806065	G	A	0/0	0/0	0/0	0/0	0/0	0/0	0/0	0/0	0/0	0/0	0/0	0/0	0/0	0/0	0/0	0/0	0/0	0/0	0/0	0/0
39806069	C	CGGA	0/0	0/0	0/0	0/0	0/0	0/0	0/0	0/0	0/0	0/0	0/0	0/0	0/0	0/0	0/0	0/0	0/0	0/0	0/0	0/0
39806258	G	A	0/0	0/0	0/0	1/1	0/0	0/0	0/0	1/1	1/1	0/0	0/0	0/0	0/1	0/0	0/0	0/0	0/0	0/0	0/1	1/1
39806296	C	A	0/0	0/0	0/0	0/0	0/0	0/0	0/0	0/0	0/0	0/0	0/0	0/0	0/0	0/0	0/0	0/0	0/0	0/0	0/0	0/0
39806323	C	T	0/0	0/0	0/0	0/0	0/0	0/0	0/0	0/0	0/0	0/0	0/0	0/0	0/0	0/0	0/0	0/0	0/0	0/0	0/0	0/0
39806365	A	G	0/1	0/0	0/0	0/0	0/0	0/0	0/0	0/0	0/0	0/0	0/0	0/0	0/1	0/0	0/0	0/0	0/0	1/1	0/0	0/0

39806519	G	A	0/0	0/0	0/0	0/0	1/1	0/0	0/0	0/0	0/0	0/0	0/0	0/0	0/0	0/0	0/0	1/1	0/0	0/0	0/0	0/0
39806725	G	GTT,GTTT,GTTTT,GT,GTTTTT	2/3	4/4	2/2	1/1	4/4	2/2	3/5	2/3	2/2	0/0	0/0	1/4	2/2	1/1	2/3	4/4	1/1	2/2	2/3	1/2
39806726	T	TTC	./.	./.	./.	./.	./.	./.	1/1	./.	./.	0/0	0/0	./.	./.	./.	./.	./.	./.	./.	./.	./.
39806904	G	A	0/0	0/0	0/0	0/0	0/0	0/0	0/0	0/0	0/0	0/0	0/0	0/0	0/0	0/0	0/0	0/0	0/0	0/0	0/0	0/0
39806920	A	G	0/0	0/0	0/0	0/0	0/0	0/0	0/0	0/0	0/0	0/0	0/0	0/0	0/0	0/0	0/0	0/0	0/0	0/0	0/0	0/0
39806921	G	C	0/0	0/0	0/0	0/0	0/0	0/0	0/0	0/0	0/0	0/0	0/0	0/0	0/0	0/0	0/0	0/0	0/0	0/0	0/0	0/0
39806926	G	T	0/0	0/0	0/0	0/0	1/1	0/0	0/0	0/0	0/0	0/0	0/0	0/0	0/0	0/0	0/0	1/1	0/0	0/0	0/0	0/0
39807085	C	A	0/0	0/0	0/0	./.	1/1	0/0	0/0	0/0	0/0	0/0	0/0	0/0	0/0	0/0	0/0	1/1	0/0	./.	0/0	0/0
39807087	G	T	0/0	0/0	0/0	./.	0/0	0/0	0/0	./.	0/0	0/0	0/0	0/0	0/0	0/0	0/0	./.	0/0	./.	./.	0/0
39807090	C	CT	0/1	0/0	1/1	./.	1/1	./.	./.	0/0	./.	0/0	0/0	0/0	0/1	1/1	0/0	1/1	./.	1/1	./.	0/0
39807091	TA	T,TTAA,TTAAA,AA	0/1	0/0	./.	2/2	./.	1/1	1/1	2/2	2/2	0/0	0/0	0/0	0/2	./.	1/1	./.	2/2	./.	1/1	2/2
39807092	A	T,*	0/2	0/0	0/0	./.	0/0	2/2	2/2	./.	./.	0/0	0/0	0/0	0/0	0/0	2/2	0/0	./.	0/0	2/2	./.
39807099	A	AC,AAT	0/0	0/0	0/0	0/0	0/0	0/0	0/0	0/0	0/0	0/0	0/0	0/0	0/0	0/0	0/0	0/0	0/0	0/0	0/0	0/0
39807101	T	A	0/0	0/0	0/0	0/0	0/0	0/0	0/0	0/0	0/0	0/0	1/1	0/0	0/0	0/0	0/0	0/0	0/0	0/0	0/0	0/0
39807103	C	A	0/0	0/0	0/0	0/0	0/0	0/0	0/0	0/0	0/0	0/0	0/0	0/0	0/0	0/0	0/0	0/0	0/0	0/0	0/0	0/0
39807105	G	A	0/1	0/0	1/1	1/1	1/1	0/0	0/0	1/1	1/1	0/0	0/0	0/0	1/1	1/1	0/0	1/1	1/1	1/1	0/0	1/1
39807110	C	T	0/0	0/0	0/0	0/0	0/0	0/0	0/0	0/0	0/0	0/0	0/0	0/0	0/0	0/0	0/0	0/0	0/0	0/0	0/0	0/0
39807114	G	A	0/1	0/0	1/1	0/0	0/0	0/0	0/0	0/0	0/0	0/0	0/0	0/0	0/1	0/0	0/0	0/0	0/0	1/1	0/0	0/0
39807118	C	T	0/0	0/0	0/0	0/0	0/0	0/0	0/0	0/0	0/0	0/0	0/0	0/0	0/0	0/0	1/1	0/0	0/0	0/0	0/0	0/0
39807127	C	T	0/0	0/0	0/0	0/0	0/0	0/0	0/0	0/0	0/0	0/0	0/0	0/0	0/0	1/1	0/0	0/0	1/1	0/0	0/0	0/0
39807146	T	C	0/0	0/0	0/0	0/0	0/0	0/0	0/0	0/0	0/0	0/0	0/0	0/0	0/0	0/0	0/0	0/0	0/0	0/0	0/0	0/0

39807150	G	T	0/0	0/0	0/0	0/0	0/0	0/0	0/0	0/0	0/0	0/0	0/0	0/0	0/0	0/0	0/0	0/0	0/0	0/0	0/0	0/0
39807158	C	A	0/0	0/0	0/0	0/0	0/0	0/0	0/0	0/0	0/0	0/0	0/0	0/0	0/0	0/0	0/0	0/0	0/0	0/0	0/0	0/0
39807170	A	C,T	0/1	0/0	1/1	2/2	1/1	0/0	0/0	2/2	2/2	0/0	0/0	0/0	1/2	1/1	0/0	1/1	1/1	1/1	0/0	2/2
39807173	G	A	0/0	0/0	0/0	0/0	0/0	0/0	0/0	0/0	0/0	0/0	0/0	0/0	0/0	0/0	0/0	0/0	0/0	0/0	0/0	0/0
39807192	TA	TAA,T	0/1	0/0	1/1	2/2	0/0	0/0	0/0	2/2	2/2	0/0	0/0	0/0	1/2	1/1	0/0	0/0	1/1	1/1	0/0	2/2
39807201	C	A	0/0	0/0	0/0	0/0	0/0	0/0	0/0	0/0	0/0	0/0	0/0	0/0	0/0	0/0	0/0	./.	0/0	0/0	0/0	0/0
39807212	C	CTTTT,T	0/0	0/0	0/0	0/0	1/1	0/0	0/0	0/0	0/0	0/0	0/0	0/0	0/0	0/0	0/0	1/1	0/0	0/0	0/0	0/0
39807241	A	G	0/1	0/0	1/1	1/1	1/1	0/0	0/0	1/1	1/1	0/0	0/0	0/0	1/1	1/1	0/0	1/1	1/1	1/1	0/0	1/1
39807244	T	A	0/1	0/0	1/1	1/1	1/1	0/0	0/0	1/1	1/1	0/0	0/0	0/0	1/1	1/1	0/0	1/1	1/1	1/1	0/0	1/1
39807248	A	C	0/0	0/0	0/0	0/0	0/0	0/0	0/0	0/0	0/0	0/0	0/0	0/0	0/0	0/0	0/0	0/0	0/0	0/0	0/0	0/0
39807258	C	CTATTCAG<br>ATTTGTTG,<br>CTATTCAG<br>ATTCGTTG,<br>CTATTCAG<br>ATTCATTA	0/1	0/0	1/1	2/2	2/2	0/0	0/0	2/2	2/2	0/0	0/0	0/0	1/2	2/2	0/0	2/2	2/2	1/1	0/0	2/2
39807265	T	C	0/0	0/0	0/0	0/0	0/0	0/0	0/0	0/0	0/0	0/0	0/0	0/0	0/0	0/0	0/0	0/0	0/0	0/0	0/0	0/0
39807275	G	A	0/0	0/0	0/0	0/0	0/0	0/0	0/0	0/0	0/0	0/0	0/0	0/0	0/0	0/0	0/0	0/0	0/0	0/0	0/0	0/0
39807287	A	AT	0/0	0/0	0/0	0/0	0/0	0/0	0/0	0/0	0/0	0/0	0/0	0/0	0/0	0/0	0/0	0/0	0/0	0/0	0/0	0/0
39807295	T	A	0/0	0/0	0/0	0/0	1/1	0/0	0/0	0/0	0/0	0/0	0/0	0/0	0/0	0/0	0/0	1/1	0/0	0/0	0/0	0/0
39807307	G	A	0/0	0/0	0/0	0/0	0/0	0/0	0/0	0/0	0/0	0/0	0/0	0/0	0/0	0/0	0/0	0/0	0/0	0/0	0/0	0/0
39807413	C	A	0/0	0/0	0/0	0/0	1/1	0/0	0/0	0/0	0/0	0/0	0/0	0/0	0/0	0/0	0/0	1/1	0/0	0/0	0/0	0/0
39807444	G	A	0/1	0/0	1/1	1/1	1/1	0/0	0/0	1/1	1/1	0/0	0/0	0/0	1/1	1/1	0/0	1/1	1/1	1/1	0/0	1/1

1131	1141	1147	1166	1192	1214	1216	1217	1219	1222	1225	1226	1234	1237	1245	1252	1254	1272	1273	1275	1276	1279	1280	1286	1292	1302	1305
0/0	0/0	0/0	0/0	0/0	0/0	0/0	0/0	0/0	0/0	0/0	0/0	0/0	0/0	0/0	0/0	0/0	0/0	0/0	0/0	0/0	0/0	0/0	0/0	0/0	0/0	0/0
0/0	0/0	0/0	0/0	0/0	0/0	0/0	0/0	0/0	1/1	0/0	0/0	0/0	0/1	0/0	0/0	1/1	0/0	1/1	0/0	1/1	1/1	1/1	0/0	1/1	0/0	0/0
0/0	0/0	0/0	0/0	0/0	0/0	0/0	0/0	0/0	0/0	1/1	0/0	0/0	0/0	0/0	0/0	0/0	1/1	0/0	0/0	0/0	0/0	0/0	0/0	0/0	0/0	0/0
0/0	1/1	0/0	0/0	0/0	0/0	0/0	0/0	0/0	0/0	0/0	0/0	0/0	0/0	0/0	0/0	0/0	0/0	0/0	0/0	0/0	0/0	0/0	0/0	0/0	0/0	0/0
1/1	0/0	1/1	0/0	0/0	0/0	0/0	0/0	0/0	0/0	1/1	0/0	0/0	1/1	1/1	0/0	0/0	1/1	0/0	0/0	0/0	0/0	0/0	0/0	0/0	0/0	0/0
1/1	1/1	1/1	0/0	0/0	0/0	0/0	0/0	0/0	0/0	1/1	0/0	0/0	1/1	1/1	0/0	0/0	1/1	0/0	0/0	0/0	0/0	0/0	0/0	0/0	0/0	0/0
0/0	0/0	0/0	0/0	0/0	0/0	0/0	0/0	0/0	0/0	0/0	0/0	0/0	0/0	0/0	0/0	0/0	0/0	0/0	0/0	0/0	0/0	0/0	0/0	0/0	0/0	0/0
0/0	0/0	0/0	0/0	0/0	0/0	0/0	0/0	0/0	1/1	0/0	0/0	1/1	0/0	0/0	0/0	1/1	0/0	1/1	0/0	0/0	1/1	1/1	0/0	1/1	0/0	1/1
0/0	0/0	0/0	0/0	0/0	0/0	0/0	0/0	0/0	0/0	0/0	0/0	0/0	0/0	0/0	0/0	0/0	0/0	0/0	0/0	0/0	0/0	0/0	0/0	0/0	0/0	0/0
0/0	0/0	0/0	0/0	0/0	0/0	0/0	0/0	0/0	0/0	0/0	0/0	0/0	0/0	0/0	0/0	0/0	0/0	0/0	0/0	0/0	0/0	0/0	0/0	0/0	0/0	0/0
0/0	0/1	0/0	0/0	0/0	0/0	0/0	0/0	0/0	0/1	0/0	0/1	0/0	0/0	0/0	0/0	0/0	0/0	0/0	0/0	0/0	0/0	0/0	0/0	0/0	0/0	0/0
0/0	0/0	0/0	0/0	0/0	0/0	0/0	0/0	0/0	0/0	0/0	0/0	0/0	0/0	0/0	0/0	0/0	0/0	0/0	0/0	0/0	0/0	0/0	0/0	0/0	0/0	0/0
1/1	0/0	1/1	0/0	0/0	0/0	0/0	0/0	0/0	0/0	1/1	0/0	0/0	1/1	1/1	0/0	0/0	1/1	0/0	0/0	0/0	0/0	0/0	0/0	0/0	0/0	0/0
0/0	0/0	0/0	0/0	0/0	0/0	0/0	0/0	0/0	0/0	0/0	0/0	0/0	0/0	0/0	0/0	0/1	0/0	0/0	0/0	0/0	0/1	0/0	0/0	0/0	0/0	0/0
./.	0/0	0/0	0/0	./.	0/0	0/0	0/0	0/0	./.	0/0	0/0	1/1	./.	./.	./.	0/1	./.	1/1	0/0	./.	0/1	1/1	0/0	1/1	./.	1/1
./.	0/0	0/0	0/0	./.	0/0	0/0	0/0	0/0	0/0	0/0	0/0	0/0	./.	./.	./.	0/0	./.	0/0	0/0	0/0	0/0	0/0	0/0	0/0	./.	0/0

./.	1/1	2/2	0/0	1/1	3/3	4/4	0/1	0/1	./.	0/0	3/3	0/0	3/3	2/3	4/4	3/5	./.	3/3	0/1	2/3	3/5	./.	0/1	6/6	4/4	0/1
0/0	0/0	0/0	0/0	0/0	0/0	0/0	0/0	0/0	0/0	0/0	0/0	0/0	0/0	0/0	0/0	0/0	0/0	0/0	0/0	0/0	0/0	0/0	0/0	0/0	0/0	0/0
./.	./.	0/0	0/0	0/0	0/0	0/0	0/0	0/0	0/0	0/0	0/0	0/0	0/0	0/0	0/0	0/0	0/0	0/0	0/0	0/0	0/0	0/0	0/0	0/0	0/0	0/0
1/1	0/0	1/1	0/0	0/0	0/0	0/0	0/0	0/0	0/0	1/1	0/0	0/0	1/1	1/1	0/0	0/0	1/1	0/0	0/0	0/0	0/0	0/0	0/0	0/0	0/0	0/0
0/0	0/0	0/0	0/0	0/0	0/0	0/0	0/0	0/0	1/1	0/0	0/0	1/1	0/0	0/0	0/0	1/1	0/0	1/1	0/0	0/0	1/1	1/1	0/0	1/1	0/0	1/1
0/0	1/1	0/0	0/0	0/0	0/0	0/0	0/0	0/0	0/0	0/0	0/0	0/0	0/0	0/0	0/0	0/0	0/0	0/0	0/0	0/0	0/0	0/0	0/0	0/0	0/0	0/0
0/0	0/0	0/0	0/0	0/0	0/0	0/0	0/0	0/0	1/1	0/0	0/0	1/1	0/0	0/0	0/0	1/1	0/0	1/1	0/0	0/0	1/1	1/1	0/0	1/1	0/0	1/1
1/1	0/0	1/1	0/0	0/0	0/0	0/0	0/0	0/0	0/0	1/1	0/0	0/0	0/1	1/1	0/0	0/0	1/1	0/0	0/0	0/0	0/0	0/0	0/0	0/0	0/0	0/0
0/0	1/1	0/0	0/0	0/0	1/1	0/0	0/0	0/0	0/0	0/0	1/1	0/0	0/0	0/0	0/0	0/0	0/0	0/0	0/0	1/1	0/0	./.	1/1	0/0	0/0	0/0
1/1	2/2	1/1	0/0	0/0	2/2	0/0	0/0	0/0	0/0	1/1	2/2	1/1	0/1	1/1	0/0	0/0	1/1	0/0	0/0	2/2	0/0	./.	2/2	0/0	0/0	1/1
0/0	1/1	0/0	0/0	0/0	./.	0/0	0/0	0/0	0/0	0/0	./.	0/0	0/0	0/0	0/0	0/0	0/0	0/0	0/0	./.	0/0	./.	./.	0/0	0/0	0/0
1/1	0/0	1/1	0/0	0/0	0/0	0/0	0/0	0/0	0/0	1/1	0/0	0/0	0/1	1/1	0/0	0/0	1/1	0/0	0/0	0/0	0/0	./.	0/0	0/0	0/0	0/0
1/1	1/1	1/1	0/0	0/0	0/0	0/0	0/0	0/0	1/1	1/1	0/0	1/1	1/1	1/1	0/0	1/1	1/1	1/1	0/0	0/0	1/1	1/1	0/0	1/1	0/0	1/1
0/0	1/1	0/0	0/0	0/0	0/0	0/0	0/0	0/0	0/0	0/0	0/0	0/0	0/0	0/0	0/0	0/0	0/0	0/0	0/0	0/0	0/0	0/0	0/0	0/0	0/0	0/0
0/0	0/0	0/0	0/0	0/0	0/0	0/0	0/0	0/0	0/0	0/0	0/0	0/0	0/0	0/0	0/0	0/0	0/0	0/0	0/0	0/0	0/0	0/0	0/0	0/0	0/0	0/0
1/1	1/1	1/1	0/0	0/0	0/0	0/0	0/0	0/0	1/1	1/1	0/0	1/1	1/1	1/1	0/0	1/1	1/1	1/1	0/0	0/0	1/1	1/1	0/0	1/1	0/0	1/1
1/1	0/0	1/1	0/0	0/0	0/0	0/0	0/0	0/0	0/0	1/1	0/0	0/0	1/1	1/1	0/0	0/0	1/1	0/0	0/0	0/0	0/0	0/0	0/0	0/0	0/0	0/0
0/0	1/1	0/0	0/0	0/0	0/0	0/0	0/0	0/0	0/0	0/0	0/0	0/0	0/0	0/0	0/0	0/0	0/0	0/0	0/0	0/0	0/0	0/0	0/0	0/0	0/0	0/0
0/0	0/0	0/0	0/0	0/0	0/0	0/0	0/0	0/0	1/1	0/0	0/0	0/1	0/1	0/0	0/0	1/1	0/0	1/1	0/0	0/0	1/1	1/1	0/0	1/1	0/0	1/1
0/0	0/0	0/0	0/0	0/0	0/0	0/0	0/0	0/0	1/1	0/0	0/0	0/0	0/1	0/0	0/0	1/1	0/0	1/1	0/0	0/0	1/1	1/1	0/0	1/1	0/0	0/0
0/0	1/1	0/0	0/0	0/0	0/0	0/0	0/0	0/0	0/0	0/0	0/0	0/0	0/0	0/0	0/0	0/0	0/0	0/0	0/0	0/0	0/0	0/0	0/0	0/0	0/0	0/0
0/0	0/0	0/0	0/0	0/0	0/0	0/0	0/0	0/0	1/1	0/0	0/0	1/1	0/1	0/0	0/0	1/1	0/0	1/1	0/0	0/0	1/1	1/1	0/0	1/1	0/0	1/1
0/0	1/1	0/0	0/0	0/0	0/0	0/0	0/0	0/0	0/0	0/0	0/0	0/0	0/0	0/0	0/0	0/0	0/0	0/0	0/0	0/0	0/0	0/0	0/0	0/0	0/0	0/0
1/1	1/1	1/1	0/0	0/0	0/0	0/0	0/0	0/0	1/1	1/1	0/0	1/1	1/1	1/1	0/0	1/1	1/1	1/1	0/0	0/0	1/1	1/1	0/0	1/1	0/0	1/1
1/1	0/0	1/1	0/0	0/0	0/0	0/0	0/0	0/0	0/0	1/1	0/0	0/0	1/1	1/1	0/0	0/0	1/1	0/0	0/0	0/0	0/0	0/0	0/0	0/0	0/0	0/0
0/0	2/2	0/0	0/0	0/0	0/0	0/0	0/0	0/0	1/2	0/0	0/0	0/0	0/0	0/1	0/0	1/1	0/0	1/1	0/0	0/0	1/1	1/1	0/0	1/1	0/0	0/0

1/1	1/1	1/1	0/0	0/0	1/1	1/1	0/0	0/0	1/1	1/1	1/1	1/1	1/1	1/1	1/1	1/1	1/1	1/1	0/0	1/1	1/1	1/1	1/1	1/1	1/1	1/1
1/1	1/1	1/1	0/0	0/0	0/0	0/0	0/0	0/0	0/0	1/1	0/0	0/0	0/1	1/1	0/0	0/0	1/1	0/0	0/0	0/0	0/0	0/0	0/0	0/0	0/0	0/0
1/1	0/0	1/1	0/0	0/0	0/0	0/0	0/0	0/0	0/0	1/1	0/0	0/0	0/1	1/1	0/0	0/0	1/1	0/0	0/0	0/0	0/0	0/0	0/0	0/0	0/0	0/0
0/0	0/0	0/0	0/0	0/0	0/0	0/0	0/0	0/0	0/0	0/0	0/0	0/0	0/0	0/0	0/0	0/0	0/0	0/0	0/0	0/0	0/0	0/0	0/0	0/0	0/0	0/0
1/1	1/1	1/1	0/0	0/0	0/0	0/0	0/0	0/0	1/1	1/1	0/0	1/1	1/1	1/1	0/0	1/1	1/1	1/1	0/0	0/0	1/1	1/1	0/0	0/1	0/0	1/1
1/1	1/1	1/1	0/0	0/0	0/0	0/0	0/0	0/0	1/1	1/1	0/0	1/1	1/1	1/1	0/0	1/1	1/1	1/1	0/0	0/0	1/1	1/1	0/0	1/1	0/0	1/1
1/1	1/1	1/1	0/0	0/0	0/0	0/0	0/0	0/0	1/1	1/1	0/0	1/1	1/1	1/1	0/0	1/1	1/1	1/1	0/0	0/0	1/1	1/1	0/0	1/1	0/0	1/1
0/0	0/0	./.	0/0	0/0	0/0	0/0	0/0	0/0	0/0	0/0	0/0	0/0	0/0	0/0	0/0	0/0	./.	0/0	0/0	0/0	0/0	0/0	0/0	0/0	0/0	0/0
1/1	1/1	1/1	0/0	0/0	0/0	0/0	0/0	0/0	0/0	1/1	0/0	0/0	0/1	1/1	0/0	0/0	1/1	0/0	0/0	0/0	0/0	0/0	0/0	0/0	0/0	0/0
0/0	0/0	0/0	0/0	0/0	0/0	0/0	0/0	0/0	0/0	0/0	0/0	0/0	0/0	0/0	0/0	0/0	0/0	0/0	0/0	0/0	0/0	0/0	0/0	0/0	0/0	0/0
0/0	0/0	0/0	0/0	0/0	0/0	0/0	0/0	0/0	1/1	0/0	0/0	1/1	0/1	0/0	0/0	1/1	0/0	1/1	0/0	0/0	1/1	1/1	0/0	0/1	0/0	1/1
0/0	0/0	0/0	0/0	0/0	0/0	0/0	0/0	0/0	0/0	0/0	0/0	0/0	0/0	0/0	0/0	0/0	0/0	0/0	0/0	0/0	0/0	0/0	0/0	0/0	0/0	0/0
0/0	0/0	0/0	0/0	0/0	0/0	0/0	0/0	0/0	0/0	0/0	0/0	0/0	0/0	0/0	0/0	0/0	0/0	0/0	0/0	0/0	0/0	0/0	0/0	0/0	0/0	0/0
0/0	0/0	0/0	0/0	0/0	0/0	0/0	0/0	0/0	0/0	0/0	0/0	1/1	0/0	0/0	0/0	0/0	0/0	0/0	0/0	0/0	0/0	0/0	0/0	0/0	0/0	1/1
1/1	1/1	1/1	0/0	0/0	0/0	0/0	0/0	0/0	1/1	1/1	0/0	1/1	1/1	1/1	0/0	1/1	1/1	1/1	0/0	0/0	1/1	1/1	0/0	0/1	0/0	1/1
0/0	0/0	0/0	0/0	0/0	0/0	0/0	0/0	0/0	0/0	0/0	0/0	0/0	0/0	0/0	0/0	0/0	0/0	0/0	0/0	0/0	0/0	0/0	0/0	0/0	0/0	0/0
0/0	0/0	0/0	0/0	0/0	0/0	0/0	0/0	0/0	0/0	0/0	0/0	0/0	0/0	0/0	0/0	0/0	0/0	0/0	0/0	0/0	0/0	0/0	0/0	0/0	0/0	0/0
0/0	0/0	0/0	0/0	0/0	0/0	0/0	0/0	0/0	0/0	0/0	0/0	0/0	0/1	0/0	0/0	0/0	0/0	0/0	0/0	0/0	0/0	0/0	0/0	0/0	0/0	0/0
0/0	0/0	0/0	0/0	0/0	0/0	0/0	0/0	0/0	1/1	0/0	0/0	1/1	0/1	0/0	0/0	1/1	0/0	1/1	0/0	0/0	1/1	1/1	0/0	0/1	0/0	1/1
0/0	0/0	0/0	0/0	0/0	0/0	0/0	0/0	0/0	0/0	0/0	0/0	0/0	0/0	0/0	0/0	0/0	0/0	0/0	0/0	0/0	0/0	0/0	0/0	0/0	0/0	0/0
1/1	1/1	1/1	0/0	0/0	0/0	0/0	0/0	0/0	1/1	1/1	0/0	1/1	1/1	1/1	0/0	1/1	1/1	1/1	0/0	0/0	1/1	1/1	0/0	0/1	0/0	1/1
0/0	0/0	0/0	0/0	0/0	0/0	0/0	0/0	0/0	1/1	0/0	0/0	1/1	0/1	0/0	0/0	1/1	0/0	1/1	0/0	0/0	1/1	1/1	0/0	0/1	0/0	1/1
0/0	0/0	0/0	0/0	0/0	0/0	0/0	0/0	0/0	1/1	0/0	0/0	1/1	0/0	0/0	0/0	1/1	0/0	1/1	0/0	0/0	1/1	1/1	0/0	0/1	0/0	1/1
1/1	1/1	1/1	0/0	0/0	0/0	0/0	0/0	0/0	0/0	1/1	0/0	0/0	1/1	1/1	0/0	0/0	1/1	0/0	0/0	0/0	0/0	0/0	0/0	0/0	0/0	0/0
1/1	1/1	1/1	0/0	0/0	0/0	0/0	0/0	0/0	1/1	1/1	0/0	1/1	1/1	1/1	0/0	1/1	1/1	1/1	0/0	0/0	1/1	1/1	0/0	0/1	0/0	1/1
1/1	0/0	1/1	0/0	0/0	0/0	0/0	0/0	0/0	0/0	1/1	0/0	0/0	1/1	1/1	0/0	0/0	1/1	0/0	0/0	0/0	0/0	0/0	0/0	0/0	0/0	0/0
0/0	0/0	0/0	0/0	0/0	0/0	0/0	0/0	0/0	0/0	0/0	0/0	0/0	0/0	0/0	0/0	0/0	0/0	0/0	0/0	0/0	0/0	0/0	0/0	0/0	0/0	0/0
0/0	1/1	0/0	0/0	0/0	0/0	0/0	0/0	0/0	0/0	0/0	0/0	0/0	0/0	0/0	0/0	0/0	0/0	0/0	0/0	0/0	0/0	0/0	0/0	0/0	0/0	0/0
1/1	1/1	1/1	0/0	0/0	1/1	1/1	0/0	0/0	1/1	1/1	1/1	1/1	1/1	1/1	1/1	1/1	1/1	1/1	0/0	1/1	1/1	1/1	1/1	1/1	1/1	1/1
0/0	0/0	0/0	0/0	1/1	0/0	0/0	1/1	1/1	0/0	0/0	0/0	0/0	0/0	0/0	0/0	0/0	0/0	0/0	1/1	0/0	0/0	0/0	0/0	0/0	0/0	0/0
1/1	1/1	1/1	0/0	0/0	0/0	0/0	0/0	0/0	1/1	1/1	0/0	1/1	1/1	1/1	0/0	1/1	1/1	1/1	0/0	0/0	1/1	1/1	0/0	0/1	0/0	1/1
1/1	0/0	1/1	0/0	0/0	0/0	0/0	0/0	0/0	0/0	1/1	0/0	0/0	1/1	1/1	0/0	0/0	1/1	0/0	0/0	0/0	0/0	0/0	0/0	0/0	0/0	0/0
1/1	1/1	1/1	0/0	0/0	0/0	0/0	0/0	0/0	1/1	1/1	0/0	1/1	1/1	1/1	0/0	1/1	1/1	1/1	0/0	0/0	1/1	1/1	0/0	0/1	0/0	1/1
0/0	0/0	0/0	0/0	0/0	0/0	0/0	0/0	0/0	0/0	0/0	0/0	0/0	0/0	0/0	0/0	0/0	0/0	0/0	0/0	0/0	0/0	0/0	0/0	0/0	0/0	0/0
1/1	1/1	1/1	0/0	0/0	0/0	0/0	0/0	0/0	0/0	1/1	0/0	0/0	1/1	1/1	0/0	0/0	1/1	0/0	0/0	0/0	0/0	0/0	0/0	0/0	0/0	0/0

0/0	0/0	0/0	0/0	0/0	0/0	0/0	0/0	0/0	1/1	0/0	0/0	1/1	0/0	0/0	0/0	1/1	0/0	1/1	0/0	0/0	1/1	1/1	0/0	0/1	0/0	1/1
0/0	0/0	0/0	0/0	0/0	0/0	0/0	0/0	0/0	0/0	0/0	0/0	0/0	0/0	0/0	0/0	0/0	0/0	0/0	0/0	0/0	0/0	0/0	0/0	0/0	0/0	0/0
0/0	0/0	0/0	0/0	0/0	0/0	0/0	0/0	0/0	1/1	0/0	0/0	0/0	0/0	0/0	0/0	1/1	0/0	1/1	0/0	0/0	1/1	1/1	0/0	0/1	0/0	0/0
1/1	1/1	1/1	0/0	0/0	0/0	0/0	0/0	0/0	1/1	1/1	0/0	1/1	1/1	1/1	0/0	1/1	1/1	1/1	0/0	0/0	1/1	1/1	0/0	0/1	0/0	1/1
0/0	0/0	0/0	0/0	0/0	0/0	0/0	0/0	0/0	0/0	0/0	0/0	0/0	0/0	0/0	0/0	0/0	0/0	0/0	0/0	0/0	0/0	0/0	0/0	0/0	0/0	0/0
0/0	0/0	0/0	0/0	0/0	0/0	0/0	0/0	0/0	1/1	0/0	0/0	1/1	0/0	0/0	0/0	1/1	0/0	1/1	0/0	0/0	1/1	1/1	0/0	1/1	0/0	1/1
1/1	1/1	1/1	0/0	0/0	0/0	0/0	0/0	0/0	1/1	1/1	0/0	1/1	1/1	1/1	0/0	1/1	1/1	1/1	0/0	0/0	1/1	1/1	0/0	1/1	0/0	1/1
1/1	1/1	1/1	0/0	0/0	0/0	0/0	0/0	0/0	0/0	1/1	0/0	0/0	1/1	1/1	0/0	0/0	1/1	./.	0/0	0/0	0/0	0/0	0/0	0/0	0/0	0/0
0/0	0/0	0/0	0/0	0/0	0/0	0/0	0/0	0/0	1/1	0/0	0/0	1/1	0/0	0/0	0/0	1/1	0/0	1/1	0/0	0/0	1/1	./.	0/0	1/1	0/0	1/1
0/0	1/1	0/0	0/0	0/0	0/0	0/0	0/0	0/0	0/0	0/0	0/0	0/0	0/0	0/0	0/0	0/0	0/0	0/0	0/0	0/0	0/0	0/0	0/0	0/0	0/0	0/0
0/0	0/0	0/0	0/0	0/0	0/0	0/0	0/0	0/0	1/1	0/0	0/0	1/1	0/0	0/0	0/0	1/1	0/0	1/1	0/0	0/0	1/1	1/1	0/0	1/1	0/0	1/1
0/0	0/0	0/0	0/0	0/0	0/0	0/0	0/0	0/0	0/0	0/0	0/0	0/0	0/0	0/0	0/0	0/0	0/0	0/0	0/0	0/0	0/0	0/0	0/0	0/0	0/0	0/0
0/0	0/0	0/0	0/0	0/0	0/0	0/0	0/0	0/0	0/0	0/0	0/0	0/0	0/0	0/0	0/0	0/0	0/0	0/0	0/0	0/0	0/0	0/0	0/0	0/0	0/0	0/0
0/0	0/0	0/0	0/0	0/0	0/0	0/0	0/0	0/0	0/0	0/0	0/0	0/0	0/0	0/0	0/0	0/0	0/0	0/0	0/0	0/0	0/0	0/0	0/0	0/0	0/0	0/0
1/1	1/1	1/1	0/0	0/0	1/1	1/1	0/1	0/0	1/1	1/1	1/1	1/1	1/1	1/1	1/1	1/1	1/1	1/1	0/0	1/1	1/1	1/1	1/1	1/1	1/1	1/1
1/1	1/1	1/1	0/0	0/0	1/1	1/1	0/1	0/0	1/1	1/1	1/1	1/1	1/1	1/1	1/1	1/1	1/1	1/1	0/0	1/1	1/1	1/1	1/1	1/1	1/1	1/1
1/1	1/1	1/1	0/0	0/0	0/0	0/0	0/1	0/0	1/1	1/1	0/0	1/1	1/1	1/1	0/0	1/1	1/1	1/1	0/0	0/0	1/1	1/1	0/0	1/1	0/0	1/1
./.	./.	./.	0/0	0/0	./.	./.	0/0	0/0	1/1	./.	./.	./.	./.	./.	./.	./.	0/0	./.	0/0	./.	./.	./.	./.	0/1	0/0	./.
3/3	1/1	3/3	0/2	0/0	1/1	1/1	1/2	0/0	4/4	3/3	1/1	1/1	3/3	3/3	1/1	1/1	3/3	1/1	0/2	1/1	1/1	1/1	1/1	0/1	1/1	1/1
0/0	0/0	1/1	0/0	0/0	0/0	0/0	0/0	0/0	0/0	0/0	0/0	0/0	0/0	1/1	0/0	0/0	0/0	0/0	0/0	0/0	0/0	0/0	0/0	0/0	0/0	0/0
0/0	0/0	0/0	0/0	0/0	0/0	0/0	0/0	0/0	0/0	0/0	0/0	0/0	0/0	0/0	0/0	0/0	0/0	0/0	0/0	0/0	0/0	0/0	0/0	0/0	0/0	0/0
0/0	0/0	0/0	0/0	0/0	0/0	0/0	0/0	0/0	0/0	0/0	0/0	0/0	0/0	0/0	0/0	0/0	0/0	0/0	0/0	0/0	0/0	0/0	0/0	0/0	0/0	0/0
0/0	0/0	0/0	0/0	0/0	0/0	0/0	0/0	0/0	0/0	0/0	0/0	0/0	0/0	0/0	0/0	0/0	0/0	0/0	0/0	0/0	0/0	0/0	0/0	0/0	0/0	0/0
0/0	0/0	0/0	0/0	0/0	0/0	0/0	0/0	0/0	0/0	0/0	0/0	0/0	0/0	0/0	0/0	0/0	0/0	0/0	0/0	0/0	0/0	0/0	0/0	0/0	0/0	0/0
1/1	0/0	1/1	0/0	0/0	0/0	0/0	0/0	0/0	0/0	1/1	0/0	0/0	0/1	1/1	0/0	0/0	1/1	0/0	0/0	0/0	0/0	0/0	0/0	0/0	0/0	0/0

0/0	0/0	0/0	0/0	0/0	0/0	0/0	0/0	0/0	0/0	0/0	0/0	0/0	0/0	0/0	0/0	0/0	0/0	0/0	0/0	0/0	0/0	0/0	0/0	0/0	0/0	0/0
1/1	1/1	1/1	0/0	0/0	0/0	0/0	0/0	0/0	0/0	1/1	0/0	0/0	0/1	1/1	0/0	0/0	1/1	0/0	0/0	0/0	0/0	0/0	0/0	0/0	0/0	0/0
1/1	1/1	1/1	0/0	0/0	0/0	0/0	0/0	0/0	0/0	1/1	0/0	0/0	0/1	1/1	0/0	0/0	1/1	0/0	0/0	0/0	0/0	0/0	0/0	0/0	0/0	0/0
1/1	1/1	1/1	0/0	0/0	1/1	1/1	0/0	0/0	1/1	1/1	1/1	1/1	1/1	1/1	1/1	1/1	1/1	1/1	0/0	1/1	1/1	./.	1/1	1/1	1/1	1/1
1/1	1/1	1/1	0/0	0/0	0/0	0/0	0/0	0/0	1/1	1/1	0/0	1/1	1/1	1/1	0/0	1/1	1/1	1/1	0/0	0/0	1/1	1/1	0/0	1/1	0/0	1/1
0/0	0/0	0/0	0/0	0/0	0/0	0/0	0/0	0/0	0/0	0/0	0/0	0/0	0/0	0/0	0/0	0/0	0/0	0/0	0/0	0/0	0/0	0/0	0/0	0/0	0/0	0/0
1/1	1/1	1/1	0/0	0/0	0/0	0/0	0/1	0/0	1/1	1/1	0/0	1/1	1/1	1/1	0/0	1/1	1/1	1/1	0/0	0/0	1/1	1/1	0/0	1/1	0/0	1/1
1/1	1/1	1/1	0/0	0/0	0/0	0/0	0/0	0/0	0/0	1/1	0/0	0/0	1/1	1/1	0/0	0/0	1/1	0/0	0/0	0/0	0/0	0/0	0/0	0/0	0/0	0/0
0/0	0/0	0/0	0/0	0/0	0/0	0/0	0/0	0/0	0/0	0/0	0/0	0/0	0/0	0/0	0/0	0/0	0/0	0/0	0/0	0/0	0/0	0/0	0/0	0/0	0/0	0/0
0/0	1/1	0/0	0/0	0/0	0/0	0/0	0/0	0/0	0/0	0/0	0/0	0/0	0/0	0/0	0/0	0/0	0/0	0/0	0/0	0/0	0/0	0/0	0/0	0/0	0/0	0/0
1/1	1/1	1/1	0/0	0/0	0/0	0/0	0/0	0/0	0/0	1/1	0/0	0/0	1/1	1/1	0/0	0/0	1/1	0/0	0/0	0/0	0/0	0/0	0/0	0/0	0/0	0/0
0/0	0/0	0/0	0/0	0/0	0/0	0/0	0/0	0/0	0/0	0/0	0/0	0/0	0/0	0/0	0/0	0/0	0/0	0/0	0/0	0/0	0/0	0/0	0/0	0/0	0/0	0/0
0/0	0/0	0/0	0/0	0/0	0/0	0/0	0/0	0/0	1/1	0/0	0/0	1/1	0/0	0/0	0/0	1/1	0/0	1/1	0/0	0/0	1/1	1/1	0/0	0/1	0/0	1/1
1/1	1/1	1/1	0/0	0/0	0/0	0/0	0/0	0/0	0/0	1/1	0/0	0/0	1/1	1/1	0/0	0/0	1/1	0/0	0/0	0/0	0/0	0/0	0/0	0/0	0/0	0/0
0/0	0/0	0/0	0/0	0/0	0/0	0/0	0/0	0/0	0/0	0/0	0/0	0/0	0/0	0/0	0/0	0/0	0/0	0/0	0/0	0/0	0/0	0/0	0/0	0/0	0/0	0/0
1/1	1/1	1/1	0/0	0/0	0/0	0/0	0/0	0/0	1/1	1/1	0/0	1/1	1/1	1/1	0/0	1/1	1/1	1/1	0/0	0/0	1/1	1/1	0/0	0/1	0/0	1/1
0/0	0/0	0/0	0/0	0/0	0/0	0/0	0/0	0/0	1/1	0/0	0/0	1/1	0/0	0/0	0/0	1/1	0/0	1/1	0/0	0/0	1/1	1/1	0/0	0/1	0/0	1/1
0/0	0/0	0/0	0/0	0/0	0/0	0/0	0/0	0/0	0/0	0/0	0/0	1/1	0/0	0/0	0/0	0/0	0/0	0/0	0/0	0/0	0/0	0/0	0/0	0/0	0/0	1/1
0/0	1/1	0/0	0/0	0/0	0/0	0/0	0/0	0/0	0/0	0/0	0/0	0/0	0/0	0/0	0/0	0/0	0/0	0/0	0/0	0/0	0/0	0/0	0/0	0/0	0/0	0/0
1/1	1/1	1/1	0/0	0/0	0/0	0/0	0/0	0/0	0/0	1/1	0/0	0/0	1/1	1/1	0/0	0/0	1/1	0/0	0/0	0/0	0/0	0/0	0/0	0/0	0/0	0/0
1/1	1/1	1/1	0/0	0/0	0/0	0/0	0/0	0/0	0/0	1/1	0/0	0/0	1/1	1/1	0/0	0/0	1/1	0/0	0/0	0/0	0/0	0/0	0/0	0/0	0/0	0/0
0/0	0/0	0/0	0/0	0/0	0/0	0/0	0/0	0/0	0/0	0/0	0/0	0/0	0/0	0/0	0/0	0/0	0/0	0/0	0/0	0/0	0/0	0/0	0/0	0/0	0/0	0/0
1/1	1/1	1/1	0/0	0/0	0/0	0/0	0/0	0/0	1/1	1/1	0/0	1/1	1/1	1/1	0/0	1/1	1/1	1/1	0/0	0/0	1/1	1/1	0/0	0/1	0/0	1/1
0/0	1/1	0/0	0/0	0/0	0/0	0/0	0/0	0/0	0/0	2/2	0/0	0/0	0/0	0/0	0/0	0/0	2/2	0/0	0/0	0/0	0/0	0/0	0/0	0/0	0/0	0/0
0/0	0/0	0/0	0/0	0/0	0/0	0/0	0/0	0/0	0/0	0/0	0/0	0/0	0/0	0/0	0/0	0/0	0/0	0/0	0/0	0/0	0/0	0/0	0/0	0/0	0/0	0/0
1/1	1/1	1/1	0/0	0/0	0/0	0/0	0/0	0/0	0/0	1/1	0/0	0/0	1/1	1/1	0/0	0/0	1/1	0/0	0/0	0/0	0/0	0/0	0/0	0/0	0/0	0/0
0/0	0/0	0/0	0/0	0/0	0/0	0/0	0/0	0/0	0/0	0/0	0/0	0/0	0/0	0/0	0/0	0/0	0/0	0/0	0/0	0/0	0/0	0/0	0/0	0/0	0/0	0/0
0/0	0/0	0/0	0/0	0/0	0/0	0/0	0/0	0/0	1/1	0/0	0/0	1/1	0/1	0/0	0/0	1/1	0/0	1/1	0/0	0/0	1/1	1/1	0/0	0/1	0/0	1/1
1/1	1/1	1/1	0/0	0/0	0/0	0/0	0/0	0/0	0/0	1/1	0/0	0/0	0/1	1/1	0/0	0/0	1/1	0/0	0/0	0/0	0/0	0/0	0/0	0/0	0/0	0/0
0/0	1/1	0/0	0/0	0/0	0/0	0/0	0/0	0/0	0/0	0/0	0/0	0/0	0/0	0/0	0/0	0/0	0/0	0/0	0/0	0/0	0/0	0/0	0/0	0/0	0/0	0/0
1/1	1/1	1/1	0/0	0/0	0/0	0/0	0/0	0/0	1/1	1/1	0/0	1/1	1/1	1/1	0/0	1/1	1/1	1/1	0/0	0/0	1/1	1/1	0/0	0/1	0/0	1/1
0/0	0/0	0/0	0/0	0/0	0/0	0/0	0/0	0/0	0/0	0/0	0/0	0/0	0/0	0/0	0/0	0/0	0/0	0/0	0/0	0/0	0/0	0/0	0/0	0/0	0/0	0/0
0/0	0/0	0/0	0/0	0/0	1/1	1/1	0/0	0/0	0/0	0/0	1/1	0/0	0/0	0/0	1/1	0/0	0/0	0/0	0/0	1/1	0/0	0/0	1/1	0/1	1/1	0/0

1/1	1/1	1/1	0/0	0/0	0/0	0/0	0/0	0/0	1/1	1/1	0/0	1/1	1/1	1/1	0/0	1/1	1/1	1/1	0/0	0/0	1/1	1/1	0/0	0/1	0/0	1/1
0/0	0/0	0/0	0/0	0/0	0/0	0/0	0/0	0/0	0/0	0/0	0/0	0/0	0/0	0/0	0/0	0/0	0/0	0/0	0/0	0/0	0/0	0/0	0/0	0/0	0/0	0/0
1/1	1/1	1/1	0/0	0/0	0/0	0/0	0/0	0/0	1/1	1/1	0/0	1/1	1/1	1/1	0/0	1/1	1/1	1/1	0/0	0/0	1/1	1/1	0/0	1/1	0/0	1/1
0/0	0/0	0/0	0/0	0/0	0/0	0/0	0/0	0/0	0/0	0/0	0/0	0/0	0/0	0/0	0/0	0/0	0/0	0/0	0/0	0/0	0/0	0/0	0/0	0/0	0/0	0/0
0/0	0/0	0/0	0/0	0/0	0/0	0/0	0/0	0/0	1/1	0/0	0/0	0/0	0/0	0/0	0/0	1/1	0/0	1/1	0/0	0/0	1/1	1/1	0/0	1/1	0/0	0/0
0/0	0/0	0/0	0/0	0/0	0/0	0/0	0/0	0/0	0/0	0/0	0/0	0/0	0/0	0/0	0/0	0/0	0/0	0/0	0/0	0/0	0/0	0/0	0/0	0/0	0/0	0/0
0/0	0/0	0/0	0/0	0/0	0/0	0/0	0/0	0/0	0/0	0/0	0/0	0/0	0/0	0/0	0/0	0/0	0/0	0/0	0/0	0/0	0/0	0/0	0/0	0/0	0/0	0/0
0/0	0/0	0/0	0/0	0/0	0/0	0/0	0/0	0/0	0/0	0/0	0/0	1/1	0/0	0/0	0/0	0/0	0/0	0/0	0/0	0/0	0/0	0/0	0/0	0/0	0/0	1/1
0/0	0/0	0/0	0/0	0/0	0/0	0/0	0/0	0/0	0/0	0/0	0/0	0/0	0/0	0/0	0/0	0/0	0/0	0/0	0/0	0/0	0/0	0/0	0/0	0/0	0/0	0/0
0/0	0/0	0/0	0/0	0/0	0/0	0/0	0/0	0/0	0/0	0/0	0/0	0/0	0/0	0/0	0/0	0/0	0/0	0/0	0/0	0/0	0/0	0/0	0/0	0/0	0/0	0/0
1/1	1/1	1/1	0/0	0/0	0/0	0/0	0/0	0/0	1/1	1/1	0/0	1/1	1/1	1/1	0/0	1/1	1/1	1/1	0/0	0/0	1/1	1/1	0/0	1/1	0/0	1/1
1/1	1/1	1/1	0/0	0/0	0/0	0/0	0/0	0/0	1/1	1/1	0/0	1/1	1/1	1/1	0/0	1/1	1/1	1/1	0/0	0/0	1/1	1/1	0/0	1/1	0/0	1/1
0/0	0/0	0/0	0/0	0/0	0/0	0/0	0/0	0/0	0/0	0/0	0/0	0/0	0/0	0/0	0/0	0/0	0/0	0/0	0/0	0/0	0/0	0/0	0/0	0/0	0/0	0/0
0/0	0/0	0/0	0/0	0/0	0/0	0/0	0/0	0/0	1/1	0/0	0/0	0/0	0/0	0/0	0/0	1/1	0/0	1/1	0/0	0/0	1/1	1/1	0/0	1/1	0/0	0/0
1/1	1/1	1/1	0/0	0/0	0/0	0/0	0/0	0/0	0/0	1/1	0/0	0/0	1/1	1/1	0/0	0/0	1/1	0/0	0/0	0/0	0/0	0/0	0/0	0/0	0/0	0/0
1/1	2/2	1/1	0/0	0/0	2/2	2/2	0/0	0/0	1/1	1/1	2/2	1/1	1/1	1/1	2/2	1/1	1/1	0/1	0/0	2/2	1/1	1/1	1/1	1/1	2/2	1/1
1/1	1/1	1/1	0/0	0/0	0/0	0/0	0/0	0/0	1/1	1/1	0/0	1/1	1/1	1/1	0/0	1/1	1/1	1/1	0/0	0/0	1/1	1/1	0/0	1/1	0/0	1/1
1/1	0/0	1/1	0/0	0/0	0/0	0/0	0/0	0/0	0/0	0/0	0/0	0/0	1/1	1/1	0/0	0/0	0/0	0/0	0/0	0/0	0/0	0/0	0/0	0/0	0/0	0/0
1/1	0/0	1/1	0/0	0/0	0/0	0/0	0/0	0/0	0/0	0/0	0/0	0/0	0/1	1/1	0/0	0/0	0/0	0/0	0/0	0/0	0/0	0/0	0/0	0/0	0/0	0/0
0/0	0/0	0/0	0/0	0/0	0/0	0/0	0/0	0/0	1/1	1/1	0/0	0/0	0/1	0/0	0/0	1/1	1/1	1/1	0/0	0/0	1/1	1/1	0/0	0/1	0/0	0/0
0/0	0/0	0/0	0/0	0/0	0/0	0/0	0/0	0/0	0/0	0/0	0/0	0/0	0/0	0/0	0/0	0/0	0/0	0/0	0/0	0/0	0/0	0/0	0/0	0/0	0/0	0/0
0/0	0/0	./.	0/0	0/0	0/0	0/0	0/0	0/0	0/0	0/0	0/0	0/0	0/0	0/0	0/0	0/0	0/0	0/0	0/0	0/0	0/0	0/0	0/0	0/0	0/0	0/0
0/0	0/0	./.	0/0	0/0	0/0	0/1	0/0	0/0	0/0	0/0	0/0	0/2	0/0	0/0	0/0	0/2	0/0	0/0	0/0	0/0	0/0	0/1	0/0	0/0	0/0	0/0
0/0	0/0	./.	0/0	0/0	0/0	0/0	0/0	0/0	0/0	0/0	0/0	0/0	0/0	0/0	0/0	0/0	0/0	0/0	0/0	0/0	0/0	0/0	0/0	0/0	0/0	0/0
0/0	0/0	./.	0/0	0/0	./.	0/1	0/0	0/0	0/0	0/0	0/0	0/1	0/0	0/0	0/0	0/1	0/0	0/0	0/0	0/0	0/0	0/1	0/0	0/0	0/0	0/0
0/1	0/0	./.	0/0	0/0	./.	0/1	0/0	0/0	0/0	0/0	0/0	0/1	0/0	0/0	0/0	0/1	0/0	0/0	0/0	0/0	0/0	0/1	0/0	0/0	0/0	0/0

0/1	0/0	./.	0/0	0/0	0/0	0/1	0/0	0/0	0/0	0/0	0/0	0/1	0/0	0/0	0/0	0/1	0/0	0/0	0/0	0/0	0/0	0/0	0/0	0/0	0/0	0/0
0/1	0/0	./.	0/0	0/0	0/0	0/1	0/0	0/0	0/0	0/0	0/0	0/1	0/0	0/0	0/0	0/1	0/0	0/0	0/0	0/0	0/0	0/0	0/0	0/0	0/0	0/0
0/0	0/0	./.	0/0	0/0	0/0	0/1	0/0	0/0	0/0	0/0	0/0	0/1	0/0	0/0	0/0	0/1	0/0	0/0	0/0	0/0	0/0	0/0	0/0	0/0	0/0	0/0
0/0	0/0	0/0	0/0	0/0	0/0	0/1	0/0	0/0	0/0	0/0	0/0	0/1	0/0	0/0	0/0	0/1	0/0	0/0	0/0	0/0	0/0	0/0	0/0	0/0	0/0	0/0
0/0	0/0	0/0	0/0	0/0	0/0	0/1	0/0	0/0	0/0	0/0	0/0	0/1	0/0	0/0	0/0	0/1	0/0	0/0	0/0	0/0	0/0	0/0	0/0	0/0	0/0	0/0
0/0	0/0	0/0	0/0	0/0	0/0	0/1	0/0	0/0	0/0	0/0	0/0	0/0	0/0	0/0	0/0	0/0	0/0	0/0	0/0	0/0	0/0	0/0	0/0	0/0	0/0	0/0
0/0	0/0	0/0	0/0	0/0	0/0	0/0	0/0	0/0	0/0	0/0	0/0	0/0	0/0	0/0	0/0	0/0	0/0	0/0	0/0	0/0	0/0	0/0	0/0	0/0	0/0	0/0
0/0	0/0	0/0	0/0	0/0	0/0	0/0	0/0	0/0	0/0	0/0	0/0	0/0	0/0	0/0	0/0	0/0	0/0	0/0	0/0	0/0	0/0	0/0	0/0	0/0	0/0	0/0
0/0	0/0	0/0	0/0	0/0	0/0	0/0	0/0	0/0	0/0	0/0	0/0	0/0	0/0	0/0	0/0	0/0	0/0	0/0	0/0	0/0	0/0	0/0	0/0	0/0	0/0	0/0
0/0	0/0	0/0	0/0	0/0	0/0	0/0	0/0	0/0	0/0	0/0	0/0	0/0	0/0	0/0	0/0	0/0	0/0	0/0	0/0	0/0	0/0	0/0	0/0	0/0	0/0	0/0
1/1	1/1	1/1	0/0	0/0	1/1	0/0	0/0	0/0	1/1	1/1	1/1	1/1	1/1	1/1	1/1	1/1	1/1	1/1	0/0	1/1	1/1	1/1	1/1	1/1	1/1	1/1
0/0	0/0	0/0	0/0	0/0	0/0	0/0	0/0	0/0	0/0	0/0	0/0	0/0	0/0	0/0	0/0	0/0	0/0	0/0	0/0	0/0	0/0	0/0	0/0	0/0	0/0	0/0
0/0	1/1	0/0	0/0	0/0	0/0	0/0	0/0	0/0	0/0	0/0	0/0	0/0	0/0	0/0	0/0	0/0	0/0	0/0	0/0	0/0	0/0	0/0	0/0	0/0	0/0	0/0
0/0	0/0	0/0	0/0	0/0	0/0	0/0	0/0	0/0	0/0	0/0	0/0	1/1	0/0	0/0	0/0	0/0	0/0	0/0	0/0	0/0	0/0	0/0	0/0	0/0	0/0	1/1
0/0	0/0	0/0	0/0	0/0	0/0	0/0	0/0	0/0	0/0	0/0	0/0	0/0	0/0	0/0	0/0	0/0	0/0	0/0	0/0	0/0	0/0	0/0	0/0	0/0	0/0	0/0
0/0	0/0	0/0	0/0	0/0	0/0	0/0	0/0	0/0	0/0	0/0	0/0	0/0	0/0	0/0	0/0	0/0	0/0	0/0	0/0	0/0	0/0	0/0	0/0	0/0	0/0	0/0
1/1	1/1	1/1	0/0	0/0	0/0	0/0	0/0	0/0	1/1	1/1	0/0	1/1	1/1	1/1	0/0	1/1	1/1	1/1	0/0	0/0	1/1	1/1	0/0	1/1	0/0	1/1
./.	1/1	0/0	0/0	0/0	0/0	0/0	0/0	0/0	./.	0/0	0/0	0/0	0/0	./.	0/0	./.	0/0	0/0	0/0	0/0	0/0	0/0	0/0	0/0	0/0	0/0
./.	1/1	0/0	0/0	0/0	0/0	0/0	0/0	0/0	1/1	1/1	0/0	1/1	0/0	./.	0/0	1/1	1/1	1/1	0/0	0/0	1/1	1/1	0/0	1/1	0/0	1/1
1/1	0/0	1/1	0/0	0/0	0/0	0/0	0/0	0/0	0/0	0/0	0/0	0/0	1/1	1/1	0/0	0/0	0/0	0/0	0/0	0/0	0/0	0/0	0/0	0/0	0/0	0/0
1/1	0/0	1/1	0/0	0/0	0/0	0/0	0/0	0/0	0/0	0/0	0/0	0/0	0/1	1/1	0/0	0/0	0/0	0/0	0/0	0/0	0/0	./.	0/0	0/0	0/0	0/0
0/0	0/1	0/0	0/0	0/0	0/0	0/0	./.	./.	./.	./.	0/0	0/0	0/0	0/0	0/0	./.	./.	./.	0/0	./.	./.	./.	0/0	./.	0/0	0/0
0/0	0/1	0/0	0/0	0/0	./.	0/0	./.	./.	1/1	./.	0/0	0/1	0/0	0/0	0/0	./.	0/0	./.	0/0	./.	./.	./.	0/0	0/1	0/0	./.
0/0	0/2	0/0	0/0	0/0	./.	0/0	./.	./.	2/2	1/1	0/0	0/1	0/1	0/0	0/0	1/1	1/1	1/1	0/0	./.	0/1	1/1	./.	0/1	0/0	1/1

0/0	0/2	0/0	0/0	0/0	./.	0/0	./.	./.	2/2	2/2	0/0	0/2	0/2	0/0	0/0	0/0	0/0	2/2	0/0	./.	0/1	2/2	./.	0/2	0/0	2/2
0/0	0/4	0/0	0/0	1/1	3/3	0/0	1/1	1/1	4/4	4/4	2/3	0/4	0/4	0/0	3/3	4/4	0/0	4/4	1/1	3/3	0/4	4/4	3/3	0/4	3/3	4/4
0/0	1/1	0/0	0/0	0/0	0/0	0/0	0/0	0/0	0/0	0/0	0/0	0/0	0/0	0/0	0/0	0/0	0/0	0/0	0/0	0/0	0/0	0/0	0/0	0/0	0/0	0/0
1/1	1/1	1/1	0/0	0/0	0/0	0/0	0/0	0/0	1/1	1/1	0/0	1/1	1/1	1/1	0/0	1/1	1/1	1/1	0/0	0/0	1/1	1/1	0/0	0/1	0/0	1/1
0/0	1/1	0/0	0/0	0/0	0/0	0/0	0/0	0/0	1/1	1/1	0/0	1/1	0/0	0/0	0/0	1/1	1/1	1/1	0/0	0/0	1/1	1/1	0/0	0/1	0/0	1/1
0/0	0/0	0/0	0/0	0/0	0/0	0/0	0/0	0/0	0/0	0/0	0/0	0/0	0/0	0/0	0/0	0/0	0/0	0/0	0/0	0/0	0/0	0/0	0/0	0/0	0/0	0/0
0/0	0/0	0/0	0/0	0/0	0/0	0/0	0/0	0/0	0/0	0/0	0/0	0/0	0/0	0/0	0/0	0/0	0/0	0/0	0/0	0/0	0/0	./.	0/0	0/0	0/0	0/0
1/1	1/1	1/1	0/0	0/0	0/0	0/0	0/0	0/0	1/1	1/1	0/0	1/1	1/1	1/1	0/0	1/1	1/1	1/1	0/0	0/0	1/1	1/1	0/0	0/1	0/0	1/1
0/0	./.	0/0	0/0	0/0	0/0	0/0	0/0	0/0	./.	./.	0/0	0/0	0/0	0/0	0/0	./.	0/0	./.	0/0	0/0	./.	0/0	0/0	0/0	0/0	0/0
0/0	2/2	0/0	0/0	0/0	0/0	0/0	0/0	0/0	1/1	1/1	0/0	1/1	0/1	0/0	0/0	1/1	1/1	1/1	0/0	0/0	1/2	1/1	0/0	0/1	0/0	2/2
0/0	1/1	0/0	0/0	0/0	0/0	0/0	0/0	0/0	1/1	1/1	0/0	1/1	0/1	0/0	0/0	1/1	1/1	1/1	0/0	0/0	1/1	1/1	0/0	0/1	0/0	1/1
0/0	0/0	0/0	0/0	0/0	0/0	0/0	0/0	0/0	1/1	1/1	0/0	1/1	0/0	0/0	0/0	1/1	1/1	1/1	0/0	0/0	1/1	1/1	0/0	0/1	0/0	1/1
1/1	0/0	1/1	0/0	0/0	0/0	0/0	0/0	0/0	0/0	0/0	0/0	0/0	0/1	1/1	0/0	0/0	0/0	0/0	0/0	0/0	0/0	0/0	0/0	0/0	0/0	0/0
0/0	1/1	0/0	0/0	0/0	0/0	0/0	0/0	0/0	1/1	1/1	0/0	1/1	0/0	0/0	0/0	1/1	1/1	1/1	0/0	0/0	1/1	1/1	0/0	0/1	0/0	1/1
1/1	1/1	1/1	0/0	0/0	0/0	0/0	0/0	0/0	1/1	1/1	0/0	1/1	1/1	1/1	0/0	1/1	1/1	1/1	0/0	0/0	1/1	1/1	0/0	0/1	0/0	1/1
0/0	0/0	0/0	0/0	0/0	0/0	0/0	0/0	0/0	0/0	0/0	0/0	0/0	0/0	0/0	0/0	0/0	0/0	0/0	0/0	0/0	0/0	0/0	0/0	0/0	0/0	0/0
0/0	0/0	0/0	0/0	0/0	0/0	0/0	0/0	0/0	0/0	0/0	0/0	0/0	0/0	0/0	0/0	0/0	0/0	0/0	0/0	0/0	0/0	0/0	0/0	0/0	0/0	0/0
1/1	0/0	1/1	0/0	0/0	0/0	0/0	0/0	0/0	0/0	0/0	0/0	0/0	0/1	1/1	0/0	0/0	0/0	0/0	0/0	0/0	0/0	0/0	0/0	0/0	0/0	0/0
0/0	0/0	0/0	0/0	0/0	0/0	0/0	0/0	0/0	0/0	0/0	0/0	0/0	0/0	0/0	0/0	0/0	0/0	0/0	0/0	0/0	0/0	0/0	0/0	0/0	0/0	0/0
1/1	0/0	1/1	0/0	0/0	0/0	0/0	0/0	0/0	0/0	0/0	0/0	0/0	0/1	1/1	0/0	0/0	0/0	0/0	0/0	0/0	0/0	0/0	0/0	0/0	0/0	0/0
0/0	0/0	0/0	0/0	0/0	0/0	0/0	0/0	0/0	0/0	0/0	0/0	1/1	0/0	0/0	0/0	0/0	0/0	0/0	0/0	0/0	0/0	0/0	0/0	0/0	0/0	1/1
1/1	0/0	1/1	0/0	0/0	0/0	0/0	0/0	0/0	0/0	0/0	0/0	0/0	1/1	1/1	0/0	0/0	0/0	0/0	0/0	0/0	0/0	0/0	0/0	0/0	0/0	0/0
1/1	0/0	1/1	0/0	0/0	0/0	0/0	0/0	0/0	0/0	0/0	0/0	0/0	1/1	1/1	0/0	0/0	0/0	0/0	0/0	0/0	0/0	0/0	0/0	0/0	0/0	0/0
0/0	1/1	0/0	0/0	0/0	0/0	0/0	0/0	0/0	0/0	0/0	0/0	0/0	0/0	0/0	0/0	0/0	0/0	0/0	0/0	0/0	0/0	0/0	0/0	0/0	0/0	0/0
0/0	1/1	0/0	0/0	0/0	0/0	0/0	0/0	0/0	0/0	0/0	0/0	0/0	0/0	0/0	0/0	0/0	0/0	0/0	0/0	0/0	0/0	0/0	0/0	0/0	0/0	0/0
0/0	1/1	0/0	0/0	0/0	0/0	0/0	0/0	0/0	1/1	1/1	0/0	1/1	0/0	0/0	0/0	1/1	1/1	1/1	0/0	0/0	1/1	1/1	0/0	1/1	0/0	1/1
0/0	0/0	0/0	0/0	0/0	0/0	0/0	0/0	0/0	0/0	0/0	0/0	0/0	0/0	0/0	0/0	0/0	0/0	0/0	0/0	0/0	0/0	0/0	0/0	0/0	0/0	0/0
1/1	1/1	1/1	0/0	0/0	0/0	0/0	0/0	0/0	1/1	1/1	0/0	1/1	1/1	1/1	0/0	1/1	1/1	1/1	0/0	0/0	1/1	1/1	0/0	1/1	0/0	1/1
0/0	0/0	0/0	0/0	0/0	0/0	0/0	0/0	0/0	0/0	0/0	0/0	0/0	0/0	0/0	0/0	0/0	0/0	0/0	0/0	0/0	0/0	0/0	0/0	0/0	0/0	0/0
1/1	1/1	1/1	0/0	0/0	0/0	0/0	0/0	0/0	1/1	1/1	0/0	1/1	1/1	1/1	0/0	1/1	1/1	1/1	0/0	0/0	1/1	1/1	0/0	1/1	0/0	1/1

0/0	1/1	0/0	0/0	0/0	0/0	0/0	0/0	0/0	0/0	0/0	0/0	0/0	0/0	0/0	0/0	0/0	0/0	0/0	0/0	0/0	0/0	0/0	0/0	0/0	0/0	0/0
0/0	0/0	0/0	0/0	0/0	0/0	0/0	0/0	0/0	0/0	0/0	0/0	0/0	0/0	0/0	0/0	0/0	0/0	0/0	0/0	0/0	0/0	0/0	0/0	0/0	0/0	0/0
0/0	0/0	0/0	0/0	0/0	0/0	0/0	0/0	0/0	0/0	0/0	0/0	0/0	0/0	0/0	0/0	0/0	0/0	0/0	0/0	0/0	0/0	0/0	0/0	0/0	0/0	0/0
0/0	0/0	0/0	0/0	0/0	0/0	0/0	0/0	0/0	1/1	1/1	0/0	0/0	0/0	0/0	0/0	1/1	1/1	1/1	0/0	0/0	1/1	1/1	0/0	1/1	0/0	0/0
0/0	1/1	0/0	0/0	0/0	0/0	0/0	0/0	0/0	1/1	1/1	0/0	1/1	0/0	0/0	0/0	1/1	1/1	1/1	0/0	0/0	1/1	1/1	0/0	1/1	0/0	1/1
1/1	1/1	1/1	0/0	0/0	0/0	0/0	0/0	0/0	1/1	1/1	0/0	1/1	1/1	1/1	0/0	1/1	1/1	1/1	0/0	0/0	1/1	1/1	0/0	1/1	0/0	1/1
0/0	1/1	0/0	0/0	0/0	0/0	0/0	0/0	0/0	1/1	1/1	0/0	1/1	0/0	0/0	0/0	1/1	1/1	1/1	0/0	0/0	1/1	1/1	0/0	1/1	0/0	1/1
0/0	0/0	0/0	0/0	0/0	0/0	0/0	0/0	0/0	0/0	0/0	0/0	1/1	0/0	0/0	0/0	0/0	0/0	0/0	0/0	0/0	0/0	0/0	0/0	0/0	0/0	1/1
0/0	0/0	0/0	0/0	0/0	0/0	0/0	0/0	0/0	0/0	0/0	0/0	0/0	0/0	0/0	0/0	0/0	0/0	0/0	0/0	0/0	0/0	0/0	0/0	0/0	0/0	0/0
0/0	0/0	0/0	0/0	0/0	0/0	0/0	0/0	0/0	0/0	0/0	0/0	0/0	0/0	0/0	0/0	0/0	0/0	0/0	0/0	0/0	0/0	0/0	0/0	0/0	0/0	0/0
0/0	0/0	0/0	0/0	0/0	0/0	0/0	0/0	0/0	0/0	0/0	0/0	0/0	0/0	0/0	0/0	0/0	0/0	0/0	0/0	0/0	0/0	0/0	0/0	0/0	0/0	0/0
1/1	0/0	1/1	0/0	0/0	0/0	0/0	0/0	0/0	0/0	0/0	0/0	0/0	1/1	1/1	0/0	0/0	0/0	0/0	0/0	0/0	0/0	0/0	0/0	0/0	0/0	0/0
1/1	2/2	1/1	0/0	0/0	0/0	0/0	0/0	0/0	1/1	1/1	0/0	0/0	1/1	1/1	0/0	1/1	1/1	1/1	0/0	0/0	1/1	1/1	0/0	1/1	0/0	0/1
1/1	1/1	1/1	0/0	0/0	0/0	0/0	0/0	0/0	1/1	1/1	0/0	1/1	1/1	1/1	0/0	1/1	1/1	1/1	0/0	0/0	1/1	1/1	0/0	0/1	0/0	1/1
1/1	1/1	1/1	1/1	1/1	1/1	1/1	1/1	1/1	1/1	1/1	1/1	1/1	1/1	1/1	1/1	1/1	1/1	1/1	1/1	1/1	1/1	1/1	1/1	1/1	1/1	1/1
0/0	0/0	0/0	0/0	0/0	0/0	0/0	0/0	0/0	0/0	0/0	0/0	0/0	0/0	0/0	0/0	0/0	0/0	0/0	0/0	0/0	0/0	0/0	0/0	0/0	0/0	0/0
0/0	0/0	0/0	0/0	0/0	0/0	0/0	0/0	0/0	1/1	1/1	0/0	0/0	0/0	0/0	0/0	1/1	1/1	1/1	0/0	0/0	1/1	1/1	0/0	1/1	0/0	0/0
0/0	1/1	0/0	0/0	0/0	0/0	0/0	0/0	0/0	1/1	1/1	0/0	1/1	0/0	0/0	0/0	1/1	1/1	1/1	0/0	0/0	1/1	1/1	0/0	1/1	0/0	1/1
1/1	1/1	1/1	0/0	0/0	0/0	0/0	0/0	0/0	1/1	1/1	0/0	1/1	1/1	1/1	0/0	1/1	1/1	1/1	0/0	0/0	1/1	1/1	0/0	1/1	0/0	1/1
0/0	0/0	0/0	0/0	0/0	0/0	0/0	0/0	0/0	0/0	0/0	0/0	0/0	0/0	0/0	0/0	0/0	0/0	0/0	0/0	0/0	0/0	0/0	0/0	0/0	0/0	0/0
0/0	0/0	0/0	0/0	0/0	0/0	0/0	0/0	0/0	0/0	0/0	0/0	0/0	0/0	0/0	0/0	0/0	0/0	0/0	0/0	0/0	0/0	0/0	0/0	0/0	0/0	0/0
0/0	1/1	0/0	0/0	0/0	0/0	0/0	0/0	0/0	0/0	0/0	0/0	0/0	0/0	0/0	0/0	0/0	0/0	0/0	0/0	0/0	0/0	0/0	0/0	0/0	0/0	0/0
1/1	1/1	1/1	0/0	0/0	0/0	0/0	0/0	0/0	1/1	1/1	0/0	1/1	1/1	1/1	0/0	1/1	1/1	1/1	0/0	0/0	1/1	1/1	0/0	1/1	0/0	1/1
0/0	0/0	0/0	0/0	0/0	0/0	0/0	0/0	0/0	0/0	0/0	0/0	0/0	0/0	0/0	0/0	0/0	0/0	0/0	0/0	0/0	0/0	0/0	0/0	0/0	0/0	0/0
0/0	1/1	0/0	0/0	0/0	0/0	0/0	0/0	0/0	0/0	0/0	0/0	0/0	0/0	0/0	0/0	0/0	0/0	0/0	0/0	0/0	0/0	0/0	0/0	0/0	0/0	0/0
0/0	0/0	0/0	0/0	0/0	0/0	0/0	0/0	0/0	0/0	0/0	0/0	0/0	0/0	0/0	0/0	0/0	0/0	0/0	0/0	0/0	0/0	0/0	0/0	0/0	0/0	0/0
0/0	1/1	0/0	0/0	0/0	0/0	0/0	0/0	0/0	0/0	0/0	0/0	0/0	0/0	0/0	0/0	0/0	0/0	0/0	0/0	0/0	0/0	0/0	0/0	0/0	0/0	0/0
0/0	0/0	0/0	0/0	0/0	0/0	0/0	0/0	0/0	0/0	0/0	0/0	0/0	0/0	0/0	0/0	0/0	0/0	0/0	0/0	0/0	0/0	0/0	0/0	0/0	0/0	0/0
0/0	0/0	0/0	0/0	0/0	0/0	0/0	0/0	0/0	0/0	0/0	0/0	0/0	0/0	0/0	0/0	0/0	0/0	0/0	0/0	0/0	0/0	0/0	0/0	0/0	0/0	0/0
0/0	0/0	0/0	0/0	0/0	0/0	0/0	0/0	0/0	0/0	0/0	0/0	0/0	0/0	0/0	0/0	0/0	0/0	0/0	0/0	0/0	0/0	0/0	0/0	0/0	0/0	0/0
0/0	0/0	0/0	0/0	0/0	0/0	0/0	0/0	0/0	0/0	0/0	0/0	0/0	0/0	0/0	0/0	0/0	0/0	0/0	0/0	0/0	0/0	0/0	0/0	0/0	0/0	0/0
1/1	0/0	1/1	0/0	0/0	0/0	0/0	0/0	0/0	0/0	0/0	0/0	0/0	0/1	1/1	0/0	0/0	0/0	0/0	0/0	0/0	0/0	0/0	0/0	0/0	0/0	0/0
0/0	0/0	0/0	0/0	0/0	0/0	0/0	0/0	0/0	0/0	0/0	0/0	0/0	0/0	0/0	0/0	0/0	0/0	0/0	0/0	0/0	0/0	0/0	0/0	0/0	0/0	0/0
0/0	0/0	0/0	0/0	0/0	0/0	0/0	0/0	0/0	0/0	0/0	0/0	0/0	0/0	0/0	0/0	0/0	0/0	0/0	0/0	0/0	0/0	0/0	0/0	0/0	0/0	1/1
0/0	0/0	0/0	0/0	0/0	0/0	0/0	0/0	0/0	0/0	1/1	0/0	0/0	0/1	0/0	0/0	1/1	1/1	1/1	0/0	0/0	1/1	1/1	0/0	0/0	0/0	0/0

0/0	0/0	0/0	0/0	0/0	0/0	0/0	0/0	0/0	0/0	0/0	0/0	1/1	0/0	0/0	0/0	0/0	0/0	0/0	0/0	0/0	0/0	0/0	0/0	0/0	0/0	1/1
2/3	4/4	1/4	4/4	3/5	2/2	3/3	3/5	3/3	2/3	2/3	2/3	4/4	1/2	2/3	2/2	2/3	2/3	2/3	3/5	2/2	2/2	2/3	3/5	2/2	2/2	0/4
./.	./.	./.	./.	./.	./.	./.	./.	./.	./.	./.	./.	./.	./.	./.	./.	./.	./.	./.	./.	./.	./.	./.	./.	./.	./.	./.
0/0	0/0	0/0	0/0	0/0	0/0	0/0	0/0	0/0	0/0	0/0	0/0	0/0	0/0	0/0	0/0	0/0	0/0	0/0	0/0	0/0	0/0	0/0	0/0	0/0	0/0	0/0
0/0	0/0	0/0	0/0	0/0	0/0	0/0	0/0	0/0	0/0	0/0	0/0	0/0	0/0	0/0	0/0	0/0	0/0	0/0	0/0	0/0	0/0	0/0	0/0	0/0	0/0	0/0
0/0	1/1	0/0	0/0	0/0	0/0	0/0	0/0	0/0	0/0	0/0	0/0	0/0	0/0	0/0	0/0	0/0	0/0	0/0	0/0	0/0	0/0	0/0	0/0	0/0	0/0	0/0
0/0	0/0	0/0	0/0	0/0	0/0	0/0	0/0	0/0	0/0	0/0	0/0	1/1	0/0	0/0	0/0	0/0	0/0	0/0	0/0	0/0	0/0	0/0	0/0	0/0	0/0	1/1
0/0	0/0	0/0	0/0	0/0	0/0	./.	0/0	0/0	0/0	./.	0/0	1/1	0/0	0/0	0/0	0/0	0/0	./.	0/0	0/0	0/0	0/0	0/0	0/0	0/0	1/1
0/0	1/1	./.	0/0	0/0	0/0	./.	./.	0/0	0/0	./.	./.	./.	0/0	0/0	0/0	0/0	0/0	./.	0/0	0/0	0/0	./.	0/0	0/0	0/0	0/0
0/0	1/1	./.	0/0	0/0	./.	./.	./.	0/0	1/1	1/1	./.	1/1	0/1	0/0	0/0	1/1	1/1	1/1	0/0	./.	1/1	1/1	0/0	0/1	./.	1/1
2/2	./.	2/2	0/0	1/1	1/1	1/1	1/1	1/1	./.	./.	1/1	./.	0/2	2/2	1/1	./.	./.	./.	1/1	1/1	./.	./.	1/1	0/1	1/1	./.
./.	./.	./.	0/0	2/2	2/2	2/2	2/2	2/2	0/0	./.	2/2	0/0	./.	./.	2/2	./.	./.	0/0	2/2	2/2	./.	0/0	2/2	0/2	2/2	0/0
0/0	1/1	0/0	0/0	0/0	0/0	0/0	0/0	0/0	0/0	0/0	0/0	0/0	0/0	0/0	0/0	0/0	0/0	0/0	0/0	0/0	0/0	0/0	0/0	0/0	0/0	0/0
0/0	0/0	0/0	0/0	0/0	0/0	0/0	0/0	0/0	0/0	0/0	0/0	0/0	0/0	0/0	0/0	0/0	0/0	0/0	0/0	0/0	0/0	0/0	0/0	0/0	0/0	0/0
0/0	0/0	0/0	0/0	0/0	0/0	0/0	0/0	0/0	0/0	0/0	0/0	0/0	0/0	0/0	0/0	0/0	0/0	0/0	0/0	0/0	0/0	0/0	0/0	0/0	0/0	0/0
1/1	1/1	1/1	0/0	0/0	0/0	0/0	0/0	0/0	1/1	1/1	0/0	1/1	1/1	1/1	0/0	1/1	1/1	1/1	0/0	0/0	1/1	1/1	0/0	0/1	0/0	1/1
0/0	0/0	0/0	0/0	0/0	0/0	0/0	0/0	0/0	0/0	0/0	0/0	0/0	0/0	0/0	0/0	0/0	0/0	0/0	0/0	0/0	0/0	0/0	0/0	0/0	0/0	0/0
0/0	0/0	0/0	0/0	0/0	0/0	0/0	0/0	0/0	1/1	1/1	0/0	0/0	0/0	0/0	0/0	1/1	1/1	1/1	0/0	0/0	1/1	1/1	0/0	0/1	0/0	0/0
0/0	0/0	0/0	0/0	0/0	0/0	0/0	0/0	0/0	0/0	0/0	0/0	0/0	0/0	0/0	0/0	0/0	0/0	0/0	0/0	0/0	0/0	0/0	0/0	0/0	0/0	0/0
0/0	0/0	0/0	0/0	0/0	0/0	0/0	0/0	0/0	0/0	0/0	0/0	0/0	0/0	0/0	0/0	0/0	0/0	0/0	0/0	0/0	0/0	0/0	0/0	0/0	0/0	0/0
0/0	0/0	0/0	0/0	0/0	0/0	0/0	0/0	0/0	0/0	0/0	0/0	0/0	0/0	0/0	0/0	0/0	0/0	0/0	0/0	0/0	0/0	0/0	0/0	0/0	0/0	0/0

0/0	0/0	0/0	0/0	0/0	0/0	0/0	0/0	0/0	0/0	0/0	0/0	0/0	0/0	0/0	0/0	0/0	0/0	0/0	0/0	0/0	0/0	0/0	0/0	0/0	0/0	0/0
0/0	0/0	0/0	0/0	0/0	0/0	0/0	0/0	0/0	0/0	0/0	0/0	0/0	0/0	0/0	0/0	0/0	0/0	0/0	0/0	0/0	0/0	0/0	0/0	0/0	0/0	0/0
2/2	1/1	2/2	0/0	0/0	0/0	0/0	0/0	0/0	1/1	1/1	0/0	1/1	2/2	2/2	0/0	1/1	1/1	1/1	0/0	0/0	1/1	1/1	0/0	0/1	0/0	1/1
0/0	0/0	0/0	0/0	0/0	0/0	0/0	0/0	0/0	0/0	0/0	0/0	0/0	0/0	0/0	0/0	0/0	0/0	0/0	0/0	0/0	0/0	0/0	0/0	0/0	0/0	0/0
2/2	0/0	2/2	0/0	0/0	0/0	0/0	0/0	0/0	1/1	1/1	0/0	0/0	1/2	2/2	0/0	1/1	1/1	1/1	0/0	0/0	1/1	1/1	0/0	0/1	0/0	0/0
0/0	0/0	0/0	0/0	0/0	0/0	0/0	0/0	0/0	0/0	0/0	0/0	0/0	0/0	0/0	0/0	0/0	0/0	0/0	0/0	0/0	0/0	0/0	0/0	0/0	0/0	0/0
0/0	0/0	0/0	0/0	0/0	0/0	0/0	0/0	0/0	0/0	0/0	0/0	1/1	0/2	0/0	0/0	0/0	0/0	0/0	0/0	0/0	0/0	0/0	0/0	0/0	0/0	1/1
1/1	1/1	1/1	0/0	0/0	0/0	0/0	0/0	0/0	1/1	1/1	0/0	1/1	1/1	1/1	0/0	1/1	1/1	1/1	0/0	0/0	1/1	1/1	0/0	1/1	0/0	1/1
1/1	1/1	1/1	0/0	0/0	0/0	0/0	0/0	0/0	1/1	1/1	0/0	1/1	1/1	1/1	0/0	1/1	1/1	1/1	0/0	0/0	1/1	1/1	0/0	1/1	0/0	1/1
0/0	0/0	0/0	0/0	0/0	0/0	0/0	0/0	0/0	0/0	0/0	0/0	0/0	0/0	0/0	0/0	0/0	0/0	0/0	0/0	0/0	0/0	0/0	0/0	0/0	0/0	0/0
2/2	2/2	2/2	0/0	0/0	0/0	0/0	0/0	0/0	1/1	1/1	0/0	2/2	2/2	2/2	0/0	1/1	1/1	1/1	0/0	0/0	1/1	1/1	0/0	1/1	0/0	2/2
0/0	0/0	0/0	0/0	0/0	0/0	0/0	0/0	0/0	0/0	0/0	0/0	0/0	0/0	0/0	0/0	0/0	0/0	0/0	0/0	0/0	0/0	0/0	0/0	0/0	0/0	0/0
0/0	0/0	0/0	0/0	0/0	0/0	0/0	0/0	0/0	0/0	0/0	0/0	0/0	0/0	0/0	0/0	0/0	0/0	0/0	0/0	0/0	0/0	0/0	0/0	0/0	0/0	0/0
0/0	0/0	0/0	0/0	0/0	0/0	0/0	0/0	0/0	0/0	0/0	0/0	0/0	0/0	0/0	0/0	0/0	0/0	0/0	0/0	0/0	0/0	0/1	0/0	0/0	0/0	0/0
0/0	1/1	0/0	0/0	0/0	0/0	0/0	0/0	0/0	0/0	0/0	0/0	1/1	0/0	0/0	0/0	0/0	0/0	0/0	0/0	0/0	0/0	0/0	0/0	0/0	0/0	1/1
0/0	0/0	0/0	0/0	0/0	0/0	0/0	0/0	0/0	0/0	0/0	0/0	0/0	0/0	0/0	0/0	0/0	0/0	0/0	0/0	0/0	0/0	0/0	0/0	0/0	0/0	0/0
0/0	0/0	0/0	0/0	0/0	0/0	0/0	0/0	0/0	0/0	0/0	0/0	1/1	0/0	0/0	0/0	0/0	0/0	0/0	0/0	0/0	0/0	0/0	0/0	0/0	0/0	1/1
1/1	1/1	1/1	0/0	0/0	0/0	0/0	0/0	0/0	1/1	1/1	0/0	1/1	1/1	1/1	0/0	1/1	1/1	1/1	0/0	0/0	1/1	1/1	0/0	1/1	0/0	1/1

1329	1332	1334	1341	1342	1361	1362	1365	1370	1377	1399	1400	1420	1436	1449	14	20	25	30	37	39	414	423	436	440	442	450
0/0	0/0	./.	0/0	0/0	0/0	0/0	0/0	0/0	0/0	0/0	0/0	0/0	0/0	0/0	0/0	0/0	0/0	0/0	0/0	0/0	0/0	0/0	0/0	0/0	0/0	0/0
0/0	0/0	./.	1/1	1/1	0/0	1/1	1/1	1/1	0/0	1/1	0/0	1/1	0/0	1/1	1/1	0/0	0/0	0/0	0/0	0/0	0/0	0/0	0/0	0/0	0/0	0/0
0/0	0/0	./.	0/0	0/0	0/0	0/0	0/0	0/0	0/0	0/0	0/0	0/0	0/0	0/0	0/0	0/0	0/0	0/0	0/0	0/0	0/0	0/0	0/0	0/0	0/0	0/0
0/0	0/0	./.	0/0	0/0	0/0	0/0	0/0	0/0	0/0	0/0	1/1	0/0	0/0	0/0	0/0	0/0	0/0	0/0	0/0	0/0	0/0	0/0	0/0	0/0	0/0	0/0
0/0	1/1	./.	0/0	0/0	1/1	0/0	0/0	0/0	0/0	0/0	1/1	0/0	1/1	0/0	0/0	0/0	1/1	1/1	0/0	1/1	0/0	0/0	0/0	1/1	1/1	1/1
0/0	1/1	./.	0/0	0/0	1/1	0/0	0/0	0/0	0/0	0/0	1/1	0/0	1/1	0/0	0/0	0/0	1/1	1/1	0/0	1/1	0/0	0/0	0/0	1/1	1/1	1/1
0/0	0/0	./.	0/0	0/0	0/0	0/0	0/0	0/0	0/0	0/0	0/0	0/0	0/0	0/0	0/0	0/0	0/0	0/0	0/0	0/0	0/0	0/0	0/1	0/0	0/0	0/0
0/0	0/0	./.	1/1	1/1	0/0	1/1	1/1	1/1	0/0	1/1	0/0	1/1	0/0	1/1	1/1	1/1	0/0	0/0	0/0	0/0	1/1	1/1	./.	0/0	0/0	0/0
0/0	0/0	./.	0/0	0/0	0/0	0/0	0/0	0/0	0/0	0/0	0/0	0/0	0/0	0/0	0/0	0/0	0/0	0/0	0/0	0/0	0/0	0/0	0/0	0/0	0/0	0/0
0/0	0/0	./.	0/0	0/1	0/0	0/0	0/0	0/0	0/0	0/0	0/0	0/0	0/0	0/0	0/0	0/0	0/0	0/0	0/0	0/0	0/0	0/0	0/0	0/0	0/0	0/0
0/0	0/0	./.	0/0	0/0	0/1	0/0	0/0	0/0	0/0	0/0	0/0	0/1	0/0	0/0	0/0	0/0	0/0	0/0	0/0	0/0	0/0	0/0	0/0	0/0	0/0	0/0
0/0	0/0	./.	0/0	0/0	0/0	0/0	0/0	0/0	0/0	0/0	0/0	0/0	0/0	0/0	0/0	0/0	0/0	0/0	0/0	0/0	0/0	0/0	0/0	0/0	0/0	0/0
0/0	1/1	./.	0/0	0/0	1/1	0/0	0/0	0/0	0/0	0/0	1/1	0/0	1/1	0/0	0/0	0/0	1/1	1/1	0/0	1/1	0/0	0/0	0/0	1/1	1/1	1/1
0/0	0/0	./.	0/0	./.	0/0	0/0	./.	0/1	0/0	./.	0/0	0/0	0/0	0/0	./.	0/0	0/0	0/0	0/0	0/0	0/0	0/0	0/0	0/0	./.	0/0
0/0	./.	./.	1/1	./.	0/0	1/1	1/1	0/1	0/0	1/1	0/0	1/1	0/0	1/1	1/1	1/1	0/0	0/0	0/0	./.	1/1	1/1	1/1	0/0	./.	0/0
0/0	./.	./.	0/0	./.	0/0	0/0	./.	0/0	0/0	./.	0/0	0/0	0/0	0/0	./.	./.	0/0	0/0	0/0	./.	0/0	0/0	0/0	0/0	./.	0/0

4/4	2/3	./.	3/3	./.	2/3	3/3	3/3	3/5	0/0	./.	./.	1/1	3/6	./.	./.	1/1	0/2	2/2	0/4	./.	0/1	1/1	0/4	0/1	1/2	3/6
0/0	0/0	./.	0/0	./.	0/0	0/0	0/0	0/0	0/0	0/0	0/0	0/0	0/0	0/0	./.	0/0	0/0	0/0	0/0	0/0	0/0	0/0	0/0	0/0	0/0	0/0
0/0	0/0	./.	0/0	./.	0/0	0/0	0/0	0/0	0/0	./.	0/0	0/0	0/0	./.	./.	0/0	0/0	0/0	0/0	0/0	0/0	0/0	0/0	0/0	0/0	0/0
0/0	1/1	./.	0/0	0/0	1/1	0/0	0/0	0/0	0/0	0/0	0/0	0/0	1/1	0/0	0/0	0/0	1/1	1/1	0/0	1/1	0/0	0/0	0/0	1/1	1/1	1/1
0/0	0/0	./.	1/1	./.	0/0	1/1	1/1	1/1	0/0	1/1	0/0	1/1	0/0	1/1	1/1	1/1	0/0	0/0	0/0	0/0	1/1	1/1	1/1	0/0	0/0	0/0
0/0	0/0	./.	0/0	0/0	0/0	0/0	0/0	0/0	0/0	0/0	1/1	0/0	0/0	0/0	0/0	0/0	0/0	0/0	0/0	0/0	0/0	0/0	0/0	0/0	0/0	0/0
0/0	0/0	./.	1/1	1/1	0/0	./.	1/1	1/1	0/0	1/1	0/0	1/1	0/0	1/1	1/1	1/1	0/0	0/0	0/0	0/0	1/1	1/1	1/1	0/0	0/0	0/0
0/0	1/1	./.	0/0	0/0	1/1	0/0	0/0	0/0	0/0	0/0	0/0	0/0	1/1	0/0	0/0	0/0	1/1	1/1	0/0	1/1	0/0	0/0	0/0	1/1	1/1	1/1
0/0	0/0	./.	0/0	0/0	0/0	0/0	0/0	0/0	0/0	0/0	0/0	0/0	0/0	0/0	0/0	0/0	0/0	0/0	0/0	0/0	0/0	0/0	0/0	0/0	0/0	0/0
0/0	1/1	./.	0/0	0/0	1/1	0/0	0/0	0/0	0/0	0/0	1/1	0/0	1/1	0/0	0/0	1/1	1/1	1/1	0/0	1/1	1/1	1/1	1/1	1/1	1/1	1/1
0/0	0/0	./.	0/0	0/0	0/0	0/0	0/0	0/0	0/0	0/0	0/0	0/0	0/0	0/0	0/0	0/0	0/0	0/0	0/0	0/0	0/0	0/0	0/0	0/0	0/0	0/0
0/0	1/1	./.	0/0	0/0	1/1	0/0	0/0	0/0	0/0	0/0	0/0	0/0	1/1	0/0	0/0	0/0	1/1	1/1	0/0	1/1	0/0	0/0	0/0	1/1	1/1	1/1
0/0	1/1	./.	1/1	1/1	1/1	1/1	1/1	1/1	0/0	1/1	1/1	1/1	1/1	1/1	1/1	1/1	1/1	1/1	0/0	1/1	1/1	1/1	1/1	1/1	1/1	1/1
0/0	0/0	./.	0/0	0/0	0/0	0/0	0/0	0/0	0/0	0/0	1/1	0/0	0/0	0/0	0/0	0/0	0/0	0/0	0/0	0/0	0/0	0/0	0/0	0/0	0/0	0/0
0/0	0/0	./.	0/0	0/0	0/0	0/0	0/0	0/0	0/0	0/0	0/0	0/0	0/0	0/0	0/0	0/0	0/0	0/0	0/0	0/0	0/0	0/0	0/0	0/0	0/0	0/0
0/0	1/1	./.	1/1	1/1	1/1	1/1	1/1	1/1	0/0	1/1	1/1	1/1	1/1	1/1	1/1	1/1	1/1	1/1	0/0	1/1	1/1	1/1	1/1	1/1	1/1	1/1
0/0	1/1	./.	0/0	0/0	1/1	0/0	0/0	0/0	0/0	0/0	0/0	0/0	1/1	0/0	0/0	0/0	1/1	1/1	0/0	1/1	0/0	0/0	0/0	1/1	1/1	1/1
0/0	0/0	./.	0/0	0/0	0/0	0/0	0/0	0/0	0/0	0/0	1/1	0/0	0/0	0/0	0/0	0/0	0/0	0/0	0/0	0/0	0/0	0/0	0/0	0/0	0/0	0/0
0/0	0/0	./.	1/1	1/1	0/0	1/1	1/1	1/1	0/0	1/1	0/0	1/1	0/0	1/1	1/1	1/1	0/0	0/0	0/0	0/0	1/1	1/1	1/1	0/0	0/0	0/0
0/0	0/0	./.	1/1	1/1	0/0	1/1	1/1	1/1	0/0	1/1	0/0	1/1	0/0	1/1	1/1	0/0	0/0	0/0	0/0	0/0	0/0	0/0	0/0	0/0	0/0	0/0
0/0	0/0	./.	0/0	0/0	0/0	0/0	0/0	0/0	0/0	0/0	1/1	0/0	0/0	0/0	0/0	0/0	0/0	0/0	0/0	0/0	0/0	0/0	0/0	0/0	0/0	0/0
0/0	0/0	./.	1/1	1/1	0/0	1/1	1/1	1/1	0/0	1/1	0/0	1/1	0/0	1/1	1/1	1/1	0/0	0/0	0/0	0/0	1/1	1/1	1/1	0/0	0/0	0/0
0/0	0/0	./.	0/0	0/0	0/0	0/0	0/0	0/0	0/0	0/0	1/1	0/0	0/0	0/0	0/0	0/0	0/0	0/0	0/0	0/0	0/0	0/0	0/0	0/0	0/0	0/0
0/0	1/1	./.	1/1	1/1	1/1	1/1	1/1	1/1	0/0	1/1	1/1	1/1	1/1	1/1	1/1	1/1	1/1	1/1	0/0	1/1	1/1	1/1	1/1	1/1	1/1	1/1
0/0	1/1	./.	0/0	0/0	1/1	0/0	0/0	0/0	0/0	0/0	0/0	0/0	1/1	0/0	0/0	0/0	1/1	1/1	0/0	1/1	0/0	0/0	0/0	1/1	1/1	1/1
0/0	0/0	./.	1/1	1/1	0/0	1/1	1/1	1/1	0/0	0/1	2/2	1/1	0/0	1/1	1/1	0/0	0/0	0/0	0/0	0/0	0/0	0/0	0/0	0/0	0/0	0/0

1/1	1/1	./.	1/1	1/1	1/1	1/1	1/1	1/1	0/0	1/1	1/1	1/1	1/1	1/1	1/1	1/1	1/1	1/1	0/0	1/1	1/1	1/1	1/1	1/1	1/1	1/1
0/0	1/1	./.	0/0	0/0	1/1	0/0	0/0	0/0	0/0	0/0	1/1	0/0	1/1	0/0	0/0	0/0	1/1	1/1	0/0	1/1	0/0	0/0	0/0	1/1	1/1	1/1
0/0	1/1	./.	0/0	0/0	1/1	0/0	0/0	0/0	0/0	0/0	0/0	0/0	1/1	0/0	0/0	0/0	1/1	1/1	0/0	1/1	0/0	0/0	0/0	1/1	1/1	1/1
0/0	0/0	./.	0/0	0/0	0/0	0/0	0/0	0/0	0/0	0/0	0/0	0/0	0/0	0/0	0/0	0/0	0/0	0/0	0/0	0/0	0/0	0/0	0/0	0/0	0/0	0/0
0/0	1/1	./.	1/1	1/1	1/1	1/1	1/1	1/1	0/0	1/1	1/1	1/1	1/1	1/1	1/1	1/1	1/1	1/1	0/0	1/1	1/1	1/1	1/1	1/1	1/1	1/1
0/0	1/1	./.	1/1	1/1	1/1	1/1	1/1	1/1	0/0	1/1	1/1	1/1	1/1	1/1	1/1	1/1	1/1	1/1	0/0	1/1	1/1	1/1	1/1	1/1	1/1	1/1
0/0	1/1	./.	1/1	1/1	1/1	1/1	1/1	1/1	0/0	1/1	1/1	1/1	1/1	1/1	1/1	1/1	1/1	1/1	0/0	1/1	1/1	1/1	1/1	1/1	1/1	1/1
0/0	0/0	./.	0/0	0/0	0/0	0/0	0/0	0/0	0/0	0/0	1/1	0/0	0/0	0/0	0/0	0/0	0/0	0/0	0/0	0/0	0/0	0/0	0/0	0/0	0/0	0/0
0/0	1/1	./.	0/0	0/0	1/1	0/0	0/0	0/0	0/0	0/0	0/0	0/0	1/1	0/0	0/0	0/0	1/1	1/1	0/0	1/1	0/0	0/0	0/0	1/1	1/1	1/1
0/0	0/0	./.	0/0	0/0	0/0	0/0	0/0	0/0	0/0	0/0	0/0	0/0	0/0	0/0	0/0	0/0	0/0	0/0	0/0	0/0	0/0	0/0	0/0	0/0	0/0	0/0
0/0	0/0	./.	1/1	1/1	0/0	1/1	1/1	1/1	0/0	1/1	0/0	1/1	0/0	1/1	1/1	1/1	0/0	0/0	0/0	0/0	1/1	1/1	1/1	0/0	0/0	0/0
0/0	0/0	./.	0/0	0/0	0/0	0/0	0/0	0/0	0/0	0/0	0/0	0/0	0/0	0/0	0/0	0/0	0/0	0/0	0/0	0/0	0/0	0/0	0/0	0/0	0/0	0/0
0/0	0/0	./.	0/0	0/0	0/0	0/0	0/0	0/0	0/0	0/0	0/0	0/0	0/0	0/0	0/0	0/0	0/0	0/0	0/0	0/0	0/0	0/0	0/0	0/0	0/0	0/0
0/0	0/0	./.	0/0	0/0	0/0	0/0	0/0	0/0	0/0	0/0	0/0	0/0	0/0	0/0	0/0	1/1	0/0	0/0	0/0	0/0	1/1	1/1	1/1	0/0	0/0	0/0
0/0	1/1	./.	1/1	1/1	1/1	1/1	1/1	1/1	0/0	1/1	1/1	1/1	1/1	1/1	1/1	1/1	1/1	1/1	0/0	1/1	1/1	1/1	1/1	1/1	1/1	1/1
0/0	0/0	./.	0/0	0/0	0/0	0/0	0/0	0/0	0/0	0/0	1/1	0/0	0/0	0/0	0/0	0/0	0/0	0/0	0/0	0/0	0/0	0/0	0/0	0/0	0/0	0/0
0/0	0/0	./.	0/0	0/0	0/0	0/0	0/0	0/0	0/0	0/0	0/0	0/0	0/0	0/0	0/0	0/0	0/0	0/0	0/0	0/0	0/0	0/0	0/0	0/0	0/0	0/0
0/0	1/1	./.	0/0	0/0	0/0	0/0	0/0	0/0	0/0	0/0	0/0	0/0	1/1	0/0	0/0	0/0	0/0	0/0	0/0	0/0	0/0	0/0	0/0	0/0	0/0	0/0
0/0	0/0	./.	1/1	1/1	0/0	1/1	1/1	1/1	0/0	1/1	0/0	1/1	0/0	1/1	1/1	1/1	0/0	0/0	0/0	0/0	1/1	1/1	1/1	0/0	0/0	0/0
0/0	0/0	./.	0/0	0/0	0/0	0/0	0/0	0/0	0/0	0/0	1/1	0/0	0/0	0/0	0/0	0/0	0/0	0/0	0/0	0/0	0/0	0/0	0/0	0/0	0/0	0/0
0/0	1/1	./.	1/1	1/1	1/1	1/1	1/1	1/1	0/0	1/1	1/1	1/1	1/1	1/1	1/1	1/1	1/1	1/1	0/0	1/1	1/1	1/1	1/1	1/1	1/1	1/1
0/0	0/0	./.	1/1	1/1	0/0	1/1	1/1	1/1	0/0	1/1	0/0	1/1	0/0	1/1	1/1	1/1	0/0	0/0	0/0	0/0	1/1	1/1	1/1	0/0	0/0	0/0
0/0	0/0	./.	1/1	1/1	0/0	1/1	1/1	1/1	0/0	1/1	0/0	1/1	0/0	1/1	1/1	1/1	0/0	0/0	0/0	0/0	1/1	1/1	1/1	0/0	0/0	0/0
0/0	1/1	./.	0/0	0/0	1/1	0/0	0/0	0/0	0/0	0/0	1/1	0/0	1/1	0/0	0/0	0/0	1/1	1/1	0/0	1/1	0/0	0/0	0/0	1/1	1/1	1/1
0/0	1/1	./.	1/1	1/1	1/1	1/1	1/1	1/1	0/0	1/1	1/1	1/1	1/1	1/1	1/1	1/1	1/1	1/1	0/0	1/1	1/1	1/1	1/1	1/1	1/1	1/1
0/0	1/1	./.	0/0	0/0	1/1	0/0	0/0	0/0	0/0	0/0	1/1	0/0	1/1	0/0	0/0	0/0	1/1	1/1	0/0	1/1	0/0	0/0	0/0	1/1	1/1	1/1
0/0	0/0	./.	0/0	0/0	0/0	0/0	0/0	0/0	0/0	0/0	0/0	0/0	0/0	0/0	0/0	0/0	0/0	0/0	0/0	0/0	0/0	0/0	0/0	0/0	0/0	0/0
0/0	0/0	./.	0/0	0/0	0/0	0/0	0/0	0/0	0/0	0/0	1/1	0/0	0/0	0/0	0/0	0/0	0/0	0/0	0/0	0/0	0/0	0/0	0/0	0/0	0/0	0/0
1/1	1/1	./.	1/1	1/1	1/1	1/1	1/1	1/1	0/0	1/1	1/1	1/1	1/1	1/1	1/1	1/1	1/1	1/1	0/0	1/1	1/1	1/1	1/1	1/1	1/1	1/1
0/0	0/0	./.	0/0	0/0	0/0	0/0	0/0	0/0	0/0	./.	0/0	0/0	0/0	0/0	0/0	0/0	0/0	0/0	0/0	0/0	0/0	./.	0/0	0/0	0/0	0/0
0/0	1/1	./.	./.	1/1	1/1	1/1	1/1	1/1	0/0	./.	1/1	1/1	1/1	./.	1/1	1/1	1/1	1/1	0/0	1/1	1/1	1/1	1/1	1/1	1/1	1/1
0/0	1/1	./.	./.	0/0	1/1	0/0	0/0	0/0	0/0	./.	0/0	0/0	1/1	0/0	0/0	0/0	1/1	1/1	0/0	1/1	0/0	0/0	0/0	1/1	1/1	1/1
0/0	1/1	./.	./.	1/1	1/1	1/1	1/1	1/1	0/0	./.	1/1	1/1	1/1	./.	1/1	1/1	1/1	1/1	0/0	1/1	1/1	1/1	1/1	1/1	1/1	1/1
0/0	0/0	./.	./.	0/0	0/0	0/0	0/0	0/0	0/0	./.	0/0	0/0	0/0	0/0	0/0	0/0	0/0	0/0	0/0	0/0	0/0	0/0	0/0	0/0	0/0	0/0
0/0	1/1	./.	./.	0/0	1/1	0/0	0/0	0/0	0/0	./.	1/1	0/0	1/1	0/0	0/0	0/0	1/1	1/1	0/0	1/1	0/0	0/0	0/0	1/1	1/1	1/1

0/0	0/0	./.	./.	1/1	0/0	1/1	1/1	1/1	0/0	./.	0/0	1/1	0/0	./.	1/1	1/1	0/0	0/0	0/0	0/0	1/1	1/1	1/1	0/0	0/0	0/0
0/0	0/0	./.	./.	0/0	0/0	0/0	0/0	0/0	0/0	0/0	0/0	0/0	0/0	0/0	0/0	0/0	0/0	0/0	0/0	1/1	0/0	0/0	0/0	0/0	0/0	0/0
0/0	0/0	./.	./.	1/1	0/0	1/1	1/1	1/1	0/0	./.	0/0	1/1	0/0	./.	1/1	0/0	0/0	0/0	0/0	0/0	0/0	0/0	0/0	0/0	0/0	0/0
0/0	1/1	./.	./.	1/1	1/1	1/1	1/1	1/1	0/0	./.	1/1	1/1	1/1	./.	1/1	1/1	1/1	1/1	0/0	1/1	1/1	1/1	1/1	1/1	1/1	1/1
0/0	0/0	./.	0/0	0/0	0/0	0/0	0/0	0/0	0/0	0/0	0/0	0/0	0/0	0/0	0/0	0/0	0/0	0/0	0/0	0/0	0/0	0/0	0/0	0/0	0/0	0/0
0/0	0/0	./.	1/1	1/1	0/0	1/1	1/1	1/1	0/0	1/1	0/0	1/1	0/0	1/1	1/1	1/1	0/0	0/0	0/0	0/0	1/1	1/1	1/1	0/0	0/0	0/0
0/0	1/1	./.	1/1	1/1	1/1	1/1	1/1	1/1	0/0	1/1	1/1	1/1	1/1	1/1	1/1	1/1	1/1	1/1	0/0	1/1	1/1	1/1	1/1	1/1	1/1	1/1
0/0	1/1	./.	./.	0/0	1/1	./.	0/0	0/0	0/0	./.	0/0	./.	1/1	0/0	./.	0/0	1/1	1/1	0/0	1/1	0/0	0/0	./.	1/1	1/1	1/1
0/0	0/0	./.	1/1	1/1	0/0	1/1	1/1	1/1	0/0	1/1	0/0	1/1	0/0	1/1	1/1	1/1	0/0	0/0	0/0	0/0	1/1	1/1	1/1	0/0	0/0	0/0
0/0	0/0	./.	0/0	0/0	0/0	0/0	0/0	0/0	0/0	0/0	0/0	0/0	0/0	0/0	0/0	0/0	0/0	0/0	0/0	0/0	0/0	0/0	0/0	0/0	0/0	0/0
0/0	0/0	./.	1/1	1/1	0/0	1/1	1/1	1/1	0/0	1/1	0/0	1/1	0/0	1/1	1/1	1/1	0/0	0/0	0/0	0/0	1/1	1/1	1/1	0/0	0/0	0/0
0/0	0/0	./.	0/0	0/0	0/0	0/0	0/0	0/0	0/0	0/0	1/1	0/0	0/0	0/0	0/0	0/0	0/0	0/0	0/0	0/0	0/0	0/0	0/0	0/0	0/0	0/0
0/0	0/0	./.	0/0	0/0	0/0	0/0	0/0	0/0	0/0	0/0	1/1	0/0	0/0	0/0	0/0	0/0	0/0	0/0	0/0	0/0	0/0	0/0	0/0	0/0	0/0	0/0
0/0	0/0	./.	0/0	0/0	0/0	0/0	0/0	0/0	0/0	0/0	0/0	0/0	0/0	0/0	0/0	0/0	0/0	0/0	0/0	0/0	0/0	0/0	0/0	0/0	0/0	0/0
1/1	1/1	./.	1/1	1/1	1/1	1/1	1/1	1/1	0/0	1/1	1/1	1/1	1/1	1/1	1/1	1/1	1/1	1/1	0/0	1/1	1/1	1/1	1/1	1/1	1/1	1/1
1/1	1/1	./.	1/1	1/1	1/1	1/1	1/1	1/1	0/0	1/1	1/1	1/1	1/1	1/1	1/1	1/1	1/1	1/1	0/0	1/1	1/1	1/1	1/1	1/1	1/1	1/1
0/0	1/1	./.	1/1	1/1	1/1	1/1	1/1	1/1	0/0	1/1	1/1	1/1	1/1	1/1	1/1	1/1	1/1	1/1	0/0	1/1	1/1	1/1	1/1	1/1	1/1	1/1
./.	./.	./.	./.	./.	./.	./.	./.	./.	0/0	./.	./.	1/1	./.	./.	./.	./.	0/0	./.	0/0	./.	./.	./.	./.	./.	./.	./.
1/1	3/3	./.	1/1	1/1	3/3	1/1	1/1	1/1	0/0	1/1	3/3	4/4	3/3	1/1	1/1	1/1	3/3	3/3	0/0	3/3	1/1	1/1	1/1	3/3	3/3	3/3
0/0	0/0	./.	0/0	0/0	1/1	0/0	0/0	0/0	0/0	0/0	0/0	0/0	0/0	0/0	0/0	0/0	1/1	1/1	0/0	0/0	0/0	0/0	0/0	0/0	1/1	0/0
0/0	0/0	./.	0/0	0/0	0/0	0/0	0/0	0/0	0/0	0/0	0/0	0/0	0/0	0/0	0/0	0/0	0/0	0/0	0/0	0/0	0/0	0/0	0/0	0/0	0/0	0/0
0/0	0/0	./.	0/0	0/0	0/0	0/0	0/0	0/0	0/0	0/0	0/0	0/0	0/0	0/0	0/0	0/0	0/0	0/0	0/0	0/0	0/0	0/0	0/0	0/0	0/0	0/0
0/0	0/0	./.	0/0	0/0	0/0	0/0	0/0	0/0	0/0	0/0	0/0	0/0	0/0	0/0	0/0	0/0	0/0	0/0	0/0	0/0	0/0	0/0	0/0	0/0	0/0	0/0
0/0	0/0	./.	0/0	0/0	0/0	0/0	0/0	0/0	0/0	0/0	1/1	0/0	0/0	0/0	0/0	0/0	0/0	0/0	0/0	0/0	0/0	0/0	0/0	0/0	0/0	0/0
0/0	1/1	./.	0/0	0/0	1/1	0/0	0/0	0/0	0/0	0/0	0/0	0/0	1/1	0/0	0/0	0/0	1/1	1/1	0/0	1/1	0/0	0/0	0/0	1/1	1/1	0/0

0/0	0/0	./.	0/0	0/0	0/0	0/0	0/0	0/0	0/0	0/0	0/0	0/0	0/0	0/0	0/0	0/0	0/0	0/0	0/0	0/0	0/0	0/0	0/0	0/0	0/0	1/1
0/0	1/1	./.	0/0	0/0	1/1	0/0	0/0	0/0	0/0	0/0	0/0	0/0	1/1	0/0	0/0	0/0	1/1	1/1	0/0	1/1	0/0	0/0	0/0	1/1	1/1	1/1
0/0	1/1	./.	0/0	0/0	1/1	0/0	0/0	0/0	0/0	0/0	0/0	0/0	1/1	0/0	0/0	0/0	1/1	1/1	0/0	1/1	0/0	0/0	0/0	1/1	1/1	1/1
1/1	1/1	./.	./.	1/1	1/1	./.	1/1	1/1	0/0	1/1	1/1	1/1	1/1	1/1	1/1	1/1	1/1	1/1	0/0	./.	1/1	1/1	1/1	1/1	1/1	1/1
0/0	1/1	./.	1/1	1/1	1/1	1/1	1/1	1/1	0/0	1/1	1/1	1/1	1/1	1/1	1/1	1/1	1/1	1/1	0/0	0/1	1/1	1/1	1/1	1/1	1/1	1/1
0/0	0/0	./.	0/0	0/0	0/0	0/0	0/0	0/0	0/0	0/0	0/0	0/0	0/0	0/0	0/0	0/0	0/0	0/0	0/0	0/0	0/0	0/0	0/0	0/0	0/0	1/1
0/0	1/1	./.	1/1	1/1	1/1	1/1	1/1	1/1	0/0	1/1	1/1	1/1	1/1	1/1	1/1	1/1	1/1	1/1	0/0	0/1	1/1	1/1	1/1	1/1	1/1	1/1
0/0	1/1	./.	0/0	0/0	1/1	0/0	0/0	0/0	0/0	0/0	0/0	0/0	1/1	0/0	0/0	0/0	1/1	1/1	0/0	0/1	0/0	0/0	0/0	1/1	1/1	1/1
0/0	0/0	./.	0/0	0/0	0/0	0/0	0/0	0/0	0/0	0/0	1/1	0/0	0/0	0/0	0/0	0/0	0/0	0/0	0/0	0/0	0/0	0/0	0/0	0/0	0/0	0/0
0/0	0/0	./.	0/0	0/0	0/0	0/0	0/0	0/0	0/0	0/0	0/0	0/0	0/0	0/0	0/0	0/0	0/0	0/0	0/0	0/0	0/0	0/0	0/0	0/0	0/0	0/0
0/0	1/1	./.	0/0	0/0	1/1	0/0	0/0	0/0	0/0	0/0	0/0	0/0	1/1	0/0	0/0	0/0	1/1	1/1	0/0	1/1	0/0	0/0	0/0	1/1	1/1	1/1
0/0	0/0	./.	0/0	0/0	0/0	0/0	0/0	0/0	0/0	0/0	0/0	0/0	0/0	0/0	0/0	0/0	0/0	0/0	0/0	0/0	0/0	0/0	0/0	0/0	0/0	0/0
0/0	0/0	./.	1/1	1/1	0/0	1/1	1/1	1/1	0/0	1/1	0/0	1/1	0/0	1/1	1/1	1/1	0/0	0/0	0/0	0/0	1/1	1/1	1/1	0/0	0/0	0/0
0/0	1/1	./.	0/0	0/0	1/1	0/0	0/0	0/0	0/0	0/0	0/0	0/0	1/1	0/0	0/0	0/0	1/1	1/1	0/0	1/1	0/0	0/0	0/0	1/1	1/1	1/1
0/0	0/0	./.	0/0	0/0	0/0	0/0	0/0	0/0	0/0	0/0	0/0	0/0	0/0	0/0	0/0	0/0	0/0	0/0	0/0	0/0	0/0	0/0	0/0	0/0	0/0	0/0
0/0	1/1	./.	1/1	1/1	1/1	1/1	1/1	1/1	0/0	1/1	1/1	1/1	1/1	1/1	1/1	1/1	1/1	1/1	0/0	1/1	1/1	1/1	1/1	1/1	1/1	1/1
0/0	0/0	./.	1/1	1/1	0/0	1/1	1/1	1/1	0/0	1/1	0/0	1/1	0/0	1/1	1/1	1/1	0/0	0/0	0/0	0/0	1/1	1/1	1/1	0/0	0/0	0/0
0/0	0/0	./.	0/0	0/0	0/0	0/0	0/0	0/0	0/0	0/0	0/0	0/0	0/0	0/0	0/0	1/1	0/0	0/0	0/0	0/0	1/1	1/1	1/1	0/0	0/0	0/0
0/0	0/0	./.	0/0	0/0	0/0	0/0	0/0	0/0	0/0	0/0	0/0	0/0	0/0	0/0	0/0	0/0	0/0	0/0	0/0	0/0	0/0	0/0	0/0	0/0	0/0	0/0
0/0	1/1	./.	0/0	0/0	1/1	0/0	0/0	0/0	0/0	0/0	0/0	0/0	1/1	0/0	0/0	0/0	1/1	1/1	0/0	1/1	0/0	0/0	0/0	1/1	1/1	1/1
0/0	1/1	./.	0/0	0/0	1/1	0/0	0/0	0/0	0/0	0/0	0/0	0/0	1/1	0/0	0/0	0/0	1/1	1/1	0/0	1/1	0/0	0/0	0/0	1/1	1/1	1/1
0/0	0/0	./.	0/0	0/0	0/0	0/0	0/0	0/0	0/0	0/0	0/0	0/0	0/0	0/0	0/0	0/0	0/0	0/0	0/0	0/0	0/0	0/0	0/0	0/0	0/0	0/0
0/0	1/1	./.	1/1	1/1	1/1	1/1	1/1	1/1	0/0	1/1	1/1	1/1	1/1	1/1	1/1	1/1	1/1	1/1	0/0	1/1	1/1	1/1	1/1	1/1	1/1	1/1
0/0	0/0	./.	0/0	0/0	0/0	0/1	0/0	0/0	0/0	0/0	0/0	0/0	0/0	0/0	0/0	0/0	0/0	0/0	0/0	0/0	0/0	0/0	0/0	0/0	0/0	0/0
0/0	0/0	./.	0/0	0/0	0/0	0/0	0/0	0/0	0/0	0/0	1/1	0/0	0/0	0/0	0/0	0/0	0/0	0/0	0/0	0/0	0/0	0/0	0/0	0/0	0/0	0/0
0/0	1/1	./.	0/0	0/0	1/1	0/0	0/0	0/0	0/0	0/0	0/0	0/0	1/1	0/0	0/0	0/0	1/1	1/1	0/0	1/1	0/0	0/0	0/0	1/1	1/1	1/1
0/0	0/0	./.	0/0	0/0	0/0	0/0	0/0	0/0	0/0	0/0	0/0	0/0	0/0	0/0	0/0	0/0	0/0	0/0	0/0	0/0	0/0	0/0	0/0	0/0	0/0	0/0
0/0	0/0	./.	1/1	1/1	0/0	1/1	1/1	1/1	0/0	1/1	0/0	1/1	0/0	1/1	1/1	1/1	0/0	0/0	0/0	0/0	1/1	1/1	1/1	0/0	0/0	0/0
0/0	1/1	./.	0/0	0/0	1/1	0/0	0/0	0/0	0/0	0/0	0/0	0/0	1/1	0/0	0/0	0/0	1/1	1/1	0/0	1/1	0/0	0/0	0/0	1/1	1/1	1/1
0/0	0/0	./.	0/0	0/0	0/0	0/0	0/0	0/0	0/0	0/0	0/0	0/0	0/0	0/0	0/0	0/0	0/0	0/0	0/0	0/0	0/0	0/0	0/0	0/0	0/0	0/0
0/0	1/1	./.	1/1	1/1	1/1	1/1	1/1	1/1	0/0	1/1	1/1	1/1	1/1	1/1	1/1	1/1	1/1	1/1	0/0	1/1	1/1	1/1	1/1	1/1	1/1	1/1
0/0	0/0	./.	0/0	0/0	0/0	0/0	0/0	0/0	0/0	0/0	0/0	0/0	0/0	0/0	0/0	0/0	0/0	0/0	0/0	0/0	0/0	0/0	0/0	0/0	0/0	0/0
1/1	0/0	./.	0/0	0/0	0/0	0/0	0/0	0/0	0/0	0/0	0/0	0/0	0/0	0/0	0/0	0/0	0/0	0/0	0/0	0/0	0/0	0/0	0/0	0/0	0/0	0/0

0/0	1/1	./.	1/1	1/1	1/1	1/1	1/1	1/1	0/0	1/1	1/1	1/1	1/1	1/1	1/1	1/1	1/1	1/1	0/0	1/1	1/1	1/1	1/1	1/1	1/1	1/1
0/0	0/0	./.	0/0	0/0	0/0	0/0	0/0	0/0	0/0	0/0	0/0	0/0	0/0	0/0	0/0	0/0	0/0	0/0	0/0	0/0	0/0	0/0	0/0	0/0	0/0	0/0
0/0	1/1	./.	1/1	1/1	1/1	1/1	1/1	1/1	0/0	1/1	1/1	1/1	1/1	1/1	1/1	1/1	1/1	1/1	0/0	1/1	1/1	1/1	1/1	1/1	1/1	1/1
0/0	0/0	./.	0/0	0/0	0/0	0/0	0/0	0/0	0/0	1/1	0/0	0/0	0/0	0/0	0/0	0/0	0/0	0/0	0/0	0/0	0/0	0/0	0/0	0/0	0/0	0/0
0/0	0/0	./.	1/1	1/1	0/0	1/1	1/1	1/1	0/0	1/1	0/0	1/1	0/0	1/1	1/1	0/0	0/0	0/0	0/0	0/0	0/0	0/0	0/0	0/0	0/0	0/0
0/0	0/0	./.	0/0	0/0	0/0	0/0	0/0	0/0	0/0	0/0	0/0	0/0	0/0	0/0	0/0	0/0	0/0	0/0	0/0	0/0	0/0	0/0	0/0	0/0	0/0	0/0
0/0	0/0	./.	0/0	0/0	0/0	0/0	0/0	0/0	0/0	0/0	1/1	0/0	0/0	0/0	0/0	0/0	0/0	0/0	0/0	0/0	0/0	0/0	0/0	0/0	0/0	0/0
0/0	0/0	./.	0/0	0/0	0/0	0/0	0/0	0/0	0/0	0/0	0/0	0/0	0/0	0/0	0/0	1/1	0/0	0/0	0/0	0/0	1/1	1/1	1/1	0/0	0/0	0/0
0/0	0/0	./.	0/0	0/0	0/0	0/0	0/0	0/0	0/0	0/0	0/0	0/0	0/0	0/0	0/0	0/0	0/0	0/0	0/0	0/0	0/0	0/0	0/0	0/0	0/0	0/0
0/0	0/0	./.	0/0	0/0	0/0	0/0	0/0	0/0	0/0	0/0	1/1	0/0	0/0	0/0	0/0	0/0	0/0	0/0	0/0	0/0	0/0	0/0	0/0	0/0	0/0	0/0
0/0	1/1	./.	1/1	1/1	1/1	1/1	1/1	1/1	0/0	1/1	1/1	1/1	1/1	1/1	1/1	1/1	1/1	1/1	0/0	1/1	1/1	1/1	1/1	1/1	1/1	1/1
0/0	1/1	./.	1/1	1/1	1/1	1/1	1/1	1/1	0/0	1/1	1/1	1/1	1/1	1/1	1/1	1/1	1/1	1/1	0/0	1/1	1/1	1/1	1/1	1/1	1/1	1/1
0/0	0/0	./.	0/0	0/0	0/0	0/0	0/0	0/0	0/0	0/0	1/1	0/0	0/0	0/0	0/0	0/0	0/0	0/0	0/0	0/0	0/0	0/0	0/0	0/0	0/0	0/0
0/0	0/0	./.	1/1	1/1	0/0	1/1	1/1	1/1	0/0	1/1	0/0	1/1	0/0	1/1	1/1	0/0	0/0	0/0	0/0	0/0	0/0	0/0	0/0	0/0	0/0	0/0
0/0	1/1	./.	0/0	0/0	1/1	0/0	0/0	0/0	0/0	0/0	0/0	./.	1/1	0/0	0/0	0/0	1/1	1/1	0/0	1/1	0/0	0/0	0/0	1/1	1/1	1/1
2/2	1/1	./.	1/1	1/1	1/1	1/1	1/2	1/1	0/0	1/1	0/0	1/1	1/1	1/1	1/1	1/1	1/1	1/1	0/0	1/1	1/1	1/1	1/1	1/1	1/1	0/1
0/0	1/1	./.	1/1	1/1	1/1	1/1	1/1	1/1	0/0	1/1	1/1	1/1	1/1	1/1	1/1	1/1	1/1	1/1	0/0	1/1	1/1	1/1	1/1	1/1	1/1	1/1
0/0	1/1	0/0	0/0	0/0	1/1	0/0	0/0	0/0	0/0	0/0	0/0	0/0	1/1	1/1	0/0	0/0	1/1	1/1	0/0	1/1	0/0	0/0	0/0	1/1	1/1	1/1
0/0	1/1	0/0	0/0	0/0	1/1	0/0	0/0	0/0	0/0	0/0	0/0	0/0	1/1	1/1	0/0	0/0	1/1	1/1	0/0	1/1	0/0	0/0	0/0	1/1	1/1	1/1
0/0	0/0	0/0	1/1	1/1	0/0	1/1	1/1	1/1	0/0	0/0	0/0	1/1	0/0	0/0	1/1	0/0	0/0	0/0	0/0	0/0	0/0	0/0	0/0	0/0	0/0	0/0
0/0	0/0	0/0	0/0	0/0	0/0	0/0	0/0	0/0	0/0	0/0	0/0	0/0	0/0	0/0	0/0	0/0	0/0	0/0	0/0	0/0	0/0	0/0	0/0	0/0	0/0	0/0
0/0	0/0	1/1	0/0	0/0	0/0	0/0	0/0	0/0	0/0	0/0	0/0	0/0	0/0	0/0	0/0	0/0	0/0	0/0	0/0	0/0	0/0	0/0	0/0	0/0	0/0	0/0
0/0	0/1	0/0	0/0	0/0	0/1	0/0	0/0	0/0	0/0	0/0	0/0	0/0	./.	0/0	0/0	0/0	0/0	0/0	0/0	0/1	0/0	0/0	0/0	0/0	0/0	0/0
0/0	0/0	1/1	0/0	0/0	0/0	0/0	0/0	0/0	0/0	0/0	0/0	0/0	0/0	0/0	0/0	0/0	0/0	0/0	0/0	0/0	0/0	0/0	0/0	0/0	0/0	0/0
0/0	0/1	0/0	0/0	0/0	0/1	0/0	0/0	0/0	0/0	0/0	0/0	0/1	0/0	0/0	0/1	0/0	0/1	0/0	0/0	0/1	0/0	0/0	0/1	0/0	0/0	0/0
0/0	0/1	0/0	0/0	0/0	0/1	0/0	0/0	0/0	0/0	./.	0/0	0/1	0/0	0/0	0/1	0/0	0/1	0/0	0/0	0/1	0/0	0/0	0/1	./.	0/0	0/0

0/0	0/1	0/0	0/0	0/0	0/1	0/0	0/0	0/0	0/0	0/0	0/0	0/1	0/0	0/0	0/0	0/0	0/0	0/0	0/0	0/1	0/0	0/0	0/1	0/0	0/0	0/0
0/0	0/1	0/0	0/0	0/0	0/1	0/0	0/0	0/0	0/0	./.	0/0	0/1	0/0	./.	0/1	0/0	0/0	0/0	0/0	0/1	0/0	0/0	0/1	./.	0/0	0/0
0/0	0/1	0/0	0/0	0/0	0/1	0/0	0/0	0/0	0/0	0/0	0/0	0/1	0/0	./.	0/1	0/0	0/0	0/0	0/0	0/1	0/0	0/0	0/1	0/0	0/0	0/0
0/0	0/1	0/0	0/0	0/0	0/1	0/0	0/0	0/0	0/0	0/0	0/0	0/1	0/0	./.	0/1	0/0	0/0	0/0	0/0	0/1	0/0	0/0	0/1	0/0	0/0	0/0
0/0	0/1	0/0	0/0	0/0	0/1	0/0	0/0	0/0	0/0	0/0	0/0	0/1	0/0	./.	0/1	0/0	0/0	0/0	0/0	0/1	0/0	0/0	0/1	0/0	0/0	0/0
0/0	0/1	0/0	0/0	0/0	0/1	0/0	0/0	0/0	0/0	0/0	0/0	0/0	0/0	0/0	0/0	0/0	0/0	0/0	0/0	0/1	0/0	0/0	0/1	0/0	0/0	0/0
0/0	0/0	0/0	0/0	0/0	0/0	0/0	0/0	0/0	0/0	0/0	0/0	0/0	0/0	0/0	0/1	0/0	0/0	0/0	0/0	0/0	0/0	0/0	0/0	0/0	0/0	0/0
0/0	0/0	0/0	0/0	0/0	0/0	0/0	0/0	0/0	0/0	0/0	0/0	0/0	0/0	0/0	0/2	0/0	0/0	0/0	0/0	0/0	0/0	0/0	0/1	0/0	0/0	0/0
0/0	0/0	0/0	0/0	0/0	0/0	0/0	0/0	0/0	0/0	0/0	0/0	0/0	0/0	0/0	0/2	0/0	0/0	0/0	0/0	0/0	0/0	0/0	0/1	0/0	0/0	0/0
0/0	0/0	0/0	0/0	0/0	0/0	0/0	1/1	0/0	0/0	0/0	0/0	0/0	0/0	0/0	0/0	0/0	0/0	0/0	0/0	0/0	0/0	0/0	0/0	0/0	0/0	0/0
0/0	1/1	1/1	1/1	1/1	1/1	1/1	1/1	1/1	0/0	1/1	1/1	1/1	1/1	1/1	1/1	1/1	1/1	1/1	0/0	1/1	1/1	1/1	1/1	1/1	1/1	1/1
0/0	0/0	1/1	0/0	0/0	0/0	0/0	0/0	0/0	0/0	0/0	0/0	0/0	0/0	0/0	0/0	0/0	0/0	0/0	0/0	0/0	0/0	0/0	0/0	0/0	0/0	0/0
0/0	0/0	0/0	0/0	0/0	0/0	0/0	0/0	0/0	0/0	1/1	0/0	0/0	0/0	0/0	0/0	0/0	0/0	0/0	0/0	0/0	0/0	0/0	0/0	0/0	0/0	0/0
0/0	0/0	0/0	0/0	0/0	0/0	0/0	0/0	0/0	0/0	0/0	1/1	0/0	0/0	0/0	0/0	1/1	0/0	0/0	0/0	0/0	1/1	1/1	1/1	0/0	0/0	0/0
0/0	0/0	1/1	0/0	0/0	0/0	0/0	0/0	0/0	0/0	0/0	0/0	0/0	0/0	0/0	0/0	0/0	0/0	0/0	0/0	0/0	0/0	0/0	0/0	0/0	0/0	0/0
0/0	0/0	0/0	0/0	0/0	0/0	0/0	0/0	0/0	0/0	0/0	0/0	0/0	0/0	0/0	0/0	0/0	0/0	0/0	0/0	0/0	0/0	0/0	0/0	0/0	0/0	0/0
0/0	1/1	1/1	1/1	1/1	1/1	1/1	1/1	1/1	0/0	1/1	1/1	1/1	1/1	1/1	1/1	1/1	1/1	1/1	0/0	1/1	1/1	1/1	1/1	1/1	1/1	1/1
0/0	./.	0/0	./.	0/0	0/0	0/0	0/0	0/0	0/0	1/1	0/0	0/0	./.	0/0	0/0	0/0	./.	./.	0/0	0/0	0/0	./.	0/0	0/0	./.	./.
0/0	./.	1/1	1/1	1/1	0/0	1/1	1/1	1/1	0/0	1/1	1/1	1/1	./.	0/0	1/1	1/1	./.	./.	0/0	0/0	1/1	1/1	1/1	./.	./.	./.
0/0	1/1	0/0	0/0	0/0	1/1	0/0	0/0	0/0	0/0	0/0	0/0	0/0	1/1	1/1	0/0	0/0	1/1	1/1	0/0	1/1	0/0	0/0	0/0	1/1	1/1	1/1
0/0	1/1	0/0	0/0	0/0	1/1	0/0	0/0	0/0	0/0	0/0	0/0	0/0	1/1	1/1	0/0	0/0	1/1	1/1	0/0	1/1	0/0	0/0	0/0	1/1	1/1	1/1
0/0	0/0	2/2	./.	./.	0/0	0/0	0/0	0/0	0/0	./.	0/0	./.	0/0	0/0	./.	./.	0/0	0/0	0/0	0/0	0/0	./.	./.	0/0	0/0	0/0
0/0	0/0	2/2	./.	0/0	0/0	./.	0/0	0/0	0/0	0/1	./.	0/0	0/0	0/0	0/0	./.	0/0	0/0	0/0	0/0	1/1	./.	1/1	0/0	0/0	0/0
0/0	0/0	2/2	1/1	0/1	0/0	1/1	1/1	1/1	0/0	0/1	1/1	1/1	0/0	0/0	1/1	1/1	0/0	0/0	0/0	0/0	2/2	1/1	0/0	0/0	0/0	0/0

0/0	0/0	2/2	2/2	0/1	0/0	2/2	0/0	2/2	0/0	0/2	2/2	0/0	0/0	0/0	0/0	0/0	0/0	0/0	0/0	0/0	2/2	2/2	2/2	0/0	0/0	0/0
0/0	0/0	4/4	4/4	0/4	0/0	4/4	0/0	4/4	0/0	0/4	4/4	0/0	0/0	0/0	0/0	4/4	0/0	0/0	0/0	0/0	4/4	4/4	4/4	0/0	0/0	0/0
0/0	0/0	0/0	0/0	0/0	0/0	0/0	0/0	0/0	0/0	1/1	0/0	0/0	0/0	0/0	0/0	0/0	0/0	0/0	0/0	0/0	0/0	0/0	0/0	0/0	0/0	0/0
0/0	1/1	1/1	1/1	1/1	1/1	1/1	1/1	1/1	0/0	1/1	1/1	1/1	1/1	1/1	1/1	1/1	1/1	1/1	0/0	1/1	1/1	1/1	1/1	1/1	1/1	1/1
0/0	0/0	0/0	1/1	1/1	0/0	1/1	1/1	1/1	0/0	1/1	1/1	1/1	0/0	0/0	1/1	1/1	0/0	0/0	0/0	0/0	1/1	1/1	1/1	0/0	0/0	0/0
0/0	0/0	1/1	0/0	0/0	0/0	0/0	0/0	0/0	0/0	0/0	0/0	0/0	0/0	0/0	0/0	0/0	0/0	0/0	0/0	0/0	0/0	0/0	0/0	0/0	0/0	0/0
0/0	0/0	1/1	0/0	0/0	0/0	0/0	0/0	0/0	0/0	0/0	0/0	0/0	0/0	0/0	0/0	0/0	0/0	0/0	0/0	0/0	0/0	0/0	0/0	0/0	0/0	0/0
0/0	1/1	1/1	1/1	1/1	1/1	1/1	1/1	1/1	0/0	1/1	1/1	1/1	1/1	1/1	1/1	1/1	1/1	1/1	0/0	1/1	1/1	1/1	1/1	1/1	1/1	1/1
0/0	0/0	0/0	0/0	0/0	0/0	./.	0/0	./.	0/0	0/0	./.	./.	0/0	0/0	0/0	./.	0/0	0/0	0/0	0/0	0/0	0/0	0/0	0/0	0/0	0/0
0/0	0/0	0/0	1/1	1/1	0/0	1/1	1/1	1/1	0/0	2/2	1/1	1/1	0/0	0/0	1/1	1/1	0/0	0/0	0/0	0/0	1/1	1/2	1/1	0/0	0/0	0/0
0/0	0/0	0/0	1/1	1/1	0/0	1/1	1/1	1/1	0/0	1/1	1/1	1/1	0/0	0/0	1/1	1/1	0/0	0/0	0/0	0/0	1/1	1/1	1/1	0/0	0/0	0/0
0/0	0/0	0/0	1/1	1/1	0/0	1/1	1/1	1/1	0/0	0/0	1/1	1/1	0/0	0/0	1/1	1/1	0/0	0/0	0/0	0/0	1/1	1/1	1/1	0/0	0/0	0/0
0/0	1/1	0/0	0/0	0/0	1/1	0/0	0/0	0/0	0/0	0/0	0/0	0/0	1/1	1/1	0/0	0/0	1/1	1/1	0/0	1/1	0/0	0/0	0/0	1/1	1/1	1/1
0/0	0/0	0/0	1/1	1/1	0/0	1/1	1/1	1/1	0/0	1/1	1/1	1/1	0/0	0/0	1/1	1/1	0/0	0/0	0/0	0/0	1/1	1/1	1/1	0/0	0/0	0/0
0/0	1/1	1/1	1/1	1/1	1/1	1/1	1/1	1/1	0/0	1/1	1/1	1/1	1/1	1/1	1/1	1/1	1/1	1/1	0/0	1/1	1/1	1/1	1/1	1/1	1/1	1/1
0/0	0/0	0/0	0/0	0/0	0/0	0/0	0/0	0/0	0/0	0/0	0/0	0/0	0/0	0/0	0/0	0/0	0/0	0/0	0/0	0/0	0/0	0/0	0/0	0/0	0/0	0/0
0/0	0/0	0/0	0/0	0/0	0/0	0/0	0/0	0/0	0/0	0/0	0/0	0/0	0/0	0/0	0/0	0/0	0/0	0/0	0/0	0/0	0/0	0/0	0/0	0/0	0/0	0/0
0/0	1/1	./.	0/0	0/0	1/1	0/0	0/0	0/0	0/0	0/0	0/0	0/0	1/1	1/1	0/0	0/0	1/1	1/1	0/0	1/1	0/0	0/0	0/0	1/1	1/1	1/1
0/0	0/0	1/1	0/0	0/0	0/0	0/0	0/0	0/0	0/0	0/0	0/0	0/0	0/0	0/0	0/0	0/0	0/0	0/0	0/0	0/0	0/0	0/0	0/0	0/0	0/0	0/0
0/0	1/1	0/0	0/0	0/0	1/1	0/0	0/0	0/0	0/0	0/0	0/0	0/0	1/1	1/1	0/0	0/0	1/1	1/1	0/0	1/1	0/0	0/0	0/0	1/1	1/1	1/1
0/0	0/0	0/0	0/0	0/0	0/0	0/0	0/0	0/0	0/0	0/0	1/1	0/0	0/0	0/0	0/0	1/1	0/0	0/0	0/0	0/0	1/1	1/1	1/1	0/0	0/0	0/0
0/0	1/1	0/0	0/0	0/0	1/1	0/0	0/0	0/0	0/0	0/0	0/0	0/0	1/1	1/1	0/0	0/0	1/1	1/1	0/0	1/1	0/0	0/0	0/0	1/1	1/1	1/1
0/0	1/1	0/0	0/0	0/0	1/1	0/0	0/0	0/0	0/0	0/0	0/0	0/0	1/1	1/1	0/0	0/0	1/1	1/1	0/0	1/1	0/0	0/0	0/0	1/1	1/1	1/1
0/0	0/0	0/0	0/0	0/0	0/0	0/0	0/0	0/0	0/0	1/1	0/0	0/0	0/0	0/0	0/0	0/0	0/0	0/0	0/0	0/0	0/0	0/0	0/0	0/0	0/0	0/0
0/0	0/0	0/0	0/0	0/0	0/0	0/0	0/0	0/0	0/0	1/1	0/0	0/0	0/0	0/0	0/0	0/0	0/0	0/0	0/0	0/0	0/0	0/0	0/0	0/0	0/0	0/0
0/0	0/0	0/0	1/1	1/1	0/0	1/1	1/1	1/1	0/0	1/1	1/1	1/1	0/0	0/0	1/1	1/1	0/0	0/0	0/0	0/0	1/1	1/1	1/1	0/0	0/0	0/0
0/0	0/0	0/0	0/0	0/0	0/0	0/0	0/0	0/0	0/0	0/0	0/0	0/0	0/0	0/0	0/0	0/0	0/0	0/0	0/0	0/0	0/0	0/0	0/0	0/0	0/0	0/0
0/0	1/1	1/1	1/1	1/1	1/1	1/1	1/1	1/1	0/0	1/1	1/1	1/1	1/1	1/1	1/1	1/1	1/1	1/1	0/0	1/1	1/1	1/1	1/1	1/1	1/1	1/1
0/0	0/0	0/0	0/0	0/0	0/0	0/0	0/0	0/0	0/0	0/0	0/0	0/0	0/0	0/0	0/0	0/0	0/0	0/0	0/0	0/0	0/0	0/0	0/0	0/0	0/0	0/0
0/0	1/1	1/1	1/1	1/1	1/1	1/1	1/1	1/1	0/0	1/1	1/1	1/1	1/1	1/1	1/1	1/1	1/1	1/1	0/0	1/1	1/1	1/1	1/1	1/1	1/1	1/1

0/0	0/0	0/0	0/0	0/0	0/0	0/0	0/0	0/0	0/0	1/1	0/0	0/0	0/0	0/0	0/0	0/0	0/0	0/0	0/0	0/0	0/0	0/0	0/0	0/0	0/0	0/0
0/0	0/0	0/0	0/0	0/0	0/0	0/0	0/0	0/0	0/0	0/0	0/0	0/0	0/0	0/0	0/0	0/0	0/0	0/0	0/0	0/0	0/0	0/0	0/0	0/0	0/0	0/0
0/0	0/0	0/0	0/0	0/0	1/1	0/0	0/0	0/0	0/0	0/0	0/0	0/0	0/0	0/0	0/0	0/0	0/0	0/0	0/0	0/0	0/0	0/0	0/0	0/0	0/0	0/0
0/0	0/0	0/0	1/1	1/1	0/0	1/1	1/1	1/1	0/0	0/0	0/0	1/1	0/0	0/0	1/1	0/0	0/0	0/0	0/0	0/0	0/0	0/0	0/0	0/0	0/0	0/0
0/0	0/0	0/0	1/1	1/1	0/0	1/1	1/1	1/1	0/0	1/1	1/1	1/1	0/0	0/0	1/1	1/1	0/0	0/0	0/0	0/0	1/1	1/1	1/1	0/0	0/0	0/0
0/0	1/1	0/0	1/1	1/1	1/1	1/1	1/1	1/1	0/0	1/1	1/1	1/1	1/1	1/1	1/1	1/1	1/1	1/1	0/0	1/1	1/1	1/1	1/1	1/1	1/1	1/1
0/0	0/0	0/0	1/1	1/1	0/0	1/1	1/1	1/1	0/0	1/1	1/1	1/1	0/0	0/0	1/1	1/1	0/0	0/0	0/0	0/0	1/1	1/1	1/1	0/0	0/0	0/0
0/0	0/0	0/0	0/0	0/0	0/0	0/0	0/0	0/0	0/0	0/0	1/1	0/0	0/0	0/0	0/0	1/1	0/0	0/0	0/0	0/0	1/1	1/1	1/1	0/0	0/0	0/0
0/0	0/0	0/0	0/0	0/0	0/0	0/0	0/0	0/0	0/0	0/0	0/0	0/0	0/0	0/0	0/0	0/0	0/0	0/0	0/0	0/0	0/0	0/0	0/0	0/0	0/0	0/0
0/0	0/0	0/0	0/0	0/0	0/0	0/0	0/0	0/0	0/0	0/0	0/0	0/0	0/0	0/0	0/0	0/0	0/0	0/0	0/0	0/0	0/0	0/0	0/0	0/0	0/0	0/0
0/0	0/0	0/0	0/0	0/0	0/0	0/0	0/0	0/0	0/0	0/0	0/0	0/0	0/0	0/0	0/0	0/0	0/0	0/0	0/0	0/0	0/0	0/0	0/0	0/0	0/0	0/0
0/0	1/1	0/0	0/0	0/0	1/1	0/0	0/0	0/0	0/0	0/0	0/0	0/0	1/1	1/1	0/0	0/0	1/1	1/1	0/0	1/1	0/0	0/0	0/0	1/1	1/1	1/1
0/0	1/1	0/0	1/1	1/1	1/1	1/1	1/1	1/1	0/0	2/2	0/0	1/1	1/1	1/1	0/1	0/0	1/1	1/1	0/0	0/1	0/0	0/0	0/0	1/1	1/1	1/1
0/0	1/1	1/1	1/1	1/1	1/1	1/1	1/1	1/1	0/0	1/1	1/1	1/1	1/1	1/1	1/1	1/1	1/1	1/1	0/0	1/1	1/1	1/1	1/1	1/1	1/1	1/1
1/1	1/1	1/1	1/1	1/1	1/1	1/1	1/1	1/1	1/1	1/1	1/1	1/1	1/1	1/1	1/1	1/1	1/1	1/1	0/0	1/1	1/1	1/1	1/1	1/1	1/1	1/1
0/0	0/0	0/0	0/0	0/0	0/0	0/0	0/0	0/0	0/0	0/0	0/0	0/0	0/0	0/0	0/0	0/0	0/0	0/0	0/0	0/0	0/0	0/0	0/0	0/0	0/0	0/0
0/0	0/0	0/0	1/1	1/1	0/0	1/1	1/1	1/1	0/0	0/0	0/0	1/1	0/0	0/0	1/1	0/0	0/0	0/0	0/0	0/0	0/0	0/0	0/0	0/0	0/0	0/0
0/0	0/0	1/1	1/1	1/1	0/0	1/1	1/1	1/1	0/0	1/1	1/1	1/1	0/0	0/0	1/1	1/1	0/0	0/0	0/0	0/0	1/1	1/1	1/1	0/0	0/0	0/0
0/0	1/1	1/1	1/1	1/1	1/1	1/1	1/1	1/1	0/0	1/1	1/1	1/1	1/1	1/1	1/1	1/1	1/1	1/1	0/0	1/1	1/1	1/1	1/1	1/1	1/1	1/1
0/0	0/0	0/0	0/0	0/0	0/0	0/0	0/0	0/0	0/0	0/0	0/0	0/0	0/0	0/0	0/0	0/0	0/0	0/0	0/0	0/0	0/0	0/0	0/0	0/0	0/0	0/0
0/0	0/0	1/1	0/0	0/0	0/0	0/0	0/0	0/0	0/0	0/0	0/0	0/0	0/0	0/0	0/0	0/0	0/0	0/0	0/0	0/0	0/0	0/0	0/0	0/0	0/0	0/0
0/0	0/0	0/0	0/0	0/0	0/0	0/0	0/0	0/0	0/0	1/1	0/0	0/0	0/0	0/0	0/0	0/0	0/0	0/0	0/0	0/0	0/0	0/0	0/0	0/0	0/0	0/0
0/0	1/1	1/1	1/1	1/1	1/1	1/1	1/1	1/1	0/0	1/1	1/1	1/1	1/1	1/1	1/1	1/1	1/1	1/1	0/0	1/1	1/1	1/1	1/1	1/1	1/1	1/1
0/0	0/0	0/0	0/0	0/0	0/0	0/0	0/0	0/0	0/0	0/0	0/0	0/0	0/0	0/0	0/0	0/0	0/0	0/0	0/0	0/0	0/0	0/0	0/0	0/0	0/0	0/0
0/0	0/0	0/0	0/0	0/0	0/0	0/0	0/0	0/0	0/0	1/1	0/0	0/0	0/0	0/0	0/0	0/0	0/0	0/0	0/0	0/0	0/0	0/0	0/0	0/0	0/0	0/0
0/0	0/0	1/1	0/0	0/0	0/0	0/0	0/0	0/0	0/0	0/0	0/0	0/0	0/0	0/0	0/0	0/0	0/0	0/0	0/0	0/0	0/0	0/0	0/0	0/0	0/0	0/0
0/0	0/0	0/0	0/0	0/0	0/0	0/0	0/0	0/0	0/0	0/0	0/0	0/0	0/0	0/0	0/0	0/0	0/0	0/0	0/0	0/0	0/0	0/0	0/0	0/0	0/0	0/0
0/0	0/0	1/1	0/0	0/0	0/0	0/0	0/0	0/0	0/0	0/0	0/0	0/0	0/0	0/0	0/0	0/0	0/0	0/0	0/0	0/0	0/0	0/0	0/0	0/0	0/0	0/0
0/0	0/0	1/1	0/0	0/0	0/0	0/0	0/0	0/0	0/0	0/0	0/0	0/0	0/0	0/0	0/0	0/0	0/0	0/0	0/0	0/0	0/0	0/0	0/0	0/0	0/0	0/0
0/0	0/0	0/0	0/0	0/0	0/0	0/0	0/0	0/0	0/0	0/0	0/0	0/0	0/0	0/0	0/0	0/0	0/0	0/0	0/0	0/0	0/0	0/0	0/0	0/0	0/0	0/0
0/0	0/0	0/0	0/0	0/0	0/0	0/0	0/0	0/0	0/0	0/0	0/0	0/0	0/0	0/0	0/0	0/0	0/0	0/0	0/0	0/0	0/0	0/0	0/0	0/0	0/0	0/0
0/0	1/1	0/0	0/0	0/0	1/1	0/0	0/0	0/0	0/0	0/0	0/0	0/0	1/1	1/1	0/0	0/0	1/1	1/1	0/0	0/0	0/0	0/0	0/0	1/1	1/1	1/1
0/0	0/0	1/1	0/0	0/0	0/0	0/0	0/0	0/0	0/0	0/0	0/0	0/0	0/0	0/0	0/0	0/0	0/0	0/0	0/0	0/0	0/0	0/0	0/0	0/0	0/0	0/0
0/0	0/0	0/0	0/0	0/0	0/0	0/0	0/0	0/0	0/0	0/0	0/0	0/0	0/0	0/0	0/0	0/0	0/0	0/0	0/0	0/0	0/0	0/0	0/0	0/0	0/0	0/0
0/0	0/0	0/0	1/1	1/1	0/0	1/1	1/1	1/1	0/0	0/0	0/0	0/0	0/0	0/0	1/1	0/0	0/0	0/0	0/0	0/0	0/0	0/0	0/0	0/0	0/0	0/0

0/0	0/0	0/0	0/0	0/0	0/0	0/0	0/0	0/0	0/0	0/0	1/1	0/0	0/0	0/0	0/0	1/1	0/0	0/0	0/0	0/0	1/1	1/1	1/1	0/0	0/0	0/0
3/3	1/1	1/1	2/2	2/2	2/2	2/3	2/2	2/3	4/4	1/4	4/4	2/3	1/2	2/2	2/2	4/4	2/2	2/2	0/0	1/1	1/4	4/4	4/4	1/1	2/2	1/1
./.	./.	./.	./.	./.	./.	./.	./.	./.	./.	./.	./.	./.	./.	./.	./.	./.	./.	./.	0/0	./.	./.	./.	./.	./.	./.	./.
0/0	0/0	1/1	0/0	0/0	0/0	0/0	0/0	0/0	0/0	0/0	0/0	0/0	0/0	0/0	0/0	0/0	0/0	0/0	0/0	0/0	0/0	0/0	0/0	0/0	0/0	0/0
0/0	0/0	0/0	0/0	0/0	0/0	0/0	0/0	0/0	0/0	0/0	0/0	0/0	0/0	0/0	0/0	0/0	0/0	0/0	0/0	0/0	0/0	0/0	0/0	0/0	0/0	0/0
0/0	0/0	0/0	0/0	0/0	0/0	0/0	0/0	0/0	0/0	1/1	0/0	0/0	0/0	0/0	0/0	0/0	0/0	0/0	0/0	0/0	0/0	0/0	0/0	0/0	0/0	0/0
0/0	0/0	0/0	0/0	0/0	0/0	0/0	0/0	0/0	0/0	0/0	1/1	0/0	0/0	0/0	0/0	1/1	0/0	0/0	0/0	0/0	1/1	1/1	1/1	0/0	0/0	0/0
0/0	0/0	0/0	0/0	0/0	0/0	0/0	0/0	0/0	0/0	0/0	1/1	0/0	0/0	0/0	0/0	1/1	0/0	./.	0/0	0/0	1/1	1/1	1/1	0/0	0/0	0/0
0/0	0/0	0/0	0/0	0/0	0/0	0/0	0/0	./.	0/0	1/1	0/0	0/0	./.	0/0	0/0	./.	0/0	./.	0/0	0/0	0/0	0/0	0/0	0/0	./.	0/0
./.	./.	0/0	1/1	1/1	0/0	1/1	1/1	1/1	0/0	1/1	1/1	1/1	0/0	./.	1/1	1/1	./.	0/0	0/0	1/1	1/1	1/1	1/1	0/0	./.	./.
1/1	2/2	4/4	./.	./.	2/2	./.	./.	./.	0/0	./.	./.	./.	2/3	2/2	./.	./.	2/2	2/2	0/0	./.	./.	./.	./.	2/2	2/2	2/2
2/2	./.	0/0	./.	0/0	./.	./.	0/0	0/0	0/0	./.	./.	0/0	./.	./.	0/0	./.	./.	./.	0/0	./.	0/0	0/0	0/0	./.	./.	./.
0/0	0/0	0/0	0/0	0/0	0/0	0/0	0/0	0/0	0/0	1/1	0/0	0/0	0/0	0/0	0/0	0/0	0/0	0/0	0/0	0/0	0/0	0/0	0/0	0/0	0/0	0/0
0/0	0/0	0/0	0/0	0/0	0/0	0/0	0/0	0/0	0/0	0/0	0/0	0/0	0/0	0/0	0/0	0/0	0/0	0/0	0/0	0/0	0/0	0/0	0/0	0/0	0/0	0/0
0/0	0/0	0/0	0/0	0/0	0/0	0/0	0/0	0/0	0/0	0/0	0/0	0/0	0/0	0/0	0/0	0/0	0/0	0/0	0/0	0/0	0/0	0/0	0/0	0/0	0/0	0/0
0/0	1/1	1/1	1/1	1/1	1/1	1/1	1/1	1/1	0/0	1/1	1/1	1/1	1/1	1/1	1/1	1/1	1/1	1/1	0/0	1/1	1/1	1/1	1/1	1/1	1/1	1/1
0/0	0/0	1/1	0/0	0/0	0/0	0/0	0/0	0/0	0/0	0/0	0/0	0/0	0/0	0/0	0/0	0/0	0/0	0/0	0/0	0/0	0/0	0/0	0/0	0/0	0/0	0/0
0/0	0/0	0/0	1/1	1/1	0/0	1/1	1/1	1/1	0/0	0/0	0/0	1/1	0/0	0/0	1/1	0/0	0/0	0/0	0/0	0/0	0/0	0/0	0/0	0/0	0/0	0/0
0/0	0/0	0/0	0/0	0/0	0/0	0/0	0/0	0/0	0/0	0/0	0/0	0/0	0/0	0/0	0/0	0/0	0/0	0/0	0/0	0/0	0/0	0/0	0/0	0/0	0/0	0/0
0/0	0/0	0/0	0/0	0/0	0/0	0/0	0/0	0/0	0/0	0/0	0/0	0/0	0/0	0/0	0/0	0/0	0/0	0/0	0/0	1/1	0/0	0/0	0/0	0/0	0/0	0/0
0/0	0/0	0/0	0/0	0/0	0/0	0/0	0/0	0/0	0/0	0/0	0/0	0/0	0/0	0/0	0/0	0/0	0/0	0/0	0/0	0/0	0/0	0/0	0/0	0/0	0/0	0/0

0/0	0/0	1/1	0/0	0/0	0/0	0/0	0/0	0/0	0/0	0/0	0/0	0/0	0/0	0/0	0/0	0/0	0/0	0/0	0/0	0/0	0/0	0/0	0/0	0/0	0/0	0/0
0/0	0/0	0/0	0/0	0/0	0/0	0/0	0/0	0/0	0/0	0/0	0/0	0/0	0/0	0/0	0/0	0/0	0/0	0/0	0/0	0/0	0/0	0/0	0/0	0/0	0/0	0/0
0/0	2/2	0/0	1/1	1/1	2/2	1/1	1/1	1/1	0/0	1/1	1/1	1/1	2/2	2/2	1/1	1/1	2/2	2/2	0/0	1/1	1/1	1/1	1/1	1/1	2/2	2/2
0/0	0/0	1/1	0/0	0/0	0/0	0/0	0/0	0/0	0/0	0/0	0/0	0/0	0/0	0/0	0/0	0/0	0/0	0/0	0/0	0/0	0/0	0/0	0/0	0/0	0/0	0/0
0/0	2/2	0/0	1/1	1/1	2/2	1/1	1/1	1/1	0/0	0/0	0/0	1/1	2/2	2/2	1/1	0/0	2/2	2/2	0/0	1/1	0/0	0/0	0/0	2/2	2/2	2/2
0/0	0/0	0/0	0/0	0/0	0/0	0/0	0/0	0/0	0/0	0/0	0/0	0/0	0/0	0/0	0/0	./.	0/0	0/0	0/0	0/0	0/0	0/0	0/0	0/0	0/0	0/0
0/0	2/2	0/0	0/0	0/0	0/0	0/0	0/0	0/0	0/0	0/0	0/0	0/0	0/0	0/0	0/0	1/1	0/0	0/0	0/0	0/0	1/1	1/1	1/1	0/0	0/0	0/0
0/0	1/1	1/1	1/1	1/1	1/1	1/1	1/1	1/1	0/0	1/1	1/1	1/1	1/1	1/1	1/1	1/1	1/1	1/1	0/0	1/1	1/1	1/1	1/1	1/1	1/1	1/1
0/0	1/1	0/0	1/1	1/1	1/1	1/1	1/1	1/1	0/0	1/1	1/1	1/1	1/1	1/1	1/1	1/1	1/1	1/1	0/0	1/1	1/1	1/1	1/1	1/1	1/1	1/1
0/0	0/0	0/0	0/0	0/0	0/0	0/0	0/0	0/0	0/0	0/0	0/0	0/0	0/0	0/0	0/0	0/0	0/0	0/0	0/0	0/0	0/0	0/0	0/0	0/0	0/0	0/0
0/0	2/2	3/3	1/1	1/1	2/2	1/1	1/1	1/1	0/0	2/2	2/2	1/1	2/2	2/2	1/1	2/2	2/2	2/2	0/0	2/2	2/2	2/2	2/2	2/2	2/2	2/2
0/0	0/0	0/0	0/0	0/0	0/0	0/0	0/0	0/0	0/0	0/0	0/0	0/0	0/0	0/0	0/0	0/0	0/0	0/0	0/0	0/0	0/0	0/0	0/0	0/0	0/0	0/0
0/0	0/0	0/0	0/0	0/0	0/0	0/0	0/0	0/0	0/0	0/0	0/0	0/0	0/0	0/0	0/0	0/0	0/0	0/0	0/0	0/0	0/0	0/0	0/0	0/0	0/0	0/0
0/0	0/0	0/0	0/0	0/0	0/0	0/0	0/0	0/0	0/0	0/0	0/0	0/0	0/0	0/0	0/0	0/0	0/0	0/0	0/0	0/0	0/0	0/0	0/0	0/0	0/0	0/0
0/0	0/0	0/0	0/0	0/0	0/0	0/0	0/0	0/0	0/0	1/1	1/1	0/0	0/0	0/0	0/0	1/1	0/0	0/0	0/0	0/0	1/1	1/1	1/1	0/0	0/0	0/0
0/0	0/0	0/0	0/0	0/0	0/0	0/0	0/0	0/0	0/0	0/0	0/0	0/0	0/0	0/0	0/0	0/0	0/0	0/0	0/0	0/0	0/0	0/0	0/0	0/0	0/0	0/0
0/0	0/0	0/0	0/0	0/0	0/0	0/0	0/0	0/0	0/0	0/0	1/1	0/0	0/0	0/0	0/0	1/1	0/0	0/0	0/0	0/0	1/1	1/1	1/1	0/0	0/0	0/0
0/0	1/1	1/1	1/1	1/1	1/1	1/1	1/1	1/1	0/0	1/1	1/1	1/1	1/1	1/1	1/1	1/1	1/1	1/1	0/0	1/1	1/1	1/1	1/1	1/1	1/1	1/1

452	453	461	462	463	468	469	46	471	476	477	484	489	491	494	500	505	50	511	515	517	51	520	521	522	523	525
0/0	0/0	0/0	0/0	0/0	0/0	0/0	0/0	0/0	0/0	0/0	0/0	0/0	0/0	0/0	0/0	0/0	0/0	0/0	0/0	0/0	0/0	0/0	0/0	0/0	0/0	0/0
0/0	1/1	1/1	0/0	1/1	0/0	0/0	0/0	0/0	0/0	1/1	0/0	0/0	1/1	1/1	0/0	0/0	1/1	0/0	0/0	0/0	0/0	0/0	1/1	1/1	0/0	1/1
0/0	0/0	0/0	0/0	0/0	0/0	0/0	0/0	0/0	0/0	0/0	0/0	0/0	0/0	0/0	0/0	0/0	0/0	0/0	0/0	0/0	0/0	0/0	0/0	0/0	0/0	0/0
0/0	0/0	0/0	0/0	0/0	0/0	0/0	0/0	0/0	0/0	0/0	0/0	0/0	0/0	0/0	0/0	0/0	0/0	0/0	0/0	0/0	0/0	0/0	0/0	0/0	0/0	0/0
0/0	0/0	0/0	1/1	0/0	1/1	0/0	1/1	1/1	1/1	0/0	1/1	0/0	0/0	0/0	1/1	1/1	0/0	1/1	1/1	1/1	0/1	1/1	0/0	0/0	0/0	0/0
0/0	0/0	0/0	1/1	0/0	1/1	0/0	1/1	1/1	1/1	0/0	1/1	0/0	0/0	0/0	1/1	1/1	0/0	1/1	1/1	1/1	0/1	1/1	0/0	0/0	0/0	0/0
0/0	0/0	0/0	0/0	0/0	0/0	0/0	0/0	0/0	0/0	0/0	0/0	0/0	0/0	0/0	0/0	0/0	0/0	0/0	0/0	0/0	0/0	0/0	0/0	0/0	0/0	0/0
1/1	1/1	1/1	0/0	1/1	0/0	1/1	0/0	0/0	0/0	1/1	0/0	1/1	1/1	1/1	0/0	0/0	1/1	0/0	0/0	0/0	0/0	0/0	1/1	1/1	1/1	1/1
0/0	0/0	0/0	0/0	0/0	0/0	0/0	0/0	0/0	0/0	0/0	0/0	0/0	0/0	0/0	0/0	0/0	0/0	0/0	0/0	0/0	0/0	0/0	0/0	0/0	0/0	0/0
0/0	0/0	0/0	0/0	0/0	0/0	0/0	0/0	0/0	0/0	0/0	0/0	0/0	0/0	0/0	0/0	0/0	0/0	0/0	0/0	0/0	0/0	0/0	0/0	0/0	0/0	0/0
0/0	0/0	0/0	0/0	0/0	0/0	0/0	0/1	0/0	0/1	0/0	0/0	0/0	0/0	0/1	0/0	0/0	0/0	0/0	0/0	0/0	0/0	0/0	0/0	0/0	0/0	0/0
0/0	0/0	0/0	0/0	0/0	0/0	0/0	0/0	0/0	0/0	0/0	0/0	0/0	0/0	0/0	0/0	0/0	0/0	0/0	0/0	0/0	0/0	0/0	0/0	0/0	0/0	0/0
0/0	0/0	0/0	1/1	0/0	1/1	0/0	1/1	1/1	1/1	0/0	1/1	0/0	0/0	0/0	1/1	1/1	0/0	1/1	1/1	1/1	0/1	1/1	0/0	0/0	0/0	0/0
1/1	0/1	0/1	0/0	0/1	0/0	0/0	0/0	0/0	0/0	0/1	0/0	0/0	0/0	./.	0/0	0/0	1/1	./.	0/0	0/0	0/0	0/0	0/0	0/0	./.	0/1
0/0	0/1	0/1	0/0	0/1	0/0	1/1	./.	0/0	./.	0/1	0/0	1/1	1/1	1/1	./.	0/0	0/0	./.	0/0	./.	0/0	./.	1/1	1/1	1/1	0/1
0/0	0/0	0/0	0/0	0/0	0/0	0/0	./.	0/0	./.	0/0	0/0	0/0	0/0	./.	./.	0/0	0/0	./.	0/0	./.	0/0	./.	./.	./.	./.	0/0

5/5	1/5	3/5	1/2	1/5	0/3	0/1	3/3	1/3	1/2	1/5	1/2	1/3	./.	./.	3/6	3/3	5/5	2/2	0/3	./.	2/2	2/2	./.	./.	6/6	3/5
0/0	0/0	0/0	0/0	0/0	0/0	0/0	0/0	0/0	0/0	0/0	0/0	0/0	./.	./.	0/0	0/0	0/0	0/0	0/0	./.	0/0	0/0	./.	./.	0/0	0/0
0/0	0/0	0/0	0/0	0/0	0/0	0/0	0/0	0/0	0/0	0/0	0/0	0/0	./.	./.	0/0	0/0	0/0	0/0	0/0	./.	0/0	0/0	./.	./.	0/0	0/0
0/0	0/0	0/0	1/1	0/0	1/1	0/0	1/1	1/1	1/1	0/0	1/1	0/0	0/0	0/0	1/1	1/1	0/0	1/1	1/1	1/1	0/1	1/1	0/0	0/0	0/0	0/0
1/1	1/1	1/1	0/0	1/1	0/0	1/1	0/0	0/0	0/0	1/1	0/0	1/1	1/1	1/1	0/0	0/0	1/1	0/0	0/0	0/0	0/0	0/0	1/1	1/1	1/1	1/1
0/0	0/0	0/0	0/0	0/0	0/0	0/0	0/0	0/0	0/0	0/0	0/0	0/0	0/0	0/0	0/0	0/0	0/0	0/0	0/0	0/0	0/0	0/0	0/0	0/0	0/0	0/0
1/1	1/1	1/1	0/0	1/1	0/0	1/1	0/0	0/0	0/0	1/1	0/0	1/1	1/1	1/1	0/0	0/0	1/1	0/0	0/0	0/0	0/0	0/0	1/1	1/1	1/1	1/1
0/0	0/0	0/0	1/1	0/0	1/1	0/0	1/1	1/1	1/1	0/0	1/1	0/0	0/0	0/0	1/1	1/1	0/0	1/1	1/1	1/1	0/0	1/1	0/0	0/0	0/0	0/0
0/0	0/0	0/0	0/0	0/0	0/0	0/0	0/0	0/0	0/0	0/0	0/0	0/0	0/0	0/0	0/0	0/0	0/0	0/0	0/0	0/0	1/1	0/0	0/0	0/0	0/0	0/0
1/1	0/0	0/0	1/1	0/0	1/1	1/1	1/1	1/1	1/1	0/0	1/1	1/1	0/0	0/0	1/1	1/1	0/0	1/1	1/1	1/1	2/2	1/1	0/0	0/0	1/1	0/0
0/0	0/0	0/0	0/0	0/0	0/0	0/0	0/0	0/0	0/0	0/0	0/0	0/0	0/0	0/0	0/0	0/0	0/0	0/0	0/0	0/0	./.	0/0	0/0	0/0	0/0	0/0
0/0	0/0	0/0	1/1	0/0	1/1	0/0	1/1	1/1	1/1	0/0	1/1	0/0	0/0	0/0	1/1	1/1	0/0	1/1	1/1	1/1	0/0	1/1	0/0	0/0	0/0	0/0
1/1	1/1	1/1	1/1	1/1	1/1	1/1	1/1	1/1	1/1	1/1	1/1	1/1	1/1	1/1	1/1	1/1	1/1	1/1	1/1	1/1	0/1	1/1	1/1	1/1	1/1	1/1
0/0	0/0	0/0	0/0	0/0	0/0	0/0	0/0	0/0	0/0	0/0	0/0	0/0	0/0	0/0	0/0	0/0	0/0	0/0	0/0	0/0	0/0	0/0	0/0	0/0	0/0	0/0
0/0	0/0	0/0	0/0	0/0	0/0	0/0	0/0	0/0	0/0	0/0	0/0	0/0	0/0	0/0	0/0	0/0	0/0	0/0	0/0	0/0	0/0	0/0	0/0	0/0	0/0	0/0
1/1	1/1	1/1	1/1	1/1	1/1	1/1	1/1	1/1	1/1	1/1	1/1	1/1	1/1	1/1	1/1	1/1	1/1	1/1	1/1	1/1	0/1	1/1	1/1	1/1	1/1	1/1
0/0	0/0	0/0	1/1	0/0	1/1	0/0	1/1	1/1	1/1	0/0	1/1	0/0	0/0	0/0	1/1	1/1	0/0	1/1	1/1	1/1	0/1	1/1	0/0	0/0	0/0	0/0
0/0	0/0	0/0	0/0	0/0	0/0	0/0	0/0	0/0	0/0	0/0	0/0	0/0	0/0	0/0	0/0	0/0	0/0	0/0	0/0	0/0	0/0	0/0	0/0	0/0	0/0	0/0
1/1	1/1	1/1	0/0	1/1	0/0	1/1	0/0	0/0	0/0	1/1	0/0	1/1	1/1	1/1	0/0	0/0	1/1	0/0	0/0	0/0	0/0	0/0	1/1	1/1	1/1	1/1
0/0	1/1	1/1	0/0	1/1	0/0	0/0	0/0	0/0	0/0	1/1	0/0	0/0	1/1	1/1	0/0	0/0	1/1	0/0	0/0	0/0	0/0	0/0	1/1	1/1	0/0	1/1
0/0	0/0	0/0	0/0	0/0	0/0	0/0	0/0	0/0	0/0	0/0	0/0	0/0	0/0	0/0	0/0	0/0	0/0	0/0	0/0	0/0	0/0	0/0	0/0	0/0	0/0	0/0
1/1	1/1	1/1	0/0	1/1	0/0	1/1	0/0	0/0	0/0	1/1	0/0	1/1	1/1	1/1	0/0	0/0	1/1	0/0	0/0	0/0	0/0	0/0	1/1	1/1	1/1	1/1
0/0	0/0	0/0	0/0	0/0	0/0	0/0	0/0	0/0	0/0	0/0	0/0	0/0	0/0	0/0	0/0	0/0	0/0	0/0	0/0	0/0	0/0	0/0	0/0	0/0	0/0	0/0
1/1	1/1	1/1	1/1	1/1	1/1	1/1	1/1	1/1	1/1	1/1	1/1	1/1	1/1	1/1	1/1	1/1	1/1	1/1	1/1	1/1	0/0	1/1	1/1	1/1	1/1	1/1
0/0	0/0	0/0	1/1	0/0	1/1	0/0	1/1	1/1	1/1	0/0	1/1	0/0	0/0	0/0	1/1	1/1	0/0	1/1	1/1	1/1	0/1	1/1	0/0	0/0	0/0	0/0
0/0	1/1	1/1	0/0	1/1	0/0	0/0	0/0	0/0	0/0	1/1	0/0	0/0	1/1	1/1	0/0	1/1	1/1	0/0	0/0	0/0	0/0	0/0	1/1	1/1	0/0	1/1

1/1	1/1	1/1	1/1	1/1	1/1	1/1	1/1	1/1	1/1	1/1	1/1	1/1	1/1	1/1	1/1	1/1	1/1	1/1	1/1	1/1	1/1	1/1	1/1	1/1	1/1	1/1
0/0	0/0	0/0	1/1	0/0	1/1	0/0	1/1	1/1	1/1	0/0	1/1	0/0	0/0	0/0	1/1	1/1	0/0	1/1	1/1	1/1	0/1	1/1	0/0	0/0	0/0	0/0
0/0	0/0	0/0	1/1	0/0	1/1	0/0	1/1	1/1	1/1	0/0	1/1	0/0	0/0	0/0	1/1	1/1	0/0	1/1	1/1	1/1	0/1	1/1	0/0	0/0	0/0	0/0
0/0	0/0	0/0	0/0	0/0	0/0	0/0	0/0	0/0	0/0	0/0	0/0	0/0	0/0	0/0	0/0	0/0	0/0	0/0	0/0	0/0	0/0	0/0	0/0	0/0	0/0	0/0
1/1	1/1	1/1	1/1	1/1	1/1	1/1	1/1	1/1	1/1	1/1	1/1	1/1	1/1	1/1	1/1	1/1	1/1	1/1	1/1	1/1	0/1	1/1	1/1	1/1	1/1	1/1
1/1	1/1	1/1	1/1	1/1	1/1	1/1	1/1	1/1	1/1	1/1	1/1	1/1	1/1	1/1	1/1	1/1	1/1	1/1	1/1	1/1	0/1	1/1	1/1	1/1	1/1	1/1
1/1	1/1	1/1	1/1	1/1	1/1	1/1	1/1	1/1	1/1	1/1	1/1	1/1	1/1	1/1	1/1	1/1	1/1	1/1	1/1	1/1	0/1	1/1	1/1	1/1	1/1	1/1
0/0	0/0	0/0	0/0	0/0	0/0	0/0	0/0	0/0	./.	0/0	0/0	0/0	0/0	0/0	0/0	0/0	0/0	0/0	0/0	0/0	0/0	0/0	0/0	0/0	0/0	0/0
0/0	0/0	0/0	1/1	0/0	1/1	0/0	1/1	1/1	1/1	0/0	1/1	0/0	0/0	0/0	1/1	1/1	0/0	1/1	1/1	1/1	0/1	1/1	0/0	0/0	0/0	0/0
0/0	0/0	0/0	0/0	0/0	0/0	0/0	0/0	0/0	0/0	0/0	0/0	0/0	0/0	0/0	0/0	0/0	0/0	0/0	0/0	0/0	0/0	0/0	0/0	0/0	0/0	0/0
1/1	1/1	1/1	0/0	1/1	0/0	1/1	0/0	0/0	0/0	1/1	0/0	1/1	1/1	1/1	0/0	0/0	1/1	0/0	0/0	0/0	0/0	0/0	1/1	1/1	1/1	1/1
0/0	0/0	0/0	0/0	0/0	0/0	0/0	0/0	0/0	0/0	0/0	0/0	0/0	0/0	0/0	1/1	0/0	0/0	0/0	0/0	0/0	0/0	0/0	0/0	0/0	0/0	0/0
0/0	0/0	0/0	0/0	0/0	0/0	0/0	0/0	0/0	0/0	0/0	0/0	0/0	0/0	0/0	0/0	0/0	0/0	0/0	0/0	0/0	0/0	0/0	0/0	0/0	0/0	0/0
1/1	0/0	0/0	0/0	0/0	0/0	1/1	0/0	0/0	0/0	0/0	0/0	1/1	0/0	0/0	0/0	0/0	0/0	0/0	0/0	0/0	0/0	0/0	0/0	0/0	1/1	0/0
1/1	1/1	1/1	1/1	1/1	1/1	1/1	1/1	1/1	1/1	1/1	1/1	1/1	1/1	1/1	1/1	1/1	1/1	1/1	1/1	1/1	0/1	1/1	1/1	1/1	1/1	1/1
0/0	0/0	0/0	0/0	0/0	0/0	0/0	0/0	0/0	0/0	0/0	0/0	0/0	0/0	0/0	0/0	0/0	0/0	0/0	0/0	0/0	0/0	0/0	0/0	0/0	0/0	0/0
0/0	0/0	0/0	0/0	0/0	0/0	0/0	0/0	0/0	0/0	0/0	0/0	0/0	0/0	0/0	0/0	0/0	0/0	0/0	0/0	0/0	0/0	0/0	0/0	0/0	0/0	0/0
0/0	0/0	0/0	0/0	0/0	0/0	0/0	0/0	0/0	0/0	0/0	0/0	0/0	0/0	0/0	0/0	0/0	0/0	0/0	0/0	0/0	0/0	0/0	0/0	0/0	0/0	0/0
1/1	1/1	1/1	0/0	1/1	0/0	1/1	0/0	0/0	0/0	1/1	0/0	1/1	1/1	1/1	0/0	0/0	1/1	0/0	0/0	0/0	0/1	0/0	1/1	1/1	1/1	1/1
0/0	0/0	0/0	0/0	0/0	0/0	0/0	0/0	0/0	0/0	0/0	0/0	0/0	0/0	0/0	0/0	0/0	0/0	0/0	0/0	0/0	0/0	0/0	0/0	0/0	0/0	0/0
1/1	1/1	1/1	1/1	1/1	1/1	1/1	1/1	1/1	1/1	1/1	1/1	1/1	1/1	1/1	1/1	1/1	1/1	1/1	1/1	1/1	0/1	1/1	1/1	1/1	1/1	1/1
1/1	1/1	1/1	0/0	1/1	0/0	1/1	0/0	0/0	0/0	1/1	0/0	1/1	1/1	1/1	0/0	0/0	1/1	0/0	0/0	0/0	0/1	0/0	1/1	1/1	1/1	1/1
1/1	1/1	1/1	0/0	1/1	0/0	1/1	0/0	0/0	0/0	1/1	0/0	1/1	1/1	1/1	0/0	0/0	1/1	0/0	0/0	0/0	0/1	0/0	1/1	1/1	1/1	1/1
0/0	0/0	0/0	1/1	0/0	1/1	0/0	1/1	1/1	1/1	0/0	1/1	0/0	0/0	0/0	1/1	1/1	0/0	1/1	1/1	1/1	0/1	1/1	0/0	0/0	0/0	0/0
1/1	1/1	1/1	1/1	1/1	1/1	1/1	1/1	1/1	1/1	1/1	1/1	1/1	1/1	1/1	1/1	1/1	1/1	1/1	1/1	1/1	0/1	1/1	1/1	1/1	1/1	1/1
0/0	0/0	0/0	1/1	0/0	1/1	0/0	1/1	1/1	1/1	0/0	1/1	0/0	0/0	0/0	1/1	1/1	0/0	1/1	1/1	./.	0/0	1/1	0/0	0/0	0/0	0/0
0/0	0/0	0/0	0/0	0/0	0/0	0/0	0/0	0/0	0/0	0/0	0/0	0/0	0/0	0/0	0/0	0/0	0/0	0/0	0/0	0/0	0/0	0/0	0/0	0/0	0/0	0/0
0/0	0/0	0/0	0/0	0/0	0/0	0/0	0/0	0/0	0/0	0/0	0/0	0/0	0/0	0/0	0/0	0/0	0/0	0/0	0/0	0/0	0/0	0/0	0/0	0/0	0/0	0/0
1/1	1/1	1/1	1/1	1/1	1/1	1/1	1/1	1/1	1/1	1/1	1/1	1/1	1/1	1/1	1/1	1/1	1/1	1/1	1/1	./.	1/1	1/1	1/1	1/1	./.	1/1
0/0	0/0	0/0	0/0	0/0	0/0	0/0	0/0	0/0	0/0	0/0	0/0	0/0	0/0	0/0	0/0	0/0	0/0	0/0	0/0	0/0	0/0	0/0	0/0	0/0	0/0	0/0
1/1	1/1	1/1	1/1	1/1	1/1	1/1	1/1	1/1	1/1	1/1	1/1	1/1	1/1	1/1	1/1	1/1	1/1	1/1	./.	./.	0/0	1/1	1/1	1/1	1/1	1/1
0/0	0/0	0/0	1/1	0/0	1/1	0/0	1/1	1/1	1/1	0/0	1/1	0/0	0/0	0/0	1/1	1/1	0/0	1/1	./.	./.	0/0	1/1	0/0	0/0	0/0	0/0
1/1	1/1	1/1	1/1	1/1	1/1	1/1	1/1	1/1	1/1	1/1	1/1	1/1	1/1	1/1	1/1	1/1	1/1	1/1	./.	./.	0/0	1/1	1/1	1/1	1/1	1/1
0/0	0/0	0/0	0/0	0/0	0/0	0/0	0/0	0/0	0/0	0/0	0/0	0/0	0/0	0/0	0/0	0/0	0/0	0/0	0/0	0/0	0/0	0/0	0/0	0/0	0/0	0/0
0/0	0/0	0/0	1/1	0/0	1/1	0/0	1/1	1/1	1/1	0/0	1/1	0/0	0/0	0/0	1/1	1/1	0/0	1/1	./.	./.	0/0	1/1	0/0	0/0	0/0	0/0

1/1	1/1	1/1	0/0	1/1	0/0	1/1	0/0	0/0	0/0	1/1	0/0	1/1	1/1	1/1	0/0	0/0	1/1	0/0	0/0	0/0	0/0	0/0	1/1	1/1	1/1	1/1
0/0	0/0	0/0	0/0	0/0	0/0	0/0	0/0	0/0	0/0	0/0	0/0	0/0	0/0	0/0	0/0	0/0	0/0	0/0	0/0	0/0	0/0	0/0	0/0	0/0	0/0	0/0
0/0	1/1	1/1	0/0	1/1	0/0	0/0	0/0	0/0	0/0	1/1	0/0	0/0	1/1	1/1	0/0	0/0	1/1	0/0	0/0	0/0	0/0	0/0	1/1	1/1	0/0	1/1
1/1	1/1	1/1	1/1	1/1	1/1	1/1	1/1	1/1	1/1	1/1	1/1	1/1	1/1	1/1	1/1	1/1	1/1	1/1	1/1	1/1	0/0	1/1	1/1	1/1	1/1	1/1
0/0	0/0	0/0	0/0	0/0	0/0	0/0	0/0	0/0	0/0	0/0	0/0	0/0	0/0	0/0	0/0	0/0	0/0	0/0	0/0	0/0	0/0	0/0	0/0	0/0	0/0	0/0
1/1	1/1	1/1	0/0	1/1	0/0	1/1	0/0	0/0	0/0	1/1	0/0	1/1	1/1	1/1	0/0	0/0	1/1	0/0	0/0	0/0	0/0	0/0	1/1	1/1	1/1	1/1
1/1	1/1	1/1	1/1	1/1	1/1	1/1	1/1	1/1	1/1	1/1	1/1	1/1	1/1	1/1	1/1	1/1	1/1	1/1	1/1	1/1	0/0	1/1	1/1	1/1	1/1	1/1
./.	0/0	0/0	1/1	0/0	1/1	0/0	1/1	1/1	1/1	0/0	1/1	0/0	0/0	0/0	1/1	1/1	./.	1/1	1/1	1/1	0/0	1/1	0/0	./.	0/0	0/0
1/1	1/1	1/1	0/0	1/1	0/0	1/1	0/0	0/0	0/0	1/1	0/0	1/1	1/1	1/1	0/0	0/0	1/1	0/0	0/0	0/0	0/0	0/0	1/1	1/1	1/1	1/1
0/0	0/0	0/0	0/0	0/0	0/0	0/0	0/0	0/0	0/0	0/0	0/0	0/0	0/0	0/0	0/0	0/0	0/0	0/0	0/0	0/0	0/0	0/0	0/0	0/0	0/0	0/0
1/1	1/1	1/1	0/0	1/1	0/0	1/1	0/0	0/0	0/0	1/1	0/0	1/1	1/1	1/1	0/0	0/0	1/1	0/0	0/0	0/0	0/0	0/0	1/1	1/1	1/1	1/1
0/0	0/0	0/0	0/0	0/0	0/0	0/0	0/0	0/0	0/0	0/0	0/0	0/0	0/0	0/0	0/0	0/0	0/0	0/0	0/0	0/0	0/0	0/0	0/0	0/0	0/0	0/0
0/0	0/0	0/0	0/0	0/0	0/0	0/0	0/0	0/0	0/0	0/0	0/0	0/0	0/0	0/0	0/0	0/0	0/0	0/0	0/0	0/0	0/0	0/0	0/0	0/0	0/0	0/0
0/0	0/0	0/0	0/0	0/0	0/0	0/0	0/0	0/0	0/0	0/0	0/0	0/0	0/0	0/0	0/0	0/0	0/0	0/0	0/0	0/0	0/0	0/0	0/0	0/0	0/0	0/0
1/1	1/1	1/1	1/1	1/1	1/1	1/1	1/1	1/1	1/1	1/1	1/1	1/1	1/1	1/1	1/1	1/1	1/1	1/1	1/1	1/1	1/1	1/1	1/1	1/1	1/1	1/1
1/1	1/1	1/1	1/1	1/1	1/1	1/1	1/1	1/1	1/1	1/1	1/1	1/1	1/1	1/1	1/1	1/1	1/1	1/1	1/1	1/1	1/1	1/1	1/1	1/1	1/1	1/1
1/1	1/1	1/1	1/1	1/1	1/1	1/1	1/1	1/1	1/1	1/1	1/1	1/1	1/1	1/1	1/1	1/1	1/1	1/1	1/1	1/1	0/0	1/1	1/1	1/1	1/1	1/1
./.	./.	./.	./.	./.	./.	./.	0/0	0/0	./.	./.	./.	./.	./.	./.	0/0	./.	2/2	./.	0/0	./.	./.	./.	./.	./.	./.	./.
1/1	1/1	1/1	3/3	1/1	3/3	1/1	3/3	3/3	3/3	1/1	3/3	1/1	1/1	1/1	3/3	3/3	4/4	3/3	3/3	3/3	1/1	3/3	1/1	1/1	1/1	1/1
0/0	0/0	0/0	1/1	0/0	0/0	0/0	0/0	1/1	1/1	0/0	1/1	0/0	0/0	0/0	0/0	1/1	0/0	1/1	0/0	0/0	0/0	1/1	0/0	0/0	0/0	0/0
0/0	0/0	0/0	0/0	0/0	0/0	0/0	0/0	0/0	0/0	0/0	0/0	0/0	0/0	0/0	0/0	0/0	0/0	0/0	0/0	0/0	0/0	0/0	0/0	0/0	0/0	0/0
0/0	0/0	0/0	0/0	0/0	0/0	0/0	0/0	0/0	0/0	0/0	0/0	0/0	0/0	0/0	0/0	0/0	0/0	0/0	0/0	0/0	0/0	0/0	0/0	0/0	0/0	0/0
0/0	0/0	0/0	0/0	0/0	0/0	0/0	0/0	0/0	0/0	0/0	0/0	0/0	0/0	0/0	0/0	0/0	0/0	0/0	0/0	0/0	0/0	0/0	0/0	0/0	0/0	0/0
0/0	0/0	0/0	./.	0/0	0/0	0/0	0/0	0/0	./.	0/0	0/0	0/0	0/0	0/0	0/0	0/0	0/0	0/0	0/0	./.	0/0	0/0	0/0	0/0	0/0	0/0
0/0	0/0	0/0	1/1	0/0	1/1	0/0	1/1	1/1	1/1	0/0	1/1	0/0	0/0	0/0	1/1	1/1	0/0	1/1	1/1	1/1	0/0	1/1	0/0	0/0	0/0	0/0

0/0	0/0	0/0	0/0	0/0	0/0	0/0	0/0	0/0	0/0	0/0	0/0	0/0	0/0	0/0	0/0	0/0	0/0	0/0	0/0	0/0	0/0	0/0	0/0	0/0	0/0	0/0
0/0	0/0	0/0	1/1	0/0	1/1	0/0	1/1	1/1	1/1	0/0	1/1	0/0	0/0	0/0	1/1	1/1	0/0	1/1	1/1	1/1	0/0	1/1	0/0	0/0	0/0	0/0
0/0	0/0	0/0	1/1	0/0	1/1	0/0	1/1	1/1	1/1	0/0	1/1	0/0	0/0	0/0	1/1	1/1	0/0	1/1	1/1	1/1	0/0	1/1	0/0	0/0	0/0	0/0
1/1	1/1	1/1	1/1	1/1	1/1	1/1	1/1	1/1	1/1	1/1	1/1	1/1	1/1	1/1	1/1	1/1	1/1	1/1	1/1	1/1	1/1	1/1	1/1	1/1	1/1	1/1
1/1	1/1	1/1	1/1	1/1	1/1	1/1	1/1	1/1	1/1	1/1	1/1	1/1	1/1	1/1	1/1	1/1	1/1	1/1	1/1	1/1	0/1	1/1	1/1	1/1	1/1	1/1
0/0	0/0	0/0	0/0	0/0	0/0	0/0	0/0	0/0	0/0	0/0	0/0	0/0	0/0	0/0	0/0	0/0	0/0	0/0	0/0	0/0	0/0	0/0	0/0	0/0	0/0	0/0
1/1	1/1	1/1	1/1	1/1	1/1	1/1	1/1	1/1	1/1	1/1	1/1	1/1	1/1	1/1	1/1	1/1	1/1	1/1	1/1	1/1	0/1	1/1	1/1	1/1	0/1	1/1
0/0	0/0	0/0	1/1	0/0	1/1	0/0	1/1	1/1	1/1	0/0	1/1	0/0	0/0	0/0	1/1	1/1	0/0	1/1	1/1	1/1	0/1	1/1	0/0	0/0	0/0	0/0
0/0	0/0	0/0	0/0	0/0	0/0	0/0	0/0	0/0	0/0	0/0	0/0	0/0	0/0	0/0	0/0	0/0	0/0	0/0	0/0	0/0	0/0	0/0	0/0	0/0	0/0	0/0
0/0	0/0	0/0	0/0	0/0	0/0	0/0	0/0	0/0	0/0	0/0	0/0	0/0	0/0	0/0	0/0	0/0	0/0	0/0	0/0	0/0	0/0	0/0	0/0	0/0	0/0	0/0
0/0	0/0	0/0	1/1	0/0	1/1	0/0	1/1	1/1	1/1	0/0	1/1	0/0	0/0	0/0	1/1	1/1	0/0	1/1	1/1	1/1	0/0	1/1	0/0	0/0	0/0	0/0
0/0	0/0	0/0	0/0	0/0	0/0	0/0	0/0	1/1	0/0	0/0	0/0	0/0	0/0	0/0	0/0	0/0	0/0	0/0	0/0	0/0	0/0	0/0	0/0	0/0	0/0	0/0
1/1	1/1	1/1	0/0	1/1	0/0	1/1	0/0	0/0	0/0	1/1	0/0	1/1	1/1	1/1	0/0	0/0	1/1	0/0	0/0	0/0	0/0	0/0	1/1	1/1	1/1	1/1
0/0	0/0	0/0	1/1	0/0	1/1	0/0	1/1	1/1	1/1	0/0	1/1	0/0	0/0	0/0	1/1	1/1	0/0	1/1	1/1	1/1	0/0	1/1	0/0	0/0	0/0	0/0
0/0	0/0	0/0	0/0	0/0	0/0	0/0	0/0	0/0	0/0	0/0	0/0	0/0	0/0	0/0	0/0	0/0	0/0	0/0	0/0	0/0	0/0	0/0	0/0	0/0	0/0	0/0
1/1	1/1	1/1	1/1	1/1	1/1	1/1	1/1	1/1	1/1	1/1	1/1	1/1	1/1	1/1	1/1	1/1	1/1	1/1	1/1	1/1	0/0	1/1	1/1	1/1	1/1	1/1
1/1	1/1	1/1	0/0	1/1	0/0	1/1	0/0	0/0	0/0	1/1	0/0	1/1	1/1	1/1	0/0	0/0	1/1	0/0	0/0	0/0	0/0	0/0	1/1	1/1	1/1	1/1
1/1	0/0	0/0	0/0	0/0	0/0	1/1	0/0	0/0	0/0	0/0	0/0	1/1	0/0	0/0	0/0	0/0	0/0	0/0	0/0	0/0	0/0	0/0	0/0	0/0	1/1	0/0
0/0	0/0	0/0	0/0	0/0	0/0	0/0	./.	0/0	0/0	0/0	0/0	0/0	0/0	0/0	0/0	0/0	0/0	0/0	./.	0/0	0/0	0/0	0/0	0/0	0/0	0/0
0/0	0/0	0/0	1/1	0/0	1/1	0/0	1/1	1/1	1/1	0/0	1/1	0/0	0/0	0/0	1/1	1/1	0/0	1/1	1/1	1/1	0/0	1/1	0/0	0/0	0/0	0/0
0/0	0/0	0/0	1/1	0/0	1/1	0/0	1/1	1/1	1/1	0/0	1/1	0/0	0/0	0/0	1/1	1/1	0/0	1/1	1/1	1/1	0/0	1/1	0/0	0/0	0/0	0/0
0/0	0/0	0/0	0/0	0/0	0/0	0/0	0/0	0/0	0/0	0/0	0/0	0/0	0/0	0/0	0/0	0/0	0/0	0/0	0/0	0/0	0/0	0/0	0/0	0/0	0/0	0/0
1/1	1/1	1/1	1/1	1/1	1/1	1/1	1/1	1/1	1/1	1/1	1/1	1/1	1/1	1/1	1/1	1/1	1/1	1/1	1/1	1/1	0/1	1/1	1/1	1/1	1/1	1/1
0/0	0/0	0/0	0/0	0/0	0/0	0/0	0/0	0/0	0/0	0/0	0/0	0/0	0/0	0/0	0/0	0/0	0/0	0/0	0/0	0/0	0/0	0/0	0/0	0/0	0/0	0/0
0/0	0/0	0/0	0/0	0/0	0/0	0/0	0/0	0/0	0/0	0/0	0/0	0/0	0/0	0/0	0/0	0/0	0/0	0/0	0/0	0/0	0/0	0/0	0/0	0/0	0/0	0/0
0/0	0/0	0/0	1/1	0/0	1/1	0/0	1/1	1/1	1/1	0/0	1/1	0/0	0/0	0/0	1/1	1/1	0/0	1/1	1/1	1/1	0/1	1/1	0/0	0/0	0/0	0/0
0/0	0/0	0/0	0/0	0/0	0/0	0/0	0/0	0/0	0/0	0/0	0/0	0/0	0/0	0/0	0/0	0/0	0/0	0/0	0/0	0/0	0/0	0/0	0/0	0/0	0/0	0/0
1/1	1/1	1/1	0/0	1/1	0/0	1/1	0/0	0/0	0/0	1/1	0/0	1/1	1/1	1/1	0/0	0/0	1/1	0/0	0/0	0/0	0/0	0/0	1/1	1/1	1/1	1/1
0/0	0/0	0/0	1/1	0/0	1/1	0/0	1/1	1/1	1/1	0/0	1/1	0/0	0/0	0/0	1/1	1/1	0/0	1/1	1/1	1/1	0/1	1/1	0/0	0/0	0/0	0/0
0/0	0/0	0/0	0/0	0/0	0/0	0/0	0/0	0/0	0/0	0/0	0/0	0/0	0/0	0/0	0/0	0/0	0/0	0/0	0/0	0/0	0/0	0/0	0/0	0/0	0/0	0/0
1/1	1/1	1/1	1/1	1/1	1/1	1/1	1/1	1/1	1/1	1/1	1/1	1/1	1/1	1/1	1/1	1/1	1/1	1/1	1/1	1/1	0/1	1/1	1/1	1/1	1/1	1/1
0/0	0/0	0/0	0/0	0/0	0/0	0/0	0/0	0/0	0/0	0/0	0/0	0/0	0/0	0/0	0/0	0/0	0/0	0/0	0/0	0/0	0/0	0/0	0/0	0/0	0/0	0/0
0/0	0/0	0/0	0/0	0/0	0/0	0/0	0/0	0/0	0/0	0/0	0/0	0/0	0/0	0/0	0/0	0/0	0/0	0/0	0/0	0/0	0/1	0/0	0/0	0/0	0/0	0/0

1/1	1/1	1/1	1/1	1/1	1/1	1/1	1/1	1/1	1/1	1/1	1/1	1/1	1/1	1/1	1/1	1/1	1/1	1/1	1/1	1/1	0/0	1/1	1/1	1/1	1/1	1/1
0/0	0/0	0/0	0/0	0/0	0/0	0/0	0/0	0/0	0/0	0/0	0/0	0/0	0/0	0/0	0/0	0/0	0/0	0/0	0/0	0/0	0/0	0/0	0/0	0/0	0/0	0/0
1/1	1/1	1/1	1/1	1/1	1/1	1/1	1/1	1/1	1/1	1/1	1/1	1/1	1/1	1/1	1/1	1/1	1/1	1/1	1/1	1/1	0/1	1/1	1/1	1/1	1/1	1/1
0/0	0/0	0/0	0/0	0/0	0/0	0/0	0/0	0/0	0/0	0/0	0/0	0/0	0/0	0/0	0/0	0/0	0/0	0/0	0/0	0/0	0/0	0/0	0/0	0/0	0/0	0/0
0/0	1/1	1/1	0/0	1/1	0/0	0/0	0/0	0/0	0/0	1/1	0/0	0/0	1/1	1/1	0/0	0/0	1/1	0/0	0/0	0/0	0/0	0/0	1/1	1/1	0/0	1/1
0/0	0/0	0/0	0/0	0/0	0/0	0/0	0/0	0/0	0/0	0/0	0/0	0/0	0/0	0/0	0/0	0/0	0/0	0/0	0/0	0/0	0/0	0/0	0/0	0/0	0/0	0/0
0/0	0/0	0/0	0/0	0/0	0/0	0/0	0/0	0/0	0/0	0/0	0/0	0/0	0/0	0/0	0/0	0/0	0/0	0/0	0/0	0/0	0/0	0/0	0/0	0/0	0/0	0/0
1/1	0/0	0/0	0/0	0/0	0/0	1/1	0/0	0/0	0/0	0/0	0/0	1/1	0/0	0/0	0/0	0/0	0/0	0/0	0/0	0/0	0/0	0/0	0/0	0/0	1/1	0/0
0/0	0/0	0/0	0/0	0/0	0/0	0/0	0/0	0/0	0/0	0/0	0/0	0/0	0/0	0/0	0/0	0/0	0/0	0/0	0/0	0/0	0/0	0/0	0/0	0/0	0/0	0/0
0/0	0/0	0/0	0/0	0/0	0/0	0/0	0/0	0/0	0/0	0/0	0/0	0/0	0/0	0/0	0/0	0/0	0/0	0/0	0/0	0/0	0/0	0/0	0/0	0/0	0/0	0/0
1/1	1/1	1/1	1/1	1/1	1/1	1/1	1/1	1/1	1/1	1/1	1/1	1/1	1/1	1/1	1/1	1/1	1/1	1/1	1/1	1/1	0/0	1/1	1/1	1/1	1/1	1/1
1/1	1/1	1/1	1/1	1/1	1/1	1/1	1/1	1/1	1/1	1/1	1/1	1/1	1/1	1/1	1/1	1/1	1/1	1/1	1/1	1/1	0/0	1/1	1/1	1/1	1/1	1/1
0/0	0/0	0/0	0/0	0/0	0/0	0/0	0/0	0/0	0/0	0/0	0/0	0/0	0/0	0/0	0/0	0/0	0/0	0/0	0/0	0/0	0/0	0/0	0/0	0/0	0/0	0/0
0/0	1/1	1/1	0/0	1/1	0/0	0/0	0/0	0/0	0/0	1/1	0/0	0/0	1/1	1/1	0/0	0/0	1/1	0/0	0/0	0/0	0/0	0/0	1/1	1/1	0/0	1/1
0/0	0/0	0/0	1/1	0/0	1/1	0/0	1/1	1/1	1/1	0/0	1/1	0/0	0/0	0/0	1/1	1/1	0/0	1/1	1/1	1/1	0/0	1/1	0/0	0/0	0/0	0/0
1/1	1/1	1/1	1/1	1/1	1/1	1/1	1/1	1/1	1/1	1/1	1/1	1/1	1/1	1/2	1/1	1/1	1/1	1/1	0/1	1/1	2/2	0/1	1/1	1/1	1/1	1/1
1/1	1/1	1/1	1/1	1/1	1/1	1/1	1/1	1/1	1/1	1/1	1/1	1/1	1/1	1/1	1/1	1/1	1/1	1/1	1/1	1/1	0/0	1/1	1/1	1/1	1/1	1/1
1/1	0/0	0/0	1/1	0/0	1/1	1/1	1/1	1/1	1/1	0/0	1/1	0/0	0/0	0/0	1/1	1/1	0/0	1/1	1/1	1/1	0/0	1/1	0/0	0/0	0/0	0/0
1/1	0/0	0/0	1/1	0/0	1/1	1/1	1/1	1/1	1/1	0/0	1/1	0/0	0/0	0/0	1/1	1/1	0/0	1/1	1/1	1/1	0/1	1/1	0/0	0/0	0/0	0/0
0/0	1/1	1/1	0/0	1/1	0/0	0/0	0/0	0/0	0/0	1/1	0/0	0/0	1/1	1/1	0/0	0/0	1/1	0/0	0/0	0/0	0/0	0/0	1/1	1/1	0/0	1/1
0/0	0/0	0/0	0/0	0/0	0/0	0/0	0/0	0/0	0/0	0/0	0/0	0/0	0/0	0/0	0/0	0/0	0/0	0/0	0/0	0/0	0/0	0/0	0/0	0/0	0/0	0/0
0/0	0/0	0/0	0/0	0/0	0/0	0/0	0/0	0/0	0/0	0/0	0/0	0/0	0/0	0/0	0/0	0/0	0/0	0/0	0/0	0/0	0/0	0/0	0/0	0/0	0/0	0/0
0/0	0/0	0/0	0/0	0/0	0/0	0/1	0/0	0/0	0/0	0/1	0/0	0/0	0/0	0/0	0/0	0/0	0/0	0/0	0/0	0/0	0/0	0/1	0/1	0/0	0/0	0/0
0/0	0/0	0/0	0/0	0/0	0/0	0/0	0/0	0/0	0/0	0/0	0/0	0/0	0/0	0/0	0/0	0/0	0/0	0/0	0/0	0/0	0/0	0/0	0/0	0/0	0/0	0/0
0/0	0/0	0/0	0/1	0/0	0/0	0/1	0/0	0/0	0/0	0/1	0/0	0/0	0/0	0/0	0/0	0/0	0/0	0/0	0/0	0/1	0/0	0/1	0/1	0/0	0/0	0/0
0/0	0/0	0/0	0/1	0/0	0/0	0/1	0/0	0/0	0/0	0/1	0/0	0/0	0/0	0/0	0/0	0/0	0/0	0/0	0/0	0/1	0/0	0/1	0/1	0/0	0/0	0/0

0/0	0/0	0/0	0/1	0/0	0/0	0/1	0/0	0/0	0/0	0/1	0/0	0/0	0/0	0/0	0/0	0/0	0/0	0/0	0/0	0/1	0/0	0/1	0/1	0/0	0/0	0/0
0/0	0/0	0/0	0/1	0/0	0/0	0/1	0/0	0/0	0/0	0/1	0/0	0/0	0/0	0/0	0/0	0/0	0/0	0/0	0/0	0/1	0/0	0/1	0/1	0/0	0/0	0/0
0/0	0/0	0/0	0/1	0/0	0/0	0/1	0/0	0/0	0/0	0/1	0/0	0/0	0/0	0/0	0/0	0/0	0/0	0/0	0/0	0/1	0/0	0/1	0/1	0/0	0/0	0/0
0/0	0/0	0/0	0/1	0/0	0/0	0/1	0/0	0/0	0/0	0/1	0/0	0/0	0/0	0/0	0/0	0/0	0/0	0/0	0/0	0/1	0/0	0/1	0/1	0/0	0/0	0/0
0/0	0/0	0/0	0/1	0/0	0/0	0/1	0/0	0/0	0/0	0/1	0/0	0/0	0/0	0/0	0/0	0/0	0/0	0/0	0/0	0/1	0/0	0/0	0/1	0/0	0/0	0/0
0/0	0/0	0/0	0/0	0/0	0/0	0/1	0/0	0/0	0/0	0/1	0/0	0/0	0/0	0/0	0/0	0/0	0/0	0/0	0/0	0/1	0/0	0/0	0/1	0/0	0/0	0/0
0/0	0/0	0/0	0/0	0/0	0/0	0/0	0/0	0/0	0/0	0/0	0/0	0/0	0/0	0/0	0/0	0/0	0/0	0/0	0/0	0/0	0/0	0/0	0/0	0/0	0/0	0/0
0/0	0/0	0/0	0/0	0/0	0/0	0/1	0/0	0/0	0/0	0/0	0/0	0/0	0/0	0/0	0/0	0/0	0/0	0/0	0/0	0/0	0/0	0/0	0/0	0/0	0/0	0/0
0/0	0/0	0/0	0/0	0/0	0/0	0/1	0/0	0/0	0/0	0/0	0/0	0/0	0/0	0/0	0/0	0/0	0/0	0/0	0/0	0/0	0/0	0/0	0/0	0/0	0/0	0/0
0/0	0/0	0/0	0/0	0/0	0/0	0/0	0/0	0/0	0/0	0/0	0/0	0/0	0/0	0/0	0/0	0/0	0/0	0/0	0/0	0/0	0/0	0/0	0/0	0/0	0/0	0/0
1/1	1/1	1/1	1/1	1/1	1/1	1/1	1/1	1/1	1/1	1/1	1/1	1/1	1/1	1/1	1/1	1/1	1/1	1/1	1/1	1/1	1/1	1/1	1/1	1/1	1/1	1/1
0/0	0/0	0/0	0/0	0/0	0/0	0/0	0/0	0/0	0/0	0/0	0/0	0/0	0/0	0/0	0/0	0/0	0/0	0/0	0/0	0/0	0/0	0/0	0/0	0/0	0/0	0/0
0/0	0/0	0/0	0/0	0/0	0/0	0/0	0/0	0/0	0/0	0/0	0/0	0/0	0/0	0/0	0/0	0/0	0/0	0/0	0/0	0/0	0/0	0/0	0/0	0/0	0/0	0/0
0/0	0/0	0/0	0/0	0/0	0/0	0/0	0/0	0/0	0/0	0/0	0/0	1/1	0/0	0/0	0/0	0/0	0/0	0/0	0/0	0/0	0/0	0/0	0/0	0/0	1/1	0/0
0/0	0/0	0/0	0/0	0/0	0/0	0/0	0/0	0/0	0/0	0/0	0/0	0/0	0/0	0/0	0/0	0/0	0/0	0/0	0/0	0/0	0/0	0/0	0/0	0/0	0/0	0/0
0/0	0/0	0/0	0/0	0/0	0/0	0/1	0/0	0/0	0/0	0/0	0/0	0/0	0/0	0/0	0/0	0/0	0/0	0/0	0/0	0/0	0/0	0/0	0/0	0/0	0/0	0/0
1/1	1/1	1/1	1/1	1/1	1/1	1/1	1/1	1/1	1/1	1/1	1/1	1/1	1/1	1/1	1/1	1/1	1/1	1/1	1/1	1/1	0/0	1/1	1/1	1/1	1/1	1/1
./.	0/0	0/0	./.	0/0	0/0	0/0	./.	./.	./.	0/0	./.	0/0	0/0	0/0	./.	0/0	0/0	./.	0/0	./.	0/0	./.	./.	./.	0/0	0/0
1/1	1/1	1/1	./.	1/1	0/0	1/1	./.	./.	./.	1/1	./.	1/1	1/1	1/1	./.	0/0	1/1	./.	0/0	./.	0/0	./.	1/1	1/1	1/1	1/1
0/0	0/0	0/0	1/1	0/0	1/1	0/0	1/1	1/1	1/1	0/0	1/1	0/0	0/0	0/0	1/1	1/1	0/0	1/1	1/1	1/1	0/0	1/1	0/0	0/0	0/0	0/0
0/0	0/0	0/0	1/1	0/0	1/1	0/0	1/1	1/1	1/1	0/0	1/1	0/0	0/0	0/0	1/1	1/1	0/0	1/1	1/1	1/1	0/1	1/1	0/0	0/0	0/0	0/0
./.	./.	./.	0/0	./.	0/0	0/0	0/0	0/0	0/0	0/0	0/0	0/0	./.	0/0	0/0	0/0	./.	0/0	0/0	0/0	0/0	0/0	./.	./.	./.	0/0
0/0	./.	0/0	0/0	./.	0/0	./.	0/0	0/0	0/0	0/0	0/0	0/0	./.	./.	0/0	0/0	./.	0/0	0/0	0/0	0/0	0/0	./.	0/0	1/1	./.
1/1	1/1	0/1	0/0	0/1	0/0	1/1	0/0	0/0	0/0	1/1	0/0	1/1	1/1	1/1	0/0	0/0	0/1	0/0	0/0	0/0	0/0	0/0	1/1	1/1	0/0	1/1

0/0	2/2	0/0	0/0	0/1	0/0	2/2	0/0	0/0	0/0	2/2	0/0	0/0	2/2	0/0	0/0	0/0	0/1	0/0	0/0	0/0	0/0	0/0	0/0	0/0	2/2	2/2
0/0	4/4	0/0	0/0	0/4	0/0	4/4	0/0	0/0	0/0	4/4	0/0	0/0	4/4	4/4	0/0	0/2	0/4	0/0	0/0	0/0	0/3	0/0	4/4	0/0	4/4	4/4
0/0	0/0	0/0	0/0	0/0	0/0	0/0	0/0	0/0	0/0	0/0	0/0	0/0	0/0	0/0	0/0	0/0	0/0	0/0	0/0	0/0	0/0	0/0	0/0	0/0	0/0	0/0
1/1	1/1	1/1	1/1	1/1	1/1	1/1	1/1	1/1	1/1	1/1	1/1	1/1	1/1	1/1	1/1	1/1	1/1	1/1	1/1	1/1	0/1	1/1	1/1	1/1	1/1	1/1
1/1	1/1	1/1	0/0	1/1	0/0	1/1	0/0	0/0	0/0	1/1	0/0	1/1	1/1	1/1	0/0	0/0	1/1	0/0	0/0	0/0	0/0	0/0	1/1	1/1	1/1	1/1
0/0	0/0	0/0	0/0	0/0	0/0	0/0	0/0	0/0	0/0	0/0	0/0	0/0	0/0	0/0	0/0	0/0	0/0	0/0	0/0	0/0	0/0	0/0	0/0	0/0	0/0	0/0
0/0	0/0	0/0	0/0	0/0	0/0	0/0	0/0	0/0	0/0	0/0	0/0	0/0	0/0	0/0	0/0	0/0	0/0	0/0	0/0	0/0	0/0	0/0	0/0	0/0	0/0	0/0
1/1	1/1	1/1	1/1	1/1	1/1	1/1	1/1	1/1	1/1	1/1	1/1	1/1	1/1	1/1	1/1	1/1	1/1	1/1	1/1	1/1	0/1	1/1	1/1	1/1	1/1	1/1
0/0	./.	0/0	0/0	./.	0/0	./.	0/0	0/0	0/0	./.	0/0	0/0	./.	0/0	0/0	0/0	./.	0/0	0/0	0/0	0/0	0/0	./.	0/0	./.	0/0
1/1	1/1	1/1	0/0	1/1	0/0	1/2	0/0	0/0	0/0	1/1	0/0	1/1	1/1	1/1	0/0	0/0	1/1	0/0	0/0	0/0	0/0	0/0	1/1	1/1	1/1	1/1
1/1	1/1	1/1	0/0	1/1	0/0	1/1	0/0	0/0	0/0	1/1	0/0	1/1	1/1	1/1	0/0	0/0	1/1	0/0	0/0	0/0	0/0	0/0	1/1	1/1	1/1	1/1
1/1	1/1	1/1	0/0	1/1	0/0	1/1	0/0	0/0	0/0	1/1	0/0	1/1	1/1	1/1	0/0	0/0	1/1	0/0	0/0	0/0	0/0	0/0	1/1	1/1	1/1	1/1
0/0	0/0	0/0	1/1	0/0	1/1	0/0	1/1	1/1	1/1	0/0	1/1	0/0	0/0	0/0	1/1	1/1	0/0	1/1	1/1	1/1	0/0	1/1	0/0	0/0	0/0	0/0
1/1	1/1	1/1	0/0	1/1	0/0	1/1	0/0	0/0	0/0	1/1	0/0	1/1	1/1	1/1	0/0	0/0	1/1	0/0	0/0	0/0	0/0	0/0	1/1	1/1	1/1	1/1
1/1	1/1	1/1	1/1	1/1	1/1	1/1	1/1	1/1	1/1	1/1	1/1	1/1	1/1	1/1	1/1	1/1	1/1	1/1	1/1	1/1	0/0	1/1	1/1	1/1	1/1	1/1
0/0	0/0	0/0	0/0	0/0	0/0	0/0	0/0	0/0	0/0	0/0	0/0	0/0	0/0	0/0	0/0	0/0	0/0	0/0	0/0	0/0	0/0	0/0	0/0	0/0	0/0	0/0
0/0	0/0	0/0	0/0	0/0	0/0	0/0	0/0	0/0	0/0	0/0	0/0	0/0	0/0	0/0	0/0	0/0	0/0	0/0	0/0	0/0	0/0	0/0	0/0	0/0	0/0	0/0
0/0	0/0	0/0	1/1	0/0	1/1	0/0	1/1	1/1	1/1	0/0	1/1	0/0	0/0	0/0	1/1	1/1	0/0	1/1	1/1	1/1	0/0	1/1	0/0	0/0	0/0	0/0
0/0	0/0	0/0	0/0	0/0	0/0	0/0	0/0	0/0	0/0	0/0	0/0	0/0	0/0	0/0	0/0	0/0	0/0	0/0	0/0	0/0	0/0	0/0	0/0	0/0	0/0	0/0
0/0	0/0	0/0	1/1	0/0	1/1	0/0	1/1	1/1	1/1	0/0	1/1	0/0	0/0	0/0	1/1	1/1	0/0	1/1	1/1	1/1	0/0	1/1	0/0	0/0	0/0	0/0
1/1	0/0	0/0	0/0	0/0	0/0	1/1	0/0	0/0	0/0	0/0	0/0	1/1	0/0	0/0	0/0	0/0	0/0	0/0	0/0	0/0	0/0	0/0	0/0	0/0	1/1	0/0
0/0	0/0	0/0	1/1	0/0	1/1	0/0	1/1	1/1	1/1	0/0	1/1	0/0	0/0	0/0	1/1	1/1	0/0	1/1	1/1	1/1	0/0	1/1	0/0	0/0	0/0	0/0
0/0	0/0	0/0	1/1	0/0	1/1	0/0	1/1	1/1	1/1	0/0	1/1	0/0	0/0	0/0	1/1	1/1	0/0	1/1	1/1	1/1	0/0	1/1	0/0	0/0	0/0	0/0
0/0	0/0	0/0	0/0	0/0	0/0	0/0	0/0	0/0	0/0	0/0	0/0	0/0	0/0	0/0	0/0	0/0	0/0	0/0	0/0	0/0	0/0	0/0	0/0	0/0	0/0	0/0
0/0	0/0	0/0	0/0	0/0	0/0	0/0	0/0	0/0	0/0	0/0	0/0	0/0	0/0	0/0	0/0	0/0	0/0	0/0	0/0	0/0	0/0	0/0	0/0	0/0	0/0	0/0
1/1	1/1	1/1	0/0	1/1	0/0	1/1	0/0	0/0	0/0	1/1	0/0	1/1	1/1	1/1	0/0	0/0	1/1	0/0	0/0	0/0	0/0	0/0	1/1	1/1	1/1	1/1
0/0	0/0	0/0	0/0	0/0	0/0	0/0	0/0	0/0	0/0	0/0	0/0	0/0	0/0	0/0	0/0	0/0	0/0	0/0	0/0	0/0	0/0	0/0	0/0	0/0	0/0	0/0
1/1	1/1	1/1	1/1	1/1	1/1	1/1	1/1	1/1	1/1	1/1	1/1	1/1	1/1	1/1	1/1	1/1	1/1	1/1	1/1	1/1	0/1	1/1	1/1	1/1	1/1	1/1
0/0	0/0	0/0	0/0	0/0	0/0	0/0	0/0	0/0	0/0	0/0	0/0	0/0	0/0	0/0	0/0	0/0	0/0	0/0	0/0	0/0	0/0	0/0	0/0	0/0	0/0	0/0
1/1	1/1	1/1	1/1	1/1	1/1	1/1	1/1	1/1	1/1	1/1	1/1	1/1	1/1	1/1	1/1	1/1	1/1	1/1	1/1	1/1	0/1	1/1	1/1	1/1	1/1	1/1

0/0	0/0	0/0	0/0	0/0	0/0	0/0	0/0	0/0	0/0	0/0	0/0	0/0	0/0	0/0	0/0	0/0	0/0	0/0	0/0	0/0	0/0	0/0	0/0	0/0	0/0	0/0
0/0	0/0	0/0	0/0	0/0	0/0	0/0	0/0	0/0	0/0	0/0	0/0	0/0	0/0	0/0	0/0	0/0	0/0	0/0	0/0	0/0	0/0	0/0	0/0	0/0	0/0	0/0
0/0	0/0	0/0	0/0	0/0	0/0	0/0	0/0	0/0	0/0	0/0	0/0	0/0	0/0	0/0	0/0	0/0	0/0	0/0	0/0	0/0	0/0	0/0	0/0	0/0	0/0	0/0
0/0	1/1	1/1	0/0	1/1	0/0	0/0	0/0	0/0	0/0	1/1	0/0	0/0	1/1	1/1	0/0	0/0	1/1	0/0	0/0	0/0	0/0	0/0	1/1	1/1	0/0	1/1
1/1	1/1	1/1	0/0	1/1	0/0	1/1	0/0	0/0	0/0	1/1	0/0	1/1	1/1	1/1	0/0	0/0	1/1	0/0	0/0	0/0	0/0	0/0	1/1	1/1	1/1	1/1
1/1	1/1	1/1	1/1	1/1	1/1	1/1	1/1	1/1	1/1	1/1	1/1	1/1	1/1	1/1	1/1	1/1	1/1	1/1	1/1	1/1	0/1	1/1	1/1	1/1	1/1	1/1
1/1	1/1	1/1	0/0	1/1	0/0	1/1	0/0	0/0	0/0	1/1	0/0	1/1	1/1	1/1	0/0	0/0	1/1	0/0	0/0	0/0	0/0	0/0	1/1	1/1	1/1	1/1
1/1	0/0	0/0	0/0	0/0	0/0	1/1	0/0	0/0	0/0	0/0	0/0	1/1	0/0	0/0	0/0	0/0	0/0	0/0	0/0	0/0	0/0	0/0	0/0	0/0	1/1	0/0
0/0	0/0	0/0	0/0	0/0	0/0	0/0	0/0	0/0	0/0	0/0	0/0	0/0	0/0	0/0	0/0	0/0	0/0	0/0	0/0	0/0	0/0	0/0	0/0	0/0	0/0	0/0
0/0	0/0	0/0	0/0	0/0	0/0	0/0	0/0	0/0	0/0	0/0	0/0	0/0	0/0	0/0	0/0	0/0	0/0	0/0	0/0	0/0	0/0	0/0	0/0	0/0	0/0	0/0
0/0	0/0	0/0	0/0	0/0	0/0	0/0	0/0	0/0	0/0	0/0	0/0	0/0	0/0	0/0	0/0	0/0	0/0	0/0	0/0	0/0	0/0	0/0	0/0	0/0	0/0	0/0
0/0	0/0	0/0	1/1	0/0	1/1	0/0	1/1	1/1	1/1	0/0	1/1	0/0	0/0	0/0	1/1	1/1	0/0	1/1	1/1	1/1	0/0	1/1	0/0	0/0	0/0	0/0
0/0	1/1	1/1	1/1	1/1	1/1	0/0	1/1	1/1	1/1	1/1	1/1	0/0	1/1	1/1	1/1	1/1	1/1	1/1	1/1	1/1	0/0	1/1	1/1	1/1	0/0	1/1
1/1	1/1	1/1	1/1	1/1	1/1	1/1	1/1	1/1	1/1	1/1	1/1	1/1	1/1	1/1	1/1	1/1	1/1	1/1	1/1	1/1	0/1	1/1	1/1	1/1	1/1	1/1
1/1	1/1	1/1	1/1	1/1	1/1	1/1	1/1	1/1	1/1	1/1	1/1	1/1	1/1	1/1	1/1	1/1	1/1	1/1	1/1	1/1	1/1	1/1	1/1	1/1	1/1	1/1
0/0	0/0	0/0	0/0	0/0	0/0	0/0	0/0	0/0	0/0	0/0	0/0	0/0	0/0	0/0	0/0	0/0	0/0	0/0	0/0	0/0	0/0	0/0	0/0	0/0	0/0	0/0
0/0	1/1	1/1	0/0	1/1	0/0	0/0	0/0	0/0	0/0	1/1	0/0	0/0	1/1	1/1	0/0	0/0	1/1	0/0	0/0	0/0	0/0	0/0	1/1	1/1	0/0	1/1
1/1	1/1	1/1	0/0	1/1	0/0	1/1	0/0	0/0	0/0	1/1	0/0	1/1	1/1	1/1	0/0	0/0	1/1	0/0	0/0	0/0	0/0	0/0	1/1	1/1	1/1	1/1
1/1	1/1	1/1	1/1	1/1	1/1	1/1	1/1	1/1	1/1	1/1	1/1	1/1	1/1	1/1	1/1	1/1	1/1	1/1	1/1	1/1	0/1	1/1	1/1	1/1	1/1	1/1
0/0	0/0	0/0	0/0	0/0	0/0	0/0	0/0	0/0	0/0	0/0	0/0	0/0	0/0	0/0	0/0	0/0	0/0	0/0	0/0	0/0	0/0	0/0	0/0	0/0	0/0	0/0
0/0	0/0	0/0	0/0	0/0	0/0	0/0	0/0	0/0	0/0	0/0	0/0	0/0	0/0	0/0	0/0	0/0	0/0	0/0	0/0	0/0	0/0	0/0	0/0	0/0	0/0	0/0
0/0	0/0	0/0	0/0	0/0	0/0	0/0	0/0	0/0	0/0	0/0	0/0	0/0	0/0	0/0	0/0	0/0	0/0	0/0	0/0	0/0	0/0	0/0	0/0	0/0	0/0	0/0
1/1	1/1	1/1	1/1	1/1	1/1	1/1	1/1	1/1	1/1	1/1	1/1	1/1	1/1	1/1	1/1	1/1	1/1	1/1	1/1	1/1	0/1	1/1	1/1	1/1	1/1	1/1
0/0	0/0	0/0	0/0	0/0	0/0	0/0	0/0	0/0	0/0	0/0	0/0	0/0	0/0	0/0	0/0	0/0	0/0	0/0	0/0	0/0	0/0	0/0	0/0	0/0	0/0	0/0
0/0	0/0	0/0	0/0	0/0	0/0	0/0	0/0	0/0	0/0	0/0	0/0	0/0	0/0	0/0	0/0	0/0	0/0	0/0	0/0	0/0	0/0	0/0	0/0	0/0	0/0	0/0
0/0	0/0	0/0	0/0	0/0	0/0	0/0	0/0	0/0	0/0	0/0	0/0	0/0	0/0	0/0	0/0	0/0	0/0	0/0	0/0	0/0	0/0	0/0	0/0	0/0	0/0	0/0
0/0	0/0	0/0	0/0	0/0	0/0	0/0	0/0	0/0	0/0	0/0	0/0	0/0	0/0	0/0	0/0	0/0	0/0	0/0	0/0	0/0	0/0	0/0	0/0	0/0	0/0	0/0
0/0	0/0	0/0	0/0	0/0	0/0	0/0	0/0	0/0	0/0	0/0	0/0	0/0	0/0	0/0	0/0	0/0	0/0	0/0	0/0	0/0	0/0	0/0	0/0	0/0	0/0	0/0
0/0	0/0	0/0	0/0	0/0	0/0	0/0	0/0	0/0	0/0	0/0	0/0	0/0	0/0	0/0	0/0	0/0	0/0	0/0	0/0	0/0	0/0	0/0	0/0	0/0	0/0	0/0
0/0	0/0	0/0	0/0	0/0	0/0	0/0	0/0	0/0	0/0	0/0	0/0	0/0	0/0	0/0	0/0	0/0	0/0	0/0	0/0	0/0	0/0	0/0	0/0	0/0	0/0	0/0
0/0	0/0	0/0	0/0	0/0	0/0	0/0	0/0	0/0	0/0	0/0	0/0	0/0	0/0	0/0	0/0	0/0	0/0	0/0	0/0	0/0	0/0	0/0	0/0	0/0	0/0	0/0
0/0	0/0	0/0	1/1	0/0	1/1	0/0	1/1	1/1	1/1	0/0	1/1	0/0	0/0	0/0	1/1	1/1	0/0	1/1	1/1	1/1	0/1	1/1	0/0	0/0	0/0	0/0
0/0	0/0	0/0	0/0	0/0	0/0	0/0	0/0	0/0	0/0	0/0	0/0	0/0	0/0	0/0	0/0	0/0	0/0	0/0	0/0	0/0	0/0	0/0	0/0	0/0	0/0	0/0
0/0	0/0	0/0	0/0	0/0	0/0	0/0	0/0	0/0	0/0	0/0	0/0	0/0	0/0	0/0	0/0	0/0	0/0	0/0	0/0	0/0	0/0	0/0	0/0	0/0	0/0	0/0
0/0	1/1	1/1	0/0	1/1	0/0	0/0	0/0	0/0	0/0	1/1	0/0	0/0	1/1	1/1	0/0	0/0	0/0	0/0	0/0	0/0	0/0	0/0	1/1	1/1	0/0	1/1

1/1	0/0	0/0	0/0	0/0	0/0	1/1	0/0	0/0	0/0	0/0	0/0	1/1	0/0	0/0	0/0	0/0	0/0	0/0	0/0	0/0	0/0	0/0	0/0	0/0	1/1	0/0
0/4	2/2	2/2	2/2	1/2	1/2	4/4	1/1	2/2	1/1	2/2	1/1	0/4	2/3	1/2	1/1	2/2	1/2	2/2	1/1	1/1	2/3	2/3	2/2	2/2	4/4	2/3
./.	./.	./.	./.	./.	./.	./.	./.	./.	./.	./.	./.	./.	./.	./.	./.	./.	./.	./.	./.	./.	./.	./.	./.	./.	./.	./.
0/0	0/0	0/0	0/0	0/0	0/0	0/0	0/0	0/0	0/0	0/0	0/0	0/0	0/0	0/0	0/0	0/0	0/0	0/0	0/0	0/0	0/0	0/0	0/0	0/0	0/0	0/0
0/0	0/0	0/0	0/0	0/0	0/0	0/0	0/0	0/0	0/0	0/0	0/0	0/0	0/0	0/0	0/0	0/0	0/0	0/0	0/0	0/0	0/0	0/0	0/0	0/0	0/0	0/0
0/0	0/0	0/0	0/0	0/0	0/0	0/0	0/0	0/0	0/0	0/0	0/0	0/0	0/0	0/0	0/0	0/0	0/0	0/0	0/0	0/0	0/0	0/0	0/0	0/0	0/0	0/0
1/1	0/0	0/0	0/0	0/0	0/0	1/1	0/0	0/0	0/0	0/0	0/0	1/1	0/0	0/0	0/0	0/0	0/0	0/0	0/0	0/0	0/0	0/0	0/0	0/0	1/1	0/0
1/1	0/0	0/0	./.	0/0	0/0	1/1	0/0	0/0	0/0	0/0	0/0	1/1	0/0	0/0	0/0	0/0	0/0	0/0	0/0	./.	0/0	0/0	./.	0/0	1/1	0/0
./.	0/0	0/0	./.	0/0	0/0	./.	0/0	0/0	./.	0/0	./.	0/0	0/0	0/0	0/0	0/0	0/0	./.	0/0	./.	0/0	0/0	./.	0/0	0/0	0/0
1/1	1/1	1/1	./.	1/1	./.	1/1	0/0	0/0	./.	0/1	./.	1/1	1/1	1/1	./.	0/0	1/1	./.	./.	./.	0/0	./.	1/1	1/1	1/1	1/1
./.	./.	./.	2/2	0/0	2/2	./.	2/2	2/2	2/2	./.	2/2	./.	./.	./.	2/2	2/2	./.	2/2	2/2	2/2	1/2	2/2	./.	./.	./.	./.
0/0	./.	0/0	./.	0/0	./.	0/0	./.	./.	./.	0/1	./.	./.	0/0	0/0	./.	./.	0/0	./.	./.	./.	2/2	./.	0/0	0/0	0/0	0/0
0/0	0/0	0/0	0/0	0/0	0/0	0/0	0/0	0/0	0/0	0/0	0/0	0/0	0/0	0/0	0/0	0/0	0/0	0/0	0/0	0/0	0/0	0/0	0/0	0/0	0/0	0/0
0/0	0/0	0/0	0/0	0/0	0/0	0/0	0/0	0/0	0/0	0/0	0/0	0/0	0/0	0/0	0/0	0/0	0/0	0/0	0/0	0/0	0/0	0/0	0/0	0/0	0/0	0/0
0/0	0/0	0/0	0/0	0/0	0/0	0/0	0/0	0/0	0/0	0/0	0/0	0/0	0/0	0/0	0/0	0/0	0/0	0/0	0/0	0/0	0/0	0/0	0/0	0/0	0/0	0/0
1/1	1/1	1/1	1/1	1/1	1/1	1/1	1/1	1/1	1/1	1/1	1/1	1/1	1/1	1/1	1/1	1/1	1/1	1/1	1/1	1/1	0/1	1/1	1/1	1/1	1/1	1/1
0/0	0/0	0/0	0/0	0/0	0/0	0/0	0/0	0/0	0/0	0/0	0/0	0/0	0/0	0/0	0/0	0/0	0/0	0/0	0/0	0/0	0/0	0/0	0/0	0/0	0/0	0/0
0/0	1/1	1/1	0/0	1/1	0/0	0/0	0/0	0/0	0/0	1/1	0/0	0/0	1/1	1/1	0/0	0/0	1/1	0/0	0/0	0/0	0/0	0/0	1/1	1/1	0/0	1/1
0/0	0/0	0/0	0/0	0/0	0/0	0/0	0/0	0/0	0/0	0/0	0/0	0/0	0/0	0/0	0/0	0/0	0/0	0/0	0/0	0/0	0/0	0/0	0/0	0/0	0/0	0/0
0/0	0/0	0/0	0/0	0/0	0/0	0/0	0/0	0/0	0/0	0/0	0/0	0/0	0/0	0/0	0/0	0/0	0/0	0/0	0/0	0/0	0/0	0/0	0/0	0/0	0/0	0/0
0/0	0/0	0/0	0/0	0/0	0/0	0/0	0/0	0/0	0/0	0/0	0/0	0/0	0/0	0/0	0/0	0/0	0/0	0/0	0/0	0/0	0/0	0/0	0/0	0/0	0/0	0/0

0/0	0/0	0/0	0/0	0/0	0/0	0/0	0/0	0/0	0/0	0/0	0/0	0/0	0/0	0/0	0/0	0/0	0/0	0/0	0/0	0/0	0/0	0/0	0/0	0/0	0/0	0/0
0/0	0/0	0/0	0/0	0/0	0/0	0/0	0/0	0/0	0/0	0/0	0/0	0/0	0/0	0/0	0/0	0/0	0/0	0/0	0/0	0/0	0/0	0/0	0/0	0/0	0/0	0/0
1/1	1/1	1/1	2/2	1/1	2/2	1/1	2/2	2/2	2/2	1/1	2/2	1/1	1/1	1/1	2/2	2/2	1/1	2/2	2/2	2/2	0/2	2/2	1/1	1/1	1/1	1/1
0/0	0/0	0/0	0/0	0/0	0/0	0/0	0/0	0/0	0/0	0/0	0/0	0/0	0/0	0/0	0/0	0/0	0/0	0/0	0/0	0/0	0/0	0/0	0/0	0/0	0/0	0/0
0/0	1/1	1/1	2/2	1/1	2/2	0/0	2/2	2/2	2/2	1/1	2/2	./.	1/1	1/1	2/2	2/2	1/1	2/2	2/2	2/2	0/2	2/2	1/1	1/1	0/0	1/1
0/0	0/0	0/0	0/0	0/0	0/0	0/0	0/0	0/0	0/0	0/0	0/0	./.	0/0	0/0	0/0	0/0	0/0	0/0	0/0	0/0	0/0	0/0	0/0	0/0	0/0	0/0
1/1	0/0	0/0	0/0	0/0	0/0	1/1	0/0	0/0	0/0	0/0	0/0	1/1	0/0	0/0	0/0	0/0	0/0	0/0	0/0	0/0	0/0	0/0	0/0	0/0	1/1	0/0
1/1	1/1	1/1	1/1	1/1	1/1	1/1	1/1	1/1	1/1	1/1	1/1	1/1	1/1	1/1	1/1	1/1	1/1	1/1	1/1	1/1	0/1	1/1	1/1	1/1	1/1	1/1
1/1	1/1	1/1	1/1	1/1	1/1	1/1	1/1	1/1	1/1	1/1	1/1	1/1	1/1	1/1	1/1	1/1	1/1	1/1	1/1	1/1	0/1	1/1	1/1	1/1	1/1	1/1
0/0	0/0	0/0	0/0	0/0	0/0	./.	0/0	0/0	0/0	0/0	0/0	0/0	0/0	0/0	0/0	0/0	0/0	0/0	0/0	0/0	0/0	0/0	0/0	0/0	0/0	0/0
2/2	1/1	1/1	2/2	1/1	2/2	2/2	2/2	2/2	2/2	1/1	2/2	2/2	1/1	1/1	2/2	2/2	1/1	2/2	2/2	2/2	0/2	2/2	1/1	1/1	2/2	1/1
0/0	0/0	0/0	0/0	0/0	0/0	0/0	0/0	0/0	0/0	0/0	0/0	0/1	0/0	0/0	0/0	0/0	0/0	0/0	0/0	0/0	0/0	0/0	0/0	0/0	0/0	0/0
0/0	0/0	0/0	0/0	0/0	0/0	0/0	0/0	0/0	0/0	0/0	0/0	0/0	0/0	0/0	0/0	0/0	0/0	0/0	0/0	0/0	0/0	0/0	0/0	0/0	0/0	0/0
0/0	0/0	0/0	0/0	0/0	0/0	0/0	0/0	0/0	0/0	0/0	0/0	0/0	0/0	0/0	0/0	0/0	0/0	0/0	0/0	0/0	0/0	0/0	0/0	0/0	0/0	0/0
1/1	0/0	0/0	0/0	0/0	0/0	1/1	0/0	0/0	0/0	0/0	0/0	1/1	0/0	0/0	0/0	0/0	0/0	0/0	0/0	0/0	0/0	0/0	0/0	0/0	1/1	0/0
0/0	0/0	0/0	0/0	0/0	0/0	0/0	0/0	0/0	0/0	0/0	0/0	0/0	0/0	0/0	0/0	0/0	0/0	0/0	0/0	0/0	0/0	0/0	0/0	0/0	0/0	0/0
1/1	0/0	0/0	0/0	0/0	0/0	1/1	0/0	0/0	0/0	0/0	0/0	1/1	0/0	0/0	0/0	0/0	0/0	0/0	0/0	0/0	0/0	0/0	0/0	0/0	1/1	0/0
1/1	1/1	1/1	1/1	1/1	1/1	1/1	1/1	1/1	1/1	1/1	1/1	1/1	1/1	1/1	1/1	1/1	1/1	1/1	1/1	1/1	0/1	1/1	1/1	1/1	1/1	1/1

527	528	532	539	542	546	547	548	549	551	555	556	558	561	562	564	565	566	56	571	572	576	578	581	583	585	589
0/0	0/0	0/0	0/0	0/0	0/0	0/0	0/0	0/0	0/0	0/0	0/0	0/0	0/0	0/0	./.	0/0	0/0	0/0	0/0	0/0	0/0	0/0	0/0	0/0	0/0	0/0
1/1	1/1	1/1	0/0	1/1	0/0	0/0	0/0	0/0	0/0	0/0	0/0	1/1	1/1	0/0	./.	0/0	0/1	0/0	0/0	0/0	0/0	0/0	0/0	0/0	0/0	1/1
0/0	0/0	0/0	0/0	0/0	0/0	0/0	0/0	0/0	0/0	0/0	0/0	0/0	0/0	0/0	./.	0/0	0/0	0/0	0/0	0/0	0/0	0/0	0/0	0/0	0/0	0/0
0/0	0/0	0/0	0/0	0/0	0/0	0/0	0/0	0/0	0/0	0/0	0/0	0/0	0/0	0/0	./.	0/0	0/0	0/0	0/0	0/0	0/0	1/1	0/0	0/0	0/0	0/0
0/0	0/0	0/0	1/1	0/0	0/0	0/0	1/1	1/1	0/0	0/0	0/0	0/0	0/0	1/1	./.	1/1	0/1	1/1	1/1	1/1	1/1	1/1	1/1	0/0	1/1	0/0
0/0	0/0	0/0	1/1	0/0	0/0	0/0	1/1	1/1	0/0	0/0	0/0	0/0	0/0	1/1	./.	1/1	1/1	1/1	1/1	1/1	1/1	1/1	1/1	0/0	1/1	0/0
0/0	0/0	0/0	0/0	0/0	0/0	0/0	0/0	0/0	0/0	0/0	0/0	0/0	0/0	0/0	./.	0/0	0/0	0/0	0/0	0/0	0/0	0/0	0/0	0/0	0/0	0/0
1/1	1/1	1/1	0/0	1/1	1/1	1/1	0/0	0/0	1/1	1/1	1/1	1/1	1/1	0/0	./.	0/0	0/0	0/0	0/0	0/0	0/0	0/0	0/0	1/1	0/0	1/1
0/0	0/0	0/0	0/0	0/0	0/0	0/0	0/0	0/0	0/0	0/0	0/0	0/0	0/0	0/0	./.	0/0	0/0	0/0	0/0	0/0	0/0	0/0	0/0	0/0	0/0	0/0
0/0	0/0	0/0	0/0	0/0	0/0	0/0	0/0	0/0	0/0	0/0	0/0	0/0	0/0	0/0	./.	0/0	0/0	0/0	0/0	0/0	0/1	0/0	0/0	0/0	0/0	0/0
0/0	0/0	0/0	0/0	0/0	0/0	0/0	0/0	0/0	0/0	0/0	0/0	0/0	0/0	0/0	./.	0/0	0/0	0/0	0/0	0/0	0/0	0/0	0/0	0/0	0/0	0/0
0/0	0/0	0/0	0/0	0/0	0/0	0/0	0/0	0/0	0/0	0/0	0/0	0/0	0/0	0/0	./.	0/0	0/0	0/0	0/0	0/0	0/0	0/0	0/0	1/1	0/0	0/0
0/0	0/0	0/0	1/1	0/0	0/0	0/0	1/1	1/1	0/0	0/0	0/0	0/0	0/0	1/1	./.	1/1	1/1	1/1	1/1	1/1	1/1	1/1	1/1	0/0	1/1	0/0
1/1	1/1	0/0	0/0	0/1	0/1	0/0	0/0	0/0	0/0	1/1	0/0	./.	0/0	0/0	./.	0/0	0/0	0/0	0/0	0/0	0/0	./.	0/0	0/0	0/0	0/1
0/0	0/0	1/1	0/0	0/1	0/1	1/1	0/0	./.	1/1	0/0	1/1	1/1	1/1	0/0	./.	./.	0/0	0/0	0/0	0/0	0/0	./.	./.	1/1	./.	0/1
0/0	0/0	0/0	0/0	0/0	0/0	0/0	0/0	./.	0/0	0/0	0/0	./.	0/0	0/0	./.	./.	0/0	0/0	0/0	0/0	0/0	./.	./.	0/0	./.	0/0

5/5	5/5	3/3	3/3	3/5	1/5	0/1	3/6	6/6	1/1	5/5	0/1	./.	6/6	0/1	./.	./.	6/6	1/2	6/6	0/1	3/3	./.	./.	0/4	./.	3/5
0/0	0/0	0/0	0/0	0/0	0/0	0/0	0/0	0/0	0/0	0/0	0/0	./.	0/0	0/0	./.	0/0	0/0	0/0	0/0	0/0	0/0	0/0	./.	0/0	0/0	0/0
0/0	0/0	0/0	0/0	0/0	0/0	0/0	0/0	0/0	0/0	0/0	0/0	./.	0/0	0/0	./.	./.	0/1	0/0	./.	0/0	0/0	0/0	./.	0/0	./.	0/0
0/0	0/0	0/0	1/1	0/0	0/0	0/0	1/1	1/1	0/0	0/0	0/0	0/0	0/0	1/1	./.	1/1	1/1	1/1	1/1	1/1	1/1	0/0	1/1	0/0	1/1	0/0
1/1	1/1	1/1	0/0	1/1	1/1	1/1	0/0	0/0	1/1	1/1	1/1	1/1	1/1	0/0	./.	0/0	0/1	0/0	0/0	0/0	0/0	0/0	0/0	1/1	0/0	1/1
0/0	0/0	0/0	0/0	0/0	0/0	0/0	0/0	0/0	0/0	0/0	0/0	0/0	0/0	0/0	./.	0/0	0/0	0/0	0/0	0/0	0/0	1/1	0/0	0/0	0/0	0/0
1/1	1/1	1/1	0/0	1/1	1/1	1/1	0/0	0/0	1/1	1/1	1/1	1/1	1/1	0/0	./.	0/0	0/1	0/0	0/0	0/0	0/0	0/0	0/0	1/1	0/0	1/1
0/0	0/0	0/0	1/1	0/0	0/0	0/0	1/1	1/1	0/0	0/0	0/0	0/0	0/0	1/1	./.	1/1	0/1	1/1	1/1	1/1	1/1	0/0	1/1	0/0	1/1	0/0
0/0	0/0	0/0	0/0	0/0	0/0	0/0	0/0	0/0	0/0	0/0	0/0	0/0	0/0	0/0	./.	0/0	0/0	0/0	0/0	0/0	0/0	0/0	0/0	0/0	0/0	0/0
0/0	0/0	0/0	1/1	0/0	1/1	1/1	1/1	1/1	./.	1/1	1/1	0/0	0/0	1/1	./.	1/1	0/1	1/1	1/1	1/1	1/1	1/1	1/1	1/1	1/1	0/0
0/0	0/0	0/0	0/0	0/0	0/0	0/0	0/0	0/0	0/0	0/0	0/0	0/0	0/0	0/0	./.	0/0	0/0	0/0	0/0	0/0	0/0	0/0	0/0	0/0	0/0	0/0
0/0	0/0	0/0	1/1	0/0	0/0	0/0	1/1	1/1	0/0	0/0	0/0	0/0	0/0	1/1	./.	1/1	0/1	1/1	1/1	1/1	1/1	0/0	1/1	0/0	1/1	0/0
1/1	1/1	1/1	1/1	1/1	1/1	1/1	1/1	1/1	1/1	1/1	1/1	1/1	1/1	1/1	./.	1/1	1/1	1/1	1/1	1/1	1/1	1/1	1/1	1/1	1/1	1/1
0/0	0/0	0/0	0/0	0/0	0/0	0/0	0/0	0/0	0/0	0/0	0/0	0/0	0/0	0/0	./.	0/0	0/0	0/0	0/0	0/0	0/0	1/1	0/0	0/0	0/0	0/0
0/0	0/0	0/0	0/0	0/0	0/0	0/0	0/0	0/0	0/0	0/0	0/0	0/0	0/0	0/0	./.	0/0	0/0	0/0	0/0	0/0	0/0	0/0	0/0	0/0	0/0	0/0
1/1	1/1	1/1	1/1	1/1	1/1	1/1	1/1	1/1	1/1	1/1	1/1	1/1	1/1	1/1	./.	1/1	1/1	1/1	1/1	1/1	1/1	1/1	1/1	1/1	1/1	1/1
0/0	0/0	0/0	1/1	0/0	0/0	0/0	1/1	1/1	0/0	0/0	0/0	0/0	0/0	1/1	./.	1/1	1/1	1/1	1/1	1/1	1/1	0/0	1/1	0/0	1/1	0/0
0/0	0/0	0/0	0/0	0/0	0/0	0/0	0/0	0/0	0/0	0/0	0/0	0/0	0/0	0/0	./.	0/0	0/0	0/0	0/0	0/0	0/0	1/1	0/0	0/0	0/0	0/0
1/1	1/1	1/1	0/0	1/1	1/1	1/1	0/0	0/0	1/1	1/1	1/1	1/1	1/1	0/0	./.	0/0	0/0	0/0	0/0	0/0	0/0	0/0	0/0	1/1	0/0	1/1
1/1	1/1	1/1	0/0	1/1	0/0	0/0	0/0	0/0	0/0	0/0	0/0	1/1	1/1	0/0	./.	0/0	0/0	0/0	0/0	0/0	0/0	0/0	0/0	0/0	0/0	1/1
0/0	0/0	0/0	0/0	0/0	0/0	0/0	0/0	0/0	0/0	0/0	0/0	0/0	0/0	0/0	./.	0/0	0/0	0/0	0/0	0/0	0/0	1/1	0/0	0/0	0/0	0/0
1/1	1/1	1/1	0/0	1/1	1/1	1/1	0/0	0/0	1/1	1/1	1/1	1/1	1/1	0/0	./.	0/0	0/0	0/0	0/0	0/0	0/0	0/0	0/0	1/1	0/0	1/1
0/0	0/0	0/0	0/0	0/0	0/0	0/0	0/0	0/0	0/0	0/0	0/0	0/0	0/0	0/0	./.	0/0	0/0	0/0	0/0	0/0	0/0	1/1	0/0	0/0	0/0	0/0
1/1	1/1	1/1	1/1	1/1	1/1	1/1	1/1	1/1	1/1	1/1	1/1	1/1	1/1	1/1	./.	1/1	1/1	1/1	1/1	1/1	1/1	1/1	1/1	1/1	1/1	1/1
0/0	0/0	0/0	1/1	0/0	0/0	0/0	1/1	1/1	0/0	0/0	0/0	0/0	0/0	1/1	./.	1/1	0/1	1/1	1/1	1/1	1/1	0/0	1/1	0/0	1/1	0/0
1/1	1/2	1/1	0/0	1/1	0/0	0/0	0/0	0/0	0/0	0/0	0/0	1/1	1/1	0/0	./.	0/0	0/1	0/0	0/0	0/0	0/0	2/2	0/0	0/0	0/0	1/1

1/1	1/1	1/1	1/1	1/1	1/1	1/1	1/1	1/1	1/1	1/1	1/1	1/1	1/1	1/1	./.	1/1	1/1	1/1	1/1	1/1	1/1	1/1	1/1	1/1	1/1	1/1
0/0	0/0	0/0	1/1	0/0	0/0	0/0	1/1	1/1	0/0	0/0	0/0	0/0	0/0	1/1	./.	1/1	1/1	1/1	1/1	1/1	1/1	1/1	1/1	0/0	1/1	0/0
0/0	0/0	0/0	1/1	0/0	0/0	0/0	1/1	1/1	0/0	0/0	0/0	0/0	0/0	1/1	./.	1/1	1/1	1/1	1/1	1/1	1/1	0/0	1/1	0/0	1/1	0/0
0/0	0/0	0/0	0/0	0/0	0/0	0/0	0/0	0/0	0/0	0/0	0/0	0/0	0/0	0/0	./.	0/0	0/0	0/0	0/0	0/0	0/0	0/0	0/0	0/0	0/0	0/0
1/1	1/1	1/1	1/1	1/1	1/1	1/1	1/1	1/1	1/1	1/1	1/1	1/1	1/1	1/1	./.	1/1	1/1	1/1	1/1	1/1	1/1	1/1	1/1	1/1	1/1	1/1
1/1	1/1	1/1	1/1	1/1	1/1	1/1	1/1	1/1	1/1	1/1	1/1	1/1	1/1	1/1	./.	1/1	1/1	1/1	1/1	1/1	1/1	1/1	1/1	1/1	1/1	1/1
1/1	1/1	1/1	1/1	1/1	1/1	1/1	1/1	1/1	1/1	1/1	1/1	1/1	1/1	1/1	./.	1/1	1/1	1/1	1/1	1/1	1/1	1/1	1/1	1/1	1/1	1/1
0/0	0/0	0/0	./.	0/0	0/0	0/0	0/0	0/0	0/0	0/0	0/0	0/0	0/0	0/0	./.	0/0	0/0	0/0	0/0	0/0	0/0	1/1	0/0	0/0	0/0	0/0
0/0	0/0	0/0	1/1	0/0	0/0	0/0	1/1	1/1	0/0	0/0	0/0	0/0	0/0	1/1	./.	1/1	0/1	1/1	1/1	1/1	1/1	0/0	1/1	0/0	1/1	0/0
0/0	0/0	0/0	0/0	0/0	0/0	0/0	0/0	0/0	0/0	0/0	0/0	0/0	0/0	0/0	./.	0/0	0/0	0/0	0/0	0/0	0/0	0/0	0/0	0/0	0/0	0/0
1/1	1/1	1/1	0/0	1/1	1/1	1/1	0/0	0/0	1/1	1/1	1/1	1/1	1/1	0/0	./.	0/0	0/0	0/0	0/0	0/0	0/0	0/0	0/0	1/1	0/0	1/1
0/0	0/0	0/0	0/0	0/0	0/0	0/0	0/0	0/0	0/0	0/0	0/0	0/0	0/0	0/0	./.	0/0	0/0	0/0	0/0	0/0	0/0	0/0	0/0	0/0	0/0	0/0
0/0	0/0	0/0	0/0	0/0	0/0	0/0	0/0	0/0	0/0	0/0	0/0	0/0	0/0	0/0	./.	0/0	0/0	0/0	0/0	0/0	0/0	0/0	0/0	0/0	0/0	0/0
0/0	0/0	0/0	0/0	0/0	1/1	1/1	0/0	0/0	1/1	1/1	1/1	0/0	0/0	0/0	./.	0/0	0/0	0/0	0/0	0/0	0/0	0/0	0/0	1/1	0/0	0/0
1/1	1/1	1/1	1/1	1/1	1/1	1/1	1/1	1/1	1/1	1/1	1/1	1/1	1/1	1/1	./.	1/1	1/1	1/1	1/1	1/1	1/1	1/1	1/1	1/1	1/1	1/1
0/0	0/0	0/0	0/0	0/0	0/0	0/0	0/0	0/0	0/0	0/0	0/0	0/0	0/0	0/0	./.	0/0	0/0	0/0	0/0	0/0	0/0	1/1	0/0	0/0	0/0	0/0
0/0	0/0	0/0	0/0	0/0	0/0	0/0	0/0	0/0	0/0	0/0	0/0	0/0	0/0	0/0	./.	0/0	0/0	0/0	0/0	0/0	0/0	0/0	0/0	0/0	0/0	0/0
0/0	0/0	0/0	0/0	0/0	0/0	0/0	0/0	0/0	0/0	0/0	0/0	0/0	0/0	0/0	./.	0/0	0/0	0/0	0/0	0/0	0/0	0/0	0/0	0/0	1/1	0/0
1/1	1/1	1/1	0/0	1/1	1/1	1/1	0/0	0/0	1/1	1/1	1/1	1/1	1/1	0/0	./.	0/0	0/1	0/0	0/0	0/0	0/0	0/0	0/0	1/1	0/0	1/1
0/0	0/0	0/0	0/0	0/0	0/0	0/0	0/0	0/0	0/0	0/0	0/0	0/0	0/0	0/0	./.	0/0	0/0	0/0	0/0	0/0	0/0	1/1	0/0	0/0	0/0	0/0
1/1	1/1	1/1	1/1	1/1	1/1	1/1	1/1	1/1	1/1	1/1	1/1	1/1	1/1	1/1	./.	1/1	1/1	1/1	1/1	1/1	1/1	1/1	1/1	1/1	1/1	1/1
1/1	1/1	1/1	0/0	1/1	1/1	1/1	0/0	0/0	1/1	1/1	1/1	1/1	1/1	0/0	./.	0/0	0/1	0/0	0/0	0/0	0/0	0/0	0/0	1/1	0/0	1/1
1/1	1/1	1/1	0/0	1/1	1/1	1/1	0/0	0/0	1/1	1/1	1/1	1/1	1/1	0/0	./.	0/0	0/1	0/0	0/0	0/0	0/0	0/0	0/0	1/1	0/0	1/1
0/0	0/0	0/0	1/1	0/0	0/0	0/0	1/1	1/1	0/0	0/0	0/0	0/0	0/0	1/1	./.	1/1	0/1	1/1	1/1	1/1	1/1	1/1	1/1	0/0	1/1	0/0
1/1	1/1	1/1	1/1	1/1	1/1	1/1	1/1	1/1	1/1	1/1	1/1	1/1	1/1	1/1	./.	1/1	1/1	1/1	1/1	1/1	1/1	1/1	1/1	1/1	1/1	1/1
0/0	0/0	0/0	1/1	0/0	0/0	0/0	1/1	1/1	0/0	0/0	0/0	0/0	0/0	1/1	./.	1/1	1/1	1/1	1/1	1/1	1/1	1/1	1/1	0/0	1/1	0/0
0/0	0/0	0/0	0/0	0/0	0/0	0/0	0/0	0/0	0/0	0/0	0/0	0/0	0/0	0/0	./.	0/0	0/0	0/0	0/0	0/0	0/0	0/0	0/0	1/1	0/0	0/0
0/0	0/0	0/0	0/0	0/0	0/0	0/0	0/0	0/0	0/0	0/0	0/0	0/0	0/0	0/0	./.	0/0	0/0	0/0	0/0	0/0	0/0	1/1	0/0	0/0	0/0	0/0
1/1	1/1	1/1	1/1	1/1	1/1	1/1	1/1	1/1	1/1	1/1	1/1	1/1	1/1	1/1	./.	1/1	1/1	1/1	1/1	1/1	1/1	1/1	1/1	1/1	1/1	1/1
0/0	0/0	0/0	0/0	./.	0/0	0/0	0/0	0/0	0/0	0/0	0/0	0/0	0/0	0/0	./.	0/0	0/0	0/0	0/0	0/0	0/0	0/0	0/0	./.	0/0	0/0
1/1	./.	1/1	1/1	./.	1/1	1/1	1/1	1/1	1/1	1/1	1/1	1/1	1/1	1/1	./.	1/1	1/1	1/1	1/1	1/1	1/1	1/1	1/1	1/1	1/1	1/1
0/0	0/0	0/0	1/1	0/0	0/0	0/0	1/1	1/1	0/0	0/0	0/0	0/0	0/0	1/1	./.	1/1	1/1	1/1	1/1	1/1	1/1	0/0	1/1	0/0	1/1	0/0
1/1	./.	1/1	1/1	./.	1/1	1/1	1/1	1/1	1/1	1/1	1/1	1/1	1/1	1/1	./.	1/1	1/1	1/1	1/1	1/1	1/1	1/1	1/1	1/1	1/1	1/1
0/0	0/0	0/0	0/0	0/0	0/0	0/0	0/0	0/0	0/0	0/0	0/0	0/0	0/0	0/0	./.	0/0	0/0	0/0	0/0	0/0	0/0	0/0	0/0	0/0	0/0	0/0
0/0	0/0	0/0	1/1	0/0	0/0	0/0	1/1	1/1	0/0	0/0	0/0	0/0	0/0	1/1	./.	1/1	1/1	1/1	1/1	1/1	1/1	1/1	1/1	0/0	1/1	0/0

1/1	./.	1/1	0/0	./.	1/1	1/1	0/0	0/0	1/1	1/1	1/1	1/1	1/1	0/0	./.	0/0	0/0	0/0	0/0	0/0	0/0	0/0	0/0	1/1	0/0	1/1
0/0	0/0	0/0	0/0	0/0	0/0	0/0	0/0	0/0	0/0	0/0	0/0	0/0	0/0	0/0	./.	0/0	0/0	0/0	0/0	0/0	0/0	0/0	0/0	0/0	0/0	0/0
1/1	./.	1/1	0/0	./.	0/0	0/0	0/0	0/0	0/0	0/0	0/0	1/1	1/1	0/0	./.	0/0	0/0	0/0	0/0	0/0	0/0	0/0	0/0	0/0	0/0	1/1
1/1	./.	1/1	1/1	./.	1/1	1/1	1/1	1/1	1/1	1/1	1/1	1/1	1/1	1/1	./.	1/1	1/1	1/1	1/1	1/1	1/1	1/1	1/1	1/1	1/1	1/1
0/0	0/0	0/0	0/0	0/0	0/0	0/0	0/0	0/0	0/0	0/0	0/0	0/0	0/0	0/0	./.	0/0	0/0	0/0	0/0	0/0	0/0	0/0	0/0	0/0	0/0	0/0
1/1	1/1	1/1	0/0	1/1	1/1	1/1	0/0	0/0	1/1	1/1	1/1	1/1	1/1	0/0	./.	0/0	0/0	0/0	0/0	0/0	0/0	0/0	0/0	1/1	0/0	1/1
1/1	1/1	1/1	1/1	1/1	1/1	1/1	1/1	1/1	1/1	1/1	1/1	1/1	1/1	1/1	./.	1/1	1/1	1/1	1/1	1/1	1/1	1/1	1/1	1/1	1/1	1/1
0/0	./.	0/0	1/1	0/0	0/0	0/0	1/1	1/1	./.	0/0	0/0	0/0	0/0	1/1	./.	1/1	1/1	1/1	1/1	1/1	1/1	0/0	1/1	./.	1/1	./.
1/1	1/1	1/1	0/0	1/1	1/1	1/1	0/0	0/0	1/1	1/1	1/1	1/1	1/1	0/0	./.	0/0	0/0	0/0	0/0	0/0	0/0	0/0	0/0	1/1	0/0	1/1
0/0	0/0	0/0	0/0	0/0	0/0	0/0	0/0	0/0	0/0	0/0	0/0	0/0	0/0	0/0	./.	0/0	0/0	0/0	0/0	0/0	0/0	0/0	0/0	0/0	0/0	0/0
1/1	1/1	1/1	0/0	1/1	1/1	1/1	0/0	0/0	1/1	1/1	1/1	1/1	1/1	0/0	./.	0/0	0/0	0/0	0/0	0/0	0/0	0/0	0/0	1/1	0/0	1/1
0/0	0/0	0/0	0/0	0/0	0/0	0/0	0/0	0/0	0/0	0/0	0/0	0/0	0/0	0/0	./.	0/0	0/0	0/0	0/0	0/0	0/0	1/1	0/0	0/0	0/0	0/0
0/0	0/0	0/0	0/0	0/0	0/0	0/0	0/0	0/0	0/0	0/0	0/0	0/0	0/0	0/0	./.	0/0	0/0	0/0	0/0	0/0	0/0	1/1	0/0	0/0	0/0	0/0
0/0	0/0	0/0	0/0	0/0	0/0	0/0	0/0	0/0	0/0	0/0	0/0	0/0	0/0	0/0	./.	0/0	0/0	0/0	0/0	0/0	0/0	0/0	0/0	0/0	0/0	0/0
1/1	1/1	1/1	1/1	1/1	1/1	1/1	1/1	1/1	1/1	1/1	1/1	1/1	1/1	1/1	./.	1/1	1/1	1/1	1/1	1/1	1/1	1/1	1/1	1/1	1/1	1/1
1/1	1/1	1/1	1/1	1/1	1/1	1/1	1/1	1/1	1/1	1/1	1/1	1/1	1/1	1/1	./.	1/1	1/1	1/1	1/1	1/1	1/1	1/1	1/1	1/1	1/1	1/1
1/1	1/1	1/1	1/1	1/1	1/1	1/1	1/1	1/1	1/1	1/1	1/1	1/1	1/1	1/1	./.	1/1	1/1	1/1	1/1	1/1	1/1	1/1	1/1	1/1	1/1	1/1
./.	./.	./.	./.	./.	./.	./.	./.	./.	./.	./.	./.	./.	./.	./.	./.	./.	0/0	0/0	./.	./.	./.	./.	./.	./.	./.	./.
1/1	1/1	1/1	3/3	1/1	1/1	1/1	3/3	3/3	1/1	1/1	1/1	1/1	1/1	3/3	./.	3/3	1/3	3/3	3/3	3/3	3/3	3/3	3/3	1/1	3/3	1/1
0/0	0/0	0/0	0/0	0/0	0/0	0/0	0/0	0/0	0/0	0/0	0/0	0/0	0/0	1/1	./.	1/1	0/0	1/1	0/0	1/1	0/0	0/0	0/0	0/0	0/0	0/0
0/0	0/0	0/0	0/0	0/0	0/0	0/0	0/0	0/0	0/0	0/0	0/0	0/0	0/0	0/0	./.	0/0	0/0	0/0	0/0	0/0	0/0	0/0	0/0	0/0	0/0	0/0
0/0	0/0	0/0	0/0	0/0	0/0	0/0	0/0	0/0	0/0	0/0	0/0	0/0	0/0	0/0	./.	0/0	0/0	0/0	0/0	0/0	0/0	0/0	0/0	0/0	0/0	0/0
0/0	0/0	0/0	0/0	0/0	0/0	0/0	0/0	0/0	0/0	0/0	0/0	0/0	0/0	0/0	./.	0/0	0/0	0/0	0/0	0/0	0/0	0/0	0/0	0/0	0/0	0/0
0/0	0/0	0/0	0/0	0/0	0/0	0/0	0/0	0/0	0/0	0/0	0/0	0/0	0/0	0/0	./.	0/0	0/0	0/0	./.	./.	0/0	1/1	./.	0/0	./.	0/0
0/0	0/0	0/0	1/1	0/0	0/0	0/0	1/1	1/1	0/0	0/0	0/0	0/0	0/0	1/1	./.	1/1	1/1	1/1	1/1	1/1	1/1	0/0	1/1	0/0	1/1	0/0

0/0	0/0	0/0	0/0	0/0	0/0	0/0	0/0	0/0	0/0	0/0	0/0	0/0	0/0	0/0	./.	0/0	0/0	0/0	0/0	0/0	0/0	0/0	0/0	0/0	0/0	0/0
0/0	0/0	0/0	1/1	0/0	0/0	0/0	1/1	1/1	0/0	0/0	0/0	0/0	0/0	1/1	./.	1/1	1/1	1/1	1/1	1/1	1/1	0/0	1/1	0/0	1/1	0/0
0/0	0/0	0/0	1/1	0/0	0/0	0/0	1/1	1/1	0/0	0/0	0/0	0/0	0/0	1/1	./.	1/1	0/1	1/1	1/1	1/1	1/1	0/0	1/1	0/0	1/1	0/0
1/1	1/1	1/1	1/1	1/1	1/1	1/1	1/1	1/1	1/1	1/1	1/1	1/1	1/1	1/1	./.	1/1	1/1	1/1	1/1	1/1	1/1	1/1	1/1	1/1	1/1	1/1
1/1	1/1	1/1	1/1	1/1	1/1	1/1	1/1	1/1	1/1	1/1	1/1	1/1	1/1	1/1	./.	1/1	1/1	1/1	1/1	1/1	1/1	1/1	1/1	1/1	1/1	1/1
0/0	0/0	0/0	1/1	0/0	0/0	0/0	0/0	0/0	0/0	0/0	0/0	0/0	0/0	0/0	./.	0/0	0/0	0/0	0/0	0/0	0/0	0/0	0/0	0/0	0/0	0/0
1/1	1/1	1/1	1/1	1/1	1/1	1/1	1/1	1/1	1/1	1/1	1/1	1/1	1/1	1/1	./.	1/1	1/1	1/1	1/1	1/1	1/1	1/1	1/1	1/1	1/1	1/1
0/0	0/0	0/0	1/1	0/0	0/0	0/0	1/1	1/1	0/0	0/0	0/0	0/0	0/0	1/1	./.	1/1	0/1	1/1	1/1	1/1	1/1	0/0	1/1	0/0	1/1	0/0
0/0	0/0	0/0	0/0	0/0	0/0	0/0	0/0	0/0	0/0	0/0	0/0	0/0	0/0	0/0	./.	0/0	0/0	0/0	0/0	0/0	0/0	1/1	0/0	0/0	0/0	0/0
0/0	0/0	0/0	0/0	0/0	0/0	0/0	0/0	0/0	0/0	0/0	0/0	0/0	0/0	0/0	./.	0/0	0/0	0/0	0/0	0/0	0/0	0/0	0/0	0/0	0/0	0/0
0/0	0/0	0/0	1/1	0/0	0/0	0/0	1/1	1/1	0/0	0/0	0/0	0/0	0/0	1/1	./.	1/1	0/1	1/1	1/1	1/1	1/1	0/0	1/1	0/0	1/1	0/0
0/0	0/0	0/0	0/0	0/0	0/0	0/0	0/0	0/0	0/0	0/0	0/0	0/0	0/0	0/0	./.	0/0	0/0	0/0	0/0	0/0	0/0	0/0	0/0	0/0	0/0	0/0
1/1	1/1	1/1	0/0	1/1	1/1	1/1	0/0	0/0	1/1	1/1	1/1	1/1	1/1	0/0	./.	0/0	0/1	0/0	0/0	0/0	0/0	0/0	0/0	1/1	0/0	1/1
0/0	0/0	0/0	1/1	0/0	0/0	0/0	1/1	1/1	0/0	0/0	0/0	0/0	0/0	1/1	./.	1/1	0/1	1/1	1/1	1/1	1/1	0/0	1/1	0/0	1/1	0/0
0/0	0/0	0/0	0/0	0/0	0/0	0/0	0/0	0/0	0/0	0/0	0/0	0/0	0/0	0/0	./.	0/0	0/0	0/0	0/0	0/0	0/0	0/0	0/0	0/0	0/0	0/0
1/1	1/1	1/1	1/1	1/1	1/1	1/1	1/1	1/1	1/1	1/1	1/1	1/1	1/1	1/1	./.	1/1	1/1	1/1	1/1	1/1	1/1	1/1	1/1	1/1	1/1	1/1
1/1	1/1	1/1	0/0	1/1	1/1	1/1	0/0	0/0	1/1	1/1	1/1	1/1	1/1	0/0	./.	0/0	0/1	0/0	0/0	0/0	0/0	0/0	0/0	1/1	0/0	1/1
0/0	0/0	0/0	0/0	0/0	1/1	1/1	0/0	0/0	1/1	1/1	1/1	0/0	0/0	0/0	./.	0/0	0/0	0/0	0/0	0/0	0/0	0/0	0/0	1/1	0/0	0/0
0/0	0/0	0/0	0/0	0/0	0/0	0/0	0/0	0/0	0/0	0/0	0/0	0/0	0/0	0/0	./.	0/0	0/0	0/0	0/0	0/0	0/0	0/0	0/0	0/0	0/0	0/0
0/0	0/0	0/0	1/1	0/0	0/0	0/0	1/1	1/1	0/0	0/0	0/0	0/0	0/0	1/1	./.	1/1	0/1	1/1	1/1	1/1	1/1	0/0	1/1	0/0	1/1	0/0
0/0	0/0	0/0	1/1	0/0	0/0	0/0	1/1	1/1	0/0	0/0	0/0	0/0	0/0	1/1	./.	1/1	0/1	1/1	1/1	1/1	1/1	0/0	1/1	0/0	1/1	0/0
0/0	0/0	0/0	0/0	0/0	0/0	0/0	0/0	1/1	0/0	0/0	0/0	0/0	0/0	0/0	./.	0/0	0/0	0/0	0/0	0/0	0/0	0/0	0/0	0/0	0/0	0/0
1/1	1/1	1/1	1/1	1/1	1/1	1/1	1/1	1/1	1/1	1/1	1/1	1/1	1/1	1/1	./.	1/1	1/1	1/1	1/1	1/1	1/1	1/1	1/1	1/1	1/1	1/1
0/0	0/0	0/0	0/0	0/0	0/0	0/0	0/0	0/0	0/0	0/0	0/0	0/0	0/0	0/0	./.	0/0	0/0	0/0	0/0	0/0	0/0	0/0	0/0	0/0	0/0	0/0
0/0	0/0	0/0	0/0	0/0	0/0	0/0	0/0	0/0	0/0	0/0	0/0	0/0	0/0	0/0	./.	0/0	0/0	0/0	0/0	0/0	0/0	1/1	0/0	0/0	0/0	0/0
0/0	0/0	0/0	1/1	0/0	0/0	0/0	1/1	1/1	0/0	0/0	0/0	0/0	0/0	1/1	./.	1/1	1/1	1/1	1/1	1/1	1/1	0/0	1/1	0/0	1/1	0/0
0/0	0/0	0/0	0/0	0/0	0/0	0/0	0/0	0/0	0/0	0/0	0/0	0/0	0/0	0/0	./.	0/0	0/0	0/0	0/0	0/0	0/0	0/0	0/0	0/0	0/0	0/0
1/1	1/1	1/1	0/0	1/1	1/1	1/1	0/0	0/0	1/1	1/1	1/1	1/1	1/1	0/0	./.	0/0	0/0	0/0	0/0	0/0	0/0	0/0	0/0	1/1	0/0	1/1
0/0	0/0	0/0	1/1	0/0	0/0	0/0	1/1	1/1	0/0	0/0	0/0	0/0	0/0	1/1	./.	1/1	1/1	1/1	1/1	1/1	1/1	0/0	1/1	0/0	1/1	0/0
0/0	0/0	0/0	0/0	0/0	0/0	0/0	0/0	0/0	0/0	0/0	0/0	0/0	0/0	0/0	./.	0/0	0/0	0/0	0/0	0/0	0/0	0/0	0/0	0/0	0/0	0/0
1/1	1/1	1/1	1/1	1/1	1/1	1/1	1/1	1/1	1/1	1/1	1/1	1/1	1/1	1/1	./.	1/1	1/1	1/1	1/1	1/1	1/1	1/1	1/1	1/1	1/1	1/1
0/0	0/0	0/0	0/0	0/0	0/0	0/0	0/0	0/0	0/0	0/0	1/1	0/0	0/0	0/0	./.	0/0	0/0	0/0	0/0	0/0	0/0	0/0	0/0	0/0	0/0	0/0
0/0	0/0	0/0	0/0	0/0	0/0	0/0	0/0	0/0	0/0	0/0	0/0	0/0	0/0	0/0	./.	0/0	0/0	0/0	0/0	0/0	0/0	0/0	0/0	0/0	0/0	0/0

1/1	1/1	1/1	1/1	1/1	1/1	1/1	1/1	1/1	1/1	1/1	1/1	1/1	1/1	1/1	./.	1/1	1/1	1/1	1/1	1/1	1/1	1/1	1/1	1/1	1/1	1/1
0/0	0/0	0/0	0/0	0/0	0/0	0/0	0/0	0/0	0/0	0/0	0/0	0/0	0/0	0/0	./.	0/0	0/0	0/0	0/0	0/0	0/0	0/0	0/0	0/0	0/0	0/0
1/1	1/1	1/1	1/1	1/1	1/1	1/1	1/1	1/1	1/1	1/1	1/1	1/1	1/1	1/1	./.	1/1	1/1	1/1	1/1	1/1	1/1	1/1	1/1	1/1	1/1	1/1
0/0	0/0	0/0	0/0	0/0	0/0	0/0	0/0	0/0	0/0	0/0	0/0	0/0	0/0	0/0	./.	0/0	0/0	0/0	0/0	0/0	0/0	0/0	0/0	0/0	0/0	0/0
1/1	1/1	1/1	0/0	1/1	0/0	0/0	0/0	0/0	0/0	0/0	0/0	1/1	1/1	0/0	./.	0/0	0/0	0/0	0/0	0/0	0/0	0/0	0/0	0/0	0/0	1/1
0/0	0/0	0/0	0/0	0/0	0/0	0/0	0/0	0/0	0/0	0/0	0/0	0/0	0/0	0/0	./.	0/0	0/0	0/0	0/0	0/0	0/0	0/0	0/0	0/0	0/0	0/0
0/0	0/0	0/0	0/0	0/0	0/0	0/0	0/0	0/0	0/0	0/0	0/0	0/0	0/0	0/0	./.	0/0	0/0	0/0	0/0	0/0	0/0	1/1	0/0	0/0	0/0	0/0
0/0	0/0	0/0	0/0	0/0	1/1	1/1	0/0	0/0	1/1	1/1	1/1	0/0	0/0	0/0	./.	0/0	0/0	0/0	0/0	0/0	0/0	0/0	0/0	1/1	0/0	0/0
0/0	0/0	0/0	0/0	0/0	0/0	0/0	0/0	0/0	0/0	0/0	0/0	0/0	0/0	0/0	./.	0/0	0/0	0/0	0/0	0/0	0/0	0/0	0/0	0/0	0/0	0/0
0/0	0/0	0/0	0/0	0/0	0/0	0/0	0/0	0/0	0/0	0/0	0/0	0/0	0/0	0/0	./.	0/0	0/0	0/0	0/0	0/0	0/0	1/1	0/0	0/0	0/0	0/0
1/1	1/1	1/1	1/1	1/1	1/1	1/1	1/1	1/1	1/1	1/1	1/1	1/1	1/1	1/1	./.	1/1	1/1	1/1	1/1	1/1	1/1	1/1	1/1	1/1	1/1	1/1
1/1	1/1	1/1	1/1	1/1	1/1	1/1	1/1	1/1	1/1	1/1	1/1	1/1	1/1	1/1	./.	1/1	1/1	1/1	1/1	1/1	1/1	1/1	1/1	1/1	1/1	1/1
0/0	0/0	0/0	0/0	0/0	0/0	0/0	0/0	0/0	0/0	0/0	0/0	0/0	0/0	0/0	./.	0/0	0/0	0/0	0/0	0/0	0/0	1/1	0/0	0/0	0/0	0/0
1/1	1/1	1/1	0/0	1/1	0/0	0/0	0/0	0/0	0/0	0/0	0/0	1/1	1/1	0/0	./.	0/0	0/0	0/0	0/0	0/0	0/0	0/0	0/0	0/0	0/0	1/1
0/0	0/0	0/0	1/1	0/0	0/0	0/0	1/1	1/1	0/0	0/0	0/0	0/0	0/0	1/1	./.	1/1	0/1	1/1	1/1	1/1	1/1	0/0	1/1	./.	1/1	0/0
1/1	1/1	0/1	1/1	1/1	1/1	1/1	1/1	1/1	1/1	1/1	1/1	1/1	1/1	1/1	./.	1/1	1/1	1/1	1/2	1/1	1/1	0/0	1/1	1/1	1/1	1/1
1/1	1/1	1/1	1/1	1/1	1/1	1/1	1/1	1/1	1/1	1/1	1/1	1/1	1/1	1/1	./.	1/1	1/1	1/1	1/1	1/1	1/1	1/1	1/1	1/1	1/1	1/1
0/0	0/0	0/0	1/1	0/0	1/1	0/0	1/1	1/1	0/0	1/1	1/1	0/0	0/0	1/1	0/0	1/1	0/1	0/0	1/1	1/1	1/1	0/0	1/1	0/0	1/1	0/0
0/0	0/0	0/0	1/1	0/0	1/1	0/0	1/1	1/1	0/0	1/1	0/1	0/0	0/0	1/1	0/0	1/1	0/1	0/0	1/1	1/1	1/1	0/0	1/1	0/0	1/1	0/0
1/1	1/1	1/1	0/0	1/1	0/0	0/0	0/0	0/0	0/0	0/0	0/0	1/1	1/1	0/0	0/0	0/0	0/1	0/0	0/0	0/0	0/0	0/0	0/0	0/0	0/0	1/1
0/0	0/0	0/0	0/0	0/0	0/0	0/0	0/0	0/0	0/0	0/0	0/0	0/0	0/0	0/0	0/0	0/0	0/0	0/0	0/0	0/0	0/0	0/0	0/0	0/0	0/0	0/0
0/0	0/0	0/0	0/0	0/0	0/0	0/0	0/0	0/0	0/0	0/0	0/0	0/0	0/0	0/0	0/0	0/0	0/0	0/0	0/0	0/0	0/0	0/0	0/0	0/0	0/0	0/0
0/0	0/0	0/0	0/0	0/0	0/0	0/0	0/0	0/0	0/0	0/0	0/0	0/1	0/0	0/0	0/0	0/0	0/0	0/0	0/0	0/0	0/0	0/0	0/0	0/0	0/0	0/1
0/0	0/0	0/0	0/0	0/0	0/0	0/0	0/0	0/0	0/0	0/0	0/0	0/0	0/0	0/0	0/0	0/0	0/0	0/0	0/0	0/0	0/0	0/0	0/0	0/0	0/0	0/0
0/0	0/0	0/0	0/0	0/0	0/0	0/0	0/0	0/0	0/0	0/0	0/0	0/1	0/0	0/0	0/0	0/0	0/0	0/0	0/0	0/0	0/0	0/0	0/0	0/1	0/0	0/1
0/0	0/0	0/1	0/0	0/0	0/0	0/0	0/0	0/0	0/0	0/1	0/0	0/1	0/0	0/0	0/0	0/0	0/0	0/0	0/0	0/0	0/0	0/0	0/0	0/1	0/0	0/1

0/0	0/0	0/1	0/0	0/0	0/0	0/0	0/0	0/0	0/0	0/1	0/0	0/1	0/0	0/0	0/0	0/0	0/0	0/0	0/0	0/0	0/0	0/0	0/0	0/0	0/0	0/1
0/0	0/0	0/1	0/0	0/0	0/0	0/0	0/0	0/0	0/0	0/1	0/0	0/1	0/0	0/0	0/0	0/0	0/0	0/0	0/0	0/0	0/0	0/0	0/0	0/0	0/0	0/0
0/0	0/0	0/1	0/0	0/0	0/0	0/0	0/0	0/0	0/0	0/1	0/0	0/1	0/0	0/0	0/0	0/0	0/0	0/0	0/0	0/0	0/0	0/0	0/0	0/0	0/0	0/0
0/0	0/0	0/1	0/0	0/0	0/0	0/0	0/0	0/0	0/0	0/1	0/0	0/1	0/0	0/0	0/0	0/0	0/0	0/0	0/0	0/0	0/0	0/0	0/0	0/0	0/0	0/0
0/0	0/0	0/1	0/0	0/0	0/0	0/0	0/0	0/0	0/0	0/1	0/0	0/1	0/0	0/0	0/0	0/0	0/0	0/0	0/0	0/0	0/0	0/0	0/0	0/0	0/0	0/0
0/0	0/0	0/1	0/0	0/0	0/0	0/0	0/0	0/0	0/0	0/0	0/0	0/1	0/0	0/0	0/0	0/0	0/0	0/0	0/0	0/0	0/0	0/0	0/0	0/0	0/0	0/0
0/0	0/0	0/1	0/0	0/0	0/0	0/0	0/0	0/0	0/0	0/0	0/0	0/0	0/0	0/0	0/0	0/0	0/0	0/0	0/0	0/0	0/0	0/0	0/0	0/0	0/0	0/0
0/0	0/0	0/2	0/0	0/0	0/0	0/0	0/0	0/0	0/0	0/0	0/0	0/1	0/0	0/0	0/0	0/0	0/0	0/0	0/0	0/0	0/0	0/0	0/0	0/0	0/0	0/0
0/0	0/0	0/2	0/0	0/0	0/0	0/0	0/0	0/0	0/0	0/0	0/0	0/1	0/0	0/0	0/0	0/0	0/0	0/0	0/0	0/0	0/0	0/0	0/0	0/0	0/0	0/0
0/0	0/0	0/0	0/0	0/0	0/0	0/0	0/0	0/0	0/0	0/0	0/0	0/0	0/0	0/0	0/0	0/0	0/0	0/0	0/0	0/0	0/0	0/0	0/0	0/0	0/0	0/0
1/1	1/1	1/1	1/1	1/1	1/1	1/1	1/1	1/1	1/1	1/1	1/1	1/1	1/1	1/1	1/1	1/1	1/1	1/1	1/1	1/1	1/1	1/1	1/1	1/1	1/1	1/1
0/0	0/0	0/0	0/0	0/0	0/0	0/0	0/0	0/0	0/0	0/0	0/0	0/0	0/0	0/0	0/0	0/0	0/0	0/0	0/0	0/0	0/0	0/0	0/0	0/0	0/0	0/0
0/0	0/0	0/0	0/0	0/0	0/0	0/0	0/0	0/0	0/0	0/0	0/0	0/0	0/0	0/0	0/0	0/0	0/0	0/0	0/0	0/0	0/0	0/0	0/0	0/0	0/0	0/0
0/0	0/0	0/0	0/0	0/0	0/0	1/1	0/0	0/0	1/1	0/0	0/1	0/0	0/0	0/0	1/1	0/0	0/0	1/1	0/0	0/0	0/0	1/1	0/0	1/1	0/0	0/0
0/0	0/0	0/0	0/0	0/0	0/0	0/0	0/0	0/0	0/0	0/0	0/0	0/0	0/0	0/0	0/0	0/0	0/0	0/0	0/0	0/0	0/0	0/0	0/0	0/0	0/0	0/0
0/0	0/0	0/0	0/0	0/0	0/0	0/0	0/0	0/0	0/0	0/0	0/0	0/0	0/0	0/0	0/0	0/0	0/0	0/0	0/0	0/0	0/0	0/0	0/0	0/0	0/0	0/0
1/1	1/1	1/1	1/1	1/1	1/1	1/1	1/1	1/1	1/1	1/1	1/1	1/1	1/1	1/1	1/1	1/1	1/1	1/1	1/1	1/1	1/1	1/1	1/1	1/1	1/1	1/1
0/0	0/0	0/0	0/0	0/0	0/0	0/0	0/0	0/0	0/0	0/0	0/0	0/0	0/0	./.	./.	0/0	./.	0/0	./.	./.	./.	0/0	./.	0/0	./.	0/0
1/1	1/1	1/1	0/0	1/1	1/1	1/1	./.	0/0	1/1	1/1	1/1	1/1	1/1	./.	1/1	./.	0/1	1/1	./.	./.	./.	1/1	./.	1/1	./.	1/1
0/0	0/0	0/0	1/1	0/0	0/0	0/0	1/1	1/1	0/0	0/0	0/0	0/0	0/0	1/1	0/0	1/1	0/1	0/0	1/1	1/1	1/1	0/0	1/1	0/0	1/1	0/0
0/0	0/0	0/0	1/1	0/0	0/0	0/0	1/1	1/1	0/0	0/0	0/0	0/0	0/0	1/1	0/0	1/1	1/1	0/0	1/1	1/1	1/1	0/0	1/1	0/0	1/1	0/0
0/0	0/0	./.	0/0	./.	./.	./.	0/0	0/0	./.	0/0	./.	./.	0/0	0/0	./.	0/0	0/0	./.	0/0	0/0	./.	./.	0/0	./.	0/0	0/0
./.	./.	./.	0/0	0/0	0/0	0/1	0/0	0/0	./.	./.	0/0	./.	0/0	0/0	0/0	0/0	0/0	0/0	0/0	0/0	./.	0/0	0/0	./.	0/0	0/0
0/1	1/1	1/1	0/0	1/1	1/1	0/1	0/0	0/0	0/1	1/1	1/1	0/1	1/1	0/0	1/1	0/0	0/0	1/1	0/0	0/0	./.	0/1	0/0	1/1	0/0	1/1

0/1	2/2	2/2	0/0	0/0	2/2	0/2	0/0	0/0	0/1	2/2	0/0	0/1	0/0	0/0	0/0	0/0	0/0	0/0	0/0	0/0	./.	0/0	0/0	2/2	0/0	2/2
0/0	4/4	4/4	0/0	0/0	4/4	0/4	0/0	0/0	0/4	4/4	0/0	0/4	4/4	0/0	0/0	0/0	0/0	0/0	0/0	0/0	2/2	0/0	0/0	4/4	0/0	4/4
0/0	0/0	0/0	0/0	0/0	0/0	0/0	0/0	0/0	0/0	0/0	0/0	0/0	0/0	0/0	0/0	0/0	0/0	0/0	0/0	0/0	0/0	0/0	0/0	0/0	0/0	0/0
1/1	1/1	1/1	1/1	1/1	1/1	1/1	1/1	1/1	1/1	1/1	1/1	1/1	1/1	1/1	1/1	1/1	1/1	1/1	1/1	1/1	1/1	1/1	1/1	1/1	1/1	1/1
1/1	1/1	1/1	0/0	1/1	1/1	1/1	0/0	0/0	1/1	1/1	1/1	1/1	1/1	0/0	1/1	0/0	0/1	1/1	0/0	0/0	0/0	1/1	0/0	1/1	0/0	1/1
0/0	0/0	0/0	0/0	0/0	0/0	0/0	0/0	0/0	0/0	0/0	0/0	0/0	0/0	0/0	0/0	0/0	0/0	0/0	0/0	0/0	0/0	0/0	0/0	0/0	0/0	0/0
0/0	0/0	0/0	0/0	0/0	0/0	0/0	0/0	0/0	0/0	0/0	0/0	0/0	0/0	0/0	0/0	0/0	0/0	0/0	0/0	0/0	0/0	0/0	0/0	0/0	0/0	0/0
1/1	1/1	1/1	1/1	1/1	1/1	1/1	1/1	1/1	1/1	1/1	1/1	1/1	1/1	1/1	1/1	1/1	1/1	1/1	1/1	1/1	1/1	1/1	1/1	1/1	1/1	1/1
./.	0/0	0/0	0/0	0/0	./.	0/0	0/0	0/0	0/0	./.	0/0	./.	0/0	0/0	0/0	0/0	0/0	0/0	0/0	0/0	0/0	0/0	0/0	0/0	0/0	0/0
1/1	1/1	1/1	0/0	1/1	1/1	1/1	0/0	0/0	1/1	1/1	1/1	1/1	1/1	0/0	1/1	0/0	0/1	1/1	0/0	0/0	0/0	1/1	0/0	1/1	0/0	1/1
1/1	1/1	1/1	0/0	1/1	1/1	1/1	0/0	0/0	1/1	1/1	1/1	1/1	1/1	0/0	1/1	0/0	0/1	1/1	0/0	0/0	0/0	1/1	0/0	1/1	0/0	1/1
1/1	1/1	1/1	0/0	1/1	1/1	1/1	0/0	0/0	1/1	1/1	1/1	1/1	1/1	0/0	1/1	0/0	0/1	1/1	0/0	0/0	0/0	1/1	0/0	1/1	0/0	1/1
0/0	0/0	0/0	1/1	0/0	0/0	0/0	1/1	1/1	0/0	0/0	0/0	0/0	0/0	1/1	0/0	1/1	0/1	0/0	1/1	1/1	1/1	0/0	1/1	0/0	1/1	0/0
1/1	1/1	1/1	0/0	1/1	1/1	1/1	0/0	0/0	1/1	1/1	1/1	1/1	1/1	0/0	1/1	0/0	0/0	1/1	0/0	0/0	0/0	1/1	0/0	1/1	0/0	1/1
1/1	1/1	1/1	1/1	1/1	1/1	1/1	1/1	1/1	1/1	1/1	1/1	1/1	1/1	1/1	1/1	1/1	1/1	1/1	1/1	1/1	1/1	1/1	1/1	1/1	1/1	1/1
0/0	0/0	0/0	0/0	0/0	0/0	0/0	0/0	0/0	0/0	0/0	0/0	0/0	0/0	0/0	0/0	0/0	0/0	0/0	0/0	0/0	0/0	0/0	0/0	0/0	0/0	0/0
0/0	0/0	0/0	0/0	0/0	0/0	0/0	0/0	0/0	0/0	0/0	0/0	0/0	0/0	0/0	0/0	0/0	0/0	0/0	0/0	0/0	0/0	0/0	0/0	0/0	0/0	0/0
0/0	0/0	0/0	1/1	0/0	0/0	0/0	1/1	1/1	0/0	0/0	0/0	0/0	0/0	1/1	0/0	1/1	0/1	0/0	1/1	1/1	1/1	0/0	1/1	0/0	1/1	0/0
0/0	0/0	0/0	0/0	0/0	0/0	0/0	0/0	0/0	0/0	0/0	0/0	0/0	0/0	0/0	0/0	0/0	0/0	0/0	0/0	0/0	0/0	0/0	0/0	0/0	0/0	0/0
0/0	0/0	0/0	1/1	0/0	0/0	0/0	1/1	1/1	0/0	0/0	0/0	0/0	0/0	1/1	0/0	1/1	0/1	0/0	1/1	1/1	1/1	0/0	1/1	0/0	1/1	0/0
0/0	0/0	0/0	0/0	0/0	1/1	1/1	0/0	0/0	1/1	1/1	1/1	0/0	0/0	0/0	1/1	0/0	0/0	1/1	0/0	0/0	0/0	1/1	0/0	1/1	0/0	0/0
0/0	0/0	0/0	1/1	0/0	0/0	0/0	1/1	1/1	0/0	0/0	0/0	0/0	0/0	1/1	0/0	1/1	1/1	0/0	1/1	1/1	1/1	0/0	1/1	0/0	1/1	0/0
0/0	0/0	0/0	1/1	0/0	0/0	0/0	1/1	1/1	0/0	0/0	0/0	0/0	0/0	1/1	0/0	1/1	1/1	0/0	1/1	1/1	1/1	0/0	1/1	0/0	1/1	0/0
0/0	0/0	0/0	0/0	0/0	0/0	0/0	0/0	0/0	0/0	0/0	0/0	0/0	0/0	0/0	0/0	0/0	0/0	0/0	0/0	0/0	0/0	0/0	0/0	0/0	0/0	0/0
0/0	0/0	0/0	0/0	0/0	0/0	0/0	0/0	0/0	0/0	0/0	0/0	0/0	0/0	0/0	0/0	0/0	0/0	0/0	0/0	0/0	0/0	0/0	0/0	0/0	0/0	0/0
1/1	1/1	1/1	0/0	1/1	1/1	1/1	0/0	0/0	1/1	1/1	1/1	1/1	1/1	0/0	1/1	0/0	0/0	1/1	0/0	0/0	0/0	1/1	0/0	1/1	0/0	1/1
0/0	0/0	0/0	0/0	0/0	0/0	0/0	0/0	0/0	0/0	0/0	0/0	0/0	0/0	0/0	0/0	0/0	0/0	0/0	0/0	0/0	0/0	0/0	0/0	0/0	0/0	0/0
1/1	1/1	1/1	1/1	1/1	1/1	1/1	1/1	1/1	1/1	1/1	1/1	1/1	1/1	1/1	1/1	1/1	1/1	1/1	1/1	1/1	1/1	1/1	1/1	1/1	1/1	1/1
0/0	0/0	0/0	0/0	0/0	0/0	0/0	0/0	0/0	0/0	0/0	0/0	0/0	0/0	0/0	0/0	0/0	0/0	0/0	0/0	0/0	0/0	0/0	0/0	0/0	0/0	0/0
1/1	1/1	1/1	1/1	1/1	1/1	1/1	1/1	1/1	1/1	1/1	1/1	1/1	1/1	1/1	1/1	1/1	1/1	1/1	1/1	1/1	1/1	1/1	1/1	1/1	1/1	1/1

0/0	0/0	0/0	0/0	0/0	0/0	0/0	0/0	0/0	0/0	0/0	0/0	0/0	0/0	0/0	0/0	0/0	0/0	0/0	0/0	0/0	0/0	0/0	0/0	0/0	0/0	0/0
0/0	0/0	0/0	0/0	0/0	0/0	0/0	0/0	0/0	0/0	0/0	0/0	0/0	0/0	0/0	0/0	0/0	0/0	0/0	0/0	0/0	0/0	0/0	0/0	0/0	0/0	0/0
0/0	0/0	0/0	0/0	0/0	0/0	0/0	0/0	0/0	0/0	0/0	0/0	0/0	0/0	0/0	0/0	0/0	0/0	0/0	0/0	0/0	0/0	0/0	0/0	0/0	0/0	0/0
1/1	1/1	1/1	0/0	1/1	0/0	0/0	0/0	0/0	0/0	0/0	0/0	1/1	1/1	0/0	0/0	0/0	0/0	0/0	0/0	0/0	0/0	0/0	0/0	0/0	0/0	1/1
1/1	1/1	1/1	0/0	1/1	1/1	1/1	0/0	0/0	1/1	1/1	1/1	1/1	1/1	0/0	1/1	0/0	0/0	1/1	0/0	0/0	0/0	1/1	0/0	1/1	0/0	1/1
1/1	1/1	1/1	1/1	1/1	1/1	1/1	1/1	1/1	1/1	1/1	1/1	1/1	1/1	1/1	1/1	1/1	1/1	1/1	1/1	1/1	1/1	1/1	1/1	1/1	1/1	1/1
1/1	1/1	1/1	0/0	1/1	1/1	1/1	0/0	0/0	1/1	1/1	1/1	1/1	1/1	0/0	1/1	0/0	0/0	1/1	0/0	0/0	0/0	1/1	0/0	1/1	0/0	1/1
0/0	0/0	0/0	0/0	0/0	1/1	1/1	0/0	0/0	1/1	1/1	1/1	0/0	0/0	0/0	1/1	0/0	0/0	1/1	0/0	0/0	0/0	1/1	0/0	1/1	0/0	0/0
0/0	0/0	0/0	0/0	0/0	0/0	0/0	0/0	0/0	0/0	0/0	0/0	0/0	0/0	0/0	0/0	0/0	0/0	0/0	0/0	0/0	0/0	0/0	0/0	0/0	0/0	0/0
0/0	0/0	0/0	0/0	0/0	0/0	0/0	0/0	0/0	0/0	0/0	0/0	0/0	0/0	0/0	0/0	1/1	0/0	0/0	0/0	0/0	0/0	0/0	0/0	0/0	0/0	0/0
0/0	0/0	0/0	0/0	0/0	0/0	0/0	0/0	0/0	0/0	0/0	0/0	0/0	0/0	0/0	0/0	0/0	0/0	0/0	0/0	0/0	0/0	0/0	0/0	0/0	0/0	0/0
0/0	0/0	0/0	1/1	0/0	0/0	0/0	1/1	1/1	0/0	0/0	0/0	0/0	0/0	1/1	0/0	1/1	0/1	0/0	1/1	1/1	1/1	0/0	1/1	0/0	1/1	0/0
1/1	0/1	1/1	1/1	1/1	0/0	0/0	1/1	1/1	0/0	0/0	0/0	1/1	1/1	1/1	0/0	1/1	0/1	0/0	1/1	1/1	1/1	0/0	1/1	0/0	1/1	1/1
1/1	1/1	1/1	1/1	1/1	1/1	1/1	1/1	1/1	1/1	1/1	1/1	1/1	1/1	1/1	1/1	1/1	1/1	1/1	1/1	1/1	1/1	1/1	1/1	1/1	1/1	1/1
1/1	1/1	1/1	1/1	1/1	1/1	1/1	1/1	1/1	1/1	1/1	1/1	1/1	1/1	1/1	1/1	1/1	1/1	1/1	1/1	1/1	1/1	1/1	1/1	1/1	1/1	1/1
0/0	0/0	0/0	0/0	0/0	0/0	0/0	0/0	0/0	0/0	0/0	0/0	0/0	0/0	0/0	0/0	0/0	0/0	0/0	0/0	0/0	0/0	0/0	0/0	0/0	0/0	0/0
1/1	1/1	1/1	0/0	1/1	0/0	0/0	0/0	0/0	0/0	0/0	0/0	1/1	1/1	0/0	0/0	0/0	0/0	0/0	0/0	0/0	0/0	0/0	0/0	0/0	0/0	1/1
1/1	1/1	1/1	0/0	1/1	1/1	1/1	0/0	0/0	1/1	1/1	1/1	1/1	1/1	0/0	1/1	0/0	0/0	1/1	0/0	0/0	0/0	1/1	0/0	1/1	0/0	1/1
1/1	1/1	1/1	1/1	1/1	1/1	1/1	1/1	1/1	1/1	1/1	1/1	1/1	1/1	1/1	1/1	1/1	1/1	1/1	1/1	1/1	1/1	1/1	1/1	1/1	1/1	1/1
0/0	0/0	0/0	0/0	0/0	0/0	0/0	0/0	0/0	0/0	0/0	0/0	0/0	0/0	0/0	0/0	0/0	0/0	0/0	0/0	0/0	1/1	0/0	0/0	0/0	0/0	0/0
0/0	0/0	0/0	0/0	0/0	0/0	0/0	0/0	0/0	0/0	0/0	0/0	0/0	0/0	0/0	0/0	0/0	0/0	0/0	0/0	0/0	0/0	0/0	0/0	0/0	0/0	0/0
0/0	0/0	0/0	0/0	0/0	0/0	0/0	0/0	0/0	0/0	0/0	0/0	0/0	0/0	0/0	0/0	0/0	0/0	0/0	0/0	0/0	0/0	0/0	0/0	0/0	0/0	0/0
1/1	1/1	1/1	1/1	1/1	1/1	1/1	1/1	1/1	1/1	1/1	1/1	1/1	1/1	1/1	1/1	1/1	1/1	1/1	1/1	1/1	1/1	1/1	1/1	1/1	1/1	1/1
0/0	0/0	0/0	0/0	0/0	0/0	0/0	0/0	0/0	0/0	0/0	0/0	0/0	0/0	0/0	0/0	0/0	0/0	0/0	0/0	0/0	0/0	0/0	0/0	0/0	0/0	0/0
0/0	0/0	0/0	0/0	0/0	0/0	0/0	0/0	0/0	0/0	0/0	0/0	0/0	0/0	0/0	0/0	0/0	0/0	0/0	0/0	0/0	0/0	0/0	0/0	0/0	0/0	0/0
0/0	0/0	0/0	0/0	0/0	0/0	0/0	0/0	0/0	0/0	0/0	0/0	0/0	0/0	0/0	0/0	0/0	0/0	0/0	0/0	0/0	0/0	0/0	0/0	0/0	0/0	0/0
0/0	0/0	0/0	0/0	0/0	0/0	0/0	0/0	0/0	0/0	0/0	0/0	0/0	0/0	0/0	0/0	0/0	0/0	0/0	0/0	0/0	0/0	0/0	0/0	0/0	0/0	0/0
0/0	0/0	0/0	0/0	0/0	0/0	0/0	0/0	0/0	0/0	0/0	0/0	0/0	0/0	0/0	0/0	0/0	0/0	0/0	0/0	0/0	0/0	0/0	0/0	0/0	0/0	0/0
0/0	0/0	0/0	0/0	0/0	0/0	0/0	0/0	0/0	0/0	0/0	0/0	0/0	0/0	0/0	0/0	0/0	0/0	0/0	0/0	0/0	0/0	0/0	0/0	0/0	0/0	0/0
0/0	0/0	0/0	0/0	0/0	0/0	0/0	0/0	0/0	0/0	0/0	0/0	0/0	0/0	0/0	0/0	0/0	0/0	0/0	0/0	0/0	0/0	0/0	0/0	0/0	0/0	0/0
0/0	0/0	0/0	0/0	0/0	0/0	0/0	0/0	0/0	0/0	0/0	0/0	0/0	0/0	0/0	0/0	0/0	0/0	0/0	0/0	0/0	0/0	0/0	0/0	0/0	0/0	0/0
0/0	0/0	0/0	1/1	0/0	0/0	0/0	1/1	1/1	0/0	0/0	0/0	0/0	0/0	1/1	0/0	1/1	0/1	0/0	1/1	1/1	1/1	0/0	1/1	0/0	1/1	0/0
0/0	0/0	0/0	0/0	0/0	0/0	0/0	0/0	0/0	0/0	0/0	0/0	0/0	0/0	0/0	0/0	0/0	0/0	0/0	0/0	0/0	0/0	0/0	0/0	0/0	0/0	0/0
0/0	0/0	0/0	0/0	0/0	0/0	0/0	0/0	0/0	0/0	0/0	0/0	0/0	0/0	0/0	0/0	0/0	0/0	0/0	0/0	0/0	0/0	0/0	0/0	0/0	0/0	0/0
1/1	1/1	1/1	0/0	1/1	0/0	0/0	0/0	0/0	0/0	0/0	0/0	1/1	1/1	0/0	0/0	0/0	0/1	0/0	0/0	0/0	0/0	0/0	0/0	0/0	0/0	1/1

0/0	0/0	0/0	0/0	0/0	1/1	1/1	0/0	0/0	1/1	1/1	1/1	0/0	0/0	0/0	1/1	0/0	0/0	1/1	0/0	0/0	0/0	1/1	0/0	1/1	0/0	0/0
2/3	2/2	2/2	1/2	2/2	4/4	4/4	1/1	1/2	4/4	4/4	4/4	2/2	2/2	1/1	4/4	2/2	1/2	4/4	1/1	1/2	1/1	4/4	1/1	1/4	1/1	2/2
./.	./.	./.	./.	./.	./.	./.	./.	./.	./.	./.	./.	./.	./.	./.	./.	./.	./.	./.	./.	./.	./.	./.	./.	./.	./.	./.
0/0	0/0	0/0	0/0	0/0	0/0	0/0	0/0	0/0	0/0	0/0	0/0	0/0	0/0	0/0	0/0	0/0	0/0	0/0	0/0	0/0	0/0	0/0	0/0	0/0	0/0	0/0
0/0	0/0	0/0	0/0	0/0	0/0	0/0	0/0	0/0	0/0	0/0	0/0	0/0	0/0	0/0	0/0	0/0	0/0	0/0	0/0	0/0	0/0	0/0	0/0	0/0	0/0	0/0
0/0	0/0	0/0	0/0	0/0	0/0	0/0	0/0	0/0	0/0	0/0	0/0	0/0	0/0	0/0	0/0	0/0	0/0	0/0	0/0	0/0	0/0	0/0	0/0	0/0	0/0	0/0
0/0	0/0	0/0	0/0	0/0	1/1	1/1	0/0	0/0	1/1	1/1	1/1	0/0	0/0	0/0	1/1	0/0	0/0	1/1	0/0	0/0	0/0	1/1	0/0	1/1	0/0	0/0
0/0	0/0	0/0	0/0	./.	1/1	1/1	0/0	./.	1/1	1/1	1/1	0/0	0/0	0/0	1/1	0/0	0/0	1/1	0/0	0/0	0/0	1/1	0/0	1/1	./.	0/0
0/0	0/0	0/0	0/0	./.	0/0	0/0	0/0	./.	0/0	0/0	0/0	./.	0/0	0/0	0/0	0/0	0/0	0/0	./.	0/0	0/0	0/0	0/0	./.	./.	0/0
1/1	1/1	1/1	0/0	1/1	1/1	1/1	./.	0/1	1/1	1/1	0/1	1/1	1/1	0/0	1/1	./.	0/1	1/1	./.	./.	1/1	1/1	./.	1/1	./.	1/1
./.	./.	./.	2/2	./.	./.	./.	2/2	0/2	./.	./.	./.	./.	./.	2/2	./.	2/2	0/2	./.	2/2	2/2	./.	./.	2/2	./.	2/2	./.
0/0	0/0	./.	./.	0/0	0/0	./.	./.	./.	0/0	0/0	0/1	0/0	0/0	./.	./.	./.	./.	0/0	./.	./.	./.	./.	./.	0/0	./.	0/0
0/0	0/0	0/0	0/0	0/0	0/0	0/0	0/0	0/0	0/0	0/0	0/0	0/0	0/0	0/0	0/0	0/0	0/0	0/0	0/0	0/0	0/0	0/0	0/0	0/0	0/0	0/0
0/0	0/0	0/0	0/0	0/0	0/0	0/0	0/0	0/0	0/0	0/0	0/0	0/0	0/0	0/0	0/0	0/0	0/0	0/0	0/0	0/0	0/0	0/0	0/0	0/0	0/0	0/0
0/0	0/0	0/0	0/0	0/0	0/0	0/0	0/0	0/0	0/0	0/0	0/0	0/0	0/0	0/0	0/0	0/0	0/0	0/0	0/0	0/0	0/0	0/0	0/0	0/0	0/0	0/0
1/1	1/1	1/1	1/1	1/1	1/1	1/1	1/1	1/1	1/1	1/1	1/1	1/1	1/1	1/1	1/1	1/1	1/1	1/1	1/1	1/1	1/1	1/1	1/1	1/1	1/1	1/1
0/0	0/0	0/0	0/0	0/0	0/0	0/0	0/0	0/0	0/0	0/0	0/0	0/0	0/0	0/0	0/0	0/0	0/0	0/0	0/0	0/0	0/0	0/0	0/0	0/0	0/0	0/0
1/1	1/1	1/1	0/0	1/1	0/0	0/0	0/0	0/0	0/0	0/0	0/0	1/1	1/1	0/0	0/0	0/0	0/1	0/0	0/0	0/0	0/0	0/0	0/0	0/0	0/0	1/1
0/0	0/0	0/0	0/0	0/0	0/0	0/0	0/0	0/0	0/0	0/0	0/0	0/0	0/0	0/0	0/0	0/0	0/0	0/0	0/0	0/0	0/0	0/0	0/0	0/0	0/0	0/0
0/0	0/0	0/0	0/0	0/0	0/0	0/0	0/0	0/0	0/0	0/0	0/0	0/0	0/0	0/0	0/0	0/0	0/0	0/0	0/0	0/0	0/0	0/0	0/0	0/0	0/0	0/0
0/0	0/0	0/0	0/0	0/0	0/0	0/0	0/0	0/0	0/0	0/0	0/0	0/0	0/0	0/0	0/0	0/0	0/0	0/0	0/0	0/0	0/0	0/0	0/0	0/0	0/0	0/0

0/0	0/0	0/0	0/0	0/0	0/0	0/0	0/0	0/0	0/0	0/0	0/0	0/0	0/0	0/0	0/0	0/0	0/0	0/0	0/0	0/0	0/0	0/0	0/0	0/0	0/0	0/0
0/0	0/0	0/0	0/0	0/0	0/0	0/0	0/0	0/0	0/0	0/0	0/0	0/0	0/0	0/0	0/0	0/0	0/0	0/0	0/0	0/0	0/0	0/0	0/0	0/0	0/0	0/0
1/1	1/1	1/1	2/2	1/1	1/1	1/1	2/2	2/2	1/1	1/1	1/1	1/1	1/1	2/2	1/1	2/2	1/2	1/1	2/2	2/2	2/2	1/1	2/2	1/1	2/2	1/1
0/0	0/0	0/0	0/0	0/0	0/0	0/0	0/0	0/0	0/0	0/0	0/0	0/0	0/0	0/0	0/0	0/0	0/0	0/0	0/0	0/0	0/0	0/0	0/0	0/0	0/0	0/0
1/1	1/1	1/1	2/2	1/1	0/0	0/0	2/2	2/2	0/0	0/0	0/0	0/1	1/1	2/2	0/0	2/2	2/2	0/0	2/2	2/2	2/2	0/0	2/2	0/0	2/2	1/1
0/0	0/0	0/0	0/0	0/0	0/0	0/0	0/0	0/0	0/0	0/0	0/0	1/1	0/0	0/0	0/0	0/0	0/0	0/0	0/0	0/0	0/0	0/0	0/0	0/0	0/0	0/0
0/0	0/0	0/0	0/0	0/0	1/1	1/1	0/0	0/0	1/1	1/1	1/1	0/0	0/0	0/0	1/1	0/0	0/0	1/1	0/0	0/0	0/0	0/0	0/0	1/1	0/0	0/0
1/1	1/1	1/1	1/1	1/1	1/1	1/1	1/1	1/1	1/1	1/1	1/1	1/1	1/1	1/1	1/1	1/1	1/1	1/1	1/1	1/1	1/1	1/1	1/1	1/1	1/1	1/1
1/1	1/1	1/1	1/1	1/1	1/1	1/1	1/1	1/1	1/1	1/1	1/1	1/1	1/1	1/1	1/1	1/1	1/1	1/1	1/1	1/1	1/1	1/1	1/1	1/1	1/1	1/1
0/0	0/0	0/0	0/0	0/0	./.	0/0	0/0	0/0	0/0	0/0	0/0	0/0	0/0	0/0	0/0	0/0	0/0	0/0	0/0	0/0	0/0	0/0	0/0	0/0	0/0	0/0
1/1	1/1	1/1	2/2	1/1	2/2	2/2	2/2	2/2	2/2	2/2	2/2	1/1	1/1	2/2	2/2	2/2	2/2	2/2	2/2	2/2	2/2	2/2	2/2	2/2	2/2	1/1
0/0	0/0	0/0	0/0	0/0	0/0	0/0	0/0	0/0	0/0	0/0	0/0	0/0	0/0	0/0	./.	0/0	0/0	0/0	0/0	0/0	0/0	0/0	0/0	0/0	0/0	0/0
0/0	0/0	0/0	0/0	0/0	0/0	0/0	0/0	0/0	1/1	0/0	0/0	0/0	0/0	0/0	0/0	0/0	0/0	0/0	0/0	0/0	0/0	0/0	0/0	0/0	0/0	0/0
0/0	0/0	0/0	0/0	0/0	0/0	0/0	0/0	0/0	0/0	0/0	0/0	0/0	0/0	0/0	0/0	0/0	0/0	0/0	0/0	0/0	0/0	0/0	0/0	0/0	0/0	0/0
0/0	0/0	0/0	0/0	0/0	1/1	1/1	0/0	0/0	1/1	1/1	1/1	0/0	0/0	0/0	1/1	0/0	0/0	1/1	0/0	0/0	0/0	1/1	0/0	1/1	0/0	0/0
0/0	0/0	0/0	0/0	0/0	0/0	0/0	0/0	0/0	0/0	0/0	0/0	0/0	0/0	0/0	1/1	0/0	0/0	0/0	0/0	0/0	0/0	0/0	0/0	0/0	0/0	0/0
0/0	0/0	0/0	0/0	0/0	1/1	1/1	0/0	0/0	1/1	1/1	1/1	0/0	0/0	0/0	1/1	0/0	0/0	1/1	0/0	0/0	0/0	1/1	0/0	1/1	0/0	0/0
1/1	1/1	1/1	1/1	1/1	1/1	1/1	1/1	1/1	1/1	1/1	1/1	1/1	1/1	1/1	1/1	1/1	1/1	1/1	1/1	1/1	1/1	1/1	1/1	1/1	1/1	1/1

58	592	593	595	596	598	603	606	608	609	611	612	613	616	620	622	62	632	633	643	645	64	651	654	659	667	674
0/0	0/0	0/0	0/0	0/0	0/0	0/0	0/0	0/0	0/0	0/0	0/0	0/0	0/0	0/0	0/0	0/0	0/0	0/0	0/0	0/0	0/0	0/0	0/0	0/0	0/0	0/0
0/0	0/0	0/0	0/0	0/0	0/0	0/0	0/1	0/0	0/0	1/1	0/0	0/0	1/1	0/0	0/0	0/0	0/0	0/0	0/0	0/0	1/1	0/0	0/1	0/0	1/1	0/0
0/0	0/0	0/0	0/0	0/0	0/0	0/0	0/0	0/0	0/0	0/0	0/0	0/0	0/0	0/0	0/0	0/0	0/0	0/0	0/0	0/0	0/0	0/0	0/0	0/0	0/0	0/0
0/0	0/0	0/0	1/1	0/0	0/0	0/0	0/0	0/0	0/0	0/0	0/0	0/0	0/0	0/0	1/1	0/0	1/1	0/0	0/0	0/0	0/0	0/0	0/0	0/0	0/0	0/0
0/0	1/1	0/0	0/0	0/0	1/1	0/0	0/0	1/1	0/0	0/0	0/0	0/0	0/0	1/1	1/1	1/1	1/1	1/1	1/1	1/1	0/0	0/0	1/1	1/1	0/0	1/1
0/0	1/1	0/0	1/1	0/0	1/1	0/0	0/0	1/1	0/0	0/0	0/0	0/0	0/0	1/1	1/1	1/1	1/1	1/1	1/1	1/1	0/0	0/0	0/1	1/1	0/0	1/1
0/0	0/0	0/0	0/0	0/0	0/0	0/0	0/0	0/0	0/0	0/0	0/0	0/0	0/0	0/0	0/0	0/0	0/0	0/0	0/0	0/0	0/0	0/0	0/0	0/0	0/0	0/0
0/0	0/0	1/1	0/0	1/1	0/0	1/1	1/1	0/0	1/1	1/1	1/1	1/1	1/1	0/0	0/0	0/0	0/0	0/0	0/0	0/0	1/1	1/1	0/1	0/0	1/1	0/0
0/0	0/0	0/0	0/0	0/0	0/0	0/0	0/0	0/0	0/0	0/0	0/0	0/0	0/0	0/0	0/0	0/0	0/0	0/0	0/0	0/0	0/0	0/0	0/0	0/0	0/0	0/0
0/0	0/0	0/0	0/0	0/1	0/0	0/0	0/0	0/0	0/0	0/0	0/0	0/0	0/0	0/0	0/0	0/0	0/0	0/0	0/0	0/0	0/0	0/0	0/0	0/0	0/0	0/0
0/0	0/1	0/0	0/0	0/1	0/0	0/0	0/0	0/0	0/0	0/0	0/0	0/0	0/0	0/0	0/0	0/0	0/0	0/0	0/0	0/0	0/0	0/0	0/0	0/0	0/0	0/0
0/0	0/0	0/0	0/0	0/0	0/0	0/0	0/0	0/0	0/0	0/0	0/0	0/0	0/0	0/0	0/0	0/0	0/0	0/0	0/0	0/0	0/0	1/1	0/0	0/0	0/0	0/0
0/0	1/1	0/0	0/0	0/0	1/1	0/0	0/1	1/1	0/0	0/0	0/0	0/0	0/0	1/1	1/1	1/1	1/1	1/1	1/1	1/1	0/0	0/0	0/1	1/1	0/0	1/1
0/0	0/0	./.	0/0	0/0	./.	1/1	./.	0/0	0/0	0/0	0/1	0/0	0/1	0/0	0/0	0/0	./.	0/0	0/0	0/0	0/1	0/0	0/0	0/0	0/1	0/0
./.	./.	1/1	0/0	1/1	./.	./.	1/1	0/0	1/1	1/1	0/1	1/1	0/1	./.	./.	0/0	./.	./.	./.	0/0	0/1	1/1	0/0	0/0	0/1	0/0
./.	./.	./.	0/0	0/0	./.	0/0	./.	0/0	0/0	0/0	0/0	0/0	0/0	0/0	./.	0/0	./.	./.	./.	0/0	0/0	0/0	0/0	0/0	0/0	0/0

3/6	3/3	1/1	./.	1/1	0/0	5/5	./.	2/3	0/4	3/3	1/5	0/1	3/5	3/6	./.	1/2	./.	6/6	./.	2/3	0/5	0/0	0/3	0/0	3/5	6/6
0/0	0/0	0/0	0/0	0/0	0/0	./.	0/0	0/0	0/0	0/0	0/0	0/0	0/0	0/0	0/0	0/0	./.	0/0	0/0	0/0	0/0	0/0	0/0	0/0	0/0	0/0
./.	0/0	0/0	./.	0/0	./.	./.	0/1	0/0	0/0	0/0	0/0	0/0	0/0	0/0	./.	0/0	./.	0/0	./.	0/0	0/0	0/0	0/0	0/0	0/0	0/0
0/0	1/1	0/0	0/0	0/0	1/1	0/0	0/1	1/1	0/0	0/0	0/0	0/0	0/0	1/1	0/0	1/1	0/0	1/1	1/1	1/1	0/0	0/0	0/1	1/1	0/0	1/1
0/0	0/0	1/1	0/0	1/1	0/0	1/1	0/1	0/0	1/1	1/1	1/1	1/1	1/1	0/0	0/0	0/0	0/0	0/0	0/0	0/0	0/1	1/1	0/1	0/0	1/1	0/0
0/0	0/0	0/0	1/1	0/0	0/0	0/0	0/0	0/0	0/0	0/0	0/0	0/0	0/0	0/0	1/1	0/0	1/1	0/0	0/0	0/0	0/0	0/0	0/0	0/0	0/0	0/0
0/0	0/0	1/1	0/0	1/1	0/0	1/1	0/1	0/0	1/1	1/1	1/1	1/1	1/1	0/0	0/0	0/0	0/0	0/0	0/0	0/0	1/1	1/1	0/0	0/0	1/1	0/0
0/0	1/1	0/0	0/0	0/0	1/1	0/0	0/0	1/1	0/0	0/0	0/0	0/0	0/0	1/1	0/0	1/1	0/0	1/1	1/1	1/1	0/0	0/0	1/1	1/1	0/0	1/1
1/1	0/0	0/0	1/1	0/0	0/0	0/0	0/0	0/0	0/0	0/0	0/0	0/0	0/0	0/0	0/0	0/0	0/0	0/0	0/0	0/0	0/0	0/0	0/0	0/0	0/0	0/0
2/2	1/1	1/1	2/2	1/1	1/1	1/1	0/0	1/1	1/1	0/0	1/1	1/1	0/0	1/1	1/1	1/1	1/1	1/1	1/1	1/1	0/0	1/1	0/1	1/1	0/0	1/1
./.	0/0	0/0	1/1	0/0	0/0	0/0	0/0	0/0	0/0	0/0	0/0	0/0	0/0	0/0	0/0	0/0	0/0	0/0	0/0	0/0	0/0	0/0	0/0	0/0	0/0	0/0
0/0	1/1	0/0	0/0	0/0	1/1	0/0	0/0	1/1	0/0	0/0	0/0	0/0	0/0	1/1	0/0	1/1	0/0	1/1	1/1	1/1	0/0	0/0	0/1	1/1	0/0	1/1
0/0	1/1	1/1	1/1	1/1	1/1	1/1	1/1	1/1	1/1	1/1	1/1	1/1	1/1	1/1	1/1	1/1	1/1	1/1	1/1	1/1	1/1	1/1	1/1	1/1	1/1	1/1
0/0	0/0	0/0	1/1	0/0	0/0	0/0	0/0	0/0	0/0	0/0	0/0	0/0	0/0	0/0	1/1	0/0	1/1	0/0	0/0	0/0	0/0	0/0	0/0	0/0	0/0	0/0
0/0	0/0	0/0	0/0	0/0	0/0	0/0	0/0	0/0	0/0	0/0	0/0	0/0	0/0	0/0	0/0	0/0	0/0	0/0	0/0	0/0	0/0	0/0	0/0	0/0	0/0	0/0
0/0	1/1	1/1	1/1	1/1	1/1	1/1	1/1	1/1	1/1	1/1	1/1	1/1	1/1	1/1	1/1	1/1	1/1	1/1	1/1	1/1	1/1	1/1	1/1	1/1	1/1	1/1
0/0	1/1	0/0	0/0	0/0	1/1	0/0	0/0	1/1	0/0	0/0	0/0	0/0	0/0	1/1	0/0	1/1	0/0	1/1	1/1	1/1	0/0	0/0	0/1	1/1	0/0	1/1
0/0	0/0	0/0	1/1	0/0	0/0	0/0	0/0	0/0	0/0	0/0	0/0	0/0	0/0	0/0	1/1	0/0	1/1	0/0	0/0	0/0	0/0	0/0	0/0	0/0	0/0	0/0
0/0	0/0	1/1	0/0	1/1	0/0	1/1	1/1	0/0	1/1	1/1	1/1	1/1	1/1	0/0	0/0	0/0	0/0	0/0	0/0	0/0	1/1	1/1	0/1	0/0	1/1	0/0
0/0	0/0	0/0	0/0	0/0	0/0	0/0	0/1	0/0	0/0	1/1	0/0	0/0	1/1	0/0	0/0	0/0	0/0	0/0	0/0	0/0	1/1	0/0	0/1	0/0	1/1	0/0
0/0	0/0	0/0	1/1	0/0	0/0	0/0	0/0	0/0	0/0	0/0	0/0	0/0	0/0	0/0	1/1	0/0	1/1	0/0	0/0	0/0	0/0	0/0	0/0	0/0	0/0	0/0
0/0	0/0	1/1	0/0	1/1	0/0	1/1	0/1	0/0	1/1	1/1	1/1	1/1	1/1	0/0	0/0	0/0	0/0	0/0	0/0	0/0	1/1	1/1	0/1	0/0	1/1	0/0
0/0	0/0	0/0	1/1	0/0	0/0	0/0	0/0	0/0	0/0	0/0	0/0	0/0	0/0	0/0	1/1	0/0	1/1	0/0	0/0	0/0	0/0	0/0	0/0	0/0	0/0	0/0
0/0	1/1	1/1	1/1	1/1	1/1	1/1	1/1	1/1	1/1	1/1	1/1	1/1	1/1	1/1	1/1	1/1	1/1	1/1	1/1	1/1	1/1	1/1	1/1	1/1	1/1	1/1
0/0	1/1	0/0	0/0	0/0	1/1	0/0	0/0	1/1	0/0	0/0	0/0	0/0	0/0	1/1	0/0	1/1	0/0	1/1	1/1	1/1	0/0	0/0	0/1	1/1	0/0	1/1
0/0	0/0	0/0	2/2	0/0	0/0	0/0	0/1	0/0	0/0	1/1	0/0	0/0	1/1	0/0	2/2	0/0	2/2	0/0	0/0	0/0	1/1	0/0	0/1	0/0	1/1	0/0

1/1	1/1	1/1	1/1	1/1	1/1	1/1	1/1	1/1	1/1	1/1	1/1	1/1	1/1	1/1	1/1	1/1	1/1	1/1	1/1	1/1	1/1	1/1	1/1	1/1	1/1	1/1
0/0	1/1	0/0	1/1	0/0	1/1	0/0	0/0	1/1	0/0	0/0	0/0	0/0	0/0	1/1	1/1	1/1	1/1	1/1	1/1	1/1	0/0	0/0	0/1	1/1	0/0	1/1
0/0	1/1	0/0	0/0	0/0	1/1	0/0	0/0	1/1	0/0	0/0	0/0	0/0	0/0	1/1	0/0	1/1	0/0	1/1	1/1	1/1	0/0	0/0	0/1	1/1	0/0	1/1
0/0	0/0	0/0	0/0	0/0	0/0	0/0	0/0	0/0	0/0	0/0	0/0	0/0	0/0	0/0	0/0	0/0	0/0	0/0	0/0	0/0	0/0	0/0	0/0	0/0	0/0	0/0
0/0	1/1	1/1	1/1	1/1	1/1	1/1	1/1	1/1	1/1	1/1	1/1	1/1	1/1	1/1	1/1	1/1	1/1	1/1	1/1	1/1	1/1	1/1	1/1	1/1	1/1	1/1
0/0	1/1	1/1	1/1	1/1	1/1	1/1	1/1	1/1	1/1	1/1	1/1	1/1	1/1	1/1	1/1	1/1	1/1	1/1	1/1	1/1	1/1	1/1	1/1	1/1	1/1	1/1
0/0	1/1	1/1	1/1	1/1	1/1	1/1	1/1	1/1	1/1	1/1	1/1	1/1	1/1	1/1	1/1	1/1	1/1	1/1	1/1	1/1	1/1	1/1	1/1	1/1	1/1	1/1
0/0	0/0	0/0	0/0	0/0	0/0	0/0	0/0	0/0	0/0	0/0	0/0	0/0	0/0	./.	1/1	./.	1/1	0/0	0/0	./.	0/0	0/0	0/0	0/0	0/0	0/0
0/0	1/1	0/0	1/1	0/0	1/1	0/0	0/1	1/1	0/0	0/0	0/0	0/0	0/0	1/1	0/0	1/1	0/0	1/1	1/1	1/1	0/0	0/0	1/1	1/1	0/0	1/1
0/0	0/0	0/0	0/0	0/0	0/0	0/0	0/0	0/0	0/0	0/0	0/0	0/0	0/0	0/0	0/0	0/0	0/0	0/0	0/0	0/0	0/0	0/0	0/0	0/0	0/0	0/0
0/0	0/0	1/1	0/0	1/1	0/0	1/1	0/1	0/0	1/1	1/1	1/1	1/1	1/1	0/0	0/0	0/0	0/0	0/0	0/0	0/0	1/1	1/1	0/0	0/0	1/1	0/0
0/0	0/0	0/0	0/0	0/0	0/0	0/0	0/0	0/0	0/0	0/0	0/0	0/0	0/0	0/0	0/0	0/0	0/0	0/0	0/0	0/0	0/0	0/0	0/0	0/0	0/0	0/0
0/0	0/0	0/0	0/0	0/0	0/0	0/0	0/0	1/1	0/0	0/0	0/0	0/0	0/0	0/0	0/0	0/0	0/0	0/0	0/0	0/0	0/0	0/0	0/0	0/0	0/0	0/0
0/0	0/0	1/1	0/0	1/1	0/0	1/1	0/0	0/0	1/1	0/0	1/1	1/1	0/0	0/0	0/0	0/0	0/0	0/0	0/0	0/0	0/0	1/1	0/0	0/0	0/0	0/0
0/0	1/1	1/1	1/1	1/1	1/1	1/1	1/1	1/1	1/1	1/1	1/1	1/1	1/1	1/1	1/1	1/1	1/1	1/1	1/1	1/1	1/1	1/1	1/1	1/1	1/1	1/1
0/0	0/0	0/0	0/0	0/0	0/0	0/0	0/0	0/0	0/0	0/0	0/0	0/0	0/0	0/0	1/1	0/0	1/1	0/0	0/0	0/0	0/0	0/0	0/0	0/0	0/0	0/0
0/0	0/0	0/0	0/0	0/0	0/0	0/0	0/0	0/0	0/0	0/0	0/0	0/0	0/0	0/0	0/0	0/0	0/0	0/0	0/0	0/0	0/0	0/0	0/0	0/0	0/0	0/0
0/0	0/0	0/0	0/0	0/0	0/0	0/0	0/0	1/1	0/0	0/0	0/0	0/0	0/0	0/0	0/0	0/0	0/0	0/0	0/0	0/0	0/0	0/0	0/0	0/0	0/0	0/0
0/0	0/0	1/1	0/0	1/1	0/0	1/1	1/1	0/0	1/1	1/1	1/1	1/1	1/1	0/0	0/0	0/0	0/0	0/0	0/0	0/0	1/1	1/1	0/1	0/0	1/1	0/0
0/0	0/0	0/0	0/0	0/0	0/0	0/0	0/0	0/0	0/0	0/0	0/0	0/0	0/0	0/0	1/1	0/0	1/1	0/0	0/0	0/0	0/0	0/0	0/0	0/0	0/0	0/0
0/0	1/1	1/1	1/1	1/1	1/1	1/1	1/1	1/1	1/1	1/1	1/1	1/1	1/1	1/1	1/1	1/1	1/1	1/1	1/1	1/1	1/1	1/1	1/1	1/1	1/1	1/1
0/0	0/0	1/1	0/0	1/1	0/0	1/1	1/1	0/0	1/1	1/1	1/1	1/1	1/1	0/0	0/0	0/0	0/0	0/0	0/0	0/0	1/1	1/1	0/1	0/0	1/1	0/0
0/0	0/0	1/1	0/0	1/1	0/0	1/1	1/1	0/0	1/1	1/1	1/1	1/1	1/1	0/0	0/0	0/0	0/0	0/0	0/0	0/0	0/1	1/1	0/1	0/0	1/1	0/0
0/0	1/1	0/0	1/1	0/0	1/1	0/0	0/0	1/1	0/0	0/0	0/0	0/0	0/0	1/1	1/1	1/1	1/1	1/1	1/1	1/1	0/1	0/0	0/1	1/1	0/0	1/1
0/0	1/1	1/1	1/1	1/1	1/1	1/1	1/1	1/1	1/1	1/1	1/1	1/1	1/1	1/1	1/1	1/1	1/1	1/1	1/1	1/1	1/1	1/1	1/1	1/1	1/1	1/1
0/0	1/1	0/0	0/0	0/0	1/1	0/0	0/0	1/1	0/0	0/0	0/0	0/0	0/0	1/1	1/1	1/1	1/1	1/1	1/1	1/1	0/0	0/0	0/1	1/1	0/0	1/1
0/0	0/0	0/0	0/0	0/0	0/0	0/0	0/0	0/0	0/0	0/0	0/0	0/0	0/0	0/0	0/0	0/0	0/0	0/0	0/0	0/0	0/0	0/0	0/0	0/0	0/0	0/0
0/0	0/0	0/0	1/1	0/0	0/0	0/0	0/0	0/0	0/0	0/0	0/0	0/0	0/0	0/0	1/1	0/0	1/1	0/0	0/0	0/0	0/0	0/0	0/0	0/0	0/0	0/0
1/1	1/1	1/1	1/1	1/1	1/1	1/1	1/1	1/1	1/1	1/1	1/1	1/1	1/1	1/1	1/1	1/1	1/1	1/1	1/1	1/1	1/1	1/1	1/1	1/1	1/1	1/1
0/0	0/0	0/0	0/0	0/0	0/0	0/0	0/0	0/0	0/0	0/0	0/0	0/0	0/0	0/0	0/0	0/0	0/0	0/0	0/0	0/0	0/0	0/0	0/0	0/0	0/0	0/0
0/0	1/1	1/1	1/1	1/1	1/1	1/1	1/1	1/1	1/1	1/1	1/1	1/1	1/1	1/1	1/1	1/1	1/1	1/1	1/1	1/1	1/1	1/1	1/1	1/1	1/1	1/1
0/0	1/1	0/0	0/0	0/0	1/1	0/0	0/0	1/1	0/0	0/0	0/0	0/0	0/0	1/1	0/0	1/1	0/0	1/1	1/1	1/1	0/0	0/0	1/1	1/1	0/0	1/1
0/0	1/1	1/1	1/1	1/1	1/1	1/1	1/1	1/1	1/1	1/1	1/1	1/1	1/1	1/1	1/1	1/1	1/1	1/1	1/1	1/1	1/1	1/1	1/1	1/1	1/1	1/1
0/0	0/0	0/0	0/0	0/0	0/0	0/0	0/0	0/0	0/0	0/0	0/0	0/0	0/0	0/0	0/0	0/0	0/0	0/0	0/0	0/0	0/0	0/0	0/0	0/0	0/0	0/0
0/0	1/1	0/0	1/1	0/0	1/1	0/0	0/0	1/1	0/0	0/0	0/0	0/0	0/0	1/1	1/1	1/1	1/1	1/1	1/1	1/1	0/0	0/0	1/1	1/1	0/0	1/1

0/0	0/0	1/1	0/0	1/1	0/0	1/1	1/1	0/0	1/1	1/1	1/1	1/1	1/1	0/0	0/0	0/0	0/0	0/0	0/0	0/0	1/1	1/1	0/0	0/0	1/1	0/0
0/0	0/0	0/0	0/0	0/0	0/0	0/0	0/0	0/0	0/0	0/0	0/0	0/0	0/0	0/0	0/0	0/0	0/0	0/0	0/0	0/0	0/0	0/0	0/0	0/0	0/0	0/0
0/0	0/0	0/0	0/0	0/0	0/0	0/0	1/1	0/0	0/0	1/1	0/0	0/0	1/1	0/0	0/0	0/0	0/0	0/0	0/0	0/0	1/1	0/0	0/0	0/0	1/1	0/0
0/0	1/1	1/1	1/1	1/1	1/1	1/1	1/1	1/1	1/1	1/1	1/1	1/1	1/1	1/1	1/1	1/1	1/1	1/1	1/1	1/1	1/1	1/1	1/1	1/1	1/1	1/1
0/0	0/0	0/0	0/0	0/0	0/0	./.	0/0	0/0	0/0	0/0	0/0	0/0	0/0	0/0	0/0	0/0	0/0	0/0	0/0	0/0	0/0	0/0	0/0	0/0	0/0	0/0
0/0	0/0	1/1	0/0	1/1	0/0	1/1	0/1	0/0	1/1	1/1	1/1	1/1	1/1	0/0	0/0	0/0	0/0	0/0	0/0	0/0	1/1	1/1	0/0	0/0	1/1	0/0
0/0	1/1	1/1	1/1	1/1	1/1	1/1	1/1	1/1	1/1	1/1	1/1	1/1	1/1	1/1	1/1	1/1	1/1	1/1	1/1	1/1	1/1	1/1	1/1	1/1	1/1	1/1
0/0	1/1	0/0	1/1	0/0	1/1	./.	0/1	1/1	0/0	0/0	./.	0/0	0/0	1/1	0/0	1/1	0/0	1/1	1/1	1/1	./.	./.	1/1	1/1	./.	1/1
0/0	0/0	1/1	0/0	1/1	0/0	1/1	0/1	0/0	1/1	1/1	1/1	1/1	1/1	0/0	0/0	0/0	0/0	0/0	0/0	0/0	1/1	1/1	0/0	2/2	1/1	0/0
0/0	0/0	0/0	1/1	0/0	0/0	0/0	0/0	0/0	0/0	0/0	0/0	0/0	0/0	0/0	0/0	0/0	0/0	0/0	0/0	0/0	0/0	0/0	0/0	0/0	0/0	0/0
0/0	0/0	1/1	0/0	1/1	0/0	1/1	0/1	0/0	1/1	1/1	1/1	1/1	1/1	0/0	0/0	0/0	0/0	0/0	0/0	0/0	1/1	1/1	0/0	0/0	1/1	0/0
0/0	0/0	0/0	0/0	0/0	0/0	0/0	0/0	0/0	0/0	0/0	0/0	0/0	0/0	0/0	1/1	0/0	1/1	0/0	0/0	0/0	0/0	0/0	0/0	0/0	0/0	0/0
0/0	0/0	0/0	0/0	0/0	0/0	0/0	0/0	0/0	0/0	0/0	0/0	0/0	0/0	0/0	1/1	0/0	1/1	0/0	0/0	0/0	0/0	0/0	0/0	0/0	0/0	0/0
0/0	0/0	0/0	0/0	0/0	0/0	0/0	0/0	0/0	0/0	0/0	0/0	0/0	0/0	0/0	0/0	0/0	0/0	0/0	0/0	0/0	0/0	0/0	0/0	0/0	0/0	0/0
1/1	1/1	1/1	1/1	1/1	1/1	1/1	1/1	1/1	1/1	1/1	1/1	1/1	1/1	1/1	1/1	1/1	1/1	1/1	1/1	1/1	1/1	1/1	1/1	1/1	1/1	1/1
1/1	1/1	1/1	1/1	1/1	1/1	1/1	1/1	1/1	1/1	1/1	1/1	1/1	1/1	1/1	1/1	1/1	1/1	1/1	1/1	1/1	1/1	1/1	1/1	1/1	1/1	1/1
0/0	1/1	1/1	1/1	1/1	1/1	1/1	1/1	1/1	1/1	1/1	1/1	1/1	1/1	1/1	1/1	1/1	1/1	1/1	1/1	1/1	1/1	1/1	1/1	1/1	1/1	1/1
./.	0/0	./.	./.	./.	./.	./.	./.	./.	./.	./.	./.	./.	./.	0/0	0/0	0/0	./.	0/0	./.	./.	1/1	./.	./.	./.	./.	0/0
1/1	3/3	1/1	1/1	1/1	3/3	1/1	1/1	3/3	1/1	1/1	1/1	1/1	1/1	3/3	3/3	3/3	3/3	3/3	3/3	3/3	4/4	1/1	1/3	3/3	1/1	3/3
0/0	0/0	0/0	0/0	0/0	0/0	0/0	0/0	0/0	0/0	0/0	0/0	0/0	0/0	0/0	0/0	1/1	0/0	0/0	0/1	0/0	0/0	0/0	0/0	0/0	0/0	0/0
0/0	0/0	0/0	0/0	0/0	0/0	0/0	0/0	0/0	0/0	0/0	0/0	0/0	0/0	0/0	0/0	0/0	0/0	0/0	0/0	0/0	0/0	0/0	0/0	0/0	0/0	0/0
0/0	0/0	0/0	0/0	0/0	0/0	0/0	0/0	0/0	0/0	0/0	1/1	0/0	0/0	0/0	0/0	0/0	0/0	0/0	0/0	0/0	0/0	0/0	0/0	0/0	0/0	0/0
0/0	0/0	0/0	0/0	0/0	0/0	0/0	0/0	0/0	0/0	0/0	0/0	0/0	0/0	0/0	0/0	0/0	0/0	0/0	0/0	0/0	0/0	0/0	0/0	0/0	0/0	0/0
0/0	0/0	0/0	0/0	0/0	./.	0/0	0/0	0/0	0/0	0/0	0/0	0/0	0/0	0/0	1/1	./.	1/1	0/0	0/0	0/0	0/0	0/0	0/0	0/0	0/0	0/0
0/0	1/1	0/0	0/0	0/0	1/1	0/0	0/0	1/1	0/0	0/0	0/0	0/0	0/0	1/1	0/0	1/1	0/0	1/1	1/1	1/1	0/0	0/0	0/1	1/1	0/0	1/1

0/0	0/0	0/0	0/0	0/0	0/0	0/0	0/0	0/0	0/0	0/0	0/0	0/0	0/0	0/0	0/0	0/0	0/0	0/0	0/0	0/0	0/0	0/0	0/0	0/0	0/0	0/0
0/0	1/1	0/0	1/1	0/0	1/1	0/0	0/0	1/1	0/0	0/0	0/0	0/0	0/0	1/1	0/0	1/1	0/0	1/1	1/1	1/1	0/0	0/0	0/1	1/1	0/0	1/1
0/0	1/1	0/0	1/1	0/0	1/1	0/0	0/1	1/1	0/0	0/0	0/0	0/0	0/0	1/1	0/0	1/1	0/0	1/1	1/1	1/1	0/0	0/0	0/1	1/1	0/0	1/1
1/1	1/1	1/1	1/1	1/1	1/1	1/1	1/1	1/1	1/1	1/1	1/1	1/1	1/1	1/1	1/1	1/1	1/1	1/1	1/1	1/1	1/1	1/1	1/1	1/1	1/1	1/1
0/0	1/1	1/1	1/1	1/1	1/1	1/1	1/1	1/1	1/1	1/1	1/1	1/1	1/1	1/1	1/1	1/1	1/1	1/1	1/1	1/1	1/1	1/1	1/1	1/1	1/1	1/1
0/0	0/0	0/0	0/0	0/0	0/0	0/0	0/0	0/0	0/0	0/0	0/0	0/0	0/0	0/0	0/0	0/0	0/0	0/0	0/0	0/0	0/0	0/0	0/0	0/0	0/0	0/0
0/0	1/1	1/1	1/1	1/1	1/1	1/1	1/1	1/1	1/1	1/1	1/1	1/1	1/1	1/1	1/1	1/1	1/1	1/1	1/1	1/1	1/1	1/1	1/1	1/1	1/1	1/1
0/0	1/1	0/0	1/1	0/0	1/1	0/0	0/1	1/1	0/0	0/0	0/0	0/0	0/0	1/1	0/0	1/1	0/0	1/1	1/1	1/1	0/0	0/0	1/1	1/1	0/0	1/1
0/0	0/0	0/0	0/0	0/0	0/0	0/0	0/0	0/0	0/0	0/0	0/0	0/0	0/0	0/0	1/1	0/0	1/1	0/0	0/0	0/0	0/0	0/0	0/0	0/0	0/0	0/0
0/0	0/0	0/0	1/1	0/0	0/0	0/0	0/0	0/0	0/0	0/0	0/0	0/0	0/0	0/0	0/0	0/0	0/0	0/0	0/0	0/0	0/0	0/0	0/0	0/0	0/0	0/0
0/0	1/1	0/0	1/1	0/0	1/1	0/0	0/0	1/1	0/0	0/0	0/0	0/0	0/0	1/1	0/0	1/1	0/0	1/1	1/1	1/1	0/0	0/0	0/1	1/1	0/0	1/1
0/0	0/0	0/0	0/0	0/0	0/0	0/0	0/0	0/0	0/0	0/0	0/0	0/0	0/0	0/0	0/0	0/0	0/0	0/0	0/0	0/0	0/0	0/0	0/0	0/0	0/0	0/0
0/0	0/0	1/1	0/0	1/1	0/0	1/1	0/1	0/0	1/1	1/1	1/1	1/1	1/1	0/0	0/0	0/0	0/0	0/0	0/0	0/0	1/1	1/1	0/1	0/0	1/1	0/0
0/0	1/1	0/0	1/1	0/0	1/1	0/0	0/1	1/1	0/0	0/0	0/0	0/0	0/0	1/1	0/0	1/1	0/0	1/1	1/1	1/1	0/0	0/0	0/1	1/1	0/0	1/1
0/0	0/0	0/0	0/0	0/0	0/0	0/0	0/0	0/0	0/0	0/0	0/0	0/0	0/0	0/0	0/0	0/0	0/0	0/0	0/0	0/0	0/0	0/0	0/0	0/0	0/0	0/0
0/0	1/1	1/1	1/1	1/1	1/1	1/1	1/1	1/1	1/1	1/1	1/1	1/1	1/1	1/1	1/1	1/1	1/1	1/1	1/1	1/1	1/1	1/1	1/1	1/1	1/1	1/1
0/0	0/0	1/1	0/0	1/1	0/0	1/1	0/1	0/0	1/1	1/1	1/1	1/1	1/1	0/0	0/0	0/0	0/0	0/0	0/0	0/0	1/1	1/1	0/1	0/0	1/1	0/0
0/0	0/0	1/1	0/0	1/1	0/0	1/1	0/0	0/0	1/1	0/0	1/1	1/1	0/0	0/0	0/0	0/0	0/0	0/0	0/0	0/0	0/0	1/1	0/0	0/0	0/0	0/0
0/0	0/0	0/0	1/1	0/0	0/0	0/0	0/0	0/0	0/0	0/0	0/0	0/0	0/0	0/0	0/0	0/0	0/0	0/0	0/0	0/0	0/0	0/0	0/0	0/0	0/0	./.
0/0	1/1	0/0	1/1	0/0	1/1	0/0	0/1	1/1	0/0	0/0	0/0	0/0	0/0	1/1	0/0	1/1	0/0	1/1	1/1	1/1	0/0	0/0	0/1	1/1	0/0	1/1
0/0	1/1	0/0	1/1	0/0	1/1	0/0	0/1	1/1	0/0	0/0	0/0	0/0	0/0	1/1	0/0	1/1	0/0	1/1	1/1	1/1	0/0	0/0	0/1	1/1	0/0	1/1
0/0	0/0	0/0	0/0	0/0	0/0	0/0	0/0	0/0	0/0	0/0	0/0	0/0	0/0	0/0	0/0	0/0	0/0	0/0	0/0	0/0	0/0	0/0	0/0	0/0	0/0	0/0
0/0	1/1	1/1	1/1	1/1	1/1	1/1	1/1	1/1	1/1	1/1	1/1	1/1	1/1	1/1	1/1	1/1	1/1	1/1	1/1	1/1	1/1	1/1	1/1	1/1	1/1	1/1
0/0	0/0	0/0	1/1	0/0	0/0	0/0	0/0	0/0	0/0	0/0	0/0	0/0	0/0	0/0	0/0	0/0	0/0	0/0	0/0	0/0	0/0	0/0	0/0	0/0	0/0	0/0
0/0	0/0	0/0	0/0	0/0	0/0	0/0	0/0	0/0	0/0	0/0	0/0	0/0	0/0	0/0	1/1	0/0	1/1	0/0	0/0	0/0	0/0	0/0	0/0	0/0	0/0	0/0
0/0	1/1	0/0	1/1	0/0	1/1	0/0	0/0	1/1	0/0	0/0	0/0	0/0	0/0	1/1	0/0	1/1	0/0	1/1	1/1	1/1	0/0	0/0	0/1	1/1	0/0	1/1
0/0	0/0	0/0	0/0	0/0	0/0	0/0	0/0	0/0	0/0	0/0	0/0	0/0	0/0	0/0	0/0	0/0	0/0	0/0	0/0	0/0	0/0	0/0	0/0	0/0	0/0	0/0
0/0	0/0	1/1	0/0	1/1	0/0	1/1	1/1	0/0	1/1	1/1	1/1	1/1	1/1	0/0	0/0	0/0	0/0	0/0	0/0	0/0	1/1	1/1	0/1	0/0	1/1	0/0
0/0	1/1	0/0	1/1	0/0	1/1	0/0	0/0	1/1	0/0	0/0	0/0	0/0	0/0	1/1	0/0	1/1	0/0	1/1	1/1	1/1	0/0	0/0	0/1	1/1	0/0	1/1
0/0	0/0	0/0	1/1	0/0	0/0	0/0	0/0	0/0	0/0	0/0	0/0	0/0	0/0	0/0	0/0	0/0	0/0	0/0	0/0	0/0	0/0	0/0	0/0	0/0	0/0	0/0
0/0	1/1	1/1	1/1	1/1	1/1	1/1	1/1	1/1	1/1	1/1	1/1	1/1	1/1	1/1	1/1	1/1	1/1	1/1	1/1	1/1	1/1	1/1	1/1	1/1	1/1	1/1
0/0	0/0	0/0	0/0	0/0	0/0	0/0	0/0	0/0	0/0	0/0	0/0	0/0	0/0	0/0	0/0	0/0	0/0	0/0	0/0	0/0	0/0	0/0	0/0	0/0	0/0	0/0
1/1	0/0	0/0	0/0	0/0	0/0	0/0	0/0	0/0	0/0	0/0	0/0	0/0	0/0	0/0	0/0	0/0	0/0	0/0	0/0	0/0	0/0	0/0	0/0	0/0	0/0	0/0

0/0	1/1	1/1	1/1	1/1	1/1	1/1	1/1	1/1	1/1	1/1	1/1	1/1	1/1	1/1	1/1	1/1	1/1	1/1	1/1	1/1	1/1	1/1	1/1	1/1	1/1	1/1
0/0	0/0	0/0	0/0	0/0	0/0	0/0	0/0	0/0	0/0	0/0	0/0	0/0	0/0	0/0	0/0	0/0	0/0	0/0	0/0	0/0	0/0	0/0	0/0	0/0	0/0	0/0
0/0	1/1	1/1	1/1	1/1	1/1	1/1	1/1	1/1	1/1	1/1	1/1	1/1	1/1	1/1	1/1	1/1	1/1	1/1	1/1	1/1	1/1	1/1	1/1	1/1	1/1	1/1
0/0	0/0	0/0	0/0	0/0	0/0	0/0	0/0	0/0	0/0	0/0	0/0	0/0	0/0	0/0	0/0	0/0	0/0	0/0	0/0	0/0	0/0	0/0	0/0	0/0	0/0	0/0
0/0	0/0	0/0	0/0	0/0	0/0	0/0	0/1	0/0	0/0	1/1	0/0	0/0	1/1	0/0	0/0	0/0	0/0	0/0	0/0	0/0	1/1	0/0	0/1	0/0	1/1	0/0
0/0	0/0	0/0	0/0	0/0	0/0	0/0	0/0	0/0	0/0	0/0	0/0	0/0	0/0	0/0	0/0	0/0	0/0	0/0	0/0	0/0	0/0	0/0	0/0	0/0	0/0	0/0
0/0	0/0	0/0	0/0	0/0	0/0	0/0	0/0	0/0	0/0	0/0	0/0	0/0	0/0	0/0	1/1	0/0	1/1	0/0	0/0	0/0	0/0	0/0	0/0	0/0	0/0	0/0
0/0	0/0	1/1	0/0	1/1	0/0	1/1	0/0	0/0	1/1	0/0	1/1	1/1	0/0	0/0	0/0	0/0	0/0	0/0	0/0	0/0	0/0	1/1	0/0	0/0	0/0	0/0
0/0	0/0	0/0	0/0	0/0	0/0	0/0	0/0	0/0	0/0	0/0	0/0	0/0	0/0	0/0	0/0	0/0	0/0	0/0	0/0	0/0	0/0	0/0	0/0	0/0	0/0	0/0
0/0	0/0	0/0	0/0	0/0	0/0	0/0	0/0	0/0	0/0	0/0	0/0	0/0	0/0	0/0	1/1	0/0	1/1	0/0	0/0	0/0	0/0	0/0	0/0	0/0	0/0	0/0
0/0	1/1	1/1	1/1	1/1	1/1	1/1	1/1	1/1	1/1	1/1	1/1	1/1	1/1	1/1	1/1	1/1	1/1	1/1	1/1	1/1	1/1	1/1	1/1	1/1	1/1	1/1
0/0	1/1	1/1	1/1	1/1	1/1	1/1	1/1	1/1	1/1	1/1	1/1	1/1	1/1	1/1	1/1	1/1	1/1	1/1	1/1	1/1	1/1	1/1	1/1	1/1	1/1	1/1
0/0	0/0	0/0	0/0	0/0	0/0	0/0	0/0	0/0	0/0	0/0	0/0	0/0	0/0	0/0	1/1	0/0	1/1	0/0	0/0	0/0	0/0	0/0	0/0	0/0	0/0	0/0
0/0	0/0	0/0	0/0	./.	0/0	0/0	0/1	0/0	0/0	1/1	0/0	0/0	1/1	0/0	0/0	0/0	0/0	0/0	0/0	0/0	1/1	0/0	0/1	0/0	1/1	0/0
0/0	1/1	0/0	1/1	./.	1/1	0/0	0/0	0/1	0/0	0/0	0/0	0/0	0/0	1/1	0/0	1/1	0/0	1/1	1/1	1/1	0/0	0/0	0/1	1/1	0/0	1/1
2/2	1/1	1/1	2/2	1/1	1/1	1/1	1/1	1/1	1/1	1/1	1/1	1/1	0/1	0/1	0/0	1/1	0/0	0/1	1/1	1/1	1/1	1/1	0/1	1/1	1/1	1/1
0/0	1/1	1/1	1/1	1/1	1/1	1/1	1/1	1/1	1/1	1/1	1/1	1/1	1/1	1/1	1/1	1/1	1/1	1/1	1/1	1/1	1/1	1/1	1/1	1/1	1/1	1/1
0/0	1/1	0/0	0/0	0/0	1/1	0/0	0/0	1/1	0/0	0/0	0/0	0/0	0/0	1/1	0/0	1/1	0/0	1/1	1/1	1/1	0/0	0/0	0/1	0/0	0/0	1/1
0/0	1/1	0/0	0/0	0/0	1/1	0/0	0/1	1/1	0/0	0/0	0/0	0/0	0/0	1/1	0/0	1/1	0/0	1/1	1/1	1/1	0/0	0/0	0/1	0/0	0/0	1/1
0/0	0/0	0/0	0/0	0/0	0/0	0/0	1/1	0/0	0/0	1/1	0/0	0/0	1/1	0/0	0/0	0/0	0/0	0/0	0/0	0/0	1/1	0/0	0/1	0/0	1/1	0/0
0/0	0/0	0/0	0/0	0/0	0/0	0/0	0/0	0/0	0/0	0/0	0/0	0/0	0/0	0/0	0/0	0/0	0/0	0/0	0/0	0/0	0/0	0/0	0/0	0/0	0/0	0/0
0/0	0/0	0/0	0/0	0/0	0/0	0/0	0/0	0/0	0/0	0/0	0/0	0/0	0/0	0/0	0/0	0/0	0/0	0/0	0/0	0/0	0/0	0/0	0/0	0/0	0/0	0/0
0/0	0/0	0/0	0/0	0/0	0/0	0/0	0/0	0/0	0/0	0/0	0/0	0/0	0/0	0/0	0/0	0/0	0/0	0/1	0/0	0/0	0/0	0/0	0/0	0/0	0/0	0/0
0/0	0/0	0/0	0/0	0/0	0/0	0/0	0/0	0/0	0/0	0/0	0/0	0/0	0/0	0/0	0/0	0/0	0/0	0/0	0/0	0/0	0/0	0/0	0/0	0/0	0/0	0/0
0/1	0/0	0/0	0/0	0/0	0/0	0/0	0/0	0/0	0/0	0/0	0/0	0/0	0/0	0/0	0/0	0/0	0/0	0/1	0/0	0/0	0/0	0/0	0/0	0/0	0/0	0/0
0/1	0/0	0/0	0/0	0/0	0/0	0/0	0/0	0/0	0/0	0/0	0/0	0/0	0/0	0/0	0/0	0/0	0/0	0/1	0/0	0/0	0/0	0/0	0/0	0/0	0/0	0/0

0/1	0/0	0/0	0/0	0/0	0/0	0/0	0/0	0/0	0/0	0/0	0/0	0/0	0/0	0/0	0/0	0/0	0/0	0/1	0/0	0/0	0/0	0/0	0/0	0/0	0/0	0/0
0/1	0/0	0/0	0/0	0/0	0/0	0/0	0/0	0/0	0/0	0/0	0/0	0/0	0/0	0/0	0/0	0/0	0/0	0/1	0/0	0/0	0/0	0/0	0/0	0/0	0/0	0/0
0/1	0/0	0/0	0/0	0/0	0/0	0/0	0/0	0/0	0/0	0/0	0/0	0/0	0/0	0/0	0/0	0/0	0/0	0/1	0/0	0/0	0/0	0/0	0/0	0/0	0/0	0/0
0/1	0/0	0/0	0/0	0/0	0/0	0/0	0/0	0/0	0/0	0/0	0/0	0/0	0/0	0/0	0/0	0/0	0/0	0/1	0/0	0/0	0/0	0/0	0/0	0/0	0/0	0/0
0/1	0/0	0/0	0/0	0/0	0/0	0/0	0/0	0/0	0/0	0/0	0/0	0/0	0/0	0/0	0/0	0/0	0/0	0/1	0/0	0/0	0/0	0/0	0/0	0/0	0/0	0/0
0/1	0/0	0/0	0/0	0/0	0/0	0/0	0/0	0/0	0/0	0/0	0/0	0/0	0/0	0/0	0/0	0/0	0/0	0/1	0/0	0/0	0/0	0/0	0/0	0/0	0/0	0/0
0/0	0/0	0/0	0/0	0/0	0/0	0/0	0/0	0/0	0/0	0/0	0/0	0/0	0/0	0/0	0/0	0/0	0/0	0/1	0/0	0/0	0/0	0/0	0/0	0/0	0/0	0/0
0/0	0/0	0/0	0/0	0/0	0/0	0/0	0/0	0/0	0/0	0/0	0/0	0/0	0/0	0/0	0/0	0/0	0/0	0/2	0/0	0/0	0/0	0/0	0/0	0/0	0/0	0/0
0/0	0/0	0/0	0/0	0/0	0/0	0/0	0/0	0/0	0/0	0/0	0/0	0/0	0/0	0/0	0/0	0/0	0/0	0/2	0/0	0/0	0/0	0/0	0/0	0/0	0/0	0/0
0/0	0/0	0/0	0/0	0/0	0/0	0/0	0/0	0/0	0/0	0/0	0/0	0/0	0/0	0/0	0/0	0/0	0/0	0/0	0/0	0/0	0/0	0/0	0/0	0/0	0/0	0/0
1/1	1/1	1/1	1/1	1/1	1/1	1/1	1/1	1/1	1/1	1/1	1/1	1/1	1/1	1/1	1/1	1/1	1/1	1/1	1/1	1/1	1/1	1/1	1/1	1/1	1/1	1/1
0/0	0/0	0/0	0/0	0/0	0/0	0/0	0/0	0/0	0/0	0/0	0/0	0/0	0/0	0/0	0/0	0/0	0/0	0/0	0/0	0/0	0/0	0/0	0/0	0/0	0/0	0/0
0/0	0/0	0/0	1/1	0/0	0/0	0/0	0/0	0/0	0/0	0/0	0/0	0/0	0/0	0/0	0/0	0/0	0/0	0/0	0/0	0/0	0/0	0/0	0/0	0/0	0/0	0/0
0/0	0/0	1/1	0/0	1/1	0/0	1/1	0/0	0/0	1/1	0/0	1/1	1/1	0/0	0/0	1/1	0/0	1/1	0/0	0/0	0/0	0/0	1/1	0/0	1/1	0/0	0/0
0/0	0/0	0/0	0/0	0/0	0/0	0/0	0/0	0/0	0/0	0/0	0/0	0/0	0/0	0/0	0/0	0/0	0/0	0/0	0/0	0/0	0/0	0/0	0/0	0/0	0/0	0/0
0/0	0/0	0/0	0/0	0/0	0/0	0/0	0/0	0/0	0/0	0/0	0/0	0/0	0/0	0/0	0/0	0/0	0/0	0/0	0/0	0/0	0/0	0/0	0/0	0/0	0/0	0/0
0/0	1/1	1/1	1/1	1/1	1/1	1/1	1/1	1/1	1/1	1/1	1/1	1/1	1/1	1/1	1/1	1/1	1/1	1/1	1/1	1/1	1/1	1/1	1/1	1/1	1/1	1/1
0/0	./.	0/0	1/1	0/0	0/0	0/0	./.	./.	0/0	0/0	0/0	0/0	0/0	./.	0/0	./.	0/0	./.	0/0	./.	0/0	0/0	0/0	./.	0/0	./.
0/0	./.	1/1	1/1	1/1	0/0	1/1	0/1	./.	1/1	1/1	1/1	1/1	1/1	./.	1/1	./.	1/1	./.	0/0	./.	1/1	1/1	0/1	1/1	1/1	./.
0/0	1/1	0/0	0/0	0/0	1/1	0/0	0/1	1/1	0/0	0/0	0/0	0/0	0/0	1/1	0/0	1/1	0/0	1/1	1/1	1/1	0/0	0/0	0/1	0/0	0/0	1/1
0/0	1/1	0/0	0/0	0/0	1/1	0/0	0/0	1/1	0/0	0/0	0/0	0/0	0/0	1/1	0/0	1/1	0/0	1/1	1/1	1/1	0/0	0/0	0/1	0/0	0/0	1/1
0/0	0/0	0/0	0/0	./.	0/0	./.	./.	0/0	./.	./.	0/0	./.	./.	0/0	0/0	0/0	0/0	0/0	0/0	0/0	0/0	./.	0/0	0/0	./.	0/0
0/0	0/0	0/0	1/1	./.	0/0	./.	./.	0/0	./.	./.	./.	./.	./.	0/0	0/0	0/0	0/0	0/0	0/0	0/0	./.	1/1	0/0	./.	./.	0/0
0/0	0/0	0/1	2/2	0/1	0/0	1/1	1/1	0/0	1/1	1/1	1/1	1/1	0/1	0/0	1/1	0/0	1/1	0/0	0/0	0/0	1/1	0/0	0/1	0/1	1/1	0/0

0/0	0/0	0/0	2/2	0/1	0/0	2/2	2/2	0/0	2/2	0/0	0/0	2/2	0/1	0/0	0/0	0/0	0/0	0/0	0/0	0/0	2/2	2/2	0/2	0/1	0/0	0/0
3/3	2/3	0/0	4/4	0/4	0/0	4/4	4/4	0/0	4/4	4/4	0/0	4/4	0/4	0/0	0/0	0/0	4/4	0/0	0/0	0/0	4/4	4/4	0/0	0/4	4/4	0/2
0/0	0/0	0/0	1/1	0/0	0/0	0/0	0/0	0/0	0/0	0/0	0/0	0/0	0/0	0/0	0/0	0/0	0/0	0/0	0/0	0/0	0/0	0/0	0/0	0/0	0/0	0/0
0/0	1/1	1/1	1/1	1/1	1/1	1/1	1/1	1/1	1/1	1/1	1/1	1/1	1/1	1/1	1/1	1/1	1/1	1/1	1/1	1/1	1/1	1/1	1/1	1/1	1/1	1/1
0/0	0/0	1/1	1/1	1/1	0/0	1/1	1/1	0/0	1/1	1/1	1/1	1/1	1/1	0/0	1/1	0/0	1/1	0/0	0/0	0/0	1/1	1/1	0/1	1/1	1/1	0/0
0/0	0/0	0/0	0/0	0/0	0/0	0/0	0/0	0/0	0/0	0/0	0/0	0/0	0/0	0/0	0/0	0/0	0/0	0/0	0/0	0/0	0/0	0/0	0/0	0/0	0/0	0/0
0/0	0/0	0/0	0/0	0/0	0/0	0/0	0/0	0/0	0/0	0/0	0/0	0/0	0/0	0/0	0/0	0/0	0/0	0/0	0/0	0/0	0/0	0/0	0/0	0/0	0/0	0/0
0/0	1/1	1/1	1/1	1/1	1/1	1/1	1/1	1/1	1/1	1/1	1/1	1/1	1/1	1/1	1/1	1/1	1/1	1/1	1/1	1/1	1/1	1/1	1/1	1/1	1/1	1/1
0/0	0/0	./.	./.	0/0	0/0	0/0	0/0	0/0	./.	0/0	./.	./.	./.	0/0	./.	0/0	./.	0/0	0/0	0/0	0/0	./.	0/0	./.	./.	0/0
0/0	0/0	1/1	1/2	1/1	0/0	1/1	1/1	0/1	1/1	1/1	1/1	1/1	1/1	0/0	1/1	0/0	1/1	0/0	0/0	0/0	0/1	1/1	0/1	1/1	1/1	0/0
0/0	0/0	1/1	1/1	1/1	0/0	1/1	1/1	0/1	1/1	1/1	1/1	1/1	1/1	0/0	1/1	0/0	1/1	0/0	0/0	0/0	0/1	1/1	0/1	1/1	1/1	0/0
0/0	0/0	1/1	0/0	1/1	0/0	1/1	1/1	0/1	1/1	1/1	1/1	1/1	1/1	0/0	1/1	0/0	1/1	0/0	0/0	0/0	0/1	1/1	0/1	1/1	1/1	0/0
0/0	1/1	0/0	0/0	0/0	1/1	0/0	0/0	0/1	0/0	0/0	0/0	0/0	0/0	1/1	0/0	1/1	0/0	1/1	1/1	1/1	0/1	0/0	0/1	0/0	0/0	1/1
0/0	0/0	1/1	1/1	1/1	0/0	1/1	1/1	0/0	1/1	1/1	1/1	1/1	1/1	0/0	1/1	0/0	1/1	0/0	0/0	0/0	0/1	1/1	0/1	1/1	1/1	0/0
0/0	1/1	1/1	1/1	1/1	1/1	1/1	1/1	1/1	1/1	1/1	1/1	1/1	1/1	1/1	1/1	1/1	1/1	1/1	1/1	1/1	1/1	1/1	1/1	1/1	1/1	1/1
0/0	0/0	0/0	0/0	0/0	0/0	0/0	0/0	0/0	0/0	0/0	0/0	0/0	0/0	0/0	0/0	0/0	0/0	0/0	0/0	0/0	0/0	0/0	0/0	0/0	0/0	0/0
0/0	0/0	0/0	0/0	0/0	0/0	0/0	0/0	0/0	0/0	0/0	0/0	0/0	0/0	0/0	0/0	0/0	0/0	0/0	0/0	0/0	0/0	0/0	0/0	0/0	0/0	0/0
0/0	1/1	0/0	0/0	0/0	1/1	0/0	0/0	1/1	0/0	0/0	0/0	0/0	0/0	1/1	0/0	1/1	0/0	1/1	1/1	1/1	0/1	0/0	0/1	0/0	0/0	1/1
0/0	0/0	0/0	0/0	0/0	0/0	0/0	0/0	0/0	0/0	0/0	0/0	0/0	0/0	0/0	0/0	0/0	0/0	0/0	0/0	0/0	0/0	0/0	0/0	0/0	0/0	0/0
0/0	1/1	0/0	0/0	0/0	1/1	0/0	0/0	1/1	0/0	0/0	0/0	0/0	0/0	1/1	0/0	1/1	0/0	1/1	1/1	1/1	0/1	0/0	0/1	0/0	0/0	1/1
0/0	0/0	1/1	0/0	1/1	0/0	1/1	0/0	0/0	1/1	0/0	1/1	1/1	0/0	0/0	1/1	0/0	1/1	0/0	0/0	0/0	0/0	1/1	0/0	1/1	0/0	0/0
0/0	1/1	0/0	0/0	0/0	1/1	0/0	0/0	1/1	0/0	0/0	0/0	0/0	0/0	1/1	0/0	1/1	0/0	1/1	1/1	1/1	0/1	0/0	0/1	0/0	0/0	1/1
0/0	1/1	0/0	0/0	0/0	1/1	0/0	0/0	0/1	0/0	0/0	0/0	0/0	0/0	1/1	0/0	1/1	0/0	1/1	1/1	1/1	0/0	0/0	0/1	0/0	0/0	1/1
0/0	0/0	0/0	1/1	0/0	0/0	0/0	0/0	0/0	0/0	0/0	0/0	0/0	0/0	0/0	0/0	0/0	0/0	0/0	0/0	0/0	0/0	0/0	0/0	0/0	0/0	0/0
0/0	0/0	0/0	1/1	0/0	0/0	0/0	0/0	0/0	0/0	0/0	0/0	0/0	0/0	0/0	0/0	0/0	0/0	0/0	0/0	0/0	0/0	0/0	0/0	0/0	0/0	0/0
0/0	0/0	1/1	1/1	1/1	0/0	1/1	1/1	0/0	1/1	1/1	1/1	1/1	1/1	0/0	1/1	0/0	1/1	0/0	0/0	0/0	1/1	1/1	0/1	1/1	1/1	0/0
0/0	0/0	0/0	0/0	0/0	0/0	0/0	0/0	0/0	0/0	0/0	0/0	0/0	0/0	0/0	0/0	0/0	0/0	0/0	0/0	0/0	0/0	0/0	0/0	0/0	0/0	0/0
0/0	1/1	1/1	1/1	1/1	1/1	1/1	1/1	1/1	1/1	1/1	1/1	1/1	1/1	1/1	1/1	1/1	1/1	1/1	1/1	1/1	1/1	1/1	1/1	1/1	1/1	1/1
0/0	0/0	0/0	0/0	0/0	0/0	0/0	0/0	0/0	0/0	0/0	0/0	0/0	0/0	0/0	0/0	0/0	0/0	0/0	0/0	0/0	0/0	0/0	0/0	0/0	0/0	0/0
0/0	1/1	1/1	1/1	1/1	1/1	1/1	1/1	1/1	1/1	1/1	1/1	1/1	1/1	1/1	1/1	1/1	1/1	1/1	1/1	1/1	1/1	1/1	1/1	1/1	1/1	1/1

0/0	0/0	0/0	1/1	0/0	0/0	0/0	0/0	0/0	0/0	0/0	0/0	0/0	0/0	0/0	0/0	0/0	0/0	0/0	0/0	0/0	0/0	0/0	0/0	0/0	0/0	0/0
0/0	0/0	0/0	0/0	0/0	0/0	0/0	0/0	0/0	0/0	0/0	0/0	0/0	0/0	0/0	0/0	0/0	0/0	0/0	0/0	0/0	0/0	0/0	0/0	0/0	0/0	0/0
0/0	0/0	0/0	0/0	0/0	0/0	0/0	0/0	0/0	0/0	0/0	0/0	0/0	0/0	0/0	0/0	0/0	0/0	0/0	0/0	0/0	0/0	0/0	0/0	0/0	0/0	0/0
0/0	0/0	0/0	0/0	0/0	0/0	0/0	1/1	0/0	0/0	1/1	0/0	0/0	1/1	0/0	0/0	0/0	0/0	0/0	0/0	0/0	1/1	0/0	0/1	0/0	1/1	0/0
0/0	0/0	1/1	1/1	1/1	0/0	1/1	1/1	0/0	1/1	1/1	1/1	1/1	1/1	0/0	1/1	0/0	1/1	0/0	0/0	0/0	1/1	1/1	0/1	1/1	1/1	0/0
0/0	1/1	1/1	1/1	1/1	1/1	1/1	1/1	1/1	1/1	1/1	1/1	1/1	1/1	1/1	1/1	1/1	1/1	1/1	1/1	1/1	1/1	1/1	1/1	1/1	1/1	1/1
0/0	0/0	1/1	1/1	1/1	0/0	1/1	1/1	0/0	1/1	1/1	1/1	1/1	1/1	0/0	1/1	0/0	1/1	0/0	0/0	0/0	1/1	1/1	0/1	1/1	1/1	0/0
0/0	0/0	1/1	0/0	1/1	0/0	1/1	0/0	0/0	1/1	0/0	1/1	1/1	0/0	0/0	1/1	0/0	1/1	0/0	0/0	0/0	0/0	1/1	0/0	1/1	0/0	0/0
0/0	0/0	0/0	0/0	0/0	0/0	0/0	0/0	0/0	0/0	0/0	0/0	0/0	0/0	0/0	0/0	0/0	0/0	0/0	0/0	0/0	0/0	0/0	0/0	0/0	0/0	0/0
0/0	0/0	0/0	0/0	0/0	0/0	0/0	0/0	0/0	0/0	0/0	0/0	0/0	0/0	0/0	0/0	0/0	0/0	0/0	0/1	0/0	0/0	0/0	0/0	0/0	0/0	0/0
0/0	0/0	0/0	0/0	0/0	0/0	0/0	0/0	0/0	0/0	0/0	0/0	0/0	0/0	0/0	0/0	1/1	0/0	0/0	0/0	0/0	0/0	0/0	0/0	0/0	0/0	0/0
0/0	1/1	0/0	0/0	0/0	1/1	0/0	0/1	1/1	0/0	0/0	0/0	0/0	0/0	1/1	0/0	1/1	0/0	1/1	1/1	1/1	0/0	0/0	0/1	0/0	0/0	1/1
0/0	1/1	0/0	2/2	0/0	1/1	0/0	1/1	1/1	0/0	1/1	0/0	0/0	1/1	0/0	0/0	1/1	0/0	1/1	0/1	1/1	1/1	0/0	1/1	0/0	1/1	0/0
0/0	1/1	1/1	1/1	1/1	1/1	1/1	1/1	1/1	1/1	1/1	1/1	1/1	1/1	1/1	1/1	1/1	1/1	1/1	1/1	1/1	1/1	1/1	1/1	1/1	1/1	1/1
1/1	1/1	1/1	1/1	1/1	1/1	1/1	1/1	1/1	1/1	1/1	1/1	1/1	1/1	1/1	1/1	1/1	1/1	1/1	1/1	1/1	1/1	1/1	1/1	1/1	1/1	1/1
0/0	0/0	0/0	0/0	0/0	0/0	0/0	0/0	0/0	0/0	0/0	0/0	0/0	0/0	0/0	0/0	0/0	0/0	0/0	0/0	0/0	0/0	0/0	0/0	0/0	0/0	0/0
0/0	0/0	0/0	0/0	0/0	0/0	0/0	0/1	0/0	0/0	1/1	0/0	0/0	1/1	0/0	0/0	0/0	0/0	0/0	0/0	0/0	1/1	0/0	0/1	0/0	1/1	0/0
0/0	0/0	1/1	1/1	1/1	0/0	1/1	0/1	0/0	1/1	1/1	1/1	1/1	1/1	0/0	1/1	0/0	1/1	0/0	0/0	0/0	1/1	1/1	0/1	1/1	1/1	0/0
0/0	1/1	1/1	1/1	1/1	1/1	1/1	1/1	1/1	1/1	1/1	1/1	1/1	1/1	1/1	1/1	1/1	1/1	1/1	1/1	1/1	1/1	1/1	1/1	1/1	1/1	1/1
0/0	1/1	0/0	0/0	0/0	0/0	0/0	0/0	0/0	0/0	0/0	0/0	0/0	0/0	0/0	0/0	0/0	0/0	0/0	0/0	0/0	0/0	0/0	0/0	0/0	0/0	0/0
0/0	0/0	0/0	0/0	0/0	0/0	0/0	0/0	0/0	0/0	0/0	0/0	0/0	0/0	0/0	0/0	0/0	0/0	0/0	0/0	0/0	0/0	0/0	0/0	0/0	0/0	0/0
0/0	0/0	0/0	1/1	0/0	0/0	0/0	0/0	0/0	0/0	0/0	0/0	0/0	0/0	0/0	0/0	0/0	0/0	0/0	0/0	0/0	0/0	0/0	0/0	0/0	0/0	0/0
0/0	1/1	1/1	1/1	1/1	1/1	1/1	1/1	1/1	1/1	1/1	1/1	1/1	1/1	1/1	1/1	1/1	1/1	1/1	1/1	1/1	1/1	1/1	1/1	1/1	1/1	1/1
0/0	0/0	0/0	0/0	0/0	0/0	0/0	0/0	0/0	0/0	0/0	0/0	0/0	0/0	0/0	0/0	0/0	0/0	0/0	0/0	0/0	0/0	0/0	0/0	0/0	0/0	0/0
0/0	0/0	0/0	1/1	0/0	0/0	0/0	0/0	0/0	0/0	0/0	0/0	0/0	0/0	0/0	0/0	0/0	0/0	0/0	0/0	0/0	0/0	0/0	0/0	0/0	0/0	0/0
0/0	0/0	0/0	0/0	0/0	0/0	0/0	0/0	0/0	0/0	0/0	0/0	0/0	0/0	0/0	0/0	0/0	0/0	0/0	0/0	0/0	0/0	0/0	0/0	0/0	0/0	0/0
0/0	0/0	0/0	1/1	0/0	0/0	0/0	0/0	0/0	0/0	0/0	0/0	0/0	0/0	0/0	0/0	0/0	0/0	0/0	0/0	0/0	0/0	0/0	0/0	0/0	0/0	0/0
0/0	0/0	0/0	0/0	0/0	0/0	0/0	0/0	0/0	0/0	0/0	0/0	0/0	0/0	0/0	0/0	0/0	0/0	0/0	0/0	0/0	0/0	0/0	0/0	0/0	0/0	0/0
0/0	0/0	0/0	0/0	0/0	0/0	0/0	0/0	0/0	0/0	0/0	0/0	0/0	0/0	0/0	0/0	0/0	0/0	0/0	0/0	0/0	0/0	0/0	0/0	0/0	0/0	0/0
0/0	0/0	0/0	0/0	0/0	0/0	0/0	0/0	0/0	0/0	0/0	0/0	0/0	0/0	0/0	0/0	0/0	0/0	./.	0/0	0/0	0/0	0/0	0/0	0/0	0/0	0/0
0/0	0/0	0/0	0/0	0/0	0/0	0/0	0/0	0/0	0/0	0/0	0/0	0/0	0/0	0/0	0/0	0/0	0/0	1/1	0/0	0/0	0/0	0/0	0/0	0/0	0/0	0/0
0/0	1/1	0/0	0/0	0/0	1/1	0/0	0/0	1/1	0/0	0/0	0/0	0/0	0/0	1/1	0/0	1/1	0/0	1/1	1/1	1/1	0/0	0/0	0/1	0/0	0/0	1/1
0/0	0/0	0/0	0/0	0/0	0/0	0/0	0/0	0/0	0/0	0/0	0/0	0/0	0/0	0/0	0/0	0/0	0/0	0/0	0/0	0/0	0/0	0/0	0/0	0/0	0/0	0/0
0/0	0/0	0/0	0/0	0/0	0/0	0/0	0/0	0/0	0/0	0/0	0/0	0/0	0/0	0/0	0/0	0/0	0/0	0/0	0/0	0/0	0/0	0/0	0/0	0/0	0/0	0/0
0/0	0/0	0/0	0/0	0/0	0/0	0/0	1/1	0/0	0/0	1/1	0/0	0/0	1/1	0/0	0/0	0/0	0/0	0/0	0/0	0/0	0/0	0/0	0/1	0/0	1/1	0/0

0/0	0/0	1/1	0/0	1/1	0/0	1/1	0/0	0/0	1/1	0/0	1/1	1/1	0/0	0/0	1/1	0/0	1/1	0/0	0/0	0/0	0/0	1/1	0/0	1/1	0/0	0/0
2/3	1/1	1/4	4/4	4/4	1/2	4/4	2/2	1/1	4/4	2/3	4/4	4/4	1/2	1/1	4/4	2/3	4/4	1/4	1/2	1/1	2/2	4/4	1/2	4/4	2/2	1/1
./.	./.	./.	./.	./.	./.	./.	./.	./.	./.	./.	./.	./.	./.	./.	./.	./.	./.	./.	./.	./.	./.	0/0	./.	./.	./.	./.
0/0	0/0	0/0	0/0	0/0	0/0	0/0	0/0	0/0	0/0	0/0	0/0	0/0	0/0	0/0	0/0	0/0	0/0	0/0	0/0	0/0	0/0	0/0	0/0	0/0	0/0	0/0
0/0	0/0	0/0	0/0	0/0	0/0	0/0	0/0	0/0	0/0	0/0	0/0	0/0	0/0	0/0	0/0	0/0	0/0	0/0	0/0	0/0	0/0	0/0	0/0	0/0	0/0	0/0
0/0	0/0	0/0	1/1	0/0	0/0	0/0	0/0	0/0	0/0	0/0	0/0	0/0	0/0	0/0	0/0	0/0	0/0	0/0	0/0	0/0	0/0	0/0	0/0	0/0	0/0	0/0
0/0	0/0	1/1	0/0	1/1	0/0	1/1	0/0	0/0	1/1	0/0	1/1	1/1	0/0	0/0	1/1	0/0	1/1	0/0	0/0	0/0	0/0	1/1	0/0	1/1	0/0	0/0
0/0	0/0	1/1	0/0	1/1	./.	1/1	0/0	0/0	1/1	0/0	1/1	1/1	0/0	0/0	1/1	0/0	1/1	0/0	./.	0/0	0/0	1/1	0/0	1/1	0/0	0/0
0/0	0/0	0/0	1/1	0/0	./.	0/0	0/0	0/0	./.	0/0	0/0	0/0	0/0	0/0	0/0	0/0	0/0	0/0	./.	./.	0/0	0/0	0/0	0/0	0/0	0/0
./.	0/1	1/1	1/1	1/1	./.	1/1	1/1	0/0	1/1	1/1	1/1	1/1	1/1	0/1	1/1	./.	1/1	./.	0/1	./.	1/1	1/1	0/1	1/1	1/1	0/0
1/1	./.	./.	./.	./.	2/2	./.	./.	2/2	./.	./.	./.	./.	./.	0/2	./.	2/2	./.	2/2	0/2	2/2	./.	./.	0/2	./.	./.	2/2
2/2	0/1	./.	./.	./.	./.	0/0	0/0	./.	0/0	./.	0/0	0/0	0/0	./.	./.	./.	0/0	./.	./.	./.	0/0	0/0	./.	./.	0/0	./.
0/0	0/0	0/0	1/1	0/0	0/0	0/0	0/0	0/0	0/0	0/0	0/0	0/0	0/0	0/0	0/0	0/0	0/0	0/0	0/0	0/0	0/0	0/0	0/0	0/0	0/0	0/0
0/0	0/0	0/0	0/0	0/0	0/0	0/0	0/0	0/0	0/0	0/0	0/0	0/0	0/0	0/0	0/0	0/0	0/0	0/0	0/0	0/0	0/0	0/0	0/0	0/0	0/0	0/0
0/0	0/0	0/0	0/0	0/0	0/0	0/0	0/0	0/0	0/0	0/0	0/0	0/0	0/0	0/0	0/0	0/0	0/0	0/0	0/0	0/0	0/0	0/0	0/0	0/0	0/0	0/0
0/0	1/1	1/1	1/1	1/1	1/1	1/1	1/1	1/1	1/1	1/1	1/1	1/1	1/1	1/1	1/1	1/1	1/1	1/1	1/1	1/1	1/1	1/1	1/1	1/1	1/1	1/1
0/0	0/0	0/0	0/0	0/0	0/0	0/0	0/0	0/0	0/0	0/0	0/0	0/0	0/0	0/0	0/0	0/0	0/0	0/0	0/0	0/0	0/0	0/0	0/0	0/0	0/0	0/0
0/0	0/0	0/0	0/0	0/0	0/0	0/0	1/1	0/0	0/0	1/1	0/0	0/0	1/1	0/0	0/0	0/0	0/0	0/0	0/0	0/0	1/1	0/0	0/1	0/0	1/1	0/0
0/0	0/0	0/0	0/0	0/0	0/0	0/0	0/0	0/0	0/0	0/0	0/0	0/0	0/0	0/0	0/0	0/0	0/0	0/0	0/0	0/0	0/0	0/0	0/0	0/0	0/0	0/0
0/0	0/0	0/0	0/0	0/0	0/0	0/0	0/0	0/0	0/0	0/0	0/0	0/0	0/0	0/0	0/0	0/0	0/0	0/0	0/0	0/0	0/0	0/0	0/0	0/0	0/0	0/0
0/0	0/0	0/0	0/0	0/0	0/0	0/0	0/0	0/0	0/0	0/0	0/0	0/0	0/0	0/0	0/0	0/0	0/0	0/0	0/0	0/0	0/0	0/0	0/0	0/0	0/0	0/0

0/0	0/0	0/0	0/0	0/0	0/0	0/0	0/0	0/0	0/0	0/0	0/0	0/0	0/0	0/0	0/0	0/0	0/0	0/0	0/0	0/0	0/0	0/0	0/0	0/0	0/0	0/0
0/0	0/0	0/0	0/0	0/0	0/0	0/0	0/0	0/0	0/0	0/0	0/0	0/0	0/0	0/0	0/0	0/0	0/0	0/0	0/0	0/0	0/0	0/0	0/0	0/0	0/0	0/0
0/0	2/2	1/1	1/1	1/1	2/2	1/1	1/1	2/2	1/1	1/1	1/1	1/1	1/1	2/2	1/1	2/2	1/1	2/2	2/2	2/2	1/1	1/1	1/2	1/1	1/1	2/2
0/0	0/0	0/0	0/0	0/0	0/0	0/0	0/0	0/0	0/0	0/0	0/0	0/0	0/0	0/0	0/0	0/0	0/0	0/0	0/0	0/0	0/0	0/0	0/0	0/0	0/0	0/0
0/0	2/2	0/0	0/0	0/0	2/2	0/0	1/1	2/2	0/0	1/1	0/0	0/0	1/1	2/2	0/0	2/2	0/0	2/2	2/2	2/2	0/1	0/0	1/2	0/0	1/1	2/2
0/0	0/0	0/0	0/0	0/0	0/0	0/0	0/0	0/0	0/0	0/0	0/0	0/0	0/0	0/0	0/0	0/0	0/0	0/0	0/0	0/0	0/0	0/0	0/0	0/0	0/0	0/0
0/0	0/0	1/1	0/0	1/1	0/0	1/1	0/0	0/0	1/1	0/0	1/1	1/1	0/0	0/0	0/0	0/0	0/0	0/0	0/0	0/0	0/0	1/1	0/0	1/1	0/0	0/0
0/0	1/1	1/1	1/1	1/1	1/1	1/1	1/1	1/1	1/1	1/1	1/1	1/1	1/1	1/1	1/1	1/1	1/1	1/1	1/1	1/1	1/1	1/1	1/1	1/1	1/1	1/1
0/0	1/1	1/1	1/1	1/1	1/1	1/1	1/1	1/1	1/1	1/1	1/1	1/1	1/1	1/1	1/1	1/1	1/1	1/1	1/1	1/1	1/1	1/1	1/1	1/1	1/1	1/1
0/0	./.	0/0	./.	0/0	0/0	0/0	0/0	0/0	0/0	0/0	0/0	0/0	0/0	0/0	0/0	0/0	0/0	0/0	0/0	0/0	0/0	0/0	0/0	0/0	0/0	0/0
0/0	2/2	2/2	2/2	2/2	2/2	2/2	1/1	2/2	2/2	1/1	2/2	2/2	1/1	2/2	2/2	2/2	2/2	2/2	2/2	2/2	1/1	2/2	1/2	2/2	1/1	2/2
0/0	0/0	0/0	0/0	1/1	0/0	0/0	0/0	0/0	0/0	0/0	0/0	0/0	0/0	0/0	0/0	0/0	0/0	0/0	0/0	0/0	0/0	0/0	0/0	0/0	0/0	0/0
0/0	0/0	0/0	0/0	0/0	0/0	1/1	0/0	0/0	0/0	0/0	0/0	0/0	0/0	0/0	0/0	0/0	0/0	0/0	0/0	0/0	0/0	0/0	0/0	1/1	0/0	0/0
0/0	0/0	0/0	0/0	0/0	0/0	0/0	0/0	0/0	0/0	0/0	0/0	0/0	0/0	0/0	0/0	0/0	0/0	0/0	0/0	0/0	0/0	0/0	0/0	0/0	0/0	0/0
0/0	0/0	1/1	1/1	1/1	0/0	1/1	0/0	0/0	1/1	0/0	1/1	1/1	0/0	0/0	1/1	0/0	1/1	0/0	0/0	0/0	0/0	1/1	0/0	1/1	0/0	0/0
0/0	0/0	0/0	0/0	0/0	0/0	0/0	0/0	0/0	0/0	0/0	0/0	0/0	0/0	0/0	0/0	0/0	0/0	0/0	0/0	0/0	0/0	0/0	0/0	0/0	0/0	0/0
0/0	0/0	1/1	0/0	1/1	0/0	1/1	0/0	0/0	1/1	0/0	1/1	1/1	0/0	0/0	1/1	0/0	1/1	0/0	0/0	0/0	0/0	1/1	0/0	1/1	0/0	0/0
0/0	1/1	1/1	1/1	1/1	1/1	1/1	1/1	1/1	1/1	1/1	1/1	1/1	1/1	1/1	1/1	1/1	1/1	1/1	1/1	1/1	1/1	1/1	1/1	1/1	1/1	1/1

Accession code																										
682	684	687	689	690	692	696	6	700	701	702	706	713	717	725	731	745	752	761	773	77	78	794	798	79	7	810
0/0	0/0	0/0	0/0	0/0	0/0	0/0	0/0	0/0	0/0	0/0	0/0	0/0	0/0	0/0	0/0	0/0	0/0	0/0	0/0	0/0	0/0	0/0	0/0	0/0	0/0	0/0
0/0	0/0	0/0	1/1	0/0	0/0	0/0	0/0	0/0	0/0	0/0	0/0	1/1	0/0	0/0	0/0	0/0	0/0	0/0	1/1	0/0	1/1	1/1	1/1	1/1	0/0	0/0
0/0	0/0	0/0	0/0	0/0	0/0	0/0	0/0	0/0	0/0	0/0	0/0	0/0	0/0	0/0	0/0	0/0	0/0	0/0	0/0	0/0	0/0	0/0	0/0	0/0	0/0	0/0
0/0	0/0	0/0	0/0	0/0	1/1	0/0	0/0	1/1	0/0	0/0	0/0	0/0	0/0	0/0	0/0	0/0	0/0	0/0	0/0	0/0	0/0	0/0	0/0	0/0	0/0	0/0
0/0	1/1	1/1	0/0	1/1	0/0	0/0	1/1	0/0	1/1	0/0	1/1	0/0	0/0	1/1	1/1	1/1	1/1	0/0	0/0	1/1	0/0	0/0	0/0	0/0	1/1	0/0
0/0	1/1	1/1	0/0	1/1	1/1	0/0	1/1	1/1	1/1	0/0	1/1	0/0	0/0	1/1	1/1	1/1	1/1	0/0	0/0	1/1	0/0	0/0	0/0	0/0	1/1	0/0
0/0	0/0	0/0	0/0	0/0	0/0	0/0	0/0	0/0	0/0	0/0	0/0	0/0	0/0	0/0	0/0	0/0	0/0	0/0	0/0	0/0	0/0	0/0	0/0	0/0	0/0	0/0
1/1	0/0	0/0	1/1	0/0	0/0	1/1	0/0	0/0	0/0	0/0	0/0	1/1	1/1	0/0	0/0	0/0	0/0	0/0	1/1	0/0	1/1	1/1	1/1	1/1	0/0	0/0
0/0	0/0	0/0	0/0	0/0	0/0	0/0	0/0	0/0	0/0	0/0	0/0	0/0	0/0	0/0	0/0	0/0	0/0	0/0	0/0	0/0	0/0	0/0	0/0	0/0	0/0	0/0
0/1	0/0	0/0	0/0	0/1	0/0	0/0	0/0	0/0	0/0	0/0	0/0	0/0	0/0	0/0	0/0	0/0	0/0	0/0	0/0	0/0	0/0	0/0	0/0	0/0	0/0	0/0
0/1	0/0	0/0	0/0	0/0	0/0	0/0	0/0	0/0	0/0	0/0	0/0	0/1	0/0	0/0	0/0	0/0	0/0	0/0	0/0	0/0	0/0	0/0	0/0	0/0	0/0	0/0
0/0	0/0	0/0	0/0	0/0	0/0	0/0	0/0	0/0	0/0	0/0	0/0	0/0	0/0	0/0	0/0	0/0	0/0	0/0	0/0	0/0	0/0	0/0	0/0	0/0	0/0	0/0
0/0	1/1	1/1	0/0	1/1	0/0	0/0	1/1	0/0	1/1	0/0	1/1	0/0	0/0	1/1	1/1	1/1	1/1	0/0	0/0	1/1	0/0	0/0	0/0	0/0	1/1	0/0
./.	./.	0/0	0/1	0/0	0/0	0/0	./.	0/0	0/0	./.	0/0	0/0	0/0	0/0	0/0	0/0	0/0	0/0	0/0	0/0	0/0	1/1	0/0	0/0	0/0	0/0
1/1	0/0	0/0	0/1	0/0	./.	1/1	./.	0/0	./.	./.	0/0	1/1	1/1	./.	0/0	0/0	./.	0/0	1/1	./.	1/1	0/0	0/0	1/1	0/0	0/0
0/0	0/0	0/0	0/0	0/0	./.	0/0	./.	0/0	./.	./.	0/0	0/0	0/0	./.	0/0	0/0	./.	0/0	./.	0/0	0/0	0/0	0/0	0/0	0/0	0/0

1/1	2/2	1/2	3/5	6/6	./.	0/0	0/2	3/3	2/2	2/3	2/3	6/6	0/1	./.	0/1	6/6	3/3	0/1	./.	3/3	0/1	5/5	0/4	0/1	2/2	0/1
0/0	0/0	0/0	0/0	0/0	0/0	0/0	0/0	0/0	0/0	0/0	0/0	0/0	0/0	0/0	0/0	0/0	0/0	0/0	0/0	0/0	0/0	0/0	0/0	0/0	0/0	0/0
0/0	0/0	0/0	0/0	./.	0/0	0/0	0/0	0/0	0/0	0/0	0/0	0/0	0/0	0/0	0/0	./.	0/0	0/0	0/0	0/0	0/0	0/0	0/0	0/0	0/0	0/0
0/0	1/1	1/1	0/0	1/1	0/0	0/0	1/1	0/0	1/1	0/0	1/1	0/0	0/0	1/1	0/1	1/1	1/1	0/0	0/0	1/1	0/0	0/0	0/0	0/0	1/1	0/0
1/1	0/0	0/0	1/1	0/0	0/0	1/1	0/0	0/0	0/0	0/0	0/0	1/1	1/1	0/0	0/0	0/0	0/0	0/0	1/1	0/0	1/1	1/1	0/0	1/1	0/0	0/0
0/0	0/0	0/0	0/0	0/0	1/1	0/0	0/0	1/1	0/0	0/0	0/0	0/0	0/0	0/0	0/0	0/0	0/0	0/0	0/0	0/0	0/0	0/0	0/0	0/0	0/0	0/0
1/1	0/0	0/0	1/1	0/0	0/0	1/1	0/0	0/0	0/0	0/0	0/0	1/1	1/1	0/0	0/0	0/0	0/0	0/0	1/1	0/0	1/1	1/1	0/0	1/1	0/0	0/0
0/0	1/1	1/1	0/0	1/1	0/0	0/0	1/1	0/0	./.	0/0	1/1	0/0	0/0	1/1	1/1	1/1	1/1	0/0	0/0	1/1	0/0	0/0	0/0	0/0	1/1	0/0
0/0	0/0	0/0	0/0	0/0	1/1	0/0	0/0	1/1	./.	1/1	0/0	0/0	0/0	0/0	0/0	0/0	0/0	0/0	0/0	0/0	0/0	0/0	0/0	0/0	0/0	1/1
1/1	1/1	1/1	0/0	1/1	2/2	1/1	1/1	2/2	./.	2/2	1/1	0/0	1/1	1/1	1/1	1/1	1/1	0/0	0/0	1/1	0/0	0/0	0/0	0/0	1/1	2/2
0/0	0/0	0/0	0/0	0/0	1/1	0/0	0/0	1/1	./.	./.	0/0	0/0	0/0	0/0	0/0	0/0	0/0	0/0	0/0	0/0	0/0	0/0	0/0	0/0	0/0	./.
0/0	1/1	1/1	0/0	1/1	0/0	0/0	1/1	0/0	./.	0/0	1/1	0/0	0/0	1/1	1/1	1/1	1/1	0/0	0/0	1/1	0/0	0/0	0/0	0/0	1/1	0/0
1/1	1/1	1/1	1/1	1/1	1/1	1/1	1/1	1/1	1/1	0/0	1/1	1/1	1/1	1/1	1/1	1/1	1/1	0/0	1/1	1/1	1/1	1/1	0/0	1/1	1/1	0/0
0/0	0/0	0/0	0/0	0/0	1/1	0/0	0/0	1/1	0/0	0/0	0/0	0/0	0/0	0/0	0/0	0/0	0/0	0/0	0/0	0/0	0/0	0/0	0/0	0/0	0/0	0/0
0/0	0/0	0/0	0/0	0/0	0/0	0/0	0/0	0/0	0/0	0/0	0/0	0/0	0/0	0/0	0/0	0/0	0/0	0/0	0/0	0/0	0/0	0/0	0/0	0/0	0/0	0/0
1/1	1/1	1/1	1/1	1/1	1/1	1/1	1/1	1/1	1/1	0/0	1/1	1/1	1/1	1/1	1/1	1/1	1/1	0/0	1/1	1/1	1/1	1/1	0/0	1/1	1/1	0/0
0/0	1/1	1/1	0/0	1/1	0/0	0/0	1/1	0/0	1/1	0/0	1/1	0/0	0/0	1/1	1/1	1/1	1/1	0/0	0/0	1/1	0/0	0/0	0/0	0/0	1/1	0/0
0/0	0/0	0/0	0/0	0/0	1/1	0/0	0/0	1/1	0/0	0/0	0/0	0/0	0/0	0/0	0/0	0/0	0/0	0/0	0/0	0/0	0/0	0/0	0/0	0/0	0/0	0/0
1/1	0/0	0/0	1/1	0/0	0/0	1/1	0/0	0/0	0/0	0/0	0/0	1/1	1/1	0/0	0/0	0/0	0/0	0/0	1/1	0/0	1/1	1/1	0/0	1/1	0/0	0/0
0/0	0/0	0/0	1/1	0/0	0/0	0/0	0/0	0/0	0/0	0/0	0/0	1/1	0/0	0/0	0/0	0/0	0/0	0/0	1/1	0/0	1/1	1/1	0/0	1/1	0/0	0/0
0/0	0/0	0/0	0/0	0/0	1/1	0/0	0/0	1/1	0/0	0/0	0/0	0/0	0/0	0/0	0/0	0/0	0/0	0/0	0/0	0/0	0/0	0/0	0/0	0/0	0/0	0/0
1/1	0/0	0/0	1/1	0/0	0/0	1/1	0/0	0/0	0/0	0/0	0/0	1/1	1/1	0/0	0/0	0/0	0/0	0/0	1/1	0/1	1/1	1/1	0/0	1/1	0/0	0/0
0/0	0/0	0/0	0/0	0/0	1/1	0/0	0/0	1/1	0/0	0/0	0/0	0/0	0/0	0/0	0/0	0/0	0/0	0/0	0/0	0/0	0/0	0/0	0/0	0/0	0/0	0/0
1/1	1/1	1/1	1/1	1/1	1/1	1/1	1/1	1/1	1/1	0/0	1/1	1/1	1/1	1/1	1/1	1/1	1/1	0/0	1/1	1/1	1/1	1/1	0/0	1/1	1/1	0/0
0/0	1/1	1/1	0/0	1/1	0/0	0/0	1/1	0/0	1/1	0/0	1/1	0/0	0/0	1/1	1/1	1/1	1/1	0/0	0/0	0/1	0/0	0/0	0/0	0/0	1/1	0/0
0/0	0/0	0/0	1/1	0/0	2/2	0/0	0/0	2/2	0/0	0/0	0/0	1/1	0/0	0/0	0/0	0/1	0/0	0/0	1/1	0/0	1/1	1/1	0/0	1/1	0/0	1/1

1/1	1/1	1/1	1/1	1/1	1/1	1/1	1/1	1/1	1/1	1/1	1/1	1/1	1/1	1/1	1/1	1/1	1/1	0/0	1/1	1/1	1/1	1/1	1/1	1/1	1/1	1/1
0/0	1/1	1/1	0/0	1/1	1/1	0/0	1/1	1/1	1/1	0/0	1/1	0/0	0/0	1/1	1/1	1/1	1/1	0/0	0/0	1/1	0/0	0/0	0/0	0/0	1/1	0/0
0/0	1/1	1/1	0/0	1/1	0/0	0/0	1/1	0/0	1/1	0/0	1/1	0/0	0/0	1/1	1/1	1/1	1/1	0/0	0/0	1/1	0/0	0/0	0/0	0/0	1/1	0/0
0/0	0/0	0/0	0/0	0/0	0/0	0/0	0/0	0/0	0/0	0/0	0/0	0/0	0/0	0/0	0/0	0/0	0/0	0/0	0/0	0/0	0/0	0/0	0/0	0/0	0/0	0/0
1/1	1/1	1/1	1/1	1/1	1/1	1/1	1/1	1/1	1/1	0/0	1/1	1/1	1/1	1/1	1/1	1/1	1/1	0/0	1/1	1/1	1/1	1/1	0/0	1/1	1/1	0/0
1/1	1/1	1/1	1/1	1/1	1/1	1/1	1/1	1/1	1/1	0/0	1/1	1/1	1/1	1/1	1/1	1/1	1/1	0/0	1/1	1/1	1/1	1/1	0/0	1/1	1/1	0/0
1/1	1/1	1/1	1/1	1/1	1/1	1/1	1/1	1/1	1/1	0/0	1/1	1/1	1/1	1/1	1/1	1/1	1/1	0/0	1/1	1/1	1/1	1/1	0/0	1/1	1/1	0/0
0/0	0/0	0/0	0/0	0/0	./.	0/0	0/0	./.	./.	0/0	0/0	0/0	0/0	0/0	0/0	./.	0/0	0/0	0/0	0/0	0/0	0/0	0/0	0/0	./.	0/0
0/0	1/1	1/1	0/0	1/1	1/1	0/0	1/1	1/1	1/1	0/0	1/1	0/0	0/0	1/1	1/1	1/1	1/1	0/0	0/0	1/1	0/0	0/0	0/0	0/0	1/1	0/0
0/0	0/0	0/0	0/0	0/0	0/0	0/0	0/0	0/0	0/0	0/0	0/0	0/0	0/0	0/0	0/0	0/0	0/0	0/0	0/0	0/0	0/0	0/0	0/0	0/0	0/0	0/0
1/1	0/0	0/0	1/1	0/0	0/0	1/1	0/0	0/0	0/0	0/0	0/0	1/1	1/1	0/0	0/0	0/0	0/0	0/0	1/1	0/0	1/1	1/1	0/0	1/1	0/0	0/0
0/0	0/0	0/0	0/0	0/0	0/0	0/0	0/0	0/0	0/0	0/0	0/0	0/0	0/0	0/0	0/0	0/0	0/0	0/0	0/0	0/0	0/0	0/0	0/0	0/0	0/0	0/0
0/0	0/0	0/0	0/0	0/0	0/0	0/0	0/0	0/0	0/0	0/0	0/0	0/0	0/0	0/0	0/0	0/0	0/0	0/0	0/0	0/0	0/0	0/0	0/0	0/0	0/0	0/0
1/1	0/0	0/0	0/0	0/0	0/0	1/1	0/0	0/0	0/0	0/0	0/0	0/0	1/1	0/0	0/0	0/0	0/0	0/0	0/0	0/0	0/0	0/0	0/0	0/0	0/0	0/0
1/1	1/1	1/1	1/1	1/1	1/1	1/1	1/1	1/1	1/1	0/0	1/1	1/1	1/1	1/1	1/1	1/1	1/1	0/0	1/1	1/1	1/1	1/1	0/0	1/1	1/1	0/0
0/0	0/0	0/0	0/0	0/0	0/0	0/0	0/0	0/0	0/0	0/0	0/0	0/0	0/0	0/0	0/0	0/0	0/0	0/0	0/0	0/0	0/0	0/0	0/0	0/0	0/0	0/0
0/0	0/0	0/0	0/0	0/0	0/0	0/0	0/0	0/0	0/0	0/0	0/0	0/0	0/0	0/0	0/0	0/0	0/0	0/0	0/0	0/0	0/0	0/0	0/0	0/0	0/0	0/0
0/0	0/0	0/0	0/0	0/0	0/0	0/0	0/0	0/0	0/0	0/0	0/0	0/0	0/0	0/0	0/0	0/0	0/0	0/0	0/0	1/1	0/0	0/0	0/0	0/0	0/0	0/0
1/1	0/0	0/0	1/1	0/0	0/0	1/1	0/0	0/0	0/0	0/0	0/0	1/1	1/1	0/0	0/0	0/0	0/0	0/0	1/1	0/0	1/1	1/1	0/0	1/1	0/0	0/0
0/0	0/0	0/0	0/0	0/0	0/0	0/0	0/0	0/0	0/0	0/0	0/0	0/0	0/0	0/0	0/0	0/0	0/0	0/0	0/0	0/0	0/0	0/0	0/0	0/0	0/0	0/0
1/1	1/1	1/1	1/1	1/1	1/1	1/1	1/1	1/1	1/1	0/0	1/1	1/1	1/1	1/1	1/1	1/1	1/1	0/0	1/1	1/1	1/1	1/1	0/0	1/1	1/1	0/0
1/1	0/0	0/0	1/1	0/0	0/0	1/1	0/0	0/0	0/0	0/0	0/0	1/1	1/1	0/0	0/0	0/0	0/0	0/0	1/1	0/0	1/1	1/1	0/0	1/1	0/0	0/0
1/1	0/0	0/0	1/1	0/0	0/0	1/1	0/0	0/0	0/0	0/0	0/0	1/1	1/1	0/0	0/0	0/0	0/0	0/0	1/1	0/0	1/1	1/1	0/0	1/1	0/0	0/0
0/0	1/1	1/1	0/0	1/1	1/1	0/0	1/1	1/1	1/1	0/0	1/1	0/0	0/0	1/1	1/1	1/1	1/1	0/0	0/0	1/1	0/0	0/0	0/0	0/0	1/1	0/0
1/1	1/1	1/1	1/1	1/1	1/1	1/1	1/1	1/1	1/1	0/0	1/1	1/1	1/1	1/1	1/1	1/1	1/1	0/0	1/1	1/1	1/1	1/1	0/0	1/1	1/1	0/0
0/0	1/1	1/1	0/0	1/1	0/0	0/0	1/1	0/0	1/1	0/0	1/1	0/0	0/0	1/1	0/1	1/1	1/1	0/0	0/0	1/1	0/0	0/0	0/0	0/0	1/1	0/0
0/0	0/0	0/0	0/0	0/0	0/0	0/0	0/0	0/0	0/0	0/0	0/0	0/0	0/0	0/0	0/0	0/0	0/0	0/0	0/0	0/0	0/0	0/0	0/0	0/0	0/0	0/0
0/0	0/0	0/0	0/0	0/0	1/1	./.	0/0	1/1	0/0	0/0	0/0	0/0	0/0	0/0	0/0	0/0	0/0	0/0	0/0	0/0	0/0	0/0	0/0	0/0	0/0	0/0
1/1	1/1	1/1	1/1	1/1	1/1	1/1	1/1	1/1	1/1	1/1	1/1	1/1	1/1	1/1	1/1	1/1	1/1	0/0	1/1	1/1	1/1	1/1	1/1	1/1	1/1	1/1
0/0	0/0	0/0	0/0	0/0	0/0	./.	0/0	0/0	0/0	0/0	0/0	0/0	0/0	0/0	0/0	0/0	0/0	1/1	0/0	0/0	0/0	0/0	0/0	0/0	0/0	0/0
1/1	1/1	1/1	1/1	1/1	1/1	1/1	1/1	1/1	1/1	0/0	1/1	1/1	1/1	1/1	1/1	1/1	1/1	0/0	1/1	1/1	1/1	1/1	0/0	1/1	1/1	0/0
0/0	1/1	1/1	0/0	1/1	0/0	0/0	1/1	0/0	1/1	0/0	1/1	0/0	0/0	1/1	1/1	1/1	1/1	0/0	0/0	1/1	0/0	0/0	0/0	0/0	1/1	0/0
1/1	1/1	1/1	1/1	1/1	1/1	1/1	1/1	1/1	1/1	0/0	1/1	1/1	1/1	1/1	1/1	1/1	1/1	0/0	1/1	1/1	1/1	1/1	0/0	1/1	1/1	0/0
0/0	0/0	0/0	0/0	0/0	0/0	0/0	0/0	0/0	0/0	0/0	0/0	0/0	0/0	0/0	0/0	0/0	0/0	0/0	0/0	0/0	0/0	0/0	0/0	0/0	0/0	0/0
0/0	1/1	1/1	0/0	1/1	1/1	0/0	1/1	1/1	1/1	0/0	1/1	0/0	0/0	1/1	1/1	1/1	1/1	0/0	0/0	1/1	0/0	0/0	0/0	0/0	1/1	0/0

1/1	0/0	0/0	1/1	0/0	0/0	1/1	0/0	0/0	0/0	0/0	0/0	1/1	1/1	0/0	0/0	0/0	0/0	0/0	1/1	0/0	1/1	1/1	0/0	1/1	0/0	0/0
0/0	0/0	0/0	0/0	0/0	0/0	0/0	0/0	0/0	0/0	0/0	0/0	0/0	0/0	0/0	1/1	0/0	0/0	0/0	0/0	0/0	0/0	0/0	0/0	0/0	0/0	0/0
0/0	0/0	0/0	1/1	0/0	0/0	0/0	0/0	0/0	0/0	0/0	0/0	1/1	0/0	0/0	0/0	0/0	0/0	0/0	1/1	0/0	1/1	1/1	0/0	1/1	0/0	0/0
1/1	1/1	1/1	1/1	1/1	1/1	1/1	1/1	1/1	1/1	0/0	1/1	1/1	1/1	1/1	1/1	1/1	1/1	0/0	1/1	1/1	1/1	1/1	0/0	1/1	1/1	0/0
1/1	0/0	0/0	0/0	0/0	0/0	0/0	0/0	0/0	0/0	0/0	0/0	0/0	0/0	0/0	0/0	0/0	0/0	0/0	0/0	0/0	0/0	0/0	0/0	0/0	0/0	0/0
1/1	0/0	0/0	1/1	0/0	0/0	1/1	0/0	0/0	0/0	0/0	0/0	1/1	1/1	0/0	0/0	0/0	0/0	0/0	1/1	0/0	1/1	1/1	0/0	1/1	0/0	0/0
1/1	1/1	1/1	1/1	1/1	1/1	1/1	1/1	1/1	1/1	0/0	1/1	1/1	1/1	1/1	1/1	1/1	1/1	0/0	1/1	1/1	1/1	1/1	0/0	1/1	1/1	0/0
0/0	1/1	1/1	0/0	1/1	1/1	0/0	1/1	1/1	1/1	0/0	1/1	0/0	0/0	1/1	1/1	1/1	1/1	0/0	./.	1/1	0/0	0/0	0/0	0/0	1/1	0/0
1/1	0/0	0/0	1/1	0/0	0/0	1/1	0/0	0/0	0/0	0/0	0/0	0/1	1/1	0/0	0/0	0/0	0/0	0/0	1/1	0/0	1/1	1/1	0/0	1/1	0/0	0/0
0/0	0/0	0/0	0/0	0/0	1/1	0/0	0/0	1/1	0/0	0/0	0/0	0/0	0/0	0/0	0/0	0/0	0/0	0/0	0/0	0/0	0/0	0/0	0/0	0/0	0/0	0/0
1/1	0/0	0/0	1/1	0/0	0/0	1/1	0/0	0/0	0/0	0/0	0/0	1/1	1/1	0/0	0/0	0/0	0/0	0/0	1/1	0/0	1/1	1/1	0/0	1/1	0/0	0/0
0/0	0/0	0/0	0/0	0/0	0/0	0/0	0/0	0/0	0/0	0/0	0/0	0/0	0/0	0/0	0/0	0/0	0/0	0/0	0/0	0/0	0/0	0/0	0/0	0/0	0/0	0/0
0/0	0/0	0/0	0/0	0/0	0/0	0/0	0/0	0/0	0/0	0/0	0/0	0/0	0/0	0/0	0/0	0/0	0/0	0/0	0/0	0/0	0/0	0/0	0/0	0/0	0/0	0/0
0/0	0/0	0/0	0/0	0/0	0/0	0/0	0/0	0/0	0/0	0/0	0/0	0/0	0/0	0/0	0/0	0/0	0/0	0/0	0/0	0/0	0/0	0/0	0/0	0/0	0/0	0/0
1/1	1/1	1/1	1/1	1/1	1/1	1/1	1/1	1/1	1/1	1/1	1/1	1/1	1/1	1/1	1/1	1/1	1/1	0/0	1/1	1/1	1/1	1/1	1/1	1/1	1/1	1/1
1/1	1/1	1/1	1/1	1/1	1/1	1/1	1/1	1/1	1/1	1/1	1/1	1/1	1/1	1/1	1/1	1/1	1/1	0/0	1/1	1/1	1/1	1/1	1/1	1/1	1/1	1/1
1/1	1/1	1/1	1/1	1/1	1/1	1/1	1/1	1/1	1/1	0/0	1/1	1/1	1/1	1/1	1/1	1/1	1/1	0/0	1/1	1/1	1/1	1/1	0/0	1/1	1/1	0/0
./.	./.	./.	./.	0/0	./.	./.	./.	./.	./.	./.	./.	1/1	./.	0/0	0/0	./.	./.	0/0	./.	0/0	1/1	1/1	./.	1/1	./.	./.
1/1	3/3	3/3	1/1	3/3	1/1	1/1	3/3	1/1	3/3	1/1	3/3	4/4	1/1	3/3	3/3	3/3	3/3	0/2	1/1	3/3	4/4	4/4	1/1	4/4	3/3	1/1
0/0	1/1	1/1	0/0	1/1	0/0	0/0	0/0	0/0	0/0	0/0	0/0	0/0	0/0	0/0	0/0	0/0	0/0	0/0	0/0	0/0	0/0	0/0	0/0	0/0	0/0	0/0
0/0	0/0	0/0	0/0	0/0	0/0	0/0	0/0	0/0	0/0	0/0	0/0	0/0	0/0	0/0	0/0	0/0	0/0	0/0	0/0	0/0	0/0	0/0	0/0	0/0	0/0	0/0
0/0	0/0	0/0	0/0	0/0	0/0	0/0	0/0	0/0	0/0	0/0	0/0	0/0	0/0	0/0	0/0	0/0	0/0	0/0	0/0	0/0	0/0	0/0	0/0	0/0	0/0	0/0
0/0	0/0	0/0	0/0	0/0	0/0	0/0	0/0	0/0	0/0	0/0	0/0	0/0	0/0	0/0	0/0	0/0	0/0	0/0	0/0	0/0	0/0	0/0	0/0	0/0	0/0	0/0
0/0	0/0	0/0	0/0	0/0	0/0	0/0	0/0	0/0	0/0	0/0	./.	0/0	0/0	./.	./.	0/0	0/0	0/0	0/0	0/0	0/0	0/0	0/0	0/0	0/0	0/0
0/0	1/1	1/1	0/0	1/1	0/0	0/0	1/1	0/0	1/1	0/0	1/1	0/0	0/0	1/1	1/1	1/1	1/1	0/0	0/0	1/1	0/0	0/0	0/0	0/0	1/1	0/0

0/0	0/0	0/0	0/0	0/0	0/0	0/0	0/0	0/0	0/0	0/0	0/0	0/0	0/0	0/0	0/0	0/0	0/0	0/0	0/0	0/0	0/0	0/0	0/0	0/0	0/0	0/0
0/0	1/1	1/1	0/0	1/1	1/1	0/0	1/1	1/1	1/1	0/0	1/1	0/0	0/0	1/1	1/1	1/1	1/1	0/0	0/0	1/1	0/0	0/0	0/0	0/0	1/1	0/0
0/0	1/1	1/1	0/0	1/1	1/1	0/0	1/1	1/1	1/1	0/0	1/1	0/0	0/0	1/1	1/1	1/1	1/1	0/0	0/0	1/1	0/0	0/0	0/0	0/0	1/1	0/0
1/1	1/1	1/1	1/1	1/1	1/1	1/1	1/1	1/1	1/1	1/1	1/1	1/1	1/1	1/1	1/1	1/1	1/1	0/0	1/1	./.	1/1	1/1	1/1	1/1	1/1	1/1
1/1	1/1	1/1	1/1	1/1	1/1	1/1	1/1	1/1	1/1	0/0	1/1	1/1	1/1	1/1	1/1	1/1	1/1	0/0	1/1	1/1	1/1	1/1	0/0	1/1	1/1	0/0
0/0	0/0	0/0	0/0	0/0	0/0	0/0	0/0	0/0	0/0	0/0	0/0	0/0	0/0	0/0	0/0	0/0	1/1	0/0	0/0	0/0	0/0	0/0	0/0	0/0	1/1	0/0
1/1	1/1	1/1	1/1	1/1	1/1	1/1	1/1	1/1	1/1	0/0	1/1	1/1	1/1	1/1	1/1	1/1	1/1	0/0	1/1	1/1	1/1	1/1	0/0	1/1	1/1	0/0
0/0	1/1	1/1	0/0	1/1	1/1	0/0	1/1	1/1	1/1	0/0	1/1	0/0	0/0	1/1	1/1	1/1	1/1	0/0	0/0	1/1	0/0	0/0	0/0	0/0	1/1	0/0
0/0	0/0	0/0	0/0	0/0	0/0	0/0	0/0	0/0	0/0	0/0	0/0	0/0	0/0	0/0	0/0	0/0	0/0	0/0	0/0	0/0	0/0	0/0	0/0	0/0	0/0	0/0
0/0	0/0	0/0	0/0	0/0	1/1	0/0	0/0	1/1	0/0	0/0	0/0	0/0	0/0	0/0	0/0	0/0	0/0	0/0	0/0	0/0	0/0	0/0	0/0	0/0	0/0	0/0
0/0	1/1	1/1	0/0	1/1	1/1	0/0	1/1	1/1	1/1	0/0	1/1	0/0	0/0	1/1	1/1	1/1	1/1	0/0	0/0	1/1	0/0	0/0	0/0	0/0	1/1	0/0
0/0	0/0	0/0	0/0	0/0	0/0	0/0	0/0	0/0	0/0	0/0	0/0	0/0	0/0	0/0	0/0	0/0	0/0	0/0	0/0	0/0	0/0	0/0	0/0	0/0	0/0	0/0
1/1	0/0	0/0	1/1	0/0	0/0	1/1	0/0	0/0	0/0	0/0	0/0	1/1	1/1	0/0	0/0	0/0	0/0	0/0	1/1	0/0	1/1	0/1	0/0	1/1	0/0	0/0
0/0	1/1	1/1	0/0	1/1	1/1	0/0	1/1	1/1	1/1	0/0	1/1	0/0	0/0	1/1	1/1	1/1	1/1	0/0	0/0	1/1	0/0	0/0	0/0	0/0	1/1	0/0
0/0	0/0	0/0	0/0	0/0	0/0	0/0	0/0	0/0	0/0	0/0	0/0	0/0	0/0	0/0	0/0	0/0	0/0	0/0	0/0	0/0	0/0	0/0	0/0	0/0	0/0	0/0
1/1	1/1	1/1	1/1	1/1	1/1	1/1	1/1	1/1	1/1	0/0	1/1	1/1	1/1	1/1	1/1	1/1	1/1	0/0	1/1	1/1	1/1	0/1	0/0	1/1	1/1	0/0
1/1	0/0	0/0	1/1	0/0	0/0	1/1	0/0	0/0	0/0	0/0	0/0	1/1	1/1	0/0	0/0	0/0	0/0	0/0	1/1	0/0	1/1	0/1	0/0	1/1	0/0	0/0
1/1	0/0	0/0	0/0	0/0	0/0	1/1	0/0	0/0	0/0	0/0	0/0	0/0	1/1	0/0	0/0	0/0	0/0	0/0	0/0	0/0	0/0	0/0	0/0	0/0	0/0	0/0
0/0	0/0	./.	0/0	0/0	1/1	0/0	0/0	1/1	./.	0/0	0/0	0/0	0/0	0/0	0/0	0/0	0/0	0/0	0/0	0/0	0/0	0/0	0/0	0/0	0/0	0/0
0/0	1/1	1/1	0/0	1/1	1/1	0/0	1/1	1/1	1/1	0/0	1/1	0/0	0/0	1/1	1/1	1/1	1/1	0/0	0/0	1/1	0/0	0/0	0/0	0/0	1/1	0/0
0/0	1/1	1/1	0/0	1/1	1/1	0/0	1/1	1/1	1/1	0/0	1/1	0/0	0/0	1/1	1/1	1/1	1/1	0/0	0/0	1/1	0/0	0/0	0/0	0/0	1/1	0/0
0/0	0/0	0/0	0/0	0/0	0/0	0/0	0/0	0/0	0/0	0/0	0/0	0/0	0/0	0/0	0/0	0/0	0/0	0/0	0/0	0/0	0/0	0/0	0/0	0/0	0/0	0/0
1/1	1/1	1/1	1/1	1/1	1/1	1/1	1/1	1/1	1/1	0/0	1/1	1/1	1/1	1/1	1/1	1/1	1/1	0/0	1/1	1/1	1/1	1/1	0/0	1/1	1/1	0/0
0/0	0/0	0/0	0/0	0/0	1/1	0/0	0/0	1/1	0/0	0/0	0/0	0/0	0/0	0/0	0/0	0/0	0/0	0/0	0/0	0/0	0/0	0/0	0/0	0/0	0/0	0/0
0/0	0/0	0/0	0/0	0/0	0/0	0/0	0/0	0/0	0/0	0/0	0/0	0/0	0/0	0/0	0/0	0/0	0/0	0/0	0/0	0/0	0/0	0/0	0/0	0/0	0/0	0/0
0/0	1/1	1/1	0/0	1/1	1/1	0/0	1/1	1/1	./.	0/0	1/1	0/0	0/0	1/1	1/1	1/1	1/1	0/0	0/0	1/1	0/0	0/0	0/0	0/0	1/1	0/0
0/0	0/0	0/0	0/0	0/0	0/0	0/0	0/0	0/0	0/0	0/0	0/0	0/0	0/0	0/0	0/0	0/0	0/0	0/0	0/0	0/0	0/0	0/0	0/0	0/0	0/0	0/0
1/1	0/0	0/0	1/1	0/0	0/0	1/1	0/0	0/0	0/0	0/0	0/0	1/1	1/1	0/0	0/0	0/0	0/0	0/0	1/1	0/0	1/1	1/1	0/0	1/1	0/0	0/0
0/0	1/1	1/1	0/0	1/1	1/1	0/0	1/1	1/1	1/1	0/0	1/1	0/0	0/0	1/1	1/1	1/1	1/1	0/0	0/0	1/1	0/0	0/0	0/0	0/0	1/1	0/0
0/0	0/0	0/0	0/0	0/0	1/1	0/0	0/0	1/1	0/0	0/0	0/0	0/0	0/0	0/0	0/0	0/0	0/0	0/0	0/0	0/0	0/0	0/0	0/0	0/0	0/0	0/0
1/1	1/1	1/1	1/1	1/1	1/1	1/1	1/1	1/1	1/1	0/0	1/1	1/1	1/1	1/1	1/1	1/1	1/1	0/0	1/1	1/1	1/1	1/1	0/0	1/1	1/1	0/0
0/0	0/0	0/0	0/0	0/0	0/0	0/0	0/0	0/0	0/0	0/0	0/0	0/0	0/0	0/0	0/0	0/0	0/0	0/0	0/0	0/0	0/0	0/0	0/0	0/0	0/0	0/0
0/0	0/0	0/0	0/0	0/0	0/0	0/0	0/0	0/0	0/0	0/1	0/0	0/0	0/0	0/0	0/0	0/0	0/0	0/0	0/0	0/0	0/0	0/0	0/0	0/0	0/0	1/1

1/1	1/1	1/1	1/1	1/1	1/1	1/1	1/1	1/1	1/1	0/0	1/1	1/1	1/1	1/1	1/1	1/1	1/1	0/0	1/1	1/1	1/1	1/1	0/0	1/1	1/1	0/0
0/0	0/0	0/0	0/0	0/0	0/0	0/0	0/0	0/0	0/0	0/0	0/0	0/0	0/0	0/0	0/0	0/0	0/0	0/0	0/0	0/0	0/0	0/0	0/0	0/0	0/0	0/0
1/1	1/1	1/1	1/1	1/1	1/1	1/1	1/1	1/1	1/1	0/0	1/1	1/1	1/1	1/1	1/1	1/1	1/1	0/0	1/1	1/1	1/1	1/1	0/0	1/1	1/1	0/0
0/0	0/0	0/0	0/0	0/0	0/0	0/0	0/0	0/0	0/0	0/0	0/0	0/0	0/0	0/0	0/0	0/0	0/0	0/0	0/0	0/0	0/0	0/0	0/0	0/0	0/0	0/0
0/0	0/0	0/0	1/1	0/0	0/0	0/0	0/0	0/0	0/0	0/0	0/0	1/1	0/0	0/0	0/0	0/0	0/0	0/0	1/1	0/0	1/1	1/1	0/0	1/1	0/0	0/0
0/0	0/0	0/0	0/0	0/0	0/0	0/0	0/0	0/0	0/0	0/0	0/0	0/0	0/0	0/0	0/0	0/0	0/0	0/0	0/0	0/0	0/0	0/0	0/0	0/0	0/0	0/0
0/0	0/0	0/0	0/0	0/0	0/0	0/0	0/0	0/0	0/0	0/0	0/0	0/0	0/0	0/0	0/0	0/0	0/0	0/0	0/0	0/0	0/0	0/0	0/0	0/0	0/0	0/0
1/1	0/0	0/0	0/0	0/0	0/0	1/1	0/0	0/0	0/0	0/0	0/0	0/0	1/1	0/0	0/0	0/0	0/0	0/0	0/0	0/1	0/0	0/0	0/0	0/0	0/0	0/0
0/0	0/0	0/0	0/0	0/0	0/0	0/0	0/0	0/0	0/0	0/0	0/0	0/0	0/0	0/0	0/0	0/0	0/0	0/0	0/0	0/0	0/0	0/0	0/0	0/0	0/0	0/0
0/0	0/0	0/0	0/0	0/0	0/0	0/0	0/0	0/0	0/0	0/0	0/0	0/0	0/0	0/0	0/0	0/0	0/0	0/0	0/0	0/0	0/0	0/0	0/0	0/0	0/0	0/0
1/1	1/1	1/1	1/1	1/1	1/1	1/1	1/1	1/1	1/1	0/0	1/1	1/1	1/1	1/1	1/1	1/1	1/1	0/0	1/1	1/1	1/1	1/1	0/0	1/1	1/1	0/0
1/1	1/1	1/1	1/1	1/1	1/1	1/1	1/1	1/1	1/1	0/0	1/1	1/1	1/1	1/1	1/1	1/1	1/1	0/0	1/1	1/1	1/1	1/1	0/0	1/1	1/1	0/0
0/0	0/0	0/0	0/0	0/0	0/0	0/0	0/0	0/0	0/0	0/0	0/0	0/0	0/0	0/0	0/0	0/0	0/0	0/0	0/0	0/0	0/0	0/0	0/0	0/0	0/0	0/0
0/0	0/0	0/0	1/1	0/0	0/0	0/0	0/0	0/0	0/0	0/0	0/0	1/1	0/0	0/0	0/0	0/0	0/0	0/0	1/1	0/0	1/1	1/1	0/0	1/1	./.	0/0
0/0	1/1	1/1	0/0	1/1	1/1	0/0	1/1	1/1	1/1	0/0	1/1	0/0	0/0	1/1	1/1	1/1	1/1	0/0	0/0	0/1	0/0	0/0	0/0	0/0	1/1	0/0
1/1	1/1	1/1	1/1	1/1	2/2	1/1	1/1	1/2	1/1	2/2	0/1	1/1	1/1	1/1	1/1	1/1	1/1	0/0	1/1	1/1	1/1	1/1	2/2	1/1	2/2	1/1
1/1	1/1	1/1	1/1	1/1	1/1	1/1	1/1	1/1	1/1	0/0	1/1	1/1	1/1	1/1	1/1	1/1	1/1	0/0	1/1	1/1	1/1	1/1	0/0	1/1	1/1	0/0
0/0	1/1	1/1	0/0	1/1	0/0	0/0	1/1	0/0	1/1	0/0	1/1	0/0	1/1	1/1	1/1	1/1	1/1	0/0	0/0	1/1	0/0	0/0	0/0	0/0	1/1	0/0
0/0	1/1	1/1	0/0	1/1	0/0	0/0	1/1	0/0	1/1	0/0	1/1	0/0	1/1	1/1	1/1	1/1	1/1	0/0	0/0	1/1	0/0	0/0	0/0	0/0	1/1	0/0
0/0	0/0	0/0	1/1	0/0	0/0	0/0	0/0	0/0	0/0	0/0	0/0	1/1	0/0	0/0	0/0	0/0	0/0	0/0	1/1	0/0	1/1	1/1	0/0	1/1	0/0	0/0
0/0	0/0	0/0	0/0	0/0	0/0	0/0	0/0	0/0	0/0	0/0	0/0	0/0	0/0	0/0	0/0	0/0	0/0	0/0	0/0	0/0	0/0	0/0	0/0	0/0	0/0	0/0
0/0	0/0	0/0	0/0	0/0	0/0	0/0	0/0	0/0	0/0	0/0	0/0	0/0	0/0	0/0	0/0	0/0	0/0	0/0	0/0	0/0	0/0	0/0	0/0	0/0	0/0	0/0
0/0	0/0	0/0	0/0	0/0	0/0	0/0	0/0	0/0	0/0	0/0	0/0	0/0	0/0	0/0	0/0	0/0	0/0	0/0	0/0	0/0	0/0	0/0	0/0	0/0	0/0	0/0
0/0	0/0	0/0	0/0	0/0	0/0	0/0	0/0	0/0	0/0	0/0	0/0	0/0	0/0	0/0	0/0	0/0	0/0	0/0	0/0	0/0	0/0	0/0	0/0	0/0	0/0	0/0
0/0	0/0	0/1	0/0	0/0	0/0	./.	0/0	0/1	0/0	0/0	0/0	0/0	0/0	0/0	0/0	0/0	0/0	0/0	0/0	0/0	0/0	0/0	0/1	0/0	0/0	0/0
0/0	0/0	0/1	0/0	0/0	0/0	0/0	0/0	0/1	0/0	0/0	0/0	0/0	0/0	0/0	0/0	0/0	0/0	0/0	0/0	0/0	0/0	0/0	0/1	0/0	0/0	0/0

0/0	0/0	0/1	0/0	0/0	0/0	./.	0/0	0/1	0/0	0/0	0/0	0/0	0/0	0/0	0/0	0/0	0/0	0/0	0/0	0/0	0/0	0/0	0/1	0/0	0/0	0/0
0/0	0/0	0/1	0/0	0/0	0/0	0/0	0/0	0/1	0/0	0/0	0/0	0/0	0/0	0/0	0/0	0/0	0/0	0/0	0/0	0/0	0/0	0/0	0/1	0/0	0/0	0/0
0/0	0/0	0/1	0/0	0/0	0/0	0/0	0/0	0/1	0/0	0/0	0/0	0/0	0/0	0/0	0/0	0/0	0/0	0/0	0/0	0/0	0/0	0/0	0/1	0/0	0/0	0/0
0/0	0/0	0/1	0/0	0/0	0/0	0/0	0/0	0/1	0/0	0/0	0/0	0/0	0/0	0/0	0/0	0/0	0/0	0/0	0/0	0/0	0/0	0/0	0/1	0/0	0/0	0/0
0/0	0/0	0/1	0/0	0/0	0/0	0/0	0/0	0/1	0/0	0/0	0/0	0/0	0/0	0/0	0/0	0/0	0/0	0/0	0/0	0/0	0/0	0/0	0/1	0/0	0/0	0/0
0/0	0/0	0/1	0/0	0/0	0/0	0/0	0/0	0/1	0/0	0/0	0/0	0/0	0/0	0/0	0/0	0/0	0/0	0/0	0/0	0/0	0/0	0/0	0/1	0/0	0/0	0/0
0/0	0/0	0/0	0/0	0/0	0/0	0/0	0/0	0/0	0/0	0/0	0/0	0/0	0/0	0/0	0/0	0/0	0/0	0/0	0/0	0/0	0/0	0/0	0/0	0/0	0/0	0/0
0/0	0/0	0/0	0/0	0/0	0/0	0/0	0/0	0/0	0/0	0/0	0/0	0/0	0/0	0/0	0/0	0/0	0/0	0/0	0/0	0/0	0/0	0/0	0/0	0/0	0/0	0/0
0/0	0/0	0/0	0/0	0/0	0/0	0/0	0/0	0/0	0/0	0/0	0/0	0/0	0/0	0/0	0/0	0/0	0/0	0/0	0/0	0/0	0/0	0/0	0/0	0/0	0/0	0/0
0/0	0/0	0/0	0/0	0/0	0/0	0/0	0/0	0/0	0/0	0/0	0/0	0/0	0/0	0/0	0/0	0/0	0/0	0/0	0/0	0/0	0/0	0/0	0/0	0/0	0/0	0/0
1/1	1/1	1/1	1/1	1/1	1/1	1/1	1/1	1/1	1/1	1/1	1/1	1/1	1/1	1/1	1/1	1/1	1/1	0/0	1/1	1/1	1/1	1/1	1/1	1/1	1/1	1/1
0/0	0/0	0/0	0/0	0/0	0/0	0/0	0/0	0/0	0/0	0/0	0/0	0/0	0/0	0/0	0/0	0/0	0/0	0/0	0/0	0/0	0/0	0/0	0/0	0/0	0/0	0/0
0/0	0/0	0/0	0/0	0/0	1/1	0/0	0/0	1/1	0/0	0/0	0/0	0/0	0/0	0/0	0/0	0/0	0/0	0/0	0/0	0/0	0/0	0/0	0/0	0/0	0/0	0/0
1/1	0/0	0/0	0/0	0/0	0/0	1/1	0/0	0/0	0/0	0/0	0/0	0/0	0/0	0/0	0/0	0/0	0/0	0/0	0/0	0/0	0/0	0/0	0/0	0/0	0/0	0/0
0/0	0/0	0/0	0/0	0/0	0/0	0/0	0/0	0/0	0/0	0/0	0/0	0/0	0/0	0/0	0/0	0/0	0/0	0/0	0/0	0/0	0/0	0/0	0/0	0/0	0/0	0/0
0/0	0/0	0/0	0/0	0/0	0/0	0/0	0/0	0/0	0/0	0/0	0/0	0/0	0/0	0/0	0/0	0/0	0/0	0/0	0/0	0/0	0/0	0/0	0/0	0/0	0/0	0/0
1/1	1/1	1/1	1/1	1/1	1/1	1/1	1/1	1/1	1/1	0/0	1/1	1/1	1/1	1/1	1/1	1/1	1/1	0/0	1/1	1/1	1/1	1/1	0/0	1/1	1/1	0/0
0/0	./.	./.	0/0	./.	1/1	0/0	./.	1/1	0/0	0/0	0/0	0/0	0/0	./.	0/0	0/0	0/0	0/0	./.	./.	./.	0/0	0/0	0/0	./.	0/0
1/1	./.	./.	1/1	./.	1/1	1/1	./.	1/1	./.	0/0	./.	1/1	1/1	./.	0/0	./.	0/0	0/0	1/1	./.	1/1	1/1	0/0	1/1	./.	0/0
0/0	1/1	1/1	0/0	1/1	0/0	0/0	1/1	0/0	1/1	0/0	1/1	0/0	0/0	1/1	1/1	1/1	1/1	0/0	0/0	1/1	0/0	0/0	0/0	0/0	1/1	0/0
0/0	1/1	1/1	0/0	1/1	0/0	0/0	1/1	0/0	1/1	0/0	1/1	0/0	0/0	1/1	1/1	1/1	1/1	0/0	0/0	1/1	0/0	0/0	0/0	0/0	1/1	0/0
./.	0/0	0/0	0/0	0/0	./.	./.	0/0	0/0	0/0	0/0	0/0	0/0	./.	0/0	0/0	0/0	0/0	0/0	0/0	0/0	./.	./.	0/0	./.	0/0	0/0
./.	0/0	0/0	./.	0/0	1/1	0/1	0/0	0/1	0/0	0/0	0/0	./.	./.	0/0	0/0	0/0	0/0	0/0	0/0	0/0	./.	0/1	0/0	./.	0/0	./.
1/1	0/0	0/0	1/1	0/0	2/2	0/1	0/0	0/0	0/0	0/0	0/0	1/1	1/1	0/0	0/0	0/0	0/0	0/0	0/1	0/0	1/1	0/1	0/0	1/1	0/0	./.

2/2	0/0	0/0	2/2	0/0	2/2	0/2	0/0	0/0	0/0	0/0	0/0	2/2	2/2	0/0	0/0	0/0	0/0	0/0	0/1	0/0	2/2	0/2	0/0	2/2	0/0	./.
0/0	0/0	0/0	4/4	0/0	4/4	0/4	0/0	0/0	0/0	3/3	0/0	4/4	4/4	0/2	0/0	0/2	0/0	1/1	0/0	0/0	4/4	0/4	3/3	4/4	0/0	3/3
0/0	0/0	0/0	0/0	0/0	1/1	0/0	0/0	1/1	0/0	0/0	0/0	0/0	0/0	0/0	0/0	0/0	0/0	0/0	0/0	0/0	0/0	0/0	0/0	0/0	0/0	0/0
1/1	1/1	1/1	1/1	1/1	1/1	1/1	1/1	1/1	1/1	0/0	1/1	1/1	1/1	1/1	1/1	1/1	1/1	0/0	1/1	1/1	1/1	1/1	0/0	1/1	1/1	0/0
1/1	0/0	0/0	1/1	0/0	1/1	1/1	0/0	1/1	0/0	0/0	0/0	1/1	1/1	0/0	0/0	0/0	0/0	0/0	1/1	0/1	1/1	0/1	0/0	1/1	0/0	0/0
0/0	0/0	0/0	0/0	0/0	0/0	0/0	0/0	0/0	0/0	0/0	0/0	0/0	0/0	0/0	0/0	0/0	0/0	0/0	0/0	0/0	0/0	0/0	0/0	0/0	0/0	0/0
0/0	0/0	0/0	0/0	0/0	0/0	0/0	0/0	0/0	0/0	0/0	0/0	0/0	0/0	0/0	0/0	0/0	0/0	0/0	0/0	0/0	0/0	0/0	0/0	0/0	0/0	0/0
1/1	1/1	1/1	1/1	1/1	1/1	1/1	1/1	1/1	1/1	0/0	1/1	1/1	1/1	1/1	1/1	1/1	1/1	0/0	1/1	1/1	1/1	0/1	0/0	1/1	1/1	0/0
./.	0/0	0/0	0/0	0/0	./.	./.	0/0	./.	0/0	0/0	0/0	./.	0/0	0/0	0/0	0/0	0/0	0/0	0/0	0/0	./.	0/0	0/0	./.	0/0	0/0
1/1	0/0	0/0	1/1	0/0	2/2	1/1	0/0	2/2	0/0	0/0	0/0	1/1	1/1	0/0	0/0	0/0	0/0	0/0	1/1	0/1	1/1	1/1	0/0	1/1	0/0	0/0
1/1	0/0	0/0	1/1	0/0	1/1	1/1	0/0	1/1	0/0	0/0	0/0	1/1	1/1	0/0	0/0	0/0	0/0	0/0	1/1	0/1	1/1	1/1	0/0	1/1	0/0	0/0
1/1	0/0	0/0	1/1	0/0	0/0	1/1	0/0	0/0	0/0	0/0	0/0	1/1	1/1	0/0	0/0	0/0	0/0	0/0	1/1	0/1	1/1	1/1	0/0	1/1	0/0	0/0
0/0	1/1	1/1	0/0	1/1	0/0	0/0	1/1	0/0	1/1	0/0	1/1	0/0	0/0	1/1	1/1	1/1	1/1	0/0	0/0	1/1	0/0	0/0	0/0	0/0	1/1	0/0
1/1	0/0	0/0	1/1	0/0	1/1	1/1	0/0	1/1	0/0	0/0	0/0	1/1	1/1	0/0	0/0	0/0	0/0	0/0	1/1	0/0	1/1	1/1	0/0	1/1	0/0	0/0
1/1	1/1	1/1	1/1	1/1	1/1	1/1	1/1	1/1	1/1	0/0	1/1	1/1	1/1	1/1	1/1	1/1	1/1	0/0	1/1	1/1	1/1	1/1	0/0	1/1	1/1	0/0
0/0	0/0	0/0	0/0	0/0	0/0	0/0	0/0	0/0	0/0	0/0	0/0	0/0	0/0	0/0	0/0	0/0	0/0	0/0	0/0	0/0	0/0	0/0	0/0	0/0	0/0	0/0
0/0	0/0	0/0	0/0	0/0	0/0	1/1	0/0	0/0	0/0	0/0	0/0	0/0	0/0	0/0	0/0	0/0	0/0	0/0	0/0	0/0	0/0	0/0	0/0	0/0	0/0	0/0
0/0	1/1	1/1	0/0	1/1	0/0	0/0	1/1	0/0	1/1	0/0	1/1	0/0	0/0	1/1	1/1	1/1	1/1	0/0	0/0	0/1	0/0	0/0	0/0	0/0	1/1	0/0
0/0	0/0	0/0	0/0	0/0	0/0	0/0	0/0	0/0	0/0	0/0	0/0	0/0	0/0	0/0	0/0	0/0	0/0	0/0	0/0	0/0	0/0	0/0	0/0	0/0	0/0	0/0
0/0	1/1	1/1	0/0	1/1	0/0	0/0	1/1	0/0	1/1	0/0	1/1	0/0	0/0	0/1	1/1	1/1	1/1	0/0	0/0	0/1	0/0	0/0	0/0	0/0	1/1	0/0
1/1	0/0	0/0	0/0	0/0	0/0	1/1	0/0	0/0	0/0	0/0	0/0	0/0	1/1	0/0	0/0	0/0	0/0	0/0	0/0	0/1	0/0	0/0	0/0	0/0	0/0	0/0
0/0	1/1	1/1	0/0	1/1	0/0	0/0	1/1	0/0	1/1	0/0	1/1	0/0	0/0	1/1	1/1	1/1	1/1	0/0	0/0	0/1	0/0	0/0	0/0	0/0	1/1	0/0
0/0	1/1	1/1	0/0	1/1	0/0	0/0	1/1	0/0	1/1	0/0	1/1	0/0	0/0	1/1	1/1	1/1	1/1	0/0	0/0	0/1	0/0	0/0	0/0	0/0	1/1	0/0
0/0	0/0	0/0	0/0	0/0	1/1	0/0	0/0	1/1	0/0	0/0	0/0	0/0	0/0	0/0	0/0	0/0	0/0	0/0	0/0	0/0	0/0	0/0	0/0	0/0	0/0	0/0
0/0	0/0	0/0	0/0	0/0	1/1	0/0	0/0	1/1	0/0	0/0	0/0	0/0	0/0	0/0	0/0	0/0	0/0	0/0	0/0	0/0	0/0	0/0	0/0	0/0	0/0	0/0
1/1	0/0	0/0	1/1	0/0	1/1	1/1	0/0	1/1	0/0	0/0	0/0	1/1	1/1	0/0	0/0	0/0	0/0	0/0	1/1	0/1	1/1	1/1	0/0	1/1	0/0	0/0
0/0	0/0	0/0	0/0	0/0	0/0	0/0	0/0	0/0	0/0	0/0	0/0	0/0	0/0	0/0	0/0	0/0	0/0	0/0	0/0	0/0	0/0	0/0	0/0	0/0	0/0	0/0
1/1	1/1	1/1	1/1	1/1	1/1	1/1	1/1	1/1	1/1	0/0	1/1	1/1	1/1	1/1	1/1	1/1	1/1	0/0	1/1	1/1	1/1	1/1	0/0	1/1	1/1	0/0
0/0	0/0	0/0	0/0	0/0	0/0	0/0	0/0	0/0	0/0	0/0	0/0	0/0	0/0	0/0	0/0	0/0	1/1	0/0	0/0	0/0	0/0	0/0	0/0	0/0	0/0	0/0
1/1	1/1	1/1	1/1	1/1	1/1	1/1	1/1	1/1	1/1	0/0	1/1	1/1	1/1	1/1	1/1	1/1	1/1	0/0	1/1	1/1	1/1	1/1	0/0	1/1	1/1	0/0

0/0	0/0	0/0	0/0	0/0	1/1	0/0	0/0	1/1	0/0	0/0	0/0	0/0	0/0	0/0	0/0	0/0	0/0	0/0	0/0	0/0	0/0	0/0	0/0	0/0	0/0	0/0
0/0	0/0	0/0	0/0	0/0	0/0	0/0	0/0	0/0	0/0	0/0	0/0	0/0	0/0	0/0	0/0	0/0	0/0	0/0	0/0	0/0	0/0	0/0	0/0	0/0	0/0	0/0
0/0	0/0	0/0	0/0	0/0	0/0	0/0	0/0	0/0	0/0	0/0	0/0	0/0	0/0	0/0	0/0	0/0	0/0	0/0	0/0	0/0	0/0	0/0	0/0	0/0	0/0	0/0
0/0	0/0	0/0	1/1	0/0	0/0	0/0	0/0	0/0	0/0	0/0	0/0	1/1	0/0	0/0	0/1	0/0	0/0	0/0	1/1	0/0	1/1	1/1	0/0	1/1	0/0	0/0
1/1	0/0	0/0	1/1	0/0	1/1	1/1	0/0	1/1	0/0	0/0	0/0	1/1	1/1	0/0	0/1	0/0	0/0	0/0	1/1	0/0	1/1	1/1	0/0	1/1	0/0	0/0
1/1	1/1	1/1	1/1	1/1	1/1	1/1	1/1	1/1	1/1	0/0	1/1	1/1	1/1	1/1	1/1	1/1	1/1	0/0	1/1	1/1	1/1	1/1	0/0	1/1	1/1	0/0
1/1	0/0	0/0	1/1	0/0	1/1	1/1	0/0	1/1	0/0	0/0	0/0	1/1	1/1	0/0	0/1	0/0	0/0	0/0	1/1	0/0	1/1	1/1	0/0	1/1	0/0	0/0
1/1	0/0	0/0	0/0	0/0	0/0	1/1	0/0	0/0	0/0	0/0	0/0	0/0	1/1	0/0	0/0	0/0	0/0	0/0	0/0	0/0	0/0	0/0	0/0	0/0	0/0	0/0
0/0	0/0	0/0	0/0	0/0	0/0	0/0	0/0	0/0	0/0	0/0	0/0	0/0	0/0	0/0	0/0	0/0	0/0	0/0	0/0	0/0	0/0	0/0	0/0	0/0	0/0	0/0
0/0	0/0	0/0	0/0	0/0	0/0	0/0	0/0	0/0	0/0	0/0	0/0	0/0	0/0	0/0	0/0	0/0	0/0	0/0	0/0	0/0	0/0	0/0	0/0	0/0	0/0	0/0
0/0	0/0	0/0	0/0	0/0	0/0	0/0	0/0	0/0	0/0	0/0	0/0	0/0	0/0	0/0	0/0	0/0	0/0	0/0	0/0	0/0	0/0	0/0	0/0	0/0	0/0	0/0
0/0	1/1	1/1	0/0	1/1	0/0	0/0	1/1	0/0	1/1	0/0	1/1	0/0	0/0	1/1	1/1	1/1	1/1	0/0	0/0	1/1	0/0	0/0	0/0	0/0	1/1	0/0
0/0	1/1	1/1	1/1	1/1	2/2	0/0	1/1	2/2	1/1	0/0	1/1	1/1	0/0	1/1	1/1	0/1	1/1	0/0	0/1	1/1	1/1	0/1	0/0	1/1	1/1	0/0
1/1	1/1	1/1	1/1	1/1	1/1	1/1	1/1	1/1	1/1	0/0	1/1	1/1	1/1	1/1	1/1	1/1	1/1	0/0	1/1	1/1	1/1	1/1	0/0	1/1	1/1	0/0
1/1	1/1	1/1	1/1	1/1	1/1	1/1	1/1	1/1	1/1	1/1	1/1	1/1	1/1	1/1	1/1	1/1	1/1	1/1	1/1	1/1	1/1	1/1	1/1	1/1	1/1	1/1
0/0	0/0	0/0	0/0	0/0	0/0	0/0	0/0	0/0	0/0	0/0	0/0	0/0	0/0	0/0	0/0	0/0	0/0	0/0	0/0	0/0	0/0	0/0	0/0	0/0	0/0	0/0
0/0	0/0	0/0	1/1	0/0	0/0	0/0	0/0	0/0	0/0	0/0	0/0	1/1	0/0	0/0	0/0	0/0	0/0	0/0	1/1	0/0	1/1	1/1	0/0	1/1	0/0	0/0
1/1	0/0	0/0	1/1	0/0	1/1	1/1	0/0	1/1	0/0	0/0	0/0	1/1	1/1	0/0	0/0	0/0	0/0	0/0	1/1	0/0	1/1	1/1	0/0	1/1	0/0	0/0
1/1	1/1	1/1	1/1	1/1	1/1	1/1	1/1	1/1	1/1	0/0	1/1	1/1	1/1	1/1	1/1	1/1	1/1	0/0	1/1	1/1	1/1	1/1	0/0	1/1	1/1	0/0
0/0	0/0	0/0	0/0	0/0	0/0	0/0	0/0	0/0	0/0	0/0	0/0	0/0	0/0	1/1	0/0	0/1	0/0	0/0	0/0	0/0	0/0	0/0	0/0	0/0	0/0	0/0
0/0	0/0	0/0	0/0	0/0	0/0	0/0	0/0	0/0	0/0	0/0	0/0	0/0	0/0	0/0	0/0	0/0	0/0	0/0	0/0	0/0	0/0	0/0	0/0	0/0	0/0	0/0
0/0	0/0	0/0	0/0	0/0	1/1	0/0	0/0	1/1	0/0	0/0	0/0	0/0	0/0	0/0	0/0	0/0	0/0	0/0	0/0	0/0	0/0	0/0	0/0	0/0	0/0	0/0
1/1	1/1	1/1	1/1	1/1	1/1	1/1	1/1	1/1	1/1	0/0	1/1	1/1	1/1	1/1	1/1	1/1	1/1	0/0	1/1	1/1	1/1	1/1	0/0	1/1	1/1	0/0
0/0	0/0	0/0	0/0	0/0	0/0	0/0	0/0	0/0	0/0	0/0	0/0	0/0	0/0	0/0	0/0	0/0	0/0	0/0	0/0	0/0	0/0	0/0	0/0	0/0	0/0	0/0
0/0	0/0	0/0	0/0	0/0	1/1	0/0	0/0	1/1	0/0	0/0	0/0	0/0	0/0	0/0	0/0	0/0	0/0	0/0	0/0	0/0	0/0	0/0	0/0	0/0	0/0	0/0
0/0	0/0	0/0	0/0	0/0	0/0	0/0	0/0	0/0	0/0	0/0	0/0	0/0	0/0	0/0	0/0	0/0	0/0	0/0	0/0	0/0	0/0	0/0	0/0	0/0	0/0	0/0
0/0	0/0	0/0	0/0	0/0	0/0	0/0	0/0	0/0	0/0	0/0	0/0	0/0	0/0	0/0	0/0	0/0	0/0	0/0	0/0	0/0	0/0	0/0	0/0	0/0	0/0	0/0
0/0	0/0	0/0	0/0	0/0	0/0	0/0	0/0	0/0	0/0	0/0	0/0	0/0	0/0	0/0	0/0	0/0	0/0	0/0	0/0	0/0	0/0	0/0	0/0	0/0	0/0	0/0
0/0	0/0	0/0	0/0	0/0	0/0	0/0	0/0	0/0	0/0	0/0	0/0	0/0	0/0	0/0	0/0	0/0	0/0	0/0	0/0	0/0	0/0	0/0	0/0	0/0	0/0	0/0
0/0	0/0	0/0	0/0	0/0	0/0	0/0	0/0	0/0	0/0	0/0	0/0	0/0	0/0	0/0	0/0	0/0	0/0	0/0	0/0	0/0	0/0	0/0	0/0	0/0	0/0	0/0
0/0	0/0	0/0	0/0	0/0	0/0	0/0	0/0	0/0	0/0	0/0	0/0	0/0	0/0	0/0	0/0	0/0	0/0	0/0	0/0	0/0	0/0	0/0	0/0	0/0	0/0	0/0
0/0	1/1	1/1	0/0	1/1	0/0	0/0	1/1	0/0	1/1	0/0	1/1	0/0	0/0	1/1	0/0	1/1	1/1	0/0	0/0	1/1	0/0	0/0	0/0	0/0	1/1	0/0
0/0	0/0	0/0	0/0	0/0	0/0	0/0	0/0	0/0	0/0	0/0	0/0	0/0	0/0	0/0	0/0	0/0	0/0	0/0	0/0	0/0	0/0	0/0	0/0	0/0	0/0	0/0
0/0	0/0	0/0	0/0	0/0	0/0	0/0	0/0	0/0	0/0	0/0	0/0	0/0	0/0	0/0	0/0	0/0	0/0	0/0	0/0	0/0	0/0	0/0	0/0	0/0	0/0	0/0
0/0	0/0	0/0	1/1	0/0	0/0	0/0	0/0	0/0	0/0	0/0	0/0	0/0	0/0	0/0	0/0	0/0	0/0	0/0	1/1	0/0	0/0	0/0	0/0	0/0	0/0	0/0

1/1	0/0	0/0	0/0	0/0	0/0	1/1	0/0	0/0	0/0	0/0	0/0	0/0	1/1	0/0	0/0	0/0	0/0	0/0	0/0	0/0	0/0	0/0	0/0	0/0	0/0	0/0
4/4	2/2	2/2	2/2	2/2	4/4	1/4	1/1	4/4	1/1	1/2	1/1	2/2	4/4	1/1	1/1	1/1	1/2	5/5	2/3	1/1	2/3	2/3	1/2	2/2	1/2	3/5
./.	./.	./.	./.	./.	./.	./.	./.	./.	./.	./.	./.	./.	./.	./.	./.	./.	./.	./.	./.	./.	./.	./.	./.	./.	./.	./.
0/0	0/0	0/0	0/0	0/0	0/0	0/0	0/0	0/0	0/0	0/0	0/0	0/0	0/0	0/0	0/0	0/0	0/0	0/0	0/0	0/0	0/0	0/0	0/0	0/0	0/0	0/0
0/0	0/0	0/0	0/0	0/0	0/0	0/0	0/0	0/0	0/0	0/0	0/0	0/0	0/0	0/0	0/0	0/0	0/0	0/0	0/0	0/0	0/0	0/0	0/0	0/0	0/0	0/0
0/0	0/0	0/0	0/0	0/0	1/1	0/0	0/0	1/1	0/0	0/0	0/0	0/0	0/0	0/0	0/0	0/0	0/0	0/0	0/0	0/0	0/0	0/0	0/0	0/0	0/0	0/0
1/1	0/0	0/0	0/0	0/0	0/0	1/1	0/0	0/0	0/0	0/0	0/0	0/0	1/1	0/0	0/0	0/0	0/0	0/0	0/0	0/0	0/0	0/0	0/0	0/0	0/0	0/0
1/1	0/0	0/0	./.	0/0	0/0	1/1	0/0	0/0	0/0	0/0	0/0	0/0	1/1	0/0	0/0	0/0	0/0	0/0	0/0	0/0	0/0	0/0	0/0	0/0	./.	0/0
0/0	./.	0/0	./.	0/0	1/1	0/0	0/0	1/1	0/0	0/0	0/0	0/0	./.	0/0	0/0	./.	0/0	0/0	0/0	0/0	./.	0/0	0/0	0/0	./.	./.
1/1	./.	./.	1/1	0/0	1/1	1/1	./.	1/1	0/0	0/0	0/0	1/1	1/1	1/1	0/0	0/1	0/0	./.	1/1	./.	1/1	1/1	./.	1/1	./.	./.
./.	2/2	2/2	./.	2/2	./.	./.	2/2	./.	2/2	1/1	2/2	./.	./.	./.	2/2	0/2	2/2	1/1	./.	2/2	./.	./.	1/1	./.	2/2	1/1
./.	./.	./.	0/0	./.	./.	0/0	./.	./.	./.	2/2	./.	0/0	0/0	0/0	./.	./.	./.	2/2	./.	./.	0/0	./.	2/2	0/0	./.	2/2
0/0	0/0	0/0	0/0	0/0	1/1	0/0	0/0	1/1	0/0	0/0	0/0	0/0	0/0	0/0	0/0	0/0	0/0	0/0	0/0	0/0	0/0	0/0	0/0	0/0	0/0	0/0
0/0	0/0	0/0	0/0	0/0	0/0	0/0	0/0	0/0	0/0	0/0	0/0	0/0	0/0	0/0	0/0	0/0	0/0	0/0	0/0	0/0	0/0	0/0	0/0	0/0	0/0	0/0
0/0	0/0	0/0	0/0	0/0	0/0	0/0	0/0	0/0	0/0	0/0	0/0	0/0	0/0	0/0	0/0	0/0	0/0	0/0	0/0	0/0	0/0	0/0	0/0	0/0	0/0	0/0
1/1	1/1	1/1	1/1	1/1	1/1	1/1	1/1	1/1	1/1	0/0	1/1	1/1	1/1	1/1	1/1	1/1	1/1	0/0	1/1	1/1	1/1	1/1	0/0	1/1	1/1	0/0
0/0	0/0	0/0	0/0	0/0	0/0	0/0	0/0	0/0	0/0	0/0	0/0	0/0	0/0	0/0	0/0	0/0	0/0	0/0	0/0	0/0	0/0	0/0	0/0	0/0	0/0	0/0
0/0	0/0	0/0	1/1	0/0	0/0	0/0	0/0	0/0	0/0	0/0	0/0	1/1	0/0	0/0	0/0	0/0	0/0	0/0	1/1	0/0	1/1	1/1	0/0	1/1	0/0	0/0
0/0	0/0	0/0	0/0	0/0	0/0	0/0	0/0	0/0	0/0	0/0	0/0	0/0	0/0	0/0	0/0	0/0	0/0	0/0	0/0	0/0	0/0	0/0	0/0	0/0	0/0	0/0
0/0	0/0	0/0	0/0	0/0	0/0	0/0	0/0	0/0	0/0	0/0	0/0	0/0	0/0	0/0	1/1	0/0	0/0	0/0	0/0	0/0	0/0	0/0	0/0	0/0	0/0	0/0
0/0	0/0	0/0	0/0	0/0	0/0	0/0	0/0	1/1	0/0	0/0	0/0	0/0	0/0	0/0	0/0	0/0	0/0	0/0	0/0	0/0	0/0	0/0	0/0	0/0	0/0	0/0

0/0	0/0	0/0	0/0	0/0	0/0	0/0	0/0	0/0	0/0	0/0	0/0	0/0	0/0	0/0	0/0	0/0	0/0	0/0	0/0	0/0	0/0	0/0	0/0	0/0	0/0	0/0
0/0	0/0	0/0	0/0	0/0	0/0	0/0	0/0	0/0	0/0	0/0	0/0	0/0	0/0	0/0	0/0	0/0	0/0	0/0	0/0	0/0	0/0	0/0	0/0	0/0	0/0	0/0
1/1	2/2	2/2	1/1	2/2	1/1	1/1	2/2	1/1	1/1	0/0	2/2	1/1	1/1	2/2	1/1	2/2	2/2	0/0	1/1	2/2	1/1	1/1	0/0	1/1	2/2	0/0
0/0	0/0	0/0	0/0	0/0	0/0	0/0	0/0	0/0	0/0	0/0	0/0	0/0	0/0	0/0	0/0	0/0	0/0	0/0	0/0	0/0	0/0	0/0	0/0	0/0	0/0	0/0
0/0	2/2	2/2	1/1	2/2	0/0	0/0	2/2	0/0	2/2	0/0	2/2	1/1	0/0	2/2	1/1	2/2	2/2	0/0	1/1	2/2	1/1	1/1	0/0	1/1	2/2	0/0
0/0	0/0	0/0	0/0	0/0	0/0	0/0	0/0	0/0	0/0	0/0	0/0	0/0	0/0	0/0	0/0	0/0	0/0	0/0	0/0	0/0	0/0	0/0	0/0	0/0	0/0	0/0
1/1	0/0	0/0	0/0	0/0	0/0	1/1	0/0	0/0	0/0	0/0	0/0	0/0	1/1	0/0	0/0	0/0	0/0	0/0	0/0	0/0	0/0	0/0	0/0	0/0	0/0	0/0
1/1	1/1	1/1	1/1	1/1	1/1	1/1	1/1	1/1	1/1	0/0	1/1	1/1	1/1	1/1	1/1	1/1	1/1	0/0	1/1	1/1	1/1	1/1	0/0	1/1	1/1	0/0
1/1	1/1	1/1	1/1	1/1	1/1	1/1	1/1	1/1	1/1	0/0	1/1	1/1	1/1	1/1	1/1	1/1	1/1	0/0	1/1	1/1	1/1	1/1	0/0	1/1	1/1	0/0
0/0	0/0	0/0	0/0	0/0	0/0	0/0	0/0	0/0	0/0	0/0	0/0	0/0	./.	0/0	0/0	0/0	0/0	0/0	0/0	0/0	0/0	0/0	0/0	0/0	0/0	0/0
2/2	2/2	2/2	1/1	2/2	2/2	2/2	2/2	2/2	2/2	0/0	2/2	1/1	2/2	2/2	2/2	2/2	2/2	0/0	1/1	2/2	1/1	1/1	0/0	1/1	2/2	0/0
0/0	0/0	0/0	0/0	0/0	0/0	0/0	0/0	0/0	0/0	0/0	0/0	0/0	0/0	0/0	0/0	0/0	0/0	0/0	0/0	0/0	0/0	0/0	0/0	0/0	0/0	0/0
0/0	0/0	0/0	0/0	0/0	0/0	0/0	0/0	0/0	0/0	0/0	0/0	0/0	0/0	0/0	0/0	0/0	0/0	0/0	0/0	0/0	0/0	0/0	0/0	0/0	0/0	0/0
0/0	0/0	0/0	0/0	0/0	0/0	0/0	0/0	0/0	0/0	0/0	0/0	0/0	0/0	0/0	0/0	0/0	0/0	0/0	0/0	0/0	0/0	0/0	0/0	0/0	0/0	0/0
1/1	0/0	0/0	0/0	0/0	1/1	1/1	0/0	1/1	0/0	0/0	0/0	0/0	1/1	0/0	0/0	0/0	0/0	0/0	0/0	0/0	0/0	0/0	0/0	0/0	0/0	0/0
0/0	0/0	0/0	0/0	0/0	0/0	0/0	0/0	0/0	0/0	0/0	0/0	0/0	0/0	0/0	0/0	0/0	0/0	0/0	0/0	0/0	0/0	0/0	0/0	0/0	0/0	0/0
1/1	0/0	0/0	0/0	0/0	0/0	1/1	0/0	0/0	0/0	0/0	0/0	0/0	1/1	0/0	0/0	0/0	0/0	0/0	0/0	0/0	0/0	0/0	0/0	0/0	0/0	0/0
1/1	1/1	1/1	1/1	1/1	1/1	1/1	1/1	1/1	1/1	0/0	1/1	1/1	1/1	1/1	1/1	1/1	1/1	0/0	1/1	1/1	1/1	1/1	0/0	1/1	1/1	0/0

811	812	813	821	822	829	82	847	84	851	859	86	880	885	888	893	900	905	910	916	929	931	942	953	954	967	973
0/0	0/0	0/0	0/0	0/0	0/0	0/0	0/0	0/0	0/0	0/0	0/0	0/0	0/0	0/0	0/0	0/0	0/0	0/0	0/0	0/0	0/0	0/0	0/0	0/0	0/0	0/0
0/0	0/0	1/1	1/1	1/1	0/0	0/0	0/1	0/0	0/0	0/0	1/1	0/0	0/0	0/0	0/0	0/0	1/1	1/1	0/0	0/0	0/0	0/0	0/0	0/0	0/0	0/1
0/0	0/0	0/0	0/0	0/0	0/0	0/0	0/0	0/0	0/0	0/0	0/0	0/0	0/0	0/0	0/0	0/0	0/0	0/0	0/0	0/0	0/0	0/0	0/0	0/0	0/0	0/0
0/0	0/0	0/0	0/0	0/0	0/0	0/0	0/0	0/0	0/0	0/0	0/0	0/0	0/0	0/0	0/0	0/0	0/0	0/0	0/0	0/0	0/0	0/0	0/0	0/0	1/1	0/0
0/0	0/0	0/0	0/0	0/0	0/0	0/0	0/0	1/1	1/1	0/0	0/0	0/0	0/0	0/0	0/0	0/0	0/0	0/0	0/0	0/0	1/1	1/1	1/1	1/1	1/1	0/1
0/0	0/0	0/0	0/0	0/0	0/0	0/0	0/0	1/1	1/1	0/0	0/0	0/0	0/0	0/0	0/0	0/0	0/0	0/0	0/0	0/0	1/1	1/1	1/1	1/1	1/1	0/1
0/0	0/0	0/0	0/0	0/0	0/0	0/0	0/0	0/0	0/0	0/0	0/0	0/0	0/0	0/0	0/0	0/0	0/0	0/0	0/0	0/0	0/0	0/0	0/0	0/0	0/0	0/0
1/1	0/0	1/1	1/1	1/1	0/0	0/0	1/1	0/0	0/0	0/0	1/1	0/0	0/0	0/0	0/0	1/1	1/1	1/1	0/0	0/0	0/0	0/0	0/0	0/0	0/0	0/1
0/0	0/0	0/0	0/0	0/0	0/0	0/0	0/0	0/0	0/0	0/0	0/0	0/0	0/0	0/0	0/0	0/0	0/0	0/0	0/0	0/0	0/0	0/0	0/0	0/0	0/0	0/0
0/0	0/0	0/0	0/0	0/0	0/0	0/0	0/0	0/0	0/0	0/0	0/0	0/0	0/1	0/0	0/0	0/0	0/0	0/0	0/0	0/0	0/0	0/0	0/0	0/0	0/0	0/0
0/0	0/0	0/0	0/0	0/0	0/1	0/0	0/0	0/0	0/0	0/0	0/0	0/0	0/0	0/0	0/0	0/0	0/0	0/0	0/0	0/0	0/0	0/0	0/0	0/0	0/0	0/0
0/0	0/0	0/0	0/0	0/0	0/0	0/0	0/0	0/0	0/0	0/0	0/0	0/0	0/0	0/0	0/0	0/0	0/0	0/0	0/0	0/0	0/0	0/0	0/0	0/0	0/0	0/0
0/0	0/0	0/0	0/0	0/0	0/0	0/0	0/0	1/1	1/1	0/1	0/0	0/0	0/0	0/0	0/0	0/0	0/0	0/0	0/0	0/0	1/1	1/1	1/1	1/1	1/1	0/1
0/0	0/0	0/0	0/0	0/0	0/0	0/0	0/0	0/0	0/0	0/0	0/0	0/0	0/0	0/0	0/0	0/0	0/1	0/0	0/0	0/0	0/0	0/0	0/0	0/0	0/0	0/0
1/1	0/0	1/1	1/1	1/1	0/0	0/0	1/1	0/0	0/0	0/0	1/1	0/0	0/0	./.	0/0	1/1	0/1	1/1	0/0	0/0	./.	0/0	0/0	0/0	0/0	0/1
0/0	0/0	0/0	0/0	0/0	0/0	0/0	0/0	0/0	0/0	0/1	./.	0/0	0/0	./.	0/0	0/0	0/0	0/0	0/0	0/0	./.	0/0	0/0	0/0	0/0	./.

0/0	0/1	3/3	6/6	3/6	0/4	0/0	0/3	2/3	3/3	0/5	./.	3/3	0/0	1/1	1/1	0/4	1/5	3/3	0/1	3/3	2/2	6/6	1/1	./.	2/3	0/3
0/0	0/0	0/0	0/0	0/0	0/0	0/0	0/0	0/0	0/0	0/0	0/0	0/0	0/0	0/0	0/0	0/0	0/0	0/0	0/0	0/0	0/0	0/0	0/0	0/0	0/0	0/0
0/0	0/0	0/0	0/0	0/0	0/0	./.	0/0	0/0	0/0	./.	0/0	0/0	0/0	0/0	0/0	0/0	0/0	0/0	0/0	0/0	0/0	0/0	0/0	./.	0/0	0/0
0/0	0/0	0/0	0/0	0/0	0/0	0/0	0/0	1/1	1/1	0/1	0/0	0/0	0/0	0/0	0/0	0/0	0/0	0/0	0/0	0/0	1/1	1/1	1/1	1/1	0/0	0/1
1/1	0/0	1/1	1/1	1/1	0/0	0/0	1/1	0/0	0/0	0/0	1/1	0/0	0/0	0/0	0/0	1/1	1/1	1/1	0/0	0/0	0/0	0/0	0/0	0/0	0/0	0/1
0/0	0/0	0/0	0/0	0/0	0/0	0/0	0/0	0/0	0/0	0/0	0/0	0/0	0/0	0/0	0/0	0/0	0/0	0/0	0/0	0/0	0/0	0/0	0/0	0/0	0/1	0/0
1/1	0/0	1/1	1/1	1/1	0/0	0/0	1/1	0/0	0/0	0/0	1/1	0/0	0/0	0/0	0/0	1/1	1/1	1/1	0/0	0/0	0/0	0/0	0/0	0/0	0/0	0/1
0/0	0/0	0/0	0/0	0/0	0/0	0/0	0/0	1/1	1/1	0/0	0/0	0/0	./.	0/0	0/0	0/0	0/0	0/0	0/0	0/0	1/1	1/1	1/1	1/1	0/1	0/0
0/0	1/1	0/0	0/0	0/0	0/0	1/1	0/0	0/0	0/0	1/1	0/0	1/1	1/1	1/1	1/1	0/0	0/0	0/0	0/0	1/1	0/0	0/0	0/0	0/0	0/0	0/0
1/1	2/2	0/0	0/0	0/0	0/0	2/2	0/0	1/1	1/1	2/2	0/0	2/2	2/2	2/2	2/2	1/1	0/0	0/0	0/0	2/2	1/1	1/1	1/1	1/1	1/1	0/1
0/0	./.	0/0	0/0	0/0	0/0	./.	0/0	0/0	0/0	./.	0/0	./.	./.	./.	./.	0/0	0/0	0/0	0/0	./.	0/0	0/0	0/0	0/0	0/0	0/0
0/0	0/0	0/0	0/0	0/0	0/0	0/0	0/0	1/1	1/1	0/0	0/0	0/0	0/0	0/0	0/0	0/0	0/0	0/0	0/0	0/0	1/1	1/1	1/1	1/1	0/1	0/1
1/1	0/0	1/1	1/1	1/1	0/0	0/0	1/1	1/1	1/1	0/0	1/1	0/0	0/0	0/0	0/0	1/1	1/1	1/1	0/0	0/0	1/1	1/1	1/1	1/1	1/1	0/1
0/0	0/0	0/0	0/0	0/0	0/0	0/0	0/0	0/0	0/0	0/0	0/0	0/0	0/0	0/0	0/0	0/0	0/0	0/0	0/0	0/0	0/0	0/0	0/0	0/0	0/1	0/0
0/0	0/0	0/0	0/0	0/0	0/0	0/0	0/0	0/0	0/0	0/0	0/0	0/0	0/0	0/0	0/0	0/1	0/0	0/0	0/0	0/0	0/0	0/0	0/0	0/0	0/0	0/0
1/1	0/0	1/1	1/1	1/1	0/0	0/0	1/1	1/1	1/1	0/1	1/1	0/0	0/0	0/0	0/0	1/1	1/1	1/1	0/0	0/0	1/1	1/1	1/1	1/1	1/1	0/1
0/0	0/0	0/0	0/0	0/0	0/0	0/0	0/0	1/1	1/1	0/1	0/0	0/0	0/0	0/0	0/0	0/0	0/0	0/0	0/0	0/0	1/1	1/1	1/1	1/1	0/1	0/1
0/0	0/0	0/0	0/0	0/0	0/0	0/0	0/0	0/0	0/0	0/0	0/0	0/0	0/0	0/0	0/0	0/0	0/0	0/0	0/0	0/0	0/0	0/0	0/0	0/0	0/1	0/0
1/1	0/0	1/1	1/1	1/1	0/0	0/0	1/1	0/0	0/0	0/0	1/1	0/0	0/0	0/0	0/0	1/1	1/1	1/1	0/0	0/0	0/0	0/0	0/0	0/0	0/0	0/1
0/0	0/0	1/1	1/1	1/1	0/0	0/0	1/1	0/0	0/0	0/0	1/1	0/0	0/0	0/0	0/0	0/0	1/1	1/1	0/0	0/0	0/0	0/0	0/0	0/0	0/0	0/1
0/0	0/0	0/0	0/0	0/0	0/0	0/0	0/0	0/0	0/0	0/0	0/0	0/0	0/0	0/0	0/0	0/0	0/0	0/0	0/0	0/0	0/0	0/0	0/0	0/0	0/1	0/0
1/1	0/0	1/1	1/1	1/1	0/0	0/0	1/1	0/0	0/0	0/0	1/1	0/0	0/0	0/0	0/0	1/1	1/1	1/1	0/0	0/0	0/0	0/0	0/0	0/0	0/0	0/1
0/0	0/0	0/0	0/0	0/0	0/0	0/0	0/0	0/0	0/0	0/0	0/0	0/0	0/0	0/0	0/0	0/0	0/0	0/0	0/0	0/0	0/0	0/0	0/0	0/0	0/1	0/0
1/1	0/0	1/1	1/1	1/1	0/0	0/0	1/1	1/1	1/1	0/0	1/1	0/0	0/0	0/0	0/0	1/1	1/1	1/1	0/0	0/0	1/1	1/1	1/1	1/1	1/1	0/1
0/0	0/0	0/0	0/0	0/0	0/0	0/0	0/0	1/1	1/1	0/1	0/0	0/0	0/0	0/0	0/0	0/0	0/0	0/0	0/0	0/0	1/1	1/1	1/1	1/1	0/0	0/1
0/0	1/1	1/1	1/1	1/1	0/0	0/0	0/1	0/0	0/0	0/0	1/1	0/0	0/0	0/0	0/0	0/0	1/1	1/1	0/0	0/0	0/0	0/0	0/0	0/0	2/2	0/1

1/1	1/1	1/1	1/1	1/1	0/0	1/1	1/1	1/1	1/1	1/1	1/1	1/1	1/1	1/1	1/1	1/1	1/1	1/1	0/0	1/1	1/1	1/1	1/1	1/1	1/1	0/1
0/0	0/0	0/0	0/0	0/0	0/0	0/0	0/0	1/1	1/1	0/1	0/0	0/0	0/0	0/0	0/0	0/0	0/0	0/0	0/0	0/0	1/1	1/1	1/1	1/1	1/1	0/1
0/0	0/0	0/0	0/0	0/0	0/0	0/0	0/0	1/1	1/1	0/1	0/0	0/0	0/0	0/0	0/0	0/1	0/0	0/0	0/0	0/0	1/1	1/1	1/1	1/1	0/1	0/1
0/0	0/0	0/0	0/0	0/0	0/0	0/0	0/0	0/0	0/0	0/0	0/0	0/0	0/0	0/0	0/0	0/0	0/0	0/0	0/0	0/0	0/0	0/0	0/0	0/0	0/0	0/0
1/1	0/0	1/1	1/1	1/1	0/0	0/0	1/1	1/1	1/1	0/1	1/1	0/0	0/0	0/0	0/0	1/1	1/1	1/1	0/0	0/0	1/1	1/1	1/1	1/1	1/1	1/1
1/1	0/0	1/1	1/1	1/1	0/0	0/0	1/1	1/1	1/1	0/0	1/1	0/0	0/0	0/0	0/0	1/1	1/1	1/1	0/0	0/0	1/1	1/1	1/1	1/1	1/1	0/1
1/1	0/0	1/1	1/1	1/1	0/0	0/0	1/1	1/1	1/1	0/0	1/1	0/0	0/0	0/0	0/0	1/1	1/1	1/1	0/0	0/0	1/1	1/1	1/1	1/1	1/1	1/1
0/0	0/0	0/0	0/0	0/0	0/0	0/0	0/0	0/0	0/0	0/0	0/0	0/0	0/0	0/0	0/0	0/0	0/0	0/0	0/0	0/0	./.	./.	0/0	0/0	1/1	0/0
0/0	0/0	0/0	0/0	0/0	0/0	0/0	0/0	1/1	1/1	0/0	0/0	0/0	0/0	0/0	0/0	0/0	0/0	0/0	0/0	0/0	1/1	1/1	1/1	1/1	0/0	0/1
0/0	0/0	0/0	0/0	0/0	0/0	0/0	0/0	0/0	0/0	0/0	0/0	0/0	0/0	0/0	0/0	0/0	0/0	0/0	0/0	0/0	0/0	0/0	0/0	0/0	0/0	0/0
1/1	0/0	1/1	1/1	1/1	0/0	0/0	1/1	0/0	0/0	0/0	1/1	0/0	0/0	0/0	0/0	1/1	1/1	1/1	0/0	0/0	0/0	0/0	0/0	0/0	0/0	0/1
0/0	0/0	0/0	0/0	0/0	0/0	0/0	0/0	0/0	0/0	0/0	0/0	0/0	0/0	0/0	0/0	0/0	0/0	0/0	0/0	0/0	0/0	0/0	0/0	0/0	0/0	0/0
0/0	0/0	0/0	0/0	0/0	0/0	0/0	0/0	0/0	0/0	0/0	0/0	0/0	0/0	0/0	0/0	0/0	0/0	0/0	0/0	0/0	0/0	0/0	0/0	0/0	0/0	0/0
1/1	0/0	0/0	0/0	0/0	0/0	0/0	0/0	0/0	0/0	0/0	0/0	0/0	0/0	0/0	0/0	1/1	0/0	0/0	0/0	0/0	0/0	0/0	0/0	0/0	0/0	0/0
1/1	0/0	1/1	1/1	1/1	0/0	0/0	1/1	1/1	1/1	0/0	1/1	0/0	0/0	0/0	0/1	1/1	1/1	1/1	0/0	0/0	1/1	1/1	1/1	1/1	1/1	0/1
0/0	0/0	0/0	0/0	0/0	0/0	0/0	0/0	0/0	0/0	0/0	0/0	0/0	0/0	0/0	0/0	0/0	0/0	0/0	0/0	0/0	0/0	0/0	0/0	0/0	1/1	0/0
0/0	0/0	0/0	0/0	0/0	0/0	0/0	0/0	0/0	0/0	0/0	0/0	0/0	0/0	0/0	0/0	0/0	0/0	0/0	0/0	0/0	0/0	0/0	0/0	0/0	0/0	0/0
0/0	0/0	0/0	0/0	0/0	0/0	0/0	0/0	0/0	0/0	0/0	0/0	0/0	0/0	0/0	0/0	0/0	0/0	0/0	0/0	0/0	0/0	0/0	0/0	0/0	0/0	0/0
1/1	0/0	1/1	1/1	1/1	0/0	0/0	1/1	0/0	0/0	0/0	1/1	0/0	0/0	0/0	0/1	1/1	1/1	1/1	0/0	0/0	0/0	0/0	0/0	0/0	0/0	0/1
0/0	0/0	0/0	0/0	0/0	0/0	0/0	0/0	0/0	0/0	0/0	0/0	0/0	0/0	0/0	0/0	0/0	0/0	0/0	0/0	0/0	0/0	0/0	0/0	0/0	1/1	0/0
1/1	0/0	1/1	1/1	1/1	0/0	0/0	1/1	1/1	1/1	0/0	1/1	0/0	0/0	0/0	0/1	1/1	1/1	1/1	0/0	0/0	1/1	1/1	1/1	1/1	1/1	0/1
1/1	0/0	1/1	1/1	1/1	0/0	0/0	1/1	0/0	0/0	0/0	1/1	0/0	0/0	0/0	0/1	1/1	1/1	1/1	0/0	0/0	0/0	0/0	0/0	0/0	0/0	0/1
1/1	0/0	1/1	1/1	1/1	0/0	0/0	1/1	0/0	0/0	0/0	1/1	0/0	0/0	0/0	0/0	1/1	1/1	1/1	0/0	0/0	0/0	0/0	0/0	0/0	0/0	0/1
0/0	0/0	0/0	0/0	0/0	0/0	0/0	0/0	1/1	1/1	0/0	0/0	0/0	0/0	0/0	0/0	0/0	0/0	0/0	0/0	0/0	1/1	1/1	1/1	1/1	1/1	0/1
1/1	0/0	1/1	1/1	1/1	0/0	0/0	1/1	1/1	1/1	0/0	1/1	0/0	0/0	0/0	0/0	1/1	1/1	1/1	0/0	0/0	1/1	1/1	1/1	1/1	1/1	0/1
0/0	0/0	0/0	0/0	0/0	0/0	0/0	0/0	1/1	1/1	0/0	0/0	0/0	0/0	0/0	0/0	0/0	0/0	0/0	0/0	0/0	1/1	1/1	1/1	1/1	1/1	0/0
0/0	0/0	0/0	0/0	0/0	0/0	0/0	0/0	0/0	0/0	0/0	0/0	0/0	0/0	0/0	0/0	0/0	0/0	0/0	0/0	0/0	0/0	0/0	0/0	0/0	0/0	0/0
0/0	0/0	0/0	0/0	0/0	0/0	0/0	0/0	0/0	0/0	0/0	0/0	0/0	0/0	0/0	0/0	0/0	0/0	0/0	0/0	0/0	0/0	0/0	0/0	0/0	1/1	0/0
1/1	1/1	1/1	1/1	1/1	0/0	1/1	1/1	1/1	1/1	1/1	1/1	1/1	1/1	1/1	1/1	1/1	1/1	1/1	0/0	1/1	1/1	1/1	1/1	1/1	1/1	0/1
0/0	0/0	0/0	0/0	0/0	0/0	0/0	0/0	0/0	0/0	0/0	0/0	0/0	0/0	0/0	0/0	0/0	0/0	0/0	1/1	0/0	0/0	0/0	0/0	0/0	0/0	0/1
1/1	0/0	1/1	1/1	1/1	0/0	0/0	1/1	1/1	1/1	0/0	1/1	0/0	0/0	0/0	0/0	1/1	1/1	1/1	0/0	0/0	1/1	1/1	1/1	1/1	1/1	0/1
0/0	0/0	0/0	0/0	0/0	0/0	0/0	0/0	1/1	1/1	0/0	0/0	0/0	0/0	0/0	0/0	0/0	0/0	0/0	0/0	0/0	1/1	1/1	1/1	1/1	0/0	0/1
1/1	0/0	1/1	1/1	1/1	0/0	0/0	1/1	1/1	1/1	0/0	1/1	0/0	0/0	0/0	0/0	1/1	1/1	1/1	0/0	0/0	1/1	1/1	1/1	1/1	1/1	0/1
0/0	0/0	0/0	0/0	0/0	0/0	0/0	0/0	0/0	0/0	0/0	1/1	0/0	0/0	0/0	0/0	0/0	0/0	0/0	0/0	0/0	0/0	0/0	0/0	0/0	0/0	0/0
0/0	0/0	0/0	0/0	0/0	0/0	0/0	0/0	1/1	1/1	0/0	0/0	0/0	0/0	0/0	0/0	0/0	0/0	0/0	0/0	0/0	1/1	1/1	1/1	1/1	1/1	0/1

1/1	0/0	1/1	1/1	1/1	0/0	0/0	1/1	0/0	0/0	0/0	1/1	0/0	0/0	0/0	0/0	1/1	1/1	1/1	0/0	0/0	0/0	0/0	0/0	0/0	0/0	0/1
0/0	0/0	0/0	0/0	0/0	0/0	0/0	0/0	0/0	0/0	0/0	0/0	0/0	0/0	0/0	0/0	0/0	0/0	0/0	0/0	0/0	0/0	0/0	1/1	0/0	0/0	0/1
0/0	0/0	1/1	1/1	1/1	0/0	0/0	1/1	0/0	0/0	0/0	1/1	0/0	0/0	0/0	0/0	0/0	1/1	1/1	0/0	0/0	0/0	0/0	0/0	0/0	0/0	0/1
1/1	0/0	1/1	1/1	1/1	0/0	0/0	1/1	1/1	1/1	0/1	1/1	0/0	0/0	0/0	0/0	1/1	1/1	1/1	0/0	0/0	1/1	1/1	1/1	1/1	1/1	0/1
0/0	0/0	0/0	0/0	0/0	0/0	0/0	0/0	0/0	0/0	0/0	0/0	0/0	0/0	0/0	0/0	0/0	0/0	0/0	0/0	0/0	0/0	0/0	0/0	0/0	0/0	0/0
1/1	0/0	1/1	1/1	1/1	0/0	0/0	1/1	0/0	0/0	0/0	1/1	0/0	0/0	0/0	0/0	1/1	1/1	1/1	0/0	0/0	0/0	0/0	0/0	0/0	0/0	0/1
1/1	0/0	1/1	1/1	1/1	0/0	0/0	1/1	1/1	1/1	0/1	1/1	0/0	0/0	0/0	0/0	1/1	1/1	1/1	0/0	0/0	1/1	1/1	1/1	1/1	1/1	0/1
0/0	0/0	0/0	0/0	0/0	0/0	0/0	0/0	1/1	1/1	0/1	0/0	0/0	0/0	0/0	0/0	0/0	0/0	0/0	0/0	0/0	1/1	1/1	1/1	1/1	0/0	0/1
1/1	0/0	1/1	1/1	1/1	0/0	0/0	1/1	0/0	0/0	0/0	1/1	0/0	0/0	0/0	0/0	1/1	1/1	1/1	0/0	0/0	0/0	0/0	0/0	0/0	0/0	0/1
0/0	0/0	0/0	0/0	0/0	0/0	0/0	0/0	0/0	0/0	0/0	0/0	0/0	0/0	0/0	0/0	0/0	0/0	0/0	0/0	0/0	0/0	0/0	0/0	0/0	0/0	0/0
1/1	0/0	1/1	1/1	1/1	0/0	0/0	1/1	0/0	0/0	0/0	1/1	0/0	0/0	0/0	0/0	1/1	1/1	1/1	0/0	0/0	0/0	0/0	0/0	0/0	0/0	0/1
0/0	0/0	0/0	0/0	0/0	0/0	0/0	0/0	0/0	0/0	0/0	0/0	0/0	0/0	0/0	0/0	0/0	0/0	0/0	0/0	0/0	0/0	0/0	0/0	0/0	1/1	0/0
0/0	0/0	0/0	0/0	0/0	0/0	0/0	0/0	0/0	0/0	0/0	0/0	0/0	0/0	0/0	0/0	0/0	0/0	0/0	0/0	0/0	0/0	0/0	0/0	0/0	1/1	0/0
0/0	0/0	0/0	0/0	0/0	0/0	0/0	0/0	0/0	0/0	0/0	0/0	0/0	0/0	0/0	0/0	0/0	0/0	0/0	0/0	0/0	0/0	0/0	0/0	0/0	0/0	0/0
1/1	1/1	1/1	1/1	1/1	0/0	1/1	1/1	1/1	1/1	1/1	1/1	1/1	1/1	1/1	1/1	1/1	1/1	1/1	0/0	1/1	1/1	1/1	1/1	1/1	1/1	0/1
1/1	1/1	1/1	1/1	1/1	0/0	1/1	1/1	1/1	1/1	1/1	1/1	1/1	1/1	1/1	1/1	1/1	1/1	1/1	0/0	1/1	1/1	1/1	1/1	1/1	1/1	0/1
1/1	0/0	1/1	1/1	1/1	0/0	0/0	1/1	1/1	1/1	0/1	1/1	0/0	0/0	0/0	0/0	1/1	1/1	1/1	0/0	0/0	1/1	1/1	1/1	1/1	1/1	0/1
./.	./.	1/1	1/1	1/1	0/0	./.	./.	./.	./.	./.	./.	./.	./.	./.	./.	./.	1/1	./.	0/0	./.	./.	0/0	0/0	./.	./.	0/0
1/1	1/1	4/4	4/4	4/4	0/0	1/1	1/1	3/3	3/3	1/3	1/1	1/1	1/1	1/1	1/1	1/1	4/4	1/1	0/0	1/1	3/3	3/3	3/3	3/3	3/3	1/3
0/0	0/0	0/0	0/0	0/0	0/0	0/0	0/0	0/0	0/0	0/1	0/0	0/0	0/0	0/0	0/0	0/0	0/0	0/0	0/0	0/0	0/0	0/0	0/0	0/0	0/0	0/0
0/0	0/0	0/0	0/0	0/0	0/0	0/0	0/0	0/0	0/0	0/0	0/0	0/0	0/0	0/0	0/0	0/0	0/0	0/0	0/0	0/0	0/0	0/0	0/0	0/0	0/0	0/0
0/0	0/0	0/0	0/0	0/0	0/0	0/0	0/0	0/0	0/0	0/0	0/0	0/0	0/0	0/0	0/0	0/0	0/0	0/0	0/0	0/0	0/0	0/0	0/0	0/0	0/0	0/0
0/0	0/0	0/0	0/0	0/0	0/0	0/0	0/0	0/0	0/0	0/0	0/0	0/0	0/0	0/0	0/0	0/0	0/0	0/0	0/0	0/0	0/0	0/0	0/0	0/0	0/0	0/0
0/0	0/0	0/0	0/0	0/0	0/0	0/0	0/0	0/0	0/0	0/0	0/0	0/0	0/0	0/0	0/0	0/0	0/0	0/0	0/0	0/0	0/0	0/0	0/0	0/0	0/1	0/0
0/0	0/0	0/0	0/0	0/0	0/0	0/0	0/0	1/1	1/1	0/1	0/0	0/0	0/0	0/0	0/0	0/0	0/0	0/0	0/0	0/0	1/1	1/1	1/1	1/1	0/1	0/1

0/0	0/0	0/0	0/0	0/0	0/0	0/0	0/0	0/0	0/0	0/0	0/0	0/0	0/0	0/0	0/0	0/0	0/0	0/0	0/0	0/0	0/0	0/0	0/0	0/0	0/0	0/0
0/0	0/0	0/0	0/0	0/0	0/0	0/0	0/0	1/1	1/1	0/1	0/0	0/0	0/0	0/0	0/0	0/0	0/0	0/0	0/0	0/0	1/1	1/1	1/1	1/1	0/1	0/1
0/0	0/0	0/0	0/0	0/0	0/0	0/0	0/0	1/1	1/1	0/1	0/0	0/0	0/0	0/0	0/0	0/0	0/0	0/0	0/0	0/0	1/1	1/1	1/1	1/1	0/1	0/1
1/1	1/1	1/1	1/1	1/1	0/0	1/1	1/1	1/1	1/1	1/1	1/1	1/1	1/1	1/1	1/1	1/1	1/1	1/1	0/0	1/1	1/1	1/1	1/1	1/1	1/1	0/1
1/1	0/0	1/1	1/1	1/1	0/0	0/0	1/1	1/1	1/1	0/0	1/1	0/0	0/0	0/0	0/0	1/1	1/1	1/1	0/0	0/0	1/1	1/1	1/1	1/1	1/1	0/1
0/0	0/0	0/0	0/0	0/0	0/0	0/0	0/0	0/0	1/1	0/0	0/0	0/0	0/0	0/0	0/0	0/0	0/0	0/0	0/0	0/0	0/0	0/0	0/0	0/0	0/0	0/0
1/1	0/0	1/1	1/1	1/1	0/0	0/0	1/1	1/1	1/1	0/1	1/1	0/0	0/0	0/0	0/0	1/1	1/1	1/1	0/0	0/0	1/1	1/1	1/1	1/1	1/1	0/1
0/0	0/0	0/0	0/0	0/0	0/0	0/0	0/1	1/1	1/1	0/0	0/0	0/0	0/0	0/0	0/0	0/0	0/0	0/0	0/0	0/0	1/1	1/1	1/1	1/1	0/0	0/1
0/0	0/0	0/0	0/0	0/0	0/0	0/0	0/0	0/0	0/0	0/0	0/0	0/0	0/0	0/0	0/0	0/0	0/0	0/0	0/0	0/0	0/0	0/0	0/0	0/0	0/1	0/0
0/0	0/0	0/0	0/0	0/0	0/0	0/0	0/0	0/0	0/0	0/0	0/0	0/0	0/0	0/0	0/0	0/0	0/0	0/0	0/0	0/0	0/0	0/0	0/0	0/0	0/0	0/0
0/0	0/0	0/0	0/0	0/0	0/0	0/0	0/0	1/1	1/1	0/0	0/0	0/0	0/0	0/0	0/0	0/0	0/0	0/0	0/0	0/0	1/1	1/1	1/1	1/1	0/1	0/1
0/0	0/0	0/0	0/0	0/0	0/0	0/0	0/0	0/0	0/0	0/0	0/0	0/0	0/0	0/0	0/0	0/0	0/0	0/0	0/0	0/0	0/0	0/0	0/0	0/0	0/0	0/0
1/1	0/0	1/1	1/1	1/1	0/0	0/0	1/1	0/0	0/0	0/0	1/1	0/0	0/0	0/0	0/0	1/1	1/1	1/1	0/0	0/0	0/0	0/0	0/0	0/0	0/0	0/1
0/0	0/0	0/0	0/0	0/0	0/0	0/0	0/0	1/1	1/1	0/0	0/0	0/0	0/0	0/0	0/0	0/0	0/0	0/0	0/0	0/0	1/1	1/1	1/1	1/1	0/1	0/1
0/0	0/0	0/0	0/0	0/0	0/0	0/0	0/0	0/0	0/0	0/0	0/0	0/0	0/0	0/0	0/0	0/0	0/0	0/0	0/0	0/0	0/0	0/0	0/0	0/0	0/0	0/0
1/1	0/0	1/1	1/1	1/1	0/0	0/0	1/1	1/1	1/1	0/0	1/1	0/0	0/0	0/0	0/0	1/1	1/1	1/1	0/0	0/0	1/1	1/1	1/1	1/1	1/1	0/1
1/1	0/0	1/1	1/1	1/1	0/0	0/0	1/1	0/0	0/0	0/0	1/1	0/0	0/0	0/0	0/0	1/1	1/1	1/1	0/0	0/0	0/0	0/0	0/0	0/0	0/0	0/1
1/1	0/0	0/0	0/0	0/0	0/0	0/0	0/0	0/0	0/0	0/0	0/0	0/0	0/0	0/0	0/0	1/1	0/0	0/0	0/0	0/0	0/0	0/0	0/0	0/0	0/0	0/0
0/0	0/0	0/0	0/0	0/0	0/0	0/0	0/0	0/0	0/0	0/0	0/0	0/0	0/0	0/0	0/0	0/0	0/0	0/0	0/0	0/0	0/0	0/0	./.	./.	0/0	0/0
0/0	0/0	0/0	0/0	0/0	0/0	0/0	0/0	1/1	1/1	0/0	0/0	0/0	0/0	0/0	0/0	0/0	0/0	0/0	0/0	0/0	1/1	1/1	1/1	1/1	0/0	0/1
0/0	0/0	0/0	0/0	0/0	0/0	0/0	0/0	1/1	1/1	0/0	0/0	0/0	0/0	0/0	0/0	0/0	0/0	0/0	0/0	0/0	1/1	1/1	1/1	1/1	0/0	0/1
0/0	0/0	0/0	0/0	0/0	0/0	0/0	0/0	0/0	0/0	0/0	0/0	0/0	0/0	0/0	0/0	0/0	0/0	0/0	0/0	0/0	0/0	0/0	0/0	0/0	0/0	0/0
1/1	0/0	1/1	1/1	1/1	0/0	0/0	1/1	1/1	1/1	0/0	1/1	0/0	0/0	0/0	0/0	1/1	1/1	1/1	0/0	0/0	1/1	1/1	1/1	1/1	1/1	0/1
0/0	0/0	0/0	0/0	0/0	0/0	0/0	0/0	0/0	0/0	0/0	0/0	0/0	0/0	0/0	0/0	0/0	0/0	0/0	0/0	0/0	0/0	0/0	0/0	0/0	0/0	0/0
0/0	0/0	0/0	0/0	0/0	0/0	0/0	0/0	0/0	0/0	0/0	0/0	0/0	0/0	0/0	0/0	0/0	0/0	0/0	0/0	0/0	0/0	0/0	0/0	0/0	1/1	0/0
0/0	0/0	0/0	0/0	0/0	0/0	0/0	0/0	1/1	1/1	0/0	0/0	0/0	0/0	0/0	0/0	0/0	0/0	0/0	0/0	0/0	1/1	1/1	1/1	1/1	0/0	0/1
0/0	0/0	0/0	0/0	0/0	0/0	0/0	0/0	0/0	0/0	0/0	0/0	0/0	0/0	0/0	0/0	0/0	0/0	0/0	0/0	0/0	0/0	0/0	0/0	0/0	0/0	0/0
1/1	0/0	1/1	1/1	1/1	0/0	0/0	1/1	0/0	0/0	0/0	1/1	0/0	0/0	0/0	0/0	1/1	1/1	1/1	0/0	0/0	0/0	0/0	0/0	0/0	0/0	0/1
0/0	0/0	0/0	0/0	0/0	0/0	0/0	0/0	1/1	1/1	0/0	0/0	0/0	0/0	0/0	0/0	0/0	0/0	0/0	0/0	0/0	1/1	1/1	1/1	1/1	0/0	0/1
0/0	0/0	0/0	0/0	0/0	0/0	0/0	0/0	0/0	0/0	0/0	0/0	0/0	0/0	0/0	0/0	0/0	0/0	0/0	0/0	0/0	0/0	0/0	0/0	0/0	0/0	0/0
1/1	0/0	1/1	1/1	1/1	0/0	0/0	1/1	1/1	1/1	0/0	1/1	0/0	0/0	0/0	0/0	1/1	1/1	1/1	0/0	0/0	1/1	1/1	1/1	1/1	1/1	0/1
0/0	0/0	0/0	0/0	0/0	0/0	0/0	0/0	0/0	0/0	0/0	0/0	0/0	0/0	0/0	0/0	0/0	0/0	0/0	0/0	0/0	0/0	0/0	0/0	0/0	0/0	0/0
0/0	1/1	0/0	0/0	0/0	0/0	1/1	0/0	0/0	0/0	1/1	0/0	1/1	1/1	1/1	1/1	0/0	0/0	0/0	0/0	1/1	0/0	0/0	0/0	0/0	0/0	0/0

1/1	0/0	1/1	1/1	1/1	0/0	0/0	1/1	1/1	1/1	0/1	1/1	0/0	0/0	0/0	0/0	1/1	1/1	1/1	0/0	0/0	1/1	1/1	1/1	1/1	1/1	0/1
0/0	0/0	0/0	0/0	0/0	0/0	0/0	0/0	0/0	0/0	0/0	0/0	0/0	0/0	0/0	0/0	0/0	0/0	0/0	0/0	0/0	0/0	0/0	0/0	0/0	0/0	0/0
1/1	0/0	1/1	1/1	1/1	0/0	0/0	1/1	1/1	1/1	0/1	1/1	0/0	0/0	0/0	0/0	1/1	1/1	1/1	0/0	0/0	1/1	1/1	1/1	1/1	1/1	0/1
0/0	0/0	0/0	0/0	0/0	0/0	0/0	0/0	0/0	0/0	0/0	0/0	0/0	0/0	0/0	0/0	0/0	0/0	0/0	0/0	0/0	0/0	0/0	0/0	0/0	0/0	0/0
0/0	0/0	1/1	1/1	1/1	0/0	0/0	0/1	0/0	0/0	0/0	1/1	0/0	0/0	0/0	0/0	0/0	1/1	1/1	0/0	0/0	0/0	0/0	0/0	0/0	0/0	0/1
0/0	0/0	0/0	0/0	0/0	0/0	0/0	0/0	0/0	0/0	0/0	0/0	0/0	0/0	0/0	0/0	0/0	0/0	0/0	0/0	0/0	0/0	0/0	0/0	0/0	0/0	0/0
0/0	0/0	0/0	0/0	0/0	0/0	0/0	0/0	0/0	0/0	0/0	0/0	0/0	0/0	0/0	0/0	0/0	0/0	0/0	0/0	0/0	0/0	0/0	0/0	0/0	0/1	0/0
1/1	0/0	0/0	0/0	0/0	0/0	0/0	0/0	0/0	0/0	0/0	0/0	0/0	0/0	0/0	0/0	1/1	0/0	0/0	0/0	0/0	0/0	0/0	0/0	0/0	0/0	0/0
0/0	0/0	0/0	0/0	0/0	0/0	0/0	0/0	0/0	0/0	0/0	0/0	0/0	0/0	0/0	0/0	0/0	0/0	0/0	0/0	0/0	0/0	0/0	0/0	0/0	0/0	0/0
0/0	0/0	0/0	0/0	0/0	0/0	0/0	0/0	0/0	0/0	0/0	0/0	0/0	0/0	0/0	0/0	0/0	0/0	0/0	0/0	0/0	0/0	0/0	0/0	0/0	0/1	0/0
1/1	0/0	1/1	1/1	1/1	0/0	0/0	1/1	1/1	1/1	0/1	1/1	0/0	0/0	0/0	0/0	1/1	1/1	1/1	0/0	0/0	1/1	1/1	1/1	1/1	1/1	0/1
1/1	0/0	1/1	1/1	1/1	0/0	0/0	1/1	1/1	1/1	0/1	1/1	0/0	0/0	0/0	0/0	1/1	1/1	1/1	0/0	0/0	1/1	1/1	1/1	1/1	1/1	0/1
0/0	0/0	0/0	0/0	0/0	0/0	0/0	0/0	0/0	0/0	0/0	0/0	0/0	0/0	0/0	0/0	0/0	0/0	0/0	0/0	0/0	0/0	0/0	0/0	0/0	0/1	0/0
0/0	0/0	1/1	1/1	1/1	0/0	0/0	1/1	0/0	0/0	0/0	1/1	0/0	0/0	0/0	0/0	0/0	1/1	1/1	0/0	0/0	0/0	0/0	0/0	0/0	0/0	0/1
0/0	0/0	0/0	0/0	0/0	0/0	0/0	0/0	1/1	1/1	0/1	0/0	0/0	0/0	0/0	0/0	0/0	0/0	0/0	0/0	0/0	1/1	1/1	1/1	1/1	0/1	0/1
1/1	1/1	1/1	1/1	1/1	0/0	1/1	1/1	1/1	1/1	1/1	1/1	2/2	1/1	1/1	1/1	0/1	1/1	1/1	0/0	2/2	1/1	1/1	1/1	1/1	0/1	0/1
1/1	0/0	1/1	1/1	1/1	0/0	0/0	1/1	1/1	1/1	0/1	1/1	0/0	0/0	0/0	0/0	1/1	1/1	1/1	0/0	0/0	1/1	1/1	1/1	1/1	1/1	0/1
0/0	0/0	0/0	0/0	0/0	0/0	0/0	0/0	1/1	1/1	0/1	0/0	0/0	0/0	0/0	0/0	0/0	0/0	0/0	0/0	0/0	1/1	1/1	1/1	1/1	0/0	0/1
0/0	0/0	0/0	0/0	0/0	0/0	0/0	0/0	1/1	1/1	0/0	0/0	0/0	0/0	0/0	0/0	0/0	0/0	0/0	0/0	0/0	1/1	1/1	1/1	1/1	0/0	0/1
0/0	0/0	1/1	1/1	1/1	0/0	0/0	1/1	0/0	0/0	0/0	1/1	0/0	0/0	0/0	0/0	0/0	1/1	1/1	0/0	0/0	0/0	0/0	0/0	0/0	0/0	0/1
0/0	0/0	0/0	0/0	0/0	0/0	0/0	0/0	0/0	0/0	0/0	0/0	0/0	0/0	0/0	0/0	0/0	0/0	0/0	0/0	0/0	0/0	0/0	0/0	0/0	0/0	0/0
0/0	0/0	0/0	0/0	0/0	0/0	0/0	0/0	0/0	0/0	0/0	0/0	0/0	0/0	0/0	0/0	0/0	0/0	0/0	0/0	0/0	0/0	0/0	0/0	0/0	0/0	0/0
0/0	0/0	0/0	0/0	0/0	0/0	0/0	0/0	0/0	0/0	0/0	0/0	0/0	0/0	0/1	0/0	0/0	0/0	0/0	0/0	0/0	0/0	0/0	0/1	0/0	0/0	0/0
0/0	0/0	0/0	0/0	0/0	0/0	0/0	0/0	0/0	0/0	0/0	0/0	0/0	0/0	0/0	0/0	0/0	0/0	0/0	0/0	0/0	0/0	0/0	0/0	0/0	0/0	0/0
0/0	0/0	0/0	0/0	0/1	0/0	0/0	0/0	0/0	0/0	0/0	0/0	0/0	0/0	0/1	0/0	0/0	0/0	0/0	0/0	0/0	0/0	0/0	0/1	0/0	0/0	0/0
0/0	0/0	0/0	0/0	0/1	0/0	0/0	0/0	0/0	0/0	0/0	0/0	0/0	0/0	0/1	0/0	0/0	0/0	0/0	0/0	0/0	0/0	0/0	0/1	0/0	0/0	0/0

0/0	0/0	0/0	0/0	0/1	0/0	0/0	0/0	0/0	0/0	0/0	0/0	0/0	0/0	0/1	0/0	0/0	0/0	0/0	0/0	0/0	0/0	0/0	0/1	0/0	0/0	0/0
0/0	0/0	0/0	0/0	0/1	0/0	0/0	0/0	0/0	0/0	0/0	0/0	0/0	0/0	0/1	0/0	0/0	0/0	0/0	0/0	0/0	0/0	0/0	0/1	0/0	0/0	0/0
0/0	0/0	0/0	0/0	0/1	0/0	0/0	0/0	0/0	0/0	0/0	0/0	0/0	0/0	0/1	0/0	0/0	0/0	0/0	0/0	0/0	0/0	0/0	0/1	0/0	0/0	0/0
0/0	0/0	0/0	0/0	0/1	0/0	0/0	0/0	0/0	0/0	0/0	0/0	0/0	0/0	0/1	0/0	0/0	0/0	0/0	0/0	0/0	0/0	0/0	0/1	0/0	0/0	0/0
0/0	0/0	0/0	0/0	0/1	0/0	0/0	0/0	0/0	0/0	0/0	0/0	0/0	0/0	0/1	0/0	0/0	0/0	0/0	0/0	0/0	0/0	0/0	0/1	0/0	0/0	0/0
0/0	0/0	0/0	0/0	0/1	0/0	0/0	0/0	0/0	0/0	0/0	0/0	0/0	0/0	0/1	0/0	0/0	0/0	0/0	0/0	0/0	0/0	0/0	0/1	0/0	0/0	0/0
0/0	0/0	0/0	0/0	0/1	0/0	0/0	0/0	0/0	0/0	0/0	0/0	0/0	0/0	0/0	0/0	0/0	0/0	0/0	0/0	0/0	0/0	0/0	0/0	0/0	0/0	0/0
0/0	0/0	0/0	0/0	0/2	0/0	0/0	0/0	0/0	0/0	0/0	0/0	0/0	0/0	0/0	0/0	0/0	0/0	0/0	0/0	0/0	0/0	0/0	0/0	0/0	0/0	0/0
0/0	0/0	0/0	0/0	0/2	0/0	0/0	0/0	0/0	0/0	0/0	0/0	0/0	0/0	0/0	0/0	0/0	0/0	0/0	0/0	0/0	0/0	0/0	0/0	0/0	0/0	0/0
0/0	0/0	0/0	0/0	0/0	0/0	0/0	0/0	0/0	0/0	0/0	0/0	0/0	0/0	0/0	0/0	0/0	0/0	0/0	0/0	0/0	0/0	0/0	0/0	0/0	0/0	0/0
1/1	1/1	1/1	1/1	1/1	0/0	1/1	1/1	1/1	1/1	1/1	1/1	1/1	1/1	1/1	1/1	1/1	1/1	1/1	0/0	1/1	1/1	1/1	1/1	1/1	1/1	0/1
0/0	0/0	0/0	0/0	0/0	0/0	0/0	0/0	0/0	0/0	0/0	0/0	0/0	0/0	0/0	0/0	0/0	0/0	0/0	0/0	0/0	0/0	0/0	0/0	0/0	0/0	0/0
0/0	0/0	0/0	0/0	0/0	0/0	0/0	0/0	0/0	0/0	0/0	0/0	0/0	0/0	0/0	0/0	0/0	0/0	0/0	0/0	0/0	0/0	0/0	0/0	0/0	0/0	0/0
1/1	0/0	0/0	0/0	0/0	0/0	0/0	0/0	0/0	0/0	0/0	0/0	0/0	0/0	0/0	0/0	1/1	0/0	0/0	0/0	0/0	0/0	0/0	0/0	0/0	0/1	0/0
0/0	0/0	0/0	0/0	0/0	0/0	0/0	0/0	0/0	0/0	0/0	0/0	0/0	0/0	0/0	0/0	0/0	0/0	0/0	0/0	0/0	0/0	0/0	0/0	0/0	0/0	0/0
0/0	0/0	0/0	0/0	0/0	0/0	0/0	0/0	0/0	0/0	0/0	0/0	0/0	0/0	0/0	0/0	0/0	0/0	0/0	0/0	0/0	0/0	0/0	0/0	0/0	0/0	0/0
1/1	0/0	1/1	1/1	1/1	0/0	0/0	1/1	1/1	1/1	0/1	1/1	0/0	0/0	0/0	0/1	1/1	1/1	1/1	0/0	0/0	1/1	1/1	1/1	1/1	1/1	0/1
0/0	0/0	0/0	0/0	0/0	0/0	0/0	0/0	0/0	./.	0/0	0/0	0/0	0/0	0/0	0/0	0/0	0/0	0/0	0/0	0/0	./.	0/0	./.	./.	0/0	0/0
1/1	0/0	1/1	1/1	1/1	0/0	0/0	0/1	./.	./.	0/0	1/1	0/0	0/0	0/0	0/0	1/1	1/1	1/1	0/0	0/0	./.	0/0	./.	0/0	0/1	0/1
0/0	0/0	0/0	0/0	0/0	0/0	0/0	0/0	1/1	1/1	0/1	0/0	0/0	0/0	0/0	0/0	0/0	0/0	0/0	0/0	0/0	1/1	1/1	1/1	1/1	0/0	0/1
0/0	0/0	0/0	0/0	0/0	0/0	0/0	0/1	1/1	1/1	0/1	0/0	0/0	0/0	0/0	0/0	0/0	0/0	0/0	0/0	0/0	1/1	1/1	1/1	1/1	0/0	0/1
0/0	0/0	0/0	0/0	0/0	0/0	0/0	0/0	0/0	0/0	0/0	./.	0/0	0/0	./.	0/0	./.	./.	./.	0/0	./.	0/0	0/0	0/0	0/0	0/0	0/0
0/0	0/0	./.	./.	./.	0/0	./.	0/0	0/0	0/0	0/0	./.	0/0	0/0	./.	./.	./.	./.	./.	0/0	./.	0/0	0/0	0/0	0/0	0/0	0/0
1/1	0/0	1/1	1/1	1/1	0/0	0/0	0/1	0/0	0/0	0/0	1/1	0/0	0/0	0/0	./.	0/1	1/1	1/1	0/0	./.	0/0	0/0	0/0	0/0	0/1	0/1

2/2	0/0	2/2	0/0	0/0	0/0	0/0	0/0	0/0	0/0	0/0	0/0	0/0	0/0	./.	./.	0/1	2/2	2/2	0/0	./.	0/0	0/0	0/0	0/0	0/0	0/2
4/4	3/3	4/4	4/4	4/4	0/0	3/3	0/4	2/2	0/0	0/3	4/4	3/3	3/3	3/3	3/3	0/4	4/4	4/4	1/1	3/3	0/0	0/0	0/0	0/0	0/0	0/0
0/0	0/0	0/0	0/0	0/0	0/0	0/0	0/0	0/0	0/0	0/0	0/0	0/0	0/0	0/0	0/0	0/0	0/0	0/0	0/0	0/0	0/0	0/0	0/0	0/0	0/0	0/0
1/1	0/0	1/1	1/1	1/1	0/0	0/0	1/1	1/1	1/1	0/1	1/1	0/0	0/0	0/0	0/0	1/1	1/1	1/1	0/0	0/0	1/1	1/1	1/1	1/1	1/1	0/1
1/1	0/0	1/1	1/1	1/1	0/0	0/0	1/1	0/0	0/0	0/0	1/1	0/0	0/0	0/0	0/0	1/1	1/1	1/1	0/0	0/0	0/0	0/0	0/0	0/0	0/1	0/0
0/0	0/0	0/0	0/0	0/0	0/0	0/0	0/0	0/0	0/0	0/0	0/0	0/0	0/0	0/0	0/0	0/0	0/0	0/0	0/0	0/0	0/0	0/0	0/0	0/0	0/0	0/0
0/0	0/0	0/0	0/0	0/0	0/0	0/0	0/0	0/0	0/0	0/0	0/0	0/0	0/0	0/0	0/0	0/0	0/0	0/0	0/0	0/0	0/0	0/0	0/0	0/0	0/0	0/0
1/1	0/0	1/1	1/1	1/1	0/0	0/0	1/1	1/1	1/1	0/1	1/1	0/0	0/0	0/0	0/0	1/1	1/1	1/1	0/0	0/0	1/1	1/1	1/1	1/1	1/1	0/1
0/0	0/0	./.	0/0	./.	0/0	0/0	0/0	0/0	0/0	0/0	0/0	0/0	0/0	0/0	0/0	0/0	0/0	0/0	0/0	0/0	0/0	0/0	0/0	0/0	0/0	0/0
1/1	0/0	1/1	1/1	1/1	0/0	0/0	1/1	0/0	0/0	0/0	1/1	0/0	0/0	0/0	0/0	1/1	1/1	1/1	0/0	0/0	0/0	0/0	0/0	0/0	1/1	0/1
1/1	0/0	1/1	1/1	1/1	0/0	0/0	1/1	0/0	0/0	0/0	1/1	0/0	0/0	0/0	0/0	1/1	1/1	1/1	0/0	0/0	0/0	0/0	0/0	0/0	1/1	0/1
1/1	0/0	1/1	1/1	1/1	0/0	0/0	1/1	0/0	0/0	0/0	1/1	0/0	0/0	0/0	0/0	1/1	1/1	1/1	0/0	0/0	0/0	0/0	0/0	0/0	1/1	0/1
0/0	0/0	0/0	0/0	0/0	0/0	0/0	0/0	1/1	1/1	0/1	0/0	0/0	0/0	0/0	0/0	0/0	0/0	0/0	0/0	0/0	1/1	1/1	1/1	1/1	0/0	0/1
1/1	0/0	1/1	1/1	1/1	0/0	0/0	1/1	0/0	0/0	0/0	1/1	0/0	0/0	0/0	0/0	1/1	1/1	1/1	0/0	0/0	0/0	0/0	0/0	0/0	1/1	0/1
1/1	0/0	1/1	1/1	1/1	0/0	0/0	1/1	1/1	1/1	0/1	1/1	0/0	0/0	0/0	0/0	1/1	1/1	1/1	0/0	0/0	1/1	1/1	1/1	1/1	1/1	0/1
0/0	0/0	0/0	0/0	0/0	0/0	0/0	0/0	0/0	0/0	0/0	0/0	0/0	0/0	0/0	0/0	0/0	0/0	0/0	0/0	0/0	0/0	0/0	0/0	0/0	0/0	0/0
0/0	0/0	0/0	0/0	0/0	0/0	0/0	0/0	0/0	0/0	0/0	0/0	0/0	0/0	0/0	0/0	0/0	0/0	0/0	0/0	0/0	0/0	0/0	0/0	0/0	0/0	0/0
0/0	0/0	0/0	0/0	0/0	0/0	0/0	0/0	1/1	1/1	0/1	0/0	0/0	0/0	0/0	0/0	0/1	0/0	0/0	0/0	0/0	1/1	1/1	1/1	1/1	0/0	0/1
0/0	0/0	0/0	0/0	0/0	0/0	0/0	0/0	0/0	0/0	0/0	0/0	0/0	0/0	0/0	0/0	0/0	0/0	0/0	0/0	0/0	0/0	0/0	0/0	0/0	0/0	0/0
0/0	0/0	0/0	0/0	0/0	0/0	0/0	0/0	1/1	1/1	0/1	0/0	0/0	0/0	0/0	0/0	0/1	0/0	0/0	0/0	0/0	1/1	1/1	1/1	1/1	0/0	0/1
1/1	0/0	0/0	0/0	0/0	0/0	0/0	0/0	0/0	0/0	0/0	0/0	0/0	0/0	0/0	0/0	0/1	0/0	0/0	0/0	0/0	0/0	0/0	0/0	0/0	1/1	0/0
0/0	0/0	0/0	0/0	0/0	0/0	0/0	0/0	1/1	1/1	0/0	0/0	0/0	0/0	0/0	0/0	0/1	0/0	0/0	0/0	0/0	1/1	1/1	1/1	1/1	0/0	0/1
0/0	0/0	0/0	0/0	0/0	0/0	0/0	0/0	1/1	1/1	0/0	0/0	0/0	0/0	0/0	0/0	0/1	0/0	0/0	0/0	0/0	1/1	1/1	1/1	1/1	0/0	0/1
0/0	0/0	0/0	0/0	0/0	0/0	0/0	0/0	0/0	0/0	0/0	0/0	0/0	0/0	0/0	0/0	0/0	0/0	0/0	0/0	0/0	0/0	0/0	0/0	0/0	0/0	0/0
0/0	0/0	0/0	0/0	0/0	0/0	0/0	0/0	0/0	0/0	0/0	0/0	0/0	0/0	0/0	0/0	0/0	0/0	0/0	0/0	0/0	0/0	0/0	0/0	0/0	0/0	0/0
1/1	0/0	1/1	1/1	1/1	0/0	0/0	1/1	0/0	0/0	0/0	1/1	0/0	0/0	0/0	0/0	0/1	1/1	1/1	0/0	0/0	0/0	0/0	0/0	0/0	1/1	0/1
0/0	0/0	0/0	0/0	0/0	0/0	0/0	0/0	0/0	0/0	0/0	0/0	0/0	0/0	0/0	0/0	0/0	0/0	0/0	0/0	0/0	0/0	0/0	0/0	0/0	0/0	0/0
1/1	0/0	1/1	1/1	1/1	0/0	0/0	1/1	1/1	1/1	0/1	1/1	0/0	0/0	0/0	0/0	1/1	1/1	1/1	0/0	0/0	1/1	1/1	1/1	1/1	1/1	0/1
0/0	0/0	0/0	0/0	0/0	0/0	0/0	0/0	0/0	0/0	0/0	0/0	0/0	0/0	0/0	0/0	0/0	0/0	0/0	0/0	0/0	0/0	0/0	0/0	0/0	0/0	0/0
1/1	0/0	1/1	1/1	1/1	0/0	0/0	1/1	1/1	1/1	0/1	1/1	0/0	0/0	0/0	0/0	1/1	1/1	1/1	0/0	0/0	1/1	1/1	1/1	1/1	1/1	0/1

0/0	0/0	0/0	0/0	0/0	0/0	0/0	0/0	0/0	0/0	0/0	0/0	0/0	0/0	0/0	0/0	0/0	0/0	0/0	0/0	0/0	0/0	0/0	0/0	0/0	0/0	0/0
0/0	0/0	0/0	0/0	0/0	0/0	0/0	0/0	0/0	0/0	0/0	0/0	0/0	0/0	0/0	0/0	0/0	0/0	0/0	0/0	0/0	0/0	0/0	0/0	0/0	0/0	0/0
0/0	0/0	0/0	0/0	0/0	0/0	0/0	0/0	0/0	0/0	0/0	0/0	0/0	0/0	0/0	0/0	0/0	0/0	0/0	0/0	0/0	0/0	0/0	0/0	0/0	0/0	0/0
0/0	0/0	1/1	1/1	1/1	0/0	0/0	0/1	0/0	0/0	0/0	1/1	0/0	0/0	0/0	0/0	0/0	1/1	1/1	0/0	0/0	0/0	0/0	0/0	0/0	0/0	0/1
1/1	0/0	1/1	1/1	1/1	0/0	0/0	0/1	0/0	0/0	0/0	1/1	0/0	0/0	0/0	0/0	1/1	1/1	1/1	0/0	0/0	0/0	0/0	0/0	0/0	1/1	0/1
1/1	0/0	1/1	1/1	1/1	0/0	0/0	1/1	1/1	1/1	0/0	1/1	0/0	0/0	0/0	0/0	1/1	1/1	1/1	0/0	0/0	1/1	1/1	1/1	1/1	1/1	0/1
1/1	0/0	1/1	1/1	1/1	0/0	0/0	0/1	0/0	0/0	0/0	1/1	0/0	0/0	0/0	0/0	1/1	1/1	1/1	0/0	0/0	0/0	0/0	0/0	0/0	1/1	0/1
1/1	0/0	0/0	0/0	0/0	0/0	0/0	0/0	0/0	0/0	0/0	0/0	0/0	0/0	0/0	0/0	1/1	0/0	0/0	0/0	0/0	0/0	0/0	0/0	0/0	1/1	0/0
0/0	0/0	0/0	0/0	0/0	0/0	0/0	0/0	0/0	0/0	0/0	0/0	0/0	0/0	0/0	0/0	0/0	0/0	0/0	0/0	0/0	0/0	0/0	0/0	0/0	0/0	0/0
0/0	0/0	0/0	0/0	0/0	0/0	0/0	0/0	0/0	0/0	0/0	0/0	0/0	0/0	0/0	0/0	0/0	0/0	0/0	0/0	0/0	0/0	0/0	0/0	0/0	0/0	0/0
0/0	0/0	0/0	0/0	0/0	0/0	0/0	0/0	0/0	0/0	0/0	0/0	0/0	0/0	0/0	0/0	0/0	0/0	0/0	0/0	0/0	0/0	0/0	0/0	0/0	0/0	0/0
0/0	0/0	0/0	0/0	0/0	0/0	0/0	0/1	1/1	1/1	0/1	0/0	0/0	0/0	0/0	0/0	0/0	0/0	0/0	0/0	0/0	1/1	1/1	1/1	1/1	0/0	0/1
0/0	0/0	1/1	1/1	1/1	0/0	0/0	1/1	1/1	0/0	0/0	1/1	0/0	0/0	0/2	0/0	0/0	1/1	1/1	0/0	0/0	0/0	1/1	1/1	1/2	0/0	0/1
1/1	0/0	1/1	1/1	1/1	0/0	0/0	1/1	1/1	1/1	0/1	1/1	0/0	0/0	0/0	0/0	1/1	1/1	1/1	0/0	0/0	1/1	1/1	1/1	1/1	1/1	0/1
1/1	1/1	1/1	1/1	1/1	0/0	1/1	1/1	1/1	1/1	1/1	1/1	1/1	1/1	1/1	1/1	1/1	1/1	1/1	1/1	1/1	1/1	1/1	1/1	1/1	1/1	1/1
0/0	0/0	0/0	1/1	1/1	0/0	0/0	0/0	0/0	0/0	0/0	0/0	0/0	0/0	0/0	0/0	0/0	0/0	0/0	0/0	0/0	0/0	0/0	0/0	0/0	0/0	0/0
0/0	0/0	1/1	1/1	1/1	0/0	0/0	1/1	0/0	0/0	0/0	1/1	0/0	0/0	0/0	0/0	0/0	1/1	1/1	0/0	0/0	0/0	0/0	0/0	0/0	0/0	0/1
1/1	0/0	1/1	1/1	1/1	0/0	0/0	1/1	0/0	0/0	0/0	1/1	0/0	0/0	0/0	0/0	1/1	1/1	1/1	0/0	0/0	0/0	0/0	0/0	0/0	1/1	0/1
1/1	0/0	1/1	1/1	1/1	0/0	0/0	1/1	1/1	1/1	0/1	1/1	0/0	0/0	0/0	0/0	1/1	1/1	1/1	0/0	0/0	1/1	1/1	1/1	1/1	1/1	0/1
0/0	0/0	0/0	0/0	0/0	0/0	0/0	0/0	1/1	0/0	0/0	0/0	0/0	0/0	0/0	0/0	0/0	0/0	0/0	0/0	0/0	0/0	0/0	0/0	0/0	0/0	0/0
0/0	0/0	0/0	0/0	0/0	0/0	0/0	0/0	0/0	0/0	0/0	0/0	0/0	0/0	0/0	0/0	0/0	0/0	0/0	0/0	0/0	0/0	0/0	0/0	0/0	0/0	0/0
0/0	0/0	0/0	0/0	0/0	0/0	0/0	0/0	0/0	0/0	0/0	0/0	0/0	0/0	0/0	0/0	0/0	0/0	0/0	0/0	0/0	0/0	0/0	0/0	0/0	0/0	0/0
1/1	0/0	1/1	1/1	1/1	0/0	0/0	1/1	1/1	1/1	0/1	1/1	0/0	0/0	0/0	0/0	1/1	1/1	1/1	0/0	0/0	1/1	1/1	1/1	1/1	1/1	0/1
0/0	0/0	0/0	0/0	0/0	0/0	0/0	0/0	0/0	0/0	0/0	0/0	0/0	0/0	0/0	0/0	0/0	0/0	0/0	0/0	0/0	0/0	0/0	0/0	0/0	0/0	0/0
0/0	0/0	0/0	0/0	0/0	0/0	0/0	0/0	0/0	0/0	0/0	0/0	0/0	0/0	0/0	0/0	0/0	0/0	0/0	0/0	0/0	0/0	0/0	0/0	0/0	0/0	0/0
0/0	0/0	0/0	0/0	0/0	0/0	0/0	0/0	0/0	0/0	0/0	0/0	0/0	0/0	0/0	0/0	0/0	0/0	0/0	0/0	0/0	0/0	0/0	0/0	0/0	0/0	0/0
0/0	0/0	0/0	0/0	0/0	0/0	0/0	0/0	0/0	0/0	0/0	0/0	0/0	0/0	0/0	0/0	0/0	0/0	0/0	0/0	0/0	0/0	0/0	0/0	0/0	0/0	0/0
0/0	0/0	0/0	0/0	0/0	0/0	0/0	0/0	0/0	0/0	0/0	0/0	0/0	0/0	0/0	0/0	0/0	0/0	0/0	0/0	0/0	0/0	0/0	0/0	0/0	0/0	0/0
0/0	0/0	0/0	0/0	0/0	0/0	0/0	0/0	0/0	0/0	0/0	0/0	0/0	0/0	0/0	0/0	0/0	0/0	0/0	0/0	0/0	0/0	0/0	0/0	0/0	0/0	0/0
0/0	0/0	0/0	0/0	0/0	0/0	0/0	0/0	0/0	0/0	0/0	0/0	0/0	0/0	0/0	0/0	0/0	0/0	0/0	0/0	0/0	0/0	0/0	0/0	0/0	0/0	0/0
0/0	0/0	0/0	0/0	0/0	0/0	0/0	0/0	0/0	0/0	0/0	0/0	0/0	0/0	0/0	0/0	0/0	0/0	0/0	0/0	0/0	0/0	0/0	0/0	0/0	0/0	0/0
0/0	0/0	0/0	0/0	0/0	0/0	0/0	0/0	1/1	1/1	0/0	0/0	0/0	0/0	0/0	0/0	0/0	0/0	0/0	0/0	0/0	1/1	1/1	0/0	1/1	0/0	0/0
0/0	0/0	0/0	0/0	0/0	0/0	0/0	0/0	0/0	0/0	0/0	0/0	0/0	0/0	0/0	0/0	0/0	0/0	0/0	0/0	0/0	0/0	0/0	0/0	0/0	0/0	0/0
0/0	0/0	0/0	0/0	0/0	0/0	0/0	0/0	0/0	0/0	0/0	0/0	0/0	0/0	0/0	0/0	0/0	0/0	0/0	0/0	0/0	0/0	0/0	0/0	0/0	0/0	0/0
0/0	0/0	0/0	0/0	0/0	0/0	0/0	1/1	0/0	0/0	0/0	1/1	0/0	0/0	0/0	0/0	0/0	0/0	1/1	0/0	0/0	0/0	0/0	0/0	0/0	0/0	0/1

1/1	0/0	0/0	0/0	0/0	0/0	0/0	0/0	0/0	0/0	0/0	0/0	0/0	0/0	0/0	0/0	0/1	0/0	0/0	0/0	0/0	0/0	0/0	0/0	0/0	1/1	0/0
4/4	3/3	2/3	2/2	1/2	4/4	3/5	2/2	1/1	1/1	3/3	2/3	2/3	3/5	2/3	2/3	4/4	1/2	2/2	3/3	2/2	1/1	2/2	1/1	1/1	4/4	1/2
./.	./.	./.	./.	./.	./.	./.	./.	./.	./.	./.	./.	./.	./.	./.	./.	./.	./.	./.	./.	./.	./.	./.	./.	./.	./.	./.
0/0	0/0	0/0	0/0	0/0	0/0	0/0	0/0	0/0	0/0	0/0	0/0	0/0	0/0	0/0	0/0	0/0	0/0	0/0	0/0	0/0	0/0	0/0	0/0	0/0	0/0	0/0
0/0	0/0	0/0	0/0	0/0	0/0	0/0	0/0	0/0	0/0	0/0	0/0	0/0	0/0	0/0	0/0	0/0	0/0	0/0	0/0	0/0	0/0	0/0	0/0	0/0	0/0	0/0
0/0	0/0	0/0	0/0	0/0	0/0	0/0	0/0	0/0	0/0	0/0	0/0	0/0	0/0	0/0	0/0	0/0	0/0	0/0	0/0	0/0	0/0	0/0	0/0	0/0	0/0	0/0
1/1	0/0	0/0	0/0	0/0	0/0	0/0	0/0	0/0	0/0	0/0	0/0	0/0	0/0	0/0	0/0	1/1	0/0	0/0	0/0	0/0	0/0	0/0	0/0	0/0	1/1	0/0
1/1	0/0	0/0	0/0	./.	0/0	0/0	./.	0/0	0/0	0/0	0/0	0/0	0/0	0/0	0/0	0/1	0/0	./.	0/0	0/0	0/0	0/0	./.	0/0	0/1	./.
./.	./.	0/0	0/0	./.	0/0	0/0	./.	0/0	0/0	0/0	./.	0/0	./.	0/0	0/0	0/0	0/0	./.	0/0	./.	0/0	0/0	./.	0/0	0/0	./.
1/1	./.	1/1	1/1	1/1	0/0	./.	1/1	1/1	./.	./.	1/1	0/0	./.	./.	0/0	0/1	1/1	1/1	./.	./.	0/1	./.	./.	0/0	0/1	0/1
./.	1/1	./.	0/0	./.	0/0	1/1	0/0	./.	2/2	1/2	./.	1/1	1/1	1/1	1/1	0/2	./.	./.	1/1	1/1	0/2	./.	2/2	2/2	0/2	0/2
0/0	2/2	0/0	0/0	0/0	0/0	2/2	0/0	./.	./.	2/2	0/0	2/2	2/2	2/2	2/2	0/0	0/0	0/0	2/2	2/2	./.	./.	./.	./.	0/0	0/2
0/0	0/0	0/0	0/0	0/0	0/0	0/0	0/0	0/0	0/0	0/0	0/0	0/0	0/0	0/0	0/0	0/0	0/0	0/0	0/0	0/0	0/0	0/0	0/0	0/0	0/0	0/0
0/0	0/0	0/0	0/0	0/0	0/0	0/0	0/0	0/0	0/0	0/0	0/0	0/0	0/0	0/0	0/0	0/0	0/0	0/0	0/0	0/0	0/0	0/0	0/0	0/0	0/0	0/0
0/0	0/0	0/0	0/0	0/0	0/0	0/0	0/0	0/0	0/0	0/0	0/0	0/0	0/0	0/0	0/0	0/0	0/0	0/0	0/0	0/0	0/0	0/0	0/0	0/0	0/0	0/0
1/1	0/0	1/1	1/1	1/1	0/0	0/0	1/1	1/1	1/1	0/1	1/1	0/0	0/0	0/0	0/0	1/1	1/1	1/1	0/0	0/0	1/1	./.	1/1	1/1	1/1	0/1
0/0	0/0	0/0	0/0	0/0	0/0	0/0	0/0	0/0	0/0	0/0	0/0	0/0	0/0	0/0	0/0	0/0	0/0	0/0	0/0	0/0	0/0	./.	0/0	0/0	0/0	0/0
0/0	0/0	1/1	1/1	1/1	0/0	0/0	1/1	0/0	0/0	0/0	1/1	0/0	0/0	0/0	0/0	0/0	1/1	1/1	0/0	0/0	0/0	./.	0/0	0/0	0/0	0/1
0/0	0/0	0/0	0/0	0/0	0/0	0/0	0/0	0/0	0/0	0/0	0/0	0/0	0/0	0/0	0/0	0/0	0/0	0/0	0/0	0/0	0/0	./.	0/0	0/0	0/0	0/0
0/0	0/0	0/0	0/0	0/0	0/0	0/0	0/0	0/0	0/0	0/0	0/0	0/0	0/0	0/0	0/0	0/0	0/0	0/0	0/0	0/0	0/0	./.	1/1	0/0	0/0	0/1
0/0	0/0	0/0	0/0	0/0	0/0	0/0	0/0	0/0	0/0	0/0	0/0	0/0	0/0	0/0	0/0	0/0	0/0	0/0	0/0	0/0	0/0	./.	0/0	0/0	0/0	0/0

0/0	0/0	0/0	0/0	0/0	0/0	0/0	0/0	0/0	0/0	0/0	0/0	0/0	0/0	0/0	0/0	0/0	0/0	0/0	0/0	0/0	0/0	./.	0/0	0/0	0/0	0/0
0/0	0/0	0/0	0/0	0/0	0/0	0/0	0/0	0/0	0/0	0/0	0/0	0/0	0/0	0/0	0/0	0/0	0/0	0/0	0/0	0/0	0/0	./.	0/0	0/0	0/0	0/0
1/1	0/0	1/1	1/1	1/1	0/0	0/0	1/1	2/2	2/2	0/0	2/2	0/0	0/0	0/0	0/0	1/2	1/1	1/1	0/0	0/0	2/2	./.	1/1	2/2	1/2	0/1
0/0	0/0	0/0	0/0	0/0	0/0	0/0	0/0	0/0	0/0	0/0	0/0	0/0	0/0	0/0	0/0	0/0	0/0	0/0	0/0	0/0	0/0	./.	0/0	0/0	0/0	0/0
0/0	0/0	1/1	1/1	1/1	0/0	0/0	0/1	2/2	2/2	0/0	2/2	0/0	0/0	0/0	0/0	0/2	1/1	1/1	0/0	0/0	2/2	2/2	1/1	2/2	0/2	0/1
0/0	0/0	0/0	0/0	0/0	0/0	0/0	0/0	0/0	0/0	0/0	0/0	0/0	0/0	0/0	0/0	0/0	0/0	0/0	0/0	0/0	0/0	0/0	0/0	0/0	0/0	0/0
1/1	0/0	0/0	0/0	0/0	0/0	0/0	0/0	0/0	0/0	0/0	0/0	0/0	0/0	0/0	0/0	0/1	0/0	0/0	0/0	0/0	0/0	0/0	0/0	0/0	0/0	0/0
1/1	0/0	1/1	1/1	1/1	0/0	0/0	1/1	1/1	1/1	0/0	1/1	0/0	0/0	0/0	0/0	1/1	1/1	1/1	0/0	0/0	1/1	1/1	1/1	1/1	1/1	0/1
1/1	0/0	1/1	1/1	1/1	0/0	0/0	1/1	1/1	1/1	0/0	1/1	0/0	0/0	0/0	0/0	1/1	1/1	1/1	0/0	0/0	1/1	1/1	1/1	1/1	1/1	0/1
0/0	0/0	0/0	0/0	0/0	0/0	0/0	0/0	0/0	0/0	0/0	0/0	0/0	0/0	0/0	0/0	0/0	0/0	0/0	0/0	0/0	0/0	0/0	0/0	0/0	0/0	0/0
2/2	0/0	1/1	1/1	1/1	0/0	0/0	1/1	2/2	2/2	0/0	2/2	0/0	0/0	0/0	0/0	2/2	1/1	1/1	0/0	0/0	2/2	2/2	0/2	2/2	2/2	0/2
0/0	0/0	0/0	0/0	0/0	0/0	0/0	0/0	0/0	0/0	0/0	0/0	0/0	0/0	0/0	0/0	0/0	0/0	0/0	0/0	0/0	0/0	0/0	0/0	0/0	0/0	0/0
0/0	0/0	0/0	0/0	0/0	0/0	0/0	0/0	0/0	0/0	0/0	0/0	0/0	0/0	0/0	0/0	0/0	0/0	0/0	0/0	0/0	0/0	0/0	0/0	0/0	0/0	0/0
0/0	0/0	0/0	0/0	0/0	0/0	0/0	0/0	0/0	0/0	0/0	0/0	0/0	0/0	0/0	0/0	0/0	0/0	0/0	0/0	0/0	0/0	0/0	0/0	0/0	0/0	0/0
1/1	0/0	0/0	0/0	0/0	0/0	0/0	0/0	0/0	0/0	0/0	0/0	0/0	0/0	0/0	0/0	0/1	0/0	0/0	0/0	0/0	0/0	0/0	0/0	0/0	1/1	0/0
0/0	0/0	0/0	0/0	0/0	0/0	0/0	0/0	0/0	0/0	0/0	0/0	0/0	0/0	0/0	0/0	0/0	0/0	0/0	0/0	0/0	0/0	0/0	0/0	0/0	0/0	0/0
1/1	0/0	0/0	0/0	0/0	0/0	0/0	0/0	0/0	0/0	0/0	0/0	0/0	0/0	0/0	0/0	0/1	0/0	0/0	0/0	0/0	0/0	0/0	0/0	0/0	1/1	0/0
1/1	0/0	1/1	1/1	1/1	0/0	0/0	1/1	1/1	1/1	0/0	1/1	0/0	0/0	0/0	0/0	1/1	1/1	1/1	0/0	0/0	1/1	1/1	1/1	1/1	1/1	0/1

981	989	993	999	1025	1056	1057	1058	1061	1069	1089	1098	1100	1128	1143	1170	1203	1235	1251	1255	1257	1261	1262	1264	1267	1268	1281
0/0	0/0	0/0	0/0	0/0	0/0	0/0	0/0	0/0	0/0	0/0	0/0	0/0	0/0	0/0	0/0	0/0	0/0	0/0	0/0	0/0	0/0	0/0	0/0	0/0	0/0	0/0
0/0	1/1	0/0	0/0	0/0	0/0	0/0	1/1	0/0	0/0	1/1	0/0	0/0	0/0	0/0	1/1	0/0	1/1	0/0	0/0	0/0	0/0	0/0	0/0	0/0	1/1	0/0
0/0	0/0	0/0	0/0	0/0	0/0	0/0	0/0	0/0	0/0	0/0	0/0	0/0	0/0	0/0	0/0	0/0	0/0	0/0	0/0	0/0	0/0	0/0	0/0	0/0	0/0	0/0
0/0	0/0	0/0	0/0	0/0	0/0	0/0	0/0	0/0	0/0	0/0	0/0	0/0	0/0	1/1	0/0	0/0	0/0	0/0	0/0	0/0	0/0	0/0	0/0	0/0	0/0	0/0
0/0	0/0	0/0	1/1	0/0	0/0	0/0	0/0	0/0	0/0	0/0	0/0	0/0	1/1	1/1	0/0	1/1	0/0	0/0	0/0	0/0	0/0	0/0	0/0	0/0	0/0	0/0
0/0	0/0	0/0	1/1	0/0	0/0	0/0	0/0	0/0	0/0	0/0	0/0	0/0	1/1	1/1	0/0	1/1	0/0	0/0	0/0	0/0	0/0	0/0	0/0	0/0	0/0	0/0
0/0	0/0	0/0	0/0	0/0	0/0	0/0	0/0	0/0	0/0	0/0	0/0	0/0	0/0	0/0	0/0	0/0	0/0	0/0	0/0	0/0	0/0	0/0	0/0	0/0	0/0	0/0
0/0	1/1	1/1	0/0	0/0	0/0	0/0	1/1	0/0	0/0	1/1	0/0	0/0	0/0	0/0	1/1	0/0	1/1	0/0	0/1	0/0	0/0	0/0	0/0	0/0	1/1	0/0
0/0	0/0	0/0	0/0	0/0	0/0	0/0	0/0	0/0	0/0	0/0	0/0	0/0	0/0	0/0	0/0	0/0	0/0	0/0	0/0	0/0	1/1	0/0	0/0	0/0	0/0	0/0
0/0	0/0	0/0	0/0	0/0	0/0	0/0	0/0	0/0	0/0	0/0	0/0	0/0	0/0	0/0	0/0	0/0	0/0	0/0	0/0	0/0	0/0	0/0	0/0	0/0	0/0	0/0
0/1	0/0	0/0	0/0	0/0	0/0	0/0	0/0	0/0	0/0	0/0	0/0	0/0	0/0	0/0	0/0	0/0	0/0	0/0	0/0	0/0	0/0	0/0	0/0	0/0	0/0	0/0
0/0	0/0	0/0	0/0	0/0	0/0	0/0	0/0	0/0	0/0	0/0	0/0	0/0	0/0	0/0	0/0	0/0	0/0	0/0	0/0	0/0	0/0	0/0	0/0	0/0	0/0	0/0
0/0	0/0	0/0	1/1	0/0	0/0	0/0	0/0	0/0	0/0	0/0	0/0	0/0	1/1	1/1	0/0	1/1	0/0	0/0	0/0	0/0	0/0	0/0	0/0	0/0	0/0	0/0
0/0	0/0	0/0	0/0	0/0	0/0	0/0	0/1	0/0	0/0	0/0	0/0	0/0	0/0	0/0	0/0	0/0	0/1	0/0	0/0	0/0	0/0	0/0	0/0	0/0	0/0	0/0
0/0	1/1	1/1	./.	0/0	0/0	0/0	0/1	0/0	./.	1/1	0/0	0/0	0/0	0/0	1/1	./.	0/1	0/0	0/1	0/0	./.	0/0	0/0	./.	1/1	0/0
0/0	0/0	0/0	./.	0/0	0/0	0/0	0/0	0/0	./.	0/0	0/0	0/0	0/0	0/0	0/0	./.	0/0	0/0	0/0	0/0	./.	0/0	0/0	./.	0/0	0/0

4/4	./.	0/4	3/3	2/3	4/4	4/4	3/5	0/0	4/4	1/1	0/0	0/0	2/3	0/6	3/3	./.	1/5	0/0	2/3	1/3	1/1	0/1	1/1	3/6	3/3	4/4
0/0	0/0	0/0	0/0	0/0	0/0	0/0	0/0	1/1	0/0	0/0	0/0	1/1	0/0	0/0	0/0	0/0	0/0	0/0	0/0	0/0	0/0	0/0	0/0	0/0	0/0	0/0
0/0	0/0	0/0	./.	0/0	0/0	0/0	0/0	0/0	0/0	0/0	0/0	0/0	0/0	0/0	0/0	./.	0/0	0/0	0/0	0/0	0/0	0/0	0/0	0/0	0/0	0/0
0/0	0/0	0/0	1/1	0/0	0/0	0/0	0/0	0/0	0/0	0/0	0/0	0/0	1/1	0/0	0/0	1/1	0/0	0/0	0/0	0/0	0/0	0/0	0/0	0/0	0/0	0/0
0/0	1/1	1/1	0/0	0/0	0/0	0/0	1/1	0/0	0/0	1/1	0/0	0/0	0/0	0/0	1/1	0/0	1/1	0/0	0/1	0/0	0/0	0/0	0/1	0/0	1/1	0/0
0/0	0/0	0/0	0/0	0/0	0/0	0/0	0/0	0/0	0/0	0/0	0/0	0/0	0/0	1/1	0/0	0/0	0/0	0/0	0/0	0/0	0/0	0/0	0/0	0/0	0/0	0/0
0/0	1/1	1/1	0/0	0/0	0/0	0/0	1/1	0/0	0/0	1/1	0/0	0/0	0/0	0/0	1/1	0/0	1/1	0/0	0/1	0/0	0/0	0/0	0/1	0/0	1/1	0/0
0/0	0/0	0/0	1/1	0/0	0/0	0/0	0/0	0/0	0/0	0/0	0/0	0/0	1/1	0/0	0/0	1/1	0/0	0/0	0/0	0/0	0/0	0/0	0/0	0/0	0/0	0/0
0/0	0/0	0/0	0/0	0/0	0/0	0/0	0/0	0/0	0/0	0/0	0/0	0/0	0/0	0/0	0/0	0/0	0/0	0/0	0/1	0/1	1/1	1/1	1/1	1/1	0/0	0/0
0/0	0/0	1/1	1/1	0/0	0/0	0/0	0/0	0/0	0/0	0/0	0/0	0/0	1/1	1/1	0/0	1/1	0/0	0/0	0/1	0/2	2/2	2/2	2/2	2/2	0/0	0/0
0/0	0/0	0/0	0/0	0/0	0/0	0/0	0/0	0/0	0/0	0/0	0/0	0/0	0/0	0/0	0/0	0/0	0/0	0/0	./.	0/0	./.	./.	./.	./.	0/0	0/0
0/0	0/0	0/0	1/1	0/0	0/0	0/0	0/0	0/0	0/0	0/0	0/0	0/0	1/1	0/0	0/0	1/1	0/0	0/0	0/0	0/0	0/0	0/0	0/0	0/0	0/0	0/0
0/0	1/1	1/1	1/1	0/0	0/0	0/0	1/1	0/0	0/0	1/1	0/0	0/0	1/1	1/1	1/1	1/1	1/1	0/0	0/1	0/0	0/0	0/0	0/1	0/0	1/1	0/0
0/0	0/0	0/0	0/0	0/0	0/0	0/0	0/0	0/0	0/0	0/0	0/0	0/0	0/0	1/1	0/0	0/0	0/0	0/0	0/0	0/0	0/0	0/0	0/0	0/0	0/0	0/0
0/0	0/0	0/0	0/0	0/0	0/0	0/0	0/0	0/0	0/0	0/0	0/0	0/0	0/0	0/0	0/0	0/0	0/0	0/0	0/0	0/0	0/0	0/0	0/0	0/0	0/0	0/0
0/0	1/1	1/1	1/1	0/0	0/0	0/0	1/1	0/0	0/0	1/1	0/0	0/0	1/1	1/1	1/1	1/1	1/1	0/0	0/1	0/0	0/0	0/0	0/1	0/0	1/1	0/0
0/0	0/0	0/0	1/1	0/0	0/0	0/0	0/0	0/0	0/0	0/0	0/0	0/0	1/1	0/0	0/0	1/1	0/0	0/0	0/0	0/0	0/0	0/0	0/0	0/0	0/0	0/0
0/0	0/0	0/0	0/0	0/0	0/0	0/0	0/0	0/0	0/0	0/0	0/0	0/0	0/0	1/1	0/0	0/0	0/0	0/0	0/0	0/0	0/0	0/0	0/0	0/0	0/0	0/0
0/0	1/1	1/1	0/0	0/0	0/0	0/0	1/1	0/0	0/0	1/1	0/0	0/0	0/0	0/0	1/1	0/0	1/1	0/0	0/1	0/0	0/0	0/0	0/0	0/0	1/1	0/0
0/0	1/1	0/0	0/0	0/0	0/0	0/0	1/1	0/0	0/0	1/1	0/0	0/0	0/0	0/0	1/1	0/0	1/1	0/0	0/0	0/0	0/0	0/0	0/0	0/0	1/1	0/0
0/0	0/0	0/0	0/0	0/0	0/0	0/0	0/0	0/0	0/0	0/0	0/0	0/0	0/0	1/1	0/0	0/0	0/0	0/0	0/0	0/0	0/0	0/0	0/0	0/0	0/0	0/0
0/0	1/1	1/1	0/0	0/0	0/0	0/0	1/1	0/0	0/0	1/1	0/0	0/0	0/0	0/0	1/1	0/0	1/1	0/0	0/1	0/0	0/0	0/0	0/1	0/0	1/1	0/0
0/0	0/0	0/0	0/0	0/0	0/0	0/0	0/0	0/0	0/0	0/0	0/0	0/0	0/0	1/1	0/0	0/0	0/0	0/0	0/0	0/0	0/0	0/0	0/0	0/0	0/0	0/0
0/0	1/1	1/1	1/1	0/0	0/0	0/0	1/1	0/0	0/0	1/1	0/0	0/0	1/1	1/1	1/1	1/1	1/1	0/0	0/1	0/0	0/0	0/0	0/0	0/0	1/1	0/0
0/0	0/0	0/0	1/1	0/0	0/0	0/0	0/0	0/0	0/0	0/0	0/0	0/0	1/1	0/0	0/0	1/1	0/0	0/0	0/0	0/0	0/0	0/0	0/0	0/0	0/0	0/0
0/0	1/1	0/0	0/0	0/0	0/0	0/0	1/1	0/0	0/0	1/1	0/0	0/0	0/0	2/2	1/2	0/0	1/1	0/0	0/0	0/0	0/0	0/0	0/0	0/0	1/1	0/0

1/1	1/1	1/1	1/1	0/0	1/1	1/1	1/1	0/0	1/1	1/1	0/0	0/0	1/1	1/1	1/1	1/1	1/1	0/0	1/1	0/1	1/1	1/1	1/1	1/1	1/1	1/1
0/0	0/0	0/0	1/1	0/0	0/0	0/0	0/0	0/0	0/0	0/0	0/0	0/0	1/1	1/1	0/0	1/1	0/0	0/0	0/0	0/0	0/0	0/0	0/0	0/0	0/0	0/0
0/0	0/0	0/0	1/1	0/0	0/0	0/0	0/0	0/0	0/0	0/0	0/0	0/0	1/1	0/0	0/0	1/1	0/0	0/0	0/0	0/0	0/0	0/0	0/0	0/0	0/0	0/0
0/0	0/0	0/0	0/0	0/0	0/0	0/0	0/0	0/0	0/0	0/0	0/0	0/0	0/0	0/0	0/0	0/0	0/0	0/0	0/0	0/0	0/0	0/0	0/0	0/0	0/0	0/0
0/0	1/1	1/1	1/1	0/0	0/0	0/0	1/1	0/0	0/0	1/1	0/0	0/0	1/1	1/1	1/1	1/1	1/1	0/0	0/1	0/0	0/0	0/0	0/0	0/0	1/1	0/0
0/0	1/1	1/1	1/1	0/0	0/0	0/0	1/1	0/0	0/0	1/1	0/0	0/0	1/1	1/1	1/1	1/1	1/1	0/0	0/1	0/0	0/0	0/0	0/0	0/0	1/1	0/0
0/0	1/1	1/1	1/1	0/0	0/0	0/0	1/1	0/0	0/0	1/1	0/0	0/0	1/1	1/1	1/1	1/1	1/1	0/0	0/1	0/0	0/0	0/0	0/0	0/0	1/1	0/0
0/0	0/0	0/0	./.	0/0	0/0	0/0	0/0	0/0	0/0	0/0	0/0	0/0	0/0	1/1	0/0	0/0	0/0	0/0	0/0	0/0	0/0	0/0	0/0	0/0	0/0	0/0
0/0	0/0	0/0	1/1	0/0	0/0	0/0	0/0	0/0	0/0	0/0	0/0	0/0	1/1	0/0	0/0	1/1	0/0	0/0	0/0	0/0	0/0	0/0	0/0	0/0	0/0	0/0
0/0	0/0	0/0	0/0	0/0	0/0	0/0	0/0	1/1	0/0	0/0	0/0	1/1	0/0	0/0	0/0	0/0	0/0	0/0	0/0	0/0	0/0	0/0	0/0	0/0	0/0	0/0
0/0	1/1	1/1	0/0	0/0	0/0	0/0	1/1	0/0	0/0	1/1	0/0	0/0	0/0	0/0	1/1	0/0	1/1	0/0	0/1	0/0	0/0	0/0	0/0	0/0	1/1	0/0
0/0	0/0	0/0	0/0	0/0	0/0	0/0	0/0	0/0	0/0	0/0	0/0	0/0	0/0	0/0	0/0	0/0	0/0	0/0	0/0	0/0	0/0	0/0	0/0	0/0	0/0	0/0
0/0	0/0	0/0	0/0	0/0	0/0	0/0	0/0	0/0	0/0	0/0	0/0	0/0	0/0	0/0	0/0	0/0	0/0	0/0	0/0	0/0	0/0	0/0	0/0	0/0	0/0	0/0
0/0	0/0	1/1	0/0	0/0	0/0	0/0	0/0	0/0	0/0	0/0	0/0	0/0	0/0	0/0	0/0	0/0	0/0	0/0	0/1	0/0	0/0	0/0	0/0	0/0	0/0	0/0
0/0	1/1	1/1	1/1	0/0	0/0	0/0	1/1	0/0	0/0	1/1	0/0	0/0	1/1	1/1	1/1	1/1	1/1	0/0	0/1	0/0	0/0	0/0	0/0	0/0	1/1	0/0
0/0	0/0	0/0	0/0	0/0	0/0	0/0	0/0	0/0	0/0	0/0	0/0	0/0	0/0	1/1	0/0	0/0	0/0	0/0	0/0	0/0	0/0	0/0	0/0	0/0	0/0	0/0
0/0	0/0	0/0	0/0	0/0	0/0	0/0	0/0	0/0	0/0	0/0	0/0	0/0	0/0	0/0	0/0	0/0	0/0	0/0	0/0	0/0	0/0	0/0	0/0	0/0	0/0	0/0
0/0	0/0	0/0	0/0	0/0	0/0	0/0	0/0	0/0	0/0	0/0	0/0	0/0	0/0	0/0	0/0	0/0	0/0	0/0	0/0	0/0	0/0	0/0	0/0	0/0	0/0	0/0
0/0	1/1	1/1	0/0	0/0	0/0	0/0	1/1	0/0	0/0	1/1	0/0	0/0	0/0	0/0	1/1	0/0	1/1	0/0	0/1	0/0	0/0	0/0	0/0	0/0	1/1	0/0
0/0	0/0	0/0	0/0	0/0	0/0	0/0	0/0	0/0	0/0	0/0	0/0	0/0	0/0	1/1	0/0	0/0	0/0	0/0	0/0	0/0	0/0	0/0	0/0	0/0	0/0	0/0
0/0	1/1	1/1	./.	0/0	0/0	0/0	1/1	0/0	0/0	1/1	0/0	0/0	1/1	1/1	1/1	1/1	1/1	0/0	0/1	0/0	0/0	0/0	0/0	0/0	1/1	0/0
0/0	1/1	1/1	0/0	0/0	0/0	0/0	1/1	0/0	0/0	1/1	0/0	0/0	0/0	0/0	1/1	0/0	1/1	0/0	0/1	0/0	0/0	0/0	0/0	0/0	1/1	0/0
0/0	1/1	1/1	0/0	0/0	0/0	0/0	1/1	0/0	0/0	1/1	0/0	0/0	0/0	0/0	1/1	0/0	1/1	0/0	0/1	0/0	0/0	0/0	0/0	0/0	1/1	0/0
0/0	0/0	0/0	1/1	0/0	0/0	0/0	0/0	0/0	0/0	0/0	0/0	0/0	1/1	1/1	0/0	1/1	0/0	0/0	0/0	0/0	0/0	0/0	0/0	0/0	0/0	0/0
0/0	1/1	1/1	1/1	0/0	0/0	0/0	1/1	0/0	0/0	1/1	0/0	0/0	1/1	1/1	1/1	1/1	1/1	0/0	0/1	0/0	0/0	0/0	0/0	0/0	1/1	0/0
0/0	0/0	0/0	1/1	0/0	0/0	0/0	0/0	0/0	0/0	0/0	0/0	0/0	1/1	1/1	0/0	1/1	0/0	0/0	0/0	0/0	0/0	0/0	0/0	0/0	0/0	0/0
0/0	0/0	0/0	0/0	0/0	0/0	0/0	0/0	0/0	0/0	0/0	0/0	0/0	0/0	0/0	0/0	0/0	0/0	0/0	0/0	0/0	0/0	0/0	0/0	0/0	0/0	0/0
0/0	0/0	0/0	0/0	0/0	0/0	0/0	0/0	0/0	0/0	0/0	0/0	0/0	0/0	1/1	0/0	0/0	0/0	0/0	0/0	0/0	0/0	0/0	0/0	0/0	0/0	0/0
1/1	1/1	1/1	1/1	0/0	1/1	1/1	1/1	0/0	1/1	1/1	0/0	0/0	1/1	1/1	1/1	1/1	1/1	0/0	1/1	0/1	1/1	1/1	1/1	1/1	1/1	1/1
0/0	0/0	0/0	0/0	0/0	0/0	0/0	0/0	0/0	0/0	0/0	0/0	0/0	0/0	0/0	0/0	0/0	0/0	0/0	0/0	0/1	0/0	0/0	0/0	0/0	0/0	0/0
0/0	1/1	1/1	1/1	0/0	0/0	0/0	1/1	0/0	0/0	1/1	0/0	0/0	1/1	1/1	1/1	1/1	1/1	0/0	0/1	0/0	0/0	0/0	0/0	0/0	1/1	0/0
0/0	0/0	0/0	1/1	0/0	0/0	0/0	0/0	0/0	0/0	0/0	0/0	0/0	1/1	0/0	0/0	1/1	0/0	0/0	0/0	0/0	0/0	0/0	0/0	0/0	0/0	0/0
0/0	1/1	1/1	1/1	0/0	0/0	0/0	1/1	0/0	0/0	1/1	0/0	0/0	1/1	1/1	1/1	1/1	1/1	0/0	0/1	0/0	0/0	0/0	0/0	0/0	1/1	0/0
0/0	0/0	0/0	0/0	0/0	0/0	0/0	0/0	0/0	0/0	0/0	0/0	0/0	0/0	0/0	0/0	0/0	0/0	0/0	0/0	0/0	0/0	0/0	0/0	0/0	0/0	0/0
0/0	0/0	0/0	1/1	0/0	0/0	0/0	0/0	0/0	0/0	0/0	0/0	0/0	1/1	1/1	0/0	1/1	0/0	0/0	0/0	0/0	0/0	0/0	0/0	0/0	0/0	0/0

0/0	1/1	1/1	0/0	0/0	0/0	0/0	1/1	0/0	0/0	1/1	0/0	0/0	0/0	0/0	1/1	0/0	1/1	0/0	0/1	0/0	0/0	0/0	0/0	0/0	1/1	0/0
0/0	0/0	0/0	0/0	0/0	0/0	0/0	0/0	0/0	0/0	0/0	0/0	0/0	0/0	0/0	0/0	0/0	0/0	0/0	0/0	0/0	0/0	0/0	0/0	0/0	0/0	0/0
0/0	1/1	0/0	0/0	0/0	0/0	0/0	1/1	0/0	0/0	1/1	0/0	0/0	0/0	0/0	1/1	0/0	1/1	0/0	0/0	0/0	0/0	0/0	0/0	0/0	1/1	0/0
0/0	1/1	1/1	./.	0/0	0/0	0/0	1/1	0/0	0/0	1/1	0/0	0/0	1/1	1/1	1/1	1/1	1/1	0/0	0/1	0/0	0/0	0/0	0/0	0/0	1/1	0/0
0/0	0/0	0/0	0/0	0/0	0/0	0/0	0/0	0/0	0/0	0/0	0/0	0/0	0/0	0/0	0/0	0/0	0/0	0/0	0/0	0/0	0/0	0/0	0/0	0/0	0/0	0/0
0/0	1/1	1/1	0/0	0/0	0/0	0/0	1/1	0/0	0/0	1/1	0/0	0/0	0/0	0/0	1/1	0/0	1/1	0/0	0/1	0/0	0/0	0/0	0/0	0/0	1/1	0/0
0/0	1/1	1/1	1/1	0/0	0/0	0/0	1/1	0/0	0/0	1/1	0/0	0/0	1/1	1/1	1/1	1/1	1/1	0/0	0/1	0/0	0/0	0/0	0/0	0/0	1/1	0/0
0/0	0/0	./.	1/1	0/0	0/0	0/0	0/0	0/0	0/0	0/0	0/0	0/0	1/1	0/0	0/0	1/1	0/0	0/0	0/0	0/0	0/0	0/0	0/0	0/0	0/0	0/0
0/0	1/1	1/1	0/0	0/0	0/0	0/0	1/1	0/0	0/0	1/1	0/0	0/0	0/0	0/0	1/1	0/0	1/1	0/0	0/1	0/0	0/0	0/0	0/0	0/0	1/1	0/0
0/0	0/0	0/0	0/0	0/0	0/0	0/0	0/0	0/0	0/0	0/0	0/0	0/0	0/0	0/0	0/0	0/0	0/0	0/0	0/0	0/0	0/0	0/0	0/0	0/0	0/0	0/0
0/0	1/1	1/1	0/0	0/0	0/0	0/0	1/1	0/0	0/0	1/1	0/0	0/0	0/0	0/0	1/1	0/0	1/1	0/0	0/1	0/0	0/0	0/0	0/0	0/0	1/1	0/0
0/0	0/0	0/0	0/0	0/0	0/0	0/0	0/0	0/0	0/0	0/0	0/0	0/0	0/0	1/1	0/0	0/0	0/0	0/0	0/0	0/0	0/0	0/0	0/0	0/0	0/0	0/0
0/0	0/0	0/0	0/0	0/0	0/0	0/0	0/0	0/0	0/0	0/0	0/0	0/0	0/0	1/1	0/0	0/0	0/0	0/0	0/0	0/0	0/0	0/0	0/0	0/0	0/0	0/0
0/0	0/0	0/0	0/0	0/0	0/0	0/0	0/0	0/0	0/0	0/0	0/0	0/0	0/0	0/0	0/0	0/0	0/0	0/0	0/0	0/0	0/0	0/0	0/0	0/0	0/0	0/0
1/1	1/1	1/1	1/1	0/0	1/1	1/1	1/1	0/0	1/1	1/1	0/0	0/0	1/1	1/1	1/1	1/1	1/1	0/0	1/1	0/1	1/1	1/1	1/1	1/1	1/1	1/1
1/1	1/1	1/1	1/1	0/0	1/1	1/1	1/1	0/0	1/1	1/1	0/0	0/0	1/1	1/1	1/1	1/1	1/1	0/0	1/1	0/1	1/1	1/1	1/1	1/1	1/1	1/1
0/0	1/1	1/1	1/1	0/0	0/0	0/0	1/1	0/0	0/0	1/1	0/0	0/0	1/1	1/1	1/1	1/1	1/1	0/0	0/1	0/0	0/0	0/0	0/0	0/0	1/1	0/0
./.	1/1	./.	./.	0/0	./.	./.	1/1	0/0	./.	1/1	0/0	./.	0/0	./.	./.	./.	1/1	0/0	./.	0/0	./.	./.	0/0	0/0	./.	./.
1/1	4/4	1/1	3/3	0/0	1/1	1/1	4/4	2/2	1/1	4/4	0/0	2/2	3/3	3/3	1/1	3/3	4/4	0/0	1/1	0/1	1/1	1/1	1/1	1/1	1/1	1/1
0/0	0/0	0/0	1/1	0/0	0/0	0/0	0/0	0/0	0/0	0/0	0/0	0/0	1/1	0/0	0/0	0/0	0/0	0/0	0/0	0/0	0/0	0/0	0/0	0/0	0/0	0/0
0/0	0/0	0/0	0/0	0/0	0/0	0/0	0/0	0/0	0/0	0/0	0/0	0/0	0/0	0/0	0/0	0/0	0/0	0/0	0/0	0/0	0/0	0/0	0/0	0/0	0/0	0/0
0/0	0/0	0/0	0/0	0/0	0/0	0/0	0/0	0/0	0/0	0/0	0/0	0/0	0/0	0/0	0/0	0/0	0/0	0/0	0/0	0/0	0/0	0/0	0/0	0/0	0/0	0/0
0/0	0/0	0/0	0/0	0/0	0/0	0/0	0/0	0/0	0/0	0/0	0/0	0/0	0/0	0/0	0/0	0/0	0/0	0/0	0/0	0/0	0/0	0/0	0/0	0/0	0/0	0/0
0/0	0/0	0/0	./.	0/0	0/0	0/0	0/0	0/0	0/0	0/0	0/0	0/0	0/0	1/1	0/0	0/0	0/0	0/0	0/0	0/0	0/0	0/0	0/0	0/0	0/0	0/0
0/0	0/0	0/0	1/1	0/0	0/0	0/0	0/0	0/0	0/0	0/0	0/0	0/0	1/1	0/0	0/0	1/1	0/0	0/0	0/0	0/0	0/0	0/0	0/0	0/0	0/0	0/0

0/0	0/0	0/0	0/0	0/0	0/0	0/0	0/0	0/0	0/0	0/0	0/0	0/0	0/0	0/0	0/0	0/0	0/0	0/0	0/0	0/0	0/0	0/0	0/0	0/0	0/0	0/0
0/0	0/0	0/0	1/1	0/0	0/0	0/0	0/0	0/0	0/0	0/0	0/0	0/0	1/1	0/0	0/0	1/1	0/0	0/0	0/0	0/0	0/0	0/0	0/0	0/0	0/0	0/0
0/0	0/0	0/0	1/1	0/0	0/0	0/0	0/0	0/0	0/0	0/0	0/0	0/0	1/1	0/0	0/0	1/1	0/0	0/0	0/0	0/0	0/0	0/0	0/0	0/0	0/0	0/0
1/1	1/1	1/1	1/1	0/0	1/1	1/1	1/1	0/0	1/1	1/1	0/0	0/0	1/1	1/1	1/1	1/1	1/1	0/0	1/1	0/1	1/1	1/1	1/1	1/1	1/1	1/1
0/0	1/1	1/1	1/1	0/0	0/0	0/0	1/1	0/0	0/0	1/1	0/0	0/0	1/1	1/1	1/1	1/1	1/1	0/0	0/1	0/0	0/0	0/0	0/0	0/0	1/1	0/0
0/0	0/0	0/0	0/0	0/0	0/0	0/0	0/0	0/0	0/0	0/0	0/0	0/0	0/0	0/0	0/0	0/0	0/0	0/0	0/0	0/0	0/0	0/0	0/0	0/0	0/0	0/0
0/0	1/1	1/1	1/1	0/0	0/0	0/0	1/1	0/0	0/0	1/1	0/0	0/0	1/1	1/1	1/1	1/1	1/1	0/0	0/1	0/0	0/0	0/0	0/0	0/0	1/1	0/0
0/0	0/0	0/0	1/1	0/0	0/0	0/0	0/0	0/0	0/0	0/0	0/0	0/0	1/1	0/0	0/0	1/1	0/0	0/0	0/0	0/0	0/0	0/0	0/0	0/0	0/0	0/0
0/0	0/0	0/0	0/0	0/0	0/0	0/0	0/0	0/0	0/0	0/0	0/0	0/0	0/0	1/1	0/0	0/0	0/0	0/0	0/0	0/0	0/0	0/0	0/0	0/0	0/0	0/0
0/0	0/0	0/0	0/0	0/0	0/0	0/0	0/0	0/0	0/0	0/0	0/0	0/0	0/0	0/0	0/0	0/0	0/0	0/0	0/0	0/0	0/0	0/0	0/0	0/0	0/0	0/0
0/0	0/0	0/0	1/1	0/0	0/0	0/0	0/0	0/0	0/0	0/0	0/0	0/0	1/1	0/0	0/0	1/1	0/0	0/0	0/0	0/0	0/0	0/0	0/0	0/0	0/0	0/0
0/0	0/0	0/0	0/0	0/0	0/0	0/0	0/0	0/0	0/0	0/0	0/0	0/0	0/0	0/0	0/0	0/0	0/0	0/0	0/0	0/0	0/0	0/0	0/0	0/0	0/0	0/0
0/0	1/1	1/1	0/0	0/0	0/0	0/0	1/1	0/0	0/0	1/1	0/0	0/0	0/0	0/0	1/1	0/0	1/1	0/0	0/1	0/0	0/0	0/0	0/0	0/0	1/1	0/0
0/0	0/0	0/0	1/1	0/0	0/0	0/0	0/0	0/0	0/0	0/0	0/0	0/0	1/1	0/0	0/0	1/1	0/0	0/0	0/0	0/0	0/0	0/0	0/0	0/0	0/0	0/0
0/0	0/0	0/0	0/0	0/0	0/0	0/0	0/0	0/0	0/0	0/0	0/0	0/0	0/0	0/0	0/0	0/0	0/0	0/0	0/0	0/0	0/0	0/0	0/0	0/0	0/0	0/0
0/0	1/1	1/1	1/1	0/0	0/0	0/0	1/1	0/0	0/0	1/1	0/0	0/0	1/1	1/1	1/1	1/1	1/1	0/0	0/1	0/0	0/0	0/0	0/0	0/0	1/1	0/0
0/0	1/1	1/1	0/0	0/0	0/0	0/0	1/1	0/0	0/0	1/1	0/0	0/0	0/0	0/0	1/1	0/0	1/1	0/0	0/1	0/0	0/0	0/0	0/0	0/0	1/1	0/0
0/0	0/0	1/1	0/0	0/0	0/0	0/0	0/0	0/0	0/0	0/0	0/0	0/0	0/0	0/0	0/0	0/0	0/0	0/0	0/1	0/0	0/0	0/0	0/0	0/0	0/0	0/0
0/0	0/0	0/0	0/0	0/0	0/0	0/0	0/0	0/0	0/0	0/0	0/0	0/0	0/0	0/0	0/0	0/0	0/0	0/0	0/0	0/0	0/0	0/0	0/0	0/0	0/0	0/0
0/0	0/0	0/0	1/1	0/0	0/0	0/0	0/0	0/0	0/0	0/0	0/0	0/0	1/1	0/0	0/0	1/1	0/0	0/0	0/0	0/0	0/0	0/0	0/0	0/0	0/0	0/0
0/0	0/0	0/0	1/1	0/0	0/0	0/0	0/0	0/0	0/0	0/0	0/0	0/0	1/1	0/0	0/0	1/1	0/0	0/0	0/0	0/0	0/0	0/0	0/0	0/0	0/0	0/0
0/0	0/0	0/0	0/0	0/0	0/0	0/0	0/0	0/0	0/0	0/0	0/0	0/0	0/0	0/0	0/0	0/0	0/0	0/0	0/0	0/0	0/0	0/0	0/0	0/0	0/0	0/0
0/0	1/1	1/1	1/1	0/0	0/0	0/0	1/1	0/0	0/0	1/1	0/0	0/0	1/1	1/1	1/1	1/1	1/1	0/0	0/1	0/0	0/0	0/0	0/0	0/0	1/1	0/0
0/0	0/0	0/0	0/0	0/0	0/0	0/0	0/0	0/0	0/0	0/0	0/0	0/0	0/0	0/0	0/0	0/0	0/0	0/0	0/0	0/0	0/0	0/0	0/0	0/0	0/0	0/0
0/0	0/0	0/0	0/0	0/0	0/0	0/0	0/0	0/0	0/0	0/0	0/0	0/0	0/0	1/1	0/0	0/0	0/0	0/0	0/0	0/0	0/0	0/0	0/0	0/0	0/0	0/0
0/0	0/0	0/0	1/1	0/0	0/0	0/0	0/0	0/0	0/0	0/0	0/0	0/0	1/1	0/0	0/0	1/1	0/0	0/0	0/0	0/0	0/0	0/0	0/0	0/0	0/0	0/0
0/0	0/0	1/1	0/0	0/0	0/0	0/0	0/0	0/0	0/0	0/0	0/0	0/0	0/0	0/0	0/0	0/0	0/0	0/0	0/0	0/0	0/0	0/0	0/0	0/0	0/0	0/0
0/0	1/1	1/1	0/0	0/0	0/0	0/0	1/1	0/0	0/0	1/1	0/0	0/0	0/0	0/0	1/1	0/0	1/1	0/0	0/1	0/0	0/0	0/0	0/0	0/0	1/1	0/0
0/0	0/0	0/0	1/1	0/0	0/0	0/0	0/0	0/0	0/0	0/0	0/0	0/0	1/1	0/0	0/0	1/1	0/0	0/0	0/0	0/0	0/0	0/0	0/0	0/0	0/0	0/0
0/0	0/0	0/0	0/0	0/0	0/0	0/0	0/0	0/0	0/0	0/0	0/0	0/0	0/0	0/0	0/0	0/0	0/0	0/0	0/0	0/0	0/0	0/0	0/0	0/0	0/0	0/0
0/0	1/1	1/1	1/1	0/0	0/0	0/0	1/1	0/0	0/0	1/1	0/0	0/0	1/1	1/1	1/1	1/1	1/1	0/0	0/1	0/0	0/0	0/0	0/0	0/0	1/1	0/0
0/0	0/0	0/0	0/0	0/0	0/0	0/0	0/0	0/0	0/0	0/0	0/0	0/0	0/0	0/0	0/0	0/0	0/0	0/0	0/0	0/0	0/0	0/0	0/0	0/0	0/0	0/0
0/0	0/0	0/0	0/0	0/0	1/1	1/1	0/0	0/0	1/1	0/0	0/0	0/0	0/0	0/0	0/0	0/0	0/0	0/0	0/1	1/1	1/1	1/1	1/1	1/1	0/0	1/1

0/0	1/1	1/1	1/1	0/0	0/0	0/0	1/1	0/0	0/0	1/1	0/0	0/0	1/1	1/1	1/1	1/1	1/1	0/0	0/1	0/0	0/0	0/0	0/0	0/0	1/1	0/0
0/0	0/0	0/0	0/0	0/0	0/0	0/0	0/0	0/0	0/0	0/0	0/0	0/0	0/0	0/0	0/0	0/0	0/0	0/0	0/0	0/0	0/0	0/0	0/0	0/0	0/0	0/0
0/0	1/1	1/1	1/1	0/0	0/0	0/0	1/1	0/0	0/0	1/1	0/0	0/0	1/1	1/1	1/1	1/1	1/1	0/0	0/1	0/0	0/0	0/0	0/0	0/0	1/1	0/0
0/0	0/0	0/0	0/0	0/0	0/0	0/0	0/0	0/0	0/0	0/0	0/0	0/0	0/0	0/0	0/0	0/0	0/0	0/0	0/0	0/0	0/0	0/0	0/0	0/0	0/0	0/0
0/0	1/1	0/0	0/0	0/0	0/0	0/0	1/1	0/0	0/0	1/1	0/0	0/0	0/0	0/0	1/1	0/0	1/1	0/0	0/0	0/0	0/0	0/0	0/0	0/0	1/1	0/0
0/0	0/0	0/0	0/0	0/0	0/0	0/0	0/0	0/0	0/0	0/0	0/0	0/0	0/0	0/0	0/0	0/0	0/0	0/0	0/0	0/0	0/0	0/0	0/0	0/0	0/0	0/0
0/0	0/0	0/0	0/0	0/0	0/0	0/0	0/0	0/0	0/0	0/0	0/0	0/0	0/0	1/1	0/0	0/0	0/0	0/0	0/0	0/0	0/0	0/0	0/0	0/0	0/0	0/0
0/0	0/0	1/1	0/0	0/0	0/0	0/0	0/0	0/0	0/0	0/0	0/0	0/0	0/0	0/0	0/0	0/0	0/0	0/0	0/1	0/0	0/0	0/0	0/0	0/0	0/0	0/0
0/0	0/0	0/0	0/0	0/0	0/0	0/0	0/0	0/0	0/0	0/0	0/0	0/0	0/0	0/0	0/0	0/0	0/0	0/0	0/0	0/0	0/0	0/0	0/0	0/0	0/0	0/0
0/0	0/0	0/0	0/0	0/0	0/0	0/0	0/0	0/0	0/0	0/0	0/0	0/0	0/0	1/1	0/0	0/0	0/0	0/0	0/0	0/0	0/0	0/0	0/0	0/0	0/0	0/0
0/0	1/1	1/1	1/1	0/0	0/0	0/0	1/1	0/0	0/0	1/1	0/0	0/0	1/1	1/1	1/1	1/1	1/1	0/0	0/1	0/0	0/0	0/0	0/0	0/0	1/1	0/0
0/0	1/1	1/1	1/1	0/0	0/0	0/0	1/1	0/0	0/0	1/1	0/0	0/0	1/1	1/1	1/1	1/1	1/1	0/0	0/1	0/0	0/0	0/0	0/0	0/0	1/1	0/0
0/0	0/0	0/0	0/0	0/0	0/0	0/0	0/0	0/0	0/0	0/0	0/0	0/0	0/0	1/1	0/0	0/0	0/0	0/0	0/0	0/0	0/0	0/0	0/0	0/0	0/0	0/0
0/0	1/1	0/0	0/0	0/0	0/0	0/0	1/1	0/0	0/0	1/1	0/0	0/0	0/0	0/0	1/1	0/0	1/1	0/0	0/0	0/0	0/0	0/0	0/0	0/0	1/1	0/0
0/0	0/0	0/0	1/1	0/0	0/0	0/0	0/0	0/0	0/0	0/0	0/0	0/0	1/1	0/0	0/0	1/1	0/0	0/0	0/0	0/0	0/0	0/0	0/0	0/0	0/0	0/0
2/2	1/1	1/1	0/1	0/0	2/2	2/2	1/1	0/0	2/2	1/1	0/0	0/0	1/1	0/0	1/1	0/1	1/1	0/0	1/2	1/2	2/2	1/1	1/1	2/2	1/1	2/2
0/0	1/1	1/1	1/1	0/0	0/0	0/0	1/1	0/0	0/0	1/1	0/0	0/0	1/1	1/1	1/1	1/1	1/1	0/0	0/1	0/0	0/0	0/0	0/0	0/0	1/1	0/0
0/0	0/0	0/0	1/1	0/0	0/0	0/0	0/0	0/0	0/0	0/0	0/0	0/0	1/1	0/0	0/0	1/1	0/0	0/0	0/0	0/0	0/0	0/0	0/0	0/0	0/0	0/0
0/0	0/0	0/0	1/1	0/0	0/0	0/0	0/0	0/0	0/0	0/0	0/0	0/0	1/1	0/0	0/0	1/1	0/0	0/0	0/0	0/0	0/0	0/0	0/0	0/0	0/0	0/0
0/0	1/1	0/0	0/0	0/0	0/0	0/0	1/1	0/0	0/0	1/1	0/0	0/0	0/0	0/0	1/1	0/0	1/1	0/0	0/0	0/0	0/0	0/0	0/0	0/0	1/1	0/0
0/0	0/0	0/0	0/0	0/0	0/0	0/0	0/0	0/0	0/0	0/0	0/0	0/0	0/0	0/0	0/0	0/0	0/0	0/0	0/0	0/0	0/0	0/0	0/0	0/0	0/0	0/0
0/0	0/0	0/0	0/0	0/0	0/0	0/0	0/0	0/0	0/0	0/0	0/0	0/0	0/0	0/0	0/0	0/0	0/0	0/0	0/0	0/0	0/0	0/0	0/0	0/0	0/0	0/0
0/0	0/0	0/0	0/0	0/0	0/0	0/0	0/0	0/0	0/0	0/0	0/0	0/0	0/0	0/0	0/0	0/0	0/0	0/0	0/0	0/0	0/0	0/0	0/0	0/0	0/0	0/0
0/0	0/0	0/0	0/0	0/0	0/0	0/0	0/0	0/0	0/0	0/0	0/0	0/0	0/0	0/0	0/0	0/0	0/0	0/0	0/0	0/0	0/0	0/0	0/0	0/0	0/0	0/0
0/0	0/0	0/0	0/0	0/0	0/0	0/0	0/0	0/0	0/0	0/0	0/0	0/0	0/0	0/0	0/1	0/0	0/0	0/0	0/0	0/0	0/0	0/0	0/0	0/0	0/0	0/0
0/0	0/0	0/0	0/0	0/0	0/0	0/1	0/0	0/0	0/0	0/0	0/0	0/0	0/0	0/0	0/1	0/0	0/0	0/0	0/0	0/0	0/0	0/0	0/0	0/0	0/0	0/0

0/0	0/0	0/0	0/0	0/0	0/0	0/1	0/0	0/0	0/0	0/0	0/0	0/0	0/0	0/0	0/1	0/0	0/0	0/0	0/0	0/0	0/0	0/0	0/0	0/0	0/0	0/0
0/0	0/0	0/0	0/0	0/0	0/0	0/1	0/0	0/0	0/0	0/0	0/0	0/0	0/0	0/0	0/1	0/0	0/0	0/0	0/0	0/0	0/0	0/0	0/0	0/0	0/0	0/0
0/0	0/0	0/0	0/0	0/0	0/0	0/1	0/0	0/0	0/0	0/0	0/0	0/0	0/0	0/0	0/1	0/0	0/0	0/0	0/0	0/0	0/0	0/0	0/0	0/0	0/0	0/0
0/0	0/0	0/0	0/0	0/0	0/0	0/0	0/0	0/0	0/0	0/0	0/0	0/0	0/0	0/0	0/1	0/0	0/0	0/0	0/0	0/0	0/0	0/0	0/0	0/0	0/0	0/0
0/0	0/0	0/0	0/0	0/0	0/0	0/0	0/0	0/0	0/0	0/0	0/0	0/0	0/0	0/0	0/1	0/0	0/0	0/0	0/0	0/0	0/0	0/0	0/0	0/0	0/0	0/0
0/0	0/0	0/0	0/0	0/0	0/0	0/0	0/0	0/0	0/0	0/0	0/0	0/0	0/0	0/0	0/1	0/0	0/0	0/0	0/0	0/0	0/0	0/0	0/0	0/0	0/0	0/0
0/0	0/0	0/0	0/0	0/0	0/0	0/0	0/0	0/0	0/0	0/0	0/0	0/0	0/0	0/0	0/0	0/0	0/0	0/0	0/0	0/0	0/0	0/0	0/0	0/0	0/0	0/0
0/0	0/0	0/0	0/0	0/0	0/0	0/0	0/0	0/0	0/0	0/0	0/0	0/0	0/0	0/0	0/0	0/0	0/0	0/0	0/0	0/0	0/0	0/0	0/0	0/0	0/0	0/0
0/0	0/0	0/0	0/0	0/0	0/0	0/0	0/0	0/0	0/0	0/0	0/0	0/0	0/0	0/0	0/0	0/0	0/0	0/0	0/0	0/0	0/0	0/0	0/0	0/0	0/0	0/0
0/0	0/0	0/0	0/0	0/0	0/0	0/0	0/0	0/0	0/0	0/0	0/0	0/0	0/0	0/0	0/0	0/0	0/0	0/0	0/0	0/0	0/0	0/0	0/0	0/0	0/0	0/0
1/1	1/1	1/1	1/1	0/0	0/0	0/0	1/1	0/0	0/0	1/1	0/0	0/0	1/1	1/1	1/1	1/1	1/1	0/0	1/1	1/1	1/1	1/1	1/1	1/1	1/1	0/0
0/0	0/0	0/0	0/0	0/0	0/0	0/0	0/0	0/0	0/0	0/0	0/0	0/0	0/0	0/0	0/0	0/0	0/0	0/0	0/0	0/0	0/0	0/0	0/0	0/0	0/0	0/0
0/0	0/0	0/0	0/0	0/0	0/0	0/0	0/0	0/0	0/0	0/0	0/0	0/0	0/0	0/0	0/0	0/0	0/0	0/0	0/0	0/0	0/0	0/0	0/0	0/0	0/0	0/0
0/0	0/0	1/1	0/0	0/0	0/0	0/0	0/0	0/0	0/0	0/0	0/0	0/0	0/0	1/1	0/0	0/0	0/0	0/0	0/1	0/0	0/0	0/0	0/0	0/0	0/0	0/0
0/0	0/0	0/0	0/0	0/0	0/0	0/0	0/0	0/0	0/0	0/0	0/0	0/0	0/0	0/0	0/0	0/0	0/0	0/0	0/0	0/0	0/0	0/0	0/0	0/0	0/0	0/0
0/0	0/0	0/0	0/0	0/0	0/0	0/0	0/0	0/0	0/0	0/0	0/0	0/0	0/0	0/0	0/0	0/0	0/0	0/0	0/0	0/0	0/0	0/0	0/0	0/0	0/0	0/0
0/0	1/1	1/1	1/1	0/0	0/0	0/0	1/1	0/0	0/0	1/1	0/0	0/0	1/1	1/1	1/1	1/1	1/1	0/0	0/1	0/0	0/0	0/0	0/0	0/0	1/1	0/0
0/0	0/0	0/0	./.	0/0	0/0	0/0	0/0	0/0	0/0	0/0	0/0	0/0	./.	0/0	0/0	./.	0/0	0/0	0/0	0/0	0/0	0/0	0/0	0/0	0/0	0/0
0/0	1/1	1/1	./.	0/0	0/0	0/0	1/1	0/0	0/0	1/1	0/0	0/0	./.	1/1	1/1	./.	1/1	0/0	0/1	0/0	0/0	0/0	0/0	0/0	1/1	0/0
0/0	0/0	0/0	1/1	0/0	0/0	0/0	0/0	0/0	0/0	0/0	0/0	0/0	1/1	0/0	0/0	1/1	0/0	0/0	0/0	0/0	0/0	0/0	0/0	0/0	0/0	0/0
0/0	0/0	0/0	1/1	0/0	0/0	0/0	0/0	0/0	0/0	0/0	0/0	0/0	1/1	0/0	0/0	1/1	0/0	0/0	0/0	0/0	0/0	0/0	0/0	0/0	0/0	0/0
0/0	./.	0/0	0/0	0/0	0/0	0/0	./.	0/0	0/0	0/0	0/0	0/0	0/0	0/0	./.	0/0	./.	0/0	0/0	./.	0/0	0/0	0/0	./.	0/0	0/0
0/0	0/1	0/0	0/0	0/0	0/0	0/0	0/0	0/0	./.	0/1	0/0	0/0	0/0	./.	./.	0/0	./.	0/0	0/0	./.	./.	0/0	0/0	./.	0/0	0/0
0/0	0/1	0/1	0/0	0/0	0/0	0/0	1/1	0/0	./.	0/1	0/0	0/0	0/0	1/1	1/1	0/0	1/1	0/0	0/1	./.	./.	0/0	0/0	./.	1/1	0/0

0/0	0/2	0/1	0/0	0/0	0/0	0/0	0/0	0/0	./.	0/2	0/0	0/0	0/0	2/2	2/2	0/0	2/2	0/0	0/2	./.	0/0	0/0	0/0	0/0	2/2	0/0
2/3	0/4	0/0	0/0	0/0	0/0	0/0	0/0	0/0	2/2	0/4	0/0	0/0	0/0	4/4	4/4	0/0	4/4	0/0	0/3	3/3	3/3	3/3	0/3	3/3	4/4	0/0
0/0	0/0	0/0	0/0	0/0	0/0	0/0	0/0	0/0	0/0	0/0	0/0	0/0	0/0	0/0	0/0	0/0	0/0	0/0	0/0	0/0	0/0	0/0	0/0	0/0	0/0	0/0
0/0	1/1	1/1	1/1	0/0	0/0	0/0	1/1	0/0	0/0	1/1	0/0	0/0	1/1	1/1	1/1	1/1	1/1	0/0	0/1	0/0	0/0	0/0	0/0	0/0	1/1	0/0
0/0	1/1	1/1	0/0	0/0	0/0	0/0	1/1	0/0	0/0	1/1	0/0	0/0	0/0	1/1	1/1	0/0	1/1	0/0	0/1	0/0	0/0	0/0	0/1	0/0	1/1	0/0
0/0	0/0	0/0	0/0	0/0	0/0	0/0	0/0	0/0	0/0	0/0	0/0	0/0	0/0	0/0	0/0	0/0	0/0	0/0	0/0	0/0	0/0	0/0	0/0	0/0	0/0	0/0
0/0	0/0	0/0	0/0	0/0	0/0	0/0	0/0	0/0	0/0	0/0	0/0	0/0	0/0	0/0	0/0	0/0	0/0	0/0	0/0	0/0	0/0	0/0	0/0	0/0	0/0	0/0
0/0	1/1	1/1	1/1	0/0	0/0	0/0	1/1	0/0	0/0	1/1	0/0	0/0	1/1	1/1	1/1	1/1	1/1	0/0	0/1	0/0	0/0	0/0	0/1	0/0	1/1	0/0
0/0	./.	./.	0/0	0/0	0/0	0/0	0/0	0/0	0/0	./.	0/0	0/0	0/0	0/0	./.	0/0	./.	0/0	0/0	0/0	0/0	0/0	0/0	0/0	0/0	0/0
0/0	1/1	1/1	0/0	0/0	0/0	0/0	1/1	0/0	0/0	1/1	0/0	0/0	0/0	1/1	1/1	0/0	1/1	0/0	0/1	0/0	0/0	0/0	0/1	0/0	1/1	0/0
0/0	1/1	1/1	0/0	0/0	0/0	0/0	1/1	0/0	0/0	1/1	0/0	0/0	0/0	1/1	1/1	0/0	1/1	0/0	0/1	0/0	0/0	0/0	0/1	0/0	1/1	0/0
0/0	1/1	1/1	0/0	0/0	0/0	0/0	1/1	0/0	0/0	1/1	0/0	0/0	0/0	1/1	1/1	0/0	1/1	0/0	0/1	0/0	0/0	0/0	0/1	0/0	1/1	0/0
0/0	0/0	0/0	1/1	0/0	0/0	0/0	0/0	0/0	0/0	0/0	0/0	0/0	1/1	0/0	0/0	1/1	0/0	0/0	0/0	0/0	0/0	0/0	0/0	0/0	0/0	0/0
0/0	1/1	1/1	0/0	0/0	0/0	0/0	1/1	0/0	0/0	1/1	0/0	0/0	0/0	1/1	1/1	0/0	1/1	0/0	0/1	0/0	0/0	0/0	0/1	0/0	1/1	0/0
0/0	1/1	1/1	1/1	0/0	0/0	0/0	1/1	0/0	0/0	1/1	0/0	0/0	1/1	1/1	1/1	1/1	1/1	0/0	0/1	0/0	0/0	0/0	0/1	0/0	1/1	0/0
0/0	0/0	0/0	0/0	0/0	0/0	0/0	0/0	0/0	0/0	0/0	0/0	0/0	0/0	0/0	0/0	0/0	0/0	0/0	0/0	0/0	0/0	0/0	0/0	0/0	0/0	0/0
0/0	0/0	0/0	0/0	0/0	0/0	0/0	0/0	0/0	0/0	0/0	0/0	0/0	0/0	0/0	0/0	0/0	0/0	0/0	0/0	0/0	0/0	0/0	0/0	0/0	0/0	0/0
0/0	0/0	0/0	1/1	0/0	0/0	0/0	0/0	0/0	0/0	0/0	0/0	0/0	1/1	0/0	0/0	1/1	0/0	0/0	0/0	0/0	0/0	0/0	0/0	0/0	0/0	0/0
0/0	0/0	0/0	0/0	0/0	0/0	0/0	0/0	0/0	0/0	0/0	0/0	0/0	0/0	0/0	0/0	0/0	0/0	0/0	0/0	0/0	0/0	0/0	0/0	0/0	0/0	0/0
0/0	0/0	0/0	1/1	0/0	0/0	0/0	0/0	0/0	0/0	0/0	0/0	0/0	1/1	0/0	0/0	1/1	0/0	0/0	0/0	0/0	0/0	0/0	0/0	0/0	0/0	0/0
0/0	0/0	1/1	0/0	0/0	0/0	0/0	0/0	0/0	0/0	0/0	0/0	0/0	0/0	1/1	0/0	0/0	0/0	0/0	0/1	0/0	0/0	0/0	0/0	0/0	0/0	0/0
0/0	0/0	0/0	1/1	0/0	0/0	0/0	0/0	0/0	0/0	0/0	0/0	0/0	1/1	0/0	0/0	1/1	0/0	0/0	0/0	0/0	0/0	0/0	0/0	0/0	0/0	0/0
0/0	0/0	0/0	1/1	0/0	0/0	0/0	0/0	0/0	0/0	0/0	0/0	0/0	1/1	0/0	0/0	1/1	0/0	0/0	0/0	0/0	0/0	0/0	0/0	0/0	0/0	0/0
0/0	0/0	0/0	0/0	0/0	0/0	0/0	0/0	0/0	0/0	0/0	0/0	0/0	0/0	0/0	0/0	0/0	0/0	0/0	0/0	0/0	0/0	0/0	0/0	0/0	0/0	0/0
0/0	0/0	0/0	0/0	0/0	0/0	0/0	0/0	0/0	0/0	0/0	0/0	0/0	0/0	0/0	0/0	0/0	0/0	0/0	0/0	0/0	0/0	0/0	0/0	0/0	0/0	0/0
0/0	1/1	1/1	0/0	0/0	0/0	0/0	1/1	0/0	0/0	1/1	0/0	0/0	0/0	1/1	1/1	0/0	1/1	0/0	0/1	0/0	0/0	0/0	0/0	0/0	1/1	0/0
0/0	0/0	0/0	0/0	0/0	0/0	0/0	0/0	0/0	0/0	0/0	0/0	0/0	0/0	0/0	0/0	0/0	0/0	0/0	0/0	0/0	0/0	0/0	0/0	0/0	0/0	0/0
0/0	1/1	1/1	1/1	0/0	0/0	0/0	1/1	0/0	0/0	1/1	0/0	0/0	1/1	1/1	1/1	1/1	1/1	0/0	0/1	0/0	0/0	0/0	0/0	0/0	1/1	0/0
0/0	0/0	0/0	0/0	0/0	0/0	0/0	0/0	0/0	0/0	0/0	0/0	0/0	0/0	0/0	0/0	0/0	0/0	0/0	0/0	0/0	0/0	0/0	0/0	0/0	0/0	0/0
0/0	1/1	1/1	1/1	0/0	0/0	0/0	1/1	0/0	0/0	1/1	0/0	0/0	1/1	1/1	1/1	1/1	1/1	0/0	0/1	0/0	0/0	0/0	0/0	0/0	1/1	0/0

0/0	0/0	0/0	0/0	0/0	0/0	0/0	0/0	0/0	0/0	0/0	0/0	0/0	0/0	0/0	0/0	0/0	0/0	0/0	0/0	0/0	0/0	0/0	0/0	0/0	0/0	0/0
0/0	0/0	0/0	0/0	0/0	0/0	0/0	0/0	0/0	0/0	0/0	0/0	0/0	0/0	0/0	0/0	0/0	0/0	0/0	0/0	0/0	0/0	0/0	0/0	0/0	0/0	0/0
0/0	0/0	0/0	0/0	0/0	0/0	0/0	0/0	0/0	0/0	0/0	0/0	0/0	0/0	0/0	0/0	0/0	0/0	0/0	0/0	0/0	0/0	0/0	0/0	0/0	0/0	0/0
0/0	1/1	0/0	0/0	0/0	0/0	0/0	1/1	0/0	0/0	1/1	0/0	0/0	0/0	0/0	1/1	0/0	1/1	0/0	0/0	0/0	0/0	0/0	0/0	0/0	1/1	0/0
0/0	1/1	1/1	0/0	0/0	0/0	0/0	1/1	0/0	0/0	1/1	0/0	0/0	0/0	1/1	1/1	0/0	1/1	0/0	0/1	0/0	0/0	0/0	0/0	0/0	1/1	0/0
0/0	1/1	1/1	1/1	0/0	0/0	0/0	1/1	0/0	0/0	1/1	0/0	0/0	1/1	1/1	1/1	1/1	1/1	0/0	0/1	0/0	0/0	0/0	0/0	0/0	1/1	0/0
0/0	1/1	1/1	0/0	0/0	0/0	0/0	1/1	0/0	0/0	1/1	0/0	0/0	0/0	1/1	1/1	0/0	1/1	0/0	0/1	0/0	0/0	0/0	0/0	0/0	1/1	0/0
0/0	0/0	1/1	0/0	0/0	0/0	0/0	0/0	0/0	0/0	0/0	0/0	0/0	0/0	1/1	0/0	0/0	0/0	0/0	0/1	0/0	0/0	0/0	0/0	0/0	0/0	0/0
0/0	0/0	0/0	0/0	0/0	0/0	0/0	0/0	0/0	0/0	0/0	0/0	0/0	0/0	0/0	0/0	0/0	0/0	0/0	0/0	0/0	0/0	0/0	0/0	0/0	0/0	0/0
0/0	0/0	0/0	0/0	0/0	0/0	0/0	0/0	0/0	0/0	0/0	0/0	0/0	0/0	0/0	0/0	0/0	0/0	0/0	0/0	0/0	0/0	0/0	0/0	0/0	0/0	0/0
0/0	0/0	0/0	0/0	0/0	0/0	0/0	0/0	0/0	0/0	0/0	0/0	0/0	0/0	0/0	0/0	0/0	0/0	0/0	0/0	0/0	0/0	0/0	0/0	0/0	0/0	0/0
0/0	0/0	0/0	1/1	0/0	0/0	0/0	0/0	0/0	0/0	0/0	0/0	0/0	1/1	0/0	0/0	1/1	0/0	0/0	0/0	0/0	0/0	0/0	0/0	0/0	0/0	0/0
0/0	1/1	0/0	1/1	0/0	0/0	0/0	1/1	0/0	0/0	1/1	0/0	0/0	1/1	0/0	1/1	1/1	1/1	0/0	0/0	0/0	0/0	0/0	0/1	0/0	1/1	0/0
0/0	1/1	1/1	1/1	0/0	0/0	0/0	1/1	0/0	0/0	1/1	0/0	0/0	1/1	1/1	1/1	1/1	1/1	0/0	0/1	0/0	0/0	0/0	0/1	0/0	1/1	0/0
1/1	1/1	1/1	1/1	1/1	1/1	1/1	1/1	1/1	1/1	1/1	1/1	1/1	1/1	1/1	1/1	1/1	1/1	1/1	1/1	1/1	1/1	1/1	1/1	1/1	1/1	1/1
0/0	0/0	0/0	0/0	0/0	0/0	0/0	0/0	0/0	0/0	0/0	0/0	0/0	0/0	0/0	0/0	0/0	0/0	0/0	0/0	0/0	0/0	0/0	0/0	0/0	0/0	0/0
0/0	1/1	0/0	0/0	0/0	0/0	0/0	1/1	0/0	0/0	1/1	0/0	0/0	0/0	0/0	1/1	0/0	1/1	0/0	0/0	0/0	0/0	0/0	0/1	0/0	1/1	0/0
0/0	1/1	1/1	0/0	0/0	0/0	0/0	1/1	0/0	0/0	1/1	0/0	0/0	0/0	1/1	1/1	0/0	1/1	0/0	0/1	0/0	0/0	0/0	0/1	0/0	1/1	0/0
0/0	1/1	1/1	1/1	0/0	0/0	0/0	1/1	0/0	0/0	1/1	0/0	0/0	1/1	1/1	1/1	1/1	1/1	0/0	0/1	0/0	0/0	0/0	0/1	0/0	1/1	0/0
0/0	0/0	0/0	0/0	0/0	0/0	0/0	0/0	0/0	0/0	0/0	0/0	0/0	0/0	0/0	0/0	0/0	0/0	0/0	0/0	0/0	0/0	0/0	0/0	0/0	0/0	0/0
0/0	0/0	0/0	0/0	0/0	0/0	0/0	0/0	0/0	0/0	0/0	0/0	0/0	0/0	0/0	0/0	0/0	0/0	0/0	0/0	0/0	0/0	0/0	0/0	0/0	0/0	0/0
0/0	0/0	0/0	0/0	0/0	0/0	0/0	0/0	0/0	0/0	0/0	0/0	0/0	0/0	0/0	0/0	0/0	0/0	0/0	0/0	0/0	0/0	0/0	0/0	0/0	0/0	0/0
0/0	1/1	1/1	1/1	0/0	0/0	0/0	1/1	0/0	0/0	1/1	0/0	0/0	1/1	1/1	1/1	1/1	1/1	0/0	0/1	0/0	0/0	0/0	0/0	0/0	1/1	0/0
0/0	0/0	0/0	0/0	0/0	0/0	0/0	0/0	0/0	0/0	0/0	0/0	0/0	0/0	0/0	0/0	0/0	0/0	0/0	0/0	0/0	0/0	0/0	0/0	0/0	0/0	0/0
0/0	0/0	0/0	0/0	0/0	0/0	0/0	0/0	0/0	0/0	0/0	0/0	0/0	0/0	0/0	0/0	0/0	0/0	0/0	0/0	0/0	0/0	0/0	0/0	0/0	0/0	0/0
0/0	0/0	0/0	0/0	0/0	0/0	0/0	0/0	0/0	0/0	0/0	0/0	0/0	0/0	0/0	0/0	0/0	0/0	0/0	0/0	0/0	0/0	0/0	0/0	0/0	0/0	0/0
0/0	0/0	0/0	0/0	0/0	0/0	0/0	0/0	0/0	0/0	0/0	0/0	0/0	0/0	0/0	0/0	0/0	0/0	0/0	0/0	0/0	0/0	0/0	0/0	0/0	0/0	0/0
0/0	0/0	0/0	0/0	0/0	0/0	0/0	0/0	0/0	0/0	0/0	0/0	0/0	0/0	0/0	0/0	0/0	0/0	0/0	0/0	0/0	0/0	0/0	0/0	0/0	0/0	0/0
0/0	0/0	0/0	0/0	0/0	0/0	0/0	0/0	0/0	0/0	0/0	0/0	0/0	0/0	0/0	0/0	0/0	0/0	0/0	0/0	0/0	0/0	0/0	0/0	0/0	0/0	0/0
0/0	0/0	0/0	0/0	0/0	0/0	0/0	0/0	0/0	0/0	0/0	0/0	0/0	0/0	0/0	0/0	0/0	0/0	0/0	0/0	0/0	0/0	0/0	0/0	0/0	0/0	0/0
0/0	0/0	0/0	0/0	0/0	0/0	0/0	0/0	0/0	0/0	0/0	0/0	0/0	0/0	0/0	0/0	0/0	0/0	0/0	0/0	0/0	0/0	0/0	0/0	0/0	0/0	0/0
0/0	0/0	0/0	1/1	0/0	0/0	0/0	0/0	0/0	0/0	0/0	0/0	0/0	1/1	0/0	0/0	1/1	0/0	0/0	0/0	0/0	0/0	0/0	0/0	0/0	0/0	0/0
0/0	0/0	0/0	0/0	0/0	0/0	0/0	0/0	0/0	0/0	0/0	0/0	0/0	0/0	0/0	0/0	0/0	0/0	0/0	0/0	0/0	0/0	0/0	0/0	0/0	0/0	0/0
0/0	0/0	0/0	0/0	0/0	0/0	0/0	0/0	0/0	0/0	0/0	0/0	0/0	0/0	0/0	0/0	0/0	0/0	0/0	0/0	0/0	0/0	0/0	0/0	0/0	0/0	0/0
0/0	0/0	0/0	0/0	0/0	0/0	0/0	0/0	0/0	0/0	0/0	0/0	0/0	0/0	0/0	1/1	0/0	0/0	0/0	0/0	0/0	0/0	0/0	0/0	0/0	1/1	0/0

0/0	0/0	1/1	0/0	0/0	0/0	0/0	0/0	0/0	0/0	0/0	0/0	0/0	0/0	1/1	0/0	0/0	0/0	0/0	0/1	0/0	0/0	0/0	0/0	0/0	0/0	0/0
1/2	2/2	4/4	1/2	1/4	3/5	3/5	2/2	0/0	3/3	2/2	4/4	0/0	2/2	4/4	2/3	1/1	2/2	1/4	2/4	2/3	2/2	3/3	3/3	2/2	2/2	3/3
./.	./.	./.	./.	./.	./.	./.	./.	0/0	./.	./.	./.	0/0	./.	./.	./.	./.	./.	./.	./.	./.	./.	./.	./.	./.	./.	./.
0/0	0/0	0/0	0/0	0/0	0/0	0/0	0/0	0/0	0/0	0/0	0/0	0/0	0/0	0/0	0/0	0/0	0/0	0/0	0/0	0/0	0/0	0/0	0/0	0/0	0/0	0/0
0/0	0/0	0/0	0/0	0/0	0/0	0/0	0/0	0/0	0/0	0/0	0/0	0/0	0/0	0/0	0/0	0/0	0/0	0/0	0/0	0/0	0/0	0/0	0/0	0/0	0/0	0/0
0/0	0/0	0/0	0/0	0/0	0/0	0/0	0/0	0/0	0/0	0/0	0/0	0/0	0/0	0/0	0/0	0/0	0/0	0/0	0/0	0/0	0/0	0/0	0/0	0/0	0/0	0/0
0/0	0/0	1/1	0/0	0/0	0/0	0/0	0/0	0/0	0/0	0/0	0/0	0/0	0/0	1/1	0/0	0/0	0/0	0/0	0/1	0/0	0/0	0/0	0/0	0/0	0/0	0/0
./.	0/0	1/1	0/0	0/0	0/0	0/0	0/0	0/0	0/0	0/0	0/0	0/0	0/0	1/1	0/0	0/0	0/0	0/0	0/1	0/0	0/0	0/0	0/0	0/0	0/0	0/0
./.	0/0	0/0	0/0	0/0	0/0	0/0	0/0	0/0	0/0	0/0	0/0	0/0	0/0	./.	0/0	0/0	./.	0/0	./.	0/0	./.	0/0	0/0	0/0	0/0	0/0
0/0	1/1	1/1	./.	0/0	./.	./.	1/1	0/0	./.	1/1	0/0	0/0	0/0	1/1	1/1	0/0	1/1	0/0	0/1	./.	./.	./.	./.	0/0	1/1	./.
1/1	./.	./.	2/2	0/0	1/1	1/1	./.	0/0	1/1	./.	0/0	0/0	2/2	./.	./.	2/2	./.	0/0	0/1	1/1	1/1	1/1	1/1	1/1	./.	1/1
2/2	0/0	./.	./.	0/0	2/2	2/2	0/0	0/0	2/2	./.	0/0	0/0	./.	0/0	./.	./.	./.	0/0	0/2	2/2	2/2	2/2	2/2	2/2	./.	2/2
0/0	0/0	0/0	0/0	0/0	0/0	0/0	0/0	0/0	0/0	0/0	0/0	0/0	0/0	0/0	0/0	0/0	0/0	0/0	0/0	0/0	0/0	0/0	0/0	0/0	0/0	0/0
0/0	0/0	0/0	0/0	0/0	0/0	0/0	0/0	1/1	0/0	0/0	0/0	1/1	0/0	0/0	0/0	0/0	0/0	0/0	0/0	0/0	0/0	0/0	0/0	0/0	0/0	0/0
0/0	0/0	0/0	0/0	0/0	0/0	0/0	0/0	0/0	0/0	0/0	0/0	0/0	0/0	0/0	0/0	0/0	0/0	0/0	0/0	0/0	0/0	0/0	0/0	0/0	0/0	0/0
0/0	1/1	1/1	1/1	0/0	0/0	0/0	1/1	0/0	0/0	1/1	0/0	0/0	1/1	1/1	1/1	1/1	1/1	0/0	0/1	0/0	0/0	0/0	0/0	0/0	1/1	0/0
0/0	0/0	0/0	0/0	0/0	0/0	0/0	0/0	0/0	0/0	0/0	0/0	0/0	0/0	0/0	0/0	0/0	0/0	0/0	0/0	0/0	0/0	0/0	0/0	0/0	0/0	0/0
0/0	1/1	0/0	0/0	0/0	0/0	0/0	1/1	0/0	0/0	1/1	0/0	0/0	0/0	0/0	1/1	0/0	1/1	0/0	0/0	0/0	0/0	0/0	0/0	0/0	1/1	0/0
0/0	0/0	0/0	0/0	0/0	0/0	0/0	0/0	0/0	0/0	0/0	0/0	0/0	0/0	0/0	0/0	0/0	0/0	0/0	0/0	0/0	0/0	0/0	0/0	0/0	0/0	0/0
0/0	0/0	0/0	0/0	0/0	0/0	0/0	0/0	0/0	0/0	0/0	0/0	0/0	0/0	0/0	0/0	0/0	0/0	0/0	0/0	0/0	0/0	0/0	0/0	0/0	0/0	0/0
0/0	0/0	0/0	0/0	0/0	0/0	0/0	0/0	0/0	0/0	0/0	0/0	0/0	0/0	0/0	0/0	0/0	0/0	0/0	0/0	0/0	0/0	0/0	0/0	0/0	0/0	0/0

0/0	0/0	0/0	0/0	0/0	0/0	0/0	0/0	0/0	0/0	0/0	0/0	0/0	0/0	0/0	0/0	0/0	0/0	0/0	0/0	0/0	0/0	0/0	0/0	0/0	0/0	0/0
0/0	0/0	0/0	0/0	0/0	0/0	0/0	0/0	0/0	0/0	0/0	0/0	0/0	0/0	0/0	0/0	0/0	0/0	0/0	0/0	0/0	0/0	0/0	0/0	0/0	0/0	0/0
0/0	1/1	1/1	2/2	0/0	0/0	0/0	1/1	0/0	0/0	1/1	0/0	0/0	2/2	1/1	1/1	2/2	1/1	0/0	0/1	0/0	0/0	0/0	0/0	0/0	1/1	0/0
0/0	0/0	0/0	0/0	0/0	0/0	0/0	0/0	0/0	0/0	0/0	0/0	0/0	0/0	0/0	0/0	0/0	0/0	0/0	0/0	0/0	0/0	0/0	0/0	0/0	0/0	0/0
0/0	1/1	0/0	2/2	0/0	0/0	0/0	1/1	0/0	0/0	1/1	0/0	0/0	2/2	0/0	1/1	2/2	1/1	0/0	0/0	0/0	0/0	0/0	0/0	0/0	1/1	0/0
0/0	0/0	0/0	0/0	0/0	0/0	0/0	0/0	0/0	0/0	0/0	0/0	0/0	0/0	0/0	0/0	0/0	0/0	0/0	0/0	0/0	0/0	0/0	0/0	0/0	0/0	0/0
0/0	0/0	1/1	0/0	0/0	0/0	0/0	0/0	0/0	0/0	0/0	0/0	0/0	0/0	0/0	0/0	0/0	0/0	0/0	0/1	0/0	0/0	0/0	0/0	0/0	0/0	0/0
0/0	1/1	1/1	1/1	0/0	0/0	0/0	1/1	0/0	0/0	1/1	0/0	0/0	1/1	1/1	1/1	1/1	1/1	0/0	0/1	0/0	0/0	0/0	0/0	0/0	1/1	0/0
0/0	1/1	1/1	1/1	0/0	0/0	0/0	1/1	0/0	0/0	1/1	0/0	0/0	1/1	1/1	1/1	1/1	1/1	0/0	0/1	0/0	0/0	0/0	0/0	0/0	1/1	0/0
0/0	0/0	0/0	0/0	0/0	0/0	0/0	0/0	0/0	0/0	0/0	0/0	0/0	0/0	0/0	0/0	0/0	0/0	0/0	0/0	0/0	0/0	0/0	0/0	0/0	0/0	0/0
0/0	1/1	2/2	2/2	0/0	0/0	0/0	1/1	0/0	0/0	1/1	0/0	0/0	2/2	2/2	1/1	2/2	1/1	0/0	0/2	0/0	0/0	0/0	0/0	0/0	1/1	0/0
0/0	0/0	0/0	0/0	0/0	0/0	0/0	0/0	0/0	0/0	0/0	0/0	0/0	0/0	0/0	0/0	0/0	0/0	0/0	0/1	0/0	0/0	0/0	0/0	0/0	0/0	0/0
0/0	0/0	0/0	0/0	0/0	0/0	0/0	0/0	0/0	0/0	0/0	0/0	0/0	0/0	0/0	0/0	0/0	0/0	0/0	0/0	0/0	0/0	0/0	0/0	0/0	0/0	0/0
0/0	0/0	0/0	0/0	0/0	0/0	0/0	0/0	0/0	0/0	0/0	0/0	0/0	0/0	0/0	0/0	0/0	0/0	0/0	0/0	0/0	0/0	0/0	0/0	0/0	0/0	0/0
0/0	0/0	1/1	0/0	0/0	0/0	0/0	0/0	0/0	0/0	0/0	0/0	0/0	0/0	1/1	0/0	0/0	0/0	0/0	0/1	0/0	0/0	0/0	0/0	0/0	0/0	0/0
0/0	0/0	0/0	0/0	0/0	0/0	0/0	0/0	0/0	0/0	0/0	0/0	0/0	0/0	0/0	0/0	0/0	0/0	0/0	0/0	0/0	0/0	0/0	0/0	0/0	0/0	0/0
0/0	0/0	1/1	0/0	0/0	0/0	0/0	0/0	0/0	0/0	0/0	0/0	0/0	0/0	1/1	0/0	0/0	0/0	0/0	0/1	0/0	0/0	0/0	0/0	0/0	0/0	0/0
0/0	1/1	1/1	1/1	0/0	0/0	0/0	1/1	0/0	0/0	1/1	0/0	0/0	1/1	1/1	1/1	1/1	1/1	0/0	0/1	0/0	0/0	0/0	0/0	0/0	1/1	0/0

1283	1291	1298	1301	1307	1310	1313	1320	1321	1325	1327	1339	1347	1348	1349	1350	1353	1360	1372	1374	1387	1388	1390	1409	1416	1431	1435
0/0	0/0	0/0	0/0	0/0	0/0	0/0	0/0	0/0	0/0	0/0	0/0	0/0	0/0	0/0	0/0	0/0	0/0	0/0	0/0	0/0	0/0	0/0	0/0	0/0	0/0	0/0
0/0	1/1	0/0	0/0	0/0	1/1	0/0	0/0	0/0	1/1	0/0	0/0	0/0	0/0	0/0	0/1	0/0	0/0	1/1	1/1	1/1	0/0	1/1	1/1	0/0	1/1	1/1
0/0	0/0	0/0	0/0	0/0	0/0	0/0	0/0	0/0	0/0	0/0	0/0	0/0	0/0	0/0	0/0	0/0	0/0	0/0	0/0	0/0	0/0	0/0	0/0	0/0	0/0	0/0
0/0	0/0	0/0	0/0	0/0	0/0	0/0	0/0	0/0	0/0	0/0	0/0	0/0	0/0	0/0	0/0	0/0	0/0	0/0	0/0	0/0	0/0	0/0	0/0	0/0	0/0	0/0
0/0	0/0	1/1	0/0	0/0	0/0	0/0	0/0	0/0	0/0	0/0	0/0	1/1	0/0	0/0	0/0	0/0	0/0	0/0	0/0	0/0	0/0	0/0	0/0	0/0	0/0	0/0
0/0	0/0	1/1	0/0	0/0	0/0	0/0	0/0	0/0	0/0	0/0	0/0	1/1	0/0	0/0	0/0	0/0	0/0	0/0	0/0	0/0	0/0	0/0	0/0	0/0	0/0	0/0
0/0	0/0	0/0	0/0	0/0	0/0	0/0	0/0	0/0	0/0	0/0	0/0	0/0	0/0	0/0	0/0	0/0	0/0	0/0	0/0	0/0	0/0	0/0	0/0	0/0	0/0	0/0
0/0	1/1	0/0	0/0	0/0	1/1	0/0	0/0	0/0	1/1	0/0	0/0	0/0	0/0	1/1	1/1	0/0	0/0	1/1	1/1	1/1	0/0	1/1	1/1	1/1	1/1	1/1
0/0	0/0	0/0	0/0	0/0	0/0	0/0	0/0	0/0	0/0	0/0	0/0	0/0	0/0	0/0	0/0	0/0	0/0	0/0	0/0	0/0	0/0	0/0	0/0	0/0	0/0	0/0
0/0	0/0	0/0	0/0	0/0	0/0	0/0	0/0	0/0	0/0	0/0	0/0	0/0	0/0	0/0	0/0	0/0	0/0	0/0	0/0	0/0	0/0	0/0	0/0	0/0	0/0	0/0
0/0	0/1	0/0	0/0	0/0	0/0	0/0	0/0	0/0	0/0	0/0	0/0	0/0	0/0	0/0	0/0	0/0	0/1	0/0	0/0	0/0	0/0	0/0	0/0	0/0	0/0	0/0
0/0	0/0	0/0	0/0	0/0	0/0	0/0	0/0	0/0	0/0	0/0	0/0	0/0	0/0	0/0	0/0	0/0	0/0	0/0	0/0	0/0	0/0	0/0	0/0	0/0	0/0	0/0
0/0	0/0	1/1	0/0	0/0	0/0	0/0	0/0	0/0	0/0	0/0	0/0	1/1	0/0	0/0	0/0	0/0	0/0	0/0	0/0	0/0	0/0	0/0	0/0	0/0	0/0	0/0
0/0	0/1	0/0	0/0	0/0	0/1	0/0	0/0	0/0	0/0	0/0	0/0	0/0	0/0	0/0	0/0	0/0	0/0	0/0	0/0	./.	0/0	0/0	0/0	0/0	0/0	./.
./.	0/1	./.	0/0	0/0	0/1	0/0	0/0	0/0	1/1	0/0	0/0	0/0	0/0	1/1	1/1	0/0	./.	1/1	1/1	1/1	0/0	1/1	1/1	1/1	1/1	1/1
./.	0/0	./.	0/0	0/0	0/0	0/0	0/0	0/0	0/0	0/0	0/0	0/0	0/0	0/0	0/0	0/0	./.	0/0	0/0	./.	0/0	0/0	0/0	0/0	0/0	./.

4/4	3/5	./.	0/0	0/0	3/5	0/1	0/0	4/4	3/3	0/0	0/0	3/3	2/2	0/0	./.	0/1	4/4	3/3	3/3	./.	2/2	./.	./.	0/0	3/3	0/0
0/0	0/0	0/0	0/0	0/0	0/0	0/0	0/0	0/0	0/0	0/0	0/0	0/0	0/0	0/0	0/0	0/0	0/0	0/0	0/0	0/0	0/0	0/0	./.	0/0	0/0	./.
0/0	0/0	0/0	0/0	0/0	0/0	0/0	0/0	0/0	0/0	0/0	0/0	0/0	0/0	0/0	./.	0/0	0/0	0/0	0/0	0/0	0/0	0/0	./.	0/0	0/0	./.
0/0	0/0	1/1	0/0	0/0	0/0	0/0	0/0	0/0	0/0	0/0	0/0	1/1	0/0	0/0	0/0	0/0	0/0	0/0	0/0	0/0	0/0	0/0	0/0	0/0	0/0	0/0
0/0	1/1	0/0	0/0	0/0	1/1	0/0	0/0	0/0	1/1	0/0	0/0	0/0	0/0	1/1	0/1	0/0	0/0	1/1	1/1	1/1	0/0	1/1	1/1	1/1	1/1	1/1
0/0	0/0	0/0	0/0	0/0	0/0	0/0	0/0	0/0	0/0	0/0	0/0	0/0	0/0	0/0	0/0	0/0	0/0	0/0	0/0	0/0	0/0	0/0	0/0	0/0	0/0	0/0
0/0	1/1	0/0	0/0	0/0	1/1	0/0	0/0	0/0	1/1	0/0	0/0	0/0	0/0	0/1	0/1	0/0	0/0	1/1	1/1	1/1	0/0	1/1	1/1	1/1	1/1	1/1
0/0	0/0	1/1	0/0	0/0	0/0	0/0	0/0	0/0	0/0	0/0	0/0	1/1	0/0	0/0	0/0	0/0	0/0	0/0	0/0	0/0	0/0	0/0	0/0	0/0	0/0	0/0
0/0	0/0	0/0	0/0	1/1	0/0	0/0	0/0	0/0	0/0	0/0	0/0	0/0	0/0	0/0	0/0	0/0	0/0	0/0	0/0	0/0	1/1	0/0	0/0	0/0	0/0	0/0
0/0	0/0	1/1	0/0	2/2	0/0	0/0	0/0	0/0	0/0	0/0	0/0	1/1	0/0	1/1	0/0	0/0	0/0	0/0	0/0	0/0	2/2	0/0	0/0	1/1	0/0	0/0
0/0	0/0	0/0	0/0	./.	0/0	0/0	0/0	0/0	0/0	0/0	0/0	0/0	0/0	0/0	0/0	0/0	0/0	0/0	0/0	0/0	./.	0/0	0/0	0/0	0/0	0/0
0/0	0/0	1/1	0/0	0/0	0/0	0/0	0/0	0/0	0/0	0/0	0/0	1/1	0/0	0/0	0/0	0/0	0/0	0/0	0/0	0/0	0/0	0/0	0/0	0/0	0/0	0/0
0/0	1/1	1/1	0/0	0/0	1/1	0/0	0/0	0/0	1/1	0/0	0/0	1/1	0/0	1/1	1/1	0/0	0/0	1/1	1/1	1/1	0/0	1/1	1/1	1/1	1/1	1/1
0/0	0/0	0/0	0/0	0/0	0/0	0/0	0/0	0/0	0/0	0/0	0/0	0/0	0/0	0/0	0/0	0/0	0/0	0/0	0/0	0/0	0/0	0/0	0/0	0/0	0/0	0/0
0/0	0/0	0/0	0/0	0/0	0/0	0/0	0/0	0/0	0/0	0/0	0/0	0/0	0/0	0/0	0/0	0/0	0/0	0/0	0/0	0/0	0/0	0/0	0/0	0/0	0/0	0/0
0/0	1/1	1/1	0/0	0/0	1/1	0/0	0/0	0/0	1/1	0/0	0/0	1/1	0/0	1/1	1/1	0/0	0/0	1/1	1/1	1/1	0/0	1/1	1/1	1/1	1/1	1/1
0/0	0/0	1/1	0/0	0/0	0/0	0/0	0/0	0/0	0/0	0/0	0/0	1/1	0/0	0/0	0/0	0/0	0/0	0/0	0/0	0/0	0/0	0/0	0/0	0/0	0/0	0/0
0/0	0/0	0/0	0/0	0/0	0/0	0/0	0/0	0/0	0/0	0/0	0/0	0/0	0/0	0/0	0/0	0/0	0/0	0/0	0/0	0/0	0/0	0/0	0/0	0/0	0/0	0/0
0/0	1/1	0/0	0/0	0/0	1/1	0/0	0/0	0/0	1/1	0/0	0/0	0/0	0/0	1/1	1/1	0/0	0/0	1/1	1/1	1/1	0/0	1/1	1/1	1/1	1/1	1/1
0/0	1/1	0/0	0/0	0/0	1/1	0/0	0/0	0/0	1/1	0/0	0/0	0/0	0/0	0/0	1/1	0/0	0/0	1/1	1/1	1/1	0/0	1/1	1/1	0/0	1/1	1/1
0/0	0/0	0/0	0/0	0/0	0/0	0/0	0/0	0/0	0/0	0/0	0/0	0/0	0/0	0/0	0/0	0/0	0/0	0/0	0/0	0/0	0/0	0/0	0/0	0/0	0/0	0/0
0/0	1/1	0/0	0/0	0/0	1/1	0/0	0/0	0/0	1/1	0/0	0/0	0/0	0/0	1/1	1/1	0/0	0/0	1/1	1/1	1/1	0/0	1/1	1/1	1/1	1/1	1/1
0/0	0/0	0/0	0/0	0/0	0/0	0/0	0/0	0/0	0/0	0/0	0/0	0/0	0/0	0/0	0/0	0/0	0/0	0/0	0/0	0/0	0/0	0/0	0/0	0/0	0/0	0/0
0/0	1/1	1/1	0/0	0/0	1/1	0/0	0/0	0/0	1/1	0/0	0/0	1/1	0/0	1/1	1/1	0/0	0/0	1/1	1/1	1/1	0/0	1/1	1/1	1/1	1/1	1/1
0/0	0/0	1/1	0/0	0/0	0/0	0/0	0/0	0/0	0/0	0/0	0/0	1/1	0/0	0/0	0/0	0/0	0/0	0/0	0/0	0/0	0/0	0/0	0/0	0/0	0/0	0/0
0/0	1/1	0/0	0/0	0/0	1/1	0/0	0/0	0/0	1/1	0/0	0/0	0/0	0/0	0/0	1/1	0/0	0/0	1/1	1/1	1/1	0/0	1/1	1/1	0/0	1/1	1/1

1/1	1/1	1/1	0/0	1/1	1/1	0/0	0/0	1/1	1/1	0/0	0/0	1/1	0/0	0/1	1/1	0/0	1/1	1/1	1/1	1/1	1/1	1/1	1/1	1/1	1/1	1/1
0/0	0/0	1/1	0/0	0/0	0/0	0/0	0/0	0/0	0/0	0/0	0/0	1/1	0/0	0/0	0/0	0/0	0/0	0/0	0/0	0/0	0/0	0/0	0/0	0/0	0/0	0/0
0/0	0/0	1/1	0/0	0/0	0/0	0/0	0/0	0/0	0/0	0/0	0/0	1/1	0/0	0/0	0/0	0/0	0/0	0/0	0/0	0/0	0/0	0/0	0/0	0/0	0/0	0/0
0/0	0/0	0/0	0/0	0/0	0/0	0/0	0/0	0/0	0/0	0/0	0/0	0/0	0/0	0/0	0/0	0/0	0/0	0/0	0/0	0/0	0/0	0/0	0/0	0/0	0/0	0/0
0/0	1/1	1/1	0/0	0/0	1/1	0/0	0/0	0/0	1/1	0/0	0/0	1/1	0/0	1/1	1/1	0/0	0/0	1/1	1/1	1/1	0/0	1/1	1/1	1/1	1/1	1/1
0/0	1/1	1/1	0/0	0/0	1/1	0/0	0/0	0/0	1/1	0/0	0/0	1/1	0/0	1/1	1/1	0/0	0/0	1/1	1/1	1/1	0/0	1/1	1/1	1/1	1/1	1/1
0/0	1/1	1/1	0/0	0/0	1/1	0/0	0/0	0/0	1/1	0/0	0/0	1/1	0/0	1/1	1/1	0/0	0/0	1/1	1/1	1/1	0/0	1/1	1/1	1/1	1/1	1/1
0/0	0/0	0/0	0/0	0/0	0/0	0/0	0/0	0/0	0/0	0/0	0/0	0/0	0/0	0/0	0/0	0/0	0/0	0/0	0/0	0/0	0/0	0/0	0/0	0/0	0/0	0/0
0/0	0/0	1/1	0/0	0/0	0/0	0/0	0/0	0/0	0/0	0/0	0/0	1/1	0/0	0/0	0/0	0/0	0/0	0/0	0/0	0/0	0/0	0/0	0/0	0/0	0/0	0/0
0/0	0/0	0/0	0/0	0/0	0/0	0/0	0/0	0/0	0/0	0/0	0/0	0/0	0/0	0/0	0/0	0/0	0/0	0/0	0/0	0/0	0/0	0/0	0/0	0/0	0/0	0/0
0/0	1/1	0/0	0/0	0/0	1/1	0/0	0/0	0/0	1/1	0/0	0/0	0/0	0/0	1/1	1/1	0/0	0/0	1/1	1/1	1/1	0/0	1/1	1/1	1/1	1/1	1/1
0/0	0/0	0/0	0/0	0/0	0/0	0/0	0/0	0/0	0/0	0/0	0/0	0/0	0/0	0/0	0/0	0/0	0/0	0/0	0/0	0/0	0/0	0/0	0/0	0/0	0/0	0/0
0/0	0/0	0/0	0/0	0/0	0/0	0/0	0/0	0/0	0/0	0/0	0/0	0/0	0/0	0/0	0/0	0/0	0/0	0/0	0/0	0/0	0/0	0/0	0/0	0/0	0/0	0/0
0/0	0/0	0/0	0/0	0/0	0/0	0/0	0/0	0/0	0/0	0/0	0/0	0/0	0/0	1/1	0/0	0/0	0/0	0/0	0/0	0/0	0/0	0/0	0/0	1/1	0/0	0/0
0/0	1/1	1/1	0/0	0/0	1/1	0/0	0/0	0/0	1/1	0/0	0/0	1/1	0/0	1/1	1/1	0/0	0/0	1/1	1/1	1/1	0/0	1/1	1/1	1/1	1/1	1/1
0/0	0/0	0/0	0/0	0/0	0/0	0/0	0/0	0/0	0/0	0/0	0/0	0/0	0/0	0/0	0/0	0/0	0/0	0/0	0/0	0/0	0/0	0/0	0/0	0/0	0/0	0/0
0/0	0/0	0/0	0/0	0/0	0/0	0/0	0/0	0/0	0/0	0/0	0/0	0/0	0/0	0/0	0/0	0/0	0/0	0/0	0/0	0/0	0/0	0/0	0/0	0/0	0/0	0/0
0/0	0/0	0/0	0/0	0/0	0/0	0/0	0/0	0/0	0/0	0/0	0/0	0/0	0/0	0/0	0/0	0/0	0/0	0/0	0/0	0/0	0/0	0/0	0/0	0/0	0/0	0/0
0/0	1/1	0/0	0/0	0/0	1/1	0/0	0/0	0/0	1/1	0/0	0/0	0/0	0/0	1/1	1/1	0/0	0/0	1/1	1/1	1/1	0/0	1/1	1/1	1/1	1/1	1/1
0/0	0/0	0/0	0/0	0/0	0/0	0/0	0/0	0/0	0/0	0/0	0/0	0/0	0/0	0/0	0/0	0/0	0/0	0/0	0/0	0/0	0/0	0/0	0/0	0/0	0/0	0/0
0/0	1/1	1/1	0/0	0/0	1/1	0/0	0/0	0/0	1/1	0/0	0/0	1/1	0/0	1/1	1/1	0/0	0/0	1/1	1/1	1/1	0/0	1/1	1/1	1/1	1/1	1/1
0/0	1/1	0/0	0/0	0/0	1/1	0/0	0/0	0/0	1/1	0/0	0/0	0/0	0/0	1/1	1/1	0/0	0/0	1/1	1/1	1/1	0/0	1/1	1/1	1/1	1/1	1/1
0/0	1/1	0/0	0/0	0/0	1/1	0/0	0/0	0/0	1/1	0/0	0/0	0/0	0/0	1/1	1/1	0/0	0/0	1/1	1/1	1/1	0/0	1/1	1/1	1/1	1/1	1/1
0/0	0/0	1/1	0/0	0/0	0/0	0/0	0/0	0/0	0/0	0/0	0/0	1/1	0/0	0/0	0/0	0/0	0/0	0/0	0/0	0/0	0/0	0/0	0/0	0/0	0/0	0/0
0/0	1/1	1/1	0/0	0/0	1/1	0/0	0/0	0/0	1/1	0/0	0/0	1/1	0/0	1/1	1/1	0/0	0/0	1/1	1/1	1/1	0/0	1/1	1/1	1/1	1/1	1/1
0/0	0/0	1/1	0/0	0/0	0/0	0/0	0/0	0/0	0/0	0/0	0/0	1/1	0/0	0/0	0/0	0/0	0/0	0/0	0/0	0/0	0/0	0/0	0/0	0/0	0/0	0/0
0/0	0/0	0/0	0/0	0/0	0/0	0/0	0/0	0/0	0/0	0/0	0/0	0/0	0/0	0/0	0/0	0/0	0/0	0/0	0/0	0/0	0/0	0/0	0/0	0/0	0/0	0/0
0/0	0/0	0/0	0/0	0/0	0/0	0/0	0/0	0/0	0/0	0/0	0/0	0/0	0/0	0/0	0/0	0/0	0/0	0/0	0/0	0/0	0/0	0/0	0/0	0/0	0/0	0/0
1/1	1/1	1/1	0/0	1/1	1/1	0/0	0/0	1/1	1/1	0/0	0/0	1/1	0/0	1/1	1/1	0/0	1/1	1/1	1/1	1/1	1/1	1/1	1/1	1/1	1/1	1/1
0/0	0/0	0/0	0/0	0/0	0/0	1/1	0/0	0/0	0/0	0/0	0/0	0/0	1/1	0/0	0/0	1/1	0/0	0/0	0/0	0/0	0/0	0/0	0/0	0/0	0/0	0/0
0/0	1/1	1/1	0/0	0/0	1/1	0/0	0/0	0/0	1/1	0/0	0/0	1/1	0/0	1/1	1/1	0/0	0/0	1/1	1/1	1/1	0/0	1/1	1/1	1/1	1/1	1/1
0/0	0/0	1/1	0/0	0/0	0/0	0/0	0/0	0/0	0/0	0/0	0/0	1/1	0/0	0/0	0/0	0/0	0/0	0/0	0/0	0/0	0/0	0/0	0/0	0/0	0/0	0/0
0/0	1/1	1/1	0/0	0/0	1/1	0/0	0/0	0/0	1/1	0/0	0/0	1/1	0/0	1/1	1/1	0/0	0/0	1/1	1/1	1/1	0/0	1/1	1/1	1/1	1/1	1/1
0/0	0/0	0/0	0/0	0/0	0/0	0/0	0/0	0/0	0/0	0/0	0/0	0/0	0/0	0/0	0/0	0/0	0/0	0/0	0/0	0/0	0/0	0/0	0/0	0/0	0/0	0/0
0/0	0/0	1/1	0/0	0/0	0/0	0/0	0/0	0/0	0/0	0/0	0/0	1/1	0/0	0/0	0/0	0/0	0/0	0/0	0/0	0/0	0/0	0/0	0/0	0/0	0/0	0/0

0/0	1/1	0/0	0/0	0/0	1/1	0/0	0/0	0/0	1/1	0/0	0/0	0/0	0/0	1/1	1/1	0/0	0/0	1/1	1/1	1/1	0/0	1/1	1/1	1/1	1/1	1/1
0/0	0/0	0/0	0/0	0/0	0/0	0/0	0/0	0/0	0/0	0/0	0/0	0/0	0/0	0/0	0/0	0/0	0/0	0/0	0/0	0/0	0/0	0/0	0/0	0/0	0/0	0/0
0/0	1/1	0/0	0/0	0/0	1/1	0/0	0/0	0/0	1/1	0/0	0/0	0/0	0/0	0/0	1/1	0/0	0/0	1/1	1/1	1/1	0/0	1/1	1/1	0/0	1/1	1/1
0/0	1/1	1/1	0/0	0/0	1/1	0/0	0/0	0/0	1/1	0/0	0/0	1/1	0/0	1/1	1/1	0/0	0/0	1/1	1/1	1/1	0/0	1/1	1/1	1/1	1/1	1/1
0/0	0/0	0/0	0/0	0/0	0/0	0/0	0/0	0/0	0/0	0/0	0/0	0/0	0/0	0/0	0/0	0/0	0/0	0/0	0/0	0/0	0/0	0/0	0/0	0/0	0/0	0/0
0/0	1/1	0/0	0/0	0/0	1/1	0/0	0/0	0/0	1/1	0/0	0/0	0/0	0/0	1/1	1/1	0/0	0/0	1/1	1/1	1/1	0/0	1/1	1/1	1/1	1/1	1/1
0/0	1/1	1/1	0/0	0/0	1/1	0/0	0/0	0/0	1/1	0/0	0/0	1/1	0/0	0/1	1/1	0/0	0/0	1/1	1/1	1/1	0/0	1/1	1/1	1/1	1/1	1/1
0/0	0/0	1/1	0/0	0/0	0/0	0/0	0/0	0/0	0/0	0/0	0/0	1/1	0/0	0/0	0/0	0/0	0/0	0/0	0/0	0/0	0/0	0/0	0/0	0/0	0/0	0/0
0/0	1/1	0/0	0/0	0/0	1/1	0/0	0/0	0/0	1/1	0/0	0/0	0/0	0/0	1/1	1/1	0/0	0/0	1/1	1/1	1/1	0/0	1/1	1/1	1/1	1/1	1/1
0/0	0/0	0/0	0/0	0/0	0/0	0/0	0/0	0/0	0/0	0/0	0/0	0/0	0/0	0/0	0/0	0/0	0/0	0/0	0/0	0/0	0/0	0/0	0/0	0/0	0/0	0/0
0/0	1/1	0/0	0/0	0/0	1/1	0/0	0/0	0/0	1/1	0/0	0/0	0/0	0/0	1/1	1/1	0/0	0/0	1/1	1/1	1/1	0/0	1/1	1/1	1/1	1/1	1/1
0/0	0/0	0/0	0/0	0/0	0/0	0/0	0/0	0/0	0/0	0/0	0/0	0/0	0/0	0/0	0/0	0/0	0/0	0/0	0/0	0/0	0/0	0/0	0/0	0/0	0/0	0/0
0/0	0/0	0/0	0/0	0/0	0/0	0/0	0/0	0/0	0/0	0/0	0/0	0/0	0/0	0/0	0/0	0/0	0/0	0/0	0/0	0/0	0/0	0/0	0/0	0/0	0/0	0/0
0/0	0/0	0/0	0/0	1/1	0/0	0/0	0/0	0/0	0/0	0/0	0/0	0/0	0/0	0/0	0/0	0/0	0/0	0/0	0/0	0/0	0/0	0/0	0/0	0/0	0/0	0/0
1/1	1/1	1/1	0/0	1/1	1/1	0/0	0/0	1/1	1/1	0/0	0/0	1/1	0/0	1/1	1/1	0/0	1/1	1/1	1/1	1/1	1/1	1/1	1/1	1/1	1/1	1/1
1/1	1/1	1/1	0/0	1/1	1/1	0/0	0/0	1/1	1/1	0/0	0/0	1/1	0/0	1/1	1/1	0/0	1/1	1/1	1/1	1/1	1/1	1/1	1/1	1/1	1/1	1/1
0/0	1/1	1/1	0/0	0/0	1/1	0/0	0/0	0/0	1/1	0/0	0/0	1/1	0/0	1/1	1/1	0/0	0/0	1/1	1/1	1/1	0/0	1/1	1/1	1/1	1/1	1/1
./.	./.	0/0	0/0	./.	./.	0/0	0/0	./.	1/1	0/0	0/0	./.	0/0	./.	./.	0/0	./.	./.	1/1	./.	./.	1/1	./.	./.	./.	0/0
1/1	1/1	3/3	0/0	1/1	1/1	0/0	0/0	1/1	4/4	0/0	0/0	3/3	2/2	1/1	1/1	0/0	1/1	1/1	4/4	1/1	1/1	4/4	1/1	1/1	1/1	1/1
0/0	0/0	0/0	0/0	0/0	0/0	0/0	0/0	0/0	0/0	0/0	0/0	1/1	0/0	0/0	0/0	0/0	0/0	0/0	0/0	0/0	0/0	0/0	0/0	0/0	0/0	0/0
0/0	0/0	0/0	0/0	0/0	0/0	0/0	0/0	0/0	0/0	0/0	0/0	0/0	0/0	0/0	0/0	0/0	0/0	0/0	0/0	0/0	0/0	0/0	0/0	0/0	0/0	0/0
0/0	0/0	0/0	0/0	0/0	0/0	0/0	0/0	0/0	0/0	0/0	0/0	0/0	0/0	0/0	0/0	0/0	0/0	0/0	0/0	0/0	0/0	0/0	0/0	0/0	0/0	0/0
0/0	0/0	0/0	0/0	0/0	0/0	0/0	0/0	0/0	0/0	0/0	0/0	0/0	0/0	0/0	0/0	0/0	0/0	0/0	0/0	0/0	0/0	0/0	0/0	0/0	0/0	0/0
0/0	0/0	0/0	0/0	0/0	0/0	0/0	0/0	0/0	0/0	0/0	0/0	0/0	0/0	0/0	0/0	0/0	0/0	0/0	0/0	0/0	0/0	0/0	0/0	0/0	0/0	0/0
0/0	0/0	1/1	0/0	0/0	0/0	0/0	0/0	0/0	0/0	0/0	0/0	1/1	0/0	0/0	0/0	0/0	0/0	0/0	0/0	0/0	0/0	0/0	0/0	0/0	0/0	0/0

0/0	0/0	0/0	0/0	0/0	0/0	0/0	0/0	0/0	0/0	0/0	0/0	0/0	0/0	0/0	0/0	0/0	0/0	0/0	0/0	0/0	0/0	0/0	0/0	0/0	0/0	0/0
0/0	0/0	1/1	0/0	0/0	0/0	0/0	0/0	0/0	0/0	0/0	0/0	1/1	0/0	0/0	0/0	0/0	0/0	0/0	0/0	0/0	0/0	0/0	0/0	0/0	0/0	0/0
0/0	0/0	1/1	0/0	0/0	0/0	0/0	0/0	0/0	0/0	0/0	0/0	1/1	0/0	0/0	0/0	0/0	0/0	0/0	0/0	0/0	0/0	0/0	0/0	0/0	0/0	0/0
1/1	1/1	1/1	0/0	1/1	1/1	0/0	0/0	1/1	1/1	0/0	0/0	1/1	0/0	1/1	1/1	0/0	1/1	1/1	1/1	1/1	1/1	1/1	1/1	1/1	1/1	1/1
0/0	1/1	1/1	0/0	0/0	1/1	0/0	0/0	0/0	1/1	0/0	0/0	1/1	0/0	1/1	1/1	0/0	0/0	1/1	1/1	1/1	0/0	1/1	1/1	1/1	1/1	1/1
0/0	0/0	0/0	0/0	0/0	0/0	0/0	0/0	0/0	0/0	0/0	0/0	0/0	0/0	0/0	0/0	0/0	0/0	0/0	0/0	0/0	0/0	0/0	0/0	0/0	0/0	0/0
0/0	1/1	1/1	0/0	0/0	1/1	0/0	0/0	0/0	1/1	0/0	0/0	1/1	0/0	1/1	1/1	0/0	0/0	1/1	1/1	1/1	0/0	1/1	1/1	1/1	1/1	1/1
0/0	0/0	1/1	0/0	0/0	0/0	0/0	0/0	0/0	0/0	0/0	0/0	1/1	0/1	0/0	0/0	0/0	0/0	0/0	0/0	0/0	0/0	0/0	0/0	0/0	0/0	0/0
0/0	0/0	0/0	0/0	0/0	0/0	0/0	0/0	0/0	0/0	0/0	0/0	0/0	0/0	0/0	0/0	0/0	0/0	0/0	0/0	0/0	0/0	0/0	0/0	0/0	0/0	0/0
0/0	0/0	0/0	0/0	0/0	0/0	0/0	0/0	0/0	0/0	0/0	0/0	0/0	0/0	0/0	0/0	0/0	0/0	0/0	0/0	0/0	0/0	0/0	0/0	0/0	0/0	0/0
0/0	0/0	1/1	0/0	0/0	0/0	0/0	0/0	0/0	0/0	0/0	0/0	1/1	0/0	0/0	0/0	0/0	0/0	0/0	0/0	0/0	0/0	0/0	0/0	0/0	0/0	0/0
0/0	0/0	0/0	0/0	0/0	0/0	0/0	0/0	0/0	0/0	0/0	0/0	0/0	0/0	0/0	0/0	0/0	0/0	0/0	0/0	0/0	0/0	0/0	0/0	0/0	0/0	0/0
0/0	1/1	0/0	0/0	0/0	1/1	0/0	0/0	0/0	1/1	0/0	0/0	0/0	0/0	1/1	1/1	0/0	0/0	1/1	1/1	1/1	0/0	1/1	1/1	1/1	1/1	1/1
0/0	0/0	1/1	0/0	0/0	0/0	0/0	0/0	0/0	0/0	0/0	0/0	1/1	0/1	0/0	0/0	0/0	0/0	0/0	0/0	0/0	0/0	0/0	0/0	0/0	0/0	0/0
0/0	0/0	0/0	0/0	0/0	0/0	0/0	0/0	0/0	0/0	0/0	0/0	0/0	0/0	0/0	0/0	0/0	0/0	0/0	0/0	0/0	0/0	0/0	0/0	0/0	0/0	0/0
0/0	1/1	1/1	0/0	0/0	1/1	0/0	0/0	0/0	1/1	0/0	0/0	1/1	0/1	1/1	1/1	0/0	0/0	1/1	1/1	1/1	0/0	1/1	1/1	1/1	1/1	1/1
0/0	1/1	0/0	0/0	0/0	1/1	0/0	0/0	0/0	1/1	0/0	0/0	0/0	0/0	1/1	1/1	0/0	0/0	1/1	1/1	1/1	0/0	1/1	1/1	1/1	1/1	1/1
0/0	0/0	0/0	0/0	0/0	0/0	0/0	0/0	0/0	0/0	0/0	0/0	0/0	0/0	1/1	0/0	0/0	0/0	0/0	0/0	0/0	0/0	0/0	0/0	1/1	0/0	0/0
0/0	0/0	0/0	0/0	0/0	0/0	0/0	0/0	0/0	0/0	0/0	0/0	0/0	0/0	0/0	0/0	0/0	0/0	0/0	0/0	0/0	0/0	0/0	0/0	0/0	0/0	0/0
0/0	0/0	1/1	0/0	0/0	0/0	0/0	0/0	0/0	0/0	0/0	0/0	1/1	0/1	0/0	0/0	0/0	0/0	0/0	0/0	0/0	0/0	0/0	0/0	0/0	0/0	0/0
0/0	0/0	1/1	0/0	0/0	0/0	0/0	0/0	0/0	0/0	0/0	0/0	1/1	0/1	0/0	0/0	0/0	0/0	0/0	0/0	0/0	0/0	0/0	0/0	0/0	0/0	0/0
0/0	0/0	0/0	0/0	0/0	0/0	0/0	0/0	0/0	0/0	0/0	0/0	0/0	0/0	0/0	0/0	0/0	0/0	0/0	0/0	0/0	0/0	0/0	0/0	0/0	0/0	0/0
0/0	1/1	1/1	0/0	0/0	1/1	0/0	0/0	0/0	1/1	0/0	0/0	1/1	0/1	1/1	1/1	0/0	0/0	1/1	1/1	1/1	0/0	1/1	1/1	1/1	1/1	1/1
0/0	0/0	0/0	0/0	0/0	0/0	0/0	0/0	0/0	0/0	0/0	0/0	0/0	0/0	0/0	0/0	0/0	0/0	0/0	0/0	0/0	0/0	0/0	0/0	0/0	0/0	0/0
0/0	0/0	0/0	0/0	0/0	0/0	0/0	0/0	0/0	0/0	0/0	0/0	0/0	0/0	0/0	0/0	0/0	0/0	0/0	0/0	0/0	0/0	0/0	0/0	0/0	0/0	0/0
0/0	0/0	1/1	0/0	0/0	0/0	0/0	0/0	0/0	0/0	0/0	0/0	1/1	0/0	0/0	0/0	0/0	0/0	0/0	0/0	0/0	0/0	0/0	0/0	0/0	0/0	0/0
0/0	0/0	0/0	0/0	0/0	0/0	0/0	0/0	0/0	0/0	0/0	0/0	0/0	0/0	0/0	0/0	0/0	0/0	0/0	0/0	0/0	0/0	0/0	0/0	0/0	0/0	0/0
0/0	1/1	0/0	0/0	0/0	1/1	0/0	0/0	0/0	1/1	0/0	0/0	0/0	0/0	1/1	1/1	0/0	0/0	1/1	1/1	1/1	0/0	1/1	1/1	1/1	1/1	1/1
0/0	0/0	1/1	0/0	0/0	0/0	0/0	0/0	0/0	0/0	0/0	0/0	1/1	0/1	0/0	0/0	0/0	0/0	0/0	0/0	0/0	0/0	0/0	0/0	0/0	0/0	0/0
0/0	0/0	0/0	0/0	0/0	0/0	0/0	0/0	0/0	0/0	0/0	0/0	0/0	0/0	0/0	0/0	0/0	0/0	0/0	0/0	0/0	0/0	0/0	0/0	0/0	0/0	0/0
0/0	1/1	1/1	0/0	0/0	1/1	0/0	0/0	0/0	1/1	0/0	0/0	1/1	0/0	1/1	1/1	0/0	0/0	1/1	1/1	1/1	0/0	1/1	1/1	1/1	1/1	1/1
0/0	0/0	0/0	0/0	0/0	0/0	0/0	0/0	0/0	0/0	0/0	0/0	0/0	0/0	0/0	0/0	0/0	0/0	0/0	0/0	0/0	0/0	0/0	0/0	0/0	0/0	0/0
1/1	0/0	0/0	0/0	1/1	0/0	0/0	0/0	1/1	0/0	0/0	0/0	0/0	0/0	0/0	0/0	1/1	1/1	0/0	0/0	0/0	1/1	0/0	0/0	0/0	0/0	0/0

0/0	1/1	1/1	0/0	0/0	1/1	0/0	0/0	0/0	1/1	0/0	0/0	1/1	0/0	1/1	1/1	0/0	0/0	1/1	1/1	1/1	0/0	1/1	1/1	1/1	1/1	1/1
0/0	0/0	0/0	0/0	0/0	0/0	0/0	0/0	0/0	0/0	0/0	0/0	0/0	0/0	0/0	0/0	0/0	0/0	0/0	0/0	0/0	0/0	0/0	0/0	0/0	0/0	0/0
0/0	1/1	1/1	0/0	0/0	1/1	0/0	0/0	0/0	1/1	0/0	0/0	1/1	0/0	1/1	1/1	0/0	0/0	1/1	1/1	1/1	0/0	1/1	1/1	1/1	1/1	1/1
0/0	0/0	0/0	0/0	0/0	0/0	0/0	0/0	0/0	0/0	0/0	0/0	0/0	0/0	0/0	0/0	0/0	0/0	0/0	0/0	0/0	0/0	0/0	0/0	0/0	0/0	0/0
0/0	1/1	0/0	0/0	0/0	1/1	0/0	0/0	0/0	1/1	0/0	0/0	0/0	0/0	0/0	1/1	0/0	0/0	1/1	1/1	1/1	0/0	1/1	1/1	0/0	1/1	1/1
0/0	0/0	0/0	0/0	0/0	0/0	0/0	0/0	0/0	0/0	0/0	0/0	0/0	0/0	0/0	0/0	0/0	0/0	0/0	0/0	0/0	0/0	0/0	0/0	0/0	0/0	0/0
0/0	0/0	0/0	0/0	0/0	0/0	0/0	0/0	0/0	0/0	0/0	0/0	0/0	0/0	0/0	0/0	0/0	0/0	0/0	0/0	0/0	0/0	0/0	0/0	0/0	0/0	0/0
0/0	0/0	0/0	0/0	0/0	0/0	0/0	0/0	0/0	0/0	0/0	0/0	0/0	0/0	1/1	0/0	0/0	0/0	0/0	0/0	0/0	0/0	0/0	0/0	1/1	0/0	0/0
0/0	0/0	0/0	0/0	0/0	0/0	0/0	0/0	0/0	0/0	0/0	0/0	0/0	0/0	0/0	0/0	0/0	0/0	0/0	0/0	0/0	0/0	0/0	0/0	0/0	0/0	0/0
0/0	0/0	0/0	0/0	0/0	0/0	0/0	0/0	0/0	0/0	0/0	0/0	0/0	0/0	0/0	0/0	0/0	0/0	0/0	0/0	0/0	0/0	0/0	0/0	0/0	0/0	0/0
0/0	1/1	1/1	0/0	0/0	1/1	0/0	0/0	0/0	1/1	0/0	0/0	1/1	0/0	1/1	1/1	0/0	0/0	1/1	1/1	1/1	0/0	1/1	1/1	1/1	1/1	1/1
0/0	1/1	1/1	0/0	0/0	1/1	0/0	0/0	0/0	1/1	0/0	0/0	1/1	0/0	1/1	1/1	0/0	0/0	1/1	1/1	1/1	0/0	1/1	1/1	1/1	1/1	1/1
0/0	0/0	0/0	0/0	0/0	0/0	0/0	0/0	0/0	0/0	0/0	0/0	0/0	0/0	0/0	0/0	0/0	0/0	0/0	0/0	0/0	0/0	0/0	0/0	0/0	0/0	0/0
0/0	1/1	0/0	0/0	0/0	1/1	0/0	0/0	0/0	1/1	0/0	0/0	0/0	0/0	0/0	1/1	0/0	0/0	1/1	1/1	1/1	0/0	1/1	1/1	0/0	1/1	1/1
0/0	0/0	1/1	0/0	0/0	0/0	0/0	0/0	0/0	0/0	0/0	0/0	1/1	0/0	0/0	0/0	0/0	0/0	0/0	0/0	0/0	0/0	0/0	0/0	0/0	0/0	0/0
2/2	1/1	1/1	0/0	2/2	1/1	0/0	0/0	2/2	1/1	0/0	0/0	1/2	0/0	1/1	1/1	1/1	2/2	1/1	1/1	1/1	2/2	1/1	1/1	1/1	1/1	1/1
0/0	1/1	1/1	0/0	0/0	1/1	0/0	0/0	0/0	1/1	0/0	0/0	1/1	0/0	1/1	1/1	0/0	0/0	1/1	1/1	1/1	0/0	1/1	1/1	1/1	1/1	1/1
0/0	0/0	0/0	0/0	0/0	0/0	0/0	0/0	0/0	0/0	0/0	0/0	1/1	0/0	0/0	0/0	0/0	0/0	0/0	0/0	0/0	0/0	0/0	0/0	0/0	0/0	0/0
0/0	0/0	0/0	0/0	0/0	0/0	0/0	0/0	0/0	0/0	0/0	0/0	1/1	0/0	0/0	0/0	0/0	0/0	0/0	0/0	0/0	0/0	0/0	0/0	0/0	0/0	1/1
0/0	1/1	1/1	0/0	0/0	1/1	0/0	0/0	0/0	1/1	0/0	0/0	0/0	0/0	0/0	1/1	0/0	0/0	1/1	1/1	1/1	0/0	1/1	1/1	0/0	1/1	0/0
0/0	0/0	0/0	0/0	0/0	0/0	0/0	0/0	0/0	0/0	0/0	0/0	0/0	0/0	0/0	0/0	0/0	0/0	0/0	0/0	0/0	0/0	0/0	0/0	0/0	0/0	0/0
0/0	0/0	0/0	0/0	0/0	0/0	0/0	0/0	0/0	0/0	0/0	0/0	0/0	0/0	0/0	0/0	0/0	0/0	0/0	0/0	0/0	0/0	0/0	0/0	0/0	0/0	0/0
0/0	0/0	0/0	0/0	0/0	0/0	0/0	0/0	0/0	0/0	0/0	0/0	0/0	0/0	0/0	0/0	0/0	0/0	0/0	0/0	0/0	0/0	0/0	0/0	0/0	0/0	0/0
0/0	0/0	0/0	0/0	0/0	0/0	0/0	0/0	0/0	0/0	0/0	0/0	0/0	0/0	0/0	0/0	0/0	0/0	0/0	0/0	0/0	0/0	0/0	0/0	0/0	0/0	0/0
0/0	0/0	0/0	0/0	0/0	0/0	0/0	0/0	0/0	0/0	0/0	0/0	0/0	0/0	0/0	0/0	0/0	0/0	0/0	0/0	0/0	0/0	0/0	0/0	0/0	0/0	0/0
0/0	0/0	0/0	0/1	0/0	0/0	0/0	0/0	0/0	0/0	0/0	0/0	0/0	0/0	0/0	0/0	0/0	0/0	0/0	0/0	0/1	0/0	0/0	0/0	0/1	0/0	0/0

0/0	0/0	0/0	0/1	0/0	0/0	0/0	0/0	0/0	0/0	0/0	0/0	0/0	0/0	0/0	0/0	0/0	0/0	0/0	0/0	0/1	0/0	0/0	0/0	0/0	0/0	0/0
0/0	0/0	0/0	0/1	0/0	0/0	0/0	0/0	0/0	0/0	0/0	0/0	0/0	0/0	0/0	0/0	0/0	0/0	0/0	0/0	0/1	0/0	0/0	0/0	0/1	0/0	0/0
0/0	0/0	0/0	0/1	0/0	0/0	0/0	0/0	0/0	0/0	0/0	0/0	0/0	0/0	0/0	0/0	0/0	0/0	0/0	0/0	0/1	0/0	0/0	0/0	0/0	0/0	0/0
0/0	0/0	0/0	0/1	0/0	0/0	0/0	0/0	0/0	0/0	0/0	0/0	0/0	0/0	0/0	0/0	0/0	0/0	0/0	0/0	0/1	0/0	0/0	0/0	0/0	0/0	0/0
0/0	0/0	0/0	0/1	0/0	0/0	0/0	0/0	0/0	0/0	0/0	0/0	0/0	0/0	0/0	0/0	0/0	0/0	0/0	0/0	0/0	0/0	0/0	0/0	0/0	0/0	0/0
0/0	0/0	0/0	0/0	0/0	0/0	0/0	0/0	0/0	0/0	0/0	0/0	0/0	0/0	0/0	0/0	0/0	0/0	0/0	0/0	0/0	0/0	0/0	0/0	0/0	0/0	0/0
0/0	0/0	0/0	0/0	0/0	0/0	0/0	0/0	0/0	0/0	0/0	0/0	0/0	0/0	0/0	0/0	0/0	0/0	0/0	0/0	0/0	0/0	0/0	0/0	0/0	0/0	0/0
0/0	0/0	0/0	0/0	0/0	0/0	0/0	0/0	0/0	0/0	0/0	0/0	0/0	0/0	0/0	0/0	0/0	0/0	0/0	0/0	0/0	0/0	0/0	0/0	0/0	0/0	0/0
0/0	0/0	0/0	0/0	0/0	0/0	0/0	0/0	0/0	0/0	0/0	0/0	0/0	0/0	0/0	0/0	0/0	0/0	0/0	0/0	0/0	0/0	0/0	0/0	0/0	0/0	0/0
0/0	0/0	0/0	0/0	0/0	0/0	0/0	0/0	0/0	0/0	0/0	0/0	0/0	0/0	0/0	0/0	0/0	0/0	0/0	0/0	0/0	0/0	0/0	0/0	0/0	0/0	0/0
0/0	1/1	1/1	0/0	1/1	1/1	0/0	0/0	0/0	1/1	0/0	0/0	1/1	0/0	1/1	1/1	1/1	0/0	1/1	1/1	1/1	1/1	1/1	1/1	1/1	1/1	1/1
0/0	0/0	0/0	0/0	0/0	0/0	0/0	0/0	0/0	0/0	0/0	0/0	0/0	0/0	0/0	0/0	0/0	0/0	0/0	0/0	0/0	0/0	0/0	0/0	0/0	0/0	0/0
0/0	0/0	0/0	0/0	0/0	0/0	0/0	0/0	0/0	0/0	0/0	0/0	0/0	0/0	0/0	0/0	0/0	0/0	0/0	0/0	0/0	0/0	0/0	0/0	0/0	0/0	0/0
0/0	0/0	0/0	0/0	0/0	0/0	0/0	0/0	0/0	0/0	0/0	0/0	0/0	0/0	1/1	0/0	0/0	0/0	0/0	0/0	0/0	0/0	0/0	0/0	1/1	0/0	0/0
0/0	0/0	0/0	0/0	0/0	0/0	0/0	0/0	0/0	0/0	0/0	0/0	0/0	0/0	0/0	0/0	0/0	0/0	0/0	0/0	0/0	0/0	0/0	0/0	0/0	0/0	0/0
0/0	0/0	0/0	0/0	0/0	0/0	0/0	0/0	0/0	0/0	0/0	0/0	0/0	0/0	0/0	0/0	0/0	0/0	0/0	0/0	0/0	0/0	0/0	0/0	0/0	0/0	0/0
0/0	1/1	1/1	0/0	0/0	1/1	0/0	0/0	0/0	1/1	0/0	0/0	1/1	0/1	1/1	1/1	0/0	0/0	1/1	1/1	1/1	0/0	0/0	1/1	1/1	1/1	1/1
0/0	0/0	./.	0/0	0/0	0/0	0/0	0/0	0/0	0/0	0/0	0/0	./.	0/0	0/0	0/0	0/0	0/0	./.	0/0	0/0	0/0	0/0	0/0	0/0	0/0	0/0
0/0	1/1	1/1	0/0	0/0	1/1	0/0	0/0	0/0	1/1	0/0	0/0	./.	0/0	1/1	1/1	0/0	0/0	1/1	1/1	1/1	0/0	1/1	1/1	1/1	1/1	0/0
0/0	0/0	0/0	0/0	0/0	0/0	0/0	0/0	0/0	0/0	0/0	0/0	1/1	0/0	0/0	0/0	0/0	0/0	0/0	0/0	0/0	0/0	0/0	0/0	0/0	0/0	1/1
0/0	0/0	0/0	0/0	0/0	0/0	0/0	0/0	0/0	0/0	0/0	0/0	1/1	0/0	0/0	0/0	0/0	0/0	0/0	0/0	0/0	0/0	0/0	0/0	0/0	0/0	1/1
0/0	./.	./.	0/0	0/0	./.	0/0	0/0	0/0	./.	0/0	0/0	0/0	0/0	./.	0/0	0/0	0/0	./.	./.	./.	0/0	0/0	0/0	0/0	0/0	0/0
0/0	./.	./.	0/0	./.	./.	0/0	0/0	0/0	./.	0/0	0/0	0/0	./.	./.	./.	0/0	0/0	./.	./.	0/0	0/0	0/0	0/0	./.	./.	0/0
0/0	1/1	1/1	0/0	0/0	1/1	0/0	0/0	0/0	1/1	0/0	0/0	0/0	./.	1/1	1/1	0/0	0/0	1/1	1/1	0/1	0/0	0/1	0/1	1/1	1/1	0/0

0/0	2/2	2/2	0/0	0/0	2/2	0/0	0/0	0/0	2/2	0/0	0/0	0/0	./.	2/2	2/2	0/0	0/0	2/2	0/0	0/0	0/0	0/0	0/0	2/2	2/2	0/0
0/0	4/4	4/4	0/0	3/3	4/4	1/1	0/0	0/0	4/4	0/0	0/0	0/0	1/1	4/4	4/4	3/3	0/0	4/4	4/4	0/0	3/3	0/0	0/0	4/4	4/4	0/0
0/0	0/0	0/0	0/0	0/0	0/0	0/0	0/0	0/0	0/0	0/0	0/0	0/0	0/0	0/0	0/0	0/0	0/0	0/0	0/0	0/0	0/0	0/0	0/0	0/0	0/0	0/0
0/0	1/1	1/1	0/0	0/0	1/1	0/0	0/0	0/0	1/1	0/0	0/0	1/1	0/0	1/1	1/1	0/0	0/0	1/1	1/1	1/1	0/0	1/1	1/1	1/1	1/1	1/1
0/0	1/1	1/1	0/0	0/0	1/1	0/0	0/0	0/0	1/1	0/0	0/0	0/0	0/0	1/1	1/1	0/0	0/0	1/1	1/1	1/1	0/0	1/1	1/1	1/1	1/1	0/0
0/0	0/0	0/0	0/0	0/0	0/0	0/0	0/0	0/0	0/0	0/0	0/0	0/0	0/0	0/0	0/0	0/0	0/0	0/0	0/0	0/0	0/0	0/0	0/0	0/0	0/0	0/0
0/0	0/0	0/0	0/0	0/0	0/0	0/0	0/0	0/0	0/0	0/0	0/0	0/0	0/0	0/0	0/0	0/0	0/0	0/0	0/0	0/0	0/0	0/0	0/0	0/0	0/0	0/0
0/0	1/1	1/1	0/0	0/0	1/1	0/0	0/0	0/0	1/1	0/0	0/0	1/1	0/0	1/1	1/1	0/0	0/0	1/1	1/1	1/1	0/0	1/1	1/1	1/1	1/1	1/1
0/0	0/0	./.	0/0	0/0	./.	0/0	0/0	0/0	0/0	0/0	0/0	0/0	0/0	./.	0/0	0/0	0/0	0/0	0/0	0/0	0/0	0/0	0/0	0/0	./.	0/0
0/0	1/1	1/1	0/0	0/0	1/1	0/0	0/0	0/0	1/1	0/0	0/0	0/0	0/0	1/1	1/1	0/0	0/0	1/1	1/1	1/1	0/0	1/1	1/1	1/1	1/1	0/0
0/0	1/1	1/1	0/0	0/0	1/1	0/0	0/0	0/0	1/1	0/0	0/0	0/0	0/0	1/1	1/1	0/0	0/0	1/1	1/1	1/1	0/0	1/1	1/1	1/1	1/1	0/0
0/0	1/1	1/1	0/0	0/0	1/1	0/0	0/0	0/0	1/1	0/0	0/0	0/0	0/0	1/1	1/1	0/0	0/0	1/1	1/1	1/1	0/0	1/1	1/1	1/1	1/1	0/0
0/0	0/0	0/0	0/0	0/0	0/0	0/0	0/0	0/0	0/0	0/0	0/0	1/1	0/0	0/0	0/0	0/0	0/0	0/0	0/0	0/0	0/0	0/0	0/0	0/0	0/0	1/1
0/0	1/1	1/1	0/0	0/0	1/1	0/0	0/0	0/0	1/1	0/0	0/0	0/0	0/0	1/1	0/1	0/0	0/0	1/1	1/1	1/1	0/0	1/1	1/1	1/1	1/1	0/0
0/0	1/1	1/1	0/0	0/0	1/1	0/0	0/0	0/0	1/1	0/0	0/0	1/1	0/0	1/1	0/1	0/0	0/0	1/1	1/1	1/1	0/0	1/1	1/1	1/1	1/1	1/1
0/0	0/0	0/0	0/0	0/0	0/0	0/0	0/0	0/0	0/0	0/0	0/0	0/0	0/0	0/0	0/0	0/0	0/0	0/0	0/0	0/0	0/0	0/0	0/0	0/0	0/0	0/0
0/0	0/0	0/0	0/0	0/0	0/0	0/0	0/0	0/0	0/0	0/0	0/0	0/0	0/0	0/0	0/0	0/0	0/0	0/0	0/0	0/0	0/0	0/0	0/0	0/0	0/0	0/0
0/0	0/0	0/0	0/0	0/0	0/0	0/0	0/0	0/0	0/0	0/0	0/0	1/1	0/0	0/0	0/0	0/0	0/0	0/0	0/0	0/0	0/0	0/0	0/0	0/0	0/0	1/1
0/0	0/0	0/0	0/0	0/0	0/0	0/0	0/0	0/0	0/0	0/0	0/0	0/0	0/0	0/0	0/0	0/0	0/0	0/0	0/0	0/0	0/0	0/0	0/0	0/0	0/0	0/0
0/0	0/0	0/0	0/0	0/0	0/0	0/0	0/0	0/0	0/0	0/0	0/0	1/1	0/0	0/0	0/0	0/0	0/0	0/0	0/0	0/0	0/0	0/0	0/0	0/0	0/0	1/1
0/0	0/0	0/0	0/0	0/0	0/0	0/0	0/0	0/0	0/0	0/0	0/0	0/0	0/0	1/1	0/0	0/0	0/0	0/0	0/0	0/0	0/0	0/0	0/0	1/1	0/0	0/0
0/0	0/0	0/0	0/0	0/0	0/0	0/0	0/0	0/0	0/0	0/0	0/0	1/1	0/0	0/0	0/0	0/0	0/0	0/0	0/0	0/0	0/0	0/0	0/0	0/0	0/0	1/1
0/0	0/0	0/0	0/0	0/0	0/0	0/0	0/0	0/0	0/0	0/0	0/0	1/1	0/0	0/0	0/0	0/0	0/0	0/0	0/0	0/0	0/0	0/0	0/0	0/0	0/0	1/1
0/0	0/0	0/0	0/0	0/0	0/0	0/0	0/0	0/0	0/0	0/0	0/0	0/0	0/0	0/0	0/0	0/0	0/0	0/0	0/0	0/0	0/0	0/0	0/0	0/0	0/0	0/0
0/0	0/0	0/0	0/0	0/0	0/0	0/0	0/0	0/0	0/0	0/0	0/0	0/0	0/0	0/0	0/0	0/0	0/0	0/0	0/0	0/0	0/0	0/0	0/0	0/0	0/0	0/0
0/0	1/1	1/1	0/0	0/0	1/1	0/0	0/0	0/0	1/1	0/0	0/0	0/0	0/0	1/1	1/1	0/0	0/0	1/1	1/1	1/1	0/0	1/1	1/1	1/1	1/1	0/0
0/0	0/0	0/0	0/0	0/0	0/0	0/0	0/0	0/0	0/0	0/0	0/0	0/0	0/0	0/0	0/0	0/0	0/0	0/0	0/0	0/0	0/0	0/0	0/0	0/0	0/0	0/0
0/0	1/1	1/1	0/0	0/0	1/1	0/0	0/0	0/0	1/1	0/0	0/0	1/1	0/0	1/1	1/1	0/0	0/0	1/1	1/1	1/1	0/0	1/1	1/1	1/1	1/1	1/1
0/0	0/0	0/0	0/0	0/0	0/0	0/0	0/0	0/0	0/0	0/0	0/0	0/0	0/0	0/0	0/0	0/0	0/0	0/0	0/0	0/0	0/0	0/0	0/0	0/0	0/0	0/0
0/0	1/1	1/1	0/0	0/0	1/1	0/0	0/0	0/0	1/1	0/0	0/0	1/1	0/0	1/1	1/1	0/0	0/0	1/1	1/1	1/1	0/0	1/1	1/1	1/1	1/1	1/1

0/0	0/0	0/0	0/0	0/0	0/0	0/0	0/0	0/0	0/0	0/0	0/0	0/0	0/0	0/0	0/0	0/0	0/0	0/0	0/0	0/0	0/0	0/0	0/0	0/0	0/0	0/0
0/0	0/0	0/0	0/0	0/0	0/0	0/0	0/0	0/0	0/0	0/0	0/0	0/0	0/0	0/0	0/0	0/0	0/0	0/0	0/0	0/0	0/0	0/0	0/0	0/0	0/0	0/0
0/0	0/0	0/0	0/0	0/0	0/0	0/0	0/0	0/0	0/0	0/0	0/0	0/0	0/0	0/0	0/0	0/0	0/0	0/0	0/0	0/0	0/0	0/0	0/0	0/0	0/0	0/0
0/0	1/1	1/1	0/0	0/0	1/1	0/0	0/0	0/0	1/1	0/0	0/0	0/0	0/0	0/0	1/1	0/0	0/0	1/1	1/1	1/1	0/0	1/1	1/1	0/0	1/1	0/0
0/0	1/1	1/1	0/0	0/0	1/1	0/0	0/0	0/0	1/1	0/0	0/0	0/0	0/0	1/1	1/1	0/0	0/0	1/1	1/1	1/1	0/0	1/1	1/1	1/1	1/1	0/0
0/0	1/1	1/1	0/0	0/0	1/1	0/0	0/0	0/0	1/1	0/0	0/0	1/1	0/0	1/1	1/1	0/0	0/0	1/1	1/1	1/1	0/0	1/1	1/1	1/1	1/1	1/1
0/0	1/1	1/1	0/0	0/0	1/1	0/0	0/0	0/0	1/1	0/0	0/0	0/0	0/0	1/1	1/1	0/0	0/0	1/1	1/1	1/1	0/0	1/1	1/1	1/1	1/1	0/0
0/0	0/0	0/0	0/0	0/0	0/0	0/0	0/0	0/0	0/0	0/0	0/0	0/0	0/0	1/1	0/0	0/0	0/0	0/0	0/0	0/0	0/0	0/0	0/0	1/1	0/0	0/0
0/0	0/0	0/0	0/0	0/0	0/0	0/0	0/0	0/0	0/0	1/1	0/0	0/0	0/0	0/0	0/0	0/0	0/0	0/0	0/0	0/0	0/0	0/0	0/0	0/0	0/0	0/0
0/0	0/0	0/0	0/0	0/0	0/0	0/0	0/0	0/0	0/0	0/0	0/0	0/0	0/0	0/0	0/0	0/0	0/0	0/0	0/0	0/0	0/0	0/0	0/0	0/0	0/0	0/0
0/0	0/0	0/0	0/0	0/0	0/0	0/0	0/0	0/0	0/0	0/0	0/0	0/0	0/0	0/0	0/0	0/0	0/0	0/0	0/0	0/0	0/0	0/0	0/0	0/0	0/0	0/0
0/0	0/0	0/0	0/0	0/0	0/0	0/0	0/0	0/0	0/0	0/0	0/0	1/1	0/0	0/0	0/0	0/0	0/0	0/0	0/0	0/0	0/0	0/0	0/0	0/0	0/0	1/1
0/0	1/1	1/1	0/0	0/0	1/1	0/0	0/0	0/0	1/1	0/0	0/0	1/1	0/0	0/0	1/1	0/0	0/0	1/1	1/1	1/1	0/0	1/1	1/1	0/0	1/1	1/1
0/0	1/1	1/1	0/0	0/0	1/1	0/0	0/0	0/0	1/1	0/0	0/0	1/1	0/0	1/1	1/1	0/0	0/0	1/1	1/1	1/1	0/0	1/1	1/1	1/1	1/1	1/1
1/1	1/1	1/1	1/1	1/1	1/1	1/1	1/1	1/1	1/1	1/1	1/1	1/1	1/1	1/1	1/1	1/1	1/1	1/1	1/1	1/1	1/1	1/1	1/1	1/1	1/1	1/1
0/0	0/0	0/0	0/0	0/0	0/0	0/0	0/0	0/0	0/0	0/0	0/0	0/0	0/0	0/0	0/0	0/0	0/0	0/0	0/0	0/0	0/0	0/0	0/0	0/0	0/0	0/0
0/0	1/1	1/1	0/0	0/0	1/1	0/0	0/0	0/0	1/1	0/0	0/0	0/0	0/0	0/0	1/1	0/0	0/0	1/1	1/1	1/1	0/0	1/1	1/1	0/0	1/1	0/0
0/0	1/1	1/1	0/0	0/0	1/1	0/0	0/0	0/0	1/1	0/0	0/0	0/0	0/0	1/1	1/1	0/0	0/0	1/1	1/1	1/1	0/0	1/1	1/1	1/1	1/1	0/0
0/0	1/1	1/1	0/0	0/0	1/1	0/0	0/0	0/0	1/1	0/0	0/0	1/1	0/0	1/1	1/1	0/0	0/0	1/1	1/1	1/1	0/0	1/1	1/1	1/1	1/1	1/1
0/0	0/0	0/0	0/0	0/0	0/0	0/0	0/0	0/0	0/0	0/0	0/0	0/0	0/0	0/0	0/0	0/0	0/0	0/0	0/0	0/0	0/0	0/0	0/0	0/0	0/0	0/0
0/0	0/0	0/0	0/0	0/0	0/0	0/0	0/0	0/0	0/0	0/0	0/0	0/0	0/0	0/0	0/0	0/0	0/0	0/0	0/0	0/0	0/0	0/0	0/0	0/0	0/0	0/0
0/0	0/0	0/0	0/0	0/0	0/0	0/0	0/0	0/0	0/0	0/0	0/0	0/0	0/0	0/0	0/0	0/0	0/0	0/0	0/0	0/0	0/0	0/0	0/0	0/0	0/0	0/0
0/0	1/1	1/1	0/0	0/0	1/1	0/0	0/0	0/0	1/1	0/0	0/0	1/1	0/0	1/1	1/1	0/0	0/0	1/1	1/1	1/1	0/0	1/1	1/1	1/1	1/1	1/1
0/0	0/0	0/0	0/0	0/0	0/0	0/0	0/0	0/0	0/0	0/0	0/0	0/0	0/0	0/0	0/0	0/0	0/0	0/0	0/0	0/0	0/0	0/0	0/0	0/0	0/0	0/0
0/0	0/0	0/0	0/0	0/0	0/0	0/0	0/0	0/0	0/0	0/0	0/0	0/0	0/0	0/0	0/0	0/0	0/0	0/0	0/0	0/0	0/0	0/0	0/0	0/0	0/0	0/0
0/0	0/0	0/0	0/0	0/0	0/0	0/0	0/0	0/0	0/0	0/0	0/0	0/0	0/0	0/0	0/0	0/0	0/0	0/0	0/0	0/0	0/0	0/0	0/0	0/0	0/0	0/0
0/0	0/0	0/0	0/0	0/0	0/0	0/0	0/0	0/0	0/0	0/0	0/0	0/0	0/0	0/0	0/0	0/0	0/0	0/0	0/0	0/0	0/0	0/0	0/0	0/0	0/0	0/0
0/0	0/0	0/0	0/0	0/0	0/0	0/0	0/0	0/0	0/0	0/0	0/0	0/0	0/0	0/0	0/0	0/0	0/0	0/0	0/0	0/0	0/0	0/0	0/0	0/0	0/0	0/0
0/0	0/0	0/0	0/0	0/0	0/0	0/0	0/0	0/0	0/0	0/0	0/0	0/0	0/0	0/0	0/0	0/0	0/0	0/0	0/0	0/0	0/0	0/0	0/0	0/0	0/0	0/0
0/0	0/0	0/0	0/0	0/0	0/0	0/0	0/0	0/0	0/0	0/0	0/0	0/0	0/0	0/0	0/0	0/0	0/0	0/0	0/0	0/0	0/0	0/0	0/0	0/0	0/0	0/0
0/0	0/0	0/0	0/0	0/0	0/0	0/0	0/0	0/0	0/0	0/0	0/0	0/0	0/0	0/0	0/0	0/0	0/0	0/0	0/0	0/0	0/0	0/0	0/0	0/0	0/0	0/0
0/0	0/0	0/0	0/0	0/0	0/0	0/0	0/0	0/0	0/0	0/0	0/0	1/1	0/0	0/0	0/0	0/0	0/0	0/0	0/0	0/0	0/0	0/0	0/0	0/0	0/0	1/1
0/0	0/0	0/0	0/0	0/0	0/0	0/0	0/0	0/0	0/0	0/0	0/0	0/0	0/0	0/0	0/0	0/0	0/0	0/0	0/0	0/0	0/0	0/0	0/0	0/0	0/0	0/0
0/0	0/0	0/0	0/0	0/0	0/0	0/0	0/0	0/0	0/0	0/0	0/0	0/0	0/0	0/0	0/0	0/0	0/0	0/0	0/0	0/0	0/0	0/0	0/0	0/0	0/0	0/0
0/0	1/1	1/1	0/0	0/0	1/1	0/0	0/0	0/0	0/0	0/0	0/0	0/0	0/0	0/0	1/1	0/0	0/0	1/1	0/0	1/1	0/0	0/0	1/1	0/0	1/1	0/0

0/0	0/0	0/0	0/0	0/0	0/0	0/0	0/0	0/0	0/0	0/0	0/0	0/0	0/0	1/1	0/0	0/0	0/0	0/0	0/0	0/0	0/0	0/0	0/0	1/1	0/0	0/0
3/3	2/2	2/2	4/4	1/1	2/2	3/3	4/4	3/3	2/2	4/4	4/4	2/2	3/3	4/4	2/2	3/3	3/3	2/3	2/3	2/2	2/2	2/2	2/2	4/4	2/2	2/2
./.	./.	./.	./.	./.	./.	./.	./.	./.	./.	./.	./.	./.	./.	./.	./.	./.	./.	./.	./.	./.	./.	./.	./.	./.	./.	./.
0/0	0/0	0/0	0/0	0/0	0/0	0/0	0/0	0/0	0/0	0/0	0/0	0/0	0/0	0/0	0/0	0/0	0/0	0/0	0/0	0/0	0/0	0/0	0/0	0/0	0/0	0/0
0/0	0/0	0/0	0/0	0/0	0/0	0/0	0/0	0/0	0/0	0/0	0/0	0/0	0/0	0/0	0/0	0/0	0/0	0/0	0/0	0/0	0/0	0/0	0/0	0/0	0/0	0/0
0/0	0/0	0/0	0/0	0/0	0/0	0/0	0/0	0/0	0/0	0/0	0/0	0/0	0/0	0/0	0/0	0/0	0/0	0/0	0/0	0/0	0/0	0/0	0/0	0/0	0/0	0/0
0/0	0/0	0/0	0/0	0/0	0/0	0/0	0/0	0/0	0/0	0/0	0/0	0/0	0/0	1/1	0/0	0/0	0/0	0/0	0/0	0/0	0/0	0/0	0/0	1/1	0/0	0/0
0/0	0/0	./.	0/0	0/0	0/0	0/0	0/0	0/0	0/0	0/0	0/0	0/0	0/0	0/1	0/0	0/0	0/0	0/0	0/0	0/0	0/0	0/0	0/0	1/1	0/0	0/0
0/0	./.	./.	0/0	0/0	0/0	0/0	0/0	0/0	./.	0/0	0/0	0/0	0/0	0/0	0/0	0/0	0/0	0/0	0/0	0/0	0/0	0/0	0/0	0/0	./.	0/0
./.	1/1	1/1	0/0	0/0	1/1	./.	0/0	./.	1/1	0/0	0/0	./.	./.	0/1	0/1	./.	./.	1/1	1/1	1/1	./.	1/1	1/1	1/1	1/1	0/0
1/1	./.	./.	0/0	1/1	0/0	1/1	0/0	1/1	./.	0/0	0/0	2/2	1/1	0/1	./.	1/1	1/1	./.	./.	./.	1/1	./.	0/0	./.	./.	2/2
2/2	0/0	./.	0/0	2/2	0/0	2/2	0/0	2/2	0/0	0/0	0/0	./.	2/2	0/2	0/0	2/2	2/2	./.	0/0	0/0	2/2	0/0	0/0	./.	./.	./.
0/0	0/0	0/0	0/0	0/0	0/0	0/0	0/0	0/0	0/0	0/0	0/0	0/0	0/0	0/0	0/0	0/0	0/0	0/0	0/0	0/0	0/0	0/0	0/0	0/0	0/0	0/0
0/0	0/0	0/0	0/0	0/0	0/0	0/0	0/0	0/0	0/0	0/0	0/0	0/0	0/0	0/0	0/0	0/0	0/0	0/0	0/0	0/0	0/0	0/0	0/0	0/0	0/0	0/0
0/0	0/0	0/0	0/0	0/0	0/0	0/0	0/0	0/0	0/0	0/0	0/0	0/0	0/0	0/0	0/0	0/0	0/0	0/0	0/0	0/0	0/0	0/0	0/0	0/0	0/0	0/0
0/0	1/1	1/1	0/0	0/0	1/1	0/0	0/0	0/0	1/1	0/0	0/0	1/1	0/0	0/1	0/1	0/0	0/0	1/1	1/1	1/1	0/0	1/1	1/1	1/1	1/1	1/1
0/0	0/0	0/0	0/0	0/0	0/0	0/0	0/0	0/0	0/0	0/0	0/0	0/0	0/0	0/0	0/0	0/0	0/0	0/0	0/0	0/0	0/0	0/0	0/0	0/0	0/0	0/0
0/0	1/1	1/1	0/0	0/0	1/1	0/0	0/0	0/0	1/1	0/0	0/0	0/0	0/0	0/0	0/1	0/0	0/0	1/1	1/1	1/1	0/0	1/1	1/1	0/0	1/1	0/0
0/0	0/0	0/0	0/0	0/0	0/0	0/0	0/0	0/0	0/0	0/0	0/0	0/0	0/0	0/0	0/0	0/0	0/0	0/0	0/0	0/0	0/0	0/0	0/0	0/0	0/0	0/0
0/0	0/0	0/0	0/0	0/0	0/0	0/0	0/0	0/0	0/0	0/0	0/0	0/0	0/0	0/0	0/0	0/0	0/0	0/0	0/0	0/0	0/0	0/0	0/0	0/0	0/0	0/0
0/0	0/0	0/0	0/0	0/0	0/0	0/0	0/0	0/0	0/0	0/0	0/0	0/0	0/0	0/0	0/0	0/0	0/0	0/0	0/0	0/0	0/0	0/0	0/0	0/0	0/0	0/0

0/0	0/0	0/0	0/0	0/0	0/0	0/0	0/0	0/0	0/0	0/0	0/0	0/0	0/0	0/0	0/0	0/0	0/0	0/0	0/0	0/0	0/0	0/0	0/0	0/0	0/0	0/0
0/0	0/0	0/0	0/0	0/0	0/0	0/0	0/0	0/0	0/0	0/0	0/0	0/0	0/0	0/0	0/0	0/0	0/0	0/0	0/0	0/0	0/0	0/0	0/0	0/0	0/0	0/0
0/0	1/1	1/1	0/0	0/0	1/1	0/0	0/0	0/0	1/1	0/0	0/0	2/2	0/0	1/1	1/1	0/0	0/0	1/1	1/1	1/1	0/0	1/1	1/1	1/1	1/1	2/2
0/0	0/0	0/0	0/0	0/0	0/0	0/0	0/0	0/0	0/0	0/0	0/0	0/0	0/0	0/0	0/0	0/0	0/0	0/0	0/0	0/0	0/0	0/0	0/0	0/0	0/0	0/0
0/0	1/1	1/1	0/0	0/0	1/1	0/0	0/0	0/0	1/1	0/0	0/0	2/2	0/0	0/0	1/1	0/0	0/0	1/1	1/1	1/1	0/0	1/1	1/1	0/0	1/1	2/2
0/0	0/0	0/0	0/0	0/0	0/0	0/0	0/0	0/0	0/0	0/0	0/0	0/0	0/0	0/0	0/0	0/0	0/0	0/0	0/0	0/0	0/0	0/0	0/0	0/0	0/0	0/0
0/0	0/0	0/0	0/0	0/0	0/0	0/0	0/0	0/0	0/0	0/0	0/0	0/0	0/0	1/1	0/0	0/0	0/0	0/0	0/0	0/0	0/0	0/0	0/0	1/1	0/0	0/0
0/0	1/1	1/1	0/0	0/0	1/1	0/0	0/0	0/0	1/1	0/0	0/0	1/1	0/0	1/1	1/1	0/0	0/0	1/1	1/1	1/1	0/0	1/1	1/1	1/1	1/1	1/1
0/0	1/1	1/1	0/0	0/0	1/1	0/0	0/0	0/0	1/1	0/0	0/0	1/1	0/0	1/1	1/1	0/0	0/0	1/1	1/1	1/1	0/0	1/1	1/1	1/1	1/1	1/1
0/0	0/0	0/0	0/0	0/0	0/0	0/0	0/0	0/0	0/0	0/0	0/0	0/0	0/0	0/0	0/0	0/0	0/0	0/0	0/0	0/0	0/0	0/0	0/0	0/0	0/0	0/0
0/0	1/1	1/1	0/0	0/0	1/1	0/0	0/0	0/0	1/1	0/0	0/0	2/2	0/0	2/2	1/1	0/0	0/0	1/1	1/1	1/1	0/0	1/1	1/1	2/2	1/1	2/2
0/0	0/0	0/0	0/0	0/0	0/0	0/0	0/0	0/0	0/0	0/0	0/0	0/0	0/0	0/0	0/0	0/0	0/0	0/0	0/0	0/0	0/0	0/0	0/0	0/0	0/0	0/0
0/0	0/0	0/0	0/0	0/0	0/0	0/0	0/0	0/0	0/0	0/0	0/0	0/0	0/0	0/0	0/0	0/0	0/0	0/0	0/0	0/0	0/0	0/0	0/0	0/0	0/0	0/0
0/0	0/0	0/0	0/0	0/0	0/0	0/0	0/0	0/0	0/0	0/0	0/0	0/0	0/0	0/0	0/0	0/0	0/0	0/0	0/0	0/0	0/0	0/0	0/0	0/0	0/0	0/0
0/0	0/0	0/0	0/0	0/0	0/0	0/0	0/0	0/0	0/0	0/0	0/0	0/0	0/0	1/1	0/0	0/0	0/0	0/0	0/0	0/0	0/0	0/0	0/0	1/1	0/0	0/0
0/0	0/0	0/0	0/0	0/0	0/0	0/0	0/0	0/0	0/0	0/0	0/0	0/0	0/0	0/0	0/0	0/0	0/0	0/0	0/0	0/0	0/0	0/0	0/0	0/0	0/0	0/0
0/0	0/0	0/0	0/0	0/0	0/0	0/0	0/0	0/0	0/0	0/0	0/0	0/0	0/0	1/1	0/0	0/0	0/0	0/0	0/0	0/0	0/0	0/0	0/0	1/1	0/0	0/0
0/0	1/1	1/1	0/0	0/0	1/1	0/0	0/0	0/0	1/1	0/0	0/0	1/1	0/0	1/1	1/1	0/0	0/0	1/1	1/1	1/1	0/0	1/1	1/1	1/1	1/1	1/1

1438	1442	1445	1453	19	24	433	454	455	456	472	488	501	507	516	518	536	543	557	562	565	591	594	607	615	620	625
0/0	0/0	0/0	0/0	0/0	0/0	0/0	0/0	./.	0/0	0/0	0/0	0/0	0/0	0/0	0/0	0/0	0/0	0/0	0/0	0/0	0/0	1/1	0/0	0/0	0/0	0/0
0/0	1/1	0/0	0/0	1/1	0/0	0/0	0/1	./.	0/0	0/0	0/0	0/0	0/0	0/0	0/0	0/0	0/0	1/1	0/0	0/0	0/0	0/0	0/0	0/0	0/0	0/0
0/0	0/0	0/0	0/0	0/0	0/0	0/0	0/0	./.	0/0	0/0	0/0	0/0	0/0	0/0	0/0	0/0	0/0	0/0	0/0	0/0	0/0	0/0	0/0	0/0	0/0	0/0
0/0	0/0	0/0	0/0	0/0	0/0	0/0	0/0	./.	0/0	1/1	0/0	0/0	0/0	0/0	0/0	0/0	0/0	0/0	0/0	0/0	0/0	0/0	0/0	0/0	0/0	0/0
0/0	0/0	0/0	0/0	0/0	0/0	1/1	0/1	./.	1/1	1/1	1/1	1/1	0/0	1/1	1/1	1/1	0/0	0/0	1/1	1/1	1/1	0/0	1/1	1/1	1/1	1/1
0/0	0/0	0/0	0/0	0/0	0/0	1/1	0/1	./.	1/1	1/1	1/1	1/1	0/0	1/1	1/1	1/1	0/0	0/0	1/1	1/1	1/1	0/0	1/1	1/1	1/1	1/1
0/0	0/0	0/0	0/0	0/0	0/0	0/0	0/0	./.	0/0	0/0	0/0	0/0	0/0	0/0	0/0	0/0	0/0	0/0	0/0	0/0	0/0	0/0	0/0	0/0	0/0	0/0
0/0	1/1	0/0	0/0	1/1	1/1	0/0	0/1	./.	0/0	0/0	0/0	0/0	1/1	0/0	0/0	0/0	1/1	1/1	0/0	0/0	0/0	1/1	0/0	0/0	0/0	0/0
0/0	0/0	0/0	0/0	0/0	0/0	0/0	0/0	./.	0/0	0/0	0/0	0/0	0/0	0/0	0/0	0/0	0/0	0/0	0/0	0/0	0/0	0/0	0/0	0/0	0/0	0/0
0/0	0/0	0/0	0/0	0/0	0/0	0/0	0/0	./.	0/0	0/0	0/0	0/0	0/0	0/0	0/0	0/0	0/0	0/0	0/0	0/0	0/0	0/0	0/0	0/0	0/0	0/0
0/0	0/0	0/0	0/0	0/1	0/0	0/0	0/0	./.	0/0	0/0	0/0	0/0	0/0	0/0	0/0	0/0	0/0	0/0	0/0	0/0	0/0	0/0	0/0	0/0	0/0	0/0
0/0	0/0	0/0	0/0	0/0	0/0	0/0	0/0	./.	0/0	0/0	0/0	0/0	0/0	0/0	0/0	0/0	0/0	0/0	0/0	0/0	0/0	0/0	0/0	0/0	0/0	0/0
0/0	0/0	0/0	0/0	0/0	0/0	1/1	0/0	./.	1/1	1/1	1/1	1/1	0/0	1/1	1/1	1/1	0/0	0/0	1/1	1/1	1/1	0/0	1/1	1/1	1/1	1/1
0/0	0/0	0/0	0/0	0/0	0/0	0/0	0/0	./.	0/0	0/0	0/0	0/0	0/0	0/0	0/0	0/0	0/1	0/0	0/0	0/0	0/0	./.	./.	0/0	0/0	0/0
0/0	1/1	0/0	0/0	1/1	1/1	./.	1/1	./.	./.	./.	./.	./.	1/1	./.	0/0	0/0	0/1	1/1	0/0	0/0	./.	1/1	./.	0/0	./.	0/0
0/0	0/0	0/0	0/0	0/0	0/0	./.	0/0	./.	./.	./.	./.	./.	0/0	./.	0/0	0/0	0/0	0/0	0/0	0/0	./.	0/0	./.	0/0	./.	0/0

0/0	1/1	2/2	2/3	3/3	1/1	2/2	3/3	./.	./.	2/3	1/2	1/1	1/1	1/2	1/2	2/3	1/5	./.	0/1	0/0	0/0	3/3	3/6	2/2	6/6	2/2
2/2	0/0	0/0	0/0	0/0	0/0	0/0	0/0	./.	0/0	0/0	0/0	0/0	0/0	0/0	0/0	0/0	0/0	0/0	0/0	./.	0/0	0/0	0/0	0/0	0/0	0/0
0/0	0/0	0/0	0/0	0/0	0/0	0/0	0/0	./.	./.	0/0	0/0	0/0	0/0	0/0	0/0	0/0	0/0	0/0	0/0	./.	0/0	0/0	0/0	0/0	0/0	0/0
0/0	0/0	0/0	0/0	0/0	0/0	1/1	0/0	./.	1/1	0/0	1/1	1/1	0/0	1/1	1/1	1/1	0/0	0/0	1/1	1/1	1/1	0/0	1/1	1/1	1/1	1/1
0/0	1/1	0/0	0/0	1/1	1/1	0/0	0/1	./.	0/0	0/0	0/0	0/0	1/1	0/0	0/0	0/0	1/1	1/1	0/0	0/0	0/0	1/1	0/0	0/0	0/0	0/0
0/0	0/0	0/0	0/0	0/0	0/0	0/0	0/0	./.	0/0	1/1	0/0	0/0	0/0	0/0	0/0	0/0	0/0	0/0	0/0	0/0	0/0	0/0	0/0	0/0	0/0	0/0
0/0	1/1	0/0	0/0	1/1	1/1	0/0	0/1	./.	0/0	0/0	0/0	0/0	1/1	0/0	0/0	0/0	1/1	1/1	0/0	0/0	0/0	1/1	0/0	0/0	0/0	0/0
0/0	0/0	0/0	0/0	0/0	0/0	1/1	0/1	./.	1/1	0/0	1/1	1/1	0/0	1/1	1/1	1/1	0/0	0/0	1/1	1/1	1/1	0/0	1/1	1/1	1/1	1/1
0/0	0/0	1/1	1/1	0/0	0/0	0/0	0/0	./.	0/0	0/0	0/0	0/0	0/0	0/0	0/0	0/0	0/0	0/0	0/0	0/0	0/0	0/0	0/0	0/0	0/0	0/0
0/0	0/0	2/2	2/2	0/0	1/1	1/1	0/1	./.	1/1	1/1	1/1	1/1	1/1	1/1	1/1	1/1	1/1	0/0	1/1	1/1	1/1	1/1	1/1	1/1	1/1	1/1
0/0	0/0	./.	./.	0/0	0/0	0/0	0/0	./.	0/0	0/0	0/0	0/0	0/0	0/0	0/0	0/0	0/0	0/0	0/0	0/0	0/0	0/0	0/0	0/0	0/0	0/0
0/0	0/0	0/0	0/0	0/0	0/0	1/1	0/1	./.	1/1	0/0	1/1	1/1	0/0	1/1	1/1	1/1	0/0	0/0	1/1	1/1	1/1	0/0	1/1	1/1	1/1	1/1
0/0	1/1	0/0	0/0	1/1	1/1	1/1	1/1	./.	1/1	1/1	1/1	1/1	1/1	1/1	1/1	1/1	1/1	1/1	1/1	1/1	1/1	1/1	1/1	1/1	1/1	1/1
0/0	0/0	0/0	0/0	0/0	0/0	0/0	0/0	./.	0/0	1/1	0/0	0/0	0/0	0/0	0/0	0/0	0/0	0/0	0/0	0/0	0/0	0/0	0/0	0/0	0/0	0/0
0/0	0/0	0/0	0/0	0/0	0/0	0/0	0/0	./.	0/0	0/0	0/0	0/0	0/0	0/0	0/0	0/0	0/0	0/0	0/0	0/0	0/0	0/0	0/0	0/0	0/0	0/0
0/0	1/1	0/0	0/0	1/1	1/1	1/1	1/1	./.	1/1	1/1	1/1	1/1	1/1	1/1	1/1	1/1	1/1	1/1	1/1	1/1	1/1	1/1	1/1	1/1	1/1	1/1
0/0	0/0	0/0	0/0	0/0	0/0	1/1	0/0	./.	1/1	0/0	1/1	1/1	0/0	1/1	1/1	1/1	0/0	0/0	1/1	1/1	1/1	0/0	1/1	1/1	1/1	1/1
0/0	0/0	0/0	0/0	0/0	0/0	0/0	0/0	./.	0/0	1/1	0/0	0/0	0/0	0/0	0/0	0/0	0/0	0/0	0/0	0/0	0/0	0/0	0/0	0/0	0/0	0/0
0/0	1/1	0/0	0/0	1/1	1/1	0/0	1/1	./.	0/0	0/0	0/0	0/0	1/1	0/0	0/0	0/0	1/1	1/1	0/0	0/0	0/0	1/1	0/0	0/0	0/0	0/0
0/0	1/1	0/0	0/0	1/1	0/0	0/0	1/1	./.	0/0	0/0	0/0	0/0	0/0	0/0	0/0	0/0	0/0	1/1	0/0	0/0	0/0	0/0	0/0	0/0	0/0	0/0
0/0	0/0	0/0	0/0	0/0	0/0	0/0	0/0	./.	0/0	1/1	0/0	0/0	0/0	0/0	0/0	0/0	0/0	0/0	0/0	0/0	0/0	0/0	0/0	0/0	0/0	0/0
0/0	1/1	0/0	0/0	1/1	1/1	0/0	1/1	./.	0/0	0/0	0/0	0/0	1/1	0/0	0/0	0/0	1/1	1/1	0/0	0/0	0/0	1/1	0/0	0/0	0/0	0/0
0/0	0/0	0/0	0/0	0/0	0/0	0/0	0/0	./.	0/0	1/1	0/0	0/0	0/0	0/0	0/0	0/0	0/0	0/0	0/0	0/0	0/0	0/0	0/0	0/0	0/0	0/0
0/0	1/1	0/0	0/0	1/1	1/1	1/1	1/1	./.	1/1	1/1	1/1	1/1	1/1	1/1	1/1	1/1	1/1	1/1	1/1	1/1	1/1	1/1	1/1	1/1	1/1	1/1
0/0	0/0	0/0	0/0	0/0	0/0	1/1	0/1	./.	1/1	0/0	1/1	1/1	0/0	1/1	1/1	1/1	0/0	0/0	1/1	1/1	1/1	0/0	1/1	1/1	1/1	1/1
0/0	1/1	0/0	0/0	1/1	0/0	0/0	1/1	./.	0/0	2/2	0/0	0/0	0/0	0/0	0/0	0/0	0/0	1/1	0/0	0/0	0/0	0/0	0/0	0/0	0/0	0/0

0/0	1/1	1/1	1/1	1/1	1/1	1/1	1/1	./.	1/1	1/1	1/1	1/1	1/1	1/1	1/1	1/1	1/1	1/1	1/1	1/1	1/1	1/1	1/1	1/1	1/1	1/1
0/0	0/0	0/0	0/0	0/0	0/0	1/1	0/0	./.	1/1	1/1	1/1	1/1	0/0	1/1	1/1	1/1	0/0	0/0	1/1	1/1	1/1	0/0	1/1	1/1	1/1	1/1
0/0	0/0	0/0	0/0	0/0	0/0	1/1	0/0	./.	1/1	0/0	1/1	1/1	0/0	1/1	1/1	1/1	0/0	0/0	1/1	1/1	1/1	0/0	1/1	1/1	1/1	1/1
0/0	0/0	0/0	0/0	0/0	0/0	0/0	0/0	./.	1/1	0/0	0/0	0/0	0/0	0/0	0/0	0/0	0/0	0/0	0/0	0/0	0/0	0/0	0/0	0/0	0/0	0/0
0/0	1/1	0/0	0/0	1/1	1/1	1/1	1/1	./.	1/1	1/1	1/1	1/1	1/1	1/1	1/1	1/1	1/1	1/1	1/1	1/1	1/1	1/1	1/1	1/1	1/1	1/1
0/0	1/1	0/0	0/0	1/1	1/1	1/1	1/1	./.	1/1	1/1	1/1	1/1	1/1	1/1	1/1	1/1	1/1	1/1	1/1	1/1	1/1	1/1	1/1	1/1	1/1	1/1
0/0	1/1	0/0	0/0	1/1	1/1	1/1	1/1	./.	1/1	1/1	1/1	1/1	1/1	1/1	1/1	1/1	1/1	1/1	1/1	1/1	1/1	1/1	1/1	1/1	1/1	1/1
0/0	0/0	0/0	0/0	0/0	0/0	0/0	0/0	./.	0/0	1/1	0/0	0/0	0/0	0/0	0/0	0/0	0/0	0/0	0/0	0/0	0/0	0/0	./.	0/0	0/0	0/0
0/0	0/0	0/0	0/0	0/0	0/0	1/1	0/1	./.	1/1	0/0	1/1	1/1	0/0	1/1	1/1	1/1	0/0	0/0	1/1	1/1	1/1	0/0	1/1	1/1	1/1	1/1
1/1	0/0	0/0	0/0	0/0	0/0	0/0	0/0	./.	0/0	0/0	0/0	0/0	0/0	0/0	0/0	0/0	0/0	0/0	0/0	0/0	0/0	0/0	0/0	0/0	0/0	0/0
0/0	1/1	0/0	0/0	1/1	1/1	0/0	0/1	./.	0/0	0/0	0/0	0/0	1/1	0/0	0/0	0/0	1/1	1/1	0/0	0/0	0/0	1/1	0/0	0/0	0/0	0/0
0/0	0/0	0/0	0/0	0/0	0/0	0/0	0/0	./.	0/0	0/0	0/0	0/0	0/0	0/0	0/0	0/0	0/0	0/0	0/0	0/0	0/0	0/0	0/0	0/0	0/0	0/0
0/0	0/0	0/0	0/0	0/0	0/0	0/0	0/0	./.	0/0	0/0	0/0	0/0	0/0	0/0	0/0	0/0	0/0	0/0	0/0	0/0	0/0	0/0	0/0	0/0	0/0	0/0
0/0	0/0	0/0	0/0	0/0	1/1	0/0	0/0	./.	0/0	0/0	0/0	0/0	1/1	0/0	0/0	0/0	1/1	0/0	0/0	0/0	0/0	1/1	0/0	0/0	0/0	0/0
0/0	1/1	0/0	0/0	1/1	1/1	1/1	1/1	./.	1/1	1/1	1/1	1/1	1/1	1/1	1/1	1/1	1/1	1/1	1/1	1/1	1/1	1/1	1/1	1/1	1/1	1/1
0/0	0/0	0/0	0/0	0/0	0/0	0/0	0/0	./.	0/0	1/1	0/0	0/0	0/0	0/0	0/0	0/0	0/0	0/0	0/0	0/0	0/0	0/0	0/0	0/0	0/0	0/0
0/0	0/0	0/0	0/0	0/0	0/0	0/0	0/0	./.	0/0	0/0	0/0	0/0	0/0	0/0	0/0	0/0	0/0	0/0	0/0	0/0	0/0	0/0	0/0	0/0	0/0	0/0
0/0	0/0	0/0	0/0	0/0	0/0	0/0	0/0	./.	0/0	0/0	0/0	0/0	0/0	0/0	0/0	0/0	0/0	0/0	0/0	0/0	0/0	0/0	0/0	0/0	0/0	0/0
0/0	1/1	0/0	0/0	1/1	1/1	0/0	0/1	./.	0/0	0/0	0/0	0/0	1/1	0/0	0/0	0/0	1/1	1/1	0/0	0/0	0/0	1/1	0/0	0/0	0/0	0/0
0/0	0/0	0/0	0/0	0/0	0/0	0/0	0/0	./.	0/0	1/1	0/0	0/0	0/0	0/0	0/0	0/0	0/0	0/0	0/0	0/0	0/0	0/0	0/0	0/0	0/0	0/0
0/0	1/1	0/0	0/0	1/1	1/1	1/1	1/1	./.	1/1	1/1	1/1	1/1	1/1	1/1	1/1	1/1	1/1	1/1	1/1	1/1	1/1	1/1	1/1	1/1	1/1	1/1
0/0	1/1	0/0	0/0	1/1	1/1	0/0	0/1	./.	0/0	0/0	0/0	0/0	1/1	0/0	0/0	0/0	1/1	1/1	0/0	0/0	0/0	1/1	0/0	0/0	0/0	0/0
0/0	1/1	0/0	0/0	1/1	1/1	0/0	0/1	./.	0/0	0/0	0/0	0/0	1/1	0/0	0/0	0/0	1/1	1/1	0/0	0/0	0/0	1/1	0/0	0/0	0/0	0/0
0/0	0/0	0/0	0/0	0/0	0/0	1/1	0/1	./.	1/1	1/1	1/1	1/1	0/0	1/1	1/1	1/1	0/0	0/0	1/1	1/1	1/1	0/0	1/1	1/1	1/1	1/1
0/0	1/1	0/0	0/0	1/1	1/1	1/1	1/1	./.	1/1	1/1	1/1	1/1	1/1	1/1	1/1	1/1	1/1	1/1	1/1	1/1	1/1	1/1	1/1	1/1	1/1	1/1
0/0	0/0	0/0	0/0	0/0	0/0	1/1	0/0	./.	1/1	1/1	1/1	1/1	0/0	1/1	1/1	1/1	0/0	0/0	1/1	1/1	1/1	0/0	1/1	1/1	1/1	1/1
0/0	0/0	0/0	0/0	0/0	0/0	0/0	0/0	./.	0/0	0/0	0/0	0/0	0/0	0/0	0/0	0/0	0/0	0/0	0/0	0/0	0/0	0/0	0/0	0/0	0/0	0/0
0/0	0/0	0/0	0/0	0/0	0/0	0/0	0/0	./.	0/0	1/1	0/0	0/0	0/0	0/0	0/0	0/0	0/0	0/0	0/0	0/0	0/0	0/0	0/0	0/0	0/0	0/0
0/0	1/1	1/1	1/1	1/1	1/1	1/1	1/1	./.	1/1	1/1	1/1	1/1	1/1	1/1	1/1	1/1	1/1	1/1	1/1	1/1	1/1	1/1	1/1	1/1	1/1	1/1
0/0	0/0	0/0	0/0	0/0	0/0	0/0	0/0	./.	0/0	0/0	0/0	0/0	0/0	0/0	0/0	0/0	0/0	0/0	0/0	0/0	0/0	0/0	0/0	0/0	0/0	0/0
0/0	1/1	0/0	0/0	1/1	1/1	1/1	1/1	./.	1/1	1/1	1/1	1/1	1/1	1/1	1/1	1/1	1/1	1/1	1/1	1/1	1/1	1/1	1/1	1/1	1/1	1/1
0/0	0/0	0/0	0/0	0/0	0/0	1/1	0/1	./.	1/1	0/0	1/1	1/1	0/0	1/1	1/1	1/1	0/0	0/0	1/1	1/1	1/1	0/0	1/1	1/1	1/1	1/1
0/0	1/1	0/0	0/0	1/1	1/1	1/1	1/1	./.	1/1	1/1	1/1	1/1	1/1	1/1	1/1	1/1	1/1	1/1	1/1	1/1	1/1	1/1	1/1	1/1	1/1	1/1
0/0	0/0	0/0	0/0	0/0	0/0	0/0	0/0	./.	0/0	0/0	0/0	0/0	0/0	0/0	0/0	0/0	0/0	0/0	0/0	0/0	0/0	0/0	0/0	0/0	0/0	0/0
0/0	0/0	0/0	0/0	0/0	0/0	1/1	0/1	./.	1/1	1/1	1/1	1/1	0/0	1/1	1/1	1/1	0/0	0/0	1/1	1/1	1/1	0/0	1/1	1/1	1/1	1/1

0/0	1/1	0/0	0/0	1/1	1/1	0/0	1/1	./.	0/0	0/0	0/0	0/0	1/1	0/0	0/0	0/0	1/1	1/1	0/0	0/0	0/0	1/1	0/0	0/0	0/0	0/0
0/0	0/0	0/0	0/0	0/0	0/0	0/0	0/0	./.	0/0	0/0	0/0	0/0	0/0	0/0	0/0	0/0	0/0	0/0	0/0	0/0	0/0	0/0	0/0	0/0	0/0	0/0
0/0	1/1	0/0	0/0	1/1	0/0	0/0	1/1	./.	0/0	0/0	0/0	0/0	0/0	0/0	0/0	0/0	0/0	1/1	0/0	0/0	0/0	0/0	0/0	0/0	0/0	0/0
0/0	1/1	0/0	0/0	1/1	1/1	1/1	1/1	./.	1/1	1/1	1/1	1/1	1/1	1/1	1/1	1/1	1/1	1/1	1/1	1/1	1/1	1/1	1/1	1/1	1/1	1/1
0/0	0/0	0/0	0/0	0/0	0/0	0/0	0/0	./.	0/0	0/0	0/0	0/0	0/0	0/0	0/0	0/0	0/0	0/0	0/0	0/0	0/0	0/0	0/0	0/0	0/0	0/0
0/0	1/1	0/0	0/0	1/1	1/1	0/0	0/1	./.	0/0	0/0	0/0	0/0	1/1	0/0	0/0	0/0	1/1	1/1	0/0	0/0	0/0	1/1	0/0	0/0	0/0	0/0
0/0	1/1	0/0	0/0	1/1	1/1	1/1	1/1	./.	1/1	1/1	1/1	1/1	1/1	1/1	1/1	1/1	1/1	1/1	1/1	1/1	1/1	1/1	1/1	1/1	1/1	1/1
0/0	0/0	0/0	0/0	0/0	0/0	1/1	0/1	./.	1/1	0/0	1/1	1/1	0/0	1/1	1/1	1/1	0/0	0/0	1/1	1/1	1/1	0/0	1/1	1/1	1/1	1/1
0/0	1/1	0/0	0/0	1/1	1/1	0/0	0/1	./.	0/0	0/0	0/0	2/2	1/1	0/0	0/0	0/0	1/1	1/1	0/0	0/0	0/0	1/1	0/0	0/0	0/0	0/0
0/0	0/0	0/0	0/0	0/0	0/0	0/0	0/0	./.	0/0	0/0	0/0	0/0	0/0	0/0	0/0	0/0	0/0	0/0	0/0	0/0	0/0	0/0	0/0	0/0	0/0	0/0
0/0	1/1	0/0	0/0	1/1	1/1	0/0	0/1	./.	0/0	0/0	0/0	0/0	1/1	0/0	0/0	0/0	1/1	1/1	0/0	0/0	0/0	1/1	0/0	0/0	0/0	0/0
0/0	0/0	0/0	0/0	0/0	0/0	0/0	0/0	./.	0/0	1/1	0/0	0/0	0/0	0/0	0/0	0/0	0/0	0/0	0/0	0/0	0/0	0/0	0/0	0/0	0/0	0/0
0/0	0/0	0/0	0/0	0/0	0/0	0/0	0/0	./.	0/0	1/1	0/0	0/0	0/0	0/0	0/0	0/0	0/0	0/0	0/0	0/0	0/0	0/0	0/0	0/0	0/0	0/0
0/0	0/0	0/0	0/0	0/0	0/0	0/0	0/0	./.	0/0	0/0	0/0	0/0	0/0	0/0	0/0	0/0	0/0	0/0	0/0	0/0	0/0	0/0	0/0	0/0	0/0	0/0
0/0	1/1	1/1	1/1	1/1	1/1	1/1	1/1	./.	1/1	1/1	1/1	1/1	1/1	1/1	1/1	1/1	1/1	1/1	1/1	1/1	1/1	1/1	1/1	1/1	1/1	1/1
0/0	1/1	1/1	1/1	1/1	1/1	1/1	1/1	./.	1/1	1/1	1/1	1/1	1/1	1/1	1/1	1/1	1/1	1/1	1/1	1/1	1/1	1/1	1/1	1/1	1/1	1/1
0/0	1/1	0/0	0/0	1/1	1/1	1/1	1/1	./.	1/1	1/1	1/1	1/1	1/1	1/1	1/1	1/1	1/1	1/1	1/1	1/1	1/1	1/1	1/1	1/1	1/1	1/1
0/0	1/1	./.	./.	./.	./.	./.	./.	./.	./.	./.	./.	./.	./.	0/0	./.	./.	./.	./.	./.	./.	./.	./.	./.	./.	./.	./.
2/2	4/4	1/1	1/1	1/1	1/1	3/3	1/1	./.	3/3	3/3	3/3	3/3	1/1	3/3	3/3	3/3	1/1	1/1	3/3	3/3	3/3	1/1	3/3	3/3	3/3	3/3
0/0	0/0	0/0	0/0	0/0	0/0	0/0	0/0	./.	0/0	0/0	1/1	0/0	0/0	1/1	1/1	0/0	0/0	0/0	1/1	1/1	0/0	0/0	0/0	0/0	0/0	1/1
0/0	0/0	0/0	0/0	0/0	0/0	0/0	0/0	./.	0/0	0/0	0/0	0/0	0/0	0/0	0/0	0/0	0/0	0/0	0/0	0/0	0/0	0/0	0/0	0/0	0/0	0/0
0/0	0/0	0/0	0/0	0/0	0/0	0/0	0/0	./.	0/0	0/0	0/0	0/0	0/0	0/0	0/0	0/0	0/0	0/0	0/0	0/0	0/0	0/0	0/0	0/0	0/0	0/0
0/0	0/0	0/0	0/0	0/0	0/0	0/0	0/0	./.	0/0	0/0	0/0	0/0	0/0	0/0	0/0	0/0	0/0	0/0	0/0	0/0	0/0	0/0	0/0	0/0	0/0	0/0
0/0	0/0	0/0	0/0	0/0	0/0	0/0	0/0	./.	./.	1/1	0/0	0/0	0/0	0/0	0/0	0/0	0/0	0/0	0/0	0/0	0/0	0/0	0/0	0/0	0/0	./.
0/0	0/0	0/0	0/0	0/0	0/0	1/1	0/0	./.	1/1	0/0	1/1	1/1	0/0	1/1	1/1	1/1	0/0	0/0	1/1	1/1	1/1	0/0	1/1	1/1	1/1	1/1

0/0	0/0	0/0	0/0	0/0	0/0	0/0	0/0	./.	0/0	0/0	0/0	0/0	0/0	0/0	0/0	0/0	0/0	0/0	0/0	0/0	0/0	0/0	0/0	0/0	0/0	0/0
0/0	0/0	0/0	0/0	0/0	0/0	1/1	0/0	./.	1/1	0/0	1/1	1/1	0/0	1/1	1/1	1/1	0/0	0/0	1/1	1/1	1/1	0/0	1/1	1/1	1/1	1/1
0/0	0/0	0/0	0/0	0/0	0/0	1/1	0/0	./.	1/1	0/0	1/1	1/1	0/0	1/1	1/1	1/1	0/0	0/0	1/1	1/1	1/1	0/0	1/1	1/1	1/1	1/1
0/0	1/1	1/1	1/1	1/1	1/1	1/1	1/1	./.	1/1	1/1	1/1	1/1	1/1	1/1	1/1	1/1	1/1	1/1	1/1	1/1	1/1	1/1	1/1	1/1	1/1	1/1
0/0	1/1	0/0	0/0	1/1	1/1	1/1	1/1	./.	1/1	1/1	1/1	1/1	1/1	1/1	1/1	1/1	1/1	1/1	1/1	1/1	1/1	1/1	1/1	1/1	1/1	1/1
0/0	0/0	0/0	0/0	0/0	0/0	0/0	0/0	./.	0/0	0/0	0/0	0/0	0/0	0/0	0/0	0/0	0/0	0/0	0/0	0/0	0/0	0/0	0/0	0/0	0/0	0/0
0/0	1/1	0/0	0/0	1/1	1/1	1/1	1/1	./.	1/1	1/1	1/1	1/1	1/1	1/1	1/1	1/1	1/1	1/1	1/1	1/1	1/1	1/1	1/1	1/1	1/1	1/1
0/0	0/0	0/0	0/0	0/0	0/0	1/1	0/1	./.	1/1	0/0	1/1	1/1	0/0	1/1	1/1	1/1	0/0	0/0	1/1	1/1	1/1	0/0	1/1	1/1	1/1	1/1
0/0	0/0	0/0	0/0	0/0	0/0	0/0	0/0	./.	0/0	1/1	0/0	0/0	0/0	0/0	0/0	0/0	0/0	0/0	0/0	0/0	0/0	0/0	0/0	0/0	0/0	0/0
0/0	0/0	0/0	0/0	0/0	0/0	0/0	0/0	./.	0/0	0/0	0/0	0/0	0/0	0/0	0/0	0/0	0/0	0/0	0/0	0/0	0/0	0/0	0/0	0/0	0/0	0/0
0/0	0/0	0/0	0/0	0/0	0/0	1/1	0/0	./.	1/1	0/0	1/1	1/1	0/0	1/1	1/1	1/1	0/0	0/0	1/1	1/1	1/1	0/0	1/1	1/1	1/1	1/1
0/0	0/0	0/0	0/0	0/0	0/0	0/0	0/0	./.	0/0	0/0	0/0	0/0	0/0	0/0	0/0	0/0	0/0	0/0	0/0	0/0	0/0	0/0	0/0	0/0	0/0	0/0
0/0	1/1	0/0	0/0	1/1	1/1	0/0	0/1	./.	0/0	0/0	0/0	0/0	1/1	0/0	0/0	0/0	1/1	1/1	0/0	0/0	0/0	1/1	0/0	0/0	0/0	0/0
0/0	0/0	0/0	0/0	0/0	0/0	1/1	0/0	./.	1/1	0/0	1/1	1/1	0/0	1/1	1/1	1/1	0/0	0/0	1/1	1/1	1/1	0/0	1/1	1/1	1/1	1/1
0/0	0/0	0/0	0/0	0/0	0/0	0/0	0/0	./.	0/0	0/0	0/0	0/0	0/0	0/0	0/0	0/0	0/0	0/0	0/0	0/0	0/0	0/0	0/0	0/0	0/0	0/0
0/0	1/1	0/0	0/0	1/1	1/1	1/1	1/1	./.	1/1	1/1	1/1	1/1	1/1	1/1	1/1	1/1	1/1	1/1	1/1	1/1	1/1	1/1	1/1	1/1	1/1	1/1
0/0	1/1	0/0	0/0	1/1	1/1	0/0	1/1	./.	0/0	0/0	0/0	0/0	1/1	0/0	0/0	0/0	1/1	1/1	0/0	0/0	0/0	1/1	0/0	0/0	0/0	0/0
0/0	0/0	0/0	0/0	0/0	1/1	0/0	0/0	./.	0/0	0/0	0/0	0/0	1/1	0/0	0/0	0/0	1/1	0/0	0/0	0/0	0/0	1/1	0/0	0/0	0/0	0/0
0/0	0/0	0/0	0/0	0/0	0/0	0/0	0/0	./.	0/0	0/0	./.	0/0	0/0	0/0	0/0	0/0	0/0	0/0	0/0	0/0	0/0	0/0	0/0	0/0	0/0	0/0
0/0	0/0	0/0	0/0	0/0	0/0	1/1	0/0	./.	1/1	0/0	1/1	1/1	0/0	1/1	1/1	1/1	0/0	0/0	1/1	1/1	1/1	0/0	1/1	1/1	1/1	1/1
0/0	0/0	0/0	0/0	0/0	0/0	1/1	0/0	./.	1/1	0/0	1/1	1/1	0/0	1/1	1/1	1/1	0/0	0/0	1/1	1/1	1/1	0/0	1/1	1/1	1/1	1/1
0/0	0/0	0/0	0/0	0/0	0/0	0/0	0/0	./.	0/0	0/0	0/0	0/0	0/0	0/0	0/0	0/0	0/0	0/0	0/0	0/0	0/0	0/0	0/0	0/0	0/0	0/0
0/0	1/1	0/0	0/0	1/1	1/1	1/1	1/1	./.	1/1	1/1	1/1	1/1	1/1	1/1	1/1	1/1	1/1	1/1	1/1	1/1	1/1	1/1	1/1	1/1	1/1	1/1
0/0	0/0	0/0	0/0	0/0	0/0	0/0	0/0	./.	0/0	0/0	0/0	0/0	0/0	0/0	0/0	0/0	0/0	0/0	0/0	0/0	0/0	0/0	0/0	0/0	0/0	0/0
0/0	0/0	0/0	0/0	0/0	0/0	0/0	0/0	./.	0/0	1/1	0/0	0/0	0/0	0/0	0/0	0/0	0/0	0/0	0/0	0/0	0/0	0/0	0/0	0/0	0/0	0/0
0/0	0/0	0/0	0/0	0/0	0/0	1/1	0/0	./.	1/1	0/0	1/1	1/1	0/0	1/1	1/1	1/1	0/0	0/0	1/1	1/1	1/1	0/0	1/1	1/1	1/1	1/1
0/0	0/0	0/0	0/0	0/0	0/0	0/0	0/0	./.	0/0	0/0	0/0	0/0	0/0	0/0	0/0	0/0	0/0	0/0	0/0	0/0	0/0	0/0	0/0	0/0	0/0	0/0
0/0	1/1	0/0	0/0	1/1	1/1	0/0	0/1	./.	0/0	0/0	0/0	0/0	1/1	0/0	0/0	0/0	1/1	1/1	0/0	0/0	0/0	1/1	0/0	0/0	0/0	0/0
0/0	0/0	0/0	0/0	0/0	0/0	1/1	0/1	./.	1/1	0/0	1/1	1/1	0/0	1/1	1/1	1/1	0/0	0/0	1/1	1/1	1/1	0/0	1/1	1/1	1/1	1/1
0/0	0/0	0/0	0/0	0/0	0/0	0/0	0/0	./.	0/0	0/0	0/0	0/0	0/0	0/0	0/0	0/0	0/0	0/0	0/0	0/0	0/0	0/0	0/0	0/0	0/0	0/0
0/0	1/1	0/0	0/0	1/1	1/1	1/1	1/1	./.	1/1	1/1	1/1	1/1	1/1	1/1	1/1	1/1	1/1	1/1	1/1	1/1	1/1	1/1	1/1	1/1	1/1	1/1
0/0	0/0	0/0	0/0	0/0	0/0	0/0	0/0	./.	0/0	0/0	0/0	0/0	0/0	0/0	0/0	0/0	0/0	0/0	0/0	0/0	0/0	0/0	0/0	0/0	0/0	0/0
0/0	0/0	1/1	1/1	0/0	0/0	0/0	0/0	./.	0/0	0/0	0/0	0/0	0/0	0/0	0/0	0/0	0/0	0/0	0/0	0/0	0/0	0/0	0/0	0/0	0/0	0/0

0/0	1/1	0/0	0/0	1/1	1/1	1/1	1/1	./.	1/1	1/1	1/1	1/1	1/1	1/1	1/1	1/1	1/1	1/1	1/1	1/1	1/1	1/1	1/1	1/1	1/1	1/1
0/0	0/0	0/0	0/0	0/0	0/0	1/1	0/0	./.	0/0	0/0	0/0	0/0	0/0	0/0	0/0	0/0	0/0	0/0	0/0	0/0	0/0	0/0	0/0	0/0	0/0	0/0
0/0	1/1	0/0	0/0	1/1	1/1	1/1	1/1	./.	1/1	1/1	1/1	1/1	1/1	1/1	1/1	1/1	1/1	1/1	1/1	1/1	1/1	1/1	1/1	1/1	1/1	1/1
0/0	0/0	0/0	0/0	0/0	0/0	0/0	0/0	./.	0/0	0/0	0/0	0/0	0/0	0/0	0/0	0/0	0/0	0/0	0/0	0/0	0/0	0/0	0/0	0/0	0/0	0/0
0/0	1/1	0/0	0/0	1/1	0/0	0/0	1/1	./.	0/0	0/0	0/0	0/0	0/0	0/0	0/0	0/0	0/0	1/1	0/0	0/0	0/0	0/0	0/0	0/0	0/0	0/0
0/0	0/0	0/0	0/0	0/0	0/0	0/0	0/0	./.	0/0	0/0	0/0	0/0	0/0	0/0	0/0	0/0	0/0	0/0	0/0	0/0	0/0	0/0	0/0	0/0	0/0	0/0
0/0	0/0	0/0	0/0	0/0	0/0	0/0	0/0	./.	0/0	1/1	0/0	0/0	0/0	0/0	0/0	0/0	0/0	0/0	0/0	0/0	0/0	0/0	0/0	0/0	0/0	0/0
0/0	0/0	0/0	0/0	0/0	1/1	0/0	0/0	./.	0/0	0/0	0/0	0/0	1/1	0/0	0/0	0/0	1/1	0/0	0/0	0/0	0/0	1/1	0/0	0/0	0/0	0/0
0/0	0/0	0/0	0/0	0/0	0/0	0/0	0/0	./.	0/0	0/0	0/0	0/0	0/0	0/0	0/0	0/0	0/0	0/0	0/0	0/0	0/0	0/0	0/0	0/0	0/0	0/0
0/0	0/0	0/0	0/0	0/0	0/0	0/0	0/0	./.	0/0	1/1	0/0	0/0	0/0	0/0	0/0	0/0	0/0	0/0	0/0	0/0	0/0	0/0	0/0	0/0	0/0	0/0
0/0	1/1	0/0	0/0	1/1	1/1	1/1	1/1	./.	1/1	1/1	1/1	1/1	1/1	1/1	1/1	1/1	1/1	1/1	1/1	1/1	1/1	1/1	1/1	1/1	1/1	1/1
0/0	1/1	0/0	0/0	1/1	1/1	1/1	1/1	./.	1/1	1/1	1/1	1/1	1/1	1/1	1/1	1/1	1/1	1/1	1/1	1/1	1/1	1/1	1/1	1/1	1/1	1/1
0/0	0/0	0/0	0/0	0/0	0/0	0/0	0/0	./.	0/0	1/1	0/0	0/0	0/0	0/0	0/0	0/0	0/0	0/0	0/0	0/0	0/0	0/0	0/0	0/0	0/0	0/0
0/0	1/1	0/0	0/0	1/1	0/0	0/0	1/1	./.	0/0	0/0	0/0	0/0	0/0	0/0	0/0	0/0	0/0	1/1	0/0	0/0	0/0	0/0	0/0	0/0	0/0	0/0
0/0	0/0	0/0	0/0	0/0	0/0	1/1	0/0	./.	1/1	0/0	1/1	1/1	0/0	1/1	1/1	1/1	0/0	0/0	1/1	1/1	1/1	0/0	1/1	1/1	1/1	1/1
0/0	1/1	2/2	2/2	1/1	1/1	1/1	1/1	./.	1/1	0/0	1/1	1/1	1/1	1/1	1/1	1/1	1/1	1/1	1/1	1/1	1/1	1/1	1/1	1/1	1/1	1/1
0/0	1/1	0/0	0/0	1/1	1/1	1/1	1/1	./.	1/1	1/1	1/1	1/1	1/1	1/1	1/1	1/1	1/1	1/1	1/1	1/1	1/1	1/1	1/1	1/1	1/1	1/1
0/0	0/0	0/0	0/0	0/0	1/1	1/1	0/0	0/0	1/1	0/0	1/1	1/1	0/0	1/1	1/1	1/1	1/1	0/0	1/1	1/1	1/1	0/0	1/1	1/1	1/1	1/1
0/0	0/0	0/0	0/0	0/0	1/1	1/1	0/1	0/0	1/1	0/0	1/1	1/1	0/0	1/1	1/1	1/1	1/1	0/0	1/1	1/1	1/1	0/0	1/1	1/1	1/1	1/1
0/0	1/1	0/0	0/0	1/1	0/0	0/0	1/1	0/0	0/0	0/0	0/0	0/0	0/0	0/0	0/0	0/0	0/0	1/1	0/0	0/0	0/0	0/0	0/0	0/0	0/0	0/0
0/0	0/0	0/0	0/0	0/0	0/0	0/0	0/0	0/0	0/0	0/0	0/0	0/0	1/1	0/0	0/0	0/0	0/0	0/0	0/0	0/0	0/0	0/0	0/0	0/0	0/0	0/0
0/0	0/0	0/0	0/0	0/0	0/0	0/0	0/0	0/0	0/0	0/0	0/0	0/0	0/0	0/0	0/0	0/0	0/0	0/0	0/0	0/0	0/0	0/0	0/0	0/0	0/0	0/0
0/0	0/0	0/0	0/0	0/0	0/0	0/0	0/0	0/0	0/0	0/0	0/0	0/0	0/0	0/0	0/0	0/0	0/0	0/0	0/0	0/0	0/0	0/0	0/0	0/0	0/0	0/0
0/0	0/0	0/0	0/0	0/0	0/0	0/0	0/0	0/0	0/0	0/0	0/0	0/0	0/0	0/0	0/0	0/0	0/0	0/0	0/0	0/0	0/0	0/0	0/0	0/0	0/0	0/0
0/0	0/0	0/0	0/0	0/0	0/0	0/0	0/1	0/0	0/0	0/0	0/0	0/0	0/0	0/0	0/0	0/0	0/0	0/0	0/0	0/0	0/0	0/0	0/0	0/0	0/0	0/0
0/0	0/1	0/0	0/0	0/0	0/0	0/1	0/1	0/0	0/0	0/0	0/0	0/0	0/0	0/0	0/0	0/0	0/0	0/0	0/0	0/0	0/0	0/0	0/0	0/0	0/0	./.

0/0	0/1	0/0	0/0	0/0	0/0	0/1	0/1	0/0	0/0	0/0	0/0	0/0	0/0	0/0	0/0	0/0	0/0	0/0	0/0	0/0	0/0	0/0	0/0	0/0	0/0	./.
0/0	0/0	0/0	0/0	0/0	0/0	0/1	0/1	0/0	0/0	0/0	0/0	0/0	0/0	0/0	0/0	0/0	0/0	0/0	0/0	0/0	0/0	0/0	0/0	0/0	0/0	./.
0/0	0/0	0/0	0/0	0/0	0/0	0/1	0/1	0/0	0/0	0/0	0/0	0/0	0/0	0/0	0/0	0/0	0/0	0/0	0/0	0/0	0/0	0/0	0/0	0/0	0/0	./.
0/0	0/0	0/0	0/0	0/0	0/0	0/0	0/1	0/0	0/0	0/0	0/0	0/0	0/0	0/0	0/0	0/0	0/0	0/0	0/0	0/0	0/0	0/0	0/0	0/0	0/0	./.
0/0	0/0	0/0	0/0	0/0	0/0	0/0	0/1	0/0	0/0	0/0	0/0	0/0	0/0	0/0	0/0	0/0	0/0	0/0	0/0	0/0	0/0	0/0	0/0	0/0	0/0	0/0
0/0	0/0	0/0	0/0	0/0	0/0	0/0	0/1	0/0	0/0	0/0	0/0	0/0	0/0	0/0	0/0	0/0	0/0	0/0	0/0	0/0	0/0	0/0	0/0	0/0	0/0	0/0
0/0	0/0	0/0	0/0	0/0	0/0	0/0	0/0	0/0	0/0	0/0	0/0	0/0	0/0	0/0	0/0	0/0	0/0	0/0	0/0	0/0	0/0	0/0	0/0	0/0	0/0	0/0
0/0	0/0	0/0	0/0	0/0	0/0	0/0	0/0	0/0	0/0	0/0	0/0	0/0	0/0	0/0	0/0	0/0	0/0	0/0	0/0	0/0	0/0	0/0	0/0	0/0	0/0	0/0
0/0	0/0	0/0	0/0	0/0	0/0	0/0	0/0	0/0	0/0	0/0	0/0	0/0	0/0	0/0	0/0	0/0	0/0	0/0	0/0	0/0	0/0	0/0	0/0	0/0	0/0	0/0
0/0	0/0	0/0	0/0	0/0	0/0	0/0	0/0	0/0	0/0	0/0	0/0	0/0	0/0	0/0	0/0	0/0	0/0	0/0	0/0	0/0	0/0	0/0	0/0	0/0	0/0	0/0
1/1	1/1	1/1	1/1	1/1	1/1	1/1	1/1	1/1	1/1	1/1	1/1	1/1	1/1	1/1	1/1	1/1	1/1	1/1	1/1	1/1	1/1	1/1	1/1	1/1	1/1	1/1
0/0	0/0	0/0	0/0	0/0	0/0	0/0	0/0	0/0	0/0	0/0	0/0	0/0	0/0	0/0	0/0	0/0	0/0	0/0	0/0	0/0	0/0	0/0	0/0	0/0	0/0	0/0
0/0	0/0	0/0	0/0	0/0	0/0	0/0	0/0	0/0	0/0	0/0	0/0	0/0	0/0	0/0	0/0	0/0	0/0	0/0	0/0	0/0	0/0	0/0	0/0	0/0	0/0	0/0
0/0	0/0	0/0	0/0	0/0	0/0	0/0	0/0	1/1	0/0	1/1	0/0	0/0	1/1	0/0	0/0	0/0	0/1	0/0	0/0	0/0	0/0	0/0	0/0	0/0	0/0	0/0
0/0	0/0	0/0	0/0	0/0	0/0	0/0	0/0	0/0	0/0	0/0	0/0	0/0	0/0	0/0	0/0	0/0	0/0	0/0	0/0	0/0	0/0	0/0	0/0	0/0	0/0	0/0
0/0	0/0	0/0	0/0	0/0	0/0	0/0	0/0	0/0	0/0	0/0	0/0	0/0	0/0	0/0	0/0	0/0	0/0	0/0	0/0	0/0	0/0	0/0	0/0	0/0	0/0	0/0
0/0	1/1	0/0	0/0	1/1	1/1	1/1	1/1	1/1	1/1	1/1	1/1	1/1	1/1	1/1	1/1	1/1	1/1	1/1	1/1	1/1	1/1	1/1	1/1	1/1	1/1	1/1
0/0	0/0	0/0	0/0	0/0	0/0	./.	0/0	./.	0/0	0/0	./.	0/0	0/0	./.	./.	./.	0/0	0/0	./.	./.	0/0	./.	0/0	./.	./.	./.
0/0	1/1	0/0	0/0	1/1	1/1	./.	0/1	1/1	./.	1/1	./.	0/0	1/1	./.	./.	./.	1/1	1/1	0/0	./.	./.	./.	0/0	./.	./.	./.
0/0	0/0	0/0	0/0	0/0	0/0	1/1	0/1	0/0	1/1	0/0	1/1	1/1	0/0	1/1	1/1	1/1	0/0	0/0	1/1	1/1	1/1	1/1	1/1	1/1	1/1	1/1
0/0	0/0	0/0	0/0	0/0	0/0	1/1	0/1	0/0	1/1	0/0	1/1	1/1	0/0	1/1	1/1	1/1	0/0	0/0	1/1	1/1	1/1	1/1	1/1	1/1	1/1	1/1
0/0	0/0	0/0	0/0	0/0	./.	0/0	0/0	./.	0/0	0/0	0/0	0/0	0/0	0/0	0/0	0/0	./.	./.	0/0	0/0	0/0	0/0	0/0	0/0	0/0	0/0
./.	1/1	0/0	./.	0/0	./.	0/0	0/0	./.	0/0	./.	0/0	0/0	./.	0/0	0/0	0/0	./.	0/0	0/0	0/0	0/0	0/0	0/0	0/0	0/0	0/0
./.	2/2	0/0	0/0	1/1	0/1	0/0	0/1	1/1	0/0	1/1	0/0	0/0	1/1	0/0	0/0	0/0	1/1	1/1	0/0	0/0	0/0	0/0	0/0	0/0	0/0	0/0

0/0	2/2	0/0	0/0	2/2	0/0	0/0	0/2	2/2	0/0	0/0	0/0	0/0	0/0	0/0	0/0	0/0	0/0	0/0	0/0	0/0	0/0	0/0	0/0	0/0	0/0	0/0
3/3	4/4	3/3	3/3	4/4	0/4	0/0	0/4	4/4	0/0	4/4	0/0	0/0	4/4	0/0	0/0	0/0	4/4	4/4	0/0	0/0	0/0	0/0	0/0	0/0	0/0	0/0
0/0	0/0	0/0	0/0	0/0	0/0	0/0	0/0	0/0	0/0	0/0	0/0	0/0	0/0	0/0	0/0	0/0	0/0	0/0	0/0	0/0	0/0	0/0	0/0	0/0	0/0	0/0
0/0	1/1	0/0	0/0	1/1	1/1	1/1	1/1	1/1	1/1	1/1	1/1	1/1	1/1	1/1	1/1	1/1	1/1	1/1	1/1	1/1	1/1	1/1	1/1	1/1	1/1	1/1
0/0	1/1	0/0	0/0	1/1	1/1	0/0	0/1	1/1	0/0	1/1	0/0	0/0	1/1	0/0	0/0	0/0	1/1	1/1	0/0	0/0	0/0	0/0	0/0	0/0	0/0	0/0
0/0	0/0	0/0	0/0	0/0	0/0	0/0	0/0	0/0	0/0	0/0	0/0	0/0	0/0	0/0	0/0	0/0	0/0	0/0	0/0	0/0	0/0	0/0	0/0	0/0	0/0	0/0
0/0	0/0	0/0	0/0	0/0	0/0	0/0	0/0	0/0	0/0	0/0	0/0	0/0	0/0	0/0	0/0	0/0	0/0	0/0	0/0	0/0	0/0	0/0	0/0	0/0	0/0	0/0
0/0	1/1	0/0	0/0	1/1	1/1	1/1	1/1	1/1	1/1	1/1	1/1	1/1	1/1	1/1	1/1	1/1	1/1	1/1	1/1	1/1	1/1	1/1	1/1	1/1	1/1	1/1
0/0	0/0	0/0	0/0	0/0	./.	0/0	0/0	0/0	0/0	0/0	0/0	0/0	0/0	0/0	0/0	0/0	0/0	./.	0/0	0/0	0/0	0/0	0/0	0/0	0/0	0/0
0/0	1/1	0/0	0/0	1/1	1/1	0/0	0/1	1/1	0/0	1/1	0/0	0/0	1/1	0/0	0/0	0/0	1/1	1/1	0/0	0/0	0/0	0/0	0/0	0/0	0/0	0/0
0/0	1/1	0/0	0/0	1/1	1/1	0/0	0/1	1/1	0/0	1/1	0/0	0/0	1/1	0/0	0/0	0/0	1/1	1/1	0/0	0/0	0/0	0/0	0/0	0/0	0/0	0/0
0/0	1/1	0/0	0/0	1/1	1/1	0/0	0/1	1/1	0/0	1/1	0/0	0/0	1/1	0/0	0/0	0/0	1/1	1/1	0/0	0/0	0/0	0/0	0/0	0/0	0/0	0/0
0/0	0/0	0/0	0/0	0/0	0/0	1/1	0/1	0/0	1/1	0/0	1/1	1/1	0/0	1/1	1/1	1/1	0/0	0/0	1/1	1/1	1/1	1/1	1/1	1/1	1/1	1/1
0/0	1/1	0/0	0/0	1/1	1/1	0/0	0/1	1/1	0/0	1/1	0/0	0/0	1/1	0/0	0/0	0/0	1/1	1/1	0/0	0/0	0/0	0/0	0/0	0/0	0/0	0/0
0/0	1/1	0/0	0/0	1/1	1/1	1/1	1/1	1/1	1/1	1/1	1/1	1/1	1/1	1/1	1/1	1/1	1/1	1/1	1/1	1/1	1/1	1/1	1/1	1/1	1/1	1/1
0/0	0/0	0/0	0/0	0/0	0/0	0/0	0/0	0/0	0/0	0/0	0/0	0/0	0/0	0/0	0/0	0/0	0/0	0/0	0/0	0/0	0/0	0/0	0/0	0/0	0/0	0/0
0/0	0/0	0/0	0/0	0/0	0/0	0/0	0/0	0/0	0/0	0/0	0/0	0/0	0/0	0/0	0/0	0/0	0/0	0/0	0/0	0/0	0/0	0/0	0/0	0/0	0/0	0/0
0/0	0/0	0/0	0/0	0/0	0/0	1/1	0/1	0/0	1/1	0/0	1/1	1/1	0/0	1/1	1/1	1/1	0/0	0/0	1/1	1/1	1/1	1/1	1/1	1/1	1/1	1/1
0/0	0/0	0/0	0/0	0/0	0/0	0/0	0/0	0/0	0/0	0/0	0/0	0/0	0/0	0/0	0/0	0/0	0/0	0/0	0/0	0/0	0/0	0/0	0/0	0/0	0/0	0/0
0/0	0/0	0/0	0/0	0/0	0/0	1/1	0/1	0/0	1/1	0/0	1/1	1/1	0/0	1/1	1/1	1/1	0/0	0/0	1/1	1/1	1/1	1/1	1/1	1/1	1/1	1/1
0/0	0/0	0/0	0/0	0/0	1/1	0/0	0/0	1/1	0/0	1/1	0/0	0/0	1/1	0/0	0/0	0/0	1/1	0/0	0/0	0/0	0/0	0/0	0/0	0/0	0/0	0/0
0/0	0/0	0/0	0/0	0/0	0/0	1/1	0/0	0/0	1/1	0/0	1/1	1/1	0/0	1/1	1/1	1/1	0/0	0/0	1/1	1/1	1/1	1/1	1/1	1/1	1/1	1/1
0/0	0/0	0/0	0/0	0/0	0/0	1/1	0/0	0/0	1/1	0/0	1/1	1/1	0/0	1/1	1/1	1/1	0/0	0/0	1/1	1/1	1/1	1/1	1/1	1/1	1/1	1/1
0/0	0/0	0/0	0/0	0/0	0/0	0/0	0/0	0/0	0/0	0/0	0/0	0/0	0/0	0/0	0/0	0/0	0/0	0/0	0/0	0/0	0/0	0/0	0/0	0/0	0/0	0/0
0/0	0/0	0/0	0/0	0/0	0/0	0/0	0/0	0/0	0/0	0/0	0/0	0/0	0/0	0/0	0/0	0/0	0/0	0/0	0/0	0/0	0/0	0/0	0/0	0/0	0/0	0/0
0/0	1/1	0/0	0/0	1/1	1/1	0/0	0/1	1/1	0/0	1/1	0/0	0/0	1/1	0/0	0/0	0/0	1/1	1/1	0/0	0/0	0/0	0/0	0/0	0/0	0/0	0/0
0/0	0/0	0/0	0/0	0/0	0/0	0/0	0/0	0/0	0/0	0/0	0/0	0/0	0/0	0/0	0/0	0/0	0/0	0/0	0/0	0/0	0/0	0/0	0/0	0/0	0/0	0/0
0/0	1/1	0/0	0/0	1/1	1/1	1/1	1/1	1/1	1/1	1/1	1/1	1/1	1/1	1/1	1/1	1/1	1/1	1/1	1/1	1/1	1/1	1/1	1/1	1/1	1/1	1/1
0/0	0/0	0/0	0/0	0/0	0/0	0/0	0/0	0/0	0/0	0/0	0/0	0/0	0/0	0/0	0/0	0/0	0/0	0/0	0/0	0/0	0/0	0/0	0/0	0/0	0/0	0/0
0/0	1/1	0/0	0/0	1/1	1/1	1/1	1/1	1/1	1/1	1/1	1/1	1/1	1/1	1/1	1/1	1/1	1/1	1/1	1/1	1/1	1/1	1/1	1/1	1/1	1/1	1/1

0/0	0/0	0/0	0/0	0/0	0/0	0/0	0/0	0/0	0/0	0/0	0/0	0/0	0/0	0/0	0/0	0/0	0/0	0/0	0/0	0/0	0/0	0/0	0/0	0/0	0/0	0/0
0/0	0/0	0/0	0/0	0/0	0/0	0/0	0/0	0/0	0/0	0/0	0/0	0/0	0/0	0/0	0/0	0/0	0/0	0/0	0/0	0/0	0/0	0/0	0/0	0/0	0/0	0/0
0/0	0/0	0/0	0/0	0/0	0/0	0/0	0/0	0/0	0/0	0/0	0/0	0/0	0/0	0/0	0/0	0/0	0/0	0/0	0/0	0/0	0/0	0/0	0/0	0/0	0/0	0/0
0/0	1/1	0/0	0/0	1/1	0/0	0/0	0/1	0/0	0/0	0/0	0/0	0/0	0/0	0/0	0/0	0/0	0/0	1/1	0/0	0/0	0/0	0/0	0/0	0/0	0/0	0/0
0/0	1/1	0/0	0/0	1/1	1/1	0/0	0/1	1/1	0/0	1/1	0/0	0/0	1/1	0/0	0/0	0/0	1/1	1/1	0/0	0/0	0/0	0/0	0/0	0/0	0/0	0/0
0/0	1/1	0/0	0/0	1/1	1/1	1/1	1/1	1/1	1/1	1/1	1/1	1/1	1/1	1/1	1/1	1/1	1/1	1/1	1/1	1/1	1/1	1/1	1/1	1/1	1/1	1/1
0/0	1/1	0/0	0/0	1/1	1/1	0/0	0/1	1/1	0/0	1/1	0/0	0/0	1/1	0/0	0/0	0/0	1/1	1/1	0/0	0/0	0/0	0/0	0/0	0/0	0/0	0/0
0/0	0/0	0/0	0/0	0/0	1/1	0/0	0/0	1/1	0/0	1/1	0/0	0/0	1/1	0/0	0/0	0/0	1/1	0/0	0/0	0/0	0/0	0/0	0/0	0/0	0/0	0/0
0/0	0/0	0/0	0/0	0/0	0/0	0/0	0/0	0/0	0/0	0/0	0/0	0/0	0/0	0/0	0/0	0/0	0/0	0/0	0/0	0/0	0/0	0/0	0/0	0/0	0/0	0/0
0/0	0/0	0/0	0/0	0/0	0/0	0/0	0/0	0/0	0/0	0/0	0/0	0/0	0/0	0/0	0/0	0/0	0/0	0/0	0/0	1/1	0/0	0/0	0/0	0/0	0/0	0/0
0/0	0/0	0/0	0/0	0/0	0/0	0/0	0/0	0/0	0/0	0/0	0/0	0/0	0/0	0/0	0/0	0/0	0/0	0/0	0/0	0/0	0/0	0/0	0/0	0/0	0/0	0/0
0/0	0/0	0/0	0/0	0/0	0/0	1/1	0/0	0/0	1/1	0/0	1/1	1/1	0/0	1/1	1/1	1/1	0/0	0/0	1/1	1/1	1/1	1/1	1/1	1/1	1/1	1/1
0/0	1/1	0/0	0/0	1/1	0/0	1/1	1/1	0/0	1/1	0/0	1/1	1/1	0/0	1/1	1/1	1/1	0/0	1/1	1/1	1/1	1/1	1/1	1/1	1/1	0/0	1/1
0/0	1/1	0/0	0/0	1/1	1/1	1/1	1/1	1/1	1/1	1/1	1/1	1/1	1/1	1/1	1/1	1/1	1/1	1/1	1/1	1/1	1/1	1/1	1/1	1/1	1/1	1/1
1/1	1/1	1/1	1/1	1/1	1/1	1/1	1/1	1/1	1/1	1/1	1/1	1/1	1/1	1/1	1/1	1/1	1/1	1/1	1/1	1/1	1/1	1/1	1/1	1/1	1/1	1/1
0/0	0/0	0/0	0/0	0/0	0/0	0/0	0/0	0/0	0/0	0/0	0/0	0/0	0/0	0/0	0/0	0/0	0/0	0/0	0/0	0/0	0/0	0/0	0/0	0/0	0/0	0/0
0/0	1/1	0/0	0/0	1/1	0/0	0/0	0/1	0/0	0/0	0/0	0/0	0/0	0/0	0/0	0/0	0/0	0/0	1/1	0/0	0/0	0/0	0/0	0/0	0/0	0/0	0/0
0/0	1/1	0/0	0/0	1/1	1/1	0/0	0/1	1/1	0/0	1/1	0/0	0/0	1/1	0/0	0/0	0/0	1/1	1/1	0/0	0/0	0/0	0/0	0/0	0/0	0/0	0/0
0/0	1/1	0/0	0/0	1/1	1/1	1/1	1/1	1/1	1/1	1/1	1/1	1/1	1/1	1/1	1/1	1/1	1/1	1/1	1/1	1/1	1/1	1/1	1/1	1/1	1/1	1/1
0/0	0/0	0/0	0/0	0/0	0/0	0/0	0/0	0/0	0/0	0/0	0/0	0/0	0/0	0/0	0/0	0/0	0/0	0/0	0/0	0/0	0/0	0/0	0/0	0/0	0/0	0/0
0/0	0/0	0/0	0/0	0/0	0/0	0/0	0/0	0/0	0/0	0/0	0/0	0/0	0/0	0/0	0/0	0/0	0/0	0/0	0/0	0/0	0/0	0/0	0/0	0/0	0/0	0/0
0/0	0/0	0/0	0/0	0/0	0/0	0/0	0/0	0/0	0/0	0/0	0/0	0/0	0/0	0/0	0/0	0/0	0/0	0/0	0/0	0/0	0/0	0/0	0/0	0/0	0/0	0/0
0/0	1/1	0/0	0/0	1/1	1/1	1/1	1/1	1/1	1/1	1/1	1/1	1/1	1/1	1/1	1/1	1/1	1/1	1/1	1/1	1/1	1/1	1/1	1/1	1/1	1/1	1/1
0/0	0/0	0/0	0/0	0/0	0/0	0/0	0/0	0/0	0/0	0/0	0/0	0/0	0/0	0/0	0/0	0/0	0/0	0/0	0/0	0/0	0/0	0/0	0/0	0/0	0/0	0/0
0/0	0/0	0/0	0/0	0/0	0/0	0/0	0/0	0/0	0/0	0/0	0/0	0/0	0/0	0/0	0/0	0/0	0/0	0/0	0/0	0/0	0/0	0/0	0/0	0/0	0/0	0/0
0/0	0/0	0/0	0/0	0/0	0/0	0/0	0/0	0/0	0/0	0/0	0/0	0/0	0/0	0/0	0/0	0/0	0/0	0/0	0/0	0/0	0/0	0/0	0/0	0/0	0/0	0/0
0/0	0/0	0/0	0/0	0/0	0/0	0/0	0/0	0/0	0/0	0/0	0/0	0/0	0/0	0/0	0/0	0/0	0/0	0/0	0/0	0/0	0/0	0/0	0/0	0/0	0/0	0/0
0/0	0/0	0/0	0/0	0/0	0/0	0/0	0/0	0/0	0/0	0/0	0/0	0/0	0/0	0/0	0/0	0/0	0/0	0/0	0/0	0/0	0/0	0/0	0/0	0/0	0/0	0/0
0/0	0/0	0/0	0/0	0/0	0/0	0/0	0/0	0/0	0/0	0/0	0/0	0/0	0/0	0/0	0/0	0/0	0/0	0/0	0/0	0/0	0/0	0/0	0/0	0/0	0/0	0/0
0/0	0/0	0/0	0/0	0/0	0/0	0/0	0/0	0/0	0/0	0/0	0/0	0/0	0/0	0/0	0/0	0/0	0/1	0/0	0/0	0/0	0/0	0/0	0/0	0/0	0/0	0/0
0/0	0/0	0/0	0/0	0/0	0/0	0/0	0/0	0/0	0/0	0/0	0/0	0/0	0/0	0/0	0/0	0/0	0/0	0/0	0/0	0/0	0/0	0/0	0/0	0/0	0/0	0/0
0/0	0/0	0/0	0/0	0/0	0/0	1/1	0/1	0/0	1/1	0/0	1/1	1/1	0/0	1/1	1/1	1/1	0/0	0/0	1/1	1/1	1/1	1/1	1/1	1/1	1/1	1/1
0/0	0/0	0/0	0/0	0/0	0/0	0/0	0/0	0/0	0/0	0/0	0/0	0/0	0/0	0/0	0/0	0/0	0/0	0/0	0/0	0/0	0/0	0/0	0/0	0/0	0/0	0/0
0/0	0/0	0/0	0/0	0/0	0/0	0/0	0/0	0/0	0/0	0/0	0/0	0/0	0/0	0/0	0/0	0/0	0/0	0/0	0/0	0/0	0/0	0/0	0/0	0/0	0/0	0/0
0/0	0/0	0/0	0/0	1/1	0/0	0/0	0/1	0/0	0/0	0/0	0/0	0/0	0/0	0/0	0/0	0/0	0/0	1/1	0/0	0/0	0/0	0/0	0/0	0/0	0/0	0/0

0/0	0/0	0/0	0/0	0/0	1/1	0/0	0/0	1/1	0/0	1/1	0/0	0/0	1/1	0/0	0/0	0/0	1/1	0/0	0/0	0/0	0/0	0/0	0/0	0/0	0/0	0/0
2/3	2/2	2/2	2/2	2/2	4/4	1/1	2/2	1/4	1/2	4/4	1/1	1/2	4/4	2/2	2/2	1/1	4/4	2/2	1/1	2/2	1/1	1/1	1/1	1/1	1/2	2/2
./.	./.	./.	./.	./.	./.	./.	./.	./.	./.	./.	./.	./.	./.	./.	./.	./.	./.	./.	./.	./.	./.	./.	./.	./.	./.	./.
0/0	0/0	0/0	0/0	0/0	0/0	0/0	0/0	0/0	0/0	0/0	0/0	0/0	0/0	0/0	0/0	0/0	0/0	0/0	0/0	0/0	0/0	0/0	0/0	0/0	0/0	0/0
0/0	0/0	0/0	0/0	0/0	0/0	0/0	0/0	0/0	0/0	0/0	0/0	0/0	0/0	0/0	0/0	0/0	0/0	0/0	0/0	0/0	0/0	0/0	0/0	0/0	0/0	0/0
0/0	0/0	0/0	0/0	0/0	0/0	0/0	0/0	0/0	0/0	0/0	0/0	0/0	0/0	0/0	0/0	0/0	0/0	0/0	0/0	0/0	0/0	0/0	0/0	0/0	0/0	0/0
0/0	0/0	0/0	0/0	0/0	1/1	0/0	0/0	1/1	0/0	1/1	0/0	0/0	1/1	0/0	0/0	0/0	1/1	0/0	0/0	0/0	0/0	0/0	0/0	0/0	0/0	0/0
0/0	./.	0/0	./.	0/0	1/1	0/0	0/0	1/1	./.	1/1	0/0	0/0	1/1	0/0	0/0	0/0	1/1	0/0	0/0	0/0	0/0	0/0	0/0	0/0	0/0	0/0
0/0	./.	0/0	./.	0/0	0/0	./.	0/0	0/0	./.	0/0	./.	0/0	0/0	0/0	0/0	0/0	0/0	0/0	0/0	0/0	0/0	0/0	0/0	0/0	0/0	0/0
./.	1/1	./.	./.	1/1	1/1	./.	1/1	1/1	./.	1/1	./.	./.	1/1	./.	./.	./.	1/1	1/1	0/0	./.	0/0	0/0	./.	./.	./.	./.
1/1	./.	1/1	1/1	./.	./.	2/2	./.	./.	2/2	./.	2/2	2/2	./.	2/2	3/3	2/2	./.	./.	2/2	2/2	2/2	2/2	2/2	2/2	2/2	2/2
2/2	0/0	2/2	2/2	0/0	./.	./.	./.	./.	./.	./.	./.	./.	0/0	./.	./.	./.	./.	./.	./.	./.	./.	./.	./.	./.	./.	./.
0/0	0/0	0/0	0/0	0/0	0/0	0/0	0/0	0/0	0/0	0/0	0/0	0/0	0/0	0/0	0/0	0/0	0/0	0/0	0/0	0/0	0/0	0/0	0/0	0/0	0/0	0/0
0/0	0/0	0/0	0/0	0/0	0/0	0/0	0/0	0/0	0/0	0/0	0/0	0/0	0/0	0/0	0/0	0/0	0/0	0/0	0/0	0/0	0/0	0/0	0/0	0/0	0/0	0/0
0/0	0/0	0/0	0/0	0/0	0/0	1/1	0/0	0/0	0/0	0/0	0/0	0/0	0/0	0/0	0/0	0/0	0/0	0/0	0/0	0/0	0/0	0/0	0/0	0/0	0/0	0/0
0/0	1/1	0/0	0/0	1/1	1/1	1/1	1/1	1/1	1/1	1/1	1/1	1/1	1/1	1/1	1/1	1/1	1/1	1/1	1/1	1/1	1/1	1/1	1/1	1/1	1/1	1/1
0/0	0/0	0/0	0/0	0/0	0/0	0/0	0/0	0/0	0/0	0/0	0/0	0/0	0/0	0/0	0/0	0/0	0/0	0/0	0/0	0/0	0/0	0/0	0/0	0/0	0/0	0/0
0/0	1/1	0/0	0/0	1/1	0/0	0/0	1/1	0/0	0/0	0/0	0/0	0/0	0/0	0/0	0/0	0/0	0/0	1/1	0/0	0/0	0/0	0/0	0/0	0/0	0/0	0/0
0/0	0/0	0/0	0/0	0/0	0/0	0/0	0/0	0/0	0/0	0/0	0/0	0/0	0/0	0/0	0/0	0/0	0/0	0/0	0/0	0/0	0/0	0/0	0/0	0/0	0/0	0/0
0/0	0/0	0/0	0/0	0/0	0/0	0/0	0/0	0/0	0/0	0/0	0/0	0/0	0/0	0/0	0/0	0/0	0/0	0/0	0/0	0/0	0/0	0/0	0/0	0/0	0/0	0/0
0/0	0/0	0/0	0/0	0/0	0/0	0/0	0/0	0/0	0/0	0/0	0/0	0/0	0/0	0/0	0/0	0/0	0/0	0/0	0/0	0/0	0/0	0/0	0/0	0/0	0/0	0/0

0/0	0/0	0/0	0/0	0/0	0/0	0/0	0/0	0/0	0/0	0/0	0/0	0/0	0/0	0/0	0/0	0/0	0/0	0/0	0/0	0/0	0/0	0/0	0/0	0/0	0/0	0/0
0/0	0/0	0/0	0/0	0/0	0/0	0/0	0/0	0/0	0/0	0/0	0/0	0/0	0/0	0/0	0/0	0/0	0/0	0/0	0/0	0/0	0/0	0/0	0/0	0/0	0/0	0/0
0/0	1/1	0/0	0/0	1/1	1/1	2/2	1/1	1/1	2/2	1/1	2/2	2/2	1/1	2/2	2/2	2/2	1/1	1/1	2/2	2/2	2/2	2/2	2/2	2/2	2/2	2/2
0/0	0/0	0/0	0/0	0/0	0/0	0/0	0/0	0/0	0/0	0/0	0/0	0/0	0/0	0/0	0/0	0/0	0/0	0/0	0/0	0/0	0/0	0/0	0/0	0/0	0/0	0/0
0/0	1/1	0/0	0/0	1/1	0/0	2/2	1/2	0/0	2/2	0/0	2/2	2/2	0/0	2/2	2/2	2/2	0/0	1/1	2/2	2/2	2/2	2/2	2/2	2/2	2/2	2/2
0/0	0/0	0/0	0/0	0/0	0/0	0/0	0/0	0/0	0/0	0/0	0/0	0/0	0/0	0/0	0/0	0/0	0/0	0/0	0/0	0/0	0/0	0/0	0/0	0/0	0/0	0/0
0/0	0/0	0/0	0/0	0/0	1/1	0/0	0/0	1/1	0/0	0/0	0/0	0/0	1/1	0/0	0/0	0/0	1/1	0/0	0/0	0/0	0/0	0/0	0/0	0/0	0/0	0/0
0/0	1/1	0/0	0/0	1/1	1/1	1/1	1/1	1/1	1/1	1/1	1/1	1/1	1/1	1/1	1/1	1/1	1/1	1/1	1/1	1/1	1/1	1/1	1/1	1/1	1/1	1/1
0/0	1/1	0/0	0/0	1/1	1/1	1/1	1/1	1/1	1/1	1/1	1/1	1/1	1/1	1/1	1/1	1/1	1/1	1/1	1/1	1/1	1/1	1/1	1/1	1/1	1/1	1/1
0/0	0/0	0/0	0/0	0/0	0/0	0/0	0/0	0/0	0/0	0/0	0/0	0/0	0/0	0/0	0/0	0/0	0/0	0/0	0/0	0/0	0/0	0/0	0/0	0/0	0/0	0/0
0/0	1/1	0/0	0/0	1/1	2/2	2/2	1/2	2/2	2/2	2/2	2/2	2/2	2/2	2/2	2/2	2/2	2/2	1/1	2/2	2/2	2/2	2/2	2/2	2/2	2/2	2/2
0/0	0/0	0/0	0/0	0/0	0/0	0/0	0/0	0/0	0/0	0/0	0/0	0/0	0/0	0/0	0/0	0/0	0/0	0/0	0/0	0/0	0/0	0/0	0/0	0/0	0/0	0/0
0/0	0/0	0/0	0/0	0/0	0/0	0/0	0/0	0/0	0/0	0/0	0/0	0/0	0/0	0/0	0/0	0/0	0/0	0/0	0/0	0/0	0/0	0/0	0/0	0/0	0/0	0/0
0/0	0/0	0/0	0/0	0/0	0/0	0/0	0/0	0/0	0/0	0/0	0/0	0/0	0/0	0/0	0/0	0/0	0/0	0/0	0/0	0/0	0/0	0/0	0/0	0/0	0/0	0/0
0/0	0/0	0/0	0/0	0/0	1/1	0/0	0/0	1/1	0/0	1/1	0/0	0/0	1/1	0/0	0/0	0/0	1/1	0/0	0/0	0/0	0/0	0/0	0/0	0/0	0/0	0/0
0/0	0/0	0/0	0/0	0/0	0/0	0/0	0/0	0/0	0/0	0/0	0/0	0/0	0/0	0/0	0/0	0/0	0/0	0/0	0/0	0/0	0/0	0/0	0/0	0/0	0/0	0/0
0/0	0/0	0/0	0/0	0/0	1/1	0/0	0/0	1/1	0/0	1/1	0/0	0/0	1/1	0/0	0/0	0/0	1/1	0/0	0/0	0/0	0/0	0/0	0/0	0/0	0/0	0/0
0/0	1/1	0/0	0/0	1/1	1/1	1/1	1/1	1/1	1/1	1/1	1/1	1/1	1/1	1/1	1/1	1/1	1/1	1/1	1/1	1/1	1/1	1/1	1/1	1/1	1/1	1/1

630	640	643	650	653	655	667	66	671	683	685	686	688	694	704	711	716	721	726	730	733	740	757	762	787	801	806
0/0	0/0	0/0	0/0	0/0	0/0	0/0	0/0	0/0	0/0	0/0	0/0	0/0	0/0	0/0	0/0	0/0	0/0	0/0	0/0	0/0	0/0	0/0	0/0	0/0	0/0	0/0
0/0	0/0	0/0	0/0	1/1	0/0	1/1	0/0	0/0	0/0	0/0	0/0	0/0	0/0	0/0	0/1	0/0	1/1	0/0	0/0	0/0	0/0	0/0	0/0	0/0	0/0	0/0
0/0	0/0	0/0	0/0	0/0	0/0	0/0	0/0	0/0	0/0	0/0	0/0	0/0	0/0	0/0	0/0	0/0	0/0	0/0	0/0	0/0	0/0	0/0	0/0	0/0	0/0	0/0
0/0	0/0	0/0	0/0	0/0	0/0	0/0	0/0	0/0	1/1	0/0	0/0	0/0	0/0	0/0	0/0	0/0	0/0	0/0	0/0	0/0	0/0	0/0	0/0	0/0	0/0	0/0
1/1	0/0	1/1	1/1	0/0	1/1	0/0	1/1	1/1	0/0	0/0	1/1	1/1	1/1	1/1	0/0	0/0	0/0	1/1	0/0	0/0	0/0	1/1	0/0	0/0	0/0	0/0
1/1	0/0	1/1	1/1	0/0	1/1	0/0	1/1	1/1	1/1	0/0	1/1	1/1	1/1	1/1	0/0	0/0	0/0	1/1	0/0	0/0	0/0	1/1	0/0	0/0	0/0	0/0
0/0	0/0	0/0	0/0	0/0	0/0	0/0	0/0	0/0	0/0	0/0	0/0	0/0	0/0	0/0	0/0	0/0	0/0	0/0	0/0	0/0	0/0	0/0	0/0	0/0	0/0	0/0
0/0	1/1	0/0	0/0	1/1	0/0	1/1	0/0	0/0	0/0	1/1	0/0	0/0	0/0	0/0	1/1	0/0	1/1	0/0	0/0	0/0	1/1	0/0	0/0	0/0	0/0	0/0
0/0	0/0	0/0	0/0	0/0	0/0	0/0	0/0	0/0	0/0	0/0	0/0	0/0	0/0	0/0	0/0	0/0	0/0	0/0	0/0	0/0	0/0	0/0	0/0	0/0	0/0	0/0
0/0	0/0	0/0	0/0	0/0	0/0	0/0	0/0	0/0	0/0	0/0	0/0	0/0	0/0	0/0	0/0	0/0	0/0	0/0	0/0	0/0	0/0	0/0	0/0	0/0	0/0	0/0
0/0	0/0	0/0	0/0	0/0	0/0	0/0	0/0	0/0	0/0	0/0	0/0	0/0	0/0	0/0	0/0	0/0	0/0	0/0	0/0	0/0	0/0	0/0	0/0	0/0	0/0	0/0
0/0	0/0	0/0	0/0	0/0	0/0	0/0	0/0	0/0	0/0	0/0	0/0	0/0	0/0	0/0	0/0	0/0	0/0	0/0	0/0	0/0	0/0	0/0	0/0	0/0	0/0	0/0
1/1	0/0	1/1	1/1	0/0	1/1	0/0	1/1	1/1	0/0	0/0	1/1	1/1	1/1	1/1	0/0	0/0	0/0	1/1	0/0	0/0	0/0	1/1	0/0	0/0	0/0	0/0
0/0	./.	0/0	0/0	0/0	0/0	./.	0/0	0/0	0/0	0/0	0/0	0/0	0/0	0/0	0/0	0/0	1/1	0/0	0/0	0/0	0/0	./.	0/0	0/0	0/0	0/0
0/0	1/1	0/0	0/0	1/1	./.	1/1	0/0	0/0	0/0	1/1	0/0	0/0	0/0	0/0	1/1	./.	0/0	0/0	0/0	0/0	1/1	./.	0/0	0/0	0/0	0/0
0/0	0/0	0/0	0/0	0/0	./.	0/0	0/0	0/0	0/0	./.	0/0	0/0	0/0	0/0	0/0	./.	0/0	0/0	0/0	0/0	0/0	./.	0/0	0/0	0/0	0/0

3/3	6/6	6/6	2/3	1/1	2/3	3/3	0/0	1/2	2/3	6/6	2/3	2/2	2/2	3/3	3/6	4/4	5/5	2/3	1/1	0/0	1/1	./.	0/0	0/0	0/0	4/4
0/0	0/0	0/0	0/0	0/0	0/0	0/0	0/0	0/0	0/0	0/0	0/0	0/0	0/0	0/0	0/0	0/0	0/0	0/0	0/0	0/0	0/0	0/0	0/0	0/0	0/0	0/0
0/0	0/0	0/0	0/0	./.	0/0	0/0	0/0	0/0	0/0	0/0	0/0	0/0	0/0	0/0	0/0	0/0	0/0	0/0	0/0	0/0	0/0	./.	0/0	0/0	0/0	0/0
1/1	0/0	1/1	1/1	0/0	1/1	0/0	1/1	1/1	0/0	0/0	1/1	1/1	1/1	1/1	0/0	0/0	0/0	1/1	0/0	0/0	0/0	1/1	0/0	0/0	0/0	0/0
0/0	1/1	0/0	0/0	1/1	0/0	1/1	0/0	0/0	0/0	1/1	0/0	0/0	0/0	0/0	0/1	0/0	1/1	0/0	0/0	0/0	1/1	0/0	0/0	0/0	0/0	0/0
0/0	0/0	0/0	0/0	0/0	0/0	0/0	0/0	0/0	1/1	0/0	0/0	0/0	0/0	0/0	0/0	0/0	0/0	0/0	0/0	0/0	0/0	0/0	0/0	0/0	0/0	0/0
0/0	1/1	0/0	0/0	1/1	0/0	1/1	0/0	0/0	0/0	1/1	0/0	0/0	0/0	0/0	1/1	0/0	1/1	0/0	0/0	0/0	1/1	0/0	0/0	0/0	0/0	0/0
1/1	0/0	1/1	1/1	0/0	1/1	0/0	1/1	1/1	0/0	0/0	1/1	1/1	1/1	1/1	0/0	0/0	0/0	1/1	0/0	0/0	0/0	1/1	0/0	0/0	0/0	0/0
0/0	0/0	0/0	0/0	0/0	0/0	0/0	0/0	0/0	1/1	0/0	0/0	0/0	0/0	0/0	0/0	0/0	0/0	0/0	0/0	0/0	0/0	0/0	0/0	0/0	0/0	0/0
1/1	1/1	1/1	1/1	0/0	1/1	0/0	1/1	1/1	2/2	1/1	1/1	1/1	1/1	1/1	0/0	0/0	0/0	1/1	0/0	0/0	1/1	1/1	0/0	0/0	0/0	0/0
0/0	0/0	0/0	0/0	0/0	0/0	0/0	0/0	0/0	1/1	0/0	0/0	0/0	0/0	0/0	0/0	0/0	0/0	0/0	0/0	0/0	0/0	0/0	0/0	0/0	0/0	0/0
1/1	0/0	1/1	1/1	0/0	1/1	0/0	1/1	1/1	0/0	0/0	1/1	1/1	1/1	1/1	0/0	0/0	0/0	1/1	0/0	0/0	0/0	1/1	0/0	0/0	0/0	0/0
1/1	1/1	1/1	1/1	1/1	1/1	1/1	1/1	1/1	1/1	1/1	1/1	1/1	1/1	1/1	1/1	0/0	1/1	1/1	0/0	0/0	1/1	1/1	0/0	0/0	0/0	0/0
0/0	0/0	0/0	0/0	0/0	0/0	0/0	0/0	0/0	1/1	0/0	0/0	0/0	0/0	0/0	0/0	0/0	0/0	0/0	0/0	0/0	0/0	0/0	0/0	0/0	0/0	0/0
0/0	0/0	0/0	0/0	0/0	0/0	0/0	0/0	0/0	0/0	0/0	0/0	0/0	0/0	0/0	0/0	0/0	0/0	0/0	0/0	0/0	0/0	0/0	0/0	0/0	0/0	0/0
1/1	1/1	1/1	1/1	1/1	1/1	1/1	1/1	1/1	1/1	1/1	1/1	1/1	1/1	1/1	1/1	0/0	1/1	1/1	0/0	0/0	1/1	1/1	0/0	0/0	0/0	0/0
1/1	0/0	1/1	1/1	0/0	1/1	0/0	1/1	1/1	0/0	0/0	1/1	1/1	1/1	1/1	0/0	0/0	0/0	1/1	0/0	0/0	0/0	1/1	0/0	0/0	0/0	0/0
0/0	0/0	0/0	0/0	0/0	0/0	0/0	0/0	0/0	1/1	0/0	0/0	0/0	0/0	0/0	0/0	0/0	0/0	0/0	0/0	0/0	0/0	0/0	0/0	0/0	0/0	0/0
0/0	1/1	0/0	0/0	1/1	0/0	1/1	0/0	0/0	0/0	1/1	0/0	0/0	0/0	0/0	1/1	0/0	1/1	0/0	0/0	0/0	1/1	0/0	0/0	0/0	0/0	0/0
0/0	0/0	0/0	0/0	1/1	0/0	1/1	0/0	0/0	0/0	0/0	0/0	0/0	0/0	0/0	1/1	0/0	1/1	0/0	0/0	0/0	0/0	0/0	0/0	0/0	0/0	0/0
0/0	0/0	0/0	0/0	0/0	0/0	0/0	0/0	0/0	1/1	0/0	0/0	0/0	0/0	0/0	0/0	0/0	0/0	0/0	0/0	0/0	0/0	0/0	0/0	0/0	0/0	0/0
0/0	1/1	0/0	0/0	1/1	0/0	1/1	0/0	0/0	0/0	1/1	0/0	0/0	0/0	0/0	1/1	0/0	1/1	0/0	0/0	0/0	1/1	0/0	0/0	0/0	0/0	0/0
0/0	0/0	0/0	0/0	0/0	0/0	0/0	0/0	0/0	1/1	0/0	0/0	0/0	0/0	0/0	0/0	0/0	0/0	0/0	0/0	0/0	0/0	0/0	0/0	0/0	0/0	0/0
1/1	1/1	1/1	1/1	1/1	1/1	1/1	1/1	1/1	1/1	1/1	1/1	1/1	1/1	1/1	1/1	0/0	1/1	1/1	0/0	0/0	1/1	1/1	0/0	0/0	0/0	0/0
1/1	0/0	1/1	1/1	0/0	1/1	0/0	1/1	1/1	0/0	0/0	1/1	1/1	1/1	1/1	0/0	0/0	0/0	1/1	0/0	0/0	0/0	1/1	0/0	0/0	0/0	0/0
0/0	0/0	0/0	0/0	1/1	0/0	1/1	0/0	0/0	2/2	0/0	0/0	0/0	0/0	0/0	1/1	0/0	1/1	0/0	0/0	0/0	0/0	0/0	0/0	0/0	0/0	0/0

1/1	1/1	1/1	1/1	1/1	1/1	1/1	1/1	1/1	1/1	1/1	1/1	1/1	1/1	1/1	1/1	1/1	1/1	1/1	0/0	0/0	1/1	1/1	0/0	0/0	0/0	0/0
1/1	0/0	1/1	1/1	0/0	1/1	0/0	1/1	1/1	1/1	0/0	1/1	1/1	1/1	1/1	0/0	0/0	0/0	1/1	0/0	0/0	0/0	1/1	0/0	0/0	0/0	0/0
1/1	0/0	1/1	1/1	0/0	1/1	0/0	1/1	1/1	0/0	0/0	1/1	1/1	1/1	1/1	0/0	0/0	0/0	1/1	0/0	0/0	0/0	1/1	0/0	0/0	0/0	0/0
0/0	0/0	0/0	0/0	0/0	0/0	0/0	0/0	0/0	0/0	0/0	0/0	0/0	0/0	0/0	0/0	0/0	0/0	0/0	0/0	0/0	0/0	0/0	0/0	0/0	0/0	0/0
1/1	1/1	1/1	1/1	1/1	1/1	1/1	1/1	1/1	1/1	1/1	1/1	1/1	1/1	1/1	1/1	0/0	1/1	1/1	0/0	0/0	1/1	1/1	0/0	0/0	0/0	0/0
1/1	1/1	1/1	1/1	1/1	1/1	1/1	1/1	1/1	1/1	1/1	1/1	1/1	1/1	1/1	1/1	0/0	1/1	1/1	0/0	0/0	1/1	1/1	0/0	0/0	0/0	0/0
1/1	1/1	1/1	1/1	1/1	1/1	1/1	1/1	1/1	1/1	1/1	1/1	1/1	1/1	1/1	1/1	0/0	1/1	1/1	0/0	0/0	1/1	1/1	0/0	0/0	0/0	0/0
0/0	0/0	0/0	0/0	0/0	0/0	0/0	0/0	0/0	0/0	0/0	0/0	0/0	0/0	0/0	0/0	0/0	0/0	0/0	0/0	0/0	0/0	0/0	0/0	0/0	0/0	0/0
1/1	0/0	1/1	1/1	0/0	1/1	0/0	1/1	1/1	1/1	0/0	1/1	1/1	1/1	1/1	0/0	0/0	0/0	1/1	0/0	0/0	0/0	1/1	0/0	0/0	0/0	0/0
0/0	0/0	0/0	0/0	0/0	0/0	0/0	0/0	0/0	0/0	0/0	0/0	0/0	0/0	0/0	0/0	0/0	0/0	0/0	0/0	0/0	0/0	0/0	0/0	0/0	0/0	0/0
0/0	1/1	0/0	0/0	1/1	0/0	1/1	0/0	0/0	0/0	1/1	0/0	0/0	0/0	0/0	1/1	0/0	1/1	0/0	0/0	0/0	1/1	0/0	0/0	0/0	0/0	0/0
0/0	0/0	0/0	0/0	0/0	0/0	0/0	0/0	0/0	0/0	0/0	0/0	0/0	0/0	0/0	0/0	0/0	0/0	0/0	0/0	0/0	0/0	0/0	0/0	0/0	0/0	0/0
0/0	0/0	0/0	0/0	0/0	0/0	0/0	0/0	0/0	0/0	0/0	0/0	0/0	0/0	0/0	0/0	0/0	0/0	0/0	0/0	0/0	0/0	0/0	0/0	0/0	0/0	0/0
0/0	1/1	0/0	0/0	0/0	0/0	0/0	0/0	0/0	0/0	1/1	0/0	0/0	0/0	0/0	0/0	0/0	0/0	0/0	0/0	0/0	1/1	0/0	0/0	0/0	0/0	0/0
1/1	1/1	1/1	1/1	1/1	1/1	1/1	1/1	1/1	1/1	1/1	1/1	1/1	1/1	1/1	1/1	0/0	1/1	1/1	0/0	0/0	1/1	1/1	0/0	0/0	0/0	0/0
0/0	0/0	0/0	0/0	0/0	0/0	0/0	0/0	0/0	0/0	0/0	0/0	0/0	0/0	0/0	0/0	0/0	0/0	0/0	0/0	0/0	0/0	0/0	0/0	0/0	0/0	0/0
0/0	0/0	0/0	0/0	0/0	0/0	0/0	0/0	0/0	0/0	0/0	0/0	0/0	0/0	0/0	0/0	0/0	0/0	0/0	0/0	0/0	0/0	0/0	0/0	0/0	0/0	0/0
0/0	0/0	0/0	1/1	0/0	0/0	0/0	0/0	0/0	0/0	0/0	0/0	0/0	0/0	0/0	0/0	0/0	0/0	0/0	0/0	0/0	0/0	0/0	0/0	0/0	0/0	0/0
0/0	1/1	0/0	0/0	1/1	0/0	1/1	0/0	0/0	0/0	1/1	0/0	0/0	0/0	0/0	1/1	0/0	1/1	0/0	0/0	0/0	1/1	0/0	0/0	0/0	0/0	0/0
0/0	0/0	0/0	0/0	0/0	0/0	0/0	0/0	0/0	0/0	0/0	0/0	0/0	0/0	0/0	0/0	0/0	0/0	0/0	0/0	0/0	0/0	0/0	0/0	0/0	0/0	0/0
1/1	1/1	1/1	1/1	1/1	1/1	1/1	1/1	1/1	1/1	1/1	1/1	1/1	1/1	1/1	1/1	0/0	1/1	1/1	0/0	0/0	1/1	1/1	0/0	0/0	0/0	0/0
0/0	1/1	0/0	0/0	1/1	0/0	1/1	0/0	0/0	0/0	1/1	0/0	0/0	0/0	0/0	1/1	0/0	1/1	0/0	0/0	0/0	1/1	0/0	0/0	0/0	0/0	0/0
0/0	1/1	0/0	0/0	1/1	0/0	1/1	0/0	0/0	0/0	1/1	0/0	0/0	0/0	0/0	1/1	0/0	1/1	0/0	0/0	0/0	1/1	0/0	0/0	0/0	0/0	0/0
1/1	0/0	1/1	1/1	0/0	1/1	0/0	1/1	1/1	1/1	0/0	1/1	1/1	1/1	1/1	0/0	0/0	0/0	1/1	0/0	0/0	0/0	1/1	0/0	0/0	0/0	0/0
1/1	1/1	1/1	1/1	1/1	1/1	1/1	1/1	1/1	1/1	1/1	1/1	1/1	1/1	1/1	1/1	0/0	1/1	1/1	0/0	0/0	1/1	1/1	0/0	0/0	0/0	0/0
1/1	0/0	1/1	1/1	0/0	1/1	0/0	1/1	1/1	0/0	0/0	1/1	1/1	1/1	1/1	0/0	0/0	0/0	1/1	0/0	0/0	0/0	1/1	0/0	0/0	0/0	0/0
0/0	0/0	0/0	0/0	0/0	0/0	0/0	0/0	0/0	0/0	0/0	0/0	0/0	0/0	0/0	0/0	0/0	0/0	0/0	0/0	0/0	0/0	0/0	0/0	0/0	0/0	0/0
0/0	0/0	0/0	0/0	0/0	0/0	0/0	0/0	0/0	1/1	0/0	0/0	0/0	0/0	0/0	0/0	0/0	0/0	0/0	0/0	0/0	0/0	0/0	0/0	0/0	0/0	0/0
1/1	1/1	1/1	1/1	1/1	1/1	1/1	1/1	1/1	1/1	1/1	1/1	1/1	1/1	1/1	1/1	1/1	1/1	1/1	0/0	0/0	1/1	1/1	0/0	0/0	0/0	0/0
0/0	0/0	0/0	0/0	0/0	0/0	0/0	0/0	0/0	0/0	0/0	0/0	0/0	0/0	0/0	0/0	0/0	0/0	0/0	0/0	0/0	0/0	0/0	0/0	0/0	0/0	0/0
1/1	1/1	1/1	1/1	1/1	1/1	1/1	1/1	1/1	1/1	1/1	1/1	1/1	1/1	1/1	0/1	0/0	1/1	1/1	0/0	0/0	1/1	1/1	0/0	0/0	0/0	0/0
1/1	0/0	1/1	1/1	0/0	1/1	0/0	1/1	1/1	0/0	0/0	1/1	1/1	1/1	1/1	0/0	0/0	0/0	1/1	0/0	0/0	0/0	1/1	0/0	0/0	0/0	0/0
1/1	1/1	1/1	1/1	1/1	1/1	1/1	1/1	1/1	1/1	1/1	1/1	1/1	1/1	1/1	0/1	0/0	1/1	1/1	0/0	0/0	1/1	1/1	0/0	0/0	0/0	0/0
0/0	0/0	0/0	0/0	0/0	0/0	0/0	0/0	0/0	0/0	0/0	0/0	0/0	0/0	0/0	0/0	0/0	0/0	0/0	0/0	0/0	0/0	0/0	0/0	0/0	0/0	0/0
1/1	0/0	1/1	1/1	0/0	1/1	0/0	1/1	1/1	1/1	0/0	1/1	1/1	1/1	1/1	0/0	0/0	0/0	1/1	0/0	0/0	0/0	1/1	0/0	0/0	0/0	0/0

0/0	1/1	0/0	0/0	1/1	0/0	1/1	0/0	0/0	0/0	1/1	0/0	0/0	0/0	0/0	0/1	0/0	1/1	0/0	0/0	0/0	1/1	0/0	0/0	0/0	0/0	0/0
0/0	0/0	0/0	0/0	0/0	0/0	0/0	0/0	0/0	0/0	0/0	0/0	0/0	0/0	0/0	0/0	0/0	0/0	0/0	0/0	0/0	0/0	0/0	0/0	0/0	0/0	0/0
0/0	0/0	0/0	0/0	1/1	0/0	1/1	0/0	0/0	2/2	0/0	0/0	0/0	0/0	0/0	0/1	0/0	1/1	0/0	0/0	0/0	0/0	0/0	0/0	0/0	0/0	0/0
1/1	1/1	1/1	1/1	1/1	1/1	1/1	1/1	1/1	1/1	1/1	1/1	1/1	1/1	1/1	0/1	0/0	1/1	1/1	0/0	0/0	1/1	1/1	0/0	0/0	0/0	0/0
0/0	0/0	0/0	0/0	0/0	0/0	0/0	0/0	0/0	0/0	0/0	0/0	0/0	0/0	0/0	0/0	0/0	0/0	0/0	0/0	0/0	0/0	0/0	0/0	0/0	0/0	0/0
0/0	1/1	0/0	0/0	1/1	0/0	1/1	0/0	0/0	0/0	1/1	0/0	0/0	0/0	0/0	1/1	0/0	1/1	0/0	0/0	0/0	1/1	0/0	0/0	0/0	0/0	0/0
1/1	1/1	1/1	1/1	1/1	1/1	1/1	1/1	1/1	1/1	1/1	1/1	1/1	1/1	1/1	1/1	0/0	1/1	1/1	0/0	0/0	1/1	1/1	0/0	0/0	0/0	0/0
1/1	0/0	1/1	1/1	0/0	1/1	0/0	1/1	1/1	1/1	0/0	1/1	1/1	1/1	1/1	0/0	0/0	./.	1/1	0/0	0/0	0/0	1/1	0/0	0/0	0/0	0/0
0/0	1/1	0/0	0/0	1/1	0/0	1/1	0/0	0/0	0/0	1/1	0/0	0/0	0/0	0/0	1/1	0/0	1/1	0/0	0/0	0/0	1/1	0/0	0/0	0/0	0/0	0/0
0/0	0/0	0/0	0/0	0/0	0/0	0/0	0/0	0/0	1/1	0/0	0/0	0/0	0/0	0/0	0/0	0/0	0/0	0/0	0/0	0/0	0/0	0/0	0/0	0/0	0/0	0/0
0/0	1/1	0/0	0/0	1/1	0/0	1/1	0/0	0/0	0/0	1/1	0/0	0/0	0/0	0/0	1/1	0/0	1/1	0/0	0/0	0/0	1/1	0/0	0/0	0/0	0/0	0/0
0/0	0/0	0/0	0/0	0/0	0/0	0/0	0/0	0/0	0/0	0/0	0/0	0/0	0/0	0/0	0/0	0/0	0/0	0/0	0/0	0/0	0/0	0/0	0/0	0/0	0/0	0/0
0/0	0/0	0/0	0/0	0/0	0/0	0/0	0/0	0/0	0/0	0/0	0/0	0/0	0/0	0/0	0/0	0/0	0/0	0/0	0/0	0/0	0/0	0/0	0/0	0/0	0/0	0/0
0/0	0/0	0/0	0/0	0/0	0/0	0/0	0/0	0/0	0/0	0/0	0/0	0/0	0/0	0/0	0/0	0/0	0/0	0/0	0/0	0/0	0/0	0/0	0/0	0/0	0/0	0/0
1/1	1/1	1/1	1/1	1/1	1/1	1/1	1/1	1/1	1/1	1/1	1/1	1/1	1/1	1/1	1/1	1/1	1/1	1/1	0/0	0/0	1/1	1/1	0/0	0/0	0/0	0/0
1/1	1/1	1/1	1/1	1/1	1/1	1/1	1/1	1/1	1/1	1/1	1/1	1/1	1/1	1/1	1/1	1/1	1/1	1/1	0/0	0/0	1/1	1/1	0/0	0/0	0/0	0/0
1/1	1/1	1/1	1/1	1/1	1/1	1/1	1/1	1/1	1/1	1/1	1/1	1/1	1/1	1/1	1/1	0/0	1/1	1/1	0/0	0/0	1/1	1/1	0/0	0/0	0/0	0/0
./.	./.	./.	0/0	./.	0/0	./.	./.	0/0	./.	./.	./.	./.	./.	./.	1/1	./.	./.	0/0	0/0	0/0	./.	./.	0/0	0/0	0/0	0/0
3/3	1/1	3/3	3/3	1/1	3/3	1/1	3/3	3/3	1/1	1/1	3/3	3/3	3/3	3/3	4/4	1/1	1/1	3/3	0/0	0/0	1/1	3/3	0/0	0/0	0/0	0/0
0/0	0/0	0/1	0/0	0/0	1/1	0/0	1/1	1/1	0/0	0/0	1/1	1/1	0/0	1/1	0/0	0/0	0/0	0/0	0/0	0/0	0/0	0/0	0/0	0/0	0/0	0/0
0/0	1/1	0/0	0/0	0/0	0/0	0/0	0/0	0/0	0/0	0/0	0/0	0/0	0/0	0/0	0/0	0/0	0/0	0/0	0/0	0/0	0/0	0/0	0/0	0/0	0/0	0/0
0/0	0/0	0/0	0/0	0/0	0/0	0/0	0/0	0/0	0/0	1/1	0/0	0/0	0/0	0/0	0/0	0/0	0/0	0/0	0/0	0/0	0/0	0/0	0/0	0/0	0/0	0/0
0/0	0/0	0/0	0/0	0/0	0/0	0/0	0/0	0/0	0/0	0/0	0/0	0/0	0/0	0/0	0/0	0/0	0/0	0/0	0/0	0/0	0/0	0/0	0/0	0/0	0/0	0/0
0/0	0/0	0/0	0/0	0/0	./.	0/0	0/0	0/0	0/0	0/0	0/0	0/0	0/0	0/0	0/0	0/0	0/0	0/0	0/0	0/0	0/0	0/0	0/0	0/0	0/0	0/0
1/1	0/0	1/1	1/1	0/0	1/1	0/0	1/1	1/1	0/0	0/0	1/1	1/1	1/1	1/1	0/0	0/0	0/0	1/1	0/0	0/0	0/0	1/1	0/0	0/0	0/0	0/0

0/0	0/0	0/0	0/0	0/0	0/0	0/0	0/0	0/0	0/0	0/0	0/0	0/0	0/0	0/0	0/0	0/0	0/0	0/0	0/0	0/0	0/0	0/0	0/0	0/0	0/0	0/0
1/1	0/0	1/1	1/1	0/0	1/1	0/0	1/1	1/1	1/1	0/0	1/1	1/1	1/1	1/1	0/0	0/0	0/0	1/1	0/0	0/0	0/0	1/1	0/0	0/0	0/0	0/0
1/1	0/0	1/1	1/1	0/0	1/1	0/0	1/1	1/1	1/1	0/0	1/1	1/1	1/1	1/1	0/0	0/0	0/0	1/1	0/0	0/0	0/0	1/1	0/0	0/0	0/0	0/0
1/1	1/1	1/1	1/1	1/1	1/1	1/1	1/1	1/1	1/1	1/1	1/1	1/1	1/1	1/1	1/1	1/1	1/1	1/1	0/0	0/0	1/1	1/1	0/0	0/0	0/0	0/0
1/1	1/1	1/1	1/1	1/1	1/1	1/1	1/1	1/1	1/1	1/1	1/1	1/1	1/1	1/1	1/1	0/0	1/1	1/1	0/0	0/0	1/1	1/1	0/0	0/0	0/0	0/0
1/1	0/0	0/0	0/0	0/0	0/0	0/0	0/0	0/0	0/0	0/0	0/0	0/0	0/0	0/0	0/0	0/0	0/0	0/0	0/0	0/0	0/0	0/0	0/0	0/0	0/0	0/0
1/1	1/1	1/1	1/1	1/1	1/1	1/1	1/1	1/1	1/1	1/1	1/1	1/1	1/1	1/1	1/1	0/0	1/1	1/1	0/0	0/0	1/1	1/1	0/0	0/0	0/0	0/0
1/1	0/0	1/1	1/1	0/0	1/1	0/0	1/1	1/1	1/1	0/0	1/1	1/1	1/1	1/1	0/0	0/0	0/0	1/1	0/0	0/0	0/0	1/1	0/0	0/0	0/0	0/0
0/0	0/0	0/0	0/0	0/0	0/0	0/0	0/0	0/0	0/0	0/0	0/0	0/0	0/0	0/0	0/0	0/0	0/0	0/0	0/0	0/0	0/0	0/0	0/0	0/0	0/0	0/0
0/0	0/0	0/0	0/0	0/0	0/0	0/0	0/0	0/0	1/1	0/0	0/0	0/0	0/0	0/0	0/0	0/0	0/0	0/0	0/0	0/0	0/0	0/0	0/0	0/0	0/0	0/0
1/1	0/0	1/1	1/1	0/0	1/1	0/0	1/1	1/1	1/1	0/0	1/1	1/1	1/1	1/1	0/0	0/0	0/0	1/1	0/0	0/0	0/0	1/1	0/0	0/0	0/0	0/0
0/0	0/0	0/0	0/0	0/0	0/0	0/0	0/0	0/0	0/0	0/0	0/0	0/0	0/0	0/0	0/0	0/0	0/0	0/0	0/0	0/0	0/0	0/0	0/0	0/0	0/0	0/0
0/0	1/1	0/0	0/0	1/1	0/0	1/1	0/0	0/0	0/0	1/1	0/0	0/0	0/0	0/0	1/1	0/0	1/1	0/0	0/0	0/0	1/1	0/0	0/0	0/0	0/0	0/0
1/1	0/0	1/1	1/1	0/0	1/1	0/0	1/1	1/1	1/1	0/0	1/1	1/1	1/1	1/1	0/0	0/0	0/0	1/1	0/0	0/0	0/0	1/1	0/0	0/0	0/0	0/0
0/0	0/0	0/0	0/0	0/0	0/0	0/0	0/0	0/0	0/0	0/0	0/0	0/0	0/0	0/0	0/0	0/0	0/0	0/0	0/0	0/0	0/0	0/0	0/0	0/0	0/0	0/0
1/1	1/1	1/1	1/1	1/1	1/1	1/1	1/1	1/1	1/1	1/1	1/1	1/1	1/1	1/1	0/1	0/0	1/1	1/1	0/0	0/0	1/1	1/1	0/0	0/0	0/0	0/0
0/0	1/1	0/0	0/0	1/1	0/0	1/1	0/0	0/0	0/0	1/1	0/0	0/0	0/0	0/0	0/1	0/0	1/1	0/0	0/0	0/0	1/1	0/0	0/0	0/0	0/0	0/0
0/0	1/1	0/0	0/0	0/0	0/0	0/0	0/0	0/0	0/0	1/1	0/0	0/0	0/0	0/0	0/0	0/0	0/0	0/0	0/0	0/0	1/1	0/0	0/0	0/0	0/0	0/0
0/0	0/0	0/0	0/0	0/0	0/0	0/0	0/0	0/0	1/1	0/0	0/0	0/0	0/0	0/0	0/0	0/0	0/0	0/0	0/0	0/0	0/0	0/0	0/0	0/0	0/0	0/0
1/1	0/0	1/1	1/1	0/0	1/1	0/0	1/1	1/1	1/1	0/0	1/1	1/1	1/1	1/1	0/0	0/0	0/0	1/1	0/0	0/0	0/0	1/1	0/0	0/0	0/0	0/0
1/1	0/0	1/1	1/1	0/0	1/1	0/0	1/1	1/1	1/1	0/0	1/1	1/1	1/1	1/1	0/0	0/0	0/0	1/1	0/0	0/0	0/0	1/1	0/0	0/0	0/0	0/0
0/0	0/0	0/0	0/0	0/0	0/0	0/0	0/0	0/0	0/0	0/0	0/0	0/0	0/0	0/0	0/0	0/0	0/0	0/0	0/0	0/0	0/0	0/0	0/0	0/0	0/0	0/0
1/1	1/1	1/1	1/1	1/1	1/1	1/1	1/1	1/1	1/1	1/1	1/1	1/1	1/1	1/1	1/1	0/0	1/1	1/1	0/0	0/0	1/1	1/1	0/0	0/0	0/0	0/0
0/0	0/0	0/0	0/0	0/0	0/0	0/0	0/0	0/0	1/1	0/0	0/0	0/0	0/0	0/0	0/0	0/0	0/0	0/0	0/0	0/0	0/0	0/0	0/0	0/0	0/0	0/0
0/0	0/0	0/0	0/0	0/0	0/0	0/0	0/0	0/0	0/0	0/0	0/0	0/0	0/0	0/0	0/0	0/0	0/0	0/0	0/0	0/0	0/0	0/0	0/0	0/0	0/0	0/0
1/1	0/0	1/1	1/1	0/0	1/1	0/0	1/1	1/1	1/1	0/0	1/1	1/1	1/1	1/1	0/0	0/0	0/0	1/1	0/0	0/0	0/0	1/1	0/0	0/0	0/0	0/0
0/0	0/0	0/0	0/0	0/0	0/0	0/0	0/0	0/0	0/0	0/0	0/0	0/0	0/0	0/0	0/0	0/0	0/0	0/0	0/0	0/0	0/0	0/0	0/0	0/0	0/0	0/0
0/0	1/1	0/0	0/0	1/1	0/0	1/1	0/0	0/0	0/0	1/1	0/0	0/0	0/0	0/0	1/1	0/0	1/1	0/0	0/0	0/0	1/1	0/0	0/0	0/0	0/0	0/0
1/1	0/0	1/1	1/1	0/0	1/1	0/0	1/1	1/1	1/1	0/0	1/1	1/1	1/1	1/1	0/0	0/0	0/0	1/1	0/0	0/0	0/0	1/1	0/0	0/0	0/0	0/0
0/0	0/0	0/0	0/0	0/0	0/0	0/0	0/0	0/0	1/1	0/0	0/0	0/0	0/0	0/0	0/0	0/0	0/0	0/0	0/0	0/0	0/0	0/0	0/0	0/0	0/0	0/0
1/1	1/1	1/1	1/1	1/1	1/1	1/1	1/1	1/1	1/1	1/1	1/1	1/1	1/1	1/1	1/1	0/0	1/1	1/1	0/0	0/0	1/1	1/1	0/0	0/0	0/0	0/0
0/0	0/0	0/0	0/0	0/0	0/0	0/0	0/0	0/0	0/0	0/0	0/0	0/0	0/0	0/0	0/0	0/0	0/0	0/0	0/0	0/0	0/0	0/0	0/0	0/0	0/0	0/0
0/0	0/0	0/0	0/0	0/0	0/0	0/0	0/0	0/0	0/0	0/0	0/0	0/0	0/0	0/0	0/0	1/1	0/0	0/0	0/0	0/0	0/0	0/0	0/0	0/0	0/0	0/0

1/1	1/1	1/1	1/1	1/1	1/1	1/1	1/1	1/1	1/1	1/1	1/1	1/1	1/1	1/1	0/1	0/0	1/1	1/1	0/0	0/0	1/1	1/1	0/0	0/0	0/0	0/0
0/0	0/0	0/0	0/0	0/0	0/0	0/0	0/0	0/0	0/0	0/0	0/0	0/0	0/0	0/0	0/0	0/0	0/0	0/0	0/0	0/0	0/0	0/0	0/0	0/0	0/0	0/0
1/1	1/1	1/1	1/1	1/1	1/1	1/1	1/1	1/1	1/1	1/1	1/1	1/1	1/1	1/1	0/1	0/0	1/1	1/1	0/0	0/0	1/1	1/1	0/0	0/0	0/0	0/0
0/0	0/0	0/0	0/0	0/0	0/0	0/0	0/0	0/0	0/0	0/0	0/0	0/0	0/0	0/0	0/0	0/0	0/0	0/0	0/0	0/0	0/0	0/0	0/0	0/0	0/0	0/0
0/0	0/0	0/0	0/0	1/1	0/0	1/1	0/0	0/0	0/0	0/0	0/0	0/0	0/0	0/0	0/1	0/0	1/1	0/0	0/0	0/0	0/0	0/0	0/0	0/0	0/0	0/0
0/0	0/0	0/0	0/0	0/0	0/0	0/0	0/0	0/0	0/0	0/0	0/0	0/0	0/0	0/0	0/0	0/0	0/0	0/0	0/0	0/0	0/0	0/0	0/0	0/0	0/0	0/0
0/0	0/0	0/0	0/0	0/0	0/0	0/0	0/0	0/0	0/0	0/0	0/0	0/0	0/0	0/0	0/0	0/0	0/0	0/0	0/0	0/0	0/0	0/0	0/0	0/0	0/0	0/0
0/0	1/1	0/0	0/0	0/0	0/0	0/0	0/0	0/0	0/0	1/1	0/0	0/0	0/0	0/0	0/0	0/0	0/0	0/0	0/0	0/0	1/1	0/0	0/0	0/0	0/0	0/0
0/0	0/0	0/0	0/0	0/0	0/0	0/0	0/0	0/0	0/0	0/0	0/0	0/0	0/0	0/0	0/0	0/0	0/0	0/0	0/0	0/0	0/0	0/0	0/0	0/0	0/0	0/0
0/0	0/0	0/0	0/0	0/0	0/0	0/0	0/0	0/0	0/0	0/0	0/0	0/0	0/0	0/0	0/0	0/0	0/0	0/0	0/0	0/0	0/0	0/0	0/0	0/0	0/0	0/0
1/1	1/1	1/1	1/1	1/1	1/1	1/1	1/1	1/1	1/1	1/1	1/1	1/1	1/1	1/1	1/1	0/0	1/1	1/1	0/0	0/0	1/1	1/1	0/0	0/0	0/0	0/0
1/1	1/1	1/1	1/1	1/1	1/1	1/1	1/1	1/1	1/1	1/1	1/1	1/1	1/1	1/1	1/1	0/0	1/1	1/1	0/0	0/0	1/1	1/1	0/0	0/0	0/0	0/0
0/0	0/0	0/0	0/0	0/0	0/0	0/0	0/0	0/0	0/0	0/0	0/0	0/0	0/0	0/0	0/0	0/0	0/0	0/0	0/0	0/0	0/0	0/0	0/0	0/0	0/0	0/0
0/0	0/0	0/0	0/0	1/1	0/0	1/1	0/0	0/0	0/0	0/0	0/0	0/0	0/0	0/0	1/1	0/0	1/1	0/0	0/0	0/0	0/0	0/0	0/0	0/0	0/0	0/0
1/1	0/0	1/1	1/1	0/0	1/1	0/0	1/1	1/1	1/1	0/0	1/1	1/1	1/1	1/1	0/0	0/0	0/0	1/1	0/0	0/0	0/0	1/1	0/0	0/0	0/0	0/0
1/1	1/1	1/1	1/1	1/1	1/1	1/1	1/1	1/1	2/2	1/1	1/1	1/1	1/1	1/1	1/1	2/2	1/1	1/1	0/0	0/0	1/1	1/1	0/0	0/0	0/0	0/0
1/1	1/1	1/1	1/1	1/1	1/1	1/1	1/1	1/1	1/1	1/1	1/1	1/1	1/1	1/1	1/1	0/0	1/1	1/1	0/0	0/0	1/1	1/1	0/0	0/0	0/0	0/0
1/1	0/0	1/1	1/1	0/0	1/1	0/0	1/1	1/1	0/0	0/0	1/1	1/1	1/1	1/1	0/0	0/0	0/0	1/1	0/0	0/0	0/0	1/1	0/0	0/0	0/0	0/0
1/1	0/0	1/1	1/1	0/0	1/1	0/0	1/1	1/1	0/0	0/0	1/1	1/1	1/1	1/1	0/0	0/0	0/0	1/1	0/0	0/0	0/0	1/1	0/0	0/0	0/0	0/0
0/0	0/0	0/0	0/0	1/1	0/0	1/1	0/0	0/0	0/0	0/0	0/0	0/0	0/0	0/0	1/1	0/0	1/1	0/0	0/0	0/0	0/0	0/0	0/0	0/0	0/0	0/0
0/0	0/0	0/0	0/0	0/0	0/0	0/0	0/0	0/0	0/0	0/0	0/0	0/0	0/0	0/0	0/0	0/0	0/0	0/0	0/0	0/0	0/0	0/0	0/0	0/0	0/0	0/0
0/0	0/0	0/0	0/0	0/0	0/0	0/0	0/0	0/0	0/0	0/0	0/0	0/0	0/0	0/0	0/0	0/0	0/0	0/0	0/0	0/0	0/0	0/0	0/0	0/0	0/0	0/0
0/0	0/0	0/0	0/0	0/0	0/0	0/0	0/0	0/0	0/0	0/0	0/0	0/0	0/0	0/0	0/0	0/0	0/0	0/0	0/1	0/0	0/0	0/0	0/0	0/0	0/0	0/0
0/0	0/0	0/0	0/0	0/0	0/0	0/0	0/0	0/0	0/0	0/0	0/0	0/0	0/0	0/0	0/0	0/0	0/0	0/0	0/0	0/0	0/0	0/0	0/0	0/0	0/0	0/0
0/0	0/0	0/0	0/0	0/0	0/0	0/0	0/0	0/0	0/0	0/0	0/0	0/0	0/0	0/0	0/1	0/1	0/0	0/0	0/1	0/0	0/0	0/0	0/0	0/0	0/0	0/0
0/0	0/0	0/0	0/0	0/0	0/0	0/0	0/0	0/0	0/0	0/1	0/0	0/0	0/0	0/1	0/1	0/1	0/0	0/0	0/1	0/0	0/1	0/0	0/0	0/0	0/0	0/0

0/0	0/0	0/0	0/0	0/0	0/0	0/0	0/0	0/0	0/0	0/0	0/0	0/0	0/0	0/0	0/1	0/1	0/0	0/0	0/1	0/0	0/1	0/0	0/0	0/0	0/0	0/0
0/0	0/0	0/0	0/0	0/0	0/0	0/0	0/0	0/0	0/0	0/0	0/0	0/0	0/0	0/1	0/1	0/1	0/0	0/0	0/1	0/0	0/1	0/0	0/0	0/0	0/0	0/0
0/0	0/0	0/0	0/0	0/0	0/0	0/0	0/0	0/0	0/0	0/0	0/0	0/0	0/0	0/1	0/1	0/1	0/0	0/0	0/1	0/0	0/0	0/0	0/0	0/0	0/0	0/0
0/0	0/0	0/0	0/0	0/0	0/0	0/0	0/0	0/0	0/0	0/0	0/0	0/0	0/0	0/1	0/1	0/1	0/0	0/0	0/1	0/0	0/0	0/0	0/0	0/0	0/0	0/0
0/0	0/0	0/0	0/0	0/0	0/0	0/0	0/0	0/0	0/0	0/0	0/0	0/0	0/0	0/0	0/1	0/1	0/0	0/0	0/1	0/0	0/0	0/0	0/0	0/0	0/0	0/0
0/0	0/0	0/0	0/0	0/0	0/0	0/0	0/0	0/0	0/0	0/0	0/0	0/0	0/0	0/0	0/1	0/0	0/0	0/0	0/1	0/0	0/0	0/0	0/0	0/0	0/0	0/0
0/0	0/0	0/0	0/0	0/0	0/0	0/0	0/0	0/0	0/0	0/0	0/0	0/0	0/0	0/0	0/0	0/1	0/0	0/0	0/0	0/0	0/0	0/0	0/0	0/0	0/0	0/0
0/0	0/0	0/0	0/0	0/0	0/0	0/0	0/0	0/0	0/0	0/0	0/0	0/0	0/0	0/0	0/1	0/2	0/0	0/0	0/0	0/0	0/0	0/0	0/0	0/0	0/0	0/0
0/0	0/0	0/0	0/0	0/0	0/0	0/0	0/0	0/0	0/0	0/0	0/0	0/0	0/0	0/0	0/0	0/2	0/0	0/0	0/0	0/0	0/0	0/0	0/0	0/0	0/0	0/0
0/0	0/0	0/0	0/0	0/0	0/0	0/0	0/0	0/0	0/0	0/0	0/0	0/0	0/0	0/0	0/0	0/0	0/0	0/0	0/0	0/0	0/0	0/0	0/0	0/0	0/0	0/0
1/1	1/1	1/1	1/1	1/1	1/1	1/1	1/1	1/1	1/1	1/1	1/1	1/1	1/1	1/1	1/1	0/0	1/1	1/1	0/0	0/0	1/1	1/1	0/0	0/0	0/0	0/0
0/0	0/0	0/0	0/0	0/0	0/0	0/0	0/0	0/0	0/0	0/0	0/0	0/0	0/0	0/0	0/0	0/0	0/0	0/0	0/0	0/0	0/0	0/0	0/0	0/0	0/0	0/0
0/0	0/0	0/0	0/0	0/0	0/0	0/0	0/0	0/0	1/1	0/0	0/0	0/0	0/0	0/0	0/0	0/0	0/0	0/0	0/0	0/0	0/0	0/0	0/0	0/0	0/0	0/0
0/0	1/1	0/0	0/0	0/0	0/0	0/0	0/0	0/0	0/0	1/1	0/0	0/0	0/0	0/0	0/0	0/0	0/0	0/0	0/0	0/0	1/1	0/0	0/0	0/0	0/0	0/0
0/0	0/0	0/0	0/0	0/0	0/0	0/0	0/0	0/0	0/0	0/0	0/0	0/0	0/0	0/0	0/0	0/0	0/0	0/0	0/0	0/0	0/0	0/0	0/0	0/0	0/0	0/0
0/0	0/0	0/0	0/0	0/0	0/0	0/0	0/0	0/0	0/0	0/0	0/0	0/0	0/0	0/0	0/0	0/0	0/0	0/0	0/0	0/0	0/0	0/0	0/0	0/0	0/0	0/0
1/1	1/1	1/1	1/1	1/1	1/1	1/1	1/1	1/1	1/1	1/1	1/1	1/1	1/1	1/1	1/1	0/0	1/1	1/1	0/0	0/0	1/1	1/1	0/0	0/0	0/0	0/0
./.	0/0	./.	0/0	./.	./.	0/0	0/0	./.	1/1	0/0	0/0	./.	0/0	./.	0/0	0/0	0/0	./.	0/0	0/0	0/0	0/0	0/0	0/0	0/0	0/0
./.	1/1	./.	0/0	1/1	./.	1/1	./.	./.	1/1	1/1	./.	./.	0/0	./.	1/1	0/0	1/1	0/0	0/0	0/0	1/1	./.	0/0	0/0	0/0	0/0
1/1	0/0	1/1	1/1	0/0	1/1	0/0	1/1	1/1	0/0	0/0	1/1	1/1	1/1	1/1	0/0	0/0	0/0	1/1	0/0	0/0	0/0	1/1	0/0	0/0	0/0	0/0
1/1	0/0	1/1	1/1	0/0	1/1	0/0	1/1	1/1	0/0	0/0	1/1	1/1	1/1	1/1	0/0	0/0	0/0	1/1	0/0	0/0	0/0	1/1	0/0	0/0	0/0	0/0
0/0	./.	0/0	0/0	0/0	0/0	./.	0/0	0/0	1/1	./.	0/0	0/0	0/0	0/0	0/0	0/0	./.	0/0	0/0	0/0	0/0	0/0	0/0	0/0	0/0	0/0
0/0	./.	0/0	0/0	./.	0/0	./.	0/0	0/0	2/2	./.	0/0	0/0	0/0	0/0	./.	0/0	0/0	0/0	0/0	0/0	./.	0/0	0/0	0/0	0/0	0/0
0/0	1/1	0/0	0/0	0/1	0/0	1/1	0/0	0/0	2/2	1/1	0/0	0/0	0/0	0/0	0/1	0/0	1/1	0/0	0/0	0/0	1/1	0/0	0/0	0/0	0/0	0/0

0/0	2/2	0/0	0/0	0/1	0/0	0/0	0/0	0/0	2/2	0/0	0/0	0/0	0/0	0/0	0/0	0/0	0/0	0/0	0/0	0/0	2/2	0/0	0/0	0/0	0/0	0/0
0/0	4/4	0/0	0/0	0/4	0/0	4/4	0/0	0/0	4/4	4/4	0/0	0/0	0/0	0/0	0/4	0/0	0/0	2/2	0/0	0/0	4/4	0/0	0/0	0/0	0/0	0/0
0/0	0/0	0/0	0/0	0/0	0/0	0/0	0/0	0/0	1/1	0/0	0/0	0/0	0/0	0/0	0/0	0/0	0/0	0/0	0/0	0/0	0/0	0/0	0/0	0/0	0/0	0/0
1/1	1/1	1/1	1/1	1/1	1/1	1/1	1/1	1/1	1/1	1/1	1/1	1/1	1/1	1/1	1/1	0/0	1/1	1/1	0/0	0/0	1/1	1/1	0/0	0/0	0/0	0/0
0/0	1/1	0/0	0/0	1/1	0/0	1/1	0/0	0/0	1/1	1/1	0/0	0/0	0/0	0/0	1/1	0/0	1/1	0/0	0/0	0/0	1/1	0/0	0/0	0/0	0/0	0/0
0/0	0/0	0/0	0/0	0/0	0/0	0/0	0/0	0/0	0/0	0/0	0/0	0/0	0/0	0/0	0/0	0/0	0/0	0/0	0/0	0/0	0/0	0/0	0/0	0/0	0/0	0/0
0/0	0/0	0/0	0/0	0/0	0/0	0/0	0/0	0/0	0/0	0/0	0/0	0/0	0/0	0/0	0/0	0/0	0/0	0/0	0/0	0/0	0/0	0/0	0/0	0/0	0/0	0/0
1/1	1/1	1/1	1/1	1/1	1/1	1/1	1/1	1/1	1/1	1/1	1/1	1/1	1/1	1/1	1/1	0/0	1/1	1/1	0/0	0/0	1/1	1/1	0/0	0/0	0/0	0/0
0/0	./.	0/0	0/0	0/0	0/0	0/0	0/0	0/0	0/0	0/0	0/0	0/0	0/0	0/0	./.	0/0	0/0	0/0	0/0	0/0	./.	0/0	0/0	0/0	0/0	0/0
0/0	1/1	0/0	0/0	1/1	0/0	1/1	0/0	0/0	2/2	1/1	0/0	0/0	0/0	0/0	1/1	0/0	1/1	0/0	0/0	0/0	1/1	0/0	0/0	0/0	0/0	0/0
0/0	1/1	0/0	0/0	1/1	0/0	1/1	0/0	0/0	1/1	1/1	0/0	0/0	0/0	0/0	1/1	0/0	1/1	0/0	0/0	0/0	1/1	0/0	0/0	0/0	0/0	0/0
0/0	1/1	0/0	0/0	1/1	0/0	1/1	0/0	0/0	0/0	1/1	0/0	0/0	0/0	0/0	1/1	0/0	1/1	0/0	0/0	0/0	1/1	0/0	0/0	0/0	0/0	0/0
1/1	0/0	1/1	1/1	0/0	1/1	0/0	1/1	1/1	0/0	0/0	1/1	1/1	1/1	1/1	0/0	0/0	0/0	1/1	0/0	0/0	0/0	0/1	0/0	0/0	0/0	0/0
0/0	1/1	0/0	0/0	1/1	0/0	1/1	0/0	0/0	1/1	1/1	0/0	0/0	0/0	0/0	1/1	0/0	1/1	0/0	0/0	0/0	1/1	0/0	0/0	0/0	0/0	0/0
1/1	1/1	1/1	1/1	1/1	1/1	1/1	1/1	1/1	1/1	1/1	1/1	1/1	1/1	1/1	1/1	0/0	1/1	1/1	0/0	0/0	1/1	1/1	0/0	0/0	0/0	0/0
0/0	0/0	0/0	0/0	0/0	0/0	0/0	0/0	0/0	0/0	0/0	0/0	0/0	0/0	0/0	0/0	0/0	0/0	0/0	0/0	0/0	0/0	0/0	0/0	0/0	0/0	0/0
0/0	0/0	0/0	0/0	0/0	0/0	0/0	0/0	0/0	0/0	0/0	0/0	0/0	0/0	0/0	0/0	0/0	0/0	0/0	0/0	0/0	0/0	0/0	0/0	0/0	0/0	0/0
1/1	0/0	1/1	1/1	0/0	1/1	0/0	1/1	1/1	0/0	0/0	1/1	1/1	0/1	1/1	0/0	0/0	0/0	1/1	0/0	0/0	0/0	1/1	0/0	0/0	0/0	0/0
0/0	0/0	0/0	0/0	0/0	0/0	0/0	0/0	0/0	0/0	0/0	0/0	0/0	0/0	0/0	0/0	0/0	0/0	0/0	0/0	0/0	0/0	0/0	0/0	0/0	0/0	0/0
1/1	0/0	1/1	1/1	0/0	1/1	0/0	1/1	1/1	0/0	0/0	1/1	1/1	0/1	1/1	0/0	0/0	0/0	1/1	0/0	0/0	0/0	1/1	0/0	0/0	0/0	0/0
0/0	1/1	0/0	0/0	0/0	0/0	0/0	0/0	0/0	0/0	1/1	0/0	0/0	0/0	0/0	0/0	0/0	0/0	0/0	0/0	0/0	1/1	0/0	0/0	0/0	0/0	0/0
1/1	0/0	1/1	1/1	0/0	1/1	0/0	1/1	1/1	0/0	0/0	1/1	1/1	1/1	1/1	0/0	0/0	0/0	1/1	0/0	0/0	0/0	1/1	0/0	0/0	0/0	0/0
1/1	0/0	1/1	1/1	0/0	1/1	0/0	1/1	1/1	0/0	0/0	1/1	1/1	1/1	1/1	0/0	0/0	0/0	1/1	0/0	0/0	0/0	1/1	0/0	0/0	0/0	0/0
0/0	0/0	0/0	0/0	0/0	0/0	0/0	0/0	0/0	1/1	0/0	0/0	0/0	0/0	0/0	0/0	0/0	0/0	0/0	0/0	0/0	0/0	0/0	0/0	0/0	0/0	0/0
0/0	0/0	0/0	0/0	0/0	0/0	0/0	0/0	0/0	1/1	0/0	0/0	0/0	0/0	0/0	0/0	0/0	0/0	0/0	0/0	0/0	0/0	0/0	0/0	0/0	0/0	0/0
0/0	1/1	0/0	0/0	1/1	0/0	1/1	0/0	0/0	1/1	1/1	0/0	0/0	0/0	0/0	1/1	0/0	1/1	0/0	0/0	0/0	1/1	0/0	0/0	0/0	0/0	0/0
0/0	0/0	0/0	0/0	0/0	0/0	0/0	0/0	0/0	0/0	0/0	0/0	0/0	0/0	0/0	0/0	0/0	0/0	0/0	0/0	0/0	0/0	0/0	0/0	0/0	0/0	0/0
1/1	1/1	1/1	1/1	1/1	1/1	1/1	1/1	1/1	1/1	1/1	1/1	1/1	1/1	1/1	1/1	0/0	1/1	1/1	0/0	0/0	1/1	1/1	0/0	0/0	0/0	0/0
0/0	0/0	0/0	0/0	0/0	0/0	0/0	0/0	0/0	0/0	0/0	0/0	0/0	0/0	0/0	0/0	0/0	0/0	0/0	0/0	0/0	0/0	0/0	0/0	0/0	0/0	0/0
1/1	1/1	1/1	1/1	1/1	1/1	1/1	1/1	1/1	1/1	1/1	1/1	1/1	1/1	1/1	1/1	0/0	1/1	1/1	0/0	0/0	1/1	1/1	0/0	0/0	0/0	0/0

0/0	0/0	0/0	0/0	0/0	0/0	0/0	0/0	0/0	1/1	0/0	0/0	0/0	0/0	0/0	0/0	0/0	0/0	0/0	0/0	0/0	0/0	0/0	0/0	0/0	0/0	0/0
0/0	0/0	0/0	0/0	0/0	0/0	0/0	0/0	0/0	0/0	0/0	0/0	0/0	0/0	0/0	0/0	0/0	0/0	0/0	0/0	0/0	0/0	0/0	0/0	0/0	0/0	0/0
0/0	0/0	0/0	0/0	0/0	0/0	0/0	0/0	0/0	0/0	0/0	0/0	0/0	0/0	0/0	0/0	0/0	0/0	0/0	0/0	0/0	0/0	0/0	0/0	0/0	0/0	0/0
0/0	0/0	0/0	0/0	1/1	0/0	1/1	0/0	0/0	0/0	0/0	0/0	0/0	0/0	0/0	0/1	0/0	1/1	0/0	0/0	0/0	0/0	0/0	0/0	0/0	0/0	0/0
0/0	1/1	0/0	0/0	1/1	0/0	1/1	0/0	0/0	1/1	1/1	0/0	0/0	0/0	0/0	1/1	0/0	1/1	0/0	0/0	0/0	1/1	0/0	0/0	0/0	0/0	0/0
1/1	1/1	1/1	1/1	1/1	1/1	1/1	1/1	1/1	1/1	1/1	1/1	1/1	1/1	1/1	1/1	0/0	1/1	1/1	0/0	0/0	1/1	1/1	0/0	0/0	0/0	0/0
0/0	1/1	0/0	0/0	1/1	0/0	1/1	0/0	0/0	1/1	1/1	0/0	0/0	0/0	0/0	1/1	0/0	1/1	0/0	0/0	0/0	1/1	0/0	0/0	0/0	0/0	0/0
0/0	1/1	0/0	0/0	0/0	0/0	0/0	0/0	0/0	0/0	1/1	0/0	0/0	0/0	0/0	0/0	0/0	0/0	0/0	0/0	0/0	1/1	0/0	0/0	0/0	0/0	0/0
0/0	0/0	0/0	0/0	0/0	0/0	0/0	0/0	0/0	0/0	0/0	0/0	0/0	0/0	0/0	0/0	0/0	0/0	0/0	0/0	0/0	0/0	0/0	0/0	0/0	0/0	0/0
0/0	0/0	0/1	0/0	0/0	0/0	0/0	0/0	0/0	0/0	0/0	0/0	0/0	0/0	0/0	0/0	0/0	0/0	0/0	0/0	0/0	0/0	0/0	0/0	0/0	0/0	0/0
0/0	0/0	0/0	0/0	0/0	0/0	0/0	0/0	0/0	0/0	0/0	0/0	0/0	0/0	0/0	0/0	0/0	0/0	0/0	0/0	0/0	0/0	0/0	0/0	0/0	0/0	0/0
1/1	0/0	1/1	1/1	0/0	1/1	0/0	1/1	1/1	0/0	0/0	1/1	1/1	1/1	1/1	0/0	0/0	0/0	1/1	0/0	0/0	0/0	1/1	0/0	0/0	0/0	0/0
1/1	0/0	0/1	1/1	1/1	1/1	1/1	1/1	1/1	2/2	0/0	1/1	1/1	1/1	1/1	1/1	0/0	1/1	1/1	0/0	0/0	0/0	1/1	0/0	0/0	0/0	0/0
1/1	1/1	1/1	1/1	1/1	1/1	1/1	1/1	1/1	1/1	1/1	1/1	1/1	1/1	1/1	1/1	0/0	1/1	1/1	0/0	0/0	1/1	1/1	0/0	0/0	0/0	0/0
1/1	1/1	1/1	1/1	1/1	1/1	1/1	1/1	1/1	1/1	1/1	1/1	1/1	1/1	1/1	1/1	1/1	1/1	1/1	0/0	0/0	1/1	1/1	0/0	1/1	0/0	1/1
0/0	0/0	0/0	0/0	0/0	0/0	0/0	0/0	0/0	0/0	0/0	0/0	0/0	0/0	0/0	0/0	0/0	0/0	0/0	0/0	0/0	0/0	0/0	0/0	0/0	0/0	0/0
0/0	0/0	0/0	0/0	1/1	0/0	1/1	0/0	0/0	0/0	0/0	0/0	0/0	0/0	0/0	1/1	0/0	1/1	0/0	0/0	0/0	0/0	0/0	0/0	0/0	0/0	0/0
0/0	1/1	0/0	0/0	1/1	0/0	1/1	0/0	0/0	1/1	1/1	0/0	0/0	0/0	0/0	1/1	0/0	1/1	0/0	0/0	0/0	1/1	0/0	0/0	0/0	0/0	0/0
1/1	1/1	1/1	1/1	1/1	1/1	1/1	1/1	1/1	1/1	1/1	1/1	1/1	1/1	1/1	1/1	0/0	1/1	1/1	0/0	0/0	1/1	1/1	0/0	0/0	0/0	0/0
0/0	0/0	0/0	0/0	0/0	0/0	0/0	0/0	0/0	0/0	0/0	0/0	0/0	0/0	0/0	0/0	0/0	0/0	1/1	0/0	0/0	0/0	0/0	0/0	0/0	0/0	0/0
0/0	0/0	0/0	0/0	0/0	0/0	0/0	0/0	0/0	0/0	0/0	0/0	0/0	0/0	0/0	0/0	0/0	0/0	0/0	0/0	0/0	0/0	0/0	0/0	0/0	0/0	0/0
0/0	0/0	0/0	0/0	0/0	0/0	0/0	0/0	0/0	1/1	0/0	0/0	0/0	0/0	0/0	0/0	0/0	0/0	0/0	0/0	0/0	0/0	0/0	0/0	0/0	0/0	0/0
1/1	1/1	1/1	1/1	1/1	1/1	1/1	1/1	1/1	1/1	1/1	1/1	1/1	1/1	1/1	1/1	0/0	1/1	1/1	0/0	0/0	1/1	1/1	0/0	0/0	0/0	0/0
0/0	0/0	0/0	0/0	0/0	0/0	0/0	0/0	0/0	0/0	0/0	0/0	0/0	0/0	0/0	0/0	0/0	0/0	0/0	0/0	0/0	0/0	0/0	0/0	0/0	0/0	0/0
0/0	0/0	0/0	0/0	0/0	0/0	0/0	0/0	0/0	1/1	0/0	0/0	0/0	0/0	0/0	0/0	0/0	0/0	0/0	0/0	0/0	0/0	0/0	0/0	0/0	0/0	0/0
0/0	0/0	0/0	0/0	0/0	0/0	0/0	0/0	0/0	0/0	0/0	0/0	0/0	0/0	0/0	0/0	0/0	0/0	0/0	0/0	0/0	0/0	0/0	0/0	0/0	0/0	0/0
0/0	0/0	0/0	0/0	0/0	0/0	0/0	0/0	0/0	0/0	0/0	0/0	0/0	0/0	0/0	0/0	0/0	0/0	0/0	0/0	0/0	0/0	0/0	0/0	0/0	0/0	0/0
0/0	0/0	0/0	0/0	0/0	0/0	0/0	0/0	0/0	0/0	0/0	0/0	0/0	0/0	0/0	0/0	0/0	0/0	0/0	0/0	0/0	0/0	0/0	0/0	0/0	0/0	0/0
0/0	0/0	0/0	0/0	0/0	0/0	0/0	0/0	0/0	0/0	0/0	0/0	0/0	0/0	0/0	0/0	0/0	0/0	0/0	0/0	0/0	0/0	0/0	0/0	0/0	0/0	0/0
0/0	0/0	0/0	0/0	0/0	0/0	0/0	0/0	0/0	0/0	0/0	0/0	0/0	0/0	0/0	0/0	0/0	0/0	0/0	0/0	0/0	0/0	0/0	0/0	0/0	0/0	0/0
0/0	0/0	0/0	0/0	0/0	0/0	0/0	0/0	0/0	0/0	0/0	0/0	0/0	0/0	0/0	0/0	0/0	0/0	0/0	0/0	0/0	0/0	0/0	0/0	0/0	0/0	0/0
1/1	0/0	1/1	1/1	0/0	1/1	0/0	1/1	1/1	0/0	0/0	1/1	1/1	1/1	1/1	0/0	0/0	0/0	1/1	0/0	0/0	0/0	1/1	0/0	0/0	0/0	0/0
0/0	0/0	0/0	0/0	0/0	0/0	0/0	0/0	0/0	0/0	0/0	0/0	0/0	0/0	0/0	0/0	0/0	0/0	0/0	0/0	0/0	0/0	0/0	0/0	0/0	0/0	0/0
0/0	0/0	0/0	0/0	0/0	0/0	0/0	0/0	0/0	0/0	0/0	0/0	0/0	0/0	0/0	0/0	0/0	0/0	0/0	0/0	0/0	0/0	0/0	0/0	0/0	0/0	0/0
0/0	0/0	0/0	0/0	1/1	0/0	1/1	0/0	0/0	0/0	0/0	0/0	0/0	0/0	0/0	0/0	0/0	1/1	0/0	0/0	0/0	0/0	0/0	0/0	0/0	0/0	0/0

0/0	1/1	0/0	0/0	0/0	0/0	0/0	0/0	0/0	0/0	1/1	0/0	0/0	0/0	0/0	0/0	0/0	0/0	0/0	0/0	0/0	1/1	0/0	0/0	0/0	0/0	0/0
1/1	4/4	1/2	1/1	2/2	2/2	2/2	1/1	2/3	1/4	4/4	2/2	2/2	1/1	2/2	1/2	3/3	2/2	1/1	0/0	4/4	4/4	1/4	0/4	4/4	0/0	4/4
./.	./.	./.	./.	./.	./.	./.	./.	./.	./.	./.	./.	./.	./.	./.	./.	./.	./.	./.	0/0	./.	./.	./.	0/0	./.	0/0	./.
0/0	0/0	0/0	0/0	0/0	0/0	0/0	0/0	0/0	0/0	0/0	0/0	0/0	0/0	0/0	0/0	0/0	0/0	0/0	0/0	0/0	0/0	0/0	0/0	0/0	0/0	0/0
0/0	0/0	0/0	0/0	0/0	0/0	0/0	0/0	0/0	0/0	0/0	0/0	0/0	0/0	0/0	0/0	0/0	0/0	0/0	0/0	0/0	0/0	0/0	0/0	0/0	0/0	0/0
0/0	0/0	0/0	0/0	0/0	0/0	0/0	0/0	0/0	1/1	0/0	0/0	0/0	0/0	0/0	0/0	0/0	0/0	0/0	0/0	0/0	0/0	0/0	0/0	0/0	0/0	0/0
0/0	1/1	0/0	0/0	0/0	0/0	0/0	0/0	0/0	0/0	1/1	0/0	0/0	0/0	0/0	0/1	0/0	0/0	0/0	0/0	0/0	1/1	0/0	0/0	0/0	0/0	0/0
0/0	1/1	0/0	0/0	0/0	0/0	0/0	0/0	0/0	0/0	1/1	0/0	0/0	0/0	0/0	0/0	0/0	0/0	0/0	0/0	0/0	1/1	0/0	0/0	0/0	0/0	0/0
0/0	0/0	0/0	0/0	0/0	0/0	0/0	0/0	0/0	1/1	./.	0/0	0/0	./.	0/0	0/0	0/0	0/0	0/0	0/0	0/0	0/0	0/0	0/0	0/0	0/0	0/0
./.	1/1	./.	./.	1/1	./.	1/1	./.	./.	1/1	1/1	./.	0/0	./.	./.	0/0	0/0	1/1	1/1	0/0	0/0	1/1	./.	0/0	0/0	0/0	0/0
2/2	./.	2/2	2/2	./.	2/2	./.	2/2	2/2	./.	./.	2/2	2/2	2/2	2/2	0/0	1/1	./.	./.	0/0	0/0	./.	2/2	0/0	0/0	0/0	0/0
./.	./.	./.	./.	0/0	./.	./.	./.	./.	./.	./.	./.	./.	./.	./.	0/1	2/2	./.	./.	0/0	0/0	./.	./.	0/0	0/0	0/0	0/0
0/0	0/0	0/0	0/0	0/0	0/0	0/0	0/0	0/0	1/1	0/0	0/0	0/0	0/0	0/0	0/0	0/0	0/0	0/0	0/0	0/0	0/0	0/0	0/0	0/0	0/0	0/0
0/0	0/0	0/0	0/0	0/0	0/0	0/0	0/0	0/0	0/0	0/0	0/0	0/0	0/0	0/0	0/0	0/0	0/0	0/0	0/0	0/0	0/0	0/0	0/0	0/0	0/0	0/0
0/0	0/0	0/0	0/0	0/0	0/0	0/0	0/0	0/0	0/0	0/0	0/0	0/0	0/0	0/0	0/0	0/0	0/0	0/0	0/0	0/0	0/0	0/0	0/0	0/0	0/0	0/0
1/1	1/1	1/1	1/1	1/1	1/1	1/1	1/1	1/1	1/1	1/1	1/1	1/1	1/1	1/1	0/1	0/0	1/1	1/1	0/0	0/0	1/1	1/1	0/0	0/0	0/0	0/0
0/0	0/0	0/0	0/0	0/0	0/0	0/0	0/0	0/0	0/0	0/0	0/0	0/0	0/0	0/0	0/0	0/0	0/0	0/0	0/0	0/0	0/0	0/0	0/0	0/0	0/0	0/0
0/0	0/0	0/0	0/0	1/1	0/0	1/1	0/0	0/0	0/0	0/0	0/0	0/0	0/0	0/0	0/1	0/0	1/1	0/0	0/0	0/0	0/0	0/0	0/0	0/0	0/0	0/0
0/0	0/0	0/0	0/0	0/0	0/0	0/0	0/0	0/0	0/0	0/0	0/0	0/0	0/0	0/0	0/0	0/0	0/0	0/0	0/0	0/0	0/0	0/0	0/0	0/0	0/0	0/0
0/0	0/0	0/0	0/0	0/0	0/0	0/0	0/0	0/0	0/0	0/0	0/0	0/0	0/0	0/0	0/0	0/0	0/0	0/0	0/0	0/0	0/0	0/0	0/0	0/0	0/0	0/0
0/0	0/0	0/0	0/0	0/0	0/0	0/0	0/0	0/0	0/0	0/0	0/0	0/0	0/0	0/0	0/0	0/0	0/0	0/0	0/0	0/0	0/0	0/0	0/0	0/0	0/0	0/0

0/0	0/0	0/0	0/0	0/0	0/0	0/0	0/0	0/0	0/0	0/0	0/0	0/0	0/0	0/0	0/0	0/0	0/0	0/0	0/0	0/0	0/0	0/0	0/0	0/0	0/0	0/0
0/0	0/0	0/0	0/0	0/0	0/0	0/0	0/0	0/0	0/0	0/0	0/0	0/0	0/0	0/0	0/0	0/0	0/0	0/0	0/0	0/0	0/0	0/0	0/0	0/0	0/0	0/0
2/2	1/1	2/2	2/2	1/1	2/2	1/1	2/2	2/2	1/1	1/1	2/2	2/2	2/2	2/2	0/1	0/0	1/1	2/2	0/0	0/0	1/1	2/2	0/0	0/0	0/0	0/0
0/0	0/0	0/0	0/0	0/0	0/0	0/0	0/0	0/0	0/0	0/0	0/0	0/0	0/0	0/0	0/0	0/0	0/0	0/0	0/0	0/0	0/0	0/0	0/0	0/0	0/0	0/0
2/2	0/0	2/2	2/2	1/1	2/2	1/1	2/2	2/2	0/0	0/0	2/2	2/2	2/2	2/2	1/1	0/0	1/1	2/2	0/0	0/0	0/0	2/2	0/0	0/0	0/0	0/0
0/0	0/0	0/0	0/0	0/0	0/0	0/0	0/0	0/0	0/0	0/0	0/0	0/0	0/0	0/0	0/0	0/0	0/0	0/0	0/0	0/0	0/0	0/0	0/0	0/0	0/0	0/0
0/0	1/1	0/0	0/0	0/0	0/0	0/0	0/0	0/0	0/0	1/1	0/0	0/0	0/0	0/0	0/0	0/0	0/0	0/0	0/0	0/0	1/1	0/0	0/0	0/0	0/0	0/0
1/1	1/1	1/1	1/1	1/1	1/1	1/1	1/1	1/1	1/1	1/1	1/1	1/1	1/1	1/1	1/1	0/0	1/1	1/1	0/0	0/0	1/1	1/1	0/0	0/0	0/0	0/0
1/1	1/1	1/1	1/1	1/1	1/1	1/1	1/1	1/1	1/1	1/1	1/1	1/1	1/1	1/1	1/1	0/0	1/1	1/1	0/0	0/0	1/1	1/1	0/0	0/0	0/0	0/0
0/0	0/0	0/0	0/0	0/0	0/0	0/0	0/0	0/0	0/0	0/0	0/0	0/0	0/0	0/0	0/0	0/0	0/0	0/0	0/0	0/0	0/0	0/0	0/0	0/0	0/0	0/0
2/2	2/2	2/2	2/2	1/1	2/2	1/1	2/2	2/2	2/2	2/2	2/2	2/2	2/2	2/2	1/1	0/0	1/1	2/2	0/0	0/0	2/2	2/2	0/0	0/0	0/0	0/0
0/0	0/0	0/0	0/0	0/0	0/0	0/0	0/0	0/0	0/0	0/0	0/0	0/0	0/0	0/0	0/0	0/0	0/0	0/0	0/0	0/0	0/0	0/0	0/0	0/0	0/0	0/0
0/0	0/0	0/0	0/0	0/0	0/0	0/0	0/0	0/0	0/0	0/0	0/0	0/0	0/0	0/0	0/0	0/0	0/0	0/0	0/0	0/0	0/0	0/0	0/0	0/0	0/0	0/0
0/0	0/0	0/0	0/0	0/0	0/0	0/0	0/0	0/0	0/0	0/0	0/0	0/0	0/0	0/0	1/1	0/0	0/0	0/0	0/0	0/0	0/0	0/0	0/0	0/0	0/0	0/0
0/0	1/1	0/0	0/0	0/0	0/0	0/0	0/0	0/0	1/1	1/1	0/0	0/0	0/0	0/0	0/0	0/0	0/0	0/0	0/0	0/0	1/1	0/0	0/0	0/0	0/0	0/0
0/0	0/0	0/0	0/0	0/0	0/0	0/0	0/0	0/0	0/0	0/0	0/0	0/0	0/0	0/0	0/0	0/0	0/0	0/0	0/0	0/0	0/0	0/0	0/0	0/0	0/0	0/0
0/0	1/1	0/0	0/0	0/0	0/0	0/0	0/0	0/0	0/0	1/1	0/0	0/0	0/0	0/0	0/0	0/0	0/0	0/0	0/0	0/0	1/1	0/0	0/0	0/0	0/0	0/0
1/1	1/1	1/1	1/1	1/1	1/1	1/1	1/1	1/1	1/1	1/1	1/1	1/1	1/1	1/1	1/1	0/0	1/1	1/1	0/0	0/0	1/1	1/1	0/0	0/0	0/0	0/0

823	831	837	844	846	852	854	861	871	874	876	87	883	896	897	902	908	912	918	919	922	932	937	940	941	988
0/0	0/0	0/0	0/0	0/0	0/0	0/0	0/0	0/0	0/0	0/0	0/0	0/0	0/0	0/0	0/0	0/0	./.	0/0	0/0	0/0	0/0	0/0	0/0	0/0	0/0
0/0	0/0	0/0	0/0	0/0	0/0	0/0	1/1	0/0	0/0	0/0	0/0	0/0	0/0	0/0	0/0	1/1	./.	1/1	0/0	0/0	0/0	0/0	0/0	0/0	0/0
0/0	0/0	0/0	0/0	0/0	0/0	0/0	0/0	0/0	0/0	0/0	0/0	0/0	0/0	0/0	0/0	0/0	./.	0/0	0/0	0/0	0/0	0/0	0/0	0/0	0/0
0/0	0/0	0/0	0/0	0/0	0/0	0/0	0/0	0/0	0/0	0/0	0/0	0/0	0/0	0/0	0/0	0/0	./.	0/0	0/0	0/0	0/0	0/0	0/0	0/0	0/0
0/0	0/0	0/0	0/0	0/0	0/0	0/0	0/0	0/0	0/0	0/0	1/1	0/0	1/1	0/0	0/1	0/0	./.	0/0	0/0	0/0	1/1	0/0	1/1	1/1	0/0
0/0	0/0	0/0	0/0	0/0	0/0	0/0	0/0	0/0	0/0	0/0	1/1	0/0	1/1	0/0	0/1	0/0	./.	0/0	0/0	0/0	1/1	0/0	1/1	1/1	0/0
0/0	0/0	0/0	0/0	0/0	0/0	0/0	0/0	0/0	0/0	0/0	0/0	0/0	0/0	0/0	0/0	0/0	./.	0/0	0/0	0/0	0/0	0/0	0/0	0/0	0/0
0/0	1/1	0/0	1/1	0/0	1/1	0/0	1/1	0/0	0/0	0/0	0/0	0/0	0/0	0/0	0/1	1/1	./.	1/1	0/0	0/0	0/0	0/0	0/0	0/0	0/0
0/0	0/0	0/0	0/0	0/0	0/0	0/0	0/0	0/0	0/0	0/0	0/0	0/0	0/0	0/0	0/0	0/0	./.	0/0	0/0	0/0	0/0	0/0	0/0	0/0	0/0
0/0	0/0	0/0	0/0	0/0	0/0	0/0	0/0	0/0	0/0	0/0	0/0	0/0	0/0	0/0	0/0	0/0	./.	0/0	0/0	0/0	0/0	0/0	0/0	0/0	0/0
0/0	0/0	0/0	0/0	0/0	0/0	0/0	0/0	0/0	0/0	0/0	0/0	0/0	0/0	0/0	0/0	0/0	./.	0/0	0/0	0/0	0/0	0/0	0/0	0/0	0/0
0/0	0/0	0/0	0/0	0/0	0/0	0/0	0/0	0/0	0/0	0/0	0/0	0/0	0/0	0/0	0/0	0/0	./.	0/0	0/0	0/0	0/0	0/0	0/0	0/0	0/0
0/0	0/0	0/0	0/0	0/0	0/0	0/0	0/0	0/0	0/0	0/0	1/1	0/0	1/1	0/0	0/1	0/0	./.	0/0	0/0	0/0	1/1	0/0	1/1	1/1	0/0
0/0	0/0	0/0	0/0	0/0	0/0	0/0	0/0	0/0	0/0	0/0	0/0	0/0	0/0	0/0	0/1	./.	./.	0/0	0/0	0/0	0/0	0/0	./.	0/0	0/0
0/0	1/1	0/0	1/1	0/0	1/1	0/0	1/1	0/0	0/0	0/0	0/0	0/0	0/0	0/0	0/1	1/1	./.	0/0	0/0	0/0	./.	0/0	./.	0/0	0/0
0/0	0/0	0/0	0/0	0/0	0/0	0/0	0/0	0/0	0/0	0/0	0/0	0/0	0/0	0/0	0/0	0/0	./.	0/0	0/0	0/0	./.	0/0	./.	0/0	0/0

0/1	0/0	3/3	0/0	0/0	0/0	0/0	3/3	0/4	1/1	0/0	1/1	4/4	3/6	0/0	3/5	3/6	./.	0/0	1/1	0/0	./.	1/1	./.	1/1	4/4
0/0	0/0	0/0	0/0	0/0	0/0	0/0	0/0	0/0	0/0	1/1	0/0	0/0	0/0	0/0	0/0	0/0	./.	0/0	0/0	1/1	0/0	0/0	0/0	./.	0/0
0/0	0/0	0/0	0/0	0/0	0/0	0/0	0/0	0/0	0/0	0/0	0/0	0/0	0/0	0/0	0/0	0/0	./.	0/0	0/0	0/0	0/0	0/0	./.	./.	0/0
0/0	0/0	0/0	0/0	0/0	0/0	0/0	0/0	0/0	0/0	0/0	1/1	0/0	1/1	0/0	0/1	0/0	./.	0/0	0/0	0/0	1/1	0/0	1/1	1/1	0/0
0/0	1/1	0/0	1/1	0/0	1/1	0/0	1/1	0/0	0/0	0/0	0/0	0/0	0/0	0/0	0/1	1/1	./.	0/0	0/0	0/0	0/0	0/0	0/0	0/0	0/0
0/0	0/0	0/0	0/0	0/0	0/0	0/0	0/0	0/0	0/0	0/0	0/0	0/0	0/0	0/0	0/0	0/0	./.	0/0	0/0	0/0	0/0	0/0	0/0	0/0	0/0
0/0	1/1	0/0	1/1	0/0	1/1	0/0	1/1	0/0	0/0	0/0	0/0	0/0	0/0	0/0	0/1	1/1	./.	0/0	0/0	0/0	0/0	0/0	0/0	0/0	0/0
0/0	0/0	0/0	0/0	0/0	0/0	0/0	0/0	0/0	0/0	0/0	1/1	0/0	1/1	0/0	0/1	0/0	./.	0/0	0/0	0/0	1/1	0/0	1/1	1/1	0/0
0/0	0/0	1/1	0/0	0/0	0/0	0/0	0/0	0/0	0/0	0/0	0/0	0/0	0/0	0/0	0/0	0/0	./.	0/0	0/0	0/0	0/0	0/0	0/0	0/0	0/0
0/0	1/1	2/2	1/1	0/0	1/1	0/0	0/0	0/0	0/0	0/0	1/1	0/0	1/1	0/0	1/1	0/0	./.	0/0	0/0	0/0	1/1	0/0	1/1	1/1	0/0
0/0	0/0	./.	0/0	0/0	0/0	0/0	0/0	0/0	0/0	0/0	0/0	0/0	0/0	0/0	0/0	0/0	./.	0/0	0/0	0/0	0/0	0/0	0/0	0/0	0/0
0/0	0/0	0/0	0/0	0/0	0/0	0/0	0/0	0/0	0/0	0/0	1/1	0/0	1/1	0/0	0/1	0/0	./.	0/0	0/0	0/0	1/1	0/0	1/1	1/1	0/0
0/0	1/1	0/0	1/1	0/0	1/1	0/0	1/1	0/0	0/0	0/0	1/1	0/0	1/1	0/0	1/1	1/1	./.	0/0	0/0	0/0	1/1	0/0	1/1	1/1	0/0
0/0	0/0	0/0	0/0	0/0	0/0	0/0	0/0	0/0	0/0	0/0	0/0	0/0	0/0	0/0	0/0	0/0	./.	0/0	0/0	0/0	0/0	0/0	0/0	0/0	0/0
0/0	0/0	0/0	0/0	0/0	0/0	0/0	0/0	0/0	0/0	0/0	0/0	0/0	0/0	0/0	0/0	0/0	./.	0/0	0/0	0/0	0/0	0/0	0/0	0/0	0/0
0/0	1/1	0/0	1/1	0/0	1/1	0/0	1/1	0/0	0/0	0/0	1/1	0/0	1/1	0/0	1/1	1/1	./.	0/0	0/0	0/0	1/1	0/0	1/1	1/1	0/0
0/0	0/0	0/0	0/0	0/0	0/0	0/0	0/0	0/0	0/0	0/0	1/1	0/0	1/1	0/0	0/1	0/0	./.	0/0	0/0	0/0	1/1	0/0	1/1	1/1	0/0
0/0	0/0	0/0	0/0	0/0	0/0	0/0	0/0	0/0	0/0	0/0	0/0	0/0	0/0	0/0	0/0	0/0	./.	0/0	0/0	0/0	0/0	0/0	0/0	0/0	0/0
0/0	1/1	0/0	1/1	0/0	1/1	0/0	1/1	0/0	0/0	0/0	0/0	0/0	0/0	0/0	0/1	1/1	./.	0/0	0/0	0/0	0/0	0/0	0/0	0/0	0/0
0/0	0/0	0/0	0/0	0/0	0/0	0/0	1/1	0/0	0/0	0/0	0/0	0/0	0/0	0/0	0/0	1/1	./.	0/0	0/0	0/0	0/0	0/0	0/0	0/0	0/0
0/0	0/0	0/0	0/0	0/0	0/0	0/0	0/0	0/0	0/0	0/0	0/0	0/0	0/0	0/0	0/0	0/0	./.	0/0	0/0	0/0	0/0	0/0	0/0	0/0	0/0
0/0	1/1	0/0	1/1	0/0	1/1	0/0	1/1	0/0	0/0	0/0	0/0	0/0	0/0	0/0	0/1	1/1	./.	0/0	0/0	0/0	0/0	0/0	0/0	0/0	0/0
0/0	0/0	0/0	0/0	0/0	0/0	0/0	0/0	0/0	0/0	0/0	0/0	0/0	0/0	0/0	0/0	0/0	./.	0/0	0/0	0/0	0/0	0/0	0/0	0/0	0/0
0/0	1/1	0/0	1/1	0/0	1/1	0/0	1/1	0/0	0/0	0/0	1/1	0/0	1/1	0/0	1/1	1/1	./.	0/0	0/0	0/0	1/1	0/0	1/1	1/1	0/0
0/0	0/0	0/0	0/0	0/0	0/0	0/0	0/0	0/0	0/0	0/0	1/1	0/0	1/1	0/0	0/1	0/0	./.	0/0	0/0	0/0	1/1	0/0	1/1	1/1	0/0
0/0	0/0	0/0	0/0	0/0	0/0	0/0	1/1	0/0	0/0	0/0	0/0	0/0	0/0	0/0	0/0	1/1	./.	0/0	0/0	0/0	0/0	0/0	0/0	0/0	0/0

0/0	1/1	1/1	1/1	0/0	1/1	0/0	1/1	0/0	0/0	0/0	1/1	1/1	1/1	1/1	1/1	1/1	./.	1/1	0/0	0/0	1/1	0/0	1/1	1/1	1/1
0/0	0/0	0/0	0/0	0/0	0/0	0/0	0/0	0/0	0/0	0/0	1/1	0/0	1/1	0/0	0/1	0/0	./.	0/0	0/0	0/0	1/1	0/0	1/1	1/1	0/0
0/0	0/0	0/0	0/0	0/0	0/0	0/0	0/0	0/0	0/0	0/0	1/1	0/0	1/1	0/0	0/1	0/0	./.	0/0	0/0	0/0	1/1	0/0	1/1	1/1	0/0
0/0	0/0	0/0	0/0	0/0	0/0	0/0	0/0	0/0	0/0	0/0	0/0	0/0	0/0	0/0	0/0	0/0	./.	0/0	0/0	0/0	0/0	0/0	0/0	0/0	0/0
0/0	1/1	0/0	1/1	0/0	1/1	0/0	1/1	0/0	0/0	0/0	1/1	0/0	1/1	0/0	1/1	1/1	./.	0/0	0/0	0/0	1/1	0/0	1/1	1/1	0/0
0/0	1/1	0/0	1/1	0/0	1/1	0/0	1/1	0/0	0/0	0/0	1/1	0/0	1/1	0/0	1/1	1/1	./.	0/0	0/0	0/0	1/1	0/0	1/1	1/1	0/0
0/0	1/1	0/0	1/1	0/0	1/1	0/0	1/1	0/0	0/0	0/0	1/1	0/0	1/1	0/0	1/1	1/1	./.	0/0	0/0	0/0	1/1	0/1	1/1	1/1	0/0
0/0	0/0	0/0	0/0	0/0	0/0	0/0	0/0	0/0	0/0	0/0	0/0	0/0	0/0	0/0	0/0	0/0	./.	0/0	0/0	0/0	0/0	0/0	0/0	0/0	0/0
0/0	0/0	0/0	0/0	0/0	0/0	0/0	0/0	0/0	0/0	0/0	1/1	0/0	1/1	0/0	0/1	0/0	./.	0/0	0/0	0/0	1/1	0/0	1/1	1/1	0/0
0/0	0/0	0/0	0/0	0/0	0/0	0/0	0/0	0/0	0/0	1/1	0/0	0/0	0/0	0/0	0/0	0/0	./.	0/0	0/0	1/1	0/0	0/0	0/0	0/0	0/0
0/0	1/1	0/0	1/1	0/0	1/1	0/0	1/1	0/0	0/0	0/0	0/0	0/0	0/0	0/0	0/1	1/1	./.	0/0	0/0	0/0	0/0	0/0	0/0	0/0	0/0
0/0	0/0	0/0	0/0	0/0	0/0	0/0	0/0	0/0	0/0	0/0	0/0	0/0	0/0	0/0	0/0	0/0	./.	0/0	0/0	0/0	0/0	0/0	0/0	0/0	0/0
0/0	0/0	0/0	0/0	0/0	0/0	0/0	0/0	0/0	0/0	0/0	0/0	0/0	0/0	0/0	0/0	0/0	./.	0/0	0/0	0/0	0/0	0/0	0/0	0/0	0/0
0/0	1/1	0/0	1/1	0/0	1/1	0/0	0/0	0/0	0/0	0/0	0/0	0/0	0/0	0/0	0/1	0/0	./.	0/0	0/0	0/0	0/0	0/0	0/0	0/0	0/0
0/0	1/1	0/0	1/1	0/0	1/1	0/0	1/1	0/0	0/0	0/0	1/1	0/0	1/1	0/0	1/1	1/1	./.	0/0	0/0	0/0	1/1	0/0	1/1	1/1	0/0
0/0	0/0	0/0	0/0	0/0	0/0	0/0	0/0	0/0	0/0	0/0	0/0	0/0	0/0	0/0	0/0	0/0	./.	0/0	0/0	0/0	0/0	0/0	0/0	0/0	0/0
0/0	0/0	0/0	0/0	0/0	0/0	0/0	0/0	0/0	0/0	1/1	0/0	0/0	0/0	0/0	0/0	0/0	./.	0/0	0/0	0/0	0/0	0/0	0/0	0/0	0/0
0/0	0/0	0/0	0/0	0/0	0/0	0/0	0/0	0/0	0/0	0/0	0/0	0/0	0/0	0/0	0/0	0/0	./.	0/0	0/0	0/0	0/0	0/0	0/0	0/0	0/0
0/0	1/1	0/0	1/1	0/0	1/1	0/0	1/1	0/0	0/0	0/0	0/0	0/0	0/0	0/0	0/1	1/1	./.	0/0	0/0	0/0	0/0	0/0	0/0	0/0	0/0
0/0	0/0	0/0	0/0	0/0	0/0	0/0	0/0	0/0	0/0	0/0	0/0	0/0	0/0	0/0	0/0	0/0	./.	0/0	0/0	0/0	0/0	0/0	0/0	0/0	0/0
0/0	1/1	0/0	1/1	0/0	1/1	0/0	1/1	0/0	0/0	0/0	1/1	0/0	1/1	0/0	1/1	1/1	./.	0/0	0/0	0/0	1/1	0/0	1/1	1/1	0/0
0/0	1/1	0/0	1/1	0/0	1/1	0/0	1/1	0/0	0/0	0/0	0/0	0/0	0/0	0/0	0/1	1/1	./.	0/0	0/0	0/0	0/0	0/0	0/0	0/0	0/0
0/0	1/1	0/0	1/1	0/0	1/1	0/0	1/1	0/0	0/0	0/0	0/0	0/0	0/0	0/0	0/1	1/1	./.	0/0	0/0	0/0	0/0	0/0	0/0	0/0	0/0
0/0	0/0	0/0	0/0	0/0	0/0	0/0	0/0	0/0	0/0	0/0	1/1	0/0	1/1	0/0	0/1	0/0	./.	0/0	0/0	0/0	1/1	0/0	1/1	1/1	0/0
0/0	1/1	0/0	1/1	0/0	1/1	0/0	1/1	0/0	0/0	0/0	1/1	0/0	1/1	0/0	1/1	1/1	./.	0/0	0/0	0/0	1/1	0/0	1/1	1/1	0/0
0/0	0/0	0/0	0/0	0/0	0/0	0/0	0/0	0/0	0/0	0/0	1/1	0/0	1/1	0/0	0/1	0/0	./.	0/0	0/0	0/0	1/1	0/0	1/1	1/1	0/0
0/0	0/0	0/0	0/0	0/0	0/0	0/0	0/0	0/0	0/0	0/0	0/0	0/0	0/0	0/0	0/0	0/0	./.	0/0	0/0	0/0	0/0	0/0	0/0	0/0	0/0
0/0	0/0	0/0	0/0	0/0	0/0	0/0	0/0	0/0	0/0	0/0	0/0	0/0	0/0	0/0	0/0	0/0	./.	0/0	0/0	0/0	0/0	0/0	0/0	0/0	0/0
0/0	1/1	1/1	1/1	0/0	1/1	0/0	1/1	0/0	0/0	0/0	1/1	1/1	1/1	1/1	1/1	1/1	./.	1/1	0/0	0/0	1/1	0/0	1/1	1/1	1/1
0/0	0/0	0/0	0/0	0/0	0/0	0/0	0/0	0/0	0/0	0/0	0/0	0/0	0/0	0/0	0/0	0/0	./.	0/0	1/1	0/0	0/0	0/0	0/0	0/0	0/0
0/0	1/1	0/0	1/1	0/0	1/1	0/0	1/1	0/0	0/0	0/0	1/1	0/0	1/1	0/0	1/1	1/1	./.	0/0	0/0	0/0	1/1	0/1	1/1	1/1	0/0
0/0	0/0	0/0	0/0	0/0	0/0	0/0	0/0	0/0	0/0	0/0	1/1	0/0	1/1	0/0	0/1	0/0	./.	0/0	0/0	0/0	1/1	0/1	1/1	1/1	0/0
0/0	1/1	0/0	1/1	0/0	1/1	0/0	1/1	0/0	0/0	0/0	1/1	0/0	1/1	0/0	1/1	1/1	./.	0/0	0/0	0/0	1/1	0/1	1/1	1/1	0/0
0/0	0/0	0/0	0/0	0/0	0/0	0/0	0/0	0/0	0/0	0/0	0/0	0/0	0/0	0/0	0/0	0/0	./.	0/0	0/0	0/0	0/0	0/0	0/0	0/0	0/0
0/0	0/0	0/0	0/0	0/0	0/0	0/0	0/0	0/0	0/0	0/0	1/1	0/0	1/1	0/0	0/1	0/0	./.	0/0	0/0	0/0	1/1	0/1	1/1	1/1	0/0

0/0	1/1	0/0	1/1	0/0	1/1	0/0	1/1	0/0	0/0	0/0	0/0	0/0	0/0	0/0	0/1	1/1	./.	0/0	0/0	0/0	0/0	0/0	0/0	0/0	0/0
0/0	0/0	0/0	0/0	0/0	0/0	0/0	0/0	0/0	0/0	0/0	0/0	0/0	0/0	0/0	0/0	0/0	./.	0/0	0/0	0/0	0/0	0/0	0/0	0/0	0/0
0/0	0/0	0/0	0/0	0/0	0/0	0/0	1/1	0/0	0/0	0/0	0/0	0/0	0/0	0/0	0/0	1/1	./.	0/0	0/0	0/0	0/0	0/0	0/0	0/0	0/0
0/0	1/1	0/0	1/1	0/0	1/1	0/0	1/1	0/0	0/0	0/0	1/1	0/0	1/1	0/0	1/1	1/1	./.	0/0	0/0	0/0	1/1	0/0	1/1	1/1	0/0
0/0	0/0	0/0	0/0	0/0	0/0	0/0	0/0	0/0	0/0	0/0	0/0	0/0	0/0	0/0	0/0	0/0	./.	0/0	0/0	0/0	0/0	0/0	0/0	0/0	0/0
0/0	1/1	0/0	1/1	0/0	1/1	0/0	1/1	0/0	0/0	0/0	0/0	0/0	0/0	0/0	0/1	1/1	./.	0/0	0/0	0/0	0/0	0/0	0/0	0/0	0/0
0/0	1/1	0/0	1/1	0/0	1/1	0/0	1/1	0/0	0/0	0/0	1/1	0/0	1/1	0/0	1/1	1/1	./.	0/0	0/0	0/0	1/1	0/0	1/1	1/1	0/0
0/0	0/0	0/0	0/0	0/0	0/0	0/0	0/0	0/0	0/0	0/0	1/1	0/0	1/1	0/0	0/1	0/0	./.	0/0	0/0	0/0	1/1	0/0	1/1	1/1	0/0
0/0	1/1	0/0	1/1	0/0	1/1	0/0	1/1	0/0	0/0	0/0	0/0	0/0	0/0	0/0	0/1	1/1	./.	0/0	0/0	0/0	0/0	0/0	0/0	0/0	0/0
0/0	0/0	0/0	0/0	0/0	0/0	0/0	0/0	0/0	0/0	0/0	0/0	0/0	0/0	0/0	0/0	0/0	./.	0/0	0/0	0/0	0/0	0/0	0/0	0/0	0/0
0/0	1/1	0/0	1/1	0/0	1/1	0/0	1/1	0/0	0/0	0/0	0/0	0/0	0/0	0/0	0/1	1/1	./.	0/0	0/0	0/0	0/0	0/0	0/0	0/0	0/0
0/0	0/0	0/0	0/0	0/0	0/0	0/0	0/0	0/0	0/0	0/0	0/0	0/0	0/0	0/0	0/0	0/0	./.	0/0	0/0	0/0	0/0	0/0	0/0	0/0	0/0
0/0	0/0	0/0	0/0	0/0	0/0	0/0	0/0	0/0	0/0	0/0	0/0	0/0	0/0	0/0	0/0	0/0	./.	0/0	0/0	0/0	0/0	0/0	0/0	0/0	0/0
0/0	0/0	0/0	0/0	0/0	0/0	0/0	0/0	0/0	0/0	0/0	0/0	0/0	0/0	0/0	0/0	0/0	./.	0/0	0/0	0/0	0/0	0/0	0/0	0/0	0/0
0/0	1/1	1/1	1/1	0/0	1/1	0/0	1/1	0/0	0/0	0/0	1/1	1/1	1/1	1/1	1/1	1/1	./.	1/1	0/0	0/0	1/1	0/0	1/1	1/1	1/1
0/0	1/1	1/1	1/1	0/0	1/1	0/0	1/1	0/0	0/0	0/0	1/1	1/1	1/1	1/1	1/1	1/1	./.	1/1	0/0	0/0	1/1	0/0	1/1	1/1	1/1
0/0	1/1	0/0	1/1	0/0	1/1	0/0	1/1	0/0	0/0	0/0	1/1	0/0	1/1	0/0	1/1	1/1	./.	0/0	0/0	0/0	1/1	0/0	1/1	1/1	0/0
0/0	./.	0/0	./.	0/0	./.	0/0	1/1	0/0	0/0	0/0	./.	./.	./.	0/0	./.	./.	./.	./.	0/0	0/0	0/0	0/0	0/0	./.	./.
0/0	1/1	1/1	1/1	0/0	1/1	0/0	4/4	0/0	0/0	2/2	3/3	1/1	3/3	1/1	1/3	1/1	./.	1/1	0/0	2/2	3/3	0/0	3/3	3/3	1/1
0/0	0/0	0/0	0/0	0/0	0/0	0/0	0/0	0/0	0/0	0/0	0/0	0/0	0/0	0/0	0/0	0/0	./.	0/0	0/0	0/0	0/0	0/0	0/0	0/0	0/0
0/0	0/0	0/0	0/0	0/0	0/0	0/0	0/0	0/0	0/0	0/0	0/0	0/0	0/0	0/0	0/0	0/0	./.	0/0	0/0	0/0	0/0	0/0	0/0	0/0	0/0
0/0	0/0	0/0	0/0	0/0	0/0	0/0	0/0	0/0	0/0	0/0	0/0	0/0	0/0	0/0	0/0	0/0	./.	0/0	0/0	0/0	0/0	0/0	0/0	0/0	0/0
0/0	0/0	0/0	0/0	0/0	0/0	0/0	0/0	0/0	0/0	0/0	0/0	0/0	0/0	0/0	0/0	0/0	./.	0/0	0/0	0/0	0/0	0/0	1/1	0/0	0/0
0/0	0/0	0/0	0/0	0/0	0/0	0/0	0/0	0/0	0/0	0/0	0/0	0/0	./.	0/0	0/0	0/0	./.	0/0	0/0	0/0	0/0	0/0	0/0	0/0	0/0
0/0	0/0	0/0	0/0	0/0	0/0	0/0	0/0	0/0	0/0	0/0	1/1	0/0	1/1	0/0	0/1	0/0	./.	0/0	0/0	0/0	1/1	0/0	1/1	1/1	0/0

0/0	0/0	0/0	0/0	0/0	0/0	0/0	0/0	0/0	0/0	0/0	0/0	0/0	0/0	0/0	0/0	0/0	./.	0/0	0/0	0/0	0/0	0/0	0/0	0/0	0/0
0/0	0/0	0/0	0/0	0/0	0/0	0/0	0/0	0/0	0/0	0/0	1/1	0/0	1/1	0/0	0/1	0/0	./.	0/0	0/0	0/0	1/1	0/0	1/1	1/1	0/0
0/0	0/0	0/0	0/0	0/0	0/0	0/0	0/0	0/0	0/0	0/0	1/1	0/0	1/1	0/0	0/1	0/0	./.	0/0	0/0	0/0	1/1	0/0	1/1	1/1	0/0
0/0	1/1	1/1	1/1	0/0	1/1	0/0	1/1	0/0	0/0	0/0	1/1	1/1	1/1	1/1	1/1	1/1	./.	1/1	0/0	0/0	1/1	0/0	1/1	1/1	1/1
0/0	1/1	0/0	1/1	0/0	1/1	0/0	1/1	0/0	0/0	0/0	1/1	0/0	1/1	0/0	1/1	1/1	./.	0/0	0/0	0/0	1/1	0/0	1/1	1/1	0/0
0/0	0/0	0/0	0/0	0/0	0/0	0/0	0/0	0/0	0/0	0/0	1/1	0/0	0/0	0/0	0/0	0/0	./.	0/0	0/0	0/0	0/0	0/0	0/0	0/0	0/0
0/0	1/1	0/0	1/1	0/0	1/1	0/0	1/1	0/0	0/0	0/0	1/1	0/0	1/1	0/0	1/1	1/1	./.	0/0	0/0	0/0	1/1	0/0	1/1	1/1	0/0
0/0	0/0	0/0	0/0	0/0	0/0	0/0	0/0	0/0	0/0	0/0	1/1	0/0	1/1	0/0	0/1	0/0	./.	0/0	0/0	0/0	1/1	0/0	1/1	1/1	0/0
0/0	0/0	0/0	0/0	0/0	0/0	0/0	0/0	0/0	0/0	0/0	0/0	0/0	0/0	0/0	0/0	0/0	./.	0/0	0/0	0/0	0/0	0/0	0/0	0/0	0/0
0/0	0/0	0/0	0/0	0/0	0/0	0/0	0/0	0/0	0/0	0/0	0/0	0/0	0/0	0/0	0/0	0/0	./.	0/0	0/0	0/0	0/0	0/0	0/0	0/0	0/0
0/0	0/0	0/0	0/0	0/0	0/0	0/0	0/0	0/0	0/0	0/0	1/1	0/0	1/1	0/0	0/1	0/0	./.	0/0	0/0	0/0	1/1	0/0	1/1	1/1	0/0
0/0	0/0	0/0	0/0	0/0	0/0	0/0	0/0	0/0	0/0	0/0	0/0	0/0	0/0	0/0	0/0	0/0	./.	0/0	0/0	0/0	0/0	0/0	0/0	0/0	0/0
0/0	1/1	0/0	1/1	0/0	1/1	0/0	1/1	0/0	0/0	0/0	0/0	0/0	0/0	0/0	0/1	1/1	./.	0/0	0/0	0/0	0/0	0/0	0/0	0/0	0/0
0/0	0/0	0/0	0/0	0/0	0/0	0/0	0/0	0/0	0/0	0/0	1/1	0/0	1/1	0/0	0/1	0/0	./.	0/0	0/0	0/0	1/1	0/0	1/1	1/1	0/0
0/0	0/0	0/0	0/0	0/0	0/0	0/0	0/0	0/0	0/0	0/0	0/0	0/0	0/0	0/0	0/0	0/0	./.	0/0	0/0	0/0	0/0	0/0	0/0	0/0	0/0
0/0	1/1	0/0	1/1	0/0	1/1	0/0	1/1	0/0	0/0	0/0	1/1	0/0	1/1	0/0	1/1	1/1	./.	0/0	0/0	0/0	1/1	0/0	1/1	1/1	0/0
0/0	1/1	0/0	1/1	0/0	1/1	0/0	1/1	0/0	0/0	0/0	0/0	0/0	0/0	0/0	0/1	1/1	./.	0/0	0/0	0/0	0/0	0/0	0/0	0/0	0/0
0/0	1/1	0/0	1/1	0/0	1/1	0/0	0/0	0/0	0/0	0/0	0/0	0/0	0/0	0/0	0/1	0/0	./.	0/0	0/0	0/0	0/0	0/0	0/0	0/0	0/0
0/0	0/0	0/0	0/0	0/0	0/0	0/0	0/0	0/0	0/0	0/0	0/0	0/0	0/0	0/0	0/0	0/0	./.	0/0	0/0	0/0	0/0	0/0	0/0	0/0	0/0
0/0	0/0	0/0	0/0	0/0	0/0	0/0	0/0	0/0	0/0	0/0	1/1	0/0	1/1	0/0	0/1	0/0	./.	0/0	0/0	0/0	1/1	0/0	1/1	1/1	0/0
0/0	0/0	0/0	0/0	0/0	0/0	0/0	0/0	0/0	0/0	0/0	1/1	0/0	1/1	0/0	0/1	0/0	./.	0/0	0/0	0/0	1/1	0/0	1/1	1/1	0/0
0/0	0/0	0/0	0/0	0/0	0/0	0/0	0/0	0/0	0/0	0/0	0/0	0/0	0/0	0/0	0/0	0/0	./.	0/0	0/0	0/0	0/0	0/0	0/0	0/0	0/0
0/0	1/1	0/0	1/1	0/0	1/1	0/0	1/1	0/0	0/0	0/0	1/1	0/0	1/1	0/0	1/1	1/1	./.	0/0	0/0	0/0	1/1	0/0	1/1	1/1	0/0
0/0	0/0	0/0	0/0	0/0	0/0	0/0	0/0	0/0	0/0	0/0	0/0	0/0	0/0	0/0	0/0	0/0	./.	0/0	0/0	0/0	0/0	0/0	0/0	0/0	0/0
0/0	0/0	0/0	0/0	0/0	0/0	0/0	0/0	0/0	0/0	0/0	0/0	0/0	0/0	0/0	0/0	0/0	./.	0/0	0/0	0/0	0/0	0/0	0/0	0/0	0/0
0/0	0/0	0/0	0/0	0/0	0/0	0/0	0/0	0/0	0/0	0/0	1/1	0/0	1/1	0/0	0/1	0/0	./.	0/0	0/0	0/0	1/1	0/0	1/1	1/1	0/0
0/0	0/0	0/0	0/0	0/0	0/0	0/0	0/0	0/0	0/0	0/0	0/0	0/0	0/0	0/0	0/0	0/0	./.	0/0	0/0	0/0	0/0	0/0	0/0	0/0	0/0
0/0	1/1	0/0	1/1	0/0	1/1	0/0	1/1	0/0	0/0	0/0	0/0	0/0	0/0	0/0	0/1	1/1	./.	0/0	0/0	0/0	0/0	0/0	0/0	0/0	0/0
0/0	0/0	0/0	0/0	0/0	0/0	0/0	0/0	0/0	0/0	0/0	1/1	0/0	1/1	0/0	0/1	0/0	./.	0/0	0/0	0/0	1/1	0/0	1/1	1/1	0/0
0/0	0/0	0/0	0/0	0/0	0/0	0/0	0/0	0/0	0/0	0/0	0/0	0/0	0/0	0/0	0/0	0/0	./.	0/0	0/0	0/0	0/0	0/0	0/0	0/0	0/0
0/0	1/1	0/0	1/1	0/0	1/1	0/0	1/1	0/0	0/0	0/0	1/1	0/0	1/1	0/0	1/1	1/1	./.	0/0	0/0	0/0	1/1	0/0	1/1	1/1	0/0
0/0	0/0	0/0	0/0	0/0	0/0	0/0	0/0	0/0	0/0	0/0	0/0	0/0	0/0	0/0	0/0	0/0	./.	0/0	0/0	0/0	0/0	0/0	0/0	0/0	0/0
0/0	0/0	1/1	0/0	0/0	0/0	0/0	0/0	0/0	0/0	0/0	0/0	0/0	0/0	0/0	0/0	0/0	./.	0/0	0/0	0/0	0/0	0/0	0/0	0/0	1/1

0/0	1/1	0/0	1/1	0/0	1/1	0/0	1/1	0/0	0/0	0/0	1/1	0/0	1/1	0/0	1/1	1/1	./.	0/0	0/0	0/0	1/1	0/0	1/1	1/1	0/0
0/0	0/0	0/0	0/0	0/0	0/0	0/0	0/0	0/0	0/0	0/0	0/0	0/0	0/0	0/0	0/0	0/0	./.	0/0	0/0	0/0	0/0	0/0	0/0	0/0	0/0
0/0	1/1	0/0	1/1	0/0	1/1	0/0	1/1	0/0	0/0	0/0	1/1	0/0	1/1	0/0	1/1	1/1	./.	0/0	0/0	0/0	1/1	0/0	1/1	1/1	0/0
0/0	0/0	0/0	0/0	0/0	0/0	0/0	0/0	0/0	0/0	0/0	0/0	0/0	0/0	0/0	0/0	0/0	./.	0/0	0/0	0/0	0/0	0/0	0/0	0/0	0/0
0/0	0/0	0/0	0/0	0/0	0/0	0/0	1/1	0/0	0/0	0/0	0/0	0/0	0/0	0/0	0/0	1/1	./.	0/0	0/0	0/0	0/0	0/0	0/0	0/0	0/0
0/0	0/0	0/0	0/0	0/0	0/0	0/0	0/0	0/0	0/0	0/0	0/0	0/0	0/0	0/0	0/0	0/0	./.	0/0	0/0	0/0	0/0	0/0	0/0	0/0	0/0
0/0	0/0	0/0	0/0	0/0	0/0	0/0	0/0	0/0	0/0	0/0	0/0	0/0	0/0	0/0	0/0	0/0	./.	0/0	0/0	0/0	0/0	0/0	0/0	0/0	0/0
0/0	1/1	0/0	1/1	0/0	1/1	0/0	0/0	0/0	0/0	0/0	0/0	0/0	0/0	0/0	0/1	0/0	./.	0/0	0/0	0/0	0/0	0/0	0/0	0/0	0/0
0/0	0/0	0/0	0/0	0/0	0/0	0/0	0/0	0/0	0/0	0/0	0/0	0/0	0/0	0/0	0/0	0/0	./.	0/0	0/0	0/0	0/0	0/0	0/0	0/0	0/0
0/0	0/0	0/0	0/0	0/0	0/0	0/0	0/0	0/0	0/0	0/0	0/0	0/0	0/0	0/0	0/0	0/0	./.	0/0	0/0	0/0	0/0	0/0	0/0	0/0	0/0
0/0	1/1	0/0	1/1	0/0	1/1	0/0	1/1	0/0	0/0	0/0	1/1	0/0	1/1	0/0	1/1	1/1	./.	0/1	0/0	0/0	1/1	0/0	1/1	1/1	0/0
0/0	1/1	0/0	1/1	0/0	1/1	0/0	1/1	0/0	0/0	0/0	1/1	0/0	1/1	0/0	1/1	1/1	./.	0/1	0/0	0/0	1/1	0/0	1/1	1/1	0/0
0/0	0/0	0/0	0/0	0/0	0/0	0/0	0/0	0/0	0/0	0/0	0/0	0/0	0/0	0/0	0/0	0/0	./.	0/0	0/0	0/0	0/0	0/0	0/0	0/0	0/0
0/0	0/0	0/0	0/0	0/0	0/0	0/0	1/1	0/0	0/0	0/0	0/0	0/0	0/0	0/0	0/0	1/1	./.	0/0	0/0	0/0	0/0	0/0	0/0	0/0	0/0
0/0	0/0	0/0	0/0	0/0	0/0	0/0	0/0	0/0	0/0	0/0	1/1	0/0	1/1	0/0	0/1	0/0	./.	0/0	0/0	0/0	1/1	0/0	1/1	1/1	0/0
0/0	1/1	2/2	1/1	0/0	1/1	0/0	1/1	0/0	0/0	0/0	1/1	2/2	1/1	2/2	1/1	1/1	./.	2/2	0/0	0/0	1/1	0/0	1/1	1/1	2/2
0/0	1/1	0/0	1/1	0/0	1/1	0/0	1/1	0/0	0/0	0/0	1/1	0/0	1/1	0/0	1/1	1/1	./.	0/0	0/0	0/0	1/1	0/0	1/1	1/1	0/0
0/0	0/0	0/0	0/0	0/0	0/0	0/0	0/0	0/0	0/0	0/0	1/1	0/0	1/1	0/0	0/1	0/0	0/0	0/0	0/0	0/0	1/1	0/0	0/0	1/1	0/0
0/0	0/0	0/0	0/0	0/0	0/0	0/0	0/0	0/0	0/0	0/0	1/1	0/0	1/1	0/0	0/1	0/0	0/0	0/0	0/0	0/0	1/1	0/0	0/0	1/1	0/0
0/0	0/0	0/0	0/0	0/0	0/0	0/0	1/1	0/0	0/0	0/0	0/0	0/0	0/0	0/0	0/0	1/1	0/0	0/0	0/0	0/0	0/0	0/0	1/1	0/0	0/0
0/0	0/0	0/0	0/0	0/0	0/0	0/0	0/0	0/0	0/0	0/0	0/0	0/0	0/0	0/0	0/0	0/0	0/0	0/0	0/0	0/0	0/0	0/0	0/0	0/0	0/0
0/0	0/0	0/0	0/0	0/0	0/0	0/0	0/0	0/0	0/0	0/0	0/0	0/0	0/0	0/0	0/0	0/0	0/0	0/0	0/0	0/0	0/0	0/0	0/0	0/0	0/0
0/0	0/0	0/0	0/0	0/0	0/0	0/0	0/0	0/0	0/0	0/0	0/0	0/0	0/0	0/0	0/0	0/0	0/0	0/0	0/0	0/0	0/0	0/0	0/0	0/0	0/0
0/0	0/0	0/0	0/0	0/0	0/0	0/0	0/0	0/0	0/0	0/0	0/0	0/0	0/0	0/0	0/0	0/0	0/0	0/0	0/0	0/0	0/0	0/0	0/0	0/0	0/0
0/0	0/0	0/0	0/0	0/0	0/0	0/0	0/0	0/0	0/0	0/0	0/0	0/0	0/0	0/0	0/0	0/0	0/0	0/1	0/0	0/0	0/0	0/0	0/0	0/0	0/0
0/0	0/0	0/0	0/0	0/0	0/0	0/0	0/0	0/0	0/0	0/0	0/0	0/0	0/0	0/0	0/0	0/0	0/0	0/1	0/0	0/0	0/0	0/0	0/0	0/0	0/0

0/0	0/0	0/0	0/0	0/0	0/0	0/0	0/0	0/0	0/0	0/0	0/0	0/0	0/0	0/0	0/0	0/0	0/0	0/1	0/0	0/0	0/0	0/0	0/0	0/0	0/0
0/0	0/0	0/0	0/0	0/0	0/0	0/0	0/0	0/0	0/0	0/0	0/0	0/0	0/0	0/0	0/0	0/0	0/0	0/1	0/0	0/0	0/0	0/0	0/0	0/0	0/0
0/0	0/0	0/0	0/0	0/0	0/0	0/0	0/0	0/0	0/0	0/0	0/0	0/0	0/0	0/0	0/0	0/0	0/0	0/1	0/0	0/0	0/0	0/0	0/0	0/0	0/0
0/0	0/0	0/0	0/0	0/0	0/0	0/0	0/0	0/0	0/0	0/0	0/0	0/0	0/0	0/0	0/0	0/0	0/0	0/1	0/0	0/0	0/0	0/0	0/0	0/0	0/0
0/0	0/0	0/0	0/0	0/0	0/0	0/0	0/0	0/0	0/0	0/0	0/0	0/0	0/0	0/0	0/0	0/0	0/0	0/1	0/0	0/0	0/0	0/0	0/0	0/0	0/0
0/0	0/0	0/0	0/0	0/0	0/0	0/0	0/0	0/0	0/0	0/0	0/0	0/0	0/0	0/0	0/0	0/0	0/0	0/1	0/0	0/0	0/0	0/0	0/0	0/0	0/0
0/0	0/0	0/0	0/0	0/0	0/0	0/0	0/0	0/0	0/0	0/0	0/0	0/0	0/0	0/0	0/0	0/0	0/0	0/0	0/0	0/0	0/0	0/0	0/0	0/0	0/0
0/0	0/0	0/0	0/0	0/0	0/0	0/0	0/0	0/0	0/0	0/0	0/0	0/0	0/0	0/0	0/0	0/0	0/0	0/0	0/0	0/0	0/0	0/0	0/0	0/0	0/0
0/0	0/0	0/0	0/0	0/0	0/0	0/0	0/0	0/0	0/0	0/0	0/0	0/0	0/0	0/0	0/0	0/0	0/0	0/0	0/0	0/0	0/0	0/0	0/0	0/0	0/0
0/0	0/0	0/0	0/0	0/0	0/0	0/0	0/0	0/0	0/0	0/0	0/0	0/0	0/0	0/0	0/0	0/0	0/0	0/0	0/0	0/0	0/0	0/0	0/0	0/0	0/0
0/0	1/1	1/1	1/1	0/0	1/1	0/0	1/1	0/0	0/0	0/0	1/1	1/1	1/1	1/1	1/1	1/1	1/1	1/1	0/0	0/0	1/1	0/0	1/1	1/1	0/0
0/0	0/0	0/0	0/0	0/0	0/0	0/0	0/0	0/0	0/0	0/0	0/0	0/0	0/0	0/0	0/0	0/0	0/0	0/0	0/0	0/0	0/0	0/0	0/0	0/0	0/0
0/0	0/0	0/0	0/0	0/0	0/0	0/0	0/0	0/0	0/0	0/0	0/0	0/0	0/0	0/0	0/0	0/0	0/0	0/0	0/0	0/0	0/0	0/0	0/0	0/0	0/0
0/0	1/1	0/0	1/1	0/0	1/1	0/0	0/0	0/0	0/0	0/0	0/0	0/0	0/0	0/0	0/1	0/0	1/1	0/0	0/0	0/0	0/0	0/0	0/0	0/0	0/0
0/0	0/0	0/0	0/0	0/0	0/0	0/0	0/0	0/0	0/0	0/0	0/0	0/0	0/0	0/0	0/0	0/0	0/0	0/0	0/0	0/0	0/0	0/0	0/0	0/0	0/0
0/0	0/0	0/0	0/0	0/0	0/0	0/0	0/0	0/0	0/0	0/0	0/0	0/0	0/0	0/0	0/0	0/0	0/0	0/0	0/0	0/0	0/0	0/0	0/0	0/0	0/0
0/0	1/1	0/0	1/1	0/0	1/1	0/0	1/1	0/0	0/0	0/0	1/1	0/0	1/1	0/0	1/1	1/1	1/1	0/0	0/0	0/0	1/1	0/0	1/1	1/1	0/0
0/0	0/0	0/0	0/0	0/0	0/0	0/0	0/0	0/0	0/0	0/0	./.	0/0	0/0	0/0	0/0	0/0	0/0	0/0	0/0	0/0	0/0	0/0	./.	0/0	0/0
0/0	1/1	0/0	1/1	0/0	1/1	0/0	1/1	0/0	0/0	0/0	./.	0/0	./.	0/0	0/1	1/1	1/1	0/0	0/0	0/0	0/0	0/0	1/1	0/0	0/0
0/0	0/0	0/0	0/0	0/0	0/0	0/0	0/0	0/0	0/0	0/0	1/1	0/0	1/1	0/0	0/1	0/0	0/0	0/0	0/0	0/0	1/1	0/0	0/0	1/1	0/0
0/0	0/0	0/0	0/0	0/0	0/0	0/0	0/0	0/0	0/0	0/0	1/1	0/0	1/1	0/0	0/1	0/0	0/0	0/0	0/0	0/0	1/1	0/0	0/0	1/1	0/0
0/0	0/0	0/0	0/0	0/0	0/0	0/0	./.	0/0	0/0	0/0	0/0	0/0	0/0	0/0	0/0	./.	0/0	0/0	0/0	0/0	0/0	0/0	0/0	0/0	0/0
0/0	./.	0/0	0/0	0/0	./.	0/0	0/1	0/0	0/0	0/0	0/0	./.	0/0	0/0	0/0	./.	./.	0/0	0/0	0/0	0/0	0/0	0/0	0/0	0/0
0/0	0/1	0/0	0/1	0/0	1/1	0/0	0/1	0/0	0/0	0/0	0/0	./.	0/0	0/0	0/1	1/1	1/1	0/0	0/0	0/0	0/0	0/0	1/1	0/0	0/0

0/0	0/1	0/0	0/0	0/0	2/2	0/0	0/2	0/0	0/0	0/0	0/0	./.	0/0	0/0	0/0	2/2	0/0	0/0	0/0	0/0	0/0	0/0	2/2	0/0	0/0
0/0	0/0	3/3	0/0	0/0	4/4	0/0	0/4	0/0	0/0	0/0	0/0	3/3	0/0	0/0	0/4	4/4	4/4	3/3	1/1	0/0	0/0	0/0	4/4	0/0	0/0
0/0	0/0	0/0	0/0	0/0	0/0	0/0	0/0	0/0	0/0	0/0	0/0	0/0	0/0	0/0	0/0	0/0	0/0	0/0	0/0	0/0	0/0	0/0	0/0	0/0	0/0
0/0	1/1	0/0	1/1	0/0	1/1	0/0	1/1	0/0	0/0	0/0	1/1	0/0	1/1	0/0	1/1	1/1	1/1	0/0	0/0	0/0	1/1	0/0	1/1	1/1	0/0
0/0	1/1	0/0	1/1	0/0	1/1	0/0	1/1	0/0	0/0	0/0	0/0	0/0	0/0	0/0	0/1	1/1	1/1	0/0	0/0	0/0	0/0	0/0	1/1	0/0	0/0
0/0	0/0	0/0	0/0	0/0	0/0	0/0	0/0	0/0	0/0	0/0	0/0	0/0	0/0	0/0	0/0	0/0	0/0	0/0	0/0	0/0	0/0	0/0	0/0	0/0	0/0
0/0	0/0	0/0	0/0	0/0	0/0	0/0	0/0	0/0	0/0	0/0	0/0	0/0	0/0	0/0	0/0	0/0	0/0	0/0	0/0	0/0	0/0	0/0	0/0	0/0	0/0
0/0	1/1	0/0	1/1	0/0	1/1	0/0	1/1	0/0	0/0	0/0	1/1	0/0	1/1	0/0	1/1	1/1	1/1	0/0	0/0	0/0	1/1	0/1	1/1	1/1	0/0
0/0	0/0	0/0	0/0	0/0	./.	0/0	0/0	0/0	0/0	0/0	0/0	0/0	0/0	0/0	0/0	0/0	0/0	0/0	0/0	0/0	0/0	0/0	0/0	0/0	0/0
0/0	1/1	0/0	1/1	0/0	1/1	0/0	1/1	0/0	0/0	0/0	0/0	0/0	0/0	0/0	0/1	1/1	1/1	0/0	0/0	0/0	0/0	0/0	0/1	0/0	0/0
0/0	1/1	0/0	1/1	0/0	1/1	0/0	1/1	0/0	0/0	0/0	0/0	0/0	0/0	0/0	0/1	1/1	1/1	0/0	0/0	0/0	0/0	0/0	0/1	0/0	0/0
0/0	1/1	0/0	1/1	0/0	1/1	0/0	1/1	0/0	0/0	0/0	0/0	0/0	0/0	0/0	0/1	1/1	1/1	0/0	0/0	0/0	0/0	0/0	0/1	0/0	0/0
0/0	0/0	0/0	0/0	0/0	0/0	0/0	0/0	0/0	0/0	0/0	1/1	0/0	1/1	0/0	0/1	0/0	0/0	0/0	0/0	0/0	1/1	0/0	0/1	1/1	0/0
0/0	1/1	0/0	1/1	0/0	1/1	0/0	1/1	0/0	0/0	0/0	0/0	0/0	0/0	0/0	0/1	1/1	1/1	0/0	0/0	0/0	0/0	0/0	0/1	0/0	0/0
0/0	1/1	0/0	1/1	0/0	1/1	0/0	1/1	0/0	0/0	0/0	1/1	0/0	1/1	0/0	1/1	1/1	1/1	0/0	0/0	0/0	1/1	0/0	1/1	1/1	0/0
0/0	0/0	0/0	0/0	0/0	0/0	0/0	0/0	0/0	0/0	0/0	0/0	0/0	0/0	0/0	0/0	0/0	0/0	0/0	0/0	0/0	0/0	0/0	0/0	0/0	0/0
0/0	0/0	0/0	0/0	0/0	1/1	0/0	0/0	0/0	0/0	0/0	0/0	0/0	0/0	0/0	0/0	0/0	0/0	0/0	0/0	0/0	0/0	0/0	0/0	0/0	0/0
0/0	0/0	0/0	0/0	0/0	0/0	0/0	0/0	0/0	0/0	0/0	1/1	0/0	1/1	0/0	0/1	0/0	0/0	0/0	0/0	0/0	1/1	0/0	0/0	1/1	0/0
0/0	0/0	0/0	0/0	0/0	0/0	0/0	0/0	0/0	0/0	0/0	0/0	0/0	0/0	0/0	0/0	0/0	0/0	0/0	0/0	0/0	0/0	0/0	0/0	0/0	0/0
0/0	0/0	0/0	0/0	0/0	0/0	0/0	0/0	0/0	0/0	0/0	1/1	0/0	1/1	0/0	0/1	0/0	0/0	0/0	0/0	0/0	1/1	0/0	0/0	1/1	0/0
0/0	1/1	0/0	1/1	0/0	1/1	0/0	0/0	0/0	0/0	0/0	0/0	0/0	0/0	0/0	0/1	0/0	1/1	0/0	0/0	0/0	0/0	0/0	0/0	0/0	0/0
0/0	0/0	0/0	0/0	0/0	0/0	0/0	0/0	0/0	0/0	0/0	1/1	0/0	1/1	0/0	0/1	0/0	0/0	0/0	0/0	0/0	1/1	0/0	0/0	1/1	0/0
0/0	0/0	0/0	0/0	0/0	0/0	0/0	0/0	0/0	0/0	0/0	1/1	0/0	1/1	0/0	0/1	0/0	0/0	0/0	0/0	0/0	1/1	0/0	0/0	1/1	0/0
0/0	0/0	0/0	0/0	0/0	0/0	0/0	0/0	0/0	0/0	0/0	0/0	0/0	0/0	0/0	0/0	0/0	0/0	0/0	0/0	0/0	0/0	0/0	0/0	0/0	0/0
0/0	0/0	0/0	0/0	0/0	0/0	0/0	0/0	0/0	0/0	0/0	0/0	0/0	0/0	0/0	0/0	0/0	0/0	0/0	0/0	0/0	0/0	0/0	0/0	0/0	0/0
0/0	1/1	0/0	1/1	0/0	1/1	0/0	1/1	0/0	0/0	0/0	0/0	0/0	0/0	0/0	0/1	1/1	1/1	0/0	0/0	0/0	0/0	0/0	0/1	0/0	0/0
0/0	0/0	0/0	0/0	0/0	0/0	0/0	0/0	0/0	0/0	0/0	0/0	0/0	0/0	0/0	0/0	0/0	0/0	0/0	0/0	0/0	0/0	0/0	0/0	0/0	0/0
0/0	1/1	0/0	1/1	0/0	1/1	0/0	1/1	0/0	0/0	0/0	1/1	0/0	1/1	0/0	1/1	1/1	1/1	0/0	0/0	0/0	1/1	0/0	1/1	1/1	0/0
0/0	0/0	0/0	0/0	0/0	0/0	0/0	0/0	0/0	0/0	0/0	0/0	0/0	0/0	0/0	0/0	0/0	0/0	0/0	0/0	0/0	0/0	0/0	0/0	0/0	0/0
0/0	1/1	0/0	1/1	0/0	1/1	0/0	1/1	0/0	0/0	0/0	1/1	0/0	1/1	0/0	1/1	1/1	1/1	0/0	0/0	0/0	1/1	0/0	1/1	1/1	0/0

0/0	0/0	0/0	0/0	0/0	0/0	0/0	0/0	0/0	0/0	0/0	0/0	0/0	0/0	0/0	0/0	0/0	0/0	0/0	0/0	0/0	0/0	0/0	0/0	0/0	0/0
0/0	0/0	0/0	0/0	0/0	0/0	0/0	0/0	0/0	0/0	0/0	0/0	0/0	0/0	0/0	0/0	0/0	0/0	0/0	0/0	0/0	0/0	0/0	0/0	0/0	0/0
0/0	0/0	0/0	0/0	0/0	0/0	0/0	0/0	0/0	0/0	0/0	0/0	0/0	0/0	0/0	0/0	0/0	0/0	0/0	0/0	0/0	0/0	0/0	0/0	0/0	0/0
0/0	0/0	0/0	0/0	0/0	0/0	0/0	1/1	0/0	0/0	0/0	0/0	0/0	0/0	0/0	0/0	1/1	0/0	0/0	0/0	0/0	0/0	0/0	1/1	0/0	0/0
0/0	1/1	0/0	1/1	0/0	1/1	0/0	1/1	0/0	0/0	0/0	0/0	0/0	0/0	0/0	0/1	1/1	1/1	0/0	0/0	0/0	0/0	0/0	1/1	0/0	0/0
0/0	1/1	0/0	1/1	0/0	1/1	0/0	1/1	0/0	0/0	0/0	1/1	0/0	1/1	0/0	1/1	1/1	1/1	0/0	0/0	0/0	1/1	0/0	1/1	1/1	0/0
0/0	1/1	0/0	1/1	0/0	1/1	0/0	1/1	0/0	0/0	0/0	0/0	0/0	0/0	0/0	0/1	1/1	1/1	0/0	0/0	0/0	0/0	0/0	1/1	0/0	0/0
0/0	1/1	0/0	1/1	0/0	1/1	0/0	0/0	0/0	0/0	0/0	0/0	0/0	0/0	0/0	0/1	0/0	1/1	0/0	0/0	0/0	0/0	0/0	0/0	0/0	0/0
0/0	0/0	0/0	0/0	0/0	0/0	0/0	0/0	0/0	0/0	0/0	0/0	0/0	0/0	0/0	0/0	0/0	0/0	0/0	0/0	0/0	0/0	0/0	0/0	0/0	0/0
0/0	0/0	0/0	0/0	0/0	0/0	0/0	0/0	0/0	0/0	0/0	0/0	0/0	0/0	0/0	0/0	0/0	0/0	0/0	0/0	0/0	0/0	0/0	0/0	0/0	0/0
0/0	0/0	0/0	0/0	0/0	0/0	0/0	0/0	0/0	0/0	0/0	0/0	0/0	0/0	0/0	0/0	0/0	0/0	0/0	0/0	0/0	0/0	0/0	0/0	0/0	0/0
0/0	0/0	0/0	0/0	0/0	0/0	0/0	0/0	0/0	0/0	0/0	1/1	0/0	1/1	0/0	0/1	0/0	0/0	0/0	0/0	0/0	1/1	0/0	0/0	1/1	0/0
0/0	0/0	0/0	0/0	0/0	0/0	0/0	1/1	0/0	0/0	0/0	1/1	0/0	1/1	0/0	0/1	1/1	0/0	0/0	0/0	0/0	0/0	0/0	1/1	0/0	0/0
0/0	1/1	0/0	1/1	0/0	1/1	0/0	1/1	0/0	0/0	0/0	1/1	0/0	1/1	0/0	1/1	1/1	1/1	0/0	0/0	0/0	1/1	0/0	1/1	1/1	0/0
0/0	1/1	1/1	1/1	0/0	1/1	1/1	1/1	1/1	0/0	1/1	1/1	1/1	1/1	1/1	1/1	1/1	1/1	1/1	1/1	1/1	1/1	0/0	1/1	1/1	1/1
0/0	0/0	0/0	0/0	0/0	0/0	0/0	0/0	0/0	0/0	0/0	0/0	0/0	0/0	0/0	0/0	0/0	0/0	0/0	0/0	0/0	0/0	0/0	0/0	0/0	0/0
0/0	0/0	0/0	0/0	0/0	0/0	0/0	1/1	0/0	0/0	0/0	0/0	0/0	0/0	0/0	0/0	1/1	0/0	0/0	0/0	0/0	0/0	0/0	1/1	0/0	0/0
0/0	1/1	0/0	1/1	0/0	1/1	0/0	1/1	0/0	0/0	0/0	0/0	0/0	0/0	0/0	0/1	1/1	1/1	0/0	0/0	0/0	0/0	0/0	1/1	0/0	0/0
0/0	1/1	0/0	1/1	0/0	1/1	0/0	1/1	0/0	0/0	0/0	1/1	0/0	1/1	0/0	1/1	1/1	1/1	0/0	0/0	0/0	1/1	0/0	1/1	1/1	0/0
0/0	0/0	0/0	0/0	0/0	0/0	0/0	0/0	0/0	0/0	0/0	0/0	0/0	0/0	0/0	0/0	0/0	0/0	0/0	0/0	0/0	0/0	0/0	0/0	0/0	0/0
0/0	0/0	0/0	0/0	0/0	0/0	0/0	0/0	0/0	0/0	0/0	0/0	0/0	0/0	0/0	0/0	0/0	0/0	0/0	0/0	0/0	0/0	0/0	0/0	0/0	0/0
0/0	0/0	0/0	0/0	0/0	0/0	0/0	0/0	0/0	0/0	0/0	0/0	0/0	0/0	0/0	0/0	0/0	0/0	0/0	0/0	0/0	0/0	0/0	0/0	0/0	0/0
0/0	1/1	0/0	1/1	0/0	1/1	0/0	1/1	0/0	0/0	0/0	1/1	0/0	1/1	0/0	1/1	1/1	1/1	0/0	0/0	0/0	1/1	0/0	1/1	1/1	0/0
0/0	0/0	0/0	0/0	0/0	0/0	0/0	0/0	0/0	0/0	0/0	0/0	0/0	0/0	0/0	0/0	0/0	0/0	0/0	0/0	0/0	0/0	0/0	0/0	0/0	0/0
0/0	0/0	0/0	0/0	0/0	0/0	0/0	0/0	0/0	0/0	0/0	0/0	0/0	0/0	0/0	0/0	0/0	0/0	0/0	0/0	0/0	0/0	0/0	0/0	0/0	0/0
0/0	0/0	0/0	0/0	0/0	0/0	0/0	0/0	0/0	0/0	0/0	0/0	0/0	0/0	0/0	0/0	0/0	0/0	0/0	0/0	0/0	0/0	0/0	0/0	0/0	0/0
0/0	0/0	0/0	0/0	0/0	0/0	0/0	0/0	0/0	0/0	0/0	0/0	0/0	0/0	0/0	0/0	0/0	0/0	0/0	0/0	0/0	0/0	0/0	0/0	0/0	0/0
0/0	0/0	0/0	0/0	0/0	0/0	0/0	0/0	0/0	0/0	0/0	0/0	0/0	0/0	0/0	0/0	0/0	0/0	0/0	0/0	0/0	0/0	0/0	0/0	0/0	0/0
0/0	0/0	0/0	0/0	0/0	0/0	0/0	0/0	0/0	0/0	0/0	0/0	0/0	0/0	0/0	0/0	0/0	0/0	0/0	0/0	0/0	0/0	0/0	0/0	0/0	0/0
0/0	0/0	0/0	0/0	0/0	0/0	0/0	0/0	0/0	0/0	0/0	0/0	0/0	0/0	0/0	0/0	0/0	0/0	0/0	0/0	0/0	0/0	0/0	0/0	0/0	0/0
0/0	0/0	0/0	0/0	0/0	0/0	0/0	0/0	0/0	0/0	0/0	0/0	0/0	0/0	0/0	0/0	0/0	0/0	0/0	0/0	0/0	0/0	0/0	0/0	0/0	0/0
0/0	0/0	0/0	0/0	0/0	0/0	0/0	0/0	0/0	0/0	0/0	1/1	0/0	1/1	0/0	0/1	0/0	0/0	0/0	0/0	0/0	1/1	0/1	0/0	1/1	0/0
0/0	0/0	0/0	0/0	0/0	0/0	0/0	0/0	0/0	0/0	0/0	0/0	0/0	0/0	0/0	0/0	0/0	0/0	0/0	0/0	0/0	0/0	0/0	0/0	0/0	0/0
0/0	0/0	0/0	0/0	0/0	0/0	0/0	0/0	0/0	0/0	0/0	0/0	0/0	0/0	0/0	0/0	0/0	0/0	0/0	0/0	0/0	0/0	0/0	0/0	0/0	0/0
0/0	0/0	0/0	0/0	0/0	0/0	0/0	0/0	0/0	0/0	0/0	0/0	0/0	0/0	0/0	0/0	1/1	0/0	0/0	0/0	0/0	0/0	0/0	1/1	0/0	0/0

0/0	1/1	0/0	1/1	0/0	1/1	0/0	0/0	0/0	0/0	0/0	0/0	0/0	0/0	0/0	0/1	0/0	1/1	0/0	0/0	0/0	0/0	0/0	0/0	0/0	0/0
0/0	4/4	2/2	4/4	0/0	4/4	4/4	2/2	4/4	0/0	1/1	1/1	1/2	1/1	3/3	1/4	2/2	4/4	2/2	3/5	0/0	1/1	0/0	2/3	1/1	3/3
0/0	./.	./.	./.	0/0	./.	./.	./.	./.	0/0	./.	./.	./.	./.	./.	./.	./.	./.	./.	./.	0/0	./.	0/0	./.	./.	./.
0/0	0/0	0/0	0/0	0/0	0/0	0/0	0/0	0/0	0/0	0/0	0/0	0/0	0/0	0/0	0/0	0/0	0/0	0/0	0/0	0/0	0/0	0/0	0/0	0/0	0/0
0/0	0/0	0/0	0/0	0/0	0/0	0/0	0/0	0/0	0/0	0/0	0/0	0/0	0/0	0/0	0/0	0/0	0/0	0/0	0/0	0/0	0/0	0/0	0/0	0/0	0/0
0/0	0/0	0/0	0/0	0/0	0/0	0/0	0/0	0/0	0/0	0/0	0/0	0/0	0/0	0/0	0/0	0/0	0/0	0/0	0/0	0/0	0/0	0/0	0/0	0/0	0/0
0/0	1/1	0/0	1/1	0/0	1/1	0/0	0/0	0/0	0/0	0/0	0/0	0/0	0/0	0/0	0/1	0/0	1/1	0/0	0/0	0/0	0/0	0/0	0/0	0/0	0/0
0/0	1/1	0/0	1/1	0/0	1/1	0/0	0/0	0/0	0/0	0/0	0/0	0/0	0/0	0/0	0/1	0/0	1/1	0/0	0/0	0/0	0/0	0/0	0/0	0/0	0/0
0/0	0/0	0/0	0/0	0/0	./.	0/0	0/0	0/0	0/0	0/0	0/0	0/0	0/0	0/0	0/0	0/0	./.	0/0	0/0	0/0	0/0	0/0	0/0	0/0	0/0
0/0	1/1	./.	1/1	0/0	1/1	0/0	1/1	0/0	0/0	0/0	./.	./.	./.	./.	0/1	1/1	1/1	0/0	0/0	0/0	./.	0/0	1/1	./.	./.
0/0	./.	1/1	./.	0/0	./.	0/0	./.	0/0	0/0	0/0	2/2	1/1	2/2	1/1	0/2	./.	./.	1/1	1/1	0/0	2/2	0/0	./.	2/2	1/1
0/0	./.	2/2	./.	0/0	0/0	0/0	./.	0/0	0/0	0/0	./.	2/2	./.	2/2	0/0	./.	0/0	2/2	2/2	0/0	./.	0/0	0/0	./.	2/2
0/0	0/0	0/0	0/0	0/0	0/0	0/0	0/0	0/0	0/0	0/0	0/0	0/0	0/0	0/0	0/0	0/0	0/0	0/0	0/0	0/0	0/0	0/0	0/0	0/0	0/0
0/0	0/0	0/0	0/0	0/0	0/0	0/0	0/0	0/0	0/0	0/0	0/0	0/0	0/0	0/0	0/0	0/0	0/0	0/0	0/0	0/0	0/0	0/0	0/0	0/0	0/0
0/0	0/0	0/0	0/0	0/0	0/0	0/0	0/0	0/0	0/0	0/0	0/0	0/0	0/0	0/0	0/0	0/0	0/0	0/0	0/0	0/0	0/0	0/0	0/0	0/0	0/0
0/0	1/1	0/0	1/1	0/0	1/1	0/0	1/1	0/0	0/0	0/0	1/1	0/0	1/1	0/0	1/1	1/1	1/1	0/0	0/0	0/0	1/1	0/0	1/1	1/1	0/0
0/0	0/0	0/0	0/0	0/0	0/0	0/0	0/0	0/0	0/0	0/0	0/0	0/0	0/0	0/0	0/0	0/0	0/0	0/0	0/0	0/0	0/0	0/0	0/0	0/0	0/0
0/0	0/0	0/0	0/0	0/0	0/0	0/0	1/1	0/0	0/0	0/0	0/0	0/0	0/0	0/0	0/0	1/1	0/0	0/0	0/0	0/0	0/0	0/0	1/1	0/0	0/0
0/0	0/0	0/0	0/0	0/0	0/0	0/0	0/0	0/0	0/0	0/0	0/0	0/0	0/0	0/0	0/0	0/0	0/0	0/0	0/0	0/0	0/0	0/0	0/0	0/0	0/0
0/0	0/0	0/0	0/0	0/0	0/0	0/0	0/0	0/0	0/0	0/0	0/0	0/0	0/0	0/0	0/0	0/0	0/0	0/0	0/0	0/0	0/0	0/0	0/0	0/0	0/0
0/0	0/0	0/0	0/0	0/0	0/0	0/0	0/0	0/0	0/0	0/0	0/0	0/0	0/0	0/0	0/0	0/0	0/0	0/0	0/0	0/0	0/0	0/0	0/0	0/0	0/0

0/0	0/0	0/0	0/0	0/0	0/0	0/0	0/0	0/0	0/0	0/0	0/0	0/0	0/0	0/0	0/0	0/0	0/0	0/0	0/0	0/0	0/0	0/0	0/0	0/0	0/0
0/0	0/0	0/0	0/0	0/0	0/0	0/0	0/0	0/0	0/0	0/0	0/0	0/0	0/0	0/0	0/0	0/0	0/0	0/0	0/0	0/0	0/0	0/0	0/0	0/0	0/0
0/0	1/1	0/0	1/1	0/0	1/1	0/0	1/1	0/0	0/0	0/0	2/2	0/0	2/2	0/0	1/2	2/2	1/1	0/0	0/0	0/0	2/2	0/0	1/1	2/2	0/0
0/0	0/0	0/0	0/0	0/0	0/0	0/0	0/0	0/0	0/0	0/0	0/0	0/0	0/0	0/0	0/0	0/0	0/0	0/0	0/0	0/0	0/0	0/0	0/0	0/0	0/0
0/0	0/0	0/0	0/0	0/0	0/0	0/0	1/1	0/0	0/0	0/0	2/2	0/0	2/2	0/0	0/2	2/2	0/0	0/0	0/0	0/0	2/2	0/0	1/1	2/2	0/0
0/0	0/0	0/0	0/0	0/0	0/0	0/0	0/0	0/0	0/0	0/0	0/0	0/0	0/0	0/0	0/0	0/0	0/0	0/0	0/0	0/0	0/0	0/0	0/0	0/0	0/0
0/0	1/1	0/0	1/1	0/0	1/1	0/0	0/0	0/0	0/0	0/0	0/0	0/0	0/0	0/0	0/1	0/0	1/1	0/0	0/0	0/0	0/0	0/0	0/0	0/0	0/0
0/0	1/1	0/0	1/1	0/0	1/1	0/0	1/1	0/0	0/0	0/0	1/1	0/0	1/1	0/0	1/1	1/1	1/1	0/0	0/0	0/0	1/1	0/0	1/1	1/1	0/0
0/0	1/1	0/0	1/1	0/0	1/1	0/0	1/1	0/0	0/0	0/0	1/1	0/0	1/1	0/0	1/1	1/1	1/1	0/0	0/0	0/0	1/1	0/0	1/1	1/1	0/0
0/0	0/0	0/0	0/0	0/0	0/0	0/0	0/0	0/0	0/0	0/0	0/0	0/0	0/0	0/0	0/0	0/0	0/0	0/0	0/0	0/0	0/0	0/0	0/0	0/0	0/0
0/0	2/2	0/0	2/2	0/0	2/2	0/0	1/1	0/0	0/0	0/0	2/2	0/0	2/2	0/0	2/2	2/2	2/2	0/0	0/0	0/0	2/2	0/0	1/1	2/2	0/0
0/0	0/0	0/0	0/0	0/0	0/0	0/0	0/0	0/0	0/0	0/0	0/0	0/0	0/0	0/0	0/0	0/0	0/0	0/0	0/0	0/0	0/0	0/0	0/0	0/0	0/0
0/0	0/0	0/0	0/0	0/0	0/0	0/0	0/0	0/0	0/0	0/0	0/0	0/0	0/0	0/0	0/0	0/0	0/0	0/0	0/0	0/0	0/0	0/0	0/0	0/0	0/0
0/0	0/0	0/0	0/0	0/0	0/0	0/0	0/0	0/0	0/0	0/0	0/0	0/0	0/0	0/0	0/0	0/0	0/0	0/0	0/0	0/0	0/0	0/0	0/0	0/0	0/0
0/0	1/1	0/0	1/1	0/0	1/1	0/0	0/0	0/0	0/0	0/0	0/0	0/0	0/0	0/0	0/1	0/0	1/1	0/0	0/0	0/0	0/0	0/0	0/0	0/0	0/0
0/0	0/0	0/0	0/0	0/0	0/0	0/0	0/0	0/0	0/0	0/0	0/0	0/0	0/0	0/0	0/0	0/0	0/0	0/0	0/0	0/0	0/0	0/0	0/0	0/0	0/0
0/0	1/1	0/0	1/1	0/0	1/1	0/0	0/0	0/0	0/0	0/0	0/0	0/0	0/0	0/0	0/1	0/0	1/1	0/0	0/0	0/0	0/0	0/0	0/0	0/0	0/0
0/0	1/1	0/0	1/1	0/0	1/1	0/0	1/1	0/0	0/0	0/0	1/1	0/0	1/1	0/0	1/1	1/1	1/1	0/0	0/0	0/0	1/1	0/0	1/1	1/1	0/0

<table>
<tr><th></th></tr>
<tr><td>Annotation</td></tr>
<tr><td></td></tr>
<tr><td>T|synonymous_variant|LOW|LOC_Os01g68460|LOC_Os01g68460|transcript|LOC_Os01g68460.1|protein_coding|3/3|c.489G>A|p.Gln163Gln|489/810|489/810|163/269||</td></tr>
<tr><td>A|missense_variant|MODERATE|LOC_Os01g68460|LOC_Os01g68460|transcript|LOC_Os01g68460.1|protein_coding|3/3|c.344C>T|p.Ala115Val|344/810|344/810|115/269||</td></tr>
<tr><td>T|conservative_inframe_deletion|MODERATE|LOC_Os01g68460|LOC_Os01g68460|transcript|LOC_Os01g68460.1|protein_coding|1/3|c.226_228delTTC|p.Phe76del|228/810|226/810|76/269||</td></tr>
<tr><td>A|synonymous_variant|LOW|LOC_Os01g68460|LOC_Os01g68460|transcript|LOC_Os01g68460.1|protein_coding|1/3|c.213C>T|p.His71His|213/810|213/810|71/269||</td></tr>
<tr><td>A|synonymous_variant|LOW|LOC_Os01g68460|LOC_Os01g68460|transcript|LOC_Os01g68460.1|protein_coding|1/3|c.105C>T|p.Gly35Gly|105/810|105/810|35/269||</td></tr>
<tr><td>G|missense_variant|MODERATE|LOC_Os01g68460|LOC_Os01g68460|transcript|LOC_Os01g68460.1|protein_coding|1/3|c.32A>C|p.Asn11Thr|32/810|32/810|11/269||</td></tr>
<tr><td>GACAGGCCCGTCGATCGCGGAGA|frameshift_variant|HIGH|LOC_Os01g68460|LOC_Os01g68460|transcript|LOC_Os01g68460.1|protein_coding|1/3|c.5_6insTCTCCGCGATCGACGGGCCTGT|p.Gly3fs|5/810|5/810|2/269||</td></tr>
<tr><td>A|missense_variant|MODERATE|LOC_Os01g68460|LOC_Os01g68460|transcript|LOC_Os01g68460.1|protein_coding|1/3|c.5C>T|p.Ala2Val|5/810|5/810|2/269||</td></tr>
<tr><td>C|upstream_gene_variant|MODIFIER|LOC_Os01g68460|LOC_Os01g68460|transcript|LOC_Os01g68460.1|protein_coding||c.-134A>G|||||134|</td></tr>
<tr><td>G|upstream_gene_variant|MODIFIER|LOC_Os01g68460|LOC_Os01g68460|transcript|LOC_Os01g68460.1|protein_coding||c.-166A>C|||||166|</td></tr>
<tr><td>G|upstream_gene_variant|MODIFIER|LOC_Os01g68460|LOC_Os01g68460|transcript|LOC_Os01g68460.1|protein_coding||c.-170A>C|||||170|</td></tr>
<tr><td>T|upstream_gene_variant|MODIFIER|LOC_Os01g68460|LOC_Os01g68460|transcript|LOC_Os01g68460.1|protein_coding||c.-287C>A|||||287|</td></tr>
<tr><td>G|upstream_gene_variant|MODIFIER|LOC_Os01g68460|LOC_Os01g68460|transcript|LOC_Os01g68460.1|protein_coding||c.-354G>C|||||354|</td></tr>
<tr><td>GAA|upstream_gene_variant|MODIFIER|LOC_Os01g68460|LOC_Os01g68460|transcript|LOC_Os01g68460.1|protein_coding||c.-456_-455insTT|||||456|</td></tr>
<tr><td>A|upstream_gene_variant|MODIFIER|LOC_Os01g68460|LOC_Os01g68460|transcript|LOC_Os01g68460.1|protein_coding||c.-458C>T|||||458|</td></tr>
<tr><td>AGCG|upstream_gene_variant|MODIFIER|LOC_Os01g68460|LOC_Os01g68460|transcript|LOC_Os01g68460.1|protein_coding||c.-460_-459insCGC|||||460|</td></tr>
</table>

<table>
<tr><td></td></tr>
<tr><td>GG|upstream_gene_variant|MODIFIER|LOC_Os01g68460|LOC_Os01g68460|transcript|LOC_Os01g68460.1|protein_coding||c.-461T>C|||||461|,A|upstream_gene_variant|MODIFIER|LOC_Os01g68460|LOC_Os01g68460|transcript|LOC_Os01g68460.1|protein_coding||c.-462delC|||||462|,AGG|upstream_gene_variant|MODIFIER|LOC_Os01g68460|LOC_Os01g68460|transcript|LOC_Os01g68460.1|protein_coding||c.-463_-462insC|||||463|,AGGG|upstream_gene_variant|MODIFIER|LOC_Os01g68460|LOC_Os01g68460|transcript|LOC_Os01g68460.1|protein_coding||c.-463_-462insCC|||||463|,AGGGG|upstream_gene_variant|MODIFIER|LOC_Os01g68460|LOC_Os01g68460|transcript|LOC_Os01g68460.1|protein_coding||c.-463_-462insCCC|||||463|,AGGGGG|upstream_gene_variant|MODIFIER|LOC_Os01g68460|LOC_Os01g68460|transcript|LOC_Os01g68460.1|protein_coding||c.-463_-462insCCCC|||||463|</td></tr>
<tr><td>GA|upstream_gene_variant|MODIFIER|LOC_Os01g68460|LOC_Os01g68460|transcript|LOC_Os01g68460.1|protein_coding||c.-470_-469insT|||||470|,GGA|upstream_gene_variant|MODIFIER|LOC_Os01g68460|LOC_Os01g68460|transcript|LOC_Os01g68460.1|protein_coding||c.-470_-469insTC|||||470|</td></tr>
<tr><td>G|upstream_gene_variant|MODIFIER|LOC_Os01g68460|LOC_Os01g68460|transcript|LOC_Os01g68460.1|protein_coding||c.-472T>C|||||472|</td></tr>
<tr><td>G|upstream_gene_variant|MODIFIER|LOC_Os01g68460|LOC_Os01g68460|transcript|LOC_Os01g68460.1|protein_coding||c.-499T>C|||||499|</td></tr>
<tr><td>G|upstream_gene_variant|MODIFIER|LOC_Os01g68460|LOC_Os01g68460|transcript|LOC_Os01g68460.1|protein_coding||c.-535T>C|||||535|</td></tr>
<tr><td>C|upstream_gene_variant|MODIFIER|LOC_Os01g68460|LOC_Os01g68460|transcript|LOC_Os01g68460.1|protein_coding||c.-544C>G|||||544|</td></tr>
<tr><td>T|upstream_gene_variant|MODIFIER|LOC_Os01g68460|LOC_Os01g68460|transcript|LOC_Os01g68460.1|protein_coding||c.-581G>A|||||581|</td></tr>
<tr><td>A|upstream_gene_variant|MODIFIER|LOC_Os01g68460|LOC_Os01g68460|transcript|LOC_Os01g68460.1|protein_coding||c.-622G>T|||||622|</td></tr>
<tr><td>A|upstream_gene_variant|MODIFIER|LOC_Os01g68460|LOC_Os01g68460|transcript|LOC_Os01g68460.1|protein_coding||c.-650delA|||||650|</td></tr>
<tr><td>A|upstream_gene_variant|MODIFIER|LOC_Os01g68460|LOC_Os01g68460|transcript|LOC_Os01g68460.1|protein_coding||c.-650A>T|||||650|</td></tr>
<tr><td>A|upstream_gene_variant|MODIFIER|LOC_Os01g68460|LOC_Os01g68460|transcript|LOC_Os01g68460.1|protein_coding||c.-651A>T|||||651|</td></tr>
<tr><td>C|upstream_gene_variant|MODIFIER|LOC_Os01g68460|LOC_Os01g68460|transcript|LOC_Os01g68460.1|protein_coding||c.-657A>G|||||657|</td></tr>
<tr><td>G|upstream_gene_variant|MODIFIER|LOC_Os01g68460|LOC_Os01g68460|transcript|LOC_Os01g68460.1|protein_coding||c.-815A>C|||||815|</td></tr>
<tr><td>A|upstream_gene_variant|MODIFIER|LOC_Os01g68460|LOC_Os01g68460|transcript|LOC_Os01g68460.1|protein_coding||c.-819G>T|||||819|</td></tr>
<tr><td>G|upstream_gene_variant|MODIFIER|LOC_Os01g68460|LOC_Os01g68460|transcript|LOC_Os01g68460.1|protein_coding||c.-829T>C|||||829|</td></tr>
<tr><td>C|upstream_gene_variant|MODIFIER|LOC_Os01g68460|LOC_Os01g68460|transcript|LOC_Os01g68460.1|protein_coding||c.-896A>G|||||896|</td></tr>
<tr><td>A|upstream_gene_variant|MODIFIER|LOC_Os01g68460|LOC_Os01g68460|transcript|LOC_Os01g68460.1|protein_coding||c.-899C>T|||||899|</td></tr>
<tr><td>T|upstream_gene_variant|MODIFIER|LOC_Os01g68460|LOC_Os01g68460|transcript|LOC_Os01g68460.1|protein_coding||c.-917C>A|||||917|</td></tr>
<tr><td>A|upstream_gene_variant|MODIFIER|LOC_Os01g68460|LOC_Os01g68460|transcript|LOC_Os01g68460.1|protein_coding||c.-1040C>T|||||1040|</td></tr>
<tr><td>A|upstream_gene_variant|MODIFIER|LOC_Os01g68460|LOC_Os01g68460|transcript|LOC_Os01g68460.1|protein_coding||c.-1091C>T|||||1091|</td></tr>
<tr><td>C|upstream_gene_variant|MODIFIER|LOC_Os01g68460|LOC_Os01g68460|transcript|LOC_Os01g68460.1|protein_coding||c.-1102A>G|||||1102|</td></tr>
<tr><td>G|upstream_gene_variant|MODIFIER|LOC_Os01g68460|LOC_Os01g68460|transcript|LOC_Os01g68460.1|protein_coding||c.-1139T>C|||||1139|</td></tr>
<tr><td>A|upstream_gene_variant|MODIFIER|LOC_Os01g68460|LOC_Os01g68460|transcript|LOC_Os01g68460.1|protein_coding||c.-1193C>T|||||1193|</td></tr>
<tr><td>A|upstream_gene_variant|MODIFIER|LOC_Os01g68460|LOC_Os01g68460|transcript|LOC_Os01g68460.1|protein_coding||c.-1215C>T|||||1215|</td></tr>
<tr><td>A|upstream_gene_variant|MODIFIER|LOC_Os01g68460|LOC_Os01g68460|transcript|LOC_Os01g68460.1|protein_coding||c.-1386G>T|||||1386|</td></tr>
<tr><td>CA|upstream_gene_variant|MODIFIER|LOC_Os01g68460|LOC_Os01g68460|transcript|LOC_Os01g68460.1|protein_coding||c.-1395_-1394insT|||||1395|,CAA|upstream_gene_variant|MODIFIER|LOC_Os01g68460|LOC_Os01g68460|transcript|LOC_Os01g68460.1|protein_coding||c.-1395_-1394insTT|||||1395|</td></tr>
</table>

C|upstream_gene_variant|MODIFIER|LOC_Os01g68460|LOC_Os01g68460|transcript|LOC_Os01g68460.1|protein_coding||c.-1531_-1528delTAGT|||||1528|

A|upstream_gene_variant|MODIFIER|LOC_Os01g68460|LOC_Os01g68460|transcript|LOC_Os01g68460.1|protein_coding||c.-1595delA|||||1595|

G|upstream_gene_variant|MODIFIER|LOC_Os01g68460|LOC_Os01g68460|transcript|LOC_Os01g68460.1|protein_coding||c.-1621G>C|||||1621|

T|upstream_gene_variant|MODIFIER|LOC_Os01g68460|LOC_Os01g68460|transcript|LOC_Os01g68460.1|protein_coding||c.-1638G>A|||||1638|

C|upstream_gene_variant|MODIFIER|LOC_Os01g68460|LOC_Os01g68460|transcript|LOC_Os01g68460.1|protein_coding||c.-1780T>G|||||1780|

T|upstream_gene_variant|MODIFIER|LOC_Os01g68460|LOC_Os01g68460|transcript|LOC_Os01g68460.1|protein_coding||c.-1927T>A|||||1927|

GT|upstream_gene_variant|MODIFIER|LOC_Os01g68460|LOC_Os01g68460|transcript|LOC_Os01g68460.1|protein_coding||c.-2000_-1999insA|||||2000|

C|upstream_gene_variant|MODIFIER|LOC_Os01g68460|LOC_Os01g68460|transcript|LOC_Os01g68460.1|protein_coding||c.-2032T>G|||||2032|

AGAAAAAT|upstream_gene_variant|MODIFIER|LOC_Os01g68460|LOC_Os01g68460|transcript|LOC_Os01g68460.1|protein_coding||c.-2036_-2035insATTTTTC|||||2036|

T|upstream_gene_variant|MODIFIER|LOC_Os01g68460|LOC_Os01g68460|transcript|LOC_Os01g68460.1|protein_coding||c.-2067G>A|||||2067|

T|upstream_gene_variant|MODIFIER|LOC_Os01g68460|LOC_Os01g68460|transcript|LOC_Os01g68460.1|protein_coding||c.-2131G>A|||||2131|

T|upstream_gene_variant|MODIFIER|LOC_Os01g68460|LOC_Os01g68460|transcript|LOC_Os01g68460.1|protein_coding||c.-2132G>A|||||2132|

A|upstream_gene_variant|MODIFIER|LOC_Os01g68460|LOC_Os01g68460|transcript|LOC_Os01g68460.1|protein_coding||c.-2133C>T|||||2133|

A|upstream_gene_variant|MODIFIER|LOC_Os01g68460|LOC_Os01g68460|transcript|LOC_Os01g68460.1|protein_coding||c.-2134C>T|||||2134|

C|upstream_gene_variant|MODIFIER|LOC_Os01g68460|LOC_Os01g68460|transcript|LOC_Os01g68460.1|protein_coding||c.-2185A>G|||||2185|

T|upstream_gene_variant|MODIFIER|LOC_Os01g68460|LOC_Os01g68460|transcript|LOC_Os01g68460.1|protein_coding||c.-2186G>A|||||2186|

A|upstream_gene_variant|MODIFIER|LOC_Os01g68460|LOC_Os01g68460|transcript|LOC_Os01g68460.1|protein_coding||c.-2222C>T|||||2222|

A|upstream_gene_variant|MODIFIER|LOC_Os01g68460|LOC_Os01g68460|transcript|LOC_Os01g68460.1|protein_coding||c.-2225C>T|||||2225|

T|upstream_gene_variant|MODIFIER|LOC_Os01g68460|LOC_Os01g68460|transcript|LOC_Os01g68460.1|protein_coding||c.-2250G>A|||||2250|

A|upstream_gene_variant|MODIFIER|LOC_Os01g68460|LOC_Os01g68460|transcript|LOC_Os01g68460.1|protein_coding||c.-2257C>T|||||2257|

C|upstream_gene_variant|MODIFIER|LOC_Os01g68460|LOC_Os01g68460|transcript|LOC_Os01g68460.1|protein_coding||c.-2265A>G|||||2265|

A|upstream_gene_variant|MODIFIER|LOC_Os01g68460|LOC_Os01g68460|transcript|LOC_Os01g68460.1|protein_coding||c.-2274G>T|||||2274|

A|upstream_gene_variant|MODIFIER|LOC_Os01g68460|LOC_Os01g68460|transcript|LOC_Os01g68460.1|protein_coding||c.-2300A>T|||||2300|

A|upstream_gene_variant|MODIFIER|LOC_Os01g68460|LOC_Os01g68460|transcript|LOC_Os01g68460.1|protein_coding||c.-2306G>T|||||2306|

C|upstream_gene_variant|MODIFIER|LOC_Os01g68460|LOC_Os01g68460|transcript|LOC_Os01g68460.1|protein_coding||c.-2312A>G|||||2312|

TC|upstream_gene_variant|MODIFIER|LOC_Os01g68460|LOC_Os01g68460|transcript|LOC_Os01g68460.1|protein_coding||c.-2350_-2349insG|||||2350|

T|upstream_gene_variant|MODIFIER|LOC_Os01g68460|LOC_Os01g68460|transcript|LOC_Os01g68460.1|protein_coding||c.-2385G>A|||||2385|

T|upstream_gene_variant|MODIFIER|LOC_Os01g68460|LOC_Os01g68460|transcript|LOC_Os01g68460.1|protein_coding||c.-2406G>A|||||2406|

G|upstream_gene_variant|MODIFIER|LOC_Os01g68460|LOC_Os01g68460|transcript|LOC_Os01g68460.1|protein_coding||c.-2408T>C|||||2408|

A|upstream_gene_variant|MODIFIER|LOC_Os01g68460|LOC_Os01g68460|transcript|LOC_Os01g68460.1|protein_coding||c.-2456C>T|||||2456|

C|upstream_gene_variant|MODIFIER|LOC_Os01g68460|LOC_Os01g68460|transcript|LOC_Os01g68460.1|protein_coding||c.-2486A>G|||||2486|

A|upstream_gene_variant|MODIFIER|LOC_Os01g68460|LOC_Os01g68460|transcript|LOC_Os01g68460.1|protein_coding||c.-2502C>T|||||2502|

C|upstream_gene_variant|MODIFIER|LOC_Os01g68460|LOC_Os01g68460|transcript|LOC_Os01g68460.1|protein_coding||c.-2504T>G|||||2504|

A|upstream_gene_variant|MODIFIER|LOC_Os01g68460|LOC_Os01g68460|transcript|LOC_Os01g68460.1|protein_coding||c.-2505C>T|||||2505|

A|upstream_gene_variant|MODIFIER|LOC_Os01g68460|LOC_Os01g68460|transcript|LOC_Os01g68460.1|protein_coding||c.-2511C>T|||||2511|

<table>
<tr><td>T|upstream_gene_variant|MODIFIER|LOC_Os01g68460|LOC_Os01g68460|transcript|LOC_Os01g68460.1|protein_coding||c.-2523G>A|||||2523|</td></tr>
<tr><td>A|upstream_gene_variant|MODIFIER|LOC_Os01g68460|LOC_Os01g68460|transcript|LOC_Os01g68460.1|protein_coding||c.-2524A>T|||||2524|</td></tr>
<tr><td>A|upstream_gene_variant|MODIFIER|LOC_Os01g68460|LOC_Os01g68460|transcript|LOC_Os01g68460.1|protein_coding||c.-2529C>T|||||2529|,GT|upstream_gene_variant|MODIFIER|LOC_Os01g68460|LOC_Os01g68460|transcript|LOC_Os01g68460.1|protein_coding||c.-2530_-2529insA|||||2530|</td></tr>
<tr><td>G|upstream_gene_variant|MODIFIER|LOC_Os01g68460|LOC_Os01g68460|transcript|LOC_Os01g68460.1|protein_coding||c.-2551T>C|||||2551|</td></tr>
<tr><td>GAT|upstream_gene_variant|MODIFIER|LOC_Os01g68460|LOC_Os01g68460|transcript|LOC_Os01g68460.1|protein_coding||c.-2603_-2602insAT|||||2603|</td></tr>
<tr><td>A|upstream_gene_variant|MODIFIER|LOC_Os01g68460|LOC_Os01g68460|transcript|LOC_Os01g68460.1|protein_coding||c.-2607C>T|||||2607|</td></tr>
<tr><td>G|upstream_gene_variant|MODIFIER|LOC_Os01g68460|LOC_Os01g68460|transcript|LOC_Os01g68460.1|protein_coding||c.-2620T>C|||||2620|</td></tr>
<tr><td>C|upstream_gene_variant|MODIFIER|LOC_Os01g68460|LOC_Os01g68460|transcript|LOC_Os01g68460.1|protein_coding||c.-2630A>G|||||2630|</td></tr>
<tr><td>A|upstream_gene_variant|MODIFIER|LOC_Os01g68460|LOC_Os01g68460|transcript|LOC_Os01g68460.1|protein_coding||c.-2644delA|||||2644|,ATT|upstream_gene_variant|MODIFIER|LOC_Os01g68460|LOC_Os01g68460|transcript|LOC_Os01g68460.1|protein_coding||c.-2645_-2644insA|||||2645|</td></tr>
<tr><td>C|upstream_gene_variant|MODIFIER|LOC_Os01g68460|LOC_Os01g68460|transcript|LOC_Os01g68460.1|protein_coding||c.-2659C>G|||||2659|</td></tr>
<tr><td>T|upstream_gene_variant|MODIFIER|LOC_Os01g68460|LOC_Os01g68460|transcript|LOC_Os01g68460.1|protein_coding||c.-2667C>A|||||2667|</td></tr>
<tr><td>A|upstream_gene_variant|MODIFIER|LOC_Os01g68460|LOC_Os01g68460|transcript|LOC_Os01g68460.1|protein_coding||c.-2703C>T|||||2703|</td></tr>
<tr><td>A|upstream_gene_variant|MODIFIER|LOC_Os01g68460|LOC_Os01g68460|transcript|LOC_Os01g68460.1|protein_coding||c.-2705C>T|||||2705|</td></tr>
<tr><td>A|upstream_gene_variant|MODIFIER|LOC_Os01g68460|LOC_Os01g68460|transcript|LOC_Os01g68460.1|protein_coding||c.-2710C>T|||||2710|</td></tr>
<tr><td>A|upstream_gene_variant|MODIFIER|LOC_Os01g68460|LOC_Os01g68460|transcript|LOC_Os01g68460.1|protein_coding||c.-2729C>T|||||2729|</td></tr>
<tr><td>G|upstream_gene_variant|MODIFIER|LOC_Os01g68460|LOC_Os01g68460|transcript|LOC_Os01g68460.1|protein_coding||c.-2761T>C|||||2761|</td></tr>
<tr><td>T|upstream_gene_variant|MODIFIER|LOC_Os01g68460|LOC_Os01g68460|transcript|LOC_Os01g68460.1|protein_coding||c.-2766T>A|||||2766|</td></tr>
<tr><td>GA|upstream_gene_variant|MODIFIER|LOC_Os01g68460|LOC_Os01g68460|transcript|LOC_Os01g68460.1|protein_coding||c.-2824_-2823delTC|||||2823|,G|upstream_gene_variant|MODIFIER|LOC_Os01g68460|LOC_Os01g68460|transcript|LOC_Os01g68460.1|protein_coding||c.-2825_-2823delTTC|||||2823|</td></tr>
<tr><td>GA|upstream_gene_variant|MODIFIER|LOC_Os01g68460|LOC_Os01g68460|transcript|LOC_Os01g68460.1|protein_coding||c.-2827_-2825delTTT|||||2825|,GAA|upstream_gene_variant|MODIFIER|LOC_Os01g68460|LOC_Os01g68460|transcript|LOC_Os01g68460.1|protein_coding||c.-2827_-2826delTT|||||2826|,GAAA|upstream_gene_variant|MODIFIER|LOC_Os01g68460|LOC_Os01g68460|transcript|LOC_Os01g68460.1|protein_coding||c.-2827delT|||||2827|</td></tr>
<tr><td>A|upstream_gene_variant|MODIFIER|LOC_Os01g68460|LOC_Os01g68460|transcript|LOC_Os01g68460.1|protein_coding||c.-2842C>T|||||2842|</td></tr>
<tr><td>A|upstream_gene_variant|MODIFIER|LOC_Os01g68460|LOC_Os01g68460|transcript|LOC_Os01g68460.1|protein_coding||c.-2851C>T|||||2851|</td></tr>
<tr><td>A|upstream_gene_variant|MODIFIER|LOC_Os01g68460|LOC_Os01g68460|transcript|LOC_Os01g68460.1|protein_coding||c.-2853C>T|||||2853|</td></tr>
<tr><td>T|upstream_gene_variant|MODIFIER|LOC_Os01g68460|LOC_Os01g68460|transcript|LOC_Os01g68460.1|protein_coding||c.-2863G>A|||||2863|</td></tr>
<tr><td>T|upstream_gene_variant|MODIFIER|LOC_Os01g68460|LOC_Os01g68460|transcript|LOC_Os01g68460.1|protein_coding||c.-2896G>A|||||2896|</td></tr>
<tr><td>TTCGTAC|upstream_gene_variant|MODIFIER|LOC_Os01g68460|LOC_Os01g68460|transcript|LOC_Os01g68460.1|protein_coding||c.-2903_-2902insGTACGA|||||2903|</td></tr>
</table>

<table>
<tr><td>TACTCGTACGTTATATATAA|upstream_gene_variant|MODIFIER|LOC_Os01g68460|LOC_Os01g68460|transcript|LOC_Os01g68460.1|protein_coding||c.-2907_-2906insTTATATATAACGTACGAGT|||||2907|</td></tr>
<tr><td>G|upstream_gene_variant|MODIFIER|LOC_Os01g68460|LOC_Os01g68460|transcript|LOC_Os01g68460.1|protein_coding||c.-2936T>C|||||2936|</td></tr>
<tr><td>C|upstream_gene_variant|MODIFIER|LOC_Os01g68460|LOC_Os01g68460|transcript|LOC_Os01g68460.1|protein_coding||c.-3018A>G|||||3018|</td></tr>
<tr><td>A|upstream_gene_variant|MODIFIER|LOC_Os01g68460|LOC_Os01g68460|transcript|LOC_Os01g68460.1|protein_coding||c.-3140C>T|||||3140|</td></tr>
<tr><td>G|upstream_gene_variant|MODIFIER|LOC_Os01g68460|LOC_Os01g68460|transcript|LOC_Os01g68460.1|protein_coding||c.-3222T>C|||||3222|</td></tr>
<tr><td>C|upstream_gene_variant|MODIFIER|LOC_Os01g68460|LOC_Os01g68460|transcript|LOC_Os01g68460.1|protein_coding||c.-3228A>G|||||3228|</td></tr>
<tr><td>A|upstream_gene_variant|MODIFIER|LOC_Os01g68460|LOC_Os01g68460|transcript|LOC_Os01g68460.1|protein_coding||c.-3302delA|||||3302|</td></tr>
<tr><td>A|upstream_gene_variant|MODIFIER|LOC_Os01g68460|LOC_Os01g68460|transcript|LOC_Os01g68460.1|protein_coding||c.-3342A>T|||||3342|</td></tr>
<tr><td>A|upstream_gene_variant|MODIFIER|LOC_Os01g68460|LOC_Os01g68460|transcript|LOC_Os01g68460.1|protein_coding||c.-3346C>T|||||3346|</td></tr>
<tr><td>GTATA|upstream_gene_variant|MODIFIER|LOC_Os01g68460|LOC_Os01g68460|transcript|LOC_Os01g68460.1|protein_coding||c.-3555_-3554insTATA|||||3555|</td></tr>
<tr><td>G|upstream_gene_variant|MODIFIER|LOC_Os01g68460|LOC_Os01g68460|transcript|LOC_Os01g68460.1|protein_coding||c.-3655T>C|||||3655|</td></tr>
<tr><td>T|upstream_gene_variant|MODIFIER|LOC_Os01g68460|LOC_Os01g68460|transcript|LOC_Os01g68460.1|protein_coding||c.-3815G>A|||||3815|</td></tr>
<tr><td>C|upstream_gene_variant|MODIFIER|LOC_Os01g68460|LOC_Os01g68460|transcript|LOC_Os01g68460.1|protein_coding||c.-3869A>G|||||3869|</td></tr>
<tr><td>T|upstream_gene_variant|MODIFIER|LOC_Os01g68460|LOC_Os01g68460|transcript|LOC_Os01g68460.1|protein_coding||c.-3884G>A|||||3884|</td></tr>
<tr><td>C|upstream_gene_variant|MODIFIER|LOC_Os01g68460|LOC_Os01g68460|transcript|LOC_Os01g68460.1|protein_coding||c.-3893A>G|||||3893|</td></tr>
<tr><td>G|upstream_gene_variant|MODIFIER|LOC_Os01g68460|LOC_Os01g68460|transcript|LOC_Os01g68460.1|protein_coding||c.-3914T>C|||||3914|</td></tr>
<tr><td>T|upstream_gene_variant|MODIFIER|LOC_Os01g68460|LOC_Os01g68460|transcript|LOC_Os01g68460.1|protein_coding||c.-3918G>A|||||3918|</td></tr>
<tr><td>A|upstream_gene_variant|MODIFIER|LOC_Os01g68460|LOC_Os01g68460|transcript|LOC_Os01g68460.1|protein_coding||c.-3937C>T|||||3937|</td></tr>
<tr><td>T|upstream_gene_variant|MODIFIER|LOC_Os01g68460|LOC_Os01g68460|transcript|LOC_Os01g68460.1|protein_coding||c.-3951G>A|||||3951|</td></tr>
<tr><td>CTAT|upstream_gene_variant|MODIFIER|LOC_Os01g68460|LOC_Os01g68460|transcript|LOC_Os01g68460.1|protein_coding||c.-3960_-3959insATA|||||3960|</td></tr>
<tr><td>A|upstream_gene_variant|MODIFIER|LOC_Os01g68460|LOC_Os01g68460|transcript|LOC_Os01g68460.1|protein_coding||c.-3982C>T|||||3982|</td></tr>
<tr><td>A|upstream_gene_variant|MODIFIER|LOC_Os01g68460|LOC_Os01g68460|transcript|LOC_Os01g68460.1|protein_coding||c.-3991C>T|||||3991|</td></tr>
<tr><td>G|upstream_gene_variant|MODIFIER|LOC_Os01g68460|LOC_Os01g68460|transcript|LOC_Os01g68460.1|protein_coding||c.-4002T>C|||||4002|</td></tr>
<tr><td>C|upstream_gene_variant|MODIFIER|LOC_Os01g68460|LOC_Os01g68460|transcript|LOC_Os01g68460.1|protein_coding||c.-4006A>G|||||4006|,TG|upstream_gene_variant|MODIFIER|LOC_Os01g68460|LOC_Os01g68460|transcript|LOC_Os01g68460.1|protein_coding||c.-4007_-4006insC|||||4007|</td></tr>
<tr><td>A|upstream_gene_variant|MODIFIER|LOC_Os01g68460|LOC_Os01g68460|transcript|LOC_Os01g68460.1|protein_coding||c.-4010C>T|||||4010|</td></tr>
<tr><td>G|upstream_gene_variant|MODIFIER|LOC_Os01g68460|LOC_Os01g68460|transcript|LOC_Os01g68460.1|protein_coding||c.-4041T>C|||||4041|</td></tr>
<tr><td>A|upstream_gene_variant|MODIFIER|LOC_Os01g68460|LOC_Os01g68460|transcript|LOC_Os01g68460.1|protein_coding||c.-4070delA|||||4070|</td></tr>
<tr><td>T|upstream_gene_variant|MODIFIER|LOC_Os01g68460|LOC_Os01g68460|transcript|LOC_Os01g68460.1|protein_coding||c.-4086G>A|||||4086|</td></tr>
<tr><td>A|upstream_gene_variant|MODIFIER|LOC_Os01g68460|LOC_Os01g68460|transcript|LOC_Os01g68460.1|protein_coding||c.-4093C>T|||||4093|</td></tr>
<tr><td>T|upstream_gene_variant|MODIFIER|LOC_Os01g68460|LOC_Os01g68460|transcript|LOC_Os01g68460.1|protein_coding||c.-4385G>A|||||4385|</td></tr>
<tr><td>T|upstream_gene_variant|MODIFIER|LOC_Os01g68460|LOC_Os01g68460|transcript|LOC_Os01g68460.1|protein_coding||c.-4426G>A|||||4426|</td></tr>
<tr><td>C|upstream_gene_variant|MODIFIER|LOC_Os01g68460|LOC_Os01g68460|transcript|LOC_Os01g68460.1|protein_coding||c.-4462A>G|||||4462|</td></tr>
<tr><td>A|upstream_gene_variant|MODIFIER|LOC_Os01g68460|LOC_Os01g68460|transcript|LOC_Os01g68460.1|protein_coding||c.-4500C>T|||||4500|</td></tr>
</table>

<table>
<tr><td>A|upstream_gene_variant|MODIFIER|LOC_Os01g68460|LOC_Os01g68460|transcript|LOC_Os01g68460.1|protein_coding||c.-4620A>T|||||4620|</td></tr>
<tr><td>T|upstream_gene_variant|MODIFIER|LOC_Os01g68460|LOC_Os01g68460|transcript|LOC_Os01g68460.1|protein_coding||c.-4666G>A|||||4666|</td></tr>
<tr><td>G|upstream_gene_variant|MODIFIER|LOC_Os01g68460|LOC_Os01g68460|transcript|LOC_Os01g68460.1|protein_coding||c.-4693T>C|||||4693|</td></tr>
<tr><td>T|upstream_gene_variant|MODIFIER|LOC_Os01g68460|LOC_Os01g68460|transcript|LOC_Os01g68460.1|protein_coding||c.-4742G>A|||||4742|</td></tr>
<tr><td>A|upstream_gene_variant|MODIFIER|LOC_Os01g68460|LOC_Os01g68460|transcript|LOC_Os01g68460.1|protein_coding||c.-4760C>T|||||4760|</td></tr>
<tr><td>A|upstream_gene_variant|MODIFIER|LOC_Os01g68460|LOC_Os01g68460|transcript|LOC_Os01g68460.1|protein_coding||c.-4779C>T|||||4779|</td></tr>
<tr><td>A|upstream_gene_variant|MODIFIER|LOC_Os01g68460|LOC_Os01g68460|transcript|LOC_Os01g68460.1|protein_coding||c.-4794C>T|||||4794|</td></tr>
<tr><td>A|upstream_gene_variant|MODIFIER|LOC_Os01g68460|LOC_Os01g68460|transcript|LOC_Os01g68460.1|protein_coding||c.-4798C>T|||||4798|</td></tr>
<tr><td>A|upstream_gene_variant|MODIFIER|LOC_Os01g68460|LOC_Os01g68460|transcript|LOC_Os01g68460.1|protein_coding||c.-4804C>T|||||4804|</td></tr>
<tr><td>A|upstream_gene_variant|MODIFIER|LOC_Os01g68460|LOC_Os01g68460|transcript|LOC_Os01g68460.1|protein_coding||c.-4827C>T|||||4827|</td></tr>
<tr><td>G|upstream_gene_variant|MODIFIER|LOC_Os01g68460|LOC_Os01g68460|transcript|LOC_Os01g68460.1|protein_coding||c.-4856T>C|||||4856|</td></tr>
<tr><td>A|upstream_gene_variant|MODIFIER|LOC_Os01g68460|LOC_Os01g68460|transcript|LOC_Os01g68460.1|protein_coding||c.-4859C>T|||||4859|</td></tr>
<tr><td>T|upstream_gene_variant|MODIFIER|LOC_Os01g68460|LOC_Os01g68460|transcript|LOC_Os01g68460.1|protein_coding||c.-4897G>A|||||4897|</td></tr>
<tr><td>C|upstream_gene_variant|MODIFIER|LOC_Os01g68460|LOC_Os01g68460|transcript|LOC_Os01g68460.1|protein_coding||c.-4909A>G|||||4909|</td></tr>
<tr><td>T|upstream_gene_variant|MODIFIER|LOC_Os01g68460|LOC_Os01g68460|transcript|LOC_Os01g68460.1|protein_coding||c.-4912G>A|||||4912|</td></tr>
<tr><td>TA|upstream_gene_variant|MODIFIER|LOC_Os01g68460|LOC_Os01g68460|transcript|LOC_Os01g68460.1|protein_coding||c.-4930_-4929insT|||||4930|,TAA|upstream_gene_variant|MODIFIER|LOC_Os01g68460|LOC_Os01g68460|transcript|LOC_Os01g68460.1|protein_coding||c.-4930_-4929insTT|||||4930|</td></tr>
<tr><td>A|upstream_gene_variant|MODIFIER|LOC_Os01g68460|LOC_Os01g68460|transcript|LOC_Os01g68460.1|protein_coding||c.-4997C>T|||||4997|</td></tr>
<tr><td></td></tr>
<tr><td></td></tr>
<tr><td>A|3_prime_UTR_variant|MODIFIER|LOC_Os01g68500|LOC_Os01g68500|transcript|LOC_Os01g68500.1|protein_coding|2/2|c.*248C>T|||||248|</td></tr>
<tr><td>A|missense_variant|MODERATE|LOC_Os01g68500|LOC_Os01g68500|transcript|LOC_Os01g68500.1|protein_coding|2/2|c.505G>T|p.Ala169Ser|638/967|505/516|169/171||</td></tr>
<tr><td>T|upstream_gene_variant|MODIFIER|LOC_Os01g68490|LOC_Os01g68490|transcript|LOC_Os01g68490.1|protein_coding||c.-3416delC|||||2550|</td></tr>
<tr><td>A|synonymous_variant|LOW|LOC_Os01g68500|LOC_Os01g68500|transcript|LOC_Os01g68500.1|protein_coding|1/2|c.297C>T|p.Gly99Gly|430/967|297/516|99/171||</td></tr>
<tr><td>T|missense_variant|MODERATE|LOC_Os01g68500|LOC_Os01g68500|transcript|LOC_Os01g68500.1|protein_coding|1/2|c.88G>A|p.Ala30Thr|221/967|88/516|30/171||</td></tr>
<tr><td>GCCCC|frameshift_variant|HIGH|LOC_Os01g68500|LOC_Os01g68500|transcript|LOC_Os01g68500.1|protein_coding|1/2|c.73_74insGGGG|p.Ala25fs|206/967|73/516|25/171||,C|missense_variant|MODERATE|LOC_Os01g68500|LOC_Os01g68500|transcript|LOC_Os01g68500.1|protein_coding|1/2|c.74C>G|p.Ala25Gly|207/967|74/516|25/171||,C|upstream_gene_variant|MODIFIER|LOC_Os01g68490|LOC_Os01g68490|transcript|LOC_Os01g68490.1|protein_coding||c.-3836C>G|||||2970|</td></tr>
<tr><td>A|missense_variant|MODERATE|LOC_Os01g68500|LOC_Os01g68500|transcript|LOC_Os01g68500.1|protein_coding|1/2|c.73G>T|p.Ala25Ser|206/967|73/516|25/171||</td></tr>
<tr><td>C|missense_variant|MODERATE|LOC_Os01g68500|LOC_Os01g68500|transcript|LOC_Os01g68500.1|protein_coding|1/2|c.71T>G|p.Val24Gly|204/967|71/516|24/171||</td></tr>
<tr><td>C|synonymous_variant|LOW|LOC_Os01g68500|LOC_Os01g68500|transcript|LOC_Os01g68500.1|protein_coding|1/2|c.66C>G|p.Gly22Gly|199/967|66/516|22/171||</td></tr>
</table>

<table>
<tr><td>C|missense_variant|MODERATE|LOC_Os01g68500|LOC_Os01g68500|transcript|LOC_Os01g68500.1|protein_coding|1/2|c.61T>G|p.Trp21Gly|194/967|61/516|21/171||</td></tr>
<tr><td>C|missense_variant|MODERATE|LOC_Os01g68500|LOC_Os01g68500|transcript|LOC_Os01g68500.1|protein_coding|1/2|c.56C>G|p.Ala19Gly|189/967|56/516|19/171||</td></tr>
<tr><td>C|missense_variant|MODERATE|LOC_Os01g68500|LOC_Os01g68500|transcript|LOC_Os01g68500.1|protein_coding|1/2|c.53C>G|p.Ala18Gly|186/967|53/516|18/171||</td></tr>
<tr><td>C|missense_variant|MODERATE|LOC_Os01g68500|LOC_Os01g68500|transcript|LOC_Os01g68500.1|protein_coding|1/2|c.50C>G|p.Ala17Gly|183/967|50/516|17/171||</td></tr>
<tr><td>C|missense_variant|MODERATE|LOC_Os01g68500|LOC_Os01g68500|transcript|LOC_Os01g68500.1|protein_coding|1/2|c.47T>G|p.Val16Gly|180/967|47/516|16/171||</td></tr>
<tr><td>C|missense_variant|MODERATE|LOC_Os01g68500|LOC_Os01g68500|transcript|LOC_Os01g68500.1|protein_coding|1/2|c.44T>G|p.Val15Gly|177/967|44/516|15/171||</td></tr>
<tr><td>C|frameshift_variant|HIGH|LOC_Os01g68500|LOC_Os01g68500|transcript|LOC_Os01g68500.1|protein_coding|1/2|c.40_41delAT|p.Met14fs|174/967|40/516|14/171||</td></tr>
<tr><td>C|missense_variant|MODERATE|LOC_Os01g68500|LOC_Os01g68500|transcript|LOC_Os01g68500.1|protein_coding|1/2|c.41T>G|p.Met14Arg|174/967|41/516|14/171||</td></tr>
<tr><td>A|missense_variant|MODERATE|LOC_Os01g68500|LOC_Os01g68500|transcript|LOC_Os01g68500.1|protein_coding|1/2|c.40A>T|p.Met14Leu|173/967|40/516|14/171||</td></tr>
<tr><td>A|upstream_gene_variant|MODIFIER|LOC_Os01g68500|LOC_Os01g68500|transcript|LOC_Os01g68500.1|protein_coding||c.-2021C>T|||||1888|</td></tr>
<tr><td>T|upstream_gene_variant|MODIFIER|LOC_Os01g68500|LOC_Os01g68500|transcript|LOC_Os01g68500.1|protein_coding||c.-2131T>A|||||1998|</td></tr>
<tr><td>A|upstream_gene_variant|MODIFIER|LOC_Os01g68500|LOC_Os01g68500|transcript|LOC_Os01g68500.1|protein_coding||c.-2138delC|||||2005|</td></tr>
<tr><td>C|upstream_gene_variant|MODIFIER|LOC_Os01g68500|LOC_Os01g68500|transcript|LOC_Os01g68500.1|protein_coding||c.-2242C>G|||||2109|</td></tr>
<tr><td>A|upstream_gene_variant|MODIFIER|LOC_Os01g68500|LOC_Os01g68500|transcript|LOC_Os01g68500.1|protein_coding||c.-2354C>T|||||2221|</td></tr>
<tr><td>G|upstream_gene_variant|MODIFIER|LOC_Os01g68500|LOC_Os01g68500|transcript|LOC_Os01g68500.1|protein_coding||c.-2378T>C|||||2245|</td></tr>
<tr><td>T|upstream_gene_variant|MODIFIER|LOC_Os01g68500|LOC_Os01g68500|transcript|LOC_Os01g68500.1|protein_coding||c.-2465C>A|||||2332|</td></tr>
<tr><td>C|upstream_gene_variant|MODIFIER|LOC_Os01g68500|LOC_Os01g68500|transcript|LOC_Os01g68500.1|protein_coding||c.-2561T>G|||||2428|</td></tr>
<tr><td>T|upstream_gene_variant|MODIFIER|LOC_Os01g68500|LOC_Os01g68500|transcript|LOC_Os01g68500.1|protein_coding||c.-2754G>A|||||2621|</td></tr>
<tr><td>GT|upstream_gene_variant|MODIFIER|LOC_Os01g68500|LOC_Os01g68500|transcript|LOC_Os01g68500.1|protein_coding||c.-2757_-2756insA|||||2624|</td></tr>
<tr><td>TTC|upstream_gene_variant|MODIFIER|LOC_Os01g68500|LOC_Os01g68500|transcript|LOC_Os01g68500.1|protein_coding||c.-2761_-2760insGA|||||2628|</td></tr>
<tr><td>T|upstream_gene_variant|MODIFIER|LOC_Os01g68500|LOC_Os01g68500|transcript|LOC_Os01g68500.1|protein_coding||c.-2841G>A|||||2708|</td></tr>
<tr><td>G|upstream_gene_variant|MODIFIER|LOC_Os01g68500|LOC_Os01g68500|transcript|LOC_Os01g68500.1|protein_coding||c.-2857_-2852delTGTTTT|||||2719|,GA|upstream_gene_variant|MODIFIER|LOC_Os01g68500|LOC_Os01g68500|transcript|LOC_Os01g68500.1|protein_coding||c.-2857_-2853delTGTTT|||||2720|</td></tr>
<tr><td>A|upstream_gene_variant|MODIFIER|LOC_Os01g68500|LOC_Os01g68500|transcript|LOC_Os01g68500.1|protein_coding||c.-2856_-2853delGTTT|||||2720|</td></tr>
<tr><td>A|upstream_gene_variant|MODIFIER|LOC_Os01g68500|LOC_Os01g68500|transcript|LOC_Os01g68500.1|protein_coding||c.-2856_-2854delGTT|||||2721|</td></tr>
</table>

A|upstream_gene_variant|MODIFIER|LOC_Os01g68500|LOC_Os01g68500|transcript|LOC_Os01g68500.1|protein_coding||c.-2856_-2855delGT|||||2722|

C|upstream_gene_variant|MODIFIER|LOC_Os01g68500|LOC_Os01g68500|transcript|LOC_Os01g68500.1|protein_coding||c.-2857delT|||||2724|,CAA|upstream_gene_variant|MODIFIER|LOC_Os01g68500|LOC_Os01g68500|transcript|LOC_Os01g68500.1|protein_coding||c.-2858_-2857insT|||||2725|,CAAA|upstream_gene_variant|MODIFIER|LOC_Os01g68500|LOC_Os01g68500|transcript|LOC_Os01g68500.1|protein_coding||c.-2858_-2857insTT|||||2725|

A|upstream_gene_variant|MODIFIER|LOC_Os01g68500|LOC_Os01g68500|transcript|LOC_Os01g68500.1|protein_coding||c.-2868A>T|||||2735|

G|upstream_gene_variant|MODIFIER|LOC_Os01g68500|LOC_Os01g68500|transcript|LOC_Os01g68500.1|protein_coding||c.-2871T>C|||||2738|

A|upstream_gene_variant|MODIFIER|LOC_Os01g68500|LOC_Os01g68500|transcript|LOC_Os01g68500.1|protein_coding||c.-2924C>T|||||2791|

T|upstream_gene_variant|MODIFIER|LOC_Os01g68500|LOC_Os01g68500|transcript|LOC_Os01g68500.1|protein_coding||c.-2930G>A|||||2797|

A|upstream_gene_variant|MODIFIER|LOC_Os01g68500|LOC_Os01g68500|transcript|LOC_Os01g68500.1|protein_coding||c.-2950C>T|||||2817|

G|upstream_gene_variant|MODIFIER|LOC_Os01g68500|LOC_Os01g68500|transcript|LOC_Os01g68500.1|protein_coding||c.-2957T>C|||||2824|

TTC|upstream_gene_variant|MODIFIER|LOC_Os01g68500|LOC_Os01g68500|transcript|LOC_Os01g68500.1|protein_coding||c.-2985_-2984insGA|||||2852|

CTT|upstream_gene_variant|MODIFIER|LOC_Os01g68500|LOC_Os01g68500|transcript|LOC_Os01g68500.1|protein_coding||c.-2987_-2986insAA|||||2854|,CTTT|upstream_gene_variant|MODIFIER|LOC_Os01g68500|LOC_Os01g68500|transcript|LOC_Os01g68500.1|protein_coding||c.-2987_-2986insAAA|||||2854|

T|upstream_gene_variant|MODIFIER|LOC_Os01g68500|LOC_Os01g68500|transcript|LOC_Os01g68500.1|protein_coding||c.-3001G>A|||||2868|

G|upstream_gene_variant|MODIFIER|LOC_Os01g68500|LOC_Os01g68500|transcript|LOC_Os01g68500.1|protein_coding||c.-3004G>C|||||2871|

G|upstream_gene_variant|MODIFIER|LOC_Os01g68500|LOC_Os01g68500|transcript|LOC_Os01g68500.1|protein_coding||c.-3016T>C|||||2883|

A|upstream_gene_variant|MODIFIER|LOC_Os01g68500|LOC_Os01g68500|transcript|LOC_Os01g68500.1|protein_coding||c.-3036C>T|||||2903|

C|upstream_gene_variant|MODIFIER|LOC_Os01g68500|LOC_Os01g68500|transcript|LOC_Os01g68500.1|protein_coding||c.-3042A>G|||||2909|

A|upstream_gene_variant|MODIFIER|LOC_Os01g68500|LOC_Os01g68500|transcript|LOC_Os01g68500.1|protein_coding||c.-3055C>T|||||2922|

T|upstream_gene_variant|MODIFIER|LOC_Os01g68500|LOC_Os01g68500|transcript|LOC_Os01g68500.1|protein_coding||c.-3058G>A|||||2925|

A|upstream_gene_variant|MODIFIER|LOC_Os01g68500|LOC_Os01g68500|transcript|LOC_Os01g68500.1|protein_coding||c.-3077C>T|||||2944|

T|upstream_gene_variant|MODIFIER|LOC_Os01g68500|LOC_Os01g68500|transcript|LOC_Os01g68500.1|protein_coding||c.-3087_-3082delTAGCGT|||||2949|

A|upstream_gene_variant|MODIFIER|LOC_Os01g68500|LOC_Os01g68500|transcript|LOC_Os01g68500.1|protein_coding||c.-3092G>T|||||2959|

A|upstream_gene_variant|MODIFIER|LOC_Os01g68500|LOC_Os01g68500|transcript|LOC_Os01g68500.1|protein_coding||c.-3110C>T|||||2977|

A|upstream_gene_variant|MODIFIER|LOC_Os01g68500|LOC_Os01g68500|transcript|LOC_Os01g68500.1|protein_coding||c.-3118C>T|||||2985|

C|upstream_gene_variant|MODIFIER|LOC_Os01g68500|LOC_Os01g68500|transcript|LOC_Os01g68500.1|protein_coding||c.-3169C>G|||||3036|

T|upstream_gene_variant|MODIFIER|LOC_Os01g68500|LOC_Os01g68500|transcript|LOC_Os01g68500.1|protein_coding||c.-3198G>A|||||3065|

T|upstream_gene_variant|MODIFIER|LOC_Os01g68500|LOC_Os01g68500|transcript|LOC_Os01g68500.1|protein_coding||c.-3199G>A|||||3066|

T|upstream_gene_variant|MODIFIER|LOC_Os01g68500|LOC_Os01g68500|transcript|LOC_Os01g68500.1|protein_coding||c.-3244G>A|||||3111|

A|upstream_gene_variant|MODIFIER|LOC_Os01g68500|LOC_Os01g68500|transcript|LOC_Os01g68500.1|protein_coding||c.-3259C>T|||||3126|

C|upstream_gene_variant|MODIFIER|LOC_Os01g68500|LOC_Os01g68500|transcript|LOC_Os01g68500.1|protein_coding||c.-3262A>G|||||3129|

G|upstream_gene_variant|MODIFIER|LOC_Os01g68500|LOC_Os01g68500|transcript|LOC_Os01g68500.1|protein_coding||c.-3281G>C|||||3148|

C|upstream_gene_variant|MODIFIER|LOC_Os01g68500|LOC_Os01g68500|transcript|LOC_Os01g68500.1|protein_coding||c.-3302A>G|||||3169|

<table>
<tr><td>A|upstream_gene_variant|MODIFIER|LOC_Os01g68500|LOC_Os01g68500|transcript|LOC_Os01g68500.1|protein_coding||c.-3317G>T|||||3184|</td></tr>
<tr><td>A|upstream_gene_variant|MODIFIER|LOC_Os01g68500|LOC_Os01g68500|transcript|LOC_Os01g68500.1|protein_coding||c.-3346C>T|||||3213|</td></tr>
<tr><td>T|upstream_gene_variant|MODIFIER|LOC_Os01g68500|LOC_Os01g68500|transcript|LOC_Os01g68500.1|protein_coding||c.-3348G>A|||||3215|</td></tr>
<tr><td>A|upstream_gene_variant|MODIFIER|LOC_Os01g68500|LOC_Os01g68500|transcript|LOC_Os01g68500.1|protein_coding||c.-3349C>T|||||3216|</td></tr>
<tr><td>T|upstream_gene_variant|MODIFIER|LOC_Os01g68500|LOC_Os01g68500|transcript|LOC_Os01g68500.1|protein_coding||c.-3355G>A|||||3222|</td></tr>
<tr><td>C|upstream_gene_variant|MODIFIER|LOC_Os01g68500|LOC_Os01g68500|transcript|LOC_Os01g68500.1|protein_coding||c.-3360A>G|||||3227|</td></tr>
<tr><td>T|upstream_gene_variant|MODIFIER|LOC_Os01g68500|LOC_Os01g68500|transcript|LOC_Os01g68500.1|protein_coding||c.-3378G>A|||||3245|</td></tr>
<tr><td>C|upstream_gene_variant|MODIFIER|LOC_Os01g68500|LOC_Os01g68500|transcript|LOC_Os01g68500.1|protein_coding||c.-3387A>G|||||3254|</td></tr>
<tr><td>C|upstream_gene_variant|MODIFIER|LOC_Os01g68500|LOC_Os01g68500|transcript|LOC_Os01g68500.1|protein_coding||c.-3398C>G|||||3265|</td></tr>
<tr><td>C|upstream_gene_variant|MODIFIER|LOC_Os01g68500|LOC_Os01g68500|transcript|LOC_Os01g68500.1|protein_coding||c.-3436delC|||||3303|</td></tr>
<tr><td>C|upstream_gene_variant|MODIFIER|LOC_Os01g68500|LOC_Os01g68500|transcript|LOC_Os01g68500.1|protein_coding||c.-3463A>G|||||3330|</td></tr>
<tr><td>C|upstream_gene_variant|MODIFIER|LOC_Os01g68500|LOC_Os01g68500|transcript|LOC_Os01g68500.1|protein_coding||c.-3519A>G|||||3386|</td></tr>
<tr><td>T|upstream_gene_variant|MODIFIER|LOC_Os01g68500|LOC_Os01g68500|transcript|LOC_Os01g68500.1|protein_coding||c.-3526delT|||||3393|,TAA|upstream_gene_variant|MODIFIER|LOC_Os01g68500|LOC_Os01g68500|transcript|LOC_Os01g68500.1|protein_coding||c.-3527_-3526insT|||||3394|</td></tr>
<tr><td>C|upstream_gene_variant|MODIFIER|LOC_Os01g68500|LOC_Os01g68500|transcript|LOC_Os01g68500.1|protein_coding||c.-3546A>G|||||3413|</td></tr>
<tr><td>C|upstream_gene_variant|MODIFIER|LOC_Os01g68500|LOC_Os01g68500|transcript|LOC_Os01g68500.1|protein_coding||c.-3572A>G|||||3439|</td></tr>
<tr><td>C|upstream_gene_variant|MODIFIER|LOC_Os01g68500|LOC_Os01g68500|transcript|LOC_Os01g68500.1|protein_coding||c.-3577_-3576delTG|||||3443|</td></tr>
<tr><td>T|upstream_gene_variant|MODIFIER|LOC_Os01g68500|LOC_Os01g68500|transcript|LOC_Os01g68500.1|protein_coding||c.-3583G>A|||||3450|</td></tr>
<tr><td>G|upstream_gene_variant|MODIFIER|LOC_Os01g68500|LOC_Os01g68500|transcript|LOC_Os01g68500.1|protein_coding||c.-3600T>C|||||3467|</td></tr>
<tr><td>C|upstream_gene_variant|MODIFIER|LOC_Os01g68500|LOC_Os01g68500|transcript|LOC_Os01g68500.1|protein_coding||c.-3607A>G|||||3474|</td></tr>
<tr><td>T|upstream_gene_variant|MODIFIER|LOC_Os01g68500|LOC_Os01g68500|transcript|LOC_Os01g68500.1|protein_coding||c.-3608G>A|||||3475|</td></tr>
<tr><td>T|upstream_gene_variant|MODIFIER|LOC_Os01g68500|LOC_Os01g68500|transcript|LOC_Os01g68500.1|protein_coding||c.-3614G>A|||||3481|</td></tr>
<tr><td>T|upstream_gene_variant|MODIFIER|LOC_Os01g68500|LOC_Os01g68500|transcript|LOC_Os01g68500.1|protein_coding||c.-3633G>A|||||3500|</td></tr>
<tr><td>C|upstream_gene_variant|MODIFIER|LOC_Os01g68500|LOC_Os01g68500|transcript|LOC_Os01g68500.1|protein_coding||c.-3639A>G|||||3506|</td></tr>
<tr><td>A|upstream_gene_variant|MODIFIER|LOC_Os01g68500|LOC_Os01g68500|transcript|LOC_Os01g68500.1|protein_coding||c.-3648C>T|||||3515|</td></tr>
<tr><td>A|upstream_gene_variant|MODIFIER|LOC_Os01g68500|LOC_Os01g68500|transcript|LOC_Os01g68500.1|protein_coding||c.-3652C>T|||||3519|</td></tr>
<tr><td>G|upstream_gene_variant|MODIFIER|LOC_Os01g68500|LOC_Os01g68500|transcript|LOC_Os01g68500.1|protein_coding||c.-3656T>C|||||3523|</td></tr>
<tr><td>A|upstream_gene_variant|MODIFIER|LOC_Os01g68500|LOC_Os01g68500|transcript|LOC_Os01g68500.1|protein_coding||c.-3665C>T|||||3532|</td></tr>
<tr><td>T|upstream_gene_variant|MODIFIER|LOC_Os01g68500|LOC_Os01g68500|transcript|LOC_Os01g68500.1|protein_coding||c.-3670T>A|||||3537|</td></tr>
<tr><td>C|upstream_gene_variant|MODIFIER|LOC_Os01g68500|LOC_Os01g68500|transcript|LOC_Os01g68500.1|protein_coding||c.-3676T>G|||||3543|</td></tr>
<tr><td>A|upstream_gene_variant|MODIFIER|LOC_Os01g68500|LOC_Os01g68500|transcript|LOC_Os01g68500.1|protein_coding||c.-3681C>T|||||3548|</td></tr>
<tr><td>CGGA|upstream_gene_variant|MODIFIER|LOC_Os01g68500|LOC_Os01g68500|transcript|LOC_Os01g68500.1|protein_coding||c.-3686_-3685insTCC|||||3553|</td></tr>
<tr><td>A|upstream_gene_variant|MODIFIER|LOC_Os01g68500|LOC_Os01g68500|transcript|LOC_Os01g68500.1|protein_coding||c.-3874C>T|||||3741|</td></tr>
<tr><td>A|upstream_gene_variant|MODIFIER|LOC_Os01g68500|LOC_Os01g68500|transcript|LOC_Os01g68500.1|protein_coding||c.-3912G>T|||||3779|</td></tr>
<tr><td>T|upstream_gene_variant|MODIFIER|LOC_Os01g68500|LOC_Os01g68500|transcript|LOC_Os01g68500.1|protein_coding||c.-3939G>A|||||3806|</td></tr>
<tr><td>G|upstream_gene_variant|MODIFIER|LOC_Os01g68500|LOC_Os01g68500|transcript|LOC_Os01g68500.1|protein_coding||c.-3981T>C|||||3848|</td></tr>
</table>

A|upstream_gene_variant|MODIFIER|LOC_Os01g68500|LOC_Os01g68500|transcript|LOC_Os01g68500.1|protein_coding||c.-4135C>T|||||4002|

GT|upstream_gene_variant|MODIFIER|LOC_Os01g68500|LOC_Os01g68500|transcript|LOC_Os01g68500.1|protein_coding||c.-4342_-4341insA|||||4209|,GTT|upstream_gene_variant|MODIFIER|LOC_Os01g68500|LOC_Os01g68500|transcript|LOC_Os01g68500.1|protein_coding||c.-4342_-4341insAA|||||4209|,GTTT|upstream_gene_variant|MODIFIER|LOC_Os01g68500|LOC_Os01g68500|transcript|LOC_Os01g68500.1|protein_coding||c.-4342_-4341insAAA|||||4209|,GTTTT|upstream_gene_variant|MODIFIER|LOC_Os01g68500|LOC_Os01g68500|transcript|LOC_Os01g68500.1|protein_coding||c.-4342_-4341insAAAA|||||4209|,GTTTTT|upstream_gene_variant|MODIFIER|LOC_Os01g68500|LOC_Os01g68500|transcript|LOC_Os01g68500.1|protein_coding||c.-4342_-4341insAAAAA|||||4209|

TTC|upstream_gene_variant|MODIFIER|LOC_Os01g68500|LOC_Os01g68500|transcript|LOC_Os01g68500.1|protein_coding||c.-4343_-4342insGA|||||4210|

A|upstream_gene_variant|MODIFIER|LOC_Os01g68500|LOC_Os01g68500|transcript|LOC_Os01g68500.1|protein_coding||c.-4520C>T|||||4387|

G|upstream_gene_variant|MODIFIER|LOC_Os01g68500|LOC_Os01g68500|transcript|LOC_Os01g68500.1|protein_coding||c.-4536T>C|||||4403|

C|upstream_gene_variant|MODIFIER|LOC_Os01g68500|LOC_Os01g68500|transcript|LOC_Os01g68500.1|protein_coding||c.-4537C>G|||||4404|

T|upstream_gene_variant|MODIFIER|LOC_Os01g68500|LOC_Os01g68500|transcript|LOC_Os01g68500.1|protein_coding||c.-4542C>A|||||4409|

A|upstream_gene_variant|MODIFIER|LOC_Os01g68500|LOC_Os01g68500|transcript|LOC_Os01g68500.1|protein_coding||c.-4701G>T|||||4568|

T|upstream_gene_variant|MODIFIER|LOC_Os01g68500|LOC_Os01g68500|transcript|LOC_Os01g68500.1|protein_coding||c.-4703C>A|||||4570|

CT|upstream_gene_variant|MODIFIER|LOC_Os01g68500|LOC_Os01g68500|transcript|LOC_Os01g68500.1|protein_coding||c.-4707_-4706insA|||||4574|

AA|upstream_gene_variant|MODIFIER|LOC_Os01g68500|LOC_Os01g68500|transcript|LOC_Os01g68500.1|protein_coding||c.-4707A>T|||||4574|,TTAA|upstream_gene_variant|MODIFIER|LOC_Os01g68500|LOC_Os01g68500|transcript|LOC_Os01g68500.1|protein_coding||c.-4708_-4707insTA|||||4575|,TTAAA|upstream_gene_variant|MODIFIER|LOC_Os01g68500|LOC_Os01g68500|transcript|LOC_Os01g68500.1|protein_coding||c.-4708_-4707insTTA|||||4575|,T|upstream_gene_variant|MODIFIER|LOC_Os01g68500|LOC_Os01g68500|transcript|LOC_Os01g68500.1|protein_coding||c.-4708delT|||||4575|

T|upstream_gene_variant|MODIFIER|LOC_Os01g68500|LOC_Os01g68500|transcript|LOC_Os01g68500.1|protein_coding||c.-4708T>A|||||4575|

AAT|upstream_gene_variant|MODIFIER|LOC_Os01g68500|LOC_Os01g68500|transcript|LOC_Os01g68500.1|protein_coding||c.-4716_-4715insAT|||||4583|,AC|upstream_gene_variant|MODIFIER|LOC_Os01g68500|LOC_Os01g68500|transcript|LOC_Os01g68500.1|protein_coding||c.-4716_-4715insG|||||4583|

A|upstream_gene_variant|MODIFIER|LOC_Os01g68500|LOC_Os01g68500|transcript|LOC_Os01g68500.1|protein_coding||c.-4717A>T|||||4584|

A|upstream_gene_variant|MODIFIER|LOC_Os01g68500|LOC_Os01g68500|transcript|LOC_Os01g68500.1|protein_coding||c.-4719G>T|||||4586|

A|upstream_gene_variant|MODIFIER|LOC_Os01g68500|LOC_Os01g68500|transcript|LOC_Os01g68500.1|protein_coding||c.-4721C>T|||||4588|

T|upstream_gene_variant|MODIFIER|LOC_Os01g68500|LOC_Os01g68500|transcript|LOC_Os01g68500.1|protein_coding||c.-4726G>A|||||4593|

A|upstream_gene_variant|MODIFIER|LOC_Os01g68500|LOC_Os01g68500|transcript|LOC_Os01g68500.1|protein_coding||c.-4730C>T|||||4597|

T|upstream_gene_variant|MODIFIER|LOC_Os01g68500|LOC_Os01g68500|transcript|LOC_Os01g68500.1|protein_coding||c.-4734G>A|||||4601|

T|upstream_gene_variant|MODIFIER|LOC_Os01g68500|LOC_Os01g68500|transcript|LOC_Os01g68500.1|protein_coding||c.-4743G>A|||||4610|

C|upstream_gene_variant|MODIFIER|LOC_Os01g68500|LOC_Os01g68500|transcript|LOC_Os01g68500.1|protein_coding||c.-4762A>G|||||4629|

<table>
<tr><td>T|upstream_gene_variant|MODIFIER|LOC_Os01g68500|LOC_Os01g68500|transcript|LOC_Os01g68500.1|protein_coding||c.-4766C>A|||||4633|</td></tr>
<tr><td>A|upstream_gene_variant|MODIFIER|LOC_Os01g68500|LOC_Os01g68500|transcript|LOC_Os01g68500.1|protein_coding||c.-4774G>T|||||4641|</td></tr>
<tr><td>C|upstream_gene_variant|MODIFIER|LOC_Os01g68500|LOC_Os01g68500|transcript|LOC_Os01g68500.1|protein_coding||c.-4786T>G|||||4653|,T|upstream_gene_variant|MODIFIER|LOC_Os01g68500|LOC_Os01g68500|transcript|LOC_Os01g68500.1|protein_coding||c.-4786T>A|||||4653|</td></tr>
<tr><td>A|upstream_gene_variant|MODIFIER|LOC_Os01g68500|LOC_Os01g68500|transcript|LOC_Os01g68500.1|protein_coding||c.-4789C>T|||||4656|</td></tr>
<tr><td>T|upstream_gene_variant|MODIFIER|LOC_Os01g68500|LOC_Os01g68500|transcript|LOC_Os01g68500.1|protein_coding||c.-4809delT|||||4676|,TAA|upstream_gene_variant|MODIFIER|LOC_Os01g68500|LOC_Os01g68500|transcript|LOC_Os01g68500.1|protein_coding||c.-4810_-4809insT|||||4677|</td></tr>
<tr><td>A|upstream_gene_variant|MODIFIER|LOC_Os01g68500|LOC_Os01g68500|transcript|LOC_Os01g68500.1|protein_coding||c.-4817G>T|||||4684|</td></tr>
<tr><td>T|upstream_gene_variant|MODIFIER|LOC_Os01g68500|LOC_Os01g68500|transcript|LOC_Os01g68500.1|protein_coding||c.-4828G>A|||||4695|,CTTTT|upstream_gene_variant|MODIFIER|LOC_Os01g68500|LOC_Os01g68500|transcript|LOC_Os01g68500.1|protein_coding||c.-4829_-4828insAAAA|||||4696|</td></tr>
<tr><td>G|upstream_gene_variant|MODIFIER|LOC_Os01g68500|LOC_Os01g68500|transcript|LOC_Os01g68500.1|protein_coding||c.-4857T>C|||||4724|</td></tr>
<tr><td>A|upstream_gene_variant|MODIFIER|LOC_Os01g68500|LOC_Os01g68500|transcript|LOC_Os01g68500.1|protein_coding||c.-4860A>T|||||4727|</td></tr>
<tr><td>C|upstream_gene_variant|MODIFIER|LOC_Os01g68500|LOC_Os01g68500|transcript|LOC_Os01g68500.1|protein_coding||c.-4864T>G|||||4731|</td></tr>
<tr><td>CTATTCAGATTCATTA|upstream_gene_variant|MODIFIER|LOC_Os01g68500|LOC_Os01g68500|transcript|LOC_Os01g68500.1|protein_coding||c.-4875_-4874insTAATGAATCTGAATA|||||4742|,CTATTCAGATTCGTTG|upstream_gene_variant|MODIFIER|LOC_Os01g68500|LOC_Os01g68500|transcript|LOC_Os01g68500.1|protein_coding||c.-4875_-4874insCAACGAATCTGAATA|||||4742|,CTATTCAGATTTGTTG|upstream_gene_variant|MODIFIER|LOC_Os01g68500|LOC_Os01g68500|transcript|LOC_Os01g68500.1|protein_coding||c.-4875_-4874insCAACAAATCTGAATA|||||4742|</td></tr>
<tr><td>C|upstream_gene_variant|MODIFIER|LOC_Os01g68500|LOC_Os01g68500|transcript|LOC_Os01g68500.1|protein_coding||c.-4881A>G|||||4748|</td></tr>
<tr><td>A|upstream_gene_variant|MODIFIER|LOC_Os01g68500|LOC_Os01g68500|transcript|LOC_Os01g68500.1|protein_coding||c.-4891C>T|||||4758|</td></tr>
<tr><td>AT|upstream_gene_variant|MODIFIER|LOC_Os01g68500|LOC_Os01g68500|transcript|LOC_Os01g68500.1|protein_coding||c.-4904_-4903insA|||||4771|</td></tr>
<tr><td>A|upstream_gene_variant|MODIFIER|LOC_Os01g68500|LOC_Os01g68500|transcript|LOC_Os01g68500.1|protein_coding||c.-4911A>T|||||4778|</td></tr>
<tr><td>A|upstream_gene_variant|MODIFIER|LOC_Os01g68500|LOC_Os01g68500|transcript|LOC_Os01g68500.1|protein_coding||c.-4923C>T|||||4790|</td></tr>
<tr><td>A|upstream_gene_variant|MODIFIER|LOC_Os01g68500|LOC_Os01g68500|transcript|LOC_Os01g68500.1|protein_coding||c.-5029G>T|||||4896|</td></tr>
<tr><td>A|upstream_gene_variant|MODIFIER|LOC_Os01g68500|LOC_Os01g68500|transcript|LOC_Os01g68500.1|protein_coding||c.-5060C>T|||||4927|</td></tr>
</table>
