# Supplementary figures and images for: Genome-wide association mapping combined with gene-based haplotype analysis identify a novel gene for shoot length in rice (Oryza sativa L.)
Source: Theor Appl Genet. 2023 Nov 20;136(12):251. doi: 10.1007/s00122-023-04497-6 (PMC10661777; doi:10.1007/s00122-023-04497-6)

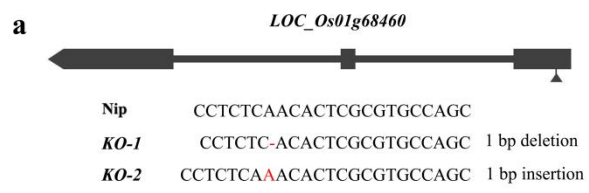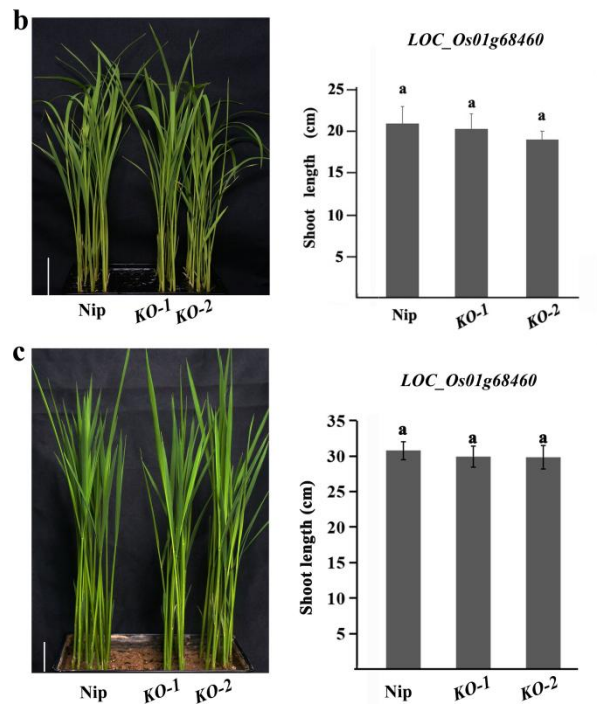

Figure S1

Supplement: Supplementary file 4 — Fig. S1 Mutation types and shoot lengths of the knockout transgenic lines of LOC_Os01g68460 a, The mutation types of LOC_Os01g68460. b and c The shoot length of the knockout transgenic (KO) lines of LOC_Os01g68460 were not significantly different from that of their wild-type Nipponbare (Nip) in nutrition solution (b) and soil culture (c). The same letter upon the histogram indicates no significant difference at P = 0.05 based on Duncan’s multiple range test. Scale bar, 3 cm (PDF 105 KB) [file 122_2023_4497_MOESM4_ESM.pdf]

**a**

| Haplotype                     | 38483533 <sup>#</sup> | 38517254 | 38538838 |
|-------------------------------|-----------------------|----------|----------|
| Hap1 <sup><i>qSL-1d</i></sup> | T                     | T        | T        |
| Hap2 <sup><i>qSL-1d</i></sup> | C                     | C        | A        |

**b**

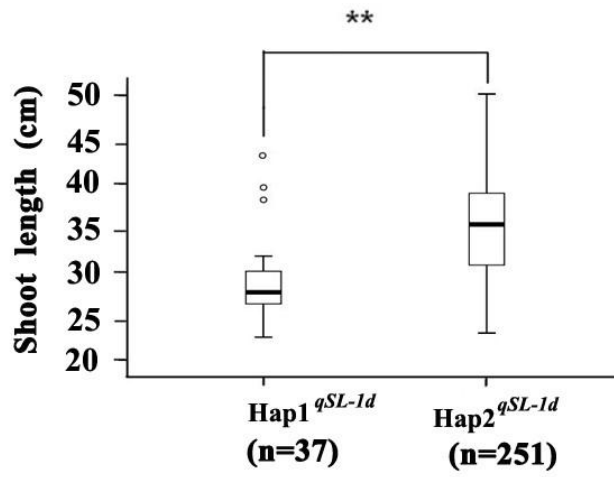

**c**

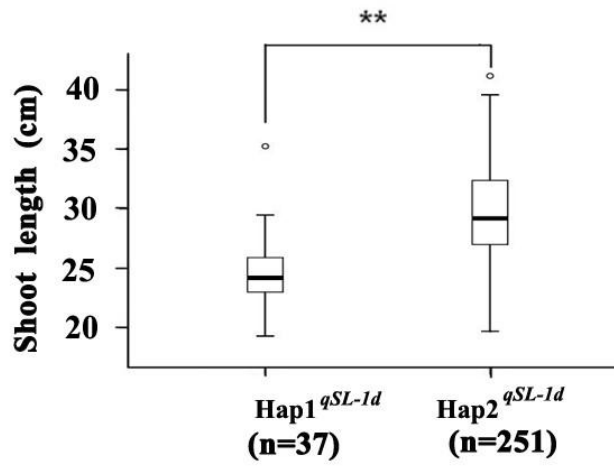

**d**

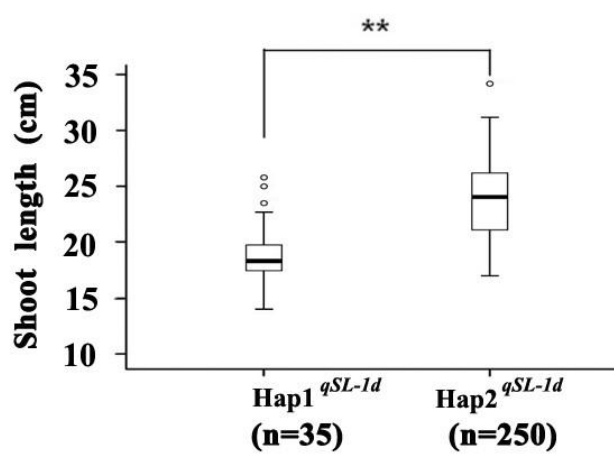

Figure S2

Supplement: Supplementary file 5 — Fig. S2 Differences in shoot length between the major haplotypes of qSL-1d. a The main haplotypes of qSL-1d. #The significant SNP position (bp); b–d Boxplots for shoot length based on the haplotypes of qSL-1d under GST (b), GSF (c) and DST (d). Numbers in parenthesis indicate the number of rice accessions with the haplotype. The black horizontal lines represent the median value; the upper side and lower side of the box represent the upper quartile and lower quartile, respectively; the whiskers represent the range of data, and small circle represents outlier. Double asterisk indicates the significant difference in shoot length at P = 0.01 based on t-test. (PDF 95 KB) [file 122_2023_4497_MOESM5_ESM.pdf]
