# Supplementary material for: Genome-wide association mapping combined with gene-based haplotype analysis identify a novel gene for shoot length in rice (Oryza sativa L.)
Source: Theor Appl Genet. 2023 Nov 20;136(12):251. doi: 10.1007/s00122-023-04497-6 (PMC10661777; doi:10.1007/s00122-023-04497-6)
Supplement: Supplementary file 6 — Fig. S3 The multiple sequence alignments between LOC_Os01g68500 and the four reported DUF538 proteins from other plants. The conserved amino acids are highlighted in dark color, and gaps are marked with dashes. The red line indicates the DUF538 domain (33-142 amino acids) of LOC_Os01g68500 protein. The amino acid sequence of LOC_Os01g68500 protein was collected from the Rice Genome Annotation Project website (http://rice.Plantbiology.msu.edu/). The full-length protein sequences of SVB in Arabidopsis, Ptom.006G00815 in Populus, GL6 in Maize and Celosia DUF538 were downloaded from the corresponding references, respectively (Marks et al. 2009; Li et al. 2019; Li et al. 2023; Gholizadeh and Kohnehrouz 2013). The multiple sequence alignments were performed using MEGA6 and GeneDoc (PDF 233 KB) [file 122_2023_4497_MOESM6_ESM.pdf]

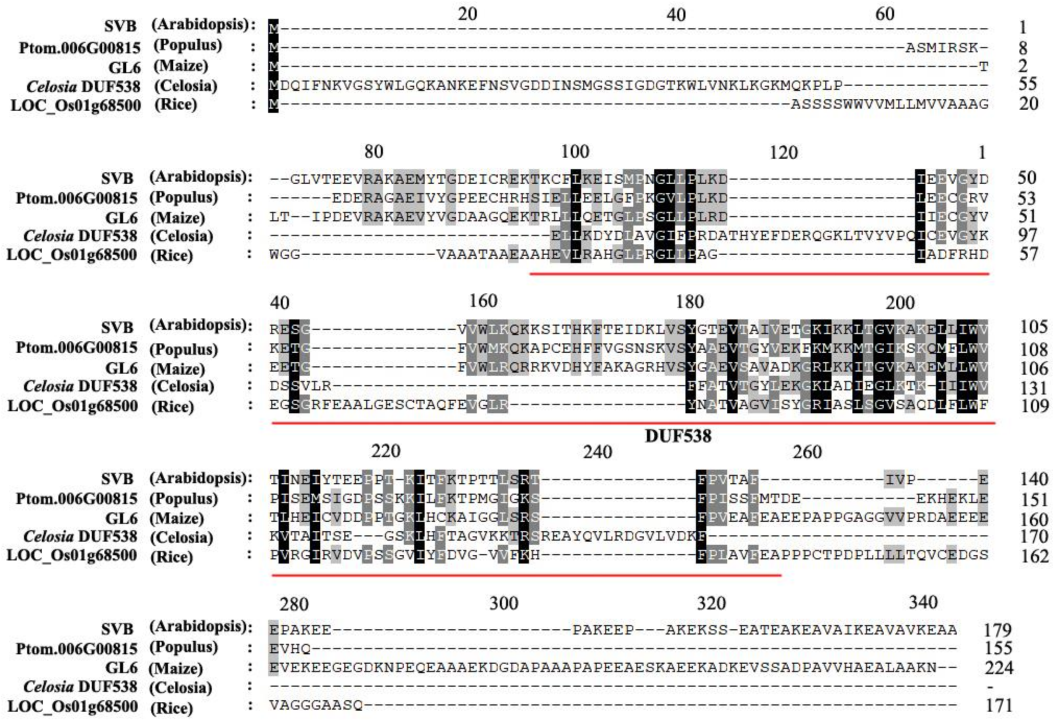

Figure S3
